# Retro-miRs: novel and functional miRNAs originating from mRNA retrotransposition

**DOI:** 10.1186/s13100-023-00301-w

**Published:** 2023-09-08

**Authors:** Rafael L. V. Mercuri, Helena B. Conceição, Gabriela D. A. Guardia, Gabriel Goldstein, Maria D. Vibranovski, Ludwig C. Hinske, Pedro A. F. Galante

**Affiliations:** 1 Hospital Sirio-Libanes, São Paulo, 01308-060 Brazil; 2 Interunidades Em Bioinformática, Universidade de São Paulo, São Paulo, 05508-000 Brazil; 3 Department of Genetics and Evolutionary Biology, University of São Paulo, São Paulo, Brazil; 4 School of Mathematical and Natural Sciences, New College of Interdisciplinary Arts and Sciences, Arizona State University, Tempe, AZ USA; 5 Institute for Digital Medicine/Clinic of Anaesthesiology, University of Augsburg, Augsburg, Germany

**Keywords:** miRNAs, Retrocopies, Gene origination, Gene expression, Cancer

## Abstract

**Background:**

Reverse-transcribed gene copies (retrocopies) have emerged as major sources of evolutionary novelty. MicroRNAs (miRNAs) are small and highly conserved RNA molecules that serve as key post-transcriptional regulators of gene expression. The origin and subsequent evolution of miRNAs have been addressed but not fully elucidated.

**Results:**

In this study, we performed a comprehensive investigation of miRNA origination through retroduplicated mRNA sequences (retro-miRs). We identified 17 retro-miRs that emerged from the mRNA retrocopies. Four of these retro-miRs had de novo origins within retrocopied sequences, while 13 retro-miRNAs were located within exon regions and duplicated along with their host mRNAs. We found that retro-miRs were primate-specific, including five retro-miRs conserved among all primates and two human-specific retro-miRs. All retro-miRs were expressed, with predicted and experimentally validated target genes except miR-10527. Notably, the target genes of retro-miRs are involved in key biological processes such as metabolic processes, cell signaling, and regulation of neurotransmitters in the central nervous system. Additionally, we found that these retro-miRs play a potential oncogenic role in cancer by targeting key cancer genes and are overexpressed in several cancer types, including liver hepatocellular carcinoma and stomach adenocarcinoma.

**Conclusions:**

Our findings demonstrated that mRNA retrotransposition is a key mechanism for the generation of novel miRNAs (retro-miRs) in primates. These retro-miRs are expressed, conserved, have target genes with important cellular functions, and play important roles in cancer.

**Supplementary Information:**

The online version contains supplementary material available at 10.1186/s13100-023-00301-w.

## Background

MicroRNAs (miRNAs) are small non-protein-coding RNAs (approximately 22 bp) with a key function of regulating gene expression at the post-transcriptional level [1]. Advances in our understanding of miRNA biology have revealed hundreds of miRNA genes that are widespread throughout the animal kingdom [2]. The biogenesis of miRNAs involves several coordinated processing steps resulting in the incorporation of mature miRNAs into the RNA-induced silencing complex (RISC) [3]. miRNAs act in gene regulation mechanisms by targeting the 3’ untranslated region (UTR) of protein-coding genes leading to translational repression or mRNA degradation [4]. Additionally, miRNAs have been shown to act as gene regulators very early on in the evolution of animals and have complex evolutionary patterns that differ from those of other genetic sequences [5].

Remarkably, it has been shown that the genome incorporation of novel miRNAs is a pivotal and instrumental step in the evolution of organismal complexity [5]. It is clear that the expression of miRNAs modulates the transcript variability of their target coding genes, fine-tuning the protein molecules produced by the coding gene, and therefore, conferring robustness to cellular pathways and gene networks. Consequently, the robustness of gene expression may have contributed to a decrease in phenotypic variation, maintaining an invariant phenotype despite endogenous and exogenous perturbations. This increased the heritability of species-specific traits [6].

For the emergence of new miRNA sequences, it is a fundamental prerequisite that the novel miRNA gene is transcribed from a genomic locus prone to produce an RNA fold recognizable by the miRNA processing machinery [7]. Essentially, there are two distinct molecular mechanisms capable of generating a novel miRNA: i) duplication of a pre-existing miRNA, followed by sub-or neofunctionalization [8], and ii) de novo origination of miRNAs [9]. While the former is majorly mediated by a local or full genome duplication [10], the latter (de novo) miRNA origination has a bias to occur in transcribed regions, frequently from introns [11, 12]) or is mediated by transposable elements [13] in the duplication of non-coding RNAs [14–16].

One overlooked source of novel miRNAs is the copies of processed mRNA, which are also known as retrocopies. Retrocopies are mRNA copies (of protein-coding genes) that have been reverse-transcribed to cDNA sequences and re-inserted into the genome in a process known as retrotransposition [17]. Until recently, retrocopies have been referred to as processed pseudogenes, but an increasing body of evidence suggests that a large fraction of retrocopies is functional [18–20]. Retrocopies are a major source of genetic novelty because they create novel genes [21], regulatory regions [22] and other noncoding genes, including miRNAs [15]. Specifically, this previous work [15] showed that two miRNAs (hsa-miR-220 and hsa-miR-492) lie within retrocopies of protein-coding genes and suggest that these retroduplicates are good "miRNA incubators''. Surprisingly, almost two decades later, no further investigations have been conducted on this issue.

In this study, we comprehensively investigated the contribution of retrocopies as a source of novel miRNAs (retrocopy-derived miRNAs (retro-miRs)) in the human genome. To accomplish this, we used an extensive range of databases concerning genomic annotations, sequence conservation, expression in healthy and cancerous tissues, information about miRNA target genes, and a complete set of bioinformatics tools. In summary, we identified 17 primate-specific retro-miRNAs and investigated their conservation, expression, gene targets, and putative functions in cancer.

## Results

### Finding miRNAs originated by mRNA retrotransposition events

**Fig. 1:**
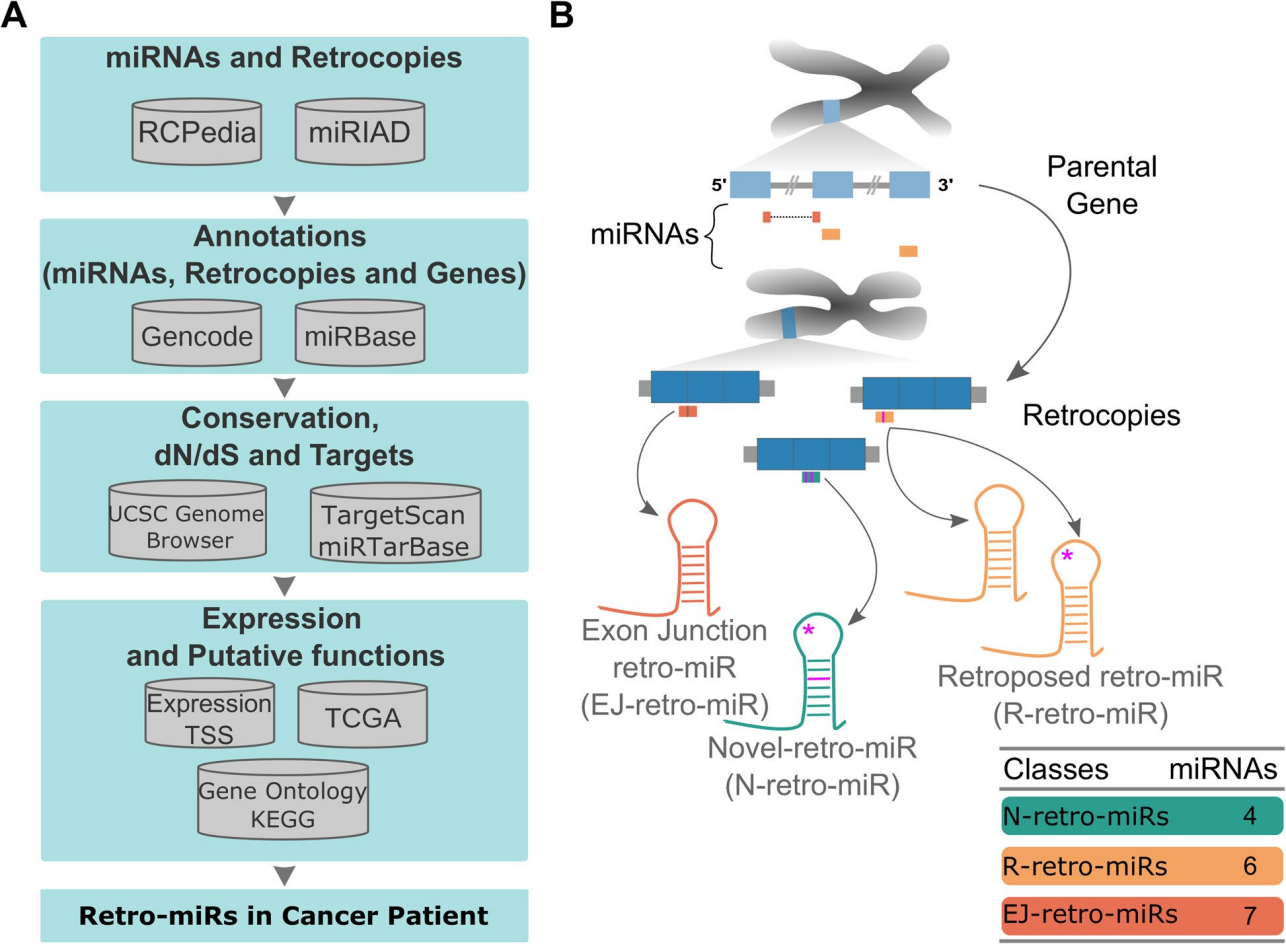
Screening microRNAs (miRNAs) originated by retrotransposition of protein-coding genes. **A** Summary of databases, datasets, and strategy used to identify retrocopy-derived miRNAs (retro-miRs). **B** Schematic representation of a parental gene, exonic miRNAs, retrocopies and retro-miRs. All three classes of retro-miRs, exon-junction retro-miRs (EJR-miR, in red), novel retro-miRs (NR-miR, in green), and retroposed retro-miRs (RR-miR, in orange) are represented. Tiny purple bars in retro-miR representations and harpins represent mismatches between them and their respective region in the parental gene

To identify miRNAs originating from mRNA retrocopies (retro-miRs), we developed a set of local pipelines and surveyed several databases (Fig. 1A). Briefly, we constructed and used a set of computational algorithms to assess and integrate information from three databases: miRBase, the reference database for information on several miRNAs [23]; miRIAD, a database containing annotation and further data on intragenic miRNAs and their host genes [24, 25]; and RCPedia, a database of retrocopies present in humans and other species [26]). Further information about the investigated genes (retrocopies and miRNAs), such as their conservation, expression, miRNA targets, and functional information, was retrieved from other databases (e.g., TargetScan [27], miRTarBase [28], FANTOM phase 5 [29, 30], and The Cancer Genome Atlas (TCGA) and locally processed (Fig. 1A). Further details are available in the Methodology section.

Using this strategy, we identified 17 retro-miRs in retrocopied sequences. These miRNAs were grouped into three distinct classes: retroposed (R-retro-miRs), exon junction (EJ)-retro-miRs, and novel retro-miRs (N-retro-miRs) (Fig. 1B). Six R-retro-miRs (35.3%; miR-4444–2, miR-1244–2, miR-1244–3, miR-1244–4, miR-1244–5, and miR-1244–6) are retroduplications of exonic miRNAs already present in their parental gene sequences (Fig. 1B, Supplementary Figure S1). These include four R-retro-miRs that are fully identical to their parental miRNAs and two others that have variations in their precursor stem, including one retro-miR with variations in the mature region (outer seed region). We found seven EJ-retro-miRs (41.2%; miR-492, miR-622, miR-4426, miR-4426–1, miR-3654, miR-3654–1, and miR-10527), which were also in the exonic region of their parental genes but spanned two exons (Fig. 1B, Supplementary Figure S2). Interestingly, none of these miRNAs have been annotated in the parental gene region, likely because they are split by an intronic sequence. The last retro-miR class, N-retro-miR, comprised four miRNAs (23.5%; miR-4788, miR-7161, miR-4468, and miR-572) that had a de novo origin in the retrocopied sequences because of the occurrence of mutations during evolution (Fig. 1B, Supplementary Figure S3). Upon checking the equivalent region in the parental gene, no evidence of a stem loop, that is, a putative miRNA, was found (Supplementary Figure S4).

Next, we investigated the characteristics of retrocopies containing retro-miRs. First, we found that the six R-retro-miRs (miRNAs present in both retrocopy and parental genes) originated from six distinct retroduplication events. Interestingly, these retroduplication events are from two parental genes, PTMA (five retrocopies with retro-miRs) and HNRNPA3 (one retrocopy with retro-miR) (Supplementary Table S1), which have been highly retroduplicated in the human genome (PTMA, 13 retrocopies; HNRNPA3, 17 retrocopies, Supplementary Table S2). Regarding the remaining retro-miRs, we found that: i) the seven EJ-retro-miRs originated from seven distinct retrocopies of five parental genes, and ii) the four N-retro-miRs originated from four distinct retrocopies of four parental genes (Supplementary Tables S1 and S2).

These results led us to investigate the events of chromosomal duplication of genes containing exonic miRNAs, in addition to the retro-miRs we identified. Briefly, we found that among the 19,768 protein-coding genes, 144 contained exonic miRNAs, of which 17 were duplicated by retrotransposition and 24 by DNA-mediated duplication. Interestingly, only two DNA-based duplications contained miRNAs in their duplicates (Supplementary Table S3). Taken together, these results suggest that retroduplication is a common pathway for the origin of novel miRNAs.

### Characteristics and conservation of retro-miR events

**Fig. 2:**
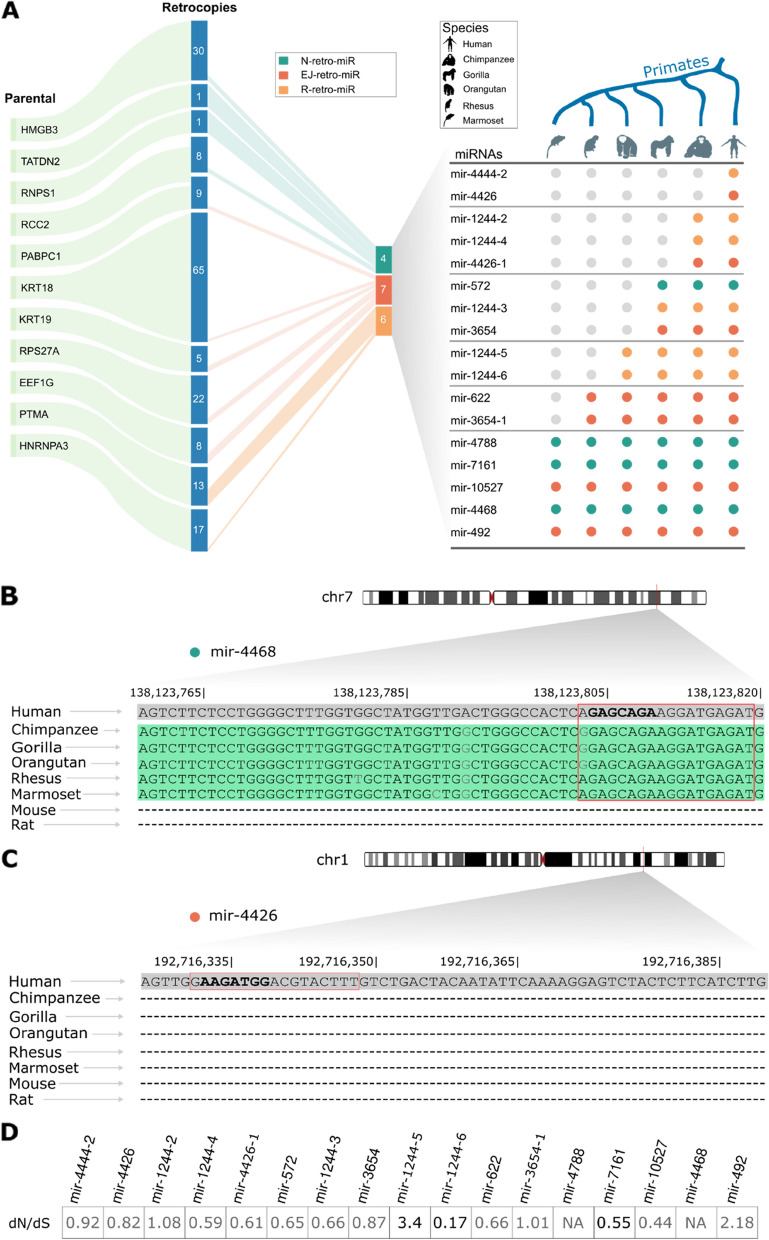
Retrocopy-derived miRNAs (retro-miRs) originated in the primate lineage and have evolved under a functional constraint. **A** We identified 17 retro-miRs that originated from 11 parental genes (left side). On the right side, we show the conservation of these retro-miRs in human, chimpanzee, gorilla, orangutan, rhesus, and marmoset genomes. Gray markings indicate the absence of the sequence in that organism. **B** An alignment between primate, mouse, and rat genomes of a retro-miR (miR-4468, a retro-miR conserved among all primates) sequence. The regions conserved are highlighted in green (matches in black; mismatches in light gray), and “-” indicates a lack of conservation. The red boxes represent the mature mRNA sequence, and the bold sequences are the miRNA seed region (2nd to 8th nt). **C** Alignment of a human-specific retro-miR (miR-4426). Lack of conservation is presented by “-”. The red boxes represent the mature sequence, and the bold sequences are the miRNA seed region (2nd to 8th nt). **D** Retro-miRs in retrocopies with a dN/dS value significantly different than 1 (*p*-value <  = 0.05, likelihood ratio test) under purifying (dN/dS < 1) or positive (dN/dS > 1) selection are highlighted in black, while those with non-significant (or non-evaluated “NA”) dN/dS values are shown in gray

To trace the evolutionary origin of each retrocopy and its retro-miRs, we used a homology-based approach (see Materials and Methods) to determine their conservation (Fig. 2A). First, this analysis revealed that all these retrocopies and their miRNAs originated in the primate lineage (Supplementary Table S4) and, in agreement with the literature on retrocopies, are primate-specific [19]. By correlating the ages of retro-miRs with their classes, we observed that the oldest events (conserved in all primates) were enriched in de novo N-retro-miRs. EJ-retro-miRs were spread across all ages, and R-retro-miRs were only present in great apes and up (Fig. 2A).

These results prompted us to investigate whether retrocopies containing retro-miRs evolved under functional constraints (purifying or positive selection) or under neutral conditions. To assess this, we used retrocopy-predicted coding regions to quantify the ratio of the number of nonsynonymous substitutions per nonsynonymous site to the number of synonymous substitutions per synonymous site (dN/dS). We found three retrocopies under neutral selection (dN/dS ~ 1, retrocopies containing retro-miR-4444–2, retro-miR-1244–2, and retro-miR-3654–1) and 12 retrocopies with a dN/dS ratio different from 1 (potentially under functional constraints) (Fig. 2D). However, only for three of these retrocopies, we confirmed a dN/dS value significantly different than 1 (*p*-value <  = 0.05, likelihood ratio test), with two retrocopies under purifying selection (retrocopies containing retro-miR-1244–6 and retro-miR-7161) and one retrocopy (containing retro-mir-1244–5) under positive selection (Fig. 2D, Supplementary Table S5).

Taken together, these results indicate that retro-miR originated at different evolutionary time points during primate evolution, with some originating early in the primate lineage (e.g., mir-492 and mir-4468) and others more recently (e.g., mir-4426). Additionally, considering the recent insertion of these retrocopies and retro-miRs into the evolutionary history of humans, we observed a tendency to purify evolutionary pressure on them.

### Expression of retro-miRs in normal tissues

**Fig. 3:**
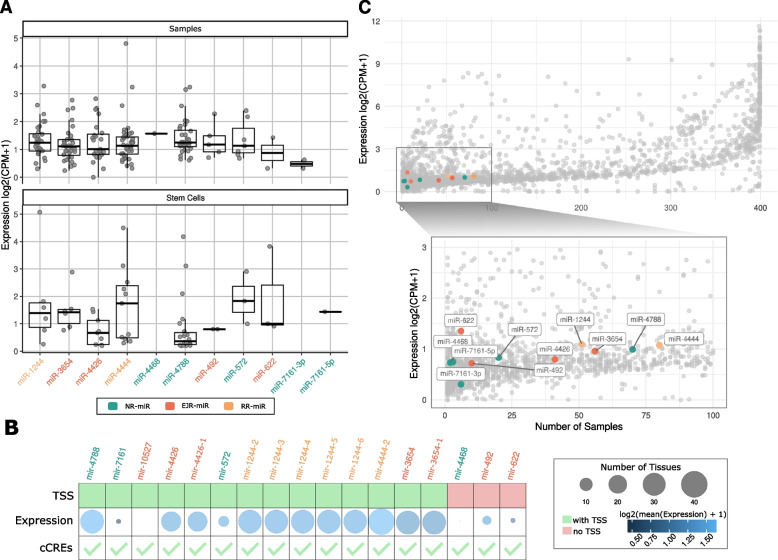
Retrocopy-derived miRNAs (retro-miRs) are expressed in normal tissues and cell lines. **A** Expression (median per tissue) of retro-miRs in normal samples (404 individuals, 32 tissues) and cell lines (48 samples of stem cells) from the FANTOM phase 5 [29, 30]. **B** Table showing the presence (in green) or absence (in red) of a transcription start site (TSS) upstream of the retro-miR, a bubble plot representing the intensity of expression of retro-miRs, as well as the number of tissues in which they were expressed and presence of ENCODE Candidate Cis-Regulatory Elements (cCREs) nearby retrocopies hosting the retro-miRs. **C** Expression level per number of tissues of all known human miRNAs. Retro-miRs are marked in colors, while all other miRNAs are represented by gray dots (expression based on samples from FANTOM phase 5 [29, 30]). Only expressed retro-miRs are shown in **A** and **C**

After dissecting the genomic features of retro-miRs, we attempted to identify their transcription and target genes, which are fundamental features of functional miRNAs. We examined evidence of retro-miR transcription using short RNA sequencing (RNA-seq) [29] and CAGE data [30]. All retro-miRNAs (except retro-miR-10527) were expressed in normal samples (404 individuals, 32 tissues) and/or cell lines (48 cell lines) (Fig. 3A). Notably, we observed distinct expression patterns of retro-miRs in different tissues and cell lines. Interestingly, a novel retro-miR (mir-4788) was highly expressed (the highest median expression among all retro-miRs) in normal samples. Conversely, in stem cells, miR-4788 showed the lowest (median) expression.

Next, to further support the true expression of these loci containing retro-miRs, we evaluated the presence of a transcription start site (TSS) (up to 4 Kbp) upstream of their 5' end using CAGE sequencing and Cis-regulatory elements (CREs), using ENCODE data. The results confirmed TSSs for 82% (14/17) and CREs for all (100%) of the retro-miRs (Fig. 3B). Additionally, we also found expressions for all retrocopies hosting these retro-miRs (Supplementary Figure S5). In agreement with the RNA-seq data, retro-miRs with a defined TSS had, in general, higher expression and were more broadly expressed in different tissues than retro-miRs without a TSS (Fig. 3B).

Next, we compared the expression of retro-miRs with that of all the other annotated miRNAs (Supplementary Table S6). Figure 3C shows that retro-miRs were in the middle of the first quartile in terms of expression levels and number of tissues expressed. Additionally, Supplementary Table S7 provides additional experimental confirmation of the expression of these retro-miRs, including confirmation through Real-Time quantitative PCR and additional short-RNA-seq datasets. Together, we observed that all retro-miRs had similar expression levels, which is expected for genes (miRNAs) that have recently evolved. Additionally, N-retro-miRs were among the miRNAs expressed in fewer tissues, a pattern that is expected for novel genes (miRNAs).

**Fig. 4:**
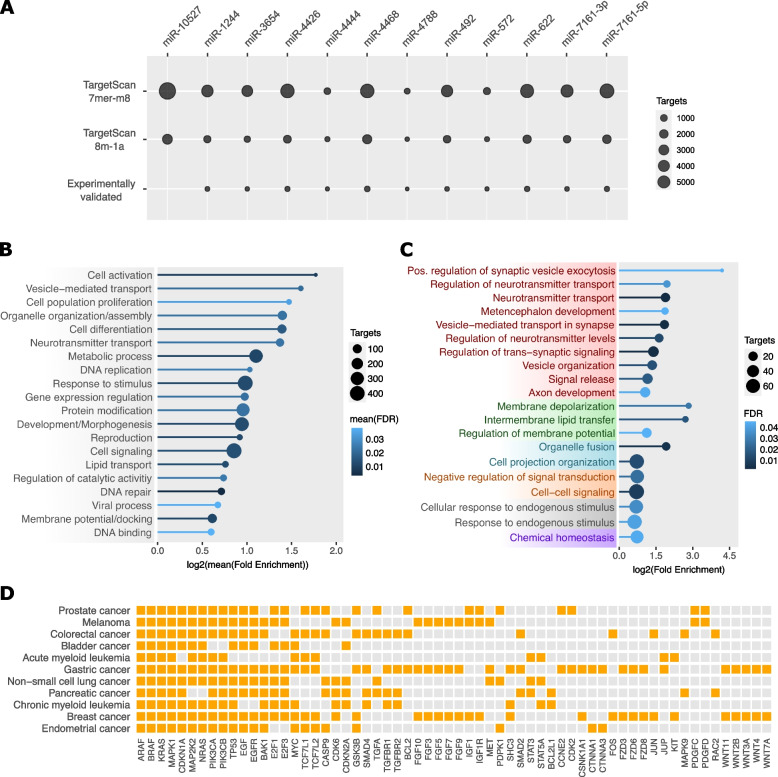
Retrocopy-derived miRNAs (retro-miRs) target genes are involved in key cell functions, including regulation of neural systems and have oncogenic roles. **A** Number of predicted (7mer-m8 and 8mer-1a) and experimentally validated target genes for retro-miRs. **B** Gene Ontology enrichment (Biological Processes) analysis of gene targets from retro-miRs. **C** Gene Ontology enrichment (Biological Processes) analysis of target genes of miR-572 and miR-4788. **D** Cancer-related KEGG pathways enriched in target genes of retro-miRs. Only enriched (> 1.5) and significant processes and pathways (FDR < 0.05) are shown

In a further investigation, we sought to identify the set of genes targeted by retro-miRs, a *sine qua non* feature of functional miRNAs [27]. Notably, we identified target genes for all retro-miRs using TargetScan (7mer-m8 and 8mer) and TargetScan (8mer), and experimentally validated the targets from mirTarBase (Fig. 4A; Supplementary Table S8). Only one retro-miR (miR-10527) had no experimentally validated targets, which is in accordance with our previous results (Fig. 3), where we reported no expression of miR-10527. Next, to evaluate the global functions of these retro-miR targets, we performed a Gene Ontology investigation of their biological processes. We found that these target genes function in key cellular processes, including the regulation of transcription, DNA replication, cell proliferation and differentiation, and DNA binding (Fig. 4B; *p*-value < 0.05; fold enrichment > 1.5). Next, we separately examined each set of targets for all retro-miRs (Supplementary Figure S6) and identified two sets of target genes. The two novel retro-miRs (miR-4788 and miR-572) had gene targets enriched in neural biological processes, particularly those involved in the regulation of neurotransmitters and synapses (Fig. 4C, Supplementary Figure S6). In addition, miR-4788 is conserved from humans to marmosets, and miR-572 is conserved from humans to gorillas. This finding may be implicated in the phenomenon of species-specific trait heritability of miRNAs.

We also assessed whether the targets of the retro-miRs were enriched in specific Kyoto Encyclopedia of Genes and Genomes (KEGG) pathways. Remarkably, we found enrichment of several cancer-specific pathways, including breast, colorectal, gastric, pancreatic, prostate, and lung cancers (Fig. 4D), in addition to more general cancer-related pathways, such as P53, ErbB, and MAPK signaling, as well as resistance to platinum drugs and EGFR tyrosine kinase inhibitors (Supplementary Table S9). In this analysis, several known cancer genes, including *BRAF*, *KRAS*, *EGFR*, and *TP53* emerged as targets of retro-miRs.

Taken together, the expression and gene target investigation revealed that retro-miRs (except miR-10527) have reliable expression and validated gene targets, which are the two fundamental characteristics of functional miRNAs. We also observed that retro-miRs target genes involved in fundamental cell processes (e.g., cell growth, regulation of transcription, and post-transcriptional regulation) and that two novel retro-miRs (miR-572 and miR-4788) regulate genes related to neuronal functions. Additionally, we observed that these retro-miRNAs targeted key cancer genes, suggesting that they play oncogenic roles in cancer tissues.

### Retro-miRs are highly or majorly transcribed in cancer tissues

**Fig. 5:**
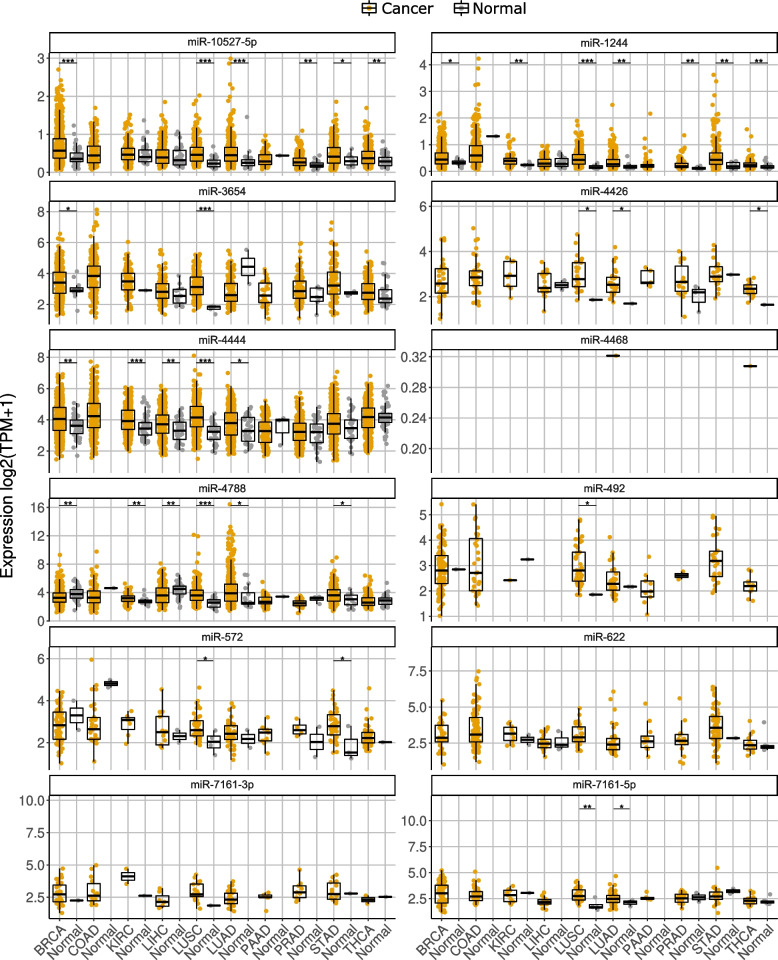
Retrocopy-derived miRNAs (retro-miRs) are highly expressed in cancer and some retro-miRs present a cancer-specific expression. Expression level of (mature) retro-miRs in different types of tumors (BRCA = Breast cancer, COAD = Colon adenocarcinoma, KIRC = Kidney renal clear cell carcinoma, LIHC = Liver hepatocellular carcinoma, LUSC = Lung squamous cell carcinoma, LUAD = Lung adenocarcinoma, PAAD = Pancreatic adenocarcinoma, PRAD = Prostate adenocarcinoma, STAD = Stomach adenocarcinoma, THCA = Thyroid carcinoma) and their respective normal samples. Wilcoxon test Tumor x Healthy samples: *p*-value <  = 0.05 and >  = 0.01 (*); < 0.01 and >  = 0.0001 (**); < 0.0001 (***)

Our previous results (Fig. 4D) prompted us to study the expression of these retro-miRs in cancer tissues. Using short RNA-seq data available in TCGA [31], we sought the expression of mature retro-miRs in 10 solid tumors. Their normal counterpart tissues were used as controls due to the relatively limited number of normal samples. Overall, this investigation revealed that retro-miRs have distinct and high expression levels in cancer (Fig. 5). Specifically, miR-10527-5p, miR-3654, miR-4444, miR-4788, and miR-1244 were highly expressed in cancer tissues but were also expressed in several normal tissues (Fig. 5). Interestingly, miR-10527-5p was highly expressed in tumors, although it was not expressed in normal samples or cell lines (Fig. 3). Remarkably, five retro-miRs emerged as putative tumor biomarkers because they presented a very low (or absent) expression in normal samples and consistent expression in cancer. Notably, two retro-miRs (miR-622 and miR-492) were exclusively expressed in seven cancer types each: BRCA (miR-622), COAD (miR-622 and miR-492), LUAD (miR-622), PAAD (miR-622 and miR-492), PRAD (miR-622 and miR-492), STAD (miR-492), and THCA (miR-492) (Fig. 5). Two mature miRNAs from the same precursor (miR-7161), miR-7161-3p and miR-7161-5p, were also exclusively expressed in six cancer types: BRCA (miR-7161-3p and miR-7161-5p), COAD (miR-7161-3p), LUAD (miR-7161-3p), LIHC (miR-7161-5p), PAAD (miR-7161-3p and miR-7161-5p), and PRAD (miR-7161-3p). Finally, miR-4426 was found to be expressed exclusively in BRCA, COAD, KIRC, and PAAD.

Taken together, these results indicate that retro-miRs are highly expressed in cancer, with some retro-miRs presenting a cancer-specific expression pattern and thus, their potential as tumor biomarkers should be further studied.

### Overall survival based on groups of retro-miRs in TCGA samples

**Fig. 6:**
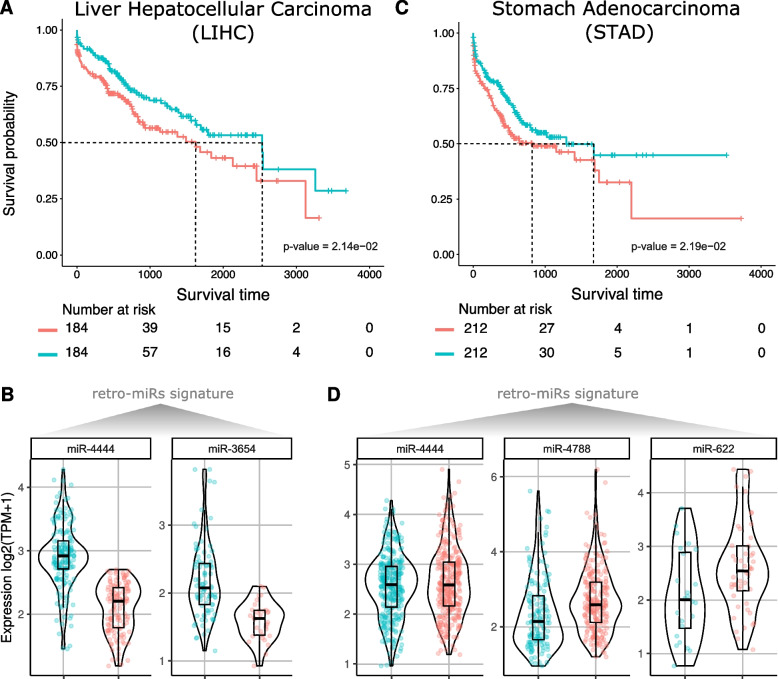
Prognostic value of retrocopy-derived miRNA (retro-miR) expression in liver hepatocellular carcinoma and stomach adenocarcinoma. **A** Kaplan–Meier plot showing the overall survival differences (log-rank test) between two groups of patients with liver hepatocellular carcinoma, stratified based on expression levels of retro-miRs: miR-4444–2 and miR-3654. **B** Violin plot of the expression of miR-4444 and miR-3654 in the groups with better and worse overall survival probability presented in **A**. **C** Kaplan–Meier plot showing the overall survival differences (log-rank test) between two groups of patients with stomach adenocarcinoma, stratified based on expression levels of retro-miRs: miR-4444, miR-4788, and miR-622. **D** Violin plot of the expression of miR-4444, miR-4788, and miR-622 in the groups with better and worse overall survival probability presented in **C**

Having confirmed the high and distinct expression of retro-miRs in cancer, we sought to determine whether these miRNAs have prognostic value in the overall survival of patients with cancer. To assess this, we used Reboot [32], an algorithm that identifies gene signatures (including those of miRNAs) associated with cancer patient prognosis. Using this strategy in approximately 8000 samples from 10 cancer types presenting with overall survival (OS) data, we found two signatures with prognostic value: a signature with two retro-miRs (Fig. 6A-B) in liver hepatocellular carcinoma (LICH) and another with three retro-miRs with prognostic value in STAD (Fig. 6C-D). In detail, Fig. 6A shows the overall survival curve of two sets of patients with LICH: patients with higher expression of miR-4444 and miR-3654 presented significantly (*p*-value < 0.0002) lower overall survival (e.g., overall survival of ~ 1650 days for 50% of patients) than patients with lower miR-4444 and miR-3654 expression (e.g., survival of ~ 2600 days (60% more) for 50% of patients). In patients with STAD, three retro-miRs (miR-622, miR-4788, and miR-4444) with high expression were associated with worse overall survival. Patients with a higher expression of these retro-miRs had a lower overall survival (~ 850 days for 50% of patients) than patients with a lower expression of these retro-miRs (~ 1300 days for 50% of patients). Thus, these data also show the prognostic value of these retro-miRs in LIHC and STAD and add a greater layer of functionality (which needs to be further explored) to these retrocopy-derived miRNAs.

## Discussion

MiRNAs are crucial regulators of gene expression and are instrumental elements in species evolution. Understanding the mechanisms underlying miRNA origination and their functions is essential for understanding the evolution of increasingly complex organisms and disease development, including cancer. Retrocopies, gene copies generated by the retroduplication of an mRNA molecule [18], are widespread in human and primate genomes and play a crucial role in shaping the genomic landscape and driving evolutionary innovation [17, 19]. In this study, we investigated retro-miRs, a class of miRNAs that emerge from the retroduplication of protein-coding genes (mRNAs). We explored retro-miR origination, conservation, expression, gene targets, and putative roles in cancer, including their potential to serve as tumor biomarkers and their association with overall cancer survival (prognostics).

MiRNAs are widespread in the animal kingdom [33]. The current miRBase release (22.1) presents 253 annotated (precursor) miRNAs in *Caenorhabditis elegans*, 882 miRNAs in chickens, 1,234 miRNAs in mice, and 1,917 miRNAs in humans. Interestingly, some miRNAs are widely conserved (e.g., mir-100, conserved in eumetazoans), but mammals, especially primates, present a high number of miRNAs that are associated with the emergence of novel phenotypes in the evolution of these species [5]. Briefly, miRNAs act primarily by buffering (reducing the variance in) the expression levels of their target genes. Finally, miRNAs ensure a stable gene expression mode against endogenous and exogenous factors, maintaining invariant (novel) phenotypes [5, 6]. A prerequisite for the origin of a novel miRNA is a transcribed genomic locus capable of producing an RNA fold that is recognizable by the miRNA processing machinery [1]. DNA duplication of pre-existing miRNAs, followed by their sub-functionalization or neofunctionalization, is a major source of novel miRNA genes [8]. However, miRNA origination from scratch (especially from intronic regions), duplication of non-coding RNAs (small nucleolar RNAs (snoRNAs) and transfer RNAs (tRNAs)), and retroduplicated elements (transposable elements) have also been reported [34]. Interestingly, Devor [15] demonstrated that two primate-specific miRNAs originated from retroduplicated genes (processed pseudogenes or retrocopies) and suggested that retrocopies are good miRNA incubators [15]. Conversely, even with the advent of genomics and bioinformatics, new investigations of miRNAs originating from retrocopies have not been conducted. This study fills this gap in the literature by identifying 17 novel miRNAs that originate from the retroduplication of protein-coding genes. Interestingly, these novel miRNAs are primate-specific, with three being shared only between humans and chimpanzees, and two are human-specific retro-miRs (Fig. 2). Surprisingly, some of these miRNAs (hsa-mir-572 and hsa-mir-622) have been studied for years but have never been found to originate from mRNA retroduplication. For example, the (retro-)miR-572 originated in RNPS1 retrocopy, RNPS1P1.

To function, an miRNA gene must be transcribed, folded, processed, and have gene targets [1]. Using short RNA sequencing data (and TSS definition) from tissues and cell lines, we confirmed that all retro-miRs (except miR-10527) were transcribed (Fig. 3). Curiously, miR-10527 shows expression in both cancer and normal tissues (Fig. 5). As expected for a set of genes (miRNAs) presenting distinct functions and under the control of different promoter regions, the expression profile of retro-miRs ranged from retro-miRs expressed in a few samples and tissues to others being highly expressed in more tissues (Fig. 3). However, it is important to acknowledge that when multiple precursor miRNAs (pre-miRNAs) have identical mature sequences (e.g., retro-miRs-4426, retro-miRs-1244, and retro-miRs-3654), there are limitations in precisely assigning the mature miRNAs to their corresponding pre-miRNAs [35]. In such cases, additional information about the primary retro-miRs loci is essential to confirm their transcription, as we have discovered in relation to their transcription start site (TSS) and cis-regulatory elements. Secondly, it is important to highlight that studies on exonic miRNAs have revealed negative correlation (mutually exclusive expression) between these miRNAs and their host genes (retrocopies or retrogenes) [36, 37], since Drosha processing of retro-miR (miRNA) will inhibit production of the host mRNA retrocopy/retrogene. Therefore, for retro-miRs, higher expression suggests their potential functionality relative to their hosts. Finally, we identified retro-miR gene targets. Notably, all retro-miRs had predicted and experimentally validated gene targets, except for miR-10527, which lacked a validated target. Gene Ontology analysis of the biological processes associated with retro-miR targets revealed genes involved in fundamental cellular processes and functions, including cell differentiation and signaling, DNA replication, gene expression regulation, and neurotransmitter transport (Fig. 4). Remarkably, two novel retro-miRs, miR-572 and miR-4788, have gene targets that are primarily involved in brain functions, such as nervous system development, regulation of neurotransmitter levels, neurotransmitter transport, and synapses. Therefore, because miRNAs provide robustness to their target gene regulatory networks, helping to maintain novel traits [5], these novel and primate-specific retro-miRs are promising candidates for further exploration in the context of key genes (miRNAs) to explain the evolution of the brain in humans and other primates.

Additionally, we found that retro-miRs target important cancer-related genes (Fig. 4D), prompting us to investigate their expression in cancer. First, we confirmed that retro-miRNAs were highly expressed in several cancer types (Fig. 5). Three retro-miRs (miR-572, miR-622, and miR-492) have been extensively studied in cancer. Studies have shown that miR-572 can act as both a tumor suppressor and oncogene, depending on the tissue and tumor type. Consistent with our findings (Fig. 5), miR-572 has been shown to have low expression levels (potentially acting as a tumor suppressor) in breast [38] and colorectal tumors [39]. On the other hand, the oncogenic role of miR-572 has been confirmed in lung tumors [40], hepatocellular carcinoma [41], and kidney cancer [42], which is also consistent with our results (Fig. 5). However, the importance of retro-miRs in other tumors (Fig. 5) remains underexplored. For miR-622, the literature also reports that this retro-miR can act as both a tumor suppressor and an onco-miR in several cancers, such as breast cancer, glioma, colorectal cancer, hepatocellular carcinoma (HCC), lung cancer, gastric cancer (GC), melanoma, ovarian carcinoma, prostate cancer, and pancreatic cancer [43], which is in agreement with our results (Fig. 5). Similar to the other two retro-miRs, the literature reports that miR-492 acts as both a tumor suppressor and an oncogene. Consistent with our expression data, this retro-miR is upregulated in metastatic hepatoblastoma [44], lung cancer [45], and stomach cancer [46]. However, for other retro-miRs (Fig. 5), few or no investigations have been performed on tumor tissues.

Finally, we also found that the expression (signature) of two sets of retro-miRs was associated with the overall survival in two cancer types: LIHC and STAD (Fig. 6). These results require further investigation.

## Conclusions

In conclusion, we conducted a comprehensive study of miRNAs derived from retroduplicated protein-coding genes in the human genome. Our findings demonstrated that these retro-miRs are expressed in a conserved environment, have regulatory targets in fundamental cellular processes, including those in the nervous system, and exhibit differential expression and functional roles in various cancer types, similar to other known miRNAs. Therefore, we do not claim that retro-miRs are a special class of miRNAs. Overall, our study sheds light on the role of mRNA retrotransposition in generating genetic novelties, such as miRNAs, and highlights the potential for future investigations into the function of retrocopies in humans and other organisms.

## Materials and methods

### Identification of retro-miRs derived from retrocopies

To identify the retro-miRs, we followed these steps: i) we used mirBase (http://www.mirbase.org/) as an annotation source for miRNA characteristics, such as their genomic position and gene name; ii) our dataset of retrocopied genes was obtained from the RCPedia database [26], which contains information on retrocopies (e.g., genomic location, sequences, expression, and conservation) and their parental genes. To convert the retrocopies’ genomic coordinates from hg19 to hg38 we used the LiftOver online tool (https://genome.ucsc.edu/cgi-bin/hgLiftOver). Subsequently, we selected miRNAs from miRBase whose genomic coordinates overlapped with those of the retrocopies using BedTools v2.26.0 (with default parameters and a minimum overlap of 5 bp) [47]. We manually removed the data of those miRNAs that originated from the insertion of a mobile element into the retrocopies. To assess the colocalization (exonic, intronic, and intergenic) of miRNAs and protein-coding genes, we used the miRIAD database [24, 25]. Additionally, we selected annotated exonic miRNAs of coding genes whose retrocopies and retro-miRs were already identified. We aligned the mature miRNA sequences from these candidates against the respective retrocopies of the coding genes using Clustal Omega available at EMBL (https://www.ebi.ac.uk/Tools/msa/clustalo/). To evaluate the formation of secondary structures, we used RNAFold version 2.4.17 [48]. When a secondary structure was formed, the candidates were considered retro-miRs.

### DNA-mediated duplication

To identify duplicated genes, we used transcripts annotated in GENCODE v32. We used tblastn 2.7.1 + [49] with default parameters to align the amino acid sequences of all transcripts against their nucleotide sequences. We considered that a gene has a DNA-mediated duplication if it has at least one alignment besides its own nucleotide transcript with a score that is divided by the score of its original nucleotide transcript and is greater than or equal to 0.7. Additionally, we searched for miRNAs in these duplicated genes and duplications of these miRNAs in the duplicated genes using the miRNA genomic coordinates provided by miRBase.

### Classification of retro-miRs

To classify retro-miRs into R-retro-miRs, EJ-retro-miRs, and N-retro-miRs, we aligned the sequences of retro-miRs against the reference genome (GRCh38) using the BLAT tool (https://genome.ucsc.edu). For each retro-miR, we investigated the alignment pattern with the parental retrocopy gene. Based on this, three major patterns emerged: (I) retro-miRs, which are exact copies of the exonic miRNA of the parental sequence (R-retro-miRs); (II) retro-miRs originating from the exon-exon junction (EJ-retro-miRs); and (III) retro-miRs originating from mutations (> 1 bp) in relation to the parental gene in the mature sequence (N-retro-miRs).

### Conservation of retro-miRs among primates

The conservation of retro-miRs among primate genomes was determined using MultiZAling, which is available in the Genome Browser (https://genome.ucsc.edu/index.html). We compared the sequences of miRNAs annotated in humans with those of the genomes of chimpanzee (Pan_tro 3.0), gorilla (GSMRT3), orangutan (WUGSC 2.0.2), rhesus (BCM Mmul_8.0.1), and marmoset (WUGSC 3.2). We considered a retro-miR to be conserved if its sequence identity with homologous sequences in these genomes was greater than 80%.

### dN/dS analysis

To measure the selective pressure acting on retrocopies containing retro-miRs, we calculated the ratio of nonsynonymous substitutions (dN) to synonymous substitutions (dS). This calculation was performed between retrocopies and their parental genes (in the case of human-specific retrocopies) or between retrocopies in humans and their orthologs in chimpanzees. In order to conduct the dN/dS analysis, it was necessary to obtain the coding region. To achieve this, we extracted the nucleotide sequences of the retrocopies from the reference genome (GRCH38) and used ORFfinder [50] to obtain the longest predicted amino acid (aa) sequence. For human retrocopies (HNRNPA3P6 and RPS27AP5), we used BLAT [51] to align the predicted aa sequence against the human genome (version GRCh38/hg38) and retrieved the best match of the parental gene nucleotide sequence (paralog). For retrocopies with a primate ortholog (RPS27AP16, PTMAP2, PTMAP9, PTMAP4, RP11-371A22.1, RNPS1P1, PTMAP8, RP11-529H20.3, EEF1GP5, KRT18P27, TATDN2P2, HMGB3P13, RCC2P3, KRT19P2, and PABPC1P4), we aligned the predicted human aa sequence against the genome of chimpanzees (panTro6) with BLAT and retrieved the best match on the retrocopy ortholog. For both the paralogs and orthologs, we used ORFfinder to find the longest predicted aa sequence equivalent to the aa sequence of the human retrocopy. ClustalW [52] was used to align the aa sequence of the retrocopy and its corresponding paralog or ortholog. PAL2NAL (codeml; CodonFreq = 2, model = 0, Nsites = 0, fix_omega = 0, omega = 0.4) [53] was used to calculate the synonymous (dS) and non-synonymous (dN) substitution rates. The p-values of dN/dS were determined by comparison to a model assuming neutral evolution (fix_omega = 1, omega = 1 for paralogs, and omega = 0.5 for orthologs) with a likelihood-ratio test [LRT], *p*-value < 0.05, chi-sq. distribution. blastn [50] was used to calculate the identity between nucleotide sequence equivalents of the coding sequence (CDS).

### Retro-miR expression and TSS definition in normal and cancer tissues

Expression of these retro-miRs in normal tissues was analyzed using preprocessed data from the FANTOM Phase 5 database and described in [29, 30, 35]. Briefly, FANTOM contains samples from 404 samples from 32 tissues and 48 cell lines. These reads were aligned by BWA [54] and the expression level is calculated using the weighted number of reads that map at each locus and described in detail in [35]. We screened for a TSS on the expected transcription orientation (based on the miRNA direction) up to a distance of 4000 base pairs of the 5' end of the retro-miR. In addition, cCRE data was downloaded from UCSC Genome Browser and cCREs that lay within a 20,000 kb window from the loci containing retrocopy/pre-retro-miRs where accounted.

We also used preprocessed miRNA expression (short RNA-seq methodology) data from TCGA and the microRNA Tissue Expression Database (miTED) [55]. Briefly, the miTED used miRNA-Seq reads from TCGA which were aligned using Bowtie [56]. The aligned reads were quantified using miRDeep2 [57] and were attributed to the mature sequence, for more detail see [55]. TCGA miRNA quantification involves a multi-step pipeline [58]. Firstly, reads are demultiplexed and aligned against the reference genome (GRCh38) using BWA. The alignments are then compared to annotations from miRBase and UCSC to classify the reads as miRNAs or other small RNAs (snoRNAs, tRNAs, or rRNAs). Expression is reported only for read alignments that have an exact match to miRNAs, excluding mismatches or non-annotated miRNAs. The final processed data is available in two forms: raw read counts and counts normalized to reads per million (RPM). The TCGA data portal (https://portal.gdc.cancer.gov/) provides access to both types of expression data, including archives for isomiR and stem-loop expression. In both datasets, we normalized the data using transcripts per million (TPM). Expression was quantified only for the mature retro-miR sequences.

### Investigating the prognostic value of retro-miRs in 10 cancer types

To investigate the prognostic value of retro-miRs in cancer, short RNA-seq and patient's survival information were extracted from TCGA for the following tumor types: breast cancer (BRCA), colon adenocarcinoma (COAD), kidney renal clear cell carcinoma (KIRC), liver hepatocellular carcinoma (LIHC), lung squamous cell carcinoma (LUSC), lung adenocarcinoma (LUAD), pancreatic adenocarcinoma (PAAD), prostate adenocarcinoma (PRAD), stomach adenocarcinoma (STAD), and thyroid carcinoma (THCA). Only retro-miRNAs (TPM > 0) were considered for the analysis.

The association between the retro-miR expression signature (combined expression of two or more retro-miRs) and overall patient survival was assessed using Reboot [32] with default parameters. Reboot is an algorithm that identifies gene signatures associated with patients’ prognosis using a multivariate strategy (penalized Cox regression) combined with a bootstrap approach. When a signature is obtained, Reboot calculates a multi-gene score for each patient based on the resulting regression coefficients and corresponding gene expression levels. It then evaluates whether this score is predictive of patient survival using a log-rank test between patient groups presenting high versus low scores (cutoff at median score).

### Finding retro-miR target genes

To find out the target genes of retro-miRs, we ran TargetScan 7.0 with the set of 3′ UTR sequences of protein-coding genes provided by the Targetscan [27]. We selected only transcripts that had 8mer-1a or 7mer-m8 site types, or if these genes were represented in miRTarBase [28] MiRTarBase is a comprehensive resource for experimentally validated microRNA (miRNA) and target gene interactions. It provides a curated collection of miRNA-target interactions that have been experimentally validated through various experimental techniques, such as luciferase reporter assays, Western blot analysis, qRT-PCR, and more. Enrichment analyses of target genes in Gene Ontology (Biological Processes) and KEGG pathways were performed using ShinyGO v0.77 (http://bioinformatics.sdstate.edu/go/). Redundant Gene Ontology terms were removed using REVIGO (http://revigo.irb.hr/). Only Gene Ontology processes and KEGG pathways with fold enrichment > 1.5 and false discovery rate (FDR) < 0.05 were considered significant.

### Supplementary Information

**Additional file 1: Figure S1.** Exonic miRNAs already present in their parental gene sequences. **Figure S2.** Retro-miRs spanning two exons. **Figure S3.** Parental genes without exonic miRNA. **Figure S4.** Regions with and without evidence of a stem loop. **Figure S5.** Retro-miRs loci are transcribed and have nearby Cis Regulatory Elements. **Figure S6.** Gene ontology analyses of retro-miRs targets.

**Additional file 2: Table S1. **Retro-miRs information.

**Additional file 3: Table S2. **Retrocopied genes with exonic miRNAs.

**Additional file 4: Table S3. **Duplications of protein coding genes containing exonic miRNAs.

**Additional file 5: Table S4. **Conservation of retro-miRs and retrocopies.

**Additional file 6: Table S5. **Dn/Ds analysis.

**Additional file 7: Table S6. **Average of miRNAs in normal samples.

**Additional file 8: Table S7. **Experimental validation of retro-miRs.

**Additional file 9: Table S8. **Targets of the retro-miRs.

**Additional file 10: Table S9. **Targets of the retro-miRs.

## Data Availability

The datasets analyzed in this study are publicly available at the mirBase repository (http://www.mirbase.org); RCPedia repository (https://www.bioinfo.mochsl.org.br/rcpedia); miRIAD repository (https://www.miriad-database.org); GENCODE v32, (https://www.gencodegenes.org/human/release_32.html); MultiZAling, https://doi.org/10.1101/gr.229102; FANTOM phase 5 database, https://fantom.gsc.riken.jp/5/; The Cancer Genome Atlas (TCGA), https://portal.gdc.cancer.gov/; microRNA Tissue Expression Database (miTED), https://dianalab.e-ce.uth.gr/mited/; and genome sequences for these species: *Homo sapiens* (GRCh38/hg38 https://doi.org/10.1038/35057062), *Pan troglodytes* (Pan_tro-3.0 https://www.ncbi.nlm.nih.gov/data-hub/genome/GCF_000001515.7), *Gorilla gorilla gorilla* (GSMRT3 https://www.ncbi.nlm.nih.gov/data-hub/genome/GCA_900006655.1); *Pongo abelli* (WUGSC 2.0.2 https://www.ncbi.nlm.nih.gov/data-hub/genome/GCA_000001545.3); *Macaca mulatta* (BCM Mmul_8.0.1 https://www.ncbi.nlm.nih.gov/data-hub/genome/GCF_000772875.2/, and *Callithrix jacchus* (WUGSC 3.2 https://www.ncbi.nlm.nih.gov/data-hub/genome/GCF_000004665.1/).
