# Supplementary material for: Retro-miRs: novel and functional miRNAs originating from mRNA retrotransposition
Source: Mob DNA. 2023 Sep 8;14:12. doi: 10.1186/s13100-023-00301-w (PMC10486083; doi:10.1186/s13100-023-00301-w)
Supplement: Supplementary file 1 — Additional file 1: Figure S1. Exonic miRNAs already present in their parental gene sequences. Figure S2. Retro-miRs spanning two exons. Figure S3. Parental genes without exonic miRNA. Figure S4. Regions with and without evidence of a stem loop. Figure S5. Retro-miRs loci are transcribed and have nearby Cis Regulatory Elements. Figure S6. Gene ontology analyses of retro-miRs targets. [file 13100_2023_301_MOESM1_ESM.pdf]

Retro-miRs: Novel and functional miRNAs originated from mRNA retrotransposition

Rafael L. Mercuri[1,2,#], Helena B. Conceição[1,2,#], Gabriela D. A. Guardia[1], Gabriel Goldstein[3], Maria D. Vibranovski[3,4], Ludwig C. Hinske[5], Pedro A F Galante[1,*]

1 - Hospital Sirio-Libanes, São Paulo, 01308-060, Brazil.
2 - Interunidades em Bioinformática, Universidade de São Paulo, São Paulo 05508-000, Brazil.
3 - Department of Genetics and Evolutionary Biology, University of São Paulo, São Paulo, Brazil.
4 -School of Mathematical and Natural Sciences, New College of Interdisciplinary Arts and Sciences, Arizona State University, AZ, USA.
5 - Institute for Digital Medicine/Clinic of Anaesthesiology, University of Augsburg, Augsburg, Germany.
# These authors contributed equally.
* Corresponding author: Pedro A F Galante

SUPPLEMENTARY DATA

Figure S1

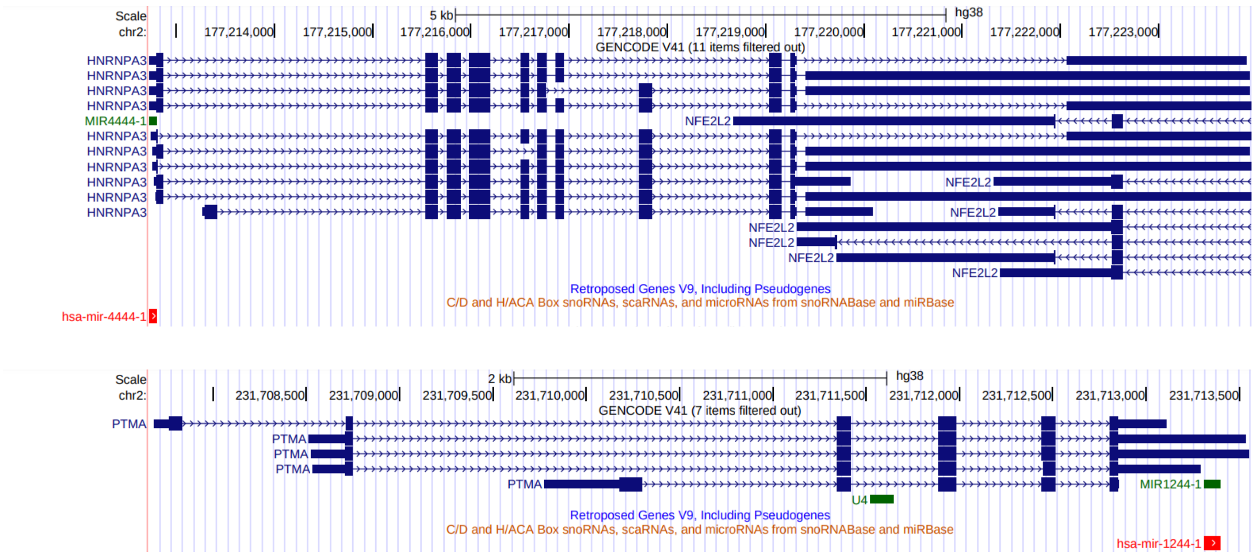

Figure S1. Exonic miRNAs already present in their parental gene sequences. Visualization of the Genome Browser in the parental gene region of the retro-miR (hsa-miR-4444; hsa-miR-1244) with the tracks of the gencode and miRNA turned on. The coding transcripts are represented in blue, while the miRNA sequences are highlighted in red.

Figure S2

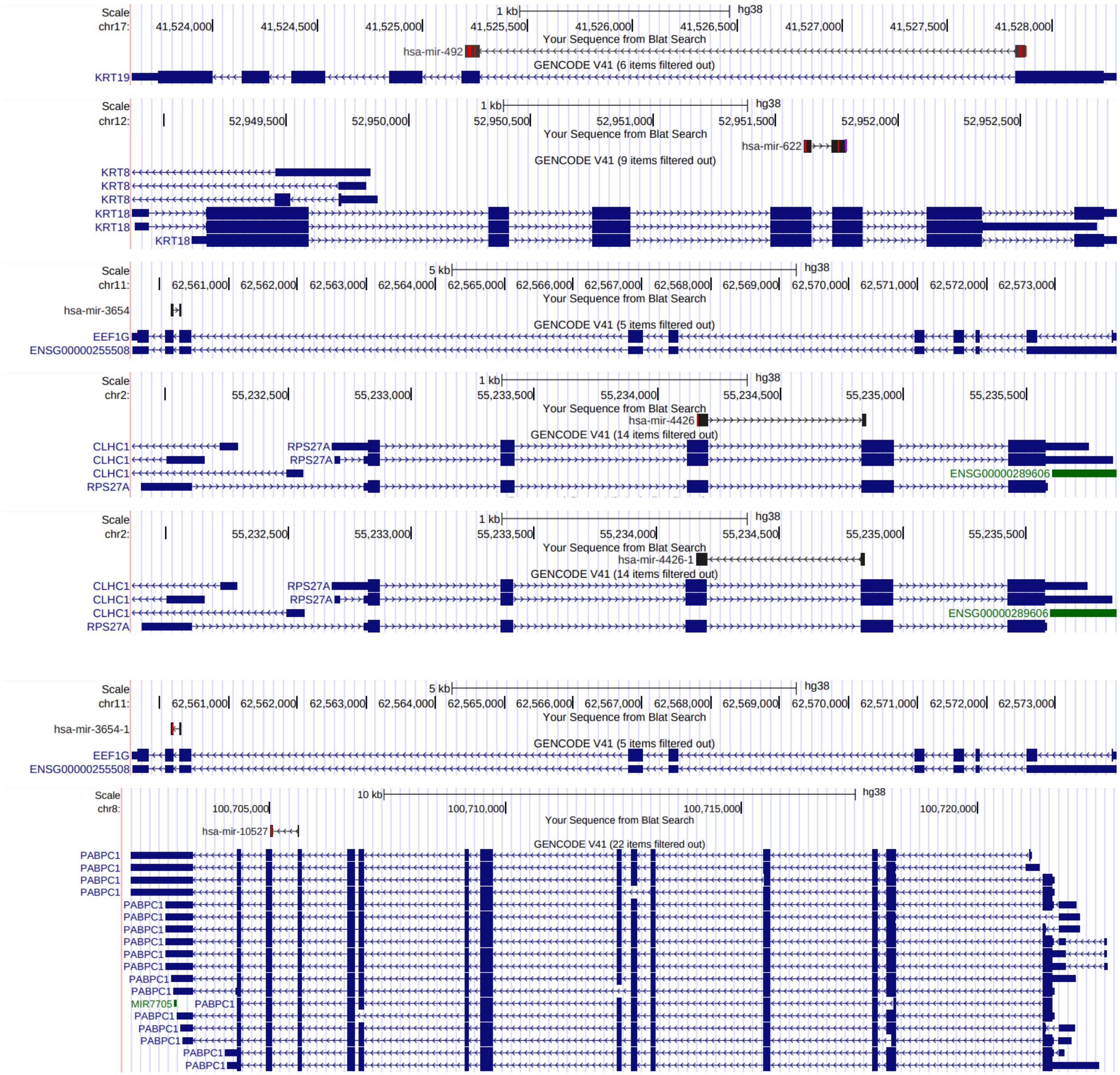

Figure S2. Retro-miRs spanning two exons. Visualization of the Genome Browser in the parental gene region of the retro-miR (hsa-miR-492; hsa-miR-622; hsa-miR-3654; hsa-miR-4426; hsa-miR-4426-1; hsa-miR-3654-1; hsa-miR-10527). The BLAT sequence search and GENCODE V41 track activated. The coding transcripts are represented in blue, while the sequence aligned in the human genome are present in the gray.

Figure S3

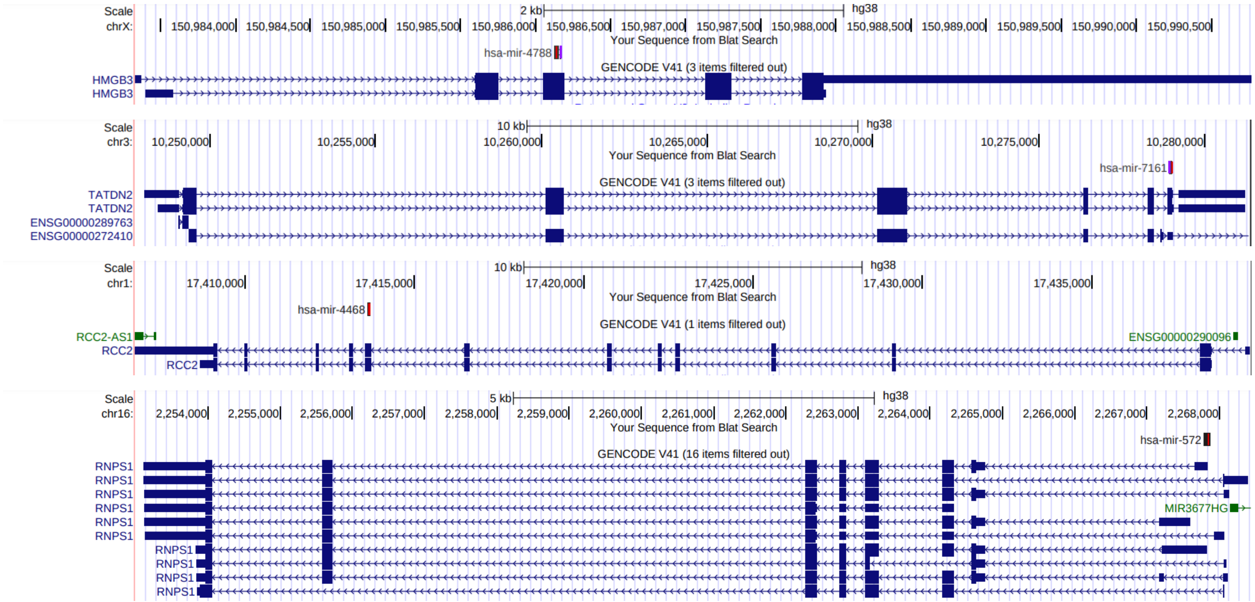

Figure S3. Parental genes without exonic miRNA. Visualization of the Genome Browser in the parental gene region of the retro-miR (hsa-miR-4788; hsa-miR-7161; hsa-miR-4468; hsa-miR-572). The BLAT sequence search and GENCODE V41 track activated. The coding transcripts are represented in blue, while the sequence aligned in the human genome are present in the gray.

Figure S4

RNPS1	chr16:2267786-2267880

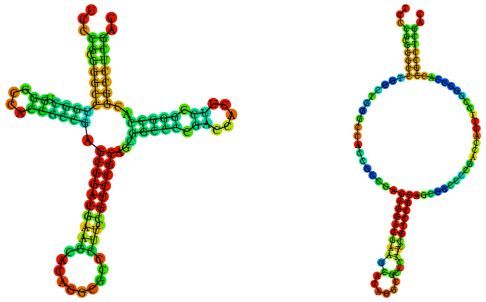

mir-572	chr4:11368827-11368921

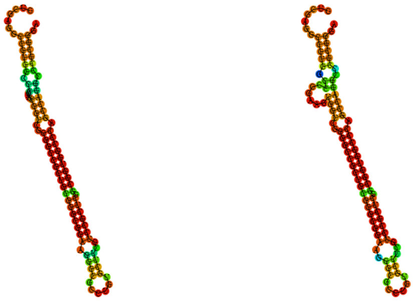

TATDN2	chr3:10278942-10279024

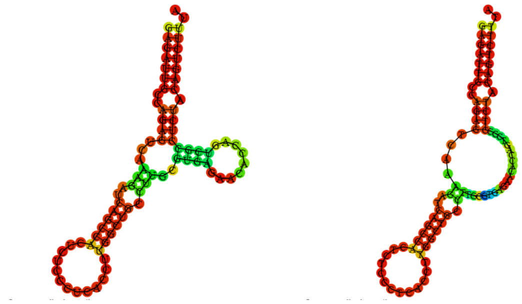

mir-7161	chr6:158609707-158609790

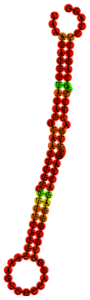

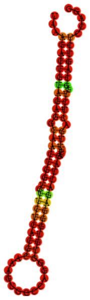

HMGB3	chrX:150986122-150986170

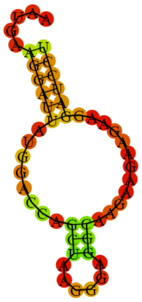

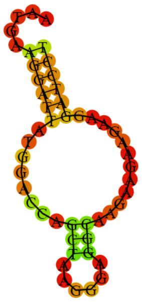

mir-4788	chr3:134437827-134437906

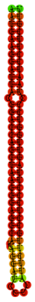

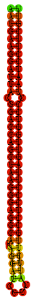

RCC2	chr1:17413631-17413694

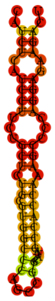

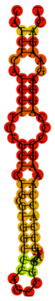

mir-4468	chr7:138123758-138123821

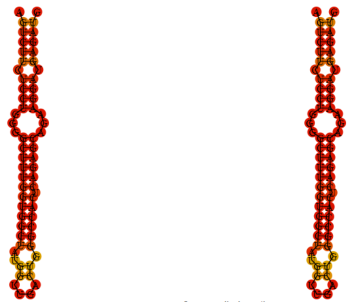

Figure S4. Regions with and without evidence of a stem loop. Presents a hairpin analysis of miRNA sequences and their corresponding parental structures. The displayed structures represent the potential stem loops for each sequence. The colors range from red to green, where red indicates a highly probable structure, and green indicates a less likely structure.

Figure S5

A

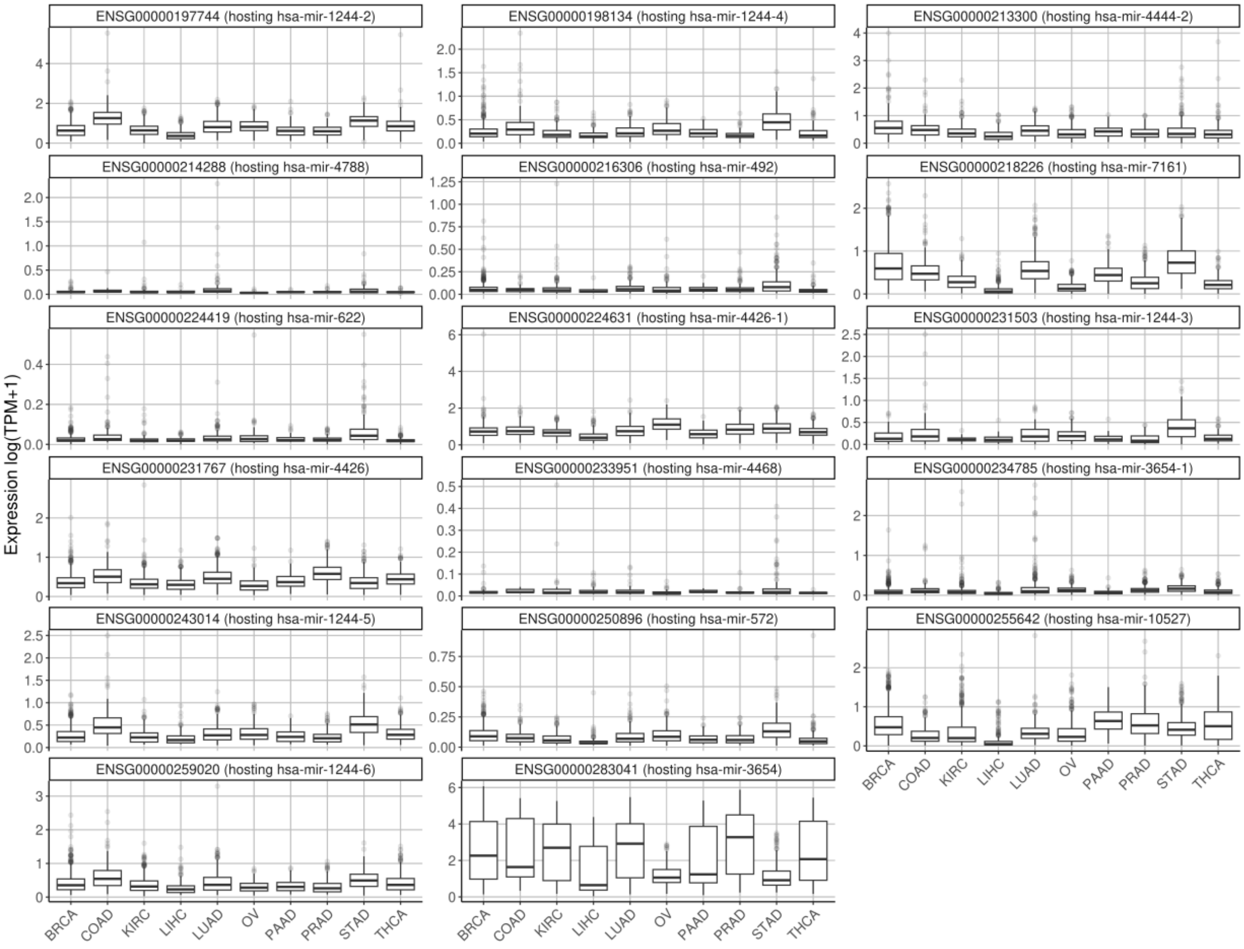
ENSG00000197744 (hosting hsa-mir-1244-2)
ENSG00000198134 (hosting hsa-mir-1244-4)
ENSG00000213300 (hosting hsa-mir-4444-2)
ENSG00000214288 (hosting hsa-mir-4788)
ENSG00000216306 (hosting hsa-mir-492)
ENSG00000218226 (hosting hsa-mir-7161)
ENSG00000224419 (hosting hsa-mir-622)
ENSG00000224631 (hosting hsa-mir-4426-1)
ENSG00000231503 (hosting hsa-mir-1244-3)
ENSG00000231767 (hosting hsa-mir-4426)
ENSG00000233951 (hosting hsa-mir-4468)
ENSG00000234785 (hosting hsa-mir-3654-1)
ENSG00000243014 (hosting hsa-mir-1244-5)
ENSG00000250896 (hosting hsa-mir-572)
ENSG00000255642 (hosting hsa-mir-10527)
ENSG00000259020 (hosting hsa-mir-1244-6)
ENSG00000283041 (hosting hsa-mir-3654)
Expression log(TPM+1)
BRCA
COAD
KIRC
LIHC
LUAD
OV
PAAD
PRAD
STAD
THCA

B

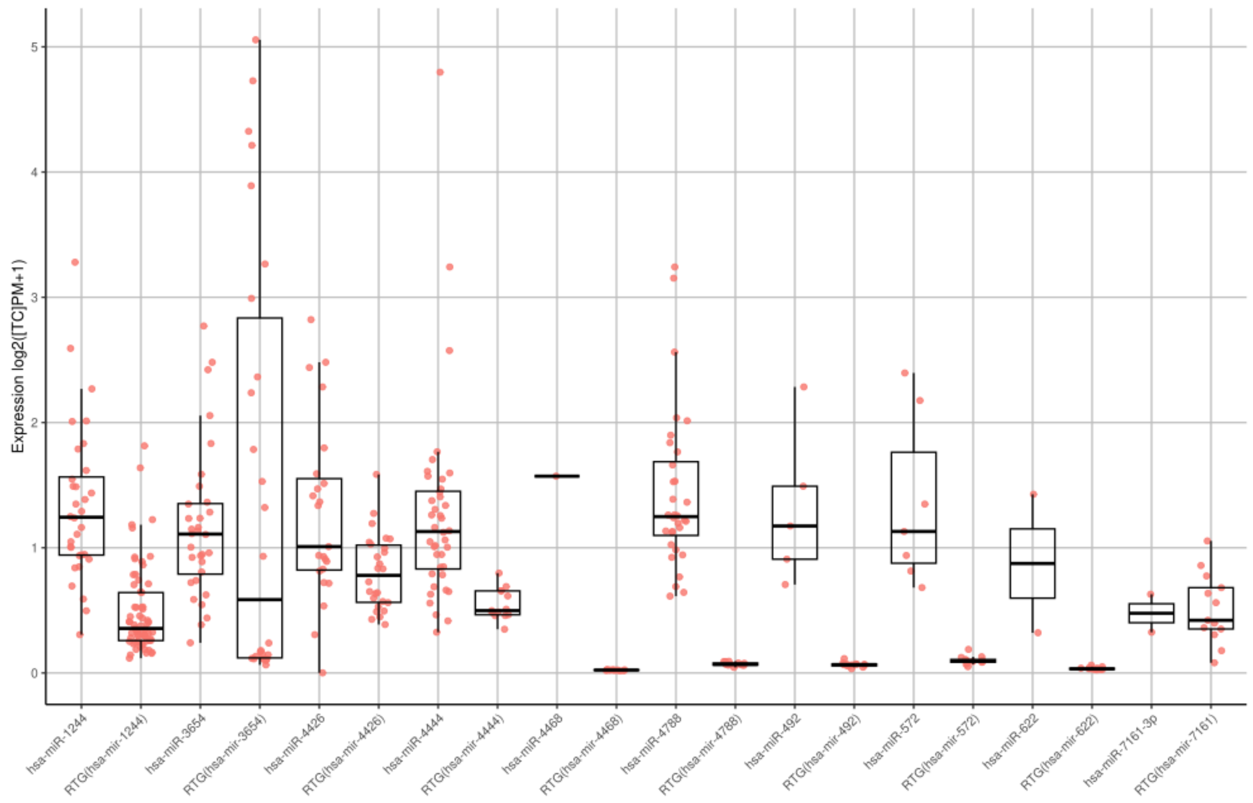

C

Retro-miRs	Genomic loci	prom (Promote)	enhP (Proximal enhancer)	enhD (Distal enhancer)	K4m3 (DNase-H 3K4me3)	CTCF (CTCF binding site)
hsa-mir-4426	chr1:192716328-192716390	0	0	15	0	2
hsa-mir-4426-1	chr16:61055707-61055769	0	0	0	0	3
hsa-mir-1244-2	chr5:118974586-118974670	3	4	6	0	1
hsa-mir-1244-3	chr12:9239467-9239551	1	1	4	0	0
hsa-mir-1244-4	chr12:12111952-12112036	0	0	6	2	1
hsa-mir-1244-5	chr3:117027474-117027558	0	0	10	0	0
hsa-mir-1244-6	chr14:92027342-92027426	2	6	8	0	0
hsa-mir-3654	chr7:133034860-133034915	0	2	14	1	0
hsa-mir-3654-1	chrX:115703812-115703867	0	0	9	1	0

Figure S5. Retro-miRs loci are transcribed and have nearby Cis Regulatory Elements. A) Expression of retrocopies (hosting retro-miRs) in TCGA. The expression levels of retrocopies in TCGA samples. The X-axis displays the tumor types used in the miRNA expression analysis (BRCA = Breast cancer, COAD = Colon adenocarcinoma, KIRC = Kidney renal clear cell carcinoma, LIHC = Liver hepatocellular carcinoma, LUSC = Lung squamous cell carcinoma, LUAD = Lung adenocarcinoma, PAAD = Pancreatic adenocarcinoma, PRAD = Prostate adenocarcinoma, STAD = Stomach adenocarcinoma, THCA = Thyroid carcinoma). The Y-axis represents the expression levels in log(TPM+1) where these were different from 0. The identifiers correspond to Ensembl IDs, providing a unique reference for each retrocopy.

B) The X-axis represents retro-miRs (miRNAs) and their host retrogenes (designated as RTG - Retro-miR's Name). We present a side-by-side comparison of retro-miRs and their host retrogenes' expression. The Y-axis displays expression levels (log2(+1)) in CPM for miRNAs and in log2(TPM+1) for retrogenes. Notably, the boxes representing retrogenes provide insight into the median expression across diverse TCGA tissues (BLCA, BRCA, COAD, KIRC, LGG, LIHC, LUAD, OV, PAAD, PRAD, SKCM, STAD, THCA). Concurrently, the boxes related to miRNAs depict the median expression across 32 distinct tissues from the FANTOM dataset. Naturally, expression to miRNAs or retrogenes that are not expressed (expression = 0) are not shown.

C) ENCODE Candidate Cis-Regulatory Elements (cCREs) nearby retrocopies hosting the retro-miRs in question: cCREs that lay within a 20,000kb window from the retrocopy where accounted. cCREs prom = cCREs with promoter-like signatures fall within 200 bp of an annotated GENCODE TSS and have high DNase and H3K4me3 signals. cCREs enhP = proximal enhancer-like signatures have high DNase and H3K27ac with low H3K4me3 max-Z score within 2 kb of a TSS. cCREs enhD = distant enhancer-like signatures have high DNase and H3K27ac with low H3K4me3 max-Z score further than 2 kb of a TSS. cCREs K4m3 = high H3K4me3 max-Z scores but low H3K27ac max-Z scores. CTCF = CTCF-only cCREs have high DNase and CTCF and low H3K4me3 and H3K27ac.

Figure S6

miR-10527

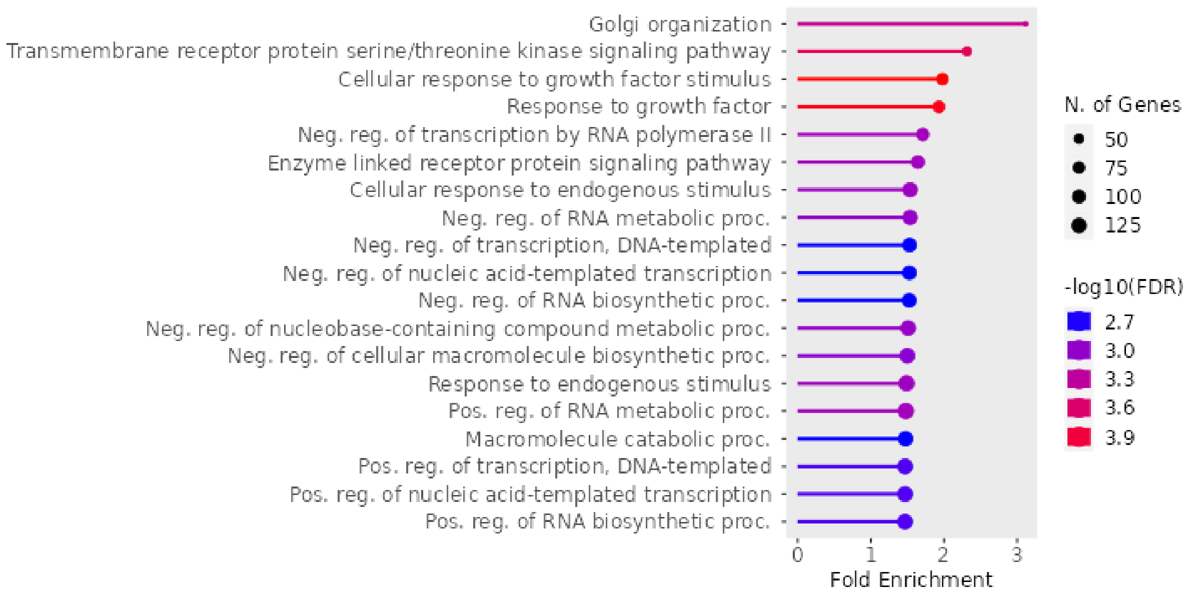

miR-1244

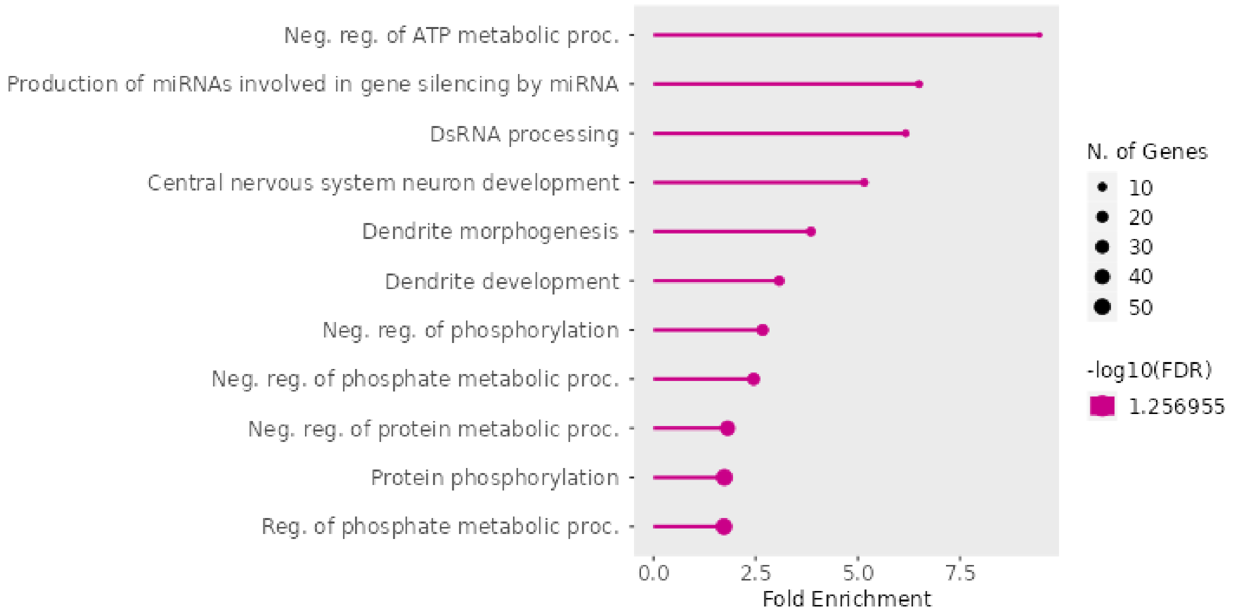

miR-3654

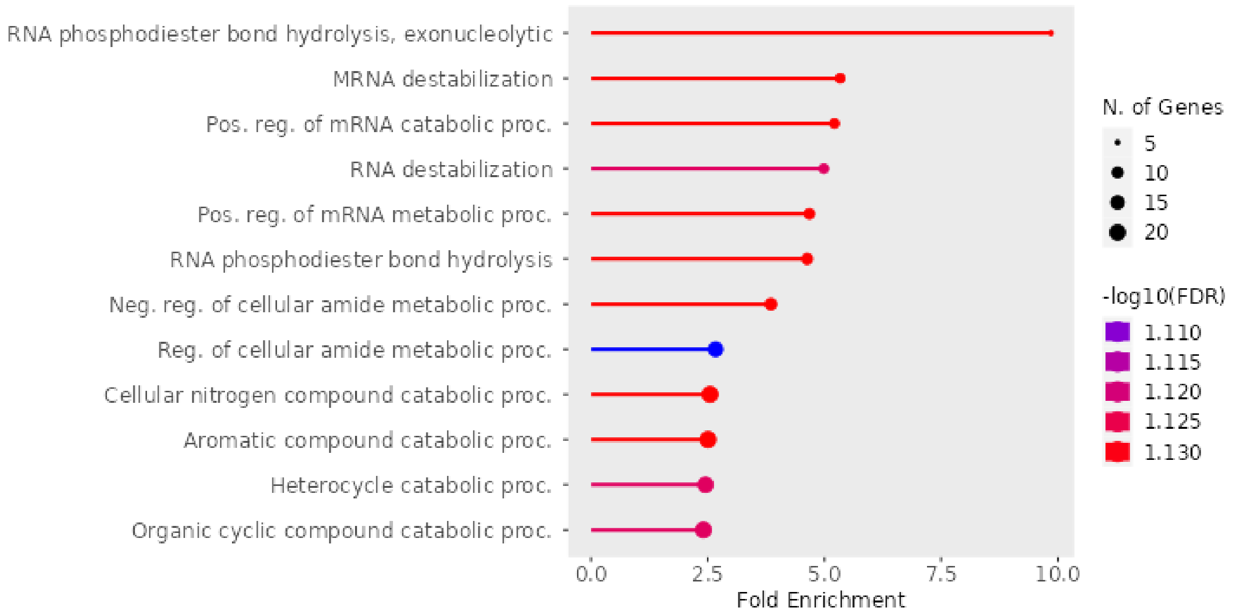

miR-4426
None

miR-4444

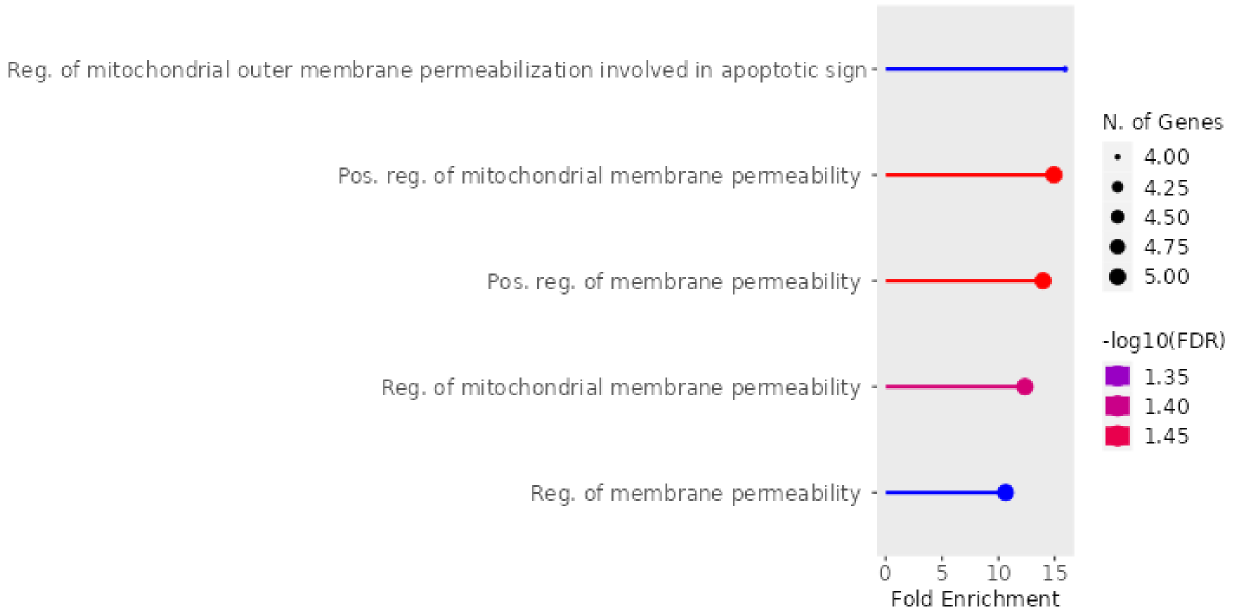

miR-4468

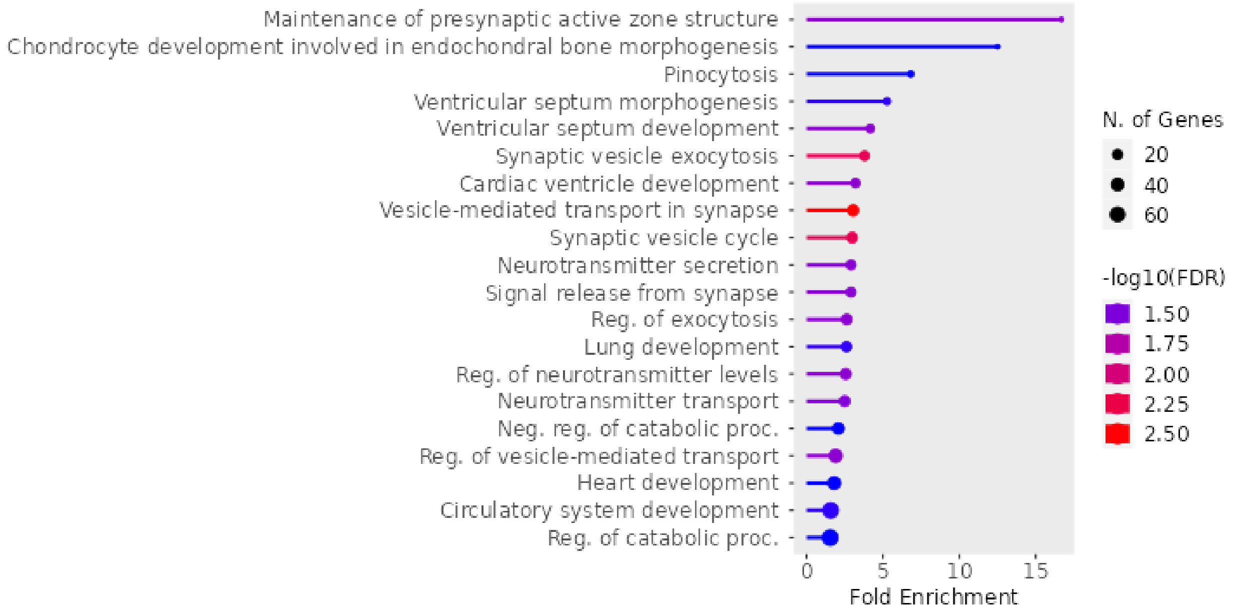

miR-4788

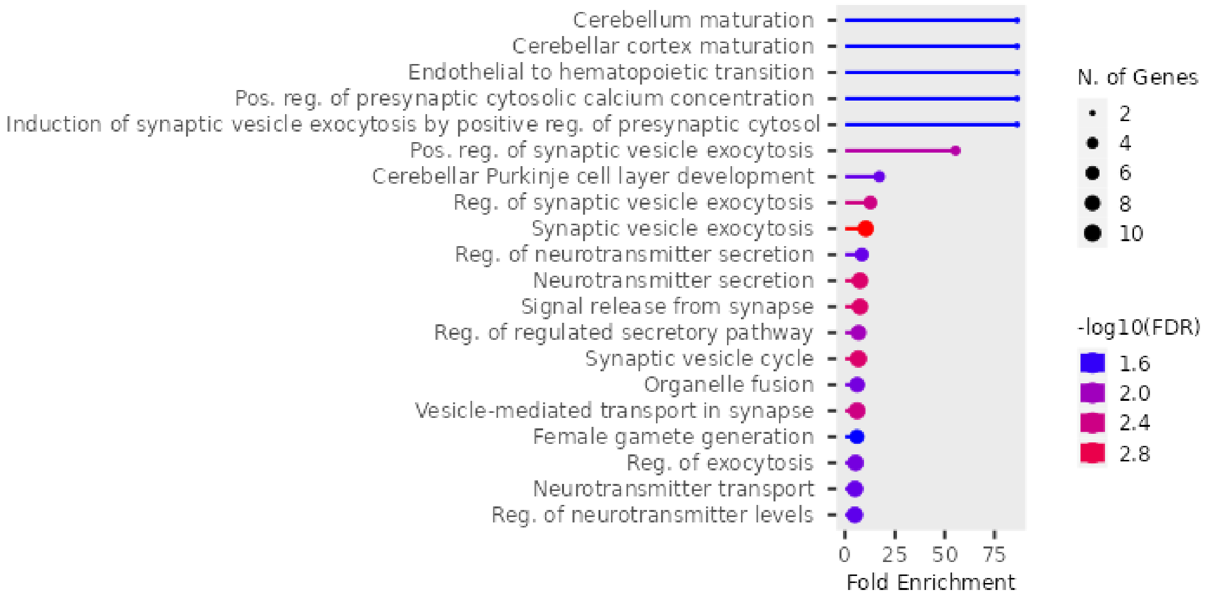

miR-492

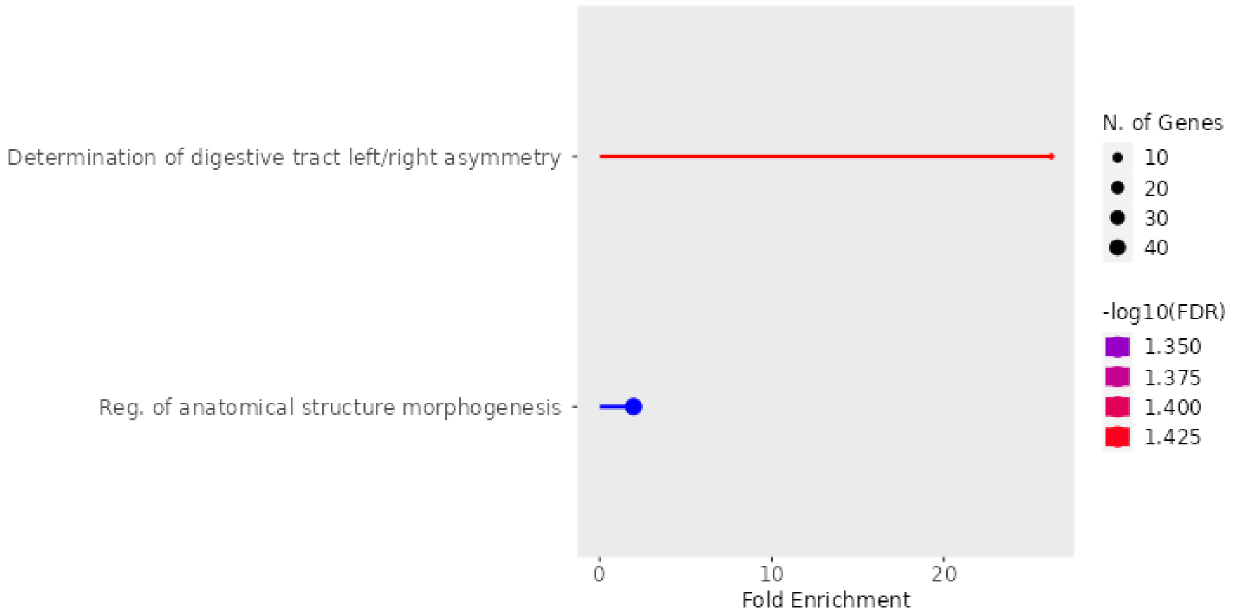

miR-7161-5p

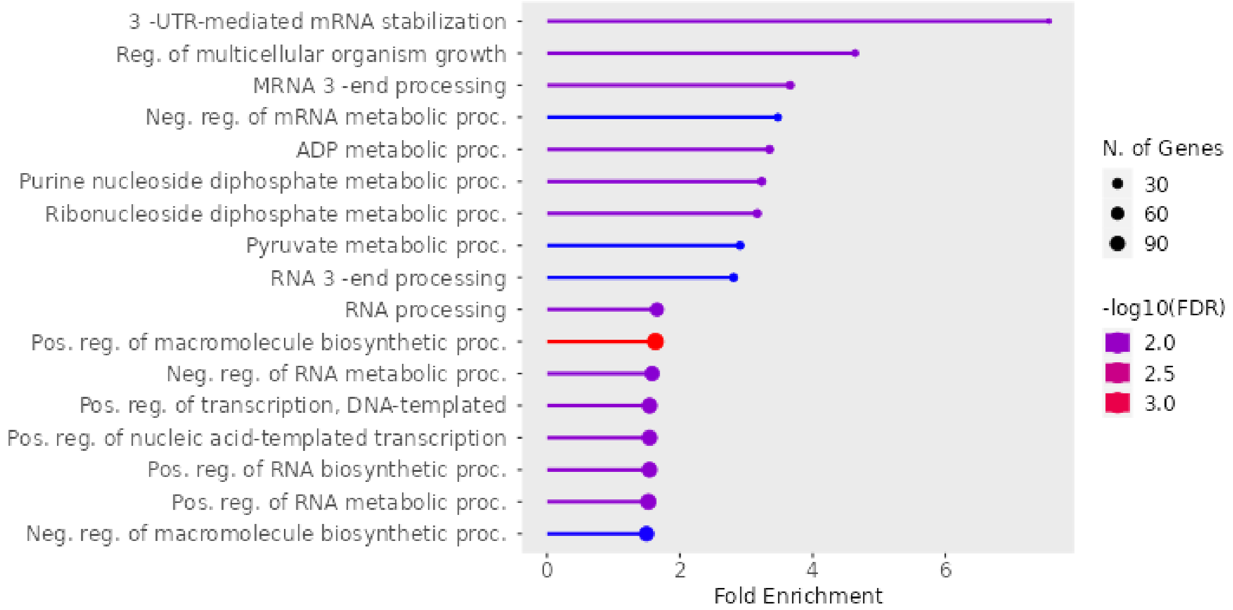

miR-7161-3p
None

miR-572

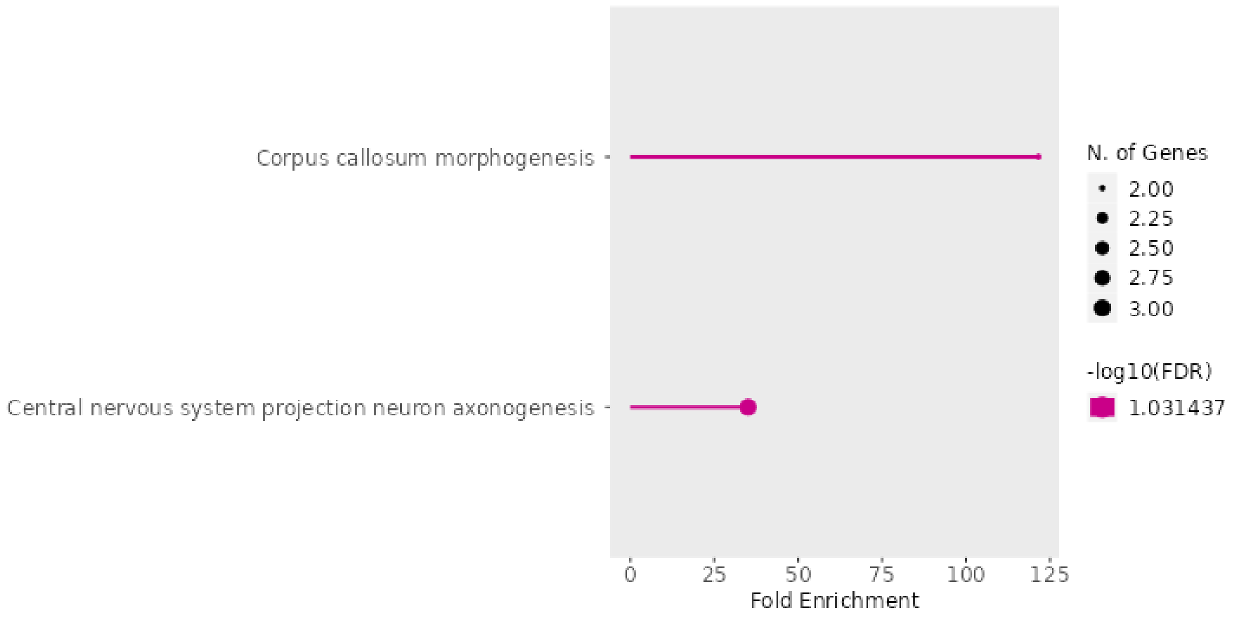

miR-622

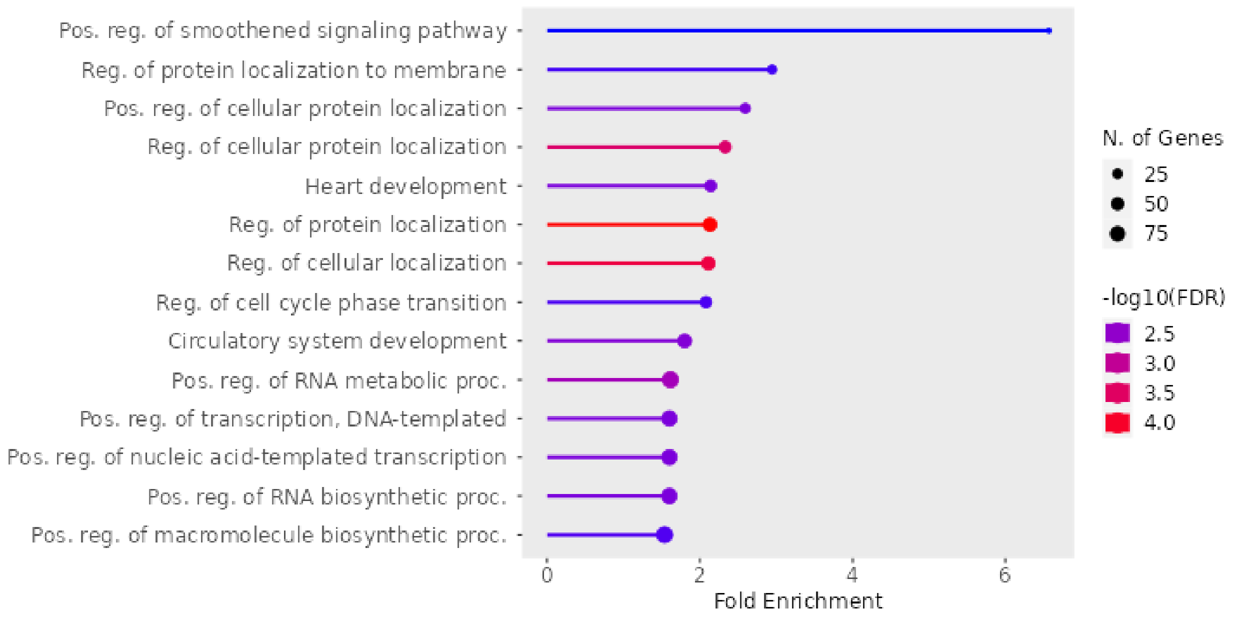

Figure S6. Gene ontology analyses of retro-miRs targets. A lollipop plot representing the results of the Gene Ontology analysis for each miRNA. The size of each point represents the number of genes associated with the respective pathway. The Y-axis displays the pathway

names, while the X-axis represents the fold enrichment values. The color of each point corresponds to the -log10(FDR), indicating the significance level of enrichment.
