# Supplementary material for: Retro-miRs: novel and functional miRNAs originating from mRNA retrotransposition
Source: Mob DNA. 2023 Sep 8;14:12. doi: 10.1186/s13100-023-00301-w (PMC10486083; doi:10.1186/s13100-023-00301-w)
Supplement: Supplementary file 2 — Additional file 2: Table S1. Retro-miRs information. [file 13100_2023_301_MOESM2_ESM.pdf]

Table S1. Retro-miRs information

miRNA (Official name)	micro-RNA	mature miRNA	Classes	Position	mat. miRNA sequence	Position	Retrocopy	Position	Parental Gene	Position
MIR4444-2	mir-4444-2	hsa-miR-4444	RTC	chr3:75214476-75214549	CUCGAGUUGGAAGAGGCG	chr3:75214518-75214535	HNRNPA3P6	chr3:75214631-75215636	HNRNPA3	chr2:177212563-177223958
MIR4426	mir-4426	hsa-miR-4426	EJ	chr1:192716328-192716390	GAAGAUGGACGUACUUU	chr1:192716333-192716349	RPS27AP5	chr1:192716132-192716653	RPS27A	chr2:55231903-55235853
-	mir-4426-1	hsa-miR-4426	EJ	chr16:61055707-61055769	GAAGAUGGACGUACUUU	chr16:61055744-61055760	RPS27AP16	chr16:61055399-61055964	RPS27A	chr2:55231903-55235853
MIR1244-2	mir-1244-2	hsa-miR-1244	RTC	chr5:118974586-118974670	AAGUAGUUGGUUUGUAUGAGAUGGUU	chr5:118974640-118974665	PTMAP2	chr5:118973796-118974122	PTMA	chr2:231706895-231713551
MIR1244-3	mir-1244-3	hsa-miR-1244	RTC	chr12:9239467-9239551	AAGUAGUUGGUUUGUAUGAGAUGGUU	chr12:9239472-9239497	PTMAP4	chr12:9239986-9240331	PTMA	chr2:231706895-231713551
MIR1244-4	mir-1244-4	hsa-miR-1244	RTC	chr12:12111952-12112036	AAGUAGUUGGUUUGUAUGAGAUGGUU	chr12:12112006-12112031	PTMAP9	chr12:12111163-12111489	PTMA	chr2:231706895-231713551
-	mir-1244-5	hsa-miR-1244	RTC	chr3:117027474-117027558	AAGUAGUUGGUUUGUAUGAGAUGGUU	chr3:117027528-117027553	PTMAP8	chr3:117026698-117027039	PTMA	chr2:231706895-231713551
-	mir-1244-6	hsa-miR-1244	RTC	chr14:92027342-92027426	AAGUAGUUGGUUUGUAUGAGAUGGUU	chr14:92027396-92027421	RP11-529H20.3	chr14:92026422-92027567	PTMA	chr2:231706895-231713551
MIR3654	mir-3654	hsa-miR-3654	EJ	chr7:133034860-133034915	GACUGGACAAGCUGAGGAA	chr7:133034860-133034878	RP11-371A22.1	chr7:133034607-133035920	EEF1G	chr11:62559596-62574086
-	mir-3654-1	hsa-miR-3654	EJ	chrX:115703812-115703867	GACUGGACAAGCUGAGGAA	chrX:115703849-115703867	EEF1GP5	chrX:115702811-115704120	EEF1G	chr11:62559596-62574086
MIR572	mir-572	hsa-miR-572	Novel	chr4:11368827-11368921	GUCCGCUCGGCGGUGGCCCA	chr4:11368887-11368906	RNPS1P1	chr4:11368821-11373738	RNPS1	chr16:2253116-2268397
MIR622	mir-622	hsa-miR-622	EJ	chr13:90231182-90231277	ACAGUCUGCUGAGGUUGGAGC	chr13:90231242-90231262	KRT18P27	chr13:90230384-90231682	KRT18	chr12:52948871-52952906
MIR7161	mir-7161	hsa-miR-7161-3p	Novel	chr6:158609707-158609790	UAGAUCUUUGACUCUGGCAGUCUCCAGG	chr6:158609763-158609790	TATDN2P2	chr6:158609706-158621636	TATDN2	chr3:10248023-10281218
MIR7161	mir-7161	hsa-miR-7161-5p	Novel	chr6:158609707-158609790	UAAAGACUGUAGAGGCAACUGGU	chr6:158609707-158609729	TATDN2P2	chr6:158609706-158621636	TATDN2	chr3:10248023-10281218
MIR4788	mir-4788	hsa-miR-4788	Novel	chr3:134437827-134437906	UUACGGACCAGCUAAGGGAGGC	chr3:134437836-134437857	HMGB3P13	chr3:134437605-134438211	HMGB3	chrX:150980509-150990771
MIR4468	mir-4468	hsa-miR-4468	Novel	chr7:138123758-138123821	AGAGCAGAAGGAUGAGAU	chr7:138123803-138123820	RCC2P3	chr7:138122202-138124595	RCC2	chr1:17406760-17439677
MIR492	mir-492	hsa-miR-492	EJ	chr12:94834398-94834513	AGGACCUGCGGGACAAGAUUCUU	chr12:94834427-94834449	KRT19P2	chr12:94834147-94835158	KRT19	chr17:41523617-41528308
MIR10527	mir-10527	hsa-miR-10527-5p	EJ	chr12:63823663-63823727	AAAGCAAAUGUUGGGUGAACGGC	chr12:63823663-63823685	PABPC1P4	chr12:63822021-63823895	PABPC1	chr8:100685816-100722809

*** The microRNA nomenclature can be divided into three main components:
i) The species, which is named using three letters. For example, "hsa" is used to represent microRNAs found in the human genome.
ii) The microRNA, which indicates whether it refers to the pre-miRNAs (prefixed with "mir," e.g., mir-576) or the mature miRNAs (prefixed with "miR," e.g., miR-576). Precursors are named with a lowercase "r" before "mir," while mature miRNAs are represented with an uppercase "R" in "mir.".
iii) Additional information, which applies to mature miRNAs, may include a type designation at the end of the name. If only one mature miRNA is reported, no additional information is provided (e.g., "hsa-miR-492"). However, if there are two annotations, they are represented as 3p and 5p, indicating the strand of the miRNA. For example, "hsa-miR-7161-3p" and "hsa-miR-7161-5p." In the case of pre-miRNAs, this additional information may indicate the existence of another miRNA gene in a different genomic position but with a similar or identical stem-loop structure. These patterns are numbered using integers, such as 1, 2, 3 (e.g., "hsa-mir-4444-1" and "hsa-mir-4444-2").
