# Supplementary material for: Retro-miRs: novel and functional miRNAs originating from mRNA retrotransposition
Source: Mob DNA. 2023 Sep 8;14:12. doi: 10.1186/s13100-023-00301-w (PMC10486083; doi:10.1186/s13100-023-00301-w)
Supplement: Supplementary file 3 — Additional file 3: Table S2. Retrocopied genes with exonic miRNAs. [file 13100_2023_301_MOESM3_ESM.pdf]

Table S2. Retrocopied genes with exonic miRNAs.

Parental Gene	Exonic miRNAs	Retrocopie	RTC Position	With miRNA			
KRT18	N	KRT18P27	chr13:90230384-90231682	Y			
TATDN2	Y	TATDN2P2	chr6:158609706-158621636	Y			
RNPS1	N	RNPS1P1	chr4:11368821-11373738	Y			
KRT19	N	KRT19P2	chr12:94834147-94835158	Y			
KRT19	N	KRT19P1	chr6:71584721-71585906	N			
KRT19	N	KRT19P3	chr4:109879070-109879897	N			
KRT19	N	KRT19P4	chr10:68260557-68261499	N			
KRT19	N	KRT19P6	chr4:91885046-91885254	N			
RCC2	N	RCC2P3	chr7:138122202-138124595	Y			
RCC2	N	RCC2P1	chrY:11781052-11782527	N			
RCC2	N	RCC2P2	chrY:19986575-19987852	N			
RCC2	N	RCC2P4	chr3:126766434-126767475	N			
RCC2	N	RCC2P5	chr3:96753185-96754671	N			
RCC2	N	RCC2P6	chr11:62371146-62373168	N			
RCC2	N	RCC2P7	chr6:56431950-56433213	N			
RCC2	N	RCC2P8	chr4:108788745-108789779	N			
EEF1G	N	EEF1GP9	chr7:133034607-133035920	Y			
EEF1G	N	EEF1GP5	chrX:115702811-115704120	Y			
EEF1G	N	EEF1GP1	chr7:125033453-125035301	N			
EEF1G	N	EEF1GP2	chr5:147922179-147923421	N			
EEF1G	N	EEF1GP3	chr3:40596207-40597494	N			
EEF1G	N	EEF1GP4	chr3:161324913-161326219	N			
EEF1G	N	EEF1GP8	chr4:129903010-129904163	N			
EEF1G	N	EEF1GP7	chr1:52573114-52573816	N			
PABPAC1	Y	PABPC1P4	chr12:63822021-63823895	Y			
PABPAC1	Y	PABPC1P1	chr4:39973444-39974338	N			
PABPAC1	Y	PABPC1P2	chr2:146587506-146589310	N			
PABPAC1	Y	PABPC1P3	chrX:74583088-74583546	N			
PABPAC1	Y	PABPC1P7	chr4:102896725-102898237	N			
PABPAC1	Y	PABPC1P10	chr3:155309427-155309807	N			

PABPAC1	Y	PABPC1P11	chr9:17589161-17591318	N			
PABPAC1	Y	PABPC1P15	chr4:39973128-39973442	N			
PABPAC1	Y	PABPC3	chr13:25096141-25099255	N			
PTMA	Y	PTMAP2	chr5:118973796-118974122	Y			
PTMA	Y	PTMAP4	chr12:9239986-9240331	Y			
PTMA	Y	PTMAP9	chr12:12111163-12111489	Y			
PTMA	Y	PTMAP8	chr3:117026698-117027039	Y			
PTMA	Y	PTMAP16	chr14:92026422-92027567	Y			
PTMA	Y	PTMAP1	chr6:30633632-30633956	N			
PTMA	Y	PTMAP3	chr20:18011955-18012284	N			
PTMA	Y	PTMAP5	chr13:81689911-81691072	N			
PTMA	Y	PTMAP10	chr7:138404195-138404523	N			
PTMA	Y	PTMAP13	chr17:76651409-76652084	N			
PTMA	Y	PTMAP14	chrX:15653107-15653855	N			
PTMA	Y	PTMAP15	chr8:103559099-103559389	N			
HNRNPA3	Y	HNRNPA3P6	chr3:75214631-75215636	Y			
HNRNPA3	Y	HNRNPA3P14	chr1:81426421-81428335	N			
HNRNPA3	Y	HNRNPA3P17	chr1:113449660-113451119	N			
HNRNPA3	Y	HNRNPA3P1	chr10:43787125-43790442	N			
HNRNPA3	Y	HNRNPA3P9	chr11:32591403-32592766	N			
HNRNPA3	Y	HNRNPA3P10	chr12:51711727-51713612	N			
HNRNPA3	Y	HNRNPA3P5	chr13:65787546-65788763	N			
HNRNPA3	Y	HNRNPA3P11	chr15:57246585-57248086	N			
HNRNPA3	Y	HNRNPA3P16	chr18:50814324-50816778	N			
HNRNPA3	Y	HNRNPA3P20	chr19:54031410-54031766	N			
HNRNPA3	Y	HNRNPA3P18	chr2:216174173-216176040	N			
HNRNPA3	Y	HNRNPA3P15	chr2:197014990-197016725	N			
HNRNPA3	Y	HNRNPA3P2	chr20:36620402-36621442	N			
HNRNPA3	Y	ENSG00000248977	chr4:40142207-40142402	N			
HNRNPA3	Y	HNRNPA3P13	chr4:82128528-82131631	N			
HNRNPA3	Y	HNRNPA3P19	chr6:25271821-25273808	N			

HNRNPA3	Y	HNRNPA3P3	chrX:140032318-140033870	N			
RPS27A	N	RPS27AP5	chr1:192716132-192716653	Y			
RPS27A	N	RPS27AP16	chr16:61055399-61055964	Y			
RPS27A	N	RPS27AP6	chr1:150881206-150881721	N			
RPS27A	N	RPS27AP24	chr14:27258697-27259175	N			
RPS27A	N	ENSG00000240813	chr17:10745481-10745815	N			
RPS27A	N	RPS27AP1	chr17:10258721-10259262	N			
RPS27A	N	RPS27AP19	chr19:7665678-7666501	N			
RPS27A	N	RPS27AP7	chr2:47883418-47883933	N			
RPS27A	N	RPS27AP22	chr2:224294926-224295787	N			
RPS27A	N	RPS27AP2	chr20:17516801-17517383	N			
RPS27A	N	RPS27AP3	chr20:37049238-37049745	N			
RPS27A	N	RPS27AP8	chr3:172225126-172225827	N			
RPS27A	N	RPS27AP18	chr5:138267027-138267796	N			
RPS27A	N	RPS27AP9	chr5:80498495-80498998	N			
RPS27A	N	RPS27AP10	chr5:141588893-141589428	N			
RPS27A	N	RPS27AP11	chr6:113581463-113581999	N			
RPS27A	N	RPS27AP12	chr7:157192326-157192862	N			
RPS27A	N	RPS27AP23	chr7:112446130-112446376	N			
RPS27A	N	RPS27AP13	chr8:53886980-53887581	N			
RPS27A	N	RPS27AP15	chr9:72741136-72741625	N			
RPS27A	N	RPS27AP17	chrX:6989352-6989815	N			
RPS27A	N	RPS27AP20	chrX:3717122-3717632	N			
HMGB3	N	HMGB3P13	chr3:134437605-134438211	Y			
HMGB3	N	HMGB3P9	chr1:92646202-92647649	N			
HMGB3	N	HMGB3P6	chr1:164356674-164358220	N			
HMGB3	N	HMGB3P10	chr1:99698241-99699197	N			
HMGB3	N	HMGB3P8	chr10:116439209-116440067	N			
HMGB3	N	HMGB3P5	chr10:110450605-110452121	N			
HMGB3	N	HMGB3P34	chr11:87477706-87481337	N			
HMGB3	N	HMGB3P7	chr13:101801793-101803581	N			

HMGB3	N	HMGB3P26	chr14:103364234-103364844	N				
HMGB3	N	HMGB3P32	chr16:57603098-57605532	N				
HMGB3	N	HMGB3P27	chr17:42648154-42648738	N				
HMGB3	N	HMGB3P35	chr18:12450417-12451057	N				
HMGB3	N	HMGB3P36	chr19:17417016-17418324	N				
HMGB3	N	HMGB3P11	chr2:104771191-104772745	N				
HMGB3	N	HMGB3P2	chr20:36076000-36076609	N				
HMGB3	N	HMGB3P1	chr20:34833564-34834486	N				
HMGB3	N	HMGB3P12	chr3:26216230-26218085	N				
HMGB3	N	HMGB3P14	chr3:134170401-134171142	N				
HMGB3	N	HMGB3P15	chr4:94195919-94196544	N				
HMGB3	N	HMGB3P16	chr5:112451842-112453021	N				
HMGB3	N	HMGB3P22	chr5:179694178-179694786	N				
HMGB3	N	HMGB3P17	chr5:123467942-123469475	N				
HMGB3	N	HMGB3P19	chr6:153938024-153939121	N				
HMGB3	N	HMGB3P18	chr6:121856769-121858746	N				
HMGB3	N	HMGB3P20	chr7:26981269-26983459	N				
HMGB3	N	HMGB3P23	chr9:31643345-31645184	N				
HMGB3	N	HMGB3P24	chr9:36302688-36305016	N				
HMGB3	N	HMGB3P31	chrX:134959064-134962399	N				
HMGB3	N	HMGB3P30	chrX:112689892-112691366	N				
KRT18	N	KRT18P28	chr1:182959007-182960364	N				
KRT18	N	KRT18P12	chr1:214532193-214533869	N				
KRT18	N	KRT18P32	chr1:238491356-238492715	N				
KRT18	N	KRT18P14	chr11:35860189-35861550	N				
KRT18	N	KRT18P59	chr11:125113568-125114938	N				
KRT18	N	KRT18P58	chr11:5993808-5995153	N				
KRT18	N	KRT18P20	chr12:104976684-104978028	N				
KRT18	N	KRT18P60	chr12:65418652-65420025	N				
KRT18	N	KRT18P6	chr14:35511731-35513091	N				
KRT18	N	KRT18P7	chr14:70594550-70595902	N				

KRT18	N	KRT18P47	chr15:88959761-88961138	N			
KRT18	N	KRT18P72	chr17:67276067-67276349	N			
KRT18	N	KRT18P61	chr17:60810163-60811539	N			
KRT18	N	KRT18P55	chr17:28275967-28277338	N			
KRT18	N	KRT18P8	chr18:9678170-9679496	N			
KRT18	N	KRT18P26	chr2:74306653-74308010	N			
KRT18	N	KRT18P52	chr2:31822513-31823417	N			
KRT18	N	KRT18P33	chr2:65666469-65667813	N			
KRT18	N	KRT18P39	chr2:203764218-203765589	N			
KRT18	N	KRT18P29	chr2:181961149-181962480	N			
KRT18	N	KRT18P19	chr2:189311193-189312545	N			
KRT18	N	KRT18P4	chr20:49956740-49958106	N			
KRT18	N	KRT18P3	chr20:22732733-22734091	N			
KRT18	N	KRT18P2	chr21:20424882-20426169	N			
KRT18	N	KRT18P23	chr22:44566964-44568321	N			
KRT18	N	KRT18P5	chr22:20482391-20484107	N			
KRT18	N	KRT18P62	chr22:19257518-19258872	N			
KRT18	N	KRT18P35	chr3:141470548-141471902	N			
KRT18	N	KRT18P43	chr3:169902913-169904258	N			
KRT18	N	KRT18P15	chr3:32258941-32260302	N			
KRT18	N	KRT18P34	chr3:157162596-157163934	N			
KRT18	N	KRT18P17	chr3:12787335-12788670	N			
KRT18	N	KRT18P54	chr4:135371108-135371874	N			
KRT18	N	KRT18P51	chr4:144572238-144572986	N			
KRT18	N	KRT18P25	chr4:40020240-40021567	N			
KRT18	N	KRT18P21	chr4:115920705-115922351	N			
KRT18	N	KRT18P63	chr4:17911672-17913044	N			
KRT18	N	KRT18P42	chr5:109588337-109589660	N			
KRT18	N	KRT18P16	chr5:123636108-123637495	N			
KRT18	N	KRT18P45	chr5:80288448-80289854	N			
KRT18	N	KRT18P31	chr5:36885204-36886552	N			

KRT18	N	KRT18P9	chr6:34189776-34191158	N			
KRT18	N	KRT18P50	chr6:95991115-95992398	N			
KRT18	N	KRT18P22	chr6:116457247-116458605	N			
KRT18	N	KRT18P37	chr8:41511239-41512590	N			
KRT18	N	KRT18P67	chr9:122837552-122838891	N			
KRT18	N	KRT18P36	chr9:30799422-30800750	N			
KRT18	N	KRT18P66	chr9:30773463-30774812	N			
KRT18	N	KRT18P44	chrX:128714504-128716141	N			
KRT18	N	KRT18P49	chrX:107428627-107429531	N			
KRT18	N	KRT18P57	chr1:111648206-111649597	N			
KRT18	N	KRT18P18	chr16:72728565-72729982	N			
KRT18	N	KRT18P40	chr19:20961058-20965274	N			
KRT18	N	KRT18P46	chr2:161580035-161581419	N			
KRT18	N	KRT18P41	chr5:170140596-170141987	N			
KRT18	N	KRT18P56	chr5:40066609-40067201	N			
KRT18	N	KRT18P38	chr6:19612695-19614090	N			
KRT18	N	KRT18P64	chr6:85287676-85289068	N			
KRT18	N	KRT18P1	chr6:28969076-28970467	N			
KRT18	N	KRT18P65	chr6:112361373-112362775	N			
KRT18	N	KRT18P24	chr9:79036337-79037748	N			
KRT18	N	KRT18P13	chr9:97698872-97700801	N			
KRT18	N	KRT18P68	chrX:45983322-45984369	N			
KRT18	N	KRT18P48	chrX:153604514-153605390	N			
