# Supplementary material for: Retro-miRs: novel and functional miRNAs originating from mRNA retrotransposition
Source: Mob DNA. 2023 Sep 8;14:12. doi: 10.1186/s13100-023-00301-w (PMC10486083; doi:10.1186/s13100-023-00301-w)
Supplement: Supplementary file 4 — Additional file 4: Table S3. Duplications of protein coding genes containing exonic miRNAs. [file 13100_2023_301_MOESM4_ESM.pdf]

Table S3. Duplications of protein coding genes containing exonic miRNAs.					
DNA mediated duplications					
**Gene1**	**Genomic position**	**With miRNA**	**Gene2**	**Genomic Position**	**With miRNA**
CCNYL1	chr2:207711540-207761839	Y	CCNY	chr10:35247025-35572669	N
EHD1	chr11:64851642-64888296	Y	EHD3	chr2:31234152-31269451	N
EHD1	chr11:64851642-64888296	Y	EHD4	chr15:41895933-41972557	N
EHD1	chr11:64851642-64888296	Y	EHD2	chr19:47713422-47743134	N
EML2	chr19:45606994-45645629	Y	EML1	chr14:99737693-99942060	N
F8A3	chrX:155456914-155458672	Y	F8A2	chrX:155382115-155383230	Y
F8A3	chrX:155456914-155458672	Y	F8A1	chrX:154886349-154888061	Y
FADS1	chr11:61799627-61829318	Y	FADS2	chr11:61792980-61867354	N
FADS1	chr11:61799627-61829318	Y	FADS3	chr11:61873519-61892051	N
FKBP1A	chr20:1368978-1393172	Y	FKBP1B	chr2:24049701-24063681	N
GNAI3	chr1:109548615-109618324	Y	GNAI1	chr7:79768028-80226181	N
GNAI3	chr1:109548615-109618324	Y	GNAI2	chr3:50226292-50259362	N
GNAI3	chr1:109548615-109618324	Y	GNAO1	chr16:56191489-56357444	N
GNAI3	chr1:109548615-109618324	Y	GNAZ	chr22:23070519-23125032	N
GNAI3	chr1:109548615-109618324	Y	GNAT3	chr7:80458635-80512064	N
GNAI3	chr1:109548615-109618324	Y	GNAT2	chr1:109603254-109619929	N
GNAI3	chr1:109548615-109618324	Y	GNAT1	chr3:50191610-50197696	N
GNAI3	chr1:109548615-109618324	Y	GNA14	chr9:77423079-77648322	N
GNAI3	chr1:109548615-109618324	Y	GNA13	chr17:65009289-65056740	N
GNAI3	chr1:109548615-109618324	Y	GNA11	chr19:3094362-3123999	N
HNRNPA3	chr2:177212563-177223958	Y	HNRNPA2B17	chr7:26173057-26201529	N
NR2F2	chr15:96325938-96340263	Y	NR2F1	chr5:93583222-93594611	N
PABPC1	chr8:100685816-100722809	Y	PABPC4	chr1:39560816-39576790	N
PABPC1	chr8:100685816-100722809	Y	PABPC1L	chr20:44910062-44959035	N
PILRB	chr7:100352176-100367733	Y	PILRA	chr7:100367530-100400096	N
PLEKHM1	chr17:45435900-45490749	Y	PLEKHM1P1	chr17:64779259-64837154	Y
PURB	chr7:44876299-44885530	Y	PURA	chr5:140107777-140125619	N
RBMS1	chr2:160272151-160493807	Y	RBMS3	chr3:28574791-30010391	N
RBMS1	chr2:160272151-160493807	Y	RBMS2	chr12:56521820-56596193	N
SERF2	chr15:43777087-43802589	Y	SERF1A	chr5:70900665-70918530	N
TAOK1	chr17:29390464-29551904	Y	TAOK2	chr16:29973868-29992261	N
TAOK1	chr17:29390464-29551904	Y	TAOK3	chr12:118149801-118372907	N
USP15	chr12:62260338-62417431	Y	USP11	chrX:47232690-47248328	N
USP15	chr12:62260338-62417431	Y	USP4	chr3:49277144-49340712	N
USP15	chr12:62260338-62417431	Y	USP32	chr17:60177327-60422470	N
USP15	chr12:62260338-62417431	Y	USP6	chr17:5116438-5175034	N
mRNA retroduplications					

Parental	Genomic position	With miRNA	RTC-Genomic Position	With miRNA
ANKRD54	chr22:37830855-37844334	Y	chr10:45646374-45647605	N
BUD31	chr7:99408641-99419616	Y	chrX:74421305-74421818	N
CCNYL1	chr2:207711640-207756174	Y	chr19:21933414-21937189	N
CCNYL1	chr2:207711640-207756174	Y	chrX:65037743-65042051	N
CDC37	chr19:10391133-10403542	Y	chr16:28425100-28427082	N
CDC37	chr19:10391133-10403542	Y	chr16:28711207-28712857	N
CDC37	chr19:10391133-10403542	Y	chr5:157413153-157414724	N
EED	chr11:86244753-86278810	Y	chr4:83492292-83492965	N
EED	chr11:86244753-86278810	Y	chrX:139479271-139480704	N
FKBP1A	chr20:1369000-1393123	Y	chr13:47079253-47082644	N
FKBP1A	chr20:1369000-1393123	Y	chr15:73434434-73435931	N
FKBP1A	chr20:1369000-1393123	Y	chr19:58336594-58338233	N
FKBP1A	chr20:1369000-1393123	Y	chr3:149180387-149181989	N
FKBP1A	chr20:1369000-1393123	Y	chr6:63921356-63922932	N
HAUS4	chr14:22946228-22957154	Y	chr1:170338060-170339530	N
HNRNPA3	chr2:177212724-177223920	Y	chr3:75214631-75215636	Y
HNRNPA3	chr2:177212724-177223920	Y	chr1:81426421-81428335	N
HNRNPA3	chr2:177212724-177223920	Y	chr1:113449660-113451119	N
HNRNPA3	chr2:177212724-177223920	Y	chr10:43787125-43790442	N
HNRNPA3	chr2:177212724-177223920	Y	chr11:32591403-32592766	N
HNRNPA3	chr2:177212724-177223920	Y	chr12:51711727-51713612	N
HNRNPA3	chr2:177212724-177223920	Y	chr13:65787546-65788763	N
HNRNPA3	chr2:177212724-177223920	Y	chr15:57246585-57248086	N
HNRNPA3	chr2:177212724-177223920	Y	chr18:50814324-50816778	N
HNRNPA3	chr2:177212724-177223920	Y	chr19:54031410-54031766	N
HNRNPA3	chr2:177212724-177223920	Y	chr2:216174173-216176040	N
HNRNPA3	chr2:177212724-177223920	Y	chr2:197014990-197016725	N
HNRNPA3	chr2:177212724-177223920	Y	chr20:36620402-36621442	N
HNRNPA3	chr2:177212724-177223920	Y	chr4:40142207-40142402	N
HNRNPA3	chr2:177212724-177223920	Y	chr4:82128528-82131631	N
HNRNPA3	chr2:177212724-177223920	Y	chr6:25271821-25273808	N
HNRNPA3	chr2:177212724-177223920	Y	chrX:140032318-140033870	N
HSP90B1	chr12:103930410-103947926	Y	chr1:92108076-92109892	N
HSP90B1	chr12:103930410-103947926	Y	chr15:99797731-99800481	N
IK	chr5:140647829-140662480	Y	chr2:58687309-58689167	N
LSP1	chr11:1853084-1892263	Y	chr5:28926928-28927133	N
PABPC1	chr8:100702795-100722762	Y	chr12:63822021-63823895	Y
PABPC1	chr8:100702795-100722762	Y	chr4:39973444-39974338	N
PABPC1	chr8:100702795-100722762	Y	chr2:146587506-146589310	N

PABPC1	chr8:100702795-100722762	Y	chrX:74583088-74583546	N	
PABPC1	chr8:100702795-100722762	Y	chr4:102896725-102898237	N	
PABPC1	chr8:100702795-100722762	Y	chr3:155309427-155309807	N	
PABPC1	chr8:100702795-100722762	Y	chr9:17589161-17591318	N	
PABPC1	chr8:100702795-100722762	Y	chr4:39973128-39973442	N	
PABPC1	chr8:100702795-100722762	Y	chr13:25096141-25099255	N	
PTMA	chr2:231708516-231713536	Y	chr5:118973796-118974122	Y	
PTMA	chr2:231708516-231713536	Y	chr12:9239986-9240331	Y	
PTMA	chr2:231708516-231713536	Y	chr12:12111163-12111489	Y	
PTMA	chr2:231708516-231713536	Y	chr3:117026698-117027039	Y	
PTMA	chr2:231708516-231713536	Y	chr14:92026422-92027567	Y	
PTMA	chr2:231708516-231713536	Y	chr6:30633632-30633956	N	
PTMA	chr2:231708516-231713536	Y	chr20:18011955-18012284	N	
PTMA	chr2:231708516-231713536	Y	chr13:81689911-81691072	N	
PTMA	chr2:231708516-231713536	Y	chr7:138404195-138404523	N	
PTMA	chr2:231708516-231713536	Y	chr17:76651409-76652084	N	
PTMA	chr2:231708516-231713536	Y	chrX:15653107-15653855	N	
PTMA	chr2:231708516-231713536	Y	chr8:103559099-103559389	N	
RAD21	chr8:116845934-116874776	Y	chrX:100056102-100059587	N	
RBBP8	chr18:22933328-23026486	Y	chr11:109855990-109856384	N	
RBMS1	chr2:160272151-160493807	Y	chr12:66626794-66629010	N	
RPS5	chr19:58386400-58394806	Y	chr1:212997300-212997862	N	
RPS5	chr19:58386400-58394806	Y	chr21:34853847-34854531	N	
RPS5	chr19:58386400-58394806	Y	chr21:35507691-35508399	N	
RPS5	chr19:58386400-58394806	Y	chr6:116579589-116580350	N	
RPS5	chr19:58386400-58394806	Y	chr8:81213033-81213750	N	
RPS5	chr19:58386400-58394806	Y	chr9:4781401-4782105	N	
RPS5	chr19:58386400-58394806	Y	chrX:6340077-6340795	N	
RPS5	chr19:58386400-58394806	Y	chrX:109096516-109097181	N	
