# Supplementary material for: Retro-miRs: novel and functional miRNAs originating from mRNA retrotransposition
Source: Mob DNA. 2023 Sep 8;14:12. doi: 10.1186/s13100-023-00301-w (PMC10486083; doi:10.1186/s13100-023-00301-w)
Supplement: Supplementary file 5 — Additional file 5: Table S4. Conservation of retro-miRs and retrocopies. [file 13100_2023_301_MOESM5_ESM.pdf]

Table S4. Conservation of retro-miRs and retrocopies.

retro-miR	Retrocopy	Retrocopy							retro-miR						
		Human	Chimp	Gorilla	Orangutan	Rhesus	Marmoset	Rodents	Human	Chimp	Gorilla	Orangutan	Rhesus	Marmoset	Rodents
mir-4444-2	HNRNPA3P6	chr3:75214631-7	-	-	-	-	-	-	chr3:75214476-7	-	-	-	-	-	-
mir-4426	RPS27AP5	chr1:192716132-	-	-	-	-	-	-	chr1:192716328-	-	-	-	-	-	-
mir-4426-1	RPS27AP16	chr16:61055399-	chr16:60201463-	-	-	-	-	-	chr16:61055707-	chr16:60201753-	-	-	-	-	-
mir-1244-2	PTMAP2	chr5:118973796-	chr5:119512345-	-	-	-	-	-	chr5:118974586-	chr5:119513334-	-	-	-	-	-
mir-1244-3	PTMAP4	chr12:9239986-9	chr12:9474823-9	CYUI01015004v	-	-	-	-	chr12:9239467-9	chr12:9474428-9	CYUI01015004v	-	-	-	-
mir-1244-4	PTMAP9	chr12:12111163-	chr12:12375093-	-	-	-	-	-	chr12:12111952-	chr12:12376069-	-	-	-	-	-
mir-1244-5	PTMAP8	chr3:117026698-	chr3:120385710-	CYUI01014906v	chr3:15708894-1	-	-	-	chr3:117027474-	chr3:120386687-	CYUI01014906v	chr3:15709058-1	-	-	-
mir-1244-6	RP11-529H20.3	chr14:92026422-	chr14:91576317-	CYUI01014949v	chr14:93322770-	-	-	-	chr14:92027342-	chr14:91577259-	CYUI01014949v	chr14:93323711-	-	-	-
mir-3654	RP11-371A22.1	chr7:133034607-	chr7:134529704-	CYUI01015523v	-	-	-	-	chr7:133034860-	chr7:134530035-	CYUI01015523v	-	-	-	-
mir-3654-1	EEF1GP5	chrX:115702811-	chrX:116313511-	CYUI01000089v	chrX:114670727-	chrX:109559802-	-	-	chrX:115703812-	chrX:116314532-	CYUI01000089v	chrX:114671749-	chrX:109560804-	-	-
mir-572	RNPS1P1	chr4:11368821-1	chr4:11084202-1	CYUI01000005v	-	-	-	-	chr4:11368827-1	chr16:2541092-2	CYUI01000005v	-	-	-	-
mir-622	KRT18P27	chr13:90230384-	chr13:90373952-	CYUI01015187v	chr13:92202953-	chr17:72037066-	-	-	chr13:90231182-	chr13:90374755-	CYUI01015187v	chr13:92202802-	chr17:72037918-	-	-
mir-7161	TATDN2P2	chr6:158609706-	chr6:160364810-	CYUI01015032v	chr6:162047714-	chr4:107336327-	chr4:159665537-	-	chr6:158609707-	chr6:160365189-	CYUI01015032v	chr6:162048113-	chr4:107346872-	chr4:159665538-	-
mir-4788	HMGB3P13	chr3:134437605-	chr3:137949619-	CYUI01014939v	chr3:136839589-	chr2:167554563-	chr17:42034131-	-	chr3:134437827-	chr3:137949845-	CYUI01014939v	chr3:136839815-	chr2:167556046-	chr17:42034879-	-
mir-4468	RCC2P3	chr7:138122202-	chr7:139612214-	CYUI01015182v	chr7:135290828-	chr3:164476602-	chr8:108025628-	-	chr7:138123758-	chr7:139613523-	CYUI01015182v	chr7:135292125-	chr3:164477901-	chr8:108026249-	-
mir-492	KRT19P2	chr12:94834147-	chr12:95064290-	CYUI01014962v	chr12:95974676-	chr11:94645995-	chr9:84853867-8	-	chr12:94834398-	chr12:95064554-	CYUI01014962v	chr12:95974944-	chr11:94645766-	chr9:84854140-8	-
mir-10527	PABPC1P4	chr12:63822021-	chr12:25448463-	CYUI01015004v	chr12:63817020-	chr11:62577992-	chr9:52830816-5	-	chr12:63823663-	chr12:25449068-	CYUI01015004v	chr12:63819051-	chr11:62579882-	chr9:52833049-5	-
