# Supplementary material for: Retro-miRs: novel and functional miRNAs originating from mRNA retrotransposition
Source: Mob DNA. 2023 Sep 8;14:12. doi: 10.1186/s13100-023-00301-w (PMC10486083; doi:10.1186/s13100-023-00301-w)
Supplement: Supplementary file 6 — Additional file 6: Table S5. Dn/Ds analysis. [file 13100_2023_301_MOESM6_ESM.pdf]

Table S5. Dn/Ds analysis

RTCs	Position	Parental Gene	Parental mRNA	miRNA	miRNA type	dNdS RTC	neutrality test	Identity of CDS' nucleotides	Comparison
HNRNPA3P6	chr3:75214462-75215755	HNRNPA3	NM_001330247.1	hsa-mir-4444-2	Retrocopied	0.9273	0.1305	97.73%	Paralog
RPS27AP5	chr1:192716145-192716683	RPS27A	NM_002954.6	hsa-mir-4426	Exon-exon Junction	0.8186	0.2506	98.41%	Paralog
RPS27AP16	chr16:61055415-61055938	RPS27A	NM_002954.6	hsa-mir-4426-1	Exon-exon Junction	0.6005	0.0618	99.05%	Ortholog
PTMAP2	chr5:118973562-118974812	PTMA	NM_002823.5	hsa-mir-1244-2	Retrocopied	1.0802	0.4298	95.75%	Ortholog
PTMAP9	chr12:12110982-12112171	PTMA	NM_002823.5	hsa-mir-1244-4	Retrocopied	0.593	0.2096	97.60%	Ortholog
PTMAP4	chr12:9239321-9240496	PTMA	NM_002823.5	hsa-mir-1244-3	Retrocopied	0.6649	0.2865	97.46%	Ortholog
RP11-371A22.1	chr7:133034529-133035940	EEF1G	NM_001404.5	hsa-mir-3654	Exon-exon Junction	0.8777	0.2925	98.79%	Ortholog
RNPS1P1	chr4:11368602-11373739	RNPS1	XM_005255049.4	hsa-mir-572	Novel	0.6505	0.1624	98.15%	Ortholog
PTMAP8	chr3:117026518-117027707	PTMA	NM_002823.5	hsa-mir-1244-5	Retrocopied	3.4166	0.0481	97.73%	Ortholog
RP11-529H20.3	chr14:92026422-92027567	PTMA	NM_002823.5	hsa-mir-1244-6	Retrocopied	0.1798	0.0093	97.03%	Ortholog
EEF1GP5	chrX:115702791-115704195	EEF1G	NM_001404.5	hsa-mir-3654-1	Exon-exon Junction	1.0182	0.4881	98.55%	Ortholog
KRT18P27	chr13:90230379-90231737	KRT18	NM_199187.2	hsa-mir-622	Exon-exon Junction	0.6624	0.2998	98.61%	Ortholog
TATDN2P2	chr6:158609337-158621992	TATDN2	NM_014760.4	hsa-mir-7161	Novel	0.5527	0.0500	97.30%	Ortholog
HMGB3P13	chr3:134437600-134439066	HMGB3	XM_024452369.1	hsa-mir-4788	Novel	NA	NA	98.59%	Ortholog
RCC2P3	chr7:138122426-138124769	RCC2	NM_018715.4	hsa-mir-4468	Novel	NA	NA	98.39%	Ortholog
KRT19P2	chr12:94834247-94835283	KRT19	NM_002276.5	hsa-mir-492	Exon-exon Junction	2.1887	0.2191	98.50%	Ortholog
PABPC1P4	chr12:63821537-63824341	PABPC1	XM_005250861.3	hsa-mir-10527	Exon-exon Junction	0.4408	0.1052	97.15%	Ortholog
