# Supplementary material for: Retro-miRs: novel and functional miRNAs originating from mRNA retrotransposition
Source: Mob DNA. 2023 Sep 8;14:12. doi: 10.1186/s13100-023-00301-w (PMC10486083; doi:10.1186/s13100-023-00301-w)
Supplement: Supplementary file 7 — Additional file 7: Table S6. Average of miRNAs in normal samples. [file 13100_2023_301_MOESM7_ESM.pdf]

Table S6. Average of miRNAs in normal samples

miR	Sample Frequency	Average Expression	Type
hsa-let-7a-2-3p	341	9.69930833	Others
hsa-let-7a-3p	389	101.3585922	Others
hsa-let-7a-5p	399	51399.66994	Others
hsa-let-7b-3p	390	133.1229594	Others
hsa-let-7b-5p	399	5402.128124	Others
hsa-let-7c-3p	275	7.546473065	Others
hsa-let-7c-5p	399	1622.181068	Others
hsa-let-7d-3p	399	274.0739463	Others
hsa-let-7d-5p	399	1617.532203	Others
hsa-let-7e-3p	376	24.23152978	Others
hsa-let-7e-5p	399	3751.298704	Others
hsa-let-7f-1-3p	380	32.2570405	Others
hsa-let-7f-2-3p	385	27.9151465	Others
hsa-let-7f-5p	399	30100.38566	Others
hsa-let-7g-3p	359	10.77628675	Others
hsa-let-7g-5p	399	6972.69882	Others
hsa-let-7i-3p	385	43.32923174	Others
hsa-let-7i-5p	399	11604.10397	Others
hsa-miR-1-3p	109	13.24242531	Others
hsa-miR-1-5p	9	2.260521889	Others
hsa-miR-100-3p	326	184.7066254	Others
hsa-miR-100-5p	398	113921.9132	Others
hsa-miR-101-3p	399	2851.293918	Others
hsa-miR-101-5p	324	10.82247732	Others
hsa-miR-103a-2-	357	2.483999253	Others
hsa-miR-103a-3p	399	5130.045404	Others
hsa-miR-105-3p	41	15.38442346	Others
hsa-miR-105-5p	80	25.97678546	Others
hsa-miR-106a-3p	45	2.133985867	Others
hsa-miR-106a-5p	399	23.12110263	Others

hsa-miR-106b-3p	399	273.5049862	Others
hsa-miR-106b-5p	399	280.1901032	Others
hsa-miR-107	399	185.6372066	Others
hsa-miR-10a-3p	287	27.80490669	Others
hsa-miR-10a-5p	399	33625.12773	Others
hsa-miR-10b-3p	192	16.50443779	Others
hsa-miR-10b-5p	399	41077.80075	Others
hsa-miR-1178-3p	5	0.3973638	Others
hsa-miR-1178-5p	2	2.289385	Others
hsa-miR-1179	181	2.909595398	Others
hsa-miR-1180-3p	389	53.2346274	Others
hsa-miR-1180-5p	130	1.599999438	Others
hsa-miR-1181	30	1.342515133	Others
hsa-miR-1181-3p	47	1.774368745	Others
hsa-miR-1185-1-	313	69.57724303	Others
hsa-miR-1185-2-	313	8.848061565	Others
hsa-miR-1185-5p	297	35.15367072	Others
hsa-miR-1193	43	1.105445535	Others
hsa-miR-1193-3p	69	2.115240971	Others
hsa-miR-1197	281	27.11590874	Others
hsa-miR-1199-3p	1	0.741915	Others
hsa-miR-1199-5p	19	0.6870378421	Others
hsa-miR-1204	7	0.4646827875	Others
hsa-miR-1205	2	7.225365	Others
hsa-miR-1206	1	8.64596	Others
hsa-miR-1207-5p	1	0.763836	Others
hsa-miR-1208	1	0.798218	Others
hsa-miR-122-3p	29	109.7704211	Others
hsa-miR-122-5p	182	2014.640767	Others
hsa-miR-1224-3p	14	6.870075571	Others
hsa-miR-1224-5p	54	3.295929056	Others

hsa-miR-1225-3p	11	1.243310636	Others
hsa-miR-1225-5p	5	0.7587872	Others
hsa-miR-1226-3p	388	14.84454309	Others
hsa-miR-1226-5p	119	1.387680261	Others
hsa-miR-1227-3p	314	4.355899576	Others
hsa-miR-1228-3p	234	5.18162291	Others
hsa-miR-1228-5p	91	1.759724582	Others
hsa-miR-1229-3p	273	3.033915723	Others
hsa-miR-1229-5p	15	1.855039667	Others
hsa-miR-1231	13	1.675606077	Others
hsa-miR-1231-3p	78	1.566921205	Others
hsa-miR-1233-3p	222	1.92226164	Others
hsa-miR-1233-5p	6	0.5037395	Others
hsa-miR-1236-3p	57	1.237158344	Others
hsa-miR-1236-5p	8	0.530617625	Others
hsa-miR-1237-3p	272	2.848867482	Others
hsa-miR-1237-5p	34	1.649339176	Others
hsa-miR-1238-3p	19	1.400712842	Others
hsa-miR-124-3p	118	162.2990618	Others
hsa-miR-124-5p	36	10.11276403	Others
hsa-miR-1243	83	2.841317952	Others
hsa-miR-1244	51	1.985828157	retro-miR
hsa-miR-1244-4-	25	0.718121788	Others
hsa-miR-1245a	117	2.412535795	Others
hsa-miR-1245a-5	158	4.529769475	Others
hsa-miR-1245b-3	57	1.058513649	Others
hsa-miR-1245b-5	37	1.146333351	Others
hsa-miR-1246	314	0.1091111603	Others
hsa-miR-1247-3p	89	10.77625074	Others
hsa-miR-1247-5p	138	71.59906339	Others
hsa-miR-1248	203	6.227616598	Others

hsa-miR-1249-3p	389	20.63413938	Others
hsa-miR-1249-5p	58	1.225401948	Others
hsa-miR-1250-3p	1	0.249354	Others
hsa-miR-1250-5p	43	1.479999163	Others
hsa-miR-1251-3p	6	1.138191167	Others
hsa-miR-1251-5p	52	8.719202385	Others
hsa-miR-1252-3p	6	0.4532036667	Others
hsa-miR-1252-5p	156	2.076527583	Others
hsa-miR-1253	8	1.73037275	Others
hsa-miR-1254	361	7.494931366	Others
hsa-miR-1255a	295	4.853829	Others
hsa-miR-1255b-5	17	1.336057706	Others
hsa-miR-1256	65	1.2314246	Others
hsa-miR-1256-3p	228	2.005517899	Others
hsa-miR-1257	192	25.81170009	Others
hsa-miR-1258	9	1.163589889	Others
hsa-miR-125a-3p	393	40.2924905	Others
hsa-miR-125a-5p	399	11922.63171	Others
hsa-miR-125b-1-	357	1014.241158	Others
hsa-miR-125b-2-	389	306.7938315	Others
hsa-miR-125b-5p	398	13994.40025	Others
hsa-miR-126-3p	388	1973.890625	Others
hsa-miR-126-5p	392	7291.948926	Others
hsa-miR-1260a	387	21.42050669	Others
hsa-miR-1260b	380	9.774149903	Others
hsa-miR-1261	6	0.1493122333	Others
hsa-miR-1262	287	4.897386983	Others
hsa-miR-1263	5	11.02735	Others
hsa-miR-1264	20	3.37516805	Others
hsa-miR-1265	16	1.409415938	Others
hsa-miR-1266-3p	24	0.6548189583	Others

hsa-miR-1266-5p	116	11.76574879	Others
hsa-miR-1267	101	1.884358495	Others
hsa-miR-1268a	5	0.8218696	Others
hsa-miR-1268b	5	0.7845952	Others
hsa-miR-1269a	108	65.10650478	Others
hsa-miR-1269b	106	0.7432535519	Others
hsa-miR-127-3p	399	20762.69898	Others
hsa-miR-127-5p	323	95.6359579	Others
hsa-miR-1270	277	7.927904105	Others
hsa-miR-1271-3p	91	2.976410451	Others
hsa-miR-1271-5p	391	284.546846	Others
hsa-miR-1272	14	44.25842636	Others
hsa-miR-1273a	4	1.595197	Others
hsa-miR-1273c	138	1.464317899	Others
hsa-miR-1273c-3	59	1.613089407	Others
hsa-miR-1273d	47	1.116681511	Others
hsa-miR-1273h-3	67	20.04937094	Others
hsa-miR-1273h-5	12	0.8170981667	Others
hsa-miR-1275	64	1.733321625	Others
hsa-miR-1276	346	9.275367179	Others
hsa-miR-1277-3p	142	1.581013472	Others
hsa-miR-1277-5p	204	6.550013127	Others
hsa-miR-1278	208	1.628660372	Others
hsa-miR-1278-5p	52	1.110622231	Others
hsa-miR-128-1-5	390	26.56054758	Others
hsa-miR-128-2-5	52	6.002896115	Others
hsa-miR-128-3p	399	981.1413028	Others
hsa-miR-1281	2	3.729175	Others
hsa-miR-1282	1	0.379531	Others
hsa-miR-1283	77	27.15339196	Others
hsa-miR-1283-2-	43	18.05420847	Others

hsa-miR-1284	213	2.661632333	Others
hsa-miR-1285-3p	363	26.64916158	Others
hsa-miR-1285-5p	5	1.3799188	Others
hsa-miR-1286	157	7.667502	Others
hsa-miR-1287-3p	37	1.514605973	Others
hsa-miR-1287-5p	325	7.260495394	Others
hsa-miR-1288-3p	32	0.9356834781	Others
hsa-miR-1288-5p	22	0.9199686818	Others
hsa-miR-1289	60	0.7582205167	Others
hsa-miR-1289-1-	133	1.227749556	Others
hsa-miR-129-1-3	263	1.575646622	Others
hsa-miR-129-2-3	266	17.14214863	Others
hsa-miR-129-5p	241	21.58097018	Others
hsa-miR-1290	141	0.01172496191	Others
hsa-miR-1291	228	3.382109453	Others
hsa-miR-1292-3p	92	1.779470804	Others
hsa-miR-1292-5p	233	2.211445215	Others
hsa-miR-1293	218	6.960957963	Others
hsa-miR-1293-3p	299	14.27731788	Others
hsa-miR-1294	281	2.78817026	Others
hsa-miR-1295a	54	0.9752857778	Others
hsa-miR-1295b-3	3	0.454784	Others
hsa-miR-1295b-5	9	6.223094556	Others
hsa-miR-1296-3p	117	1.467754585	Others
hsa-miR-1296-5p	395	95.77644163	Others
hsa-miR-1297	14	0.02808131138	Others
hsa-miR-1298-3p	26	23.22324969	Others
hsa-miR-1298-5p	77	97.63570771	Others
hsa-miR-1299	126	3.618216595	Others
hsa-miR-1301-3p	397	89.11136244	Others
hsa-miR-1301-5p	30	1.140302633	Others

hsa-miR-1302	45	0.8762730222	Others
hsa-miR-1303	295	13.01876662	Others
hsa-miR-1304-3p	399	125.0264012	Others
hsa-miR-1304-5p	348	5.882806489	Others
hsa-miR-1305	183	6.141864087	Others
hsa-miR-1306-3p	293	2.65030486	Others
hsa-miR-1306-5p	359	8.297043535	Others
hsa-miR-1307-3p	399	268.3750933	Others
hsa-miR-1307-5p	398	683.6662198	Others
hsa-miR-130a-3p	399	1939.812467	Others
hsa-miR-130a-5p	264	5.342975652	Others
hsa-miR-130b-3p	399	720.557412	Others
hsa-miR-130b-5p	399	236.9143433	Others
hsa-miR-132-3p	398	165.8062778	Others
hsa-miR-132-5p	382	20.89056157	Others
hsa-miR-1322	111	1.795348963	Others
hsa-miR-1323	67	422.0435504	Others
hsa-miR-133a-3p	268	197.1734138	Others
hsa-miR-133a-5p	33	14.48819094	Others
hsa-miR-133b	153	3.541859487	Others
hsa-miR-134-3p	210	4.526334429	Others
hsa-miR-134-5p	340	174.2387118	Others
hsa-miR-1343-3p	374	10.83398745	Others
hsa-miR-1343-5p	46	1.843758087	Others
hsa-miR-135a-3p	41	2.995396268	Others
hsa-miR-135a-5p	258	10.21144576	Others
hsa-miR-135b-3p	155	8.183405826	Others
hsa-miR-135b-5p	259	22.53643232	Others
hsa-miR-136-3p	361	839.6121462	Others
hsa-miR-136-5p	323	165.9813524	Others
hsa-miR-137	270	27.12143061	Others

hsa-miR-138-1-3	256	19.48305328	Others
hsa-miR-138-2-3	55	0.8986811673	Others
hsa-miR-138-5p	385	1189.814644	Others
hsa-miR-139-3p	83	12.2290541	Others
hsa-miR-139-5p	243	51.28733558	Others
hsa-miR-140-3p	399	1760.381835	Others
hsa-miR-140-5p	385	50.70355151	Others
hsa-miR-141-3p	332	3143.284419	Others
hsa-miR-141-5p	161	155.904508	Others
hsa-miR-142-3p	195	1195.500539	Others
hsa-miR-142-5p	346	7507.45401	Others
hsa-miR-143-3p	399	24016.27384	Others
hsa-miR-143-5p	265	57.0692496	Others
hsa-miR-144-3p	98	14.21579719	Others
hsa-miR-144-5p	153	12.76996	Others
hsa-miR-145-3p	330	187.6986524	Others
hsa-miR-145-5p	349	408.5490505	Others
hsa-miR-1468-3p	1	2.15663	Others
hsa-miR-1468-5p	383	22.77886013	Others
hsa-miR-1469	11	1.521100545	Others
hsa-miR-146a-3p	152	14.99831467	Others
hsa-miR-146a-5p	392	3773.30834	Others
hsa-miR-146b-3p	370	97.21599984	Others
hsa-miR-146b-5p	399	5548.998465	Others
hsa-miR-147a	263	0.01143810307	Others
hsa-miR-147b	277	7.902148769	Others
hsa-miR-148a-3p	399	11333.96303	Others
hsa-miR-148a-5p	394	190.5505514	Others
hsa-miR-148b-3p	399	1599.267125	Others
hsa-miR-148b-5p	396	131.6309137	Others
hsa-miR-149-3p	107	1.951552364	Others

hsa-miR-149-5p	395	491.6732782	Others
hsa-miR-150-3p	45	93.64113989	Others
hsa-miR-150-5p	301	10602.07122	Others
hsa-miR-151a-3p	399	6630.052148	Others
hsa-miR-151a-5p	399	6543.982985	Others
hsa-miR-151b	399	14.53287081	Others
hsa-miR-152-3p	398	278.7958159	Others
hsa-miR-152-5p	389	40.92609976	Others
hsa-miR-153-3p	230	9.3796923	Others
hsa-miR-153-5p	49	3.252664224	Others
hsa-miR-1537-3p	91	3.164635264	Others
hsa-miR-1537-5p	60	1.36505335	Others
hsa-miR-1538	255	2.793213102	Others
hsa-miR-1539	8	0.73855325	Others
hsa-miR-154-3p	233	6.32527715	Others
hsa-miR-154-5p	288	20.24521873	Others
hsa-miR-155-3p	122	5.1807795	Others
hsa-miR-155-5p	394	2897.683043	Others
hsa-miR-1587	11	0.2418424909	Others
hsa-miR-15a-3p	166	1.468212295	Others
hsa-miR-15a-5p	399	600.2889125	Others
hsa-miR-15b-3p	397	88.02310107	Others
hsa-miR-15b-5p	399	461.1617318	Others
hsa-miR-16-1-3p	268	2.905715254	Others
hsa-miR-16-2-3p	398	103.5726114	Others
hsa-miR-16-5p	399	6456.310301	Others
hsa-miR-17-3p	399	165.6050009	Others
hsa-miR-17-5p	399	584.0162805	Others
hsa-miR-181a-2-	397	625.431079	Others
hsa-miR-181a-3p	398	555.9112859	Others
hsa-miR-181a-5p	399	21857.48235	Others

hsa-miR-181b-2-	359	4.102113579	Others
hsa-miR-181b-3p	358	17.91252908	Others
hsa-miR-181b-5p	399	2931.448615	Others
hsa-miR-181c-3p	397	122.1529592	Others
hsa-miR-181c-5p	399	783.4742531	Others
hsa-miR-181d-3p	119	1.443590597	Others
hsa-miR-181d-5p	399	137.1600255	Others
hsa-miR-182-3p	91	4.753076714	Others
hsa-miR-182-5p	395	7549.92553	Others
hsa-miR-1825	6	0.8559405	Others
hsa-miR-1827	157	0.003981252766	Others
hsa-miR-183-3p	177	34.43382356	Others
hsa-miR-183-5p	368	1992.731561	Others
hsa-miR-184	183	95.91990884	Others
hsa-miR-185-3p	391	12.02433246	Others
hsa-miR-185-5p	395	72.38302203	Others
hsa-miR-186-3p	251	3.170291295	Others
hsa-miR-186-5p	399	5579.952411	Others
hsa-miR-187-3p	199	43.97945605	Others
hsa-miR-187-5p	25	3.9390942	Others
hsa-miR-188-3p	259	3.484785425	Others
hsa-miR-188-5p	376	18.07071172	Others
hsa-miR-18a-3p	392	38.66872724	Others
hsa-miR-18a-5p	388	91.66927593	Others
hsa-miR-18b-3p	59	26.38816235	Others
hsa-miR-18b-5p	388	3.689986428	Others
hsa-miR-1908-3p	318	12.57453752	Others
hsa-miR-1908-5p	358	16.99574293	Others
hsa-miR-1909-3p	72	1.606059903	Others
hsa-miR-1909-5p	69	1.124160609	Others
hsa-miR-190a-3p	191	2.674696515	Others

hsa-miR-190a-5p	380	77.49587083	Others
hsa-miR-190b	289	5.536113564	Others
hsa-miR-191-3p	391	18.70923188	Others
hsa-miR-191-5p	399	27426.57972	Others
hsa-miR-1910-3p	153	2.240837137	Others
hsa-miR-1910-5p	355	31.97103163	Others
hsa-miR-1911-3p	28	5.663165786	Others
hsa-miR-1911-5p	54	7.869759981	Others
hsa-miR-1912	19	14.73308858	Others
hsa-miR-1913	180	2.322559606	Others
hsa-miR-1914-3p	35	0.6651021714	Others
hsa-miR-1914-5p	201	2.747610557	Others
hsa-miR-1915-3p	30	1.8652417	Others
hsa-miR-1915-5p	81	2.466350691	Others
hsa-miR-192-3p	93	3.710746462	Others
hsa-miR-192-5p	399	4245.155166	Others
hsa-miR-193a-3p	387	631.3762329	Others
hsa-miR-193a-5p	398	302.36995	Others
hsa-miR-193b-3p	397	636.064757	Others
hsa-miR-193b-5p	356	13.98677871	Others
hsa-miR-194-3p	107	4.63636186	Others
hsa-miR-194-5p	386	100.2821766	Others
hsa-miR-195-3p	286	11.59356127	Others
hsa-miR-195-5p	392	79.42209048	Others
hsa-miR-196a-3p	183	30.4294078	Others
hsa-miR-196a-5p	318	202.4525235	Others
hsa-miR-196b-3p	144	2.380206521	Others
hsa-miR-196b-5p	318	144.8233777	Others
hsa-miR-197-3p	399	686.0983759	Others
hsa-miR-197-5p	273	2.903306333	Others
hsa-miR-1972-1-	3	0.654421	Others

hsa-miR-1973	206	0.07743504481	Others
hsa-miR-1976	199	7.112459879	Others
hsa-miR-198	5	2.1183798	Others
hsa-miR-199a-3	395	5568.391574	Others
hsa-miR-199a-5	377	2070.851954	Others
hsa-miR-199b-3	395	2415.743507	Others
hsa-miR-199b-5	379	2060.036128	Others
hsa-miR-19a-3p	399	216.3989148	Others
hsa-miR-19a-5p	278	6.400838176	Others
hsa-miR-19b-1-5	247	2.950212093	Others
hsa-miR-19b-2-5	48	0.292059811	Others
hsa-miR-19b-3p	399	616.9823663	Others
hsa-miR-200a-3	329	214.874201	Others
hsa-miR-200a-5	135	37.93217513	Others
hsa-miR-200b-3	367	1285.974055	Others
hsa-miR-200b-5	128	19.00696205	Others
hsa-miR-200c-3	315	791.1789382	Others
hsa-miR-200c-5	89	10.47836843	Others
hsa-miR-202-3p	24	62.31715596	Others
hsa-miR-202-5p	26	28.11070558	Others
hsa-miR-203a-3	265	932.4409093	Others
hsa-miR-203a-5	72	10.26052836	Others
hsa-miR-203b-3	60	4.129087417	Others
hsa-miR-203b-5	4	0.38302875	Others
hsa-miR-204-3p	55	6.978850618	Others
hsa-miR-204-5p	363	1086.449912	Others
hsa-miR-205-3p	55	41.82848093	Others
hsa-miR-205-5p	253	11106.0826	Others
hsa-miR-206	35	321.3919431	Others
hsa-miR-208a-3	4	0.7324279853	Others
hsa-miR-208a-5	2	0.440267	Others

hsa-miR-208b-3p	56	5.114529679	Others
hsa-miR-20a-3p	337	7.434448807	Others
hsa-miR-20a-5p	399	710.7124231	Others
hsa-miR-20b-3p	59	13.49828434	Others
hsa-miR-20b-5p	355	100.9987936	Others
hsa-miR-21-3p	399	8213.450197	Others
hsa-miR-21-5p	399	85734.94269	Others
hsa-miR-210-3p	399	310.7177929	Others
hsa-miR-210-5p	384	29.89434574	Others
hsa-miR-211-3p	11	15.09413391	Others
hsa-miR-211-5p	172	418.2224006	Others
hsa-miR-2110	385	9.8548522	Others
hsa-miR-2110-3p	393	18.42186592	Others
hsa-miR-2113	25	11.49124385	Others
hsa-miR-2114-3p	137	4.976139715	Others
hsa-miR-2114-5p	173	6.38484778	Others
hsa-miR-2115-3p	42	5.221084833	Others
hsa-miR-2115-5p	21	4.175269476	Others
hsa-miR-2116-3p	358	10.1692997	Others
hsa-miR-2116-5p	214	1.937088393	Others
hsa-miR-2117-5p	3	0.8840703333	Others
hsa-miR-212-3p	376	20.87321354	Others
hsa-miR-212-5p	380	38.61143721	Others
hsa-miR-214-3p	312	302.3850217	Others
hsa-miR-214-5p	278	111.2013552	Others
hsa-miR-215-3p	20	11.9815343	Others
hsa-miR-215-5p	394	115.7348673	Others
hsa-miR-216a-3p	118	46.22399146	Others
hsa-miR-216a-5p	143	22.0679935	Others
hsa-miR-216b-3p	74	3.126122292	Others
hsa-miR-216b-5p	109	8.395905009	Others

hsa-miR-217	114	33.5490784	Others
hsa-miR-218-1-3	317	25.85961102	Others
hsa-miR-218-2-3	147	0.5501793033	Others
hsa-miR-218-5p	372	83.75464045	Others
hsa-miR-219a-1-	352	6.431720344	Others
hsa-miR-219a-2-	32	136.9382153	Others
hsa-miR-219a-5p	224	2.782834321	Others
hsa-miR-219b-3p	224	4.807490763	Others
hsa-miR-219b-5p	202	2.00637452	Others
hsa-miR-22-3p	399	79222.67835	Others
hsa-miR-22-5p	396	91.92124371	Others
hsa-miR-221-3p	399	8833.266077	Others
hsa-miR-221-5p	398	860.8748245	Others
hsa-miR-222-3p	399	14457.6072	Others
hsa-miR-222-5p	379	61.0068702	Others
hsa-miR-223-3p	163	3227.202269	Others
hsa-miR-223-5p	50	48.51872474	Others
hsa-miR-224-3p	273	7.43256548	Others
hsa-miR-224-5p	374	765.5244856	Others
hsa-miR-2276-3p	149	2.84700147	Others
hsa-miR-2276-5p	27	0.7765128889	Others
hsa-miR-2277-3p	283	3.258642505	Others
hsa-miR-2277-5p	388	15.55399306	Others
hsa-miR-2278	141	1.586680064	Others
hsa-miR-2355-3p	343	4.903585848	Others
hsa-miR-2355-5p	383	26.24450184	Others
hsa-miR-2392	15	0.3584080667	Others
hsa-miR-23a-3p	399	2213.905281	Others
hsa-miR-23a-5p	387	52.50670032	Others
hsa-miR-23b-3p	399	1025.347839	Others
hsa-miR-23b-5p	287	4.411192659	Others

hsa-miR-23c	398	0.7393842116	Others
hsa-miR-24-1-5p	305	4.309592927	Others
hsa-miR-24-2-5p	392	147.845661	Others
hsa-miR-24-3p	399	1135.901213	Others
hsa-miR-2467-3p	40	0.91942205	Others
hsa-miR-2467-5p	386	20.25236245	Others
hsa-miR-25-3p	399	3897.538343	Others
hsa-miR-25-5p	394	33.20425332	Others
hsa-miR-2681-3p	14	2.121826571	Others
hsa-miR-2681-5p	19	1.534472526	Others
hsa-miR-2682-3p	209	6.950191086	Others
hsa-miR-2682-5p	266	17.3981265	Others
hsa-miR-26a-1-3	317	4.608300376	Others
hsa-miR-26a-2-3	377	24.30454622	Others
hsa-miR-26a-5p	399	25904.75451	Others
hsa-miR-26b-3p	390	42.65033103	Others
hsa-miR-26b-5p	399	3040.450326	Others
hsa-miR-27a-3p	399	4507.518597	Others
hsa-miR-27a-5p	396	104.7119206	Others
hsa-miR-27b-3p	399	24242.81582	Others
hsa-miR-27b-5p	396	120.5845464	Others
hsa-miR-28-3p	399	5401.715469	Others
hsa-miR-28-5p	399	552.7809313	Others
hsa-miR-2861	2	1.410394	Others
hsa-miR-2909-3p	282	0.008245784471	Others
hsa-miR-296-3p	351	30.8250013	Others
hsa-miR-296-5p	367	27.46174523	Others
hsa-miR-297	2	2.64327	Others
hsa-miR-298	3	3.039524667	Others
hsa-miR-298-3p	31	1.307927839	Others
hsa-miR-299-3p	314	91.01194935	Others

hsa-miR-299-5p	294	42.54708912	Others
hsa-miR-29a-3p	399	8025.972505	Others
hsa-miR-29a-5p	378	47.1144684	Others
hsa-miR-29b-1-5	383	17.66983345	Others
hsa-miR-29b-2-5	257	4.335192166	Others
hsa-miR-29b-3p	393	446.8663022	Others
hsa-miR-29c-3p	399	495.7244133	Others
hsa-miR-29c-5p	362	21.01091946	Others
hsa-miR-300	143	0.0156516654	Others
hsa-miR-301a-3p	393	648.6621295	Others
hsa-miR-301a-5p	330	6.288607624	Others
hsa-miR-301b-3p	394	120.8910974	Others
hsa-miR-301b-5p	167	2.648328677	Others
hsa-miR-302a-3p	74	4204.35228	Others
hsa-miR-302a-5p	85	3229.974271	Others
hsa-miR-302b-3p	89	3910.431933	Others
hsa-miR-302b-5p	16	64.57244475	Others
hsa-miR-302c-3p	94	2102.818679	Others
hsa-miR-302c-5p	30	1588.30855	Others
hsa-miR-302d-3p	108	3472.095705	Others
hsa-miR-302d-5p	18	339.3237064	Others
hsa-miR-302e	42	0.7394968114	Others
hsa-miR-3064-3p	99	0.9969988788	Others
hsa-miR-3064-5p	135	1.2440214	Others
hsa-miR-3065-3p	282	7.506203262	Others
hsa-miR-3065-5p	265	7.818401894	Others
hsa-miR-3074-3p	169	1.862190763	Others
hsa-miR-3074-5p	272	3.493014268	Others
hsa-miR-30a-3p	398	266.28293	Others
hsa-miR-30a-5p	399	13165.00934	Others
hsa-miR-30b-3p	343	9.133582251	Others

hsa-miR-30b-5p	399	1020.177108	Others
hsa-miR-30c-1-3	396	22.21884093	Others
hsa-miR-30c-2-3	392	40.13264365	Others
hsa-miR-30c-5p	399	3213.581554	Others
hsa-miR-30d-3p	392	67.89687388	Others
hsa-miR-30d-5p	399	8059.111624	Others
hsa-miR-30e-3p	398	199.7544702	Others
hsa-miR-30e-5p	399	5139.979835	Others
hsa-miR-31-3p	337	181.2633521	Others
hsa-miR-31-5p	387	7349.659624	Others
hsa-miR-3115	77	0.8610145325	Others
hsa-miR-3116	129	2.910793527	Others
hsa-miR-3117-3p	270	28.39183725	Others
hsa-miR-3117-5p	1	0.333893	Others
hsa-miR-3118	8	0.295297875	Others
hsa-miR-3119	3	1.938389887	Others
hsa-miR-3120-3p	57	1.493188386	Others
hsa-miR-3120-5p	16	0.5704444634	Others
hsa-miR-3121-3p	74	1.012469099	Others
hsa-miR-3121-5p	5	0.5459064	Others
hsa-miR-3122	23	0.9009868261	Others
hsa-miR-3124-3p	16	3.883619125	Others
hsa-miR-3124-5p	65	0.8609805692	Others
hsa-miR-3125	97	1.224816907	Others
hsa-miR-3126-3p	56	1.148433339	Others
hsa-miR-3126-5p	181	2.506039552	Others
hsa-miR-3127-3p	98	1.261515071	Others
hsa-miR-3127-5p	227	3.558654019	Others
hsa-miR-3128	125	1.22178132	Others
hsa-miR-3129-3p	294	8.526999201	Others
hsa-miR-3129-5p	202	2.728541485	Others

hsa-miR-3130-3p	166	1.487049627	Others
hsa-miR-3130-5p	272	2.646780511	Others
hsa-miR-3131	59	2.215687915	Others
hsa-miR-3132	7	1.779583857	Others
hsa-miR-3132-3p	37	1.500663703	Others
hsa-miR-3133	166	1.382105614	Others
hsa-miR-3134	27	0.7770093333	Others
hsa-miR-3135a	67	0.9340981343	Others
hsa-miR-3135b	10	0.0001596808013	Others
hsa-miR-3136-3p	6	0.4758383333	Others
hsa-miR-3136-5p	193	1.766670948	Others
hsa-miR-3137	10	0.8836588	Others
hsa-miR-3138	209	2.034936986	Others
hsa-miR-3138-5p	216	2.032479505	Others
hsa-miR-3139	119	1.690463538	Others
hsa-miR-3140-3p	138	1.447719833	Others
hsa-miR-3140-5p	40	0.85587565	Others
hsa-miR-3141	72	0.8180657083	Others
hsa-miR-3142	41	0.365023439	Others
hsa-miR-3142-5p	42	0.3893102619	Others
hsa-miR-3143	177	2.481039599	Others
hsa-miR-3144-3p	52	1.917420923	Others
hsa-miR-3144-5p	63	1.903519206	Others
hsa-miR-3145-3p	152	1.522395125	Others
hsa-miR-3145-5p	155	1.479470832	Others
hsa-miR-3146	37	0.9020275946	Others
hsa-miR-3147	2	1.1419585	Others
hsa-miR-3148	7	0.3920821429	Others
hsa-miR-3148-3p	5	0.3931848	Others
hsa-miR-3149	32	0.4506243125	Others
hsa-miR-3150a-3	128	1.823394086	Others

hsa-miR-3150a-5	172	1.652343424	Others
hsa-miR-3150b-3	110	5.110145125	Others
hsa-miR-3150b-5	22	0.8149491855	Others
hsa-miR-3151-3p	79	2.220559582	Others
hsa-miR-3151-5p	32	1.191384656	Others
hsa-miR-3152-3p	60	1.43200785	Others
hsa-miR-3152-5p	238	15.3148295	Others
hsa-miR-3153	2	4.004015	Others
hsa-miR-3153-5p	41	1.549851146	Others
hsa-miR-3154	76	1.756951539	Others
hsa-miR-3155a	183	2.320692699	Others
hsa-miR-3155b	25	0.1590819309	Others
hsa-miR-3156-3p	15	0.6957314667	Others
hsa-miR-3156-5p	9	0.316216	Others
hsa-miR-3157-3p	232	2.199774307	Others
hsa-miR-3157-5p	193	1.45152915	Others
hsa-miR-3158-3p	389	16.72572922	Others
hsa-miR-3158-5p	67	0.8450193433	Others
hsa-miR-3159	167	1.359548838	Others
hsa-miR-3160-3p	145	1.339974497	Others
hsa-miR-3160-5p	16	0.3829088125	Others
hsa-miR-3161	110	5.292284873	Others
hsa-miR-3162-3p	16	1.227486125	Others
hsa-miR-3162-5p	26	0.9650093462	Others
hsa-miR-3163	139	1.996691165	Others
hsa-miR-3164	27	0.8178066667	Others
hsa-miR-3164-3p	58	0.8691167241	Others
hsa-miR-3165	61	0.8534149508	Others
hsa-miR-3166	58	2.666157276	Others
hsa-miR-3167	74	1.590775581	Others
hsa-miR-3168	343	10.40711875	Others

hsa-miR-3170	35	1.336704057	Others
hsa-miR-3170-3p	118	1.563605415	Others
hsa-miR-3171	20	1.7950816	Others
hsa-miR-3173-3p	96	1.333801385	Others
hsa-miR-3173-5p	294	3.806767656	Others
hsa-miR-3174	282	3.513586085	Others
hsa-miR-3175	169	1.780504053	Others
hsa-miR-3176	384	24.17547086	Others
hsa-miR-3177-3p	292	4.118425445	Others
hsa-miR-3177-5p	146	1.326280877	Others
hsa-miR-3178	18	1.482428333	Others
hsa-miR-3178-3p	10	1.4686941	Others
hsa-miR-3179	84	1.304711833	Others
hsa-miR-3180	274	3.044441841	Others
hsa-miR-3180-3p	274	4.579103471	Others
hsa-miR-3180-5p	134	1.8439033	Others
hsa-miR-3181	128	1.454698648	Others
hsa-miR-3182	2	1.1869085	Others
hsa-miR-3183	67	1.066230806	Others
hsa-miR-3184-3p	12	1.250346833	Others
hsa-miR-3184-5p	31	1.070664806	Others
hsa-miR-3185	1	0.256387	Others
hsa-miR-3186-3p	4	0.855905	Others
hsa-miR-3186-5p	1	0.240645	Others
hsa-miR-3187-3p	261	4.113860644	Others
hsa-miR-3187-5p	62	0.7548381823	Others
hsa-miR-3188	134	2.044047694	Others
hsa-miR-3189-3p	54	1.177610481	Others
hsa-miR-3189-5p	10	2.1783842	Others
hsa-miR-3190-3p	128	1.324931914	Others
hsa-miR-3190-5p	64	0.8554430228	Others

hsa-miR-3191-3p	52	0.9406881538	Others
hsa-miR-3191-5p	60	0.9477231667	Others
hsa-miR-3192-3p	28	0.5914040357	Others
hsa-miR-3192-5p	75	0.9410772831	Others
hsa-miR-3193	115	1.7847108	Others
hsa-miR-3194-3p	75	1.64224836	Others
hsa-miR-3194-5p	159	4.782274031	Others
hsa-miR-3195	9	1.760378333	Others
hsa-miR-3196	3	2.703523333	Others
hsa-miR-3197	23	0.7790962174	Others
hsa-miR-3198	13	0.6602796154	Others
hsa-miR-3199	226	1.753113606	Others
hsa-miR-3199-1-	136	0.5724298391	Others
hsa-miR-32-3p	336	7.994208818	Others
hsa-miR-32-5p	360	33.55130569	Others
hsa-miR-3200-3p	373	20.88499021	Others
hsa-miR-3200-5p	161	2.081417255	Others
hsa-miR-3201	6	1.0862645	Others
hsa-miR-3202	59	2.454416559	Others
hsa-miR-320a	399	1419.698291	Others
hsa-miR-320b	399	7.085470571	Others
hsa-miR-320c	399	1.87645315	Others
hsa-miR-320d	397	4.324257348	Others
hsa-miR-320e	388	0.03007109061	Others
hsa-miR-323a-3p	333	107.7407293	Others
hsa-miR-323a-5p	187	4.134194358	Others
hsa-miR-323b-3p	316	53.10160894	Others
hsa-miR-323b-5p	26	1.952834923	Others
hsa-miR-324-3p	399	54.91154326	Others
hsa-miR-324-5p	396	33.48071165	Others
hsa-miR-326	365	52.79722048	Others

hsa-miR-328-3p	397	67.72649665	Others
hsa-miR-328-5p	78	1.028403936	Others
hsa-miR-329-3p	314	68.31637827	Others
hsa-miR-329-5p	178	3.619307393	Others
hsa-miR-330-3p	398	34.70826244	Others
hsa-miR-330-5p	396	63.69006389	Others
hsa-miR-331-3p	398	330.0460598	Others
hsa-miR-331-5p	385	12.84558577	Others
hsa-miR-335-3p	384	612.8460868	Others
hsa-miR-335-5p	372	214.7047858	Others
hsa-miR-337-3p	308	89.61204502	Others
hsa-miR-337-5p	285	22.65548732	Others
hsa-miR-338-3p	224	140.7777263	Others
hsa-miR-338-5p	147	43.43516647	Others
hsa-miR-339-3p	399	181.9811554	Others
hsa-miR-339-5p	397	112.1011685	Others
hsa-miR-33a-3p	334	8.694057832	Others
hsa-miR-33a-5p	372	35.71086104	Others
hsa-miR-33b-3p	355	12.03591843	Others
hsa-miR-33b-5p	373	30.42687739	Others
hsa-miR-340-3p	385	27.02979013	Others
hsa-miR-340-5p	398	623.7147266	Others
hsa-miR-342-3p	399	941.1939872	Others
hsa-miR-342-5p	372	29.07929826	Others
hsa-miR-345-3p	175	3.73957912	Others
hsa-miR-345-5p	399	756.6411358	Others
hsa-miR-346	61	8.363493262	Others
hsa-miR-34a-3p	363	17.5764296	Others
hsa-miR-34a-5p	399	609.8419622	Others
hsa-miR-34b-3p	295	10.20401633	Others
hsa-miR-34b-5p	326	28.82680229	Others

hsa-miR-34c-3p	242	5.126712864	Others
hsa-miR-34c-5p	390	319.1484476	Others
hsa-miR-3529-3p	7	0.5660562857	Others
hsa-miR-3529-5p	25	1.89778496	Others
hsa-miR-3591-3p	2	0.4656425	Others
hsa-miR-3591-5p	43	1.2866935	Others
hsa-miR-3605-3p	385	18.44564519	Others
hsa-miR-3605-5p	374	13.34001427	Others
hsa-miR-3606-3p	4	1.15514	Others
hsa-miR-3606-5p	26	0.8784254231	Others
hsa-miR-3607-3p	394	83.33646279	Others
hsa-miR-3607-5p	132	2.529625826	Others
hsa-miR-3609	236	6.179552831	Others
hsa-miR-361-3p	399	342.8743817	Others
hsa-miR-361-5p	393	303.9988229	Others
hsa-miR-3610	48	1.115829125	Others
hsa-miR-3611	197	1.667768289	Others
hsa-miR-3611-5p	30	1.082509267	Others
hsa-miR-3612	14	1.128830489	Others
hsa-miR-3612-3p	5	2.8511394	Others
hsa-miR-3613-3p	327	6.643155382	Others
hsa-miR-3613-5p	361	15.59065582	Others
hsa-miR-3614-3p	90	1.654677656	Others
hsa-miR-3614-5p	184	9.579144783	Others
hsa-miR-3615	378	24.90124354	Others
hsa-miR-3616-3p	27	1.120136481	Others
hsa-miR-3616-5p	51	1.003964255	Others
hsa-miR-3617-3p	24	1.476499375	Others
hsa-miR-3617-5p	81	1.468486379	Others
hsa-miR-3618	9	0.5837326667	Others
hsa-miR-3619-3p	85	1.684502941	Others

hsa-miR-3619-5p	155	1.885315071	Others
hsa-miR-362-3p	299	5.931888933	Others
hsa-miR-362-5p	382	26.94103781	Others
hsa-miR-3620-3p	309	3.927902052	Others
hsa-miR-3620-5p	119	1.319866134	Others
hsa-miR-3621	2	0.395634	Others
hsa-miR-3622a-3	89	2.291267481	Others
hsa-miR-3622a-5	120	2.633581525	Others
hsa-miR-3622b-3	36	1.071064056	Others
hsa-miR-3622b-5	16	0.6853446687	Others
hsa-miR-363-3p	265	240.9587437	Others
hsa-miR-363-5p	22	5.189669045	Others
hsa-miR-3646	12	0.9524416667	Others
hsa-miR-3649-5p	2	0.1203260476	Others
hsa-miR-3651	364	12.87841427	Others
hsa-miR-3652	68	1.510659412	Others
hsa-miR-3653-3p	219	6.245959828	Others
hsa-miR-3653-5p	110	3.857644973	Others
hsa-miR-3654	56	1.609513946	retro-miR
hsa-miR-3655	20	1.2867777	Others
hsa-miR-3656	4	0.949584	Others
hsa-miR-3657	19	0.9105378421	Others
hsa-miR-3657-5p	20	0.9494336	Others
hsa-miR-3658	1	0.273223	Others
hsa-miR-3658-3p	8	0.69927075	Others
hsa-miR-3659	50	3.2756445	Others
hsa-miR-365a-3p	399	126.6857484	Others
hsa-miR-365a-5p	303	5.575964205	Others
hsa-miR-365b-3p	399	126.7780924	Others
hsa-miR-365b-5p	355	10.50362257	Others
hsa-miR-3660	35	1.105655857	Others

hsa-miR-3661	367	8.270914531	Others
hsa-miR-3662	169	11.84137778	Others
hsa-miR-3663-3p	25	3.77658276	Others
hsa-miR-3663-5p	4	2.6537015	Others
hsa-miR-3664-3p	146	3.315094808	Others
hsa-miR-3664-5p	51	1.231129765	Others
hsa-miR-3665	12	1.200361917	Others
hsa-miR-3666	1	0.838687	Others
hsa-miR-3667-3p	49	2.785006061	Others
hsa-miR-3667-5p	43	1.338921488	Others
hsa-miR-3668	2	0.8641195	Others
hsa-miR-367-3p	24	88.82093503	Others
hsa-miR-367-5p	6	6.51757	Others
hsa-miR-3671	2	3.83188	Others
hsa-miR-3672	6	0.2081558333	Others
hsa-miR-3674	1	0.501772	Others
hsa-miR-3675-3p	29	2.098539207	Others
hsa-miR-3675-5p	65	2.559521723	Others
hsa-miR-3677-3p	348	10.51923519	Others
hsa-miR-3677-5p	60	0.975146	Others
hsa-miR-3678-3p	98	1.068830684	Others
hsa-miR-3678-5p	92	0.9535461196	Others
hsa-miR-3679-3p	59	0.8253634407	Others
hsa-miR-3679-5p	165	2.38478322	Others
hsa-miR-3680-3p	24	1.558685833	Others
hsa-miR-3680-5p	30	0.8220174333	Others
hsa-miR-3681-3p	5	0.6024132	Others
hsa-miR-3681-5p	96	2.527921813	Others
hsa-miR-3682-3p	25	1.0585146	Others
hsa-miR-3682-5p	13	1.369745231	Others
hsa-miR-3684	205	1.56723582	Others

hsa-miR-3685	77	1.019236805	Others
hsa-miR-3686	1	0.208666	Others
hsa-miR-3688-3p	216	2.147493227	Others
hsa-miR-3688-5p	2	0.5716835	Others
hsa-miR-3689a-3	1	1.13601	Others
hsa-miR-3689a-5	1	0.000743822	Others
hsa-miR-3689b-3	1	0.000743822	Others
hsa-miR-3689b-5	1	0.000743822	Others
hsa-miR-3689c	1	0.000743822	Others
hsa-miR-3689e	1	0.00148764	Others
hsa-miR-3689f	2	0.182555375	Others
hsa-miR-369-3p	330	87.27747981	Others
hsa-miR-369-5p	324	68.46908798	Others
hsa-miR-3690	66	30.65259158	Others
hsa-miR-3691-3p	85	1.510079906	Others
hsa-miR-3691-5p	261	4.016019632	Others
hsa-miR-3692-3p	21	0.4277535781	Others
hsa-miR-3692-5p	7	0.2619531629	Others
hsa-miR-370-3p	326	152.134047	Others
hsa-miR-370-5p	257	10.20087548	Others
hsa-miR-3714	2	0.754528	Others
hsa-miR-371a-3p	28	603.4715203	Others
hsa-miR-371a-5p	68	3142.861364	Others
hsa-miR-371b-3p	21	5.629834571	Others
hsa-miR-371b-5p	69	16.86097542	Others
hsa-miR-372-3p	47	2454.207068	Others
hsa-miR-372-5p	13	28.009732	Others
hsa-miR-373-3p	100	934.9699979	Others
hsa-miR-373-5p	18	27.44076544	Others
hsa-miR-374a-3p	385	173.8187425	Others
hsa-miR-374a-5p	390	77.0869589	Others

hsa-miR-374b-3p	362	14.53721077	Others
hsa-miR-374b-5p	394	64.96644726	Others
hsa-miR-374c-3p	206	0.04126080809	Others
hsa-miR-374c-5p	174	0.0139619128	Others
hsa-miR-375	219	976.2933309	Others
hsa-miR-376a-2-	218	4.649600506	Others
hsa-miR-376a-3p	312	53.6851615	Others
hsa-miR-376a-5p	301	87.19746063	Others
hsa-miR-376b-3p	309	37.32570046	Others
hsa-miR-376b-5p	133	0.8151510487	Others
hsa-miR-376c-3p	332	144.2529402	Others
hsa-miR-376c-5p	133	1.015277898	Others
hsa-miR-377-3p	290	29.88878967	Others
hsa-miR-377-5p	309	72.59762352	Others
hsa-miR-378a-3p	399	3402.40901	Others
hsa-miR-378a-5p	333	19.7648879	Others
hsa-miR-378b	369	0.123658952	Others
hsa-miR-378c	398	2.847999156	Others
hsa-miR-378d	394	2.419389571	Others
hsa-miR-378e	296	0.03952447893	Others
hsa-miR-378f	394	0.4670751271	Others
hsa-miR-378g	375	0.2150070827	Others
hsa-miR-378h	195	0.01856025327	Others
hsa-miR-378i	398	0.5976769787	Others
hsa-miR-378j	150	0.007560106174	Others
hsa-miR-379-3p	324	40.43663622	Others
hsa-miR-379-5p	321	58.15157226	Others
hsa-miR-380-3p	317	36.2477442	Others
hsa-miR-380-5p	134	2.453504037	Others
hsa-miR-381-3p	375	1528.68202	Others
hsa-miR-381-5p	211	5.635079365	Others

hsa-miR-382-3p	295	25.30467148	Others
hsa-miR-382-5p	332	89.19919092	Others
hsa-miR-383-3p	12	1.75233625	Others
hsa-miR-383-5p	60	12.7621567	Others
hsa-miR-384	8	0.401598	Others
hsa-miR-3907	22	1.756259682	Others
hsa-miR-3908	11	5.730428455	Others
hsa-miR-3909	393	31.03565394	Others
hsa-miR-3910	15	0.9827662	Others
hsa-miR-3911	67	0.9217009104	Others
hsa-miR-3912-3p	329	6.248749793	Others
hsa-miR-3912-5p	40	0.98251825	Others
hsa-miR-3913-3p	114	0.9459867281	Others
hsa-miR-3913-5p	296	3.614709382	Others
hsa-miR-3914	15	1.7304798	Others
hsa-miR-3915	4	0.45489625	Others
hsa-miR-3916	135	1.5174732	Others
hsa-miR-3917	100	1.63861072	Others
hsa-miR-3917-5p	11	4.283075364	Others
hsa-miR-3918	119	1.389390706	Others
hsa-miR-3918-3p	77	1.74768513	Others
hsa-miR-3919	37	4.38196473	Others
hsa-miR-3920	16	0.3930684375	Others
hsa-miR-3921	20	0.79820075	Others
hsa-miR-3922-3p	213	2.86480923	Others
hsa-miR-3922-5p	13	1.214760231	Others
hsa-miR-3923	8	0.96996875	Others
hsa-miR-3924	5	0.5792498	Others
hsa-miR-3924-5p	1	0.9267	Others
hsa-miR-3925-3p	40	1.4860044	Others
hsa-miR-3925-5p	33	1.563703364	Others

hsa-miR-3926	30	0.5972615333	Others
hsa-miR-3926-1-	15	0.3899293693	Others
hsa-miR-3927-3p	13	2.053228538	Others
hsa-miR-3927-5p	1	0.80223	Others
hsa-miR-3928-3p	352	6.191703898	Others
hsa-miR-3928-5p	58	0.9416747586	Others
hsa-miR-3929	133	23.38299498	Others
hsa-miR-3934-3p	35	1.264185886	Others
hsa-miR-3934-5p	244	2.982473041	Others
hsa-miR-3935	93	11.84346973	Others
hsa-miR-3936	20	1.43016555	Others
hsa-miR-3937	10	14.3951413	Others
hsa-miR-3938	118	6.469621788	Others
hsa-miR-3939	192	2.940908257	Others
hsa-miR-3940-3p	339	5.956613027	Others
hsa-miR-3940-5p	70	1.236909057	Others
hsa-miR-3941	71	1.309429592	Others
hsa-miR-3942-3p	122	1.313797148	Others
hsa-miR-3942-5p	248	2.481885859	Others
hsa-miR-3943	56	6.489881179	Others
hsa-miR-3944-3p	156	2.166033694	Others
hsa-miR-3944-5p	83	1.007151036	Others
hsa-miR-3945	6	0.4972398892	Others
hsa-miR-3945-3p	21	2.825441381	Others
hsa-miR-3960	6	0.2788568333	Others
hsa-miR-3960-5p	2	1.655625	Others
hsa-miR-3978	1	0.654692	Others
hsa-miR-409-3p	380	2432.080773	Others
hsa-miR-409-5p	353	591.2117869	Others
hsa-miR-410-3p	390	866.5497006	Others
hsa-miR-410-5p	132	2.645325091	Others

hsa-miR-411-3p	323	60.50982326	Others
hsa-miR-411-5p	379	1672.353392	Others
hsa-miR-412-3p	123	2.826046252	Others
hsa-miR-412-5p	258	20.18310529	Others
hsa-miR-421	399	279.7224108	Others
hsa-miR-422a	241	0.03995714896	Others
hsa-miR-423-3p	399	3967.665213	Others
hsa-miR-423-5p	399	2294.11146	Others
hsa-miR-424-3p	389	162.4278903	Others
hsa-miR-424-5p	390	402.4306872	Others
hsa-miR-425-3p	394	39.1541602	Others
hsa-miR-425-5p	399	487.3116153	Others
hsa-miR-4254	51	1.463598157	Others
hsa-miR-4256	1	0.355789	Others
hsa-miR-4258	1	0.19328	Others
hsa-miR-4259	2	0.0003094375	Others
hsa-miR-4262	1	1.00608	Others
hsa-miR-4263	1	0.528524	Others
hsa-miR-4264	1	1.45672	Others
hsa-miR-4265	3	0.1052336083	Others
hsa-miR-4266	1	0.763836	Others
hsa-miR-4269	2	0.159493	Others
hsa-miR-4280	1	1.70152	Others
hsa-miR-4282	1	0.763836	Others
hsa-miR-4284	9	0.8539171944	Others
hsa-miR-4286	99	1.468858277	Others
hsa-miR-4287	1	9.05E-07	Others
hsa-miR-4288	1	1.31877	Others
hsa-miR-4289	46	0.007034936503	Others
hsa-miR-429	241	330.911523	Others
hsa-miR-4290	5	0.5472516	Others

hsa-miR-4291	1	0.529784	Others
hsa-miR-4295	1	0.181962	Others
hsa-miR-4297	1	2.25014	Others
hsa-miR-4300	1	5.11336	Others
hsa-miR-4301	1	0.355789	Others
hsa-miR-4306	5	0.1014546881	Others
hsa-miR-431-3p	325	73.92111478	Others
hsa-miR-431-5p	337	275.8898421	Others
hsa-miR-4310	1	0.306442	Others
hsa-miR-4310-5p	7	1.014683571	Others
hsa-miR-4312	2	0.1871025	Others
hsa-miR-4313	1	2.19172	Others
hsa-miR-4315	4	0.528658	Others
hsa-miR-4317	1	0.528524	Others
hsa-miR-4318	1	0.209001	Others
hsa-miR-432-3p	190	3.25756754	Others
hsa-miR-432-5p	321	177.0500566	Others
hsa-miR-4321-5p	4	2.018132	Others
hsa-miR-4322	1	0.392872	Others
hsa-miR-4323	10	0.2761901423	Others
hsa-miR-4324	137	0.1438556831	Others
hsa-miR-4325	6	1.36E-06	Others
hsa-miR-4326	339	32.16021308	Others
hsa-miR-4328	2	0.0008318419	Others
hsa-miR-433-3p	302	26.7173306	Others
hsa-miR-433-5p	217	4.472641912	Others
hsa-miR-4417	3	1.123049667	Others
hsa-miR-4419a-5	1	0.209557	Others
hsa-miR-4419b	6	0.8651761667	Others
hsa-miR-4420	13	5.443229769	Others
hsa-miR-4421	140	5.318054	Others

hsa-miR-4422	62	1.140247177	Others
hsa-miR-4423-3p	140	2.3119509	Others
hsa-miR-4423-5p	142	2.536916042	Others
hsa-miR-4424	134	2.383839851	Others
hsa-miR-4425	25	3.36142872	Others
hsa-miR-4426	41	1.211764902	retro-miR
hsa-miR-4427	7	0.8774964286	Others
hsa-miR-4428	4	0.6233075	Others
hsa-miR-4429	305	0.1230023008	Others
hsa-miR-4430	2	0.602044	Others
hsa-miR-4431	12	7.284779358	Others
hsa-miR-4432	5	0.5549108584	Others
hsa-miR-4433a-3	11	0.9190772727	Others
hsa-miR-4433a-5	20	3.1123678	Others
hsa-miR-4433b-3	12	2.538481167	Others
hsa-miR-4433b-5	44	15.33618957	Others
hsa-miR-4434	14	1.3891785	Others
hsa-miR-4435	317	7.153847823	Others
hsa-miR-4436a-5	8	0.04486932616	Others
hsa-miR-4436b-3	24	1.381611792	Others
hsa-miR-4436b-5	18	2.2003185	Others
hsa-miR-4437	4	0.5446625	Others
hsa-miR-4438	3	0.2164496667	Others
hsa-miR-4438-5p	4	0.35148975	Others
hsa-miR-4439	16	0.8589685	Others
hsa-miR-4440	69	1.898146474	Others
hsa-miR-4441	1	0.108276	Others
hsa-miR-4442	22	2.495117186	Others
hsa-miR-4443	170	2.693817331	Others
hsa-miR-4444	80	1.932859063	retro-miR
hsa-miR-4444-1-	19	1.429692816	Others

hsa-miR-4445-3p	2	0.651022	Others
hsa-miR-4445-5p	6	0.5966388333	Others
hsa-miR-4446-3p	72	9.858388222	Others
hsa-miR-4446-5p	37	1.158123784	Others
hsa-miR-4447	6	5.50E-05	Others
hsa-miR-4448	4	0.2887123613	Others
hsa-miR-4449	171	6.263878708	Others
hsa-miR-4450-3p	1	13.2084	Others
hsa-miR-4451	1	0.0793474	Others
hsa-miR-4452-5p	30	1.2670545	Others
hsa-miR-4453	36	0.5888463056	Others
hsa-miR-4454	33	7.671105239	Others
hsa-miR-4455	13	0.173085672	Others
hsa-miR-4457	11	0.4952870909	Others
hsa-miR-4458	28	1.744355798	Others
hsa-miR-4459	5	1.003543584	Others
hsa-miR-4461	327	0.2579736366	Others
hsa-miR-4461-5p	175	0.5590602888	Others
hsa-miR-4463	10	0.5935980299	Others
hsa-miR-4464	7	12.95655743	Others
hsa-miR-4465	11	0.5210944545	Others
hsa-miR-4466	14	1.035724286	Others
hsa-miR-4467	81	1.743859358	Others
hsa-miR-4467-3p	58	3.163507362	Others
hsa-miR-4468	2	1.077271	retro-miR
hsa-miR-4469	20	0.9117886	Others
hsa-miR-4469-5p	8	1.10362575	Others
hsa-miR-4470	16	0.6341033125	Others
hsa-miR-4471	2	0.7649865	Others
hsa-miR-4472	5	0.5351874	Others
hsa-miR-4472-2-	1	0.984929	Others

hsa-miR-4473	218	2.001455991	Others
hsa-miR-4474-3p	61	0.9480609672	Others
hsa-miR-4474-5p	7	0.5515217143	Others
hsa-miR-4475	30	0.8685977311	Others
hsa-miR-4476	4	0.84179025	Others
hsa-miR-4477b	131	1.404536076	Others
hsa-miR-4478	42	1.251032167	Others
hsa-miR-4479	111	1.225130946	Others
hsa-miR-448	55	19.92471596	Others
hsa-miR-4480	2	0.8799925	Others
hsa-miR-4482-3p	123	3.660993423	Others
hsa-miR-4482-5p	17	0.9475787647	Others
hsa-miR-4483	15	0.009977477593	Others
hsa-miR-4484	282	0.2663348589	Others
hsa-miR-4485-3p	383	1.36880238	Others
hsa-miR-4485-5p	310	0.7505221053	Others
hsa-miR-4487	18	0.8285983389	Others
hsa-miR-4487-5p	18	0.94595945	Others
hsa-miR-4488	1	0.150642	Others
hsa-miR-4489	25	0.69559032	Others
hsa-miR-4489-3p	63	1.383102619	Others
hsa-miR-4490	2	0.67031	Others
hsa-miR-4491	6	4.521328667	Others
hsa-miR-4492	8	5.775828375	Others
hsa-miR-4493	8	1.2599875	Others
hsa-miR-4494	13	1.276380154	Others
hsa-miR-4495	2	0.203714	Others
hsa-miR-4495-3p	3	0.273508	Others
hsa-miR-4496	8	1.19784825	Others
hsa-miR-4497	5	0.8263956	Others
hsa-miR-4498	33	1.084584515	Others

hsa-miR-4499	1	0.240645	Others
hsa-miR-449a	259	30.50425765	Others
hsa-miR-449b-3p	28	1.593190679	Others
hsa-miR-449b-5p	229	15.72356344	Others
hsa-miR-449c-3p	18	1.188808889	Others
hsa-miR-449c-5p	254	35.51549236	Others
hsa-miR-4500	18	0.0446238897	Others
hsa-miR-4501	47	1.28097634	Others
hsa-miR-4502	9	1.983378444	Others
hsa-miR-4503	6	1.123985	Others
hsa-miR-4504	86	1.07782936	Others
hsa-miR-4505	21	1.471004286	Others
hsa-miR-4506	7	1.145892143	Others
hsa-miR-4507	26	0.6202810283	Others
hsa-miR-4508	2	0.4772915	Others
hsa-miR-4509	3	0.8039883333	Others
hsa-miR-450a-1-	65	0.9365308689	Others
hsa-miR-450a-2-	182	1.744914638	Others
hsa-miR-450a-5p	383	124.7119388	Others
hsa-miR-450b-3p	26	0.5509428144	Others
hsa-miR-450b-5p	381	230.6249318	Others
hsa-miR-4510	391	0.1015279951	Others
hsa-miR-4511	209	1.816049627	Others
hsa-miR-4512	137	1.537853788	Others
hsa-miR-4513	11	0.8949122727	Others
hsa-miR-4514	1	0.763836	Others
hsa-miR-4515	75	1.54240852	Others
hsa-miR-4516	2	1.677845	Others
hsa-miR-4517	32	1.518025844	Others
hsa-miR-4518	22	14.65837317	Others
hsa-miR-4519	49	1.92923949	Others

hsa-miR-451a	231	212.9270808	Others
hsa-miR-451b	6	0.1824100754	Others
hsa-miR-452-3p	296	26.28168601	Others
hsa-miR-452-5p	338	151.0517695	Others
hsa-miR-4520-2-	97	0.6323675765	Others
hsa-miR-4520-3p	116	3.122577352	Others
hsa-miR-4520-5p	16	1.975017688	Others
hsa-miR-4521	388	20.5079966	Others
hsa-miR-4522	43	0.9578970465	Others
hsa-miR-4523	163	2.07185311	Others
hsa-miR-4524a-3	99	2.101289202	Others
hsa-miR-4524a-5	145	3.047822221	Others
hsa-miR-4524b-3	2	0.973133	Others
hsa-miR-4524b-5	3	1.667353333	Others
hsa-miR-4525	28	0.8745911071	Others
hsa-miR-4525-3p	104	1.274460541	Others
hsa-miR-4526	68	1.048903544	Others
hsa-miR-4527	1	0.350325	Others
hsa-miR-4528	3	0.3710293567	Others
hsa-miR-4529-3p	81	1.75969137	Others
hsa-miR-4529-5p	2	0.1987005	Others
hsa-miR-4530	4	0.41154575	Others
hsa-miR-4531	3	0.5250417233	Others
hsa-miR-4532	1	2.2504	Others
hsa-miR-4533	4	0.413319	Others
hsa-miR-4534-5p	12	0.02913097307	Others
hsa-miR-4536-3p	55	1.244683891	Others
hsa-miR-4536-5p	22	0.9762780909	Others
hsa-miR-4537	2	0.494435	Others
hsa-miR-4537-3p	4	0.76807425	Others
hsa-miR-4538	3	0.411694	Others

hsa-miR-4538-3p	2	0.3554245	Others
hsa-miR-4539	6	0.5671024667	Others
hsa-miR-454-3p	397	243.7832088	Others
hsa-miR-454-5p	387	33.74912288	Others
hsa-miR-455-3p	381	142.7107786	Others
hsa-miR-455-5p	383	115.300434	Others
hsa-miR-4632-3p	38	1.287034211	Others
hsa-miR-4632-5p	1	1.24233	Others
hsa-miR-4633-3p	8	0.27755075	Others
hsa-miR-4633-5p	12	0.9646343333	Others
hsa-miR-4634	4	2.480723	Others
hsa-miR-4635	65	0.9574480462	Others
hsa-miR-4636	200	3.40785973	Others
hsa-miR-4637	18	0.7987698889	Others
hsa-miR-4638-3p	202	2.008438079	Others
hsa-miR-4638-5p	43	0.5493529535	Others
hsa-miR-4639-3p	27	0.5433010741	Others
hsa-miR-4639-5p	42	0.6124904286	Others
hsa-miR-4640-3p	154	2.861243786	Others
hsa-miR-4640-5p	57	1.557629263	Others
hsa-miR-4641	31	0.4323270323	Others
hsa-miR-4642	88	1.341796545	Others
hsa-miR-4643	9	0.3478201111	Others
hsa-miR-4643-5p	1	0.946671	Others
hsa-miR-4644	21	0.7415872381	Others
hsa-miR-4645-3p	263	5.175177768	Others
hsa-miR-4645-5p	20	0.54351525	Others
hsa-miR-4646-3p	91	0.9799666593	Others
hsa-miR-4646-5p	17	1.079143941	Others
hsa-miR-4647	99	1.035109242	Others
hsa-miR-4648	87	1.114338379	Others

hsa-miR-4649-3p	98	1.572753092	Others
hsa-miR-4649-5p	30	1.282716867	Others
hsa-miR-4650-3p	11	0.6842177273	Others
hsa-miR-4651	54	1.205612611	Others
hsa-miR-4652-3p	20	0.5785128	Others
hsa-miR-4652-5p	34	2.482533853	Others
hsa-miR-4653-3p	9	0.6712373333	Others
hsa-miR-4653-5p	53	1.393112038	Others
hsa-miR-4654	51	3.197692137	Others
hsa-miR-4655-3p	3	0.788799	Others
hsa-miR-4655-5p	25	0.7250236	Others
hsa-miR-4656	6	0.4152583333	Others
hsa-miR-4657	42	0.8144523095	Others
hsa-miR-4658	27	0.3098165755	Others
hsa-miR-4659a-3	97	0.8409669711	Others
hsa-miR-4659a-5	22	1.015927364	Others
hsa-miR-4659b-3	58	0.04670625678	Others
hsa-miR-4659b-5	11	0.2452460698	Others
hsa-miR-466	8	0.750637375	Others
hsa-miR-466-5p	74	0.9113846622	Others
hsa-miR-4660	62	1.063041484	Others
hsa-miR-4661-3p	104	1.648981183	Others
hsa-miR-4661-5p	266	4.797623199	Others
hsa-miR-4662a-3	66	0.758888832	Others
hsa-miR-4662a-5	326	11.66533219	Others
hsa-miR-4662b	66	0.3757335577	Others
hsa-miR-4663	5	0.3316906	Others
hsa-miR-4664-3p	105	4.7908852	Others
hsa-miR-4664-5p	27	1.360569333	Others
hsa-miR-4665-3p	13	1.277477	Others
hsa-miR-4665-5p	79	0.999773038	Others

hsa-miR-4666a-3	14	0.4910097857	Others
hsa-miR-4666a-5	74	1.076682405	Others
hsa-miR-4666b	1	1.26094	Others
hsa-miR-4667-3p	34	0.7985812941	Others
hsa-miR-4667-5p	80	1.135158519	Others
hsa-miR-4668-3p	23	1.089727087	Others
hsa-miR-4668-5p	103	1.232579806	Others
hsa-miR-4669	56	0.7559675536	Others
hsa-miR-4670-3p	76	0.6445580658	Others
hsa-miR-4670-5p	31	0.7128296774	Others
hsa-miR-4671-3p	85	2.869220965	Others
hsa-miR-4671-5p	41	1.670923293	Others
hsa-miR-4672	90	1.032114967	Others
hsa-miR-4673	30	1.1416275	Others
hsa-miR-4674	33	0.6579341515	Others
hsa-miR-4674-5p	4	4.52580225	Others
hsa-miR-4675	23	0.5104743478	Others
hsa-miR-4676-3p	132	1.20518353	Others
hsa-miR-4676-5p	24	0.9246504583	Others
hsa-miR-4677-3p	389	28.71170357	Others
hsa-miR-4677-5p	192	1.51481213	Others
hsa-miR-4679	18	0.5471797778	Others
hsa-miR-4679-1-	6	0.3783974	Others
hsa-miR-4679-2-	6	0.3783974	Others
hsa-miR-4680-3p	38	1.354153921	Others
hsa-miR-4680-5p	24	1.30595775	Others
hsa-miR-4681	4	0.34058875	Others
hsa-miR-4682	91	1.353049978	Others
hsa-miR-4683	46	1.003090457	Others
hsa-miR-4684-3p	28	0.8394697143	Others
hsa-miR-4684-5p	10	0.9251175	Others

hsa-miR-4685-3p	233	2.446810764	Others
hsa-miR-4685-5p	7	0.6721191429	Others
hsa-miR-4686	7	0.9959191429	Others
hsa-miR-4687-3p	109	1.272246312	Others
hsa-miR-4687-5p	216	2.17668081	Others
hsa-miR-4688	121	1.773997669	Others
hsa-miR-4689	87	1.062250299	Others
hsa-miR-4690-3p	125	1.449848416	Others
hsa-miR-4690-5p	65	0.8894065692	Others
hsa-miR-4691-3p	31	0.6848644839	Others
hsa-miR-4691-5p	5	0.7249668	Others
hsa-miR-4693-5p	2	0.3502635	Others
hsa-miR-4694-3p	21	0.9806804762	Others
hsa-miR-4695-3p	126	0.2310238337	Others
hsa-miR-4695-5p	13	1.016242538	Others
hsa-miR-4696	1	0.996989	Others
hsa-miR-4697-3p	61	4.77659918	Others
hsa-miR-4697-5p	4	0.32882625	Others
hsa-miR-4698	33	1.999269818	Others
hsa-miR-4699-3p	36	0.9138611111	Others
hsa-miR-4699-5p	54	1.120308259	Others
hsa-miR-4700-3p	27	0.8574158889	Others
hsa-miR-4700-5p	28	0.4692835357	Others
hsa-miR-4701-3p	9	0.3596386667	Others
hsa-miR-4701-5p	25	0.69100304	Others
hsa-miR-4703-3p	30	0.6085022276	Others
hsa-miR-4704-3p	13	0.6281153077	Others
hsa-miR-4704-5p	6	1.048826833	Others
hsa-miR-4705	20	1.8855778	Others
hsa-miR-4706	45	1.422905889	Others
hsa-miR-4707-3p	172	2.55464814	Others

hsa-miR-4707-5p	58	1.151177759	Others
hsa-miR-4708-3p	37	1.070314514	Others
hsa-miR-4708-5p	13	0.5046746923	Others
hsa-miR-4709-3p	60	0.83669925	Others
hsa-miR-4709-5p	94	1.233840032	Others
hsa-miR-4710	15	1.261061933	Others
hsa-miR-4711-3p	3	0.290131	Others
hsa-miR-4711-5p	18	0.6124218889	Others
hsa-miR-4712-3p	94	1.021539596	Others
hsa-miR-4712-5p	8	0.88963475	Others
hsa-miR-4713-3p	5	0.6008308	Others
hsa-miR-4713-5p	40	6.95681035	Others
hsa-miR-4714-3p	116	1.424060009	Others
hsa-miR-4714-5p	53	0.7803641698	Others
hsa-miR-4715-3p	12	0.8041994167	Others
hsa-miR-4715-5p	16	0.8184778313	Others
hsa-miR-4716-3p	7	0.4272888571	Others
hsa-miR-4716-5p	18	0.5825317778	Others
hsa-miR-4717-3p	157	1.303489522	Others
hsa-miR-4717-5p	6	0.8624993333	Others
hsa-miR-4718	5	0.4501262	Others
hsa-miR-4720-3p	5	0.1853066	Others
hsa-miR-4720-5p	64	1.114624391	Others
hsa-miR-4721	13	0.4461602308	Others
hsa-miR-4722-3p	5	1.2849664	Others
hsa-miR-4722-5p	81	1.027960845	Others
hsa-miR-4723-3p	46	0.8287427391	Others
hsa-miR-4723-5p	51	0.6963082549	Others
hsa-miR-4724-3p	4	0.45025475	Others
hsa-miR-4724-5p	63	1.183452397	Others
hsa-miR-4725-3p	123	1.489857813	Others

hsa-miR-4725-5p	94	1.824217128	Others
hsa-miR-4726-3p	9	1.066730556	Others
hsa-miR-4726-5p	115	1.089290159	Others
hsa-miR-4727-3p	39	1.254557974	Others
hsa-miR-4727-5p	16	0.7247614375	Others
hsa-miR-4728-3p	186	2.733016795	Others
hsa-miR-4728-5p	32	1.052481313	Others
hsa-miR-4729	23	1.607661565	Others
hsa-miR-4730	27	0.7027021481	Others
hsa-miR-4731-3p	115	1.394610817	Others
hsa-miR-4731-5p	88	1.353549773	Others
hsa-miR-4732-3p	10	3.6400388	Others
hsa-miR-4732-5p	3	1.558781	Others
hsa-miR-4733-3p	25	1.11682	Others
hsa-miR-4733-5p	52	0.8347599288	Others
hsa-miR-4734	26	0.7674672308	Others
hsa-miR-4735-3p	4	1.177403	Others
hsa-miR-4735-5p	12	1.406105167	Others
hsa-miR-4736	13	2.340900615	Others
hsa-miR-4737	39	1.795720721	Others
hsa-miR-4738-3p	108	1.117282806	Others
hsa-miR-4738-5p	3	0.263053	Others
hsa-miR-4739	15	0.4822870667	Others
hsa-miR-4739-3p	5	0.6634092	Others
hsa-miR-4740-3p	5	1.0948158	Others
hsa-miR-4740-5p	22	0.5850329545	Others
hsa-miR-4741	198	3.056040263	Others
hsa-miR-4742-3p	225	2.039914191	Others
hsa-miR-4742-5p	164	1.423608213	Others
hsa-miR-4743-3p	25	0.94217088	Others
hsa-miR-4743-5p	55	1.139546036	Others

hsa-miR-4744	9	0.8605125556	Others
hsa-miR-4745-3p	134	2.004919619	Others
hsa-miR-4745-5p	234	5.776414544	Others
hsa-miR-4746-3p	9	1.027521244	Others
hsa-miR-4746-5p	336	11.14177462	Others
hsa-miR-4747-3p	74	1.04134277	Others
hsa-miR-4747-5p	46	0.9523310435	Others
hsa-miR-4748	61	1.213817951	Others
hsa-miR-4749-3p	140	1.765339036	Others
hsa-miR-4749-5p	41	1.460900756	Others
hsa-miR-4750-3p	38	1.5249195	Others
hsa-miR-4750-5p	81	0.8119688148	Others
hsa-miR-4751	43	0.704947	Others
hsa-miR-4752	7	0.77731	Others
hsa-miR-4753-3p	57	0.9698001579	Others
hsa-miR-4753-5p	143	1.349589937	Others
hsa-miR-4754	25	2.95815468	Others
hsa-miR-4754-3p	24	3.537592667	Others
hsa-miR-4755-3p	159	1.404811679	Others
hsa-miR-4755-5p	91	1.002583835	Others
hsa-miR-4756-3p	12	0.37550525	Others
hsa-miR-4756-5p	13	0.3670746154	Others
hsa-miR-4757-3p	128	0.9575376875	Others
hsa-miR-4757-5p	66	0.9576987879	Others
hsa-miR-4758-3p	115	6.102259043	Others
hsa-miR-4758-5p	16	0.3366945	Others
hsa-miR-4760-3p	18	0.3188204444	Others
hsa-miR-4760-5p	34	2.893616853	Others
hsa-miR-4761-3p	152	1.73500539	Others
hsa-miR-4761-5p	37	1.315686973	Others
hsa-miR-4762-3p	30	1.258714333	Others

hsa-miR-4762-5p	254	1.790989106	Others
hsa-miR-4763-3p	31	0.7668338387	Others
hsa-miR-4763-5p	58	1.014683793	Others
hsa-miR-4764-3p	5	0.4170076	Others
hsa-miR-4764-5p	13	0.7782179906	Others
hsa-miR-4765	17	0.8852387059	Others
hsa-miR-4765-5p	14	0.9593071429	Others
hsa-miR-4766-3p	152	1.387597849	Others
hsa-miR-4766-5p	35	1.037644029	Others
hsa-miR-4767	267	3.041312543	Others
hsa-miR-4768-3p	9	0.4670341111	Others
hsa-miR-4768-5p	131	2.535888466	Others
hsa-miR-4769-3p	46	2.064938848	Others
hsa-miR-4769-5p	25	1.48925332	Others
hsa-miR-4770	71	0.2602610042	Others
hsa-miR-4771	1	0.14069	Others
hsa-miR-4771-1-	96	0.6225832677	Others
hsa-miR-4771-2-	96	0.6227150385	Others
hsa-miR-4772-3p	30	10.22387797	Others
hsa-miR-4772-5p	42	31.61867607	Others
hsa-miR-4773	34	0.9755693824	Others
hsa-miR-4773-1-	121	1.148356654	Others
hsa-miR-4773-2-	121	1.08410448	Others
hsa-miR-4774-3p	4	1.164379	Others
hsa-miR-4774-5p	14	0.8903917143	Others
hsa-miR-4775	373	19.76445313	Others
hsa-miR-4776-3p	3	0.349516	Others
hsa-miR-4776-5p	10	4.8728227	Others
hsa-miR-4777-3p	20	1.3737852	Others
hsa-miR-4777-5p	6	0.834189	Others
hsa-miR-4778-3p	15	0.3354146667	Others

hsa-miR-4778-5p	14	0.2960734286	Others
hsa-miR-4779	7	0.4417848571	Others
hsa-miR-4779-5p	41	0.6075909756	Others
hsa-miR-4780	3	11.24391667	Others
hsa-miR-4781-3p	226	2.378962487	Others
hsa-miR-4781-5p	18	0.5431053889	Others
hsa-miR-4782-3p	5	0.8658222	Others
hsa-miR-4782-5p	12	0.38655925	Others
hsa-miR-4783-3p	49	0.8715404898	Others
hsa-miR-4783-5p	1	0.216552	Others
hsa-miR-4784	22	0.9064211818	Others
hsa-miR-4785	251	2.417025432	Others
hsa-miR-4786-3p	35	0.9305124	Others
hsa-miR-4786-5p	194	1.918157052	Others
hsa-miR-4787-3p	225	6.601025458	Others
hsa-miR-4787-5p	13	1.073653	Others
hsa-miR-4788	70	1.6979504	retro-miR
hsa-miR-4789-3p	9	0.5794727778	Others
hsa-miR-4789-5p	1	0.356786	Others
hsa-miR-4790-3p	2	0.703792	Others
hsa-miR-4790-5p	4	0.612334	Others
hsa-miR-4791	104	2.509214165	Others
hsa-miR-4792	2	4.140975	Others
hsa-miR-4793-3p	36	1.807923778	Others
hsa-miR-4793-5p	1	1.25578	Others
hsa-miR-4794	22	1.15025	Others
hsa-miR-4794-3p	15	1.023788533	Others
hsa-miR-4795-3p	50	1.11720688	Others
hsa-miR-4795-5p	8	0.7940205	Others
hsa-miR-4796-3p	82	1.246360305	Others
hsa-miR-4796-5p	33	0.7735599091	Others

hsa-miR-4797-3p	218	2.320428789	Others
hsa-miR-4797-5p	51	1.110854765	Others
hsa-miR-4798-3p	1	0.203419	Others
hsa-miR-4798-5p	24	0.4310162917	Others
hsa-miR-4799-3p	3	0.904833	Others
hsa-miR-4799-5p	67	1.334412746	Others
hsa-miR-4800-3p	50	0.77400984	Others
hsa-miR-4800-5p	12	0.5258713333	Others
hsa-miR-4801	4	0.97232775	Others
hsa-miR-4802-3p	63	2.28787327	Others
hsa-miR-4802-5p	50	1.19052424	Others
hsa-miR-4803	63	1.450340873	Others
hsa-miR-4804-3p	88	0.8933735909	Others
hsa-miR-4804-5p	199	2.19849006	Others
hsa-miR-483-3p	221	162.3326542	Others
hsa-miR-483-5p	219	93.40271281	Others
hsa-miR-484	399	1053.990036	Others
hsa-miR-485-3p	319	83.91070036	Others
hsa-miR-485-5p	323	94.62021982	Others
hsa-miR-486-3p	305	21.11276793	Others
hsa-miR-486-5p	399	4442.470275	Others
hsa-miR-487a-3p	287	38.28952305	Others
hsa-miR-487a-5p	198	4.799274803	Others
hsa-miR-487b-3p	345	265.0403671	Others
hsa-miR-487b-5p	176	3.565147693	Others
hsa-miR-488-3p	80	7.383641038	Others
hsa-miR-488-5p	57	7.715636421	Others
hsa-miR-489-3p	109	196.5229398	Others
hsa-miR-489-5p	29	1.302946172	Others
hsa-miR-490-3p	187	70.34159168	Others
hsa-miR-490-5p	74	13.42676055	Others

hsa-miR-491-3p	214	2.100827366	Others
hsa-miR-491-5p	303	3.236268927	Others
hsa-miR-492	10	1.0508913	retro-miR
hsa-miR-493-3p	326	216.3081924	Others
hsa-miR-493-5p	328	194.0287088	Others
hsa-miR-494-3p	315	136.8470218	Others
hsa-miR-494-5p	178	2.781522989	Others
hsa-miR-495-3p	322	77.81452416	Others
hsa-miR-495-5p	108	1.80354588	Others
hsa-miR-496	296	21.8224022	Others
hsa-miR-497-3p	93	1.149467753	Others
hsa-miR-497-5p	376	93.44758245	Others
hsa-miR-498	23	60.33932174	Others
hsa-miR-4999-3p	27	0.7801326296	Others
hsa-miR-4999-5p	142	1.357186225	Others
hsa-miR-499a-3p	37	1.840533946	Others
hsa-miR-499a-5p	242	16.24460305	Others
hsa-miR-499b-3p	12	0.8692346667	Others
hsa-miR-499b-5p	8	0.508651875	Others
hsa-miR-5000-3p	268	3.247936642	Others
hsa-miR-5000-5p	43	0.9300578837	Others
hsa-miR-5001-3p	303	3.935293001	Others
hsa-miR-5001-5p	127	1.955032787	Others
hsa-miR-5002-3p	11	1.174107909	Others
hsa-miR-5002-5p	144	4.318268382	Others
hsa-miR-5003-3p	39	0.9054516154	Others
hsa-miR-5003-5p	10	0.3824712	Others
hsa-miR-5004-3p	24	0.5309484708	Others
hsa-miR-5004-5p	18	0.7196540722	Others
hsa-miR-5006-3p	129	1.515455256	Others
hsa-miR-5006-5p	13	0.4221226154	Others

hsa-miR-5007-3p	1	6.50542	Others
hsa-miR-5007-5p	1	6.50542	Others
hsa-miR-5008-3p	93	2.77156028	Others
hsa-miR-5008-5p	16	2.115443	Others
hsa-miR-5009-3p	7	0.6661415714	Others
hsa-miR-5009-5p	103	1.146541641	Others
hsa-miR-500a-3p	399	826.7732504	Others
hsa-miR-500a-5p	373	5.261249011	Others
hsa-miR-500b-3p	336	5.540308323	Others
hsa-miR-500b-5p	373	5.545305099	Others
hsa-miR-501-3p	399	438.9482622	Others
hsa-miR-501-5p	370	12.59900481	Others
hsa-miR-5010-3p	310	4.451204719	Others
hsa-miR-5010-5p	138	1.36597908	Others
hsa-miR-5011-3p	8	6.23893825	Others
hsa-miR-5011-5p	8	4.779299875	Others
hsa-miR-502-3p	399	34.22428753	Others
hsa-miR-502-5p	330	5.37387478	Others
hsa-miR-503-3p	279	5.936073717	Others
hsa-miR-503-5p	374	40.86919095	Others
hsa-miR-504-3p	45	1.140683956	Others
hsa-miR-504-5p	266	33.72166982	Others
hsa-miR-5047	3	5.93003	Others
hsa-miR-505-3p	396	85.15025432	Others
hsa-miR-505-5p	337	4.899711243	Others
hsa-miR-506-3p	53	62.44328092	Others
hsa-miR-506-5p	7	1.745476714	Others
hsa-miR-507	12	7.445805667	Others
hsa-miR-508-3p	89	47.04303946	Others
hsa-miR-508-5p	21	30.33261071	Others
hsa-miR-5087	25	1.43585188	Others

hsa-miR-5088-3p	16	0.7227343125	Others
hsa-miR-5088-5p	55	0.7789396364	Others
hsa-miR-5089-3p	11	0.6742474545	Others
hsa-miR-5089-5p	7	0.437755	Others
hsa-miR-509-3-5	24	16.84690996	Others
hsa-miR-509-3p	130	84.39256709	Others
hsa-miR-509-5p	23	20.25308565	Others
hsa-miR-5090	58	0.8061041414	Others
hsa-miR-5091	180	5.170495811	Others
hsa-miR-5092	100	1.51008311	Others
hsa-miR-5093	6	0.2539196667	Others
hsa-miR-5094	249	4.731525008	Others
hsa-miR-510-3p	5	1.6483402	Others
hsa-miR-510-5p	6	12.20387333	Others
hsa-miR-5100	23	0.6139257084	Others
hsa-miR-511-3p	53	72.90236843	Others
hsa-miR-511-5p	68	87.6724991	Others
hsa-miR-512-3p	100	465.5486709	Others
hsa-miR-512-5p	16	72.37233731	Others
hsa-miR-513a-3p	10	1.72550833	Others
hsa-miR-513a-5p	46	10.32105283	Others
hsa-miR-513b-3p	3	1.522537	Others
hsa-miR-513b-5p	47	2.993438143	Others
hsa-miR-513c-3p	10	2.6262095	Others
hsa-miR-513c-5p	36	13.71595047	Others
hsa-miR-514a-3p	69	53.55812501	Others
hsa-miR-514a-5p	17	17.04016083	Others
hsa-miR-514b-3p	13	3.135848237	Others
hsa-miR-514b-5p	8	8.61042125	Others
hsa-miR-515-3p	18	62.30680367	Others
hsa-miR-515-5p	80	143.6381989	Others

hsa-miR-516a-3p	33	1.175162027	Others
hsa-miR-516a-5p	115	607.1401304	Others
hsa-miR-516b-3p	31	1.43798759	Others
hsa-miR-516b-5p	82	181.1078953	Others
hsa-miR-517-5p	43	26.7983995	Others
hsa-miR-517a-3p	49	100.9841705	Others
hsa-miR-517b-3p	49	100.7204501	Others
hsa-miR-517c-3p	48	27.3247243	Others
hsa-miR-5187-3p	67	1.18192697	Others
hsa-miR-5187-5p	56	1.167168161	Others
hsa-miR-5188	62	0.8745955	Others
hsa-miR-5189-3p	73	1.230527534	Others
hsa-miR-5189-5p	92	1.044902685	Others
hsa-miR-518a-3p	37	141.6556032	Others
hsa-miR-518a-5p	54	71.86840332	Others
hsa-miR-518b	54	227.8614189	Others
hsa-miR-518c-3p	47	69.66336317	Others
hsa-miR-518c-5p	43	4.260721241	Others
hsa-miR-518d-3p	7	16.85999851	Others
hsa-miR-518d-5p	51	1.714216728	Others
hsa-miR-518e-3p	28	128.0889652	Others
hsa-miR-518e-5p	51	4.70180392	Others
hsa-miR-518f-3p	36	108.0893783	Others
hsa-miR-518f-5p	28	11.47414036	Others
hsa-miR-5190	20	0.34293605	Others
hsa-miR-5191	31	1.178091226	Others
hsa-miR-5192	1	0.19328	Others
hsa-miR-5192-5p	1	0.240645	Others
hsa-miR-5193	58	1.241471603	Others
hsa-miR-5194	12	0.7233998333	Others
hsa-miR-5195-3p	1	0.529784	Others

hsa-miR-5195-5p	2	0.3033375	Others
hsa-miR-5196-3p	13	9.693189923	Others
hsa-miR-5196-5p	2	0.2741035	Others
hsa-miR-5197-5p	3	0.8401136667	Others
hsa-miR-519a-3p	53	76.39641195	Others
hsa-miR-519a-5p	49	30.47384334	Others
hsa-miR-519b-3p	21	32.32746313	Others
hsa-miR-519b-5p	51	3.650009243	Others
hsa-miR-519c-3p	56	95.29706327	Others
hsa-miR-519c-5p	51	3.204082953	Others
hsa-miR-519d-3p	41	45.9391101	Others
hsa-miR-519d-5p	27	18.28238581	Others
hsa-miR-519e-3p	59	1.346879453	Others
hsa-miR-519e-5p	8	22.31090325	Others
hsa-miR-520a-3p	59	267.217843	Others
hsa-miR-520a-5p	33	78.06834533	Others
hsa-miR-520b	58	17.51159345	Others
hsa-miR-520c-3p	58	16.99367966	Others
hsa-miR-520c-5p	51	1.438447864	Others
hsa-miR-520d-3p	30	62.67452134	Others
hsa-miR-520d-5p	50	29.6946112	Others
hsa-miR-520e	32	11.14482743	Others
hsa-miR-520f-3p	55	48.20771808	Others
hsa-miR-520f-5p	40	0.2711950968	Others
hsa-miR-520g-3p	46	52.21830785	Others
hsa-miR-520g-5p	15	8.399412632	Others
hsa-miR-520h	31	2.953298795	Others
hsa-miR-521	14	61.78078694	Others
hsa-miR-522-3p	76	34.74603138	Others
hsa-miR-522-5p	51	4.330165697	Others
hsa-miR-523-3p	19	150.5292286	Others

hsa-miR-523-5p	51	3.672819048	Others
hsa-miR-524-3p	18	70.38335622	Others
hsa-miR-524-5p	23	24.4272261	Others
hsa-miR-525-3p	12	47.11981955	Others
hsa-miR-525-5p	37	95.01702721	Others
hsa-miR-526a	51	3.032560928	Others
hsa-miR-526a-1-	17	9.885510134	Others
hsa-miR-526b-3p	37	1.520380557	Others
hsa-miR-526b-5p	44	48.6555079	Others
hsa-miR-527	53	43.72197616	Others
hsa-miR-532-3p	399	49.92595243	Others
hsa-miR-532-5p	399	666.5792539	Others
hsa-miR-539-3p	311	97.4889684	Others
hsa-miR-539-5p	283	17.162019	Others
hsa-miR-541-3p	192	6.60305025	Others
hsa-miR-541-5p	238	6.497512584	Others
hsa-miR-542-3p	376	56.84862241	Others
hsa-miR-542-5p	361	12.0066448	Others
hsa-miR-543	317	53.73955734	Others
hsa-miR-544a	75	1.698909	Others
hsa-miR-544a-5p	69	5.26175158	Others
hsa-miR-544b	89	2.932160292	Others
hsa-miR-545-3p	136	1.555746765	Others
hsa-miR-545-5p	249	2.411431791	Others
hsa-miR-548a-3p	113	1.70685323	Others
hsa-miR-548a-5p	108	1.660391694	Others
hsa-miR-548aa	2	0.787388	Others
hsa-miR-548ab	137	0.6253626604	Others
hsa-miR-548ac	69	1.112897893	Others
hsa-miR-548ad-3	6	0.2666298623	Others
hsa-miR-548ad-5	45	0.6594988256	Others

hsa-miR-548ae-3	17	0.07873065504	Others
hsa-miR-548ae-5	282	0.5722058963	Others
hsa-miR-548ag	50	2.99487437	Others
hsa-miR-548ag-2	54	4.497806241	Others
hsa-miR-548ah-3	264	2.652727467	Others
hsa-miR-548ah-5	63	6.472612786	Others
hsa-miR-548ai	126	0.7102943376	Others
hsa-miR-548aj-3	19	0.1074614637	Others
hsa-miR-548aj-5	18	0.2114988615	Others
hsa-miR-548ak	89	0.5971953135	Others
hsa-miR-548al	274	2.39167692	Others
hsa-miR-548am-	193	0.3500178069	Others
hsa-miR-548am-	30	1.159596134	Others
hsa-miR-548an	18	0.5328078309	Others
hsa-miR-548an-3	3	1.443195667	Others
hsa-miR-548ao-3	148	1.658158822	Others
hsa-miR-548ao-5	8	1.214114859	Others
hsa-miR-548ap-5	66	0.2332925723	Others
hsa-miR-548aq-3	91	0.01592110243	Others
hsa-miR-548aq-5	87	1.491246384	Others
hsa-miR-548ar-3	131	1.621520374	Others
hsa-miR-548ar-5	26	0.3404051906	Others
hsa-miR-548as-3	15	0.7515211333	Others
hsa-miR-548as-5	116	1.00465535	Others
hsa-miR-548at-3	23	0.785089899	Others
hsa-miR-548at-5	155	1.472179006	Others
hsa-miR-548au-3	8	1.0461655	Others
hsa-miR-548au-5	229	2.726017647	Others
hsa-miR-548av-3	67	0.3512326748	Others
hsa-miR-548av-5	175	0.01068860689	Others
hsa-miR-548aw	102	0.967537902	Others

hsa-miR-548aw-3	60	0.8641132833	Others
hsa-miR-548ax	59	1.510179224	Others
hsa-miR-548ay-3	253	2.047729348	Others
hsa-miR-548ay-5	74	0.8240398145	Others
hsa-miR-548az-3	27	0.5309820741	Others
hsa-miR-548az-5	129	3.002459504	Others
hsa-miR-548b-3p	108	1.762193009	Others
hsa-miR-548b-5p	95	2.721795726	Others
hsa-miR-548ba	83	1.972115952	Others
hsa-miR-548bb-3	4	0.3001044103	Others
hsa-miR-548bb-5	1	0.210381	Others
hsa-miR-548c-3p	1	0.763836	Others
hsa-miR-548c-5p	6	4.845994833	Others
hsa-miR-548d-3p	145	1.427978194	Others
hsa-miR-548d-5p	97	0.8926428436	Others
hsa-miR-548e-3p	382	43.04143913	Others
hsa-miR-548e-5p	366	10.00982844	Others
hsa-miR-548f-3p	224	40.43452048	Others
hsa-miR-548f-5p	18	0.5905553501	Others
hsa-miR-548g-3p	113	0.07491754126	Others
hsa-miR-548g-5p	3	0.4057483333	Others
hsa-miR-548h-3p	3	1.112553333	Others
hsa-miR-548h-5p	353	11.55375225	Others
hsa-miR-548i	308	0.8561137101	Others
hsa-miR-548j-3p	147	2.198373027	Others
hsa-miR-548j-5p	214	2.366727483	Others
hsa-miR-548k	395	70.35074248	Others
hsa-miR-548l	217	1.938909562	Others
hsa-miR-548m	11	0.124288255	Others
hsa-miR-548n	279	2.830124608	Others
hsa-miR-548o-3p	383	22.16310935	Others

hsa-miR-548o-5p	8	1.702277875	Others
hsa-miR-548p	268	2.768707096	Others
hsa-miR-548q	106	0.9299389481	Others
hsa-miR-548s	324	3.849953464	Others
hsa-miR-548t-3p	2	0.863171	Others
hsa-miR-548t-5p	38	0.4152152693	Others
hsa-miR-548u	200	2.19417542	Others
hsa-miR-548v	69	2.111648826	Others
hsa-miR-548w	294	4.638374973	Others
hsa-miR-548x-2-	33	0.8283829424	Others
hsa-miR-548x-3p	17	1.631971	Others
hsa-miR-548x-5p	3	0.5912616667	Others
hsa-miR-548y	54	2.266498151	Others
hsa-miR-548y-3p	98	1.830633928	Others
hsa-miR-548z	3	0.9638253333	Others
hsa-miR-549a	167	6.580966216	Others
hsa-miR-549a-5p	241	18.9728598	Others
hsa-miR-550a-3-	377	0.3680542838	Others
hsa-miR-550a-3p	382	15.88476943	Others
hsa-miR-550a-5p	377	13.66018616	Others
hsa-miR-550b-2-	82	0.0386011199	Others
hsa-miR-550b-3p	85	0.05982718305	Others
hsa-miR-551a	120	4.8256812	Others
hsa-miR-551b-3p	151	8.628199119	Others
hsa-miR-551b-5p	54	1.849961981	Others
hsa-miR-552-3p	15	2.594183667	Others
hsa-miR-552-5p	13	1.833655538	Others
hsa-miR-553	1	0.250886	Others
hsa-miR-555	2	0.936168	Others
hsa-miR-556-3p	109	2.847187229	Others
hsa-miR-556-5p	141	5.013814014	Others

hsa-miR-557	1	1.52567	Others
hsa-miR-5571-3p	7	0.6531234286	Others
hsa-miR-5571-5p	1	0.752658	Others
hsa-miR-5572	1	0.000515509	Others
hsa-miR-5579-3p	107	2.199027561	Others
hsa-miR-5579-5p	84	1.581532131	Others
hsa-miR-558	2	1.491515	Others
hsa-miR-5580-3p	31	0.778739129	Others
hsa-miR-5580-5p	33	0.4751071818	Others
hsa-miR-5581-3p	263	2.074908441	Others
hsa-miR-5581-5p	6	1.115051667	Others
hsa-miR-5582-3p	151	1.340683974	Others
hsa-miR-5582-5p	7	3.344051429	Others
hsa-miR-5583-3p	58	1.37089331	Others
hsa-miR-5583-5p	24	1.633744375	Others
hsa-miR-5584-3p	4	1.59346525	Others
hsa-miR-5584-5p	13	0.68289	Others
hsa-miR-5585-3p	1	0.14069	Others
hsa-miR-5585-5p	40	1.25903205	Others
hsa-miR-5586-3p	15	0.7134098667	Others
hsa-miR-5586-5p	40	1.748897575	Others
hsa-miR-5587-3p	173	1.657570121	Others
hsa-miR-5587-5p	86	1.359164012	Others
hsa-miR-5588-3p	46	1.319387826	Others
hsa-miR-5588-5p	140	1.6082063	Others
hsa-miR-5589-3p	5	9.5746118	Others
hsa-miR-5589-5p	4	2.18048	Others
hsa-miR-559	4	0.61123325	Others
hsa-miR-559-3p	39	1.483122821	Others
hsa-miR-5590-3p	3	1.532485	Others
hsa-miR-5590-5p	4	1.936674	Others

hsa-miR-5591-3p	8	0.5812635	Others
hsa-miR-5591-5p	2	0.6346395	Others
hsa-miR-561-3p	157	2.385895465	Others
hsa-miR-561-5p	347	90.70397476	Others
hsa-miR-563-5p	1	0.232656	Others
hsa-miR-564	3	1.240739333	Others
hsa-miR-564-3p	30	0.9829816333	Others
hsa-miR-567	2	0.8468475	Others
hsa-miR-5680	130	2.459832046	Others
hsa-miR-5681a-5	1	0.209006	Others
hsa-miR-5682	10	1.1436576	Others
hsa-miR-5682-5p	22	1.662321727	Others
hsa-miR-5683	131	3.032249595	Others
hsa-miR-5684	48	0.7444787708	Others
hsa-miR-5685	9	0.7695624444	Others
hsa-miR-5687	6	0.47223	Others
hsa-miR-5687-5p	32	0.6079116875	Others
hsa-miR-5688	15	1.3062076	Others
hsa-miR-5689	25	0.660948	Others
hsa-miR-5689-3p	25	0.58555084	Others
hsa-miR-5690	299	6.380699225	Others
hsa-miR-5691	40	3.105157275	Others
hsa-miR-5692a	2	0.211426	Others
hsa-miR-5692b	12	0.02907880983	Others
hsa-miR-5692c	14	0.4358952143	Others
hsa-miR-5692c-2	5	0.2574764	Others
hsa-miR-5693	14	0.2599520462	Others
hsa-miR-5694	5	0.1969622	Others
hsa-miR-5694-3p	3	0.2433086667	Others
hsa-miR-5695	139	1.892594827	Others
hsa-miR-5696	147	1.241964531	Others

hsa-miR-5696-3p	211	2.142941237	Others
hsa-miR-5697	67	0.8857517463	Others
hsa-miR-5698	39	0.6385018205	Others
hsa-miR-5699-3p	250	3.493901812	Others
hsa-miR-5699-5p	301	4.256652827	Others
hsa-miR-570-3p	171	1.484070801	Others
hsa-miR-570-5p	124	0.7247689887	Others
hsa-miR-5700	24	1.161829333	Others
hsa-miR-5701	396	0.4108240339	Others
hsa-miR-5702	11	2.023371545	Others
hsa-miR-5703	15	0.6977982667	Others
hsa-miR-5704-3p	3	1.005021333	Others
hsa-miR-5705	16	0.331557875	Others
hsa-miR-5706	136	1.351892463	Others
hsa-miR-5707	4	0.18886225	Others
hsa-miR-5708	10	0.317792	Others
hsa-miR-5708-3p	1	0.965028	Others
hsa-miR-571	3	0.6469233333	Others
hsa-miR-571-5p	3	3.740306333	Others
hsa-miR-572	20	1.2939693	retro-miR
hsa-miR-572-5p	6	2.596655167	Others
hsa-miR-573	206	32.10639757	Others
hsa-miR-5739	2	0.1942145	Others
hsa-miR-574-3p	398	546.8329514	Others
hsa-miR-574-5p	348	17.39053	Others
hsa-miR-576-3p	327	5.235578563	Others
hsa-miR-576-5p	388	34.91666173	Others
hsa-miR-577	210	36.22969178	Others
hsa-miR-578	24	0.5636692083	Others
hsa-miR-578-5p	80	2.390843825	Others
hsa-miR-579-3p	127	1.158583037	Others

hsa-miR-579-5p	228	1.659279349	Others
hsa-miR-580-3p	274	3.257363518	Others
hsa-miR-580-5p	37	1.238873189	Others
hsa-miR-581	56	0.8309283929	Others
hsa-miR-582-3p	364	75.51603595	Others
hsa-miR-582-5p	346	46.25925401	Others
hsa-miR-583	1	2.06739	Others
hsa-miR-583-3p	18	1.055670152	Others
hsa-miR-584-3p	122	2.178712355	Others
hsa-miR-584-5p	382	155.0222128	Others
hsa-miR-585-3p	174	6.838303374	Others
hsa-miR-585-5p	119	3.353350269	Others
hsa-miR-586	69	0.9881528551	Others
hsa-miR-587	3	3.211489333	Others
hsa-miR-588	53	1.335034321	Others
hsa-miR-589-3p	388	34.08967983	Others
hsa-miR-589-5p	399	279.3181358	Others
hsa-miR-590-3p	377	48.73547629	Others
hsa-miR-590-5p	308	3.89613011	Others
hsa-miR-592	99	6.715159384	Others
hsa-miR-595	1	0.00282086	Others
hsa-miR-596	2	1.126903	Others
hsa-miR-597-3p	183	1.349217355	Others
hsa-miR-597-5p	134	1.855842627	Others
hsa-miR-598-3p	366	24.97776801	Others
hsa-miR-598-5p	21	1.045647048	Others
hsa-miR-599	12	0.6304654715	Others
hsa-miR-599-5p	29	0.7936171379	Others
hsa-miR-600	45	1.514248156	Others
hsa-miR-600-5p	65	1.730824738	Others
hsa-miR-602	47	0.8568929491	Others

hsa-miR-603	3	0.229216	Others
hsa-miR-605-3p	201	5.505928328	Others
hsa-miR-605-5p	169	2.137721627	Others
hsa-miR-606	6	0.5938643333	Others
hsa-miR-607	37	1.044322703	Others
hsa-miR-607-5p	101	1.290693574	Others
hsa-miR-6071	1	0.150642	Others
hsa-miR-6073	1	0.144069	Others
hsa-miR-6073-5p	3	0.5705063333	Others
hsa-miR-6078	1	1.97258	Others
hsa-miR-6080	3	1.609733333	Others
hsa-miR-6081	1	0.240645	Others
hsa-miR-6084	4	0.733203	Others
hsa-miR-6086	1	0.168138	Others
hsa-miR-6087	1	2.8264	Others
hsa-miR-6089	9	1.156661889	Others
hsa-miR-609	8	1.027718375	Others
hsa-miR-6090	2	0.7623785	Others
hsa-miR-610	89	1.338151056	Others
hsa-miR-611	35	1.054793543	Others
hsa-miR-612	21	1.669386381	Others
hsa-miR-612-3p	21	2.82456349	Others
hsa-miR-6125	131	1.770904511	Others
hsa-miR-6128	21	0.001202178072	Others
hsa-miR-6129	118	0.03206413098	Others
hsa-miR-613	1	0.63118	Others
hsa-miR-6130	298	0.002438327888	Others
hsa-miR-6131	40	0.05275916525	Others
hsa-miR-6132	31	1.161493387	Others
hsa-miR-6132-3p	167	2.453960826	Others
hsa-miR-6133	7	0.02342297437	Others

hsa-miR-6134	88	0.002052281866	Others
hsa-miR-614-5p	9	0.264707	Others
hsa-miR-615-3p	309	331.8445301	Others
hsa-miR-615-5p	180	7.762015361	Others
hsa-miR-616-3p	151	1.337215291	Others
hsa-miR-616-5p	372	10.02716059	Others
hsa-miR-617	3	1.083242333	Others
hsa-miR-618	268	12.18824605	Others
hsa-miR-619-3p	5	1.1199856	Others
hsa-miR-619-5p	1	3.12408	Others
hsa-miR-622	6	2.873285333	retro-miR
hsa-miR-623	1	0.410595	Others
hsa-miR-624-3p	191	1.582593749	Others
hsa-miR-624-5p	311	3.892916215	Others
hsa-miR-625-3p	392	46.98344861	Others
hsa-miR-625-5p	386	32.05640873	Others
hsa-miR-627-3p	294	3.441126082	Others
hsa-miR-627-5p	306	3.926648725	Others
hsa-miR-628-3p	356	8.656561652	Others
hsa-miR-628-5p	365	14.07683494	Others
hsa-miR-629-3p	340	5.279917815	Others
hsa-miR-629-5p	394	50.44277909	Others
hsa-miR-630	3	4.091016667	Others
hsa-miR-631	1	0.168537	Others
hsa-miR-631-3p	1	15.3401	Others
hsa-miR-632	14	2.199904929	Others
hsa-miR-633	6	0.6888491667	Others
hsa-miR-635	2	0.4731405	Others
hsa-miR-636	259	3.369001876	Others
hsa-miR-637	2	0.409581	Others
hsa-miR-638	5	0.02903805298	Others

hsa-miR-639	26	2.423087846	Others
hsa-miR-641	392	20.79058964	Others
hsa-miR-642a-3p	133	3.594555397	Others
hsa-miR-642a-5p	142	4.296155158	Others
hsa-miR-642b-3p	131	0.2716757789	Others
hsa-miR-642b-5p	142	0.03659293695	Others
hsa-miR-643	243	2.491687556	Others
hsa-miR-644a	1	3.3021	Others
hsa-miR-6499-5p	35	7.952848857	Others
hsa-miR-650	2	0.7143065	Others
hsa-miR-650-3p	11	5.534803818	Others
hsa-miR-6500-3p	139	5.988320549	Others
hsa-miR-6500-5p	11	0.6430802727	Others
hsa-miR-6501-3p	54	0.7804914815	Others
hsa-miR-6501-5p	117	2.319520171	Others
hsa-miR-6502-3p	3	0.7289703333	Others
hsa-miR-6502-5p	74	2.584223095	Others
hsa-miR-6503-3p	48	45.95104565	Others
hsa-miR-6503-5p	39	26.66147262	Others
hsa-miR-6504-5p	27	0.7476414074	Others
hsa-miR-6505-3p	71	1.044531187	Others
hsa-miR-6505-5p	122	1.171867847	Others
hsa-miR-6506-3p	1	0.132119	Others
hsa-miR-6506-5p	11	0.5846309091	Others
hsa-miR-6507-3p	4	0.62389575	Others
hsa-miR-6507-5p	18	1.703598333	Others
hsa-miR-6508-3p	57	0.6590161228	Others
hsa-miR-6508-5p	49	0.5681014286	Others
hsa-miR-6509-3p	69	0.9376398696	Others
hsa-miR-6509-5p	92	1.125725587	Others
hsa-miR-651-3p	5	0.8143718	Others

hsa-miR-651-5p	292	6.005315658	Others
hsa-miR-6510-3p	90	14.64901321	Others
hsa-miR-6510-5p	3	3.026723333	Others
hsa-miR-6511a-3	391	6.834367035	Others
hsa-miR-6511a-5	240	1.894070988	Others
hsa-miR-6511b-3	392	6.971450971	Others
hsa-miR-6511b-5	240	0.9593031408	Others
hsa-miR-6512-3p	3	0.5111066667	Others
hsa-miR-6512-5p	19	1.335087526	Others
hsa-miR-6513-3p	193	2.133483078	Others
hsa-miR-6513-5p	140	1.604408214	Others
hsa-miR-6514-3p	84	1.074792468	Others
hsa-miR-6514-5p	143	1.257037042	Others
hsa-miR-6515-3p	85	1.723061918	Others
hsa-miR-6515-5p	120	1.441634983	Others
hsa-miR-6516-3p	270	6.038130511	Others
hsa-miR-6516-5p	186	2.631139016	Others
hsa-miR-652-3p	398	129.4949538	Others
hsa-miR-652-5p	326	10.20141928	Others
hsa-miR-653-3p	52	32.80675419	Others
hsa-miR-653-5p	146	199.4584049	Others
hsa-miR-654-3p	375	1971.877031	Others
hsa-miR-654-5p	299	42.80139782	Others
hsa-miR-655-3p	276	17.07762765	Others
hsa-miR-655-5p	158	2.369404775	Others
hsa-miR-656-3p	289	35.78706046	Others
hsa-miR-656-5p	115	1.810472843	Others
hsa-miR-657	4	0.1113329256	Others
hsa-miR-657-5p	2	0.2339835	Others
hsa-miR-658	16	0.9936884375	Others
hsa-miR-659-3p	69	1.53552542	Others

hsa-miR-659-5p	346	6.275584483	Others
hsa-miR-660-3p	297	3.630102347	Others
hsa-miR-660-5p	395	351.138974	Others
hsa-miR-661	7	0.4235821429	Others
hsa-miR-661-5p	3	0.505429	Others
hsa-miR-662	4	0.5331125	Others
hsa-miR-664a-3p	384	37.87359378	Others
hsa-miR-664a-5p	310	7.421457497	Others
hsa-miR-664b-3p	304	3.240405073	Others
hsa-miR-664b-5p	195	2.036169082	Others
hsa-miR-665	310	59.09233769	Others
hsa-miR-668-3p	271	11.10753037	Others
hsa-miR-668-5p	7	0.967701	Others
hsa-miR-670-3p	30	4.763128	Others
hsa-miR-670-5p	3	2.017554333	Others
hsa-miR-671-3p	399	178.7705702	Others
hsa-miR-671-5p	397	37.9699661	Others
hsa-miR-6715a-3	48	1.21687184	Others
hsa-miR-6715b-3	9	0.4647553333	Others
hsa-miR-6715b-5	3	0.4645116667	Others
hsa-miR-6716-3p	305	4.430439413	Others
hsa-miR-6716-5p	14	1.536805286	Others
hsa-miR-6717-5p	3	1.152432667	Others
hsa-miR-6718-5p	21	4.391227857	Others
hsa-miR-6719-3p	5	0.043922198	Others
hsa-miR-6719-5p	1	0.649049	Others
hsa-miR-6720-3p	53	1.127315383	Others
hsa-miR-6720-5p	72	1.874750611	Others
hsa-miR-6721-5p	78	1.109150923	Others
hsa-miR-6722-3p	1	0.233608	Others
hsa-miR-6722-5p	2	1.63572	Others

hsa-miR-6723-3p	57	0.7372832045	Others
hsa-miR-6723-5p	311	0.4998204831	Others
hsa-miR-6726-3p	168	1.491964012	Others
hsa-miR-6726-5p	12	0.7099996667	Others
hsa-miR-6727-3p	34	0.9546122059	Others
hsa-miR-6727-5p	47	1.519564255	Others
hsa-miR-6728-3p	8	0.84533025	Others
hsa-miR-6728-5p	42	0.6668281667	Others
hsa-miR-6729-3p	84	1.319946119	Others
hsa-miR-6729-5p	45	1.272233511	Others
hsa-miR-6730-3p	38	1.361841368	Others
hsa-miR-6730-5p	58	1.019261034	Others
hsa-miR-6731-3p	58	3.756400845	Others
hsa-miR-6731-5p	8	0.715684625	Others
hsa-miR-6732-3p	199	2.404332734	Others
hsa-miR-6732-5p	22	0.8300161818	Others
hsa-miR-6733-3p	90	0.9992996278	Others
hsa-miR-6733-5p	146	1.276838185	Others
hsa-miR-6734-3p	55	1.499537964	Others
hsa-miR-6734-5p	152	1.591201831	Others
hsa-miR-6735-3p	102	0.8904905588	Others
hsa-miR-6735-5p	166	1.441244496	Others
hsa-miR-6736-3p	19	0.8123031053	Others
hsa-miR-6736-5p	16	0.52813375	Others
hsa-miR-6737-3p	231	3.43024497	Others
hsa-miR-6737-5p	8	0.959446	Others
hsa-miR-6738-3p	95	1.104447147	Others
hsa-miR-6738-5p	4	1.1452495	Others
hsa-miR-6739-3p	69	0.913240029	Others
hsa-miR-6739-5p	14	1.144313929	Others
hsa-miR-6740-3p	27	0.7947346407	Others

hsa-miR-6740-5p	50	0.59655928	Others
hsa-miR-6741-3p	254	2.470206717	Others
hsa-miR-6741-5p	6	1.017745833	Others
hsa-miR-6742-3p	191	1.722913152	Others
hsa-miR-6742-5p	10	1.2511394	Others
hsa-miR-6743-3p	61	1.456167852	Others
hsa-miR-6743-5p	7	1.777548	Others
hsa-miR-6744-3p	7	0.2554534286	Others
hsa-miR-6744-5p	26	0.9600524231	Others
hsa-miR-6745	2	0.9784225	Others
hsa-miR-6746-3p	135	1.608234239	Others
hsa-miR-6746-5p	36	0.6631587583	Others
hsa-miR-6747-3p	322	3.901954332	Others
hsa-miR-6747-5p	12	1.497456	Others
hsa-miR-6748-3p	39	0.7103583333	Others
hsa-miR-6748-5p	13	1.269186615	Others
hsa-miR-6749-3p	202	1.87092296	Others
hsa-miR-6749-5p	14	0.800578	Others
hsa-miR-675-3p	158	35.25570887	Others
hsa-miR-675-5p	176	22.07296506	Others
hsa-miR-6750-3p	125	1.881436304	Others
hsa-miR-6750-5p	29	1.163747862	Others
hsa-miR-6751-3p	47	1.017897021	Others
hsa-miR-6751-5p	63	0.8050803968	Others
hsa-miR-6752-3p	60	1.15635275	Others
hsa-miR-6752-5p	1	0.877487	Others
hsa-miR-6753-3p	102	1.619819649	Others
hsa-miR-6753-5p	12	0.8829690833	Others
hsa-miR-6754-3p	47	0.9179875745	Others
hsa-miR-6754-5p	23	0.7463055217	Others
hsa-miR-6755-3p	20	0.3612695	Others

hsa-miR-6755-5p	72	0.8341351806	Others
hsa-miR-6756-3p	131	2.002834934	Others
hsa-miR-6756-5p	15	0.7762504667	Others
hsa-miR-6757-3p	65	1.416042446	Others
hsa-miR-6757-5p	53	1.403034321	Others
hsa-miR-6758-3p	78	0.9843076154	Others
hsa-miR-6758-5p	22	0.8299130909	Others
hsa-miR-6759-3p	11	0.7176180909	Others
hsa-miR-6759-5p	18	0.6121528333	Others
hsa-miR-676-3p	181	2.631695199	Others
hsa-miR-676-5p	48	1.188000354	Others
hsa-miR-6760-3p	6	3.856257333	Others
hsa-miR-6761-3p	25	1.41674832	Others
hsa-miR-6761-5p	86	1.564006655	Others
hsa-miR-6762-3p	148	1.741208845	Others
hsa-miR-6762-5p	12	0.7719083333	Others
hsa-miR-6763-3p	78	1.206247205	Others
hsa-miR-6763-5p	37	0.8924620811	Others
hsa-miR-6764-3p	131	1.076352117	Others
hsa-miR-6764-5p	58	1.497554724	Others
hsa-miR-6765-3p	97	2.099261969	Others
hsa-miR-6765-5p	14	0.598212	Others
hsa-miR-6766-3p	95	1.113160337	Others
hsa-miR-6766-5p	7	0.3744447143	Others
hsa-miR-6767-3p	4	0.362904	Others
hsa-miR-6767-5p	35	0.6707715429	Others
hsa-miR-6768-3p	12	0.6447488658	Others
hsa-miR-6768-5p	35	1.066383771	Others
hsa-miR-6769a-3	12	0.9349339167	Others
hsa-miR-6769a-5	12	0.80220075	Others
hsa-miR-6769b-3	275	3.255254855	Others

hsa-miR-6769b-5	9	0.7121738889	Others
hsa-miR-6770-3p	258	2.394134178	Others
hsa-miR-6770-5p	98	1.108968921	Others
hsa-miR-6771-3p	18	1.940850087	Others
hsa-miR-6771-5p	15	1.661506467	Others
hsa-miR-6772-3p	176	1.56353775	Others
hsa-miR-6772-5p	3	0.5098333333	Others
hsa-miR-6773-3p	17	0.4966851765	Others
hsa-miR-6773-5p	54	3.20980763	Others
hsa-miR-6774-3p	33	2.570472818	Others
hsa-miR-6774-5p	22	1.031983364	Others
hsa-miR-6775-3p	252	4.107017948	Others
hsa-miR-6775-5p	5	1.200441	Others
hsa-miR-6776-3p	61	1.114343803	Others
hsa-miR-6776-5p	10	0.7435323	Others
hsa-miR-6777-3p	189	2.878902905	Others
hsa-miR-6777-5p	80	1.039409538	Others
hsa-miR-6778-3p	25	0.4410066532	Others
hsa-miR-6778-5p	3	0.178633	Others
hsa-miR-6779-3p	25	0.92893208	Others
hsa-miR-6779-5p	55	1.154275218	Others
hsa-miR-6780a-3	38	0.5910773421	Others
hsa-miR-6780a-5	78	1.1924875	Others
hsa-miR-6780b-3	71	0.8615620704	Others
hsa-miR-6780b-5	22	0.4516715455	Others
hsa-miR-6781-3p	53	0.7790619623	Others
hsa-miR-6781-5p	26	0.7192356538	Others
hsa-miR-6782-3p	69	0.8125780435	Others
hsa-miR-6782-5p	25	1.24712884	Others
hsa-miR-6783-3p	166	1.846290663	Others
hsa-miR-6783-5p	81	1.133592593	Others

hsa-miR-6784-3p	194	2.436442649	Others
hsa-miR-6784-5p	10	0.4605954	Others
hsa-miR-6785-3p	23	1.086424478	Others
hsa-miR-6785-5p	12	1.61196375	Others
hsa-miR-6786-3p	215	1.663403014	Others
hsa-miR-6786-5p	17	0.8952447647	Others
hsa-miR-6787-3p	129	1.712935674	Others
hsa-miR-6787-5p	26	0.9893592308	Others
hsa-miR-6788-3p	45	1.900689311	Others
hsa-miR-6788-5p	25	0.99063312	Others
hsa-miR-6789-3p	120	1.642944044	Others
hsa-miR-6789-5p	45	1.4817638	Others
hsa-miR-6790-3p	33	1.323741576	Others
hsa-miR-6790-5p	5	1.0197436	Others
hsa-miR-6791-3p	121	1.426954314	Others
hsa-miR-6791-5p	3	0.672603	Others
hsa-miR-6792-3p	4	1.2393125	Others
hsa-miR-6792-5p	8	1.98737175	Others
hsa-miR-6793-3p	157	1.503911389	Others
hsa-miR-6793-5p	57	0.9737851579	Others
hsa-miR-6794-3p	30	1.532346067	Others
hsa-miR-6794-5p	16	1.052246688	Others
hsa-miR-6795-3p	43	2.088462023	Others
hsa-miR-6795-5p	13	0.4245690769	Others
hsa-miR-6796-3p	40	1.8097166	Others
hsa-miR-6796-5p	27	1.587083037	Others
hsa-miR-6797-3p	208	2.489148338	Others
hsa-miR-6797-5p	27	1.857501889	Others
hsa-miR-6798-3p	306	4.061118497	Others
hsa-miR-6798-5p	3	1.148554333	Others
hsa-miR-6799-3p	62	1.054125419	Others

hsa-miR-6799-5p	16	1.500202375	Others
hsa-miR-6800-3p	79	1.065434101	Others
hsa-miR-6800-5p	15	0.9921334	Others
hsa-miR-6801-3p	58	1.218307138	Others
hsa-miR-6801-5p	14	0.8325263571	Others
hsa-miR-6802-3p	172	1.552536448	Others
hsa-miR-6802-5p	27	0.6171114074	Others
hsa-miR-6803-3p	259	3.674809788	Others
hsa-miR-6803-5p	3	0.5656203333	Others
hsa-miR-6804-3p	56	0.8333237321	Others
hsa-miR-6804-5p	55	1.590567782	Others
hsa-miR-6805-3p	130	1.505201569	Others
hsa-miR-6805-5p	51	1.304465451	Others
hsa-miR-6806-3p	145	1.108711924	Others
hsa-miR-6806-5p	6	0.2783481667	Others
hsa-miR-6807-3p	165	1.836783042	Others
hsa-miR-6807-5p	25	0.85551604	Others
hsa-miR-6808-3p	27	0.7853410741	Others
hsa-miR-6809-3p	17	1.431767193	Others
hsa-miR-6809-5p	56	1.08112375	Others
hsa-miR-6810-3p	119	2.084977739	Others
hsa-miR-6810-5p	41	0.8338660732	Others
hsa-miR-6811-3p	6	0.3196811667	Others
hsa-miR-6811-5p	57	4.399002404	Others
hsa-miR-6812-3p	210	1.659303095	Others
hsa-miR-6812-5p	19	0.7151931579	Others
hsa-miR-6813-3p	23	0.913054087	Others
hsa-miR-6813-5p	69	0.9392810145	Others
hsa-miR-6814-3p	42	0.9388433333	Others
hsa-miR-6814-5p	23	0.6982665217	Others
hsa-miR-6815-3p	41	0.609708122	Others

hsa-miR-6815-5p	90	0.9638544778	Others
hsa-miR-6816-3p	77	0.9680011299	Others
hsa-miR-6816-5p	1	1.35807	Others
hsa-miR-6817-3p	117	0.9791003846	Others
hsa-miR-6817-5p	2	0.270847603	Others
hsa-miR-6818-3p	94	1.054699191	Others
hsa-miR-6818-5p	135	1.323201607	Others
hsa-miR-6819-3p	289	3.435859391	Others
hsa-miR-6819-5p	21	1.274026238	Others
hsa-miR-6820-3p	57	1.835826702	Others
hsa-miR-6820-5p	144	1.500943368	Others
hsa-miR-6821-3p	44	0.651216	Others
hsa-miR-6821-5p	23	0.6450862609	Others
hsa-miR-6822-3p	2	0.5157275	Others
hsa-miR-6822-5p	21	0.774460619	Others
hsa-miR-6823-3p	15	1.576535467	Others
hsa-miR-6823-5p	10	2.0365523	Others
hsa-miR-6824-3p	254	1.971117988	Others
hsa-miR-6824-5p	16	0.9587214375	Others
hsa-miR-6825-3p	16	1.474776438	Others
hsa-miR-6825-5p	69	0.8135452899	Others
hsa-miR-6826-3p	19	0.5473038947	Others
hsa-miR-6826-5p	56	0.8735521786	Others
hsa-miR-6827-3p	142	1.379682474	Others
hsa-miR-6827-5p	26	0.585899269	Others
hsa-miR-6828-3p	22	1.046563364	Others
hsa-miR-6828-5p	36	0.9617932778	Others
hsa-miR-6829-3p	16	0.66636025	Others
hsa-miR-6829-5p	30	1.058864633	Others
hsa-miR-6830-3p	6	0.6124305	Others
hsa-miR-6830-5p	8	1.6797475	Others

hsa-miR-6831-3p	10	0.7524629	Others
hsa-miR-6831-5p	8	0.765524125	Others
hsa-miR-6832-3p	38	1.012352816	Others
hsa-miR-6832-5p	43	0.5290930465	Others
hsa-miR-6833-3p	93	0.9315050645	Others
hsa-miR-6833-5p	27	0.8416995185	Others
hsa-miR-6834-3p	25	1.1099898	Others
hsa-miR-6834-5p	6	0.36398	Others
hsa-miR-6835-3p	2	0.621722	Others
hsa-miR-6835-5p	10	1.3947975	Others
hsa-miR-6836-3p	74	1.337949392	Others
hsa-miR-6836-5p	123	1.33638187	Others
hsa-miR-6837-3p	167	1.564236263	Others
hsa-miR-6837-5p	40	0.98840135	Others
hsa-miR-6838-3p	5	0.7035516	Others
hsa-miR-6838-5p	72	0.8112755556	Others
hsa-miR-6839-3p	5	0.397625	Others
hsa-miR-6839-5p	61	0.9317165574	Others
hsa-miR-6840-3p	85	1.905427788	Others
hsa-miR-6840-5p	196	3.153449362	Others
hsa-miR-6841-3p	2	2.5698535	Others
hsa-miR-6841-5p	3	3.658115	Others
hsa-miR-6842-3p	270	12.05044172	Others
hsa-miR-6842-5p	31	0.954443129	Others
hsa-miR-6843-3p	46	1.055935848	Others
hsa-miR-6844	148	1.697639446	Others
hsa-miR-6845-3p	41	1.314577073	Others
hsa-miR-6845-5p	72	1.415026476	Others
hsa-miR-6846-3p	11	1.598987545	Others
hsa-miR-6846-5p	4	0.482073325	Others
hsa-miR-6847-3p	13	0.7128152308	Others

hsa-miR-6847-5p	121	1.051826438	Others
hsa-miR-6848-3p	13	0.884973	Others
hsa-miR-6848-5p	17	0.7998577647	Others
hsa-miR-6849-3p	63	0.8853564762	Others
hsa-miR-6849-5p	7	0.6703037143	Others
hsa-miR-6850-3p	112	1.848382732	Others
hsa-miR-6850-5p	68	0.9859247353	Others
hsa-miR-6851-3p	56	1.000348952	Others
hsa-miR-6851-5p	35	0.9471233429	Others
hsa-miR-6852-3p	60	1.093634383	Others
hsa-miR-6852-5p	336	4.046805756	Others
hsa-miR-6853-3p	166	1.545497289	Others
hsa-miR-6853-5p	7	0.6252202857	Others
hsa-miR-6854-3p	29	0.7579451828	Others
hsa-miR-6854-5p	78	1.129423205	Others
hsa-miR-6855-3p	118	1.474295839	Others
hsa-miR-6855-5p	41	0.6090183659	Others
hsa-miR-6856-3p	20	0.49627135	Others
hsa-miR-6856-5p	7	0.4365061429	Others
hsa-miR-6857-3p	163	1.610120215	Others
hsa-miR-6857-5p	9	0.4008684444	Others
hsa-miR-6858-3p	165	1.763319061	Others
hsa-miR-6858-5p	64	1.089301906	Others
hsa-miR-6859-3p	91	1.590245868	Others
hsa-miR-6859-5p	185	1.787965141	Others
hsa-miR-6860	31	0.902259	Others
hsa-miR-6861-3p	30	0.6801367333	Others
hsa-miR-6861-5p	7	1.011613571	Others
hsa-miR-6862-3p	80	1.331684988	Others
hsa-miR-6862-5p	122	1.246326418	Others
hsa-miR-6864-5p	46	0.5702757826	Others

hsa-miR-6865-3p	26	1.228980038	Others
hsa-miR-6865-5p	60	0.7316244667	Others
hsa-miR-6866-3p	58	0.9919469138	Others
hsa-miR-6866-5p	169	1.376550456	Others
hsa-miR-6867-3p	2	1.0602375	Others
hsa-miR-6867-5p	13	0.4615766923	Others
hsa-miR-6868-3p	154	2.183286584	Others
hsa-miR-6868-5p	2	0.2263115	Others
hsa-miR-6869-3p	9	1.841583111	Others
hsa-miR-6869-5p	68	1.494708235	Others
hsa-miR-6870-3p	61	2.007501984	Others
hsa-miR-6870-5p	7	0.9032052857	Others
hsa-miR-6871-3p	56	0.6146321607	Others
hsa-miR-6871-5p	66	0.9938352879	Others
hsa-miR-6872-3p	20	0.56823795	Others
hsa-miR-6872-5p	12	0.3511851667	Others
hsa-miR-6873-3p	54	0.7950677963	Others
hsa-miR-6873-5p	13	0.5336131538	Others
hsa-miR-6874-3p	25	0.68194996	Others
hsa-miR-6874-5p	1	0.33742	Others
hsa-miR-6875-3p	74	1.099464763	Others
hsa-miR-6875-5p	159	1.63947066	Others
hsa-miR-6876-3p	14	0.8623387857	Others
hsa-miR-6876-5p	28	0.7787158929	Others
hsa-miR-6877-3p	121	1.250399893	Others
hsa-miR-6877-5p	94	1.146010479	Others
hsa-miR-6878-3p	13	0.5315456154	Others
hsa-miR-6878-5p	15	0.4365824	Others
hsa-miR-6879-3p	137	1.626890905	Others
hsa-miR-6879-5p	2	0.269826	Others
hsa-miR-6880-3p	82	1.247083073	Others

hsa-miR-6880-5p	19	0.8647111053	Others
hsa-miR-6881-3p	101	2.239227072	Others
hsa-miR-6881-5p	12	1.496851167	Others
hsa-miR-6882-3p	33	0.6671319394	Others
hsa-miR-6882-5p	114	1.181705456	Others
hsa-miR-6883-3p	46	0.8152090217	Others
hsa-miR-6883-5p	2	1.27E-06	Others
hsa-miR-6884-3p	29	1.610216759	Others
hsa-miR-6884-5p	50	1.22824366	Others
hsa-miR-6885-3p	106	1.62976966	Others
hsa-miR-6885-5p	13	0.4834825385	Others
hsa-miR-6886-3p	82	1.186021244	Others
hsa-miR-6886-5p	209	3.057572344	Others
hsa-miR-6887-3p	38	1.234176237	Others
hsa-miR-6887-5p	11	0.7422627273	Others
hsa-miR-6888-3p	68	1.132815618	Others
hsa-miR-6888-5p	4	0.77229425	Others
hsa-miR-6889-3p	181	3.053309138	Others
hsa-miR-6889-5p	28	2.579627464	Others
hsa-miR-6890-3p	15	0.8957044667	Others
hsa-miR-6890-5p	21	0.6582334286	Others
hsa-miR-6891-3p	18	1.796169611	Others
hsa-miR-6891-5p	39	1.107595641	Others
hsa-miR-6892-3p	27	2.505494889	Others
hsa-miR-6892-5p	177	1.775024621	Others
hsa-miR-6893-3p	20	0.95047065	Others
hsa-miR-6893-5p	8	0.893117375	Others
hsa-miR-6894-3p	245	2.090336461	Others
hsa-miR-6894-5p	67	1.306789333	Others
hsa-miR-6895-3p	132	1.428277538	Others
hsa-miR-6895-5p	61	1.059419607	Others

hsa-miR-7-1-3p	377	22.02607549	Others
hsa-miR-7-2-3p	96	2.936442654	Others
hsa-miR-7-5p	389	98.43787284	Others
hsa-miR-708-3p	329	89.07758433	Others
hsa-miR-708-5p	338	117.8204803	Others
hsa-miR-7106-3p	43	0.7076193488	Others
hsa-miR-7106-5p	24	0.5921009167	Others
hsa-miR-7107-3p	24	0.6172546667	Others
hsa-miR-7107-5p	5	0.5843716	Others
hsa-miR-7108-3p	117	1.433373	Others
hsa-miR-7108-5p	27	2.098394185	Others
hsa-miR-7109-3p	40	1.055124025	Others
hsa-miR-7109-5p	50	1.12434574	Others
hsa-miR-711	2	0.4434145	Others
hsa-miR-711-5p	5	1.0533748	Others
hsa-miR-7110-3p	94	1.353680415	Others
hsa-miR-7110-5p	16	0.5704292113	Others
hsa-miR-7111-3p	131	1.92293086	Others
hsa-miR-7111-5p	61	1.089760213	Others
hsa-miR-7112-3p	49	1.703102306	Others
hsa-miR-7112-5p	5	0.8404918	Others
hsa-miR-7113-3p	15	0.797122	Others
hsa-miR-7113-5p	43	0.6438156047	Others
hsa-miR-7114-3p	230	2.239895878	Others
hsa-miR-7114-5p	30	1.203843167	Others
hsa-miR-7150	1	0.250886	Others
hsa-miR-7151-3p	44	0.6296236364	Others
hsa-miR-7151-5p	40	0.4668288	Others
hsa-miR-7152-3p	10	0.8364441	Others
hsa-miR-7152-5p	4	0.7132125	Others
hsa-miR-7153-5p	2	2.024147	Others

hsa-miR-7154-3p	2	2.70457	Others
hsa-miR-7154-5p	2	1.7672805	Others
hsa-miR-7155-3p	187	2.542877397	Others
hsa-miR-7155-5p	44	1.029244636	Others
hsa-miR-7156-5p	9	0.9883312222	Others
hsa-miR-7158-3p	18	9.962884889	Others
hsa-miR-7158-5p	4	2.38849775	Others
hsa-miR-7159-3p	1	3.32418	Others
hsa-miR-7160-3p	4	0.3738005	Others
hsa-miR-7160-5p	1	1.01153	Others
hsa-miR-7161-3p	6	0.362514	retro-miR
hsa-miR-7161-5p	3	1.111821	retro-miR
hsa-miR-718	6	1.328346833	Others
hsa-miR-744-3p	369	9.925125959	Others
hsa-miR-744-5p	399	303.1509707	Others
hsa-miR-7515	1	0.232342	Others
hsa-miR-758-3p	330	176.5578976	Others
hsa-miR-758-5p	259	8.404609965	Others
hsa-miR-760	304	7.033290467	Others
hsa-miR-761	1	0.273223	Others
hsa-miR-762	14	0.7428785286	Others
hsa-miR-764	1	0.545939	Others
hsa-miR-7641	397	0.459646111	Others
hsa-miR-7641-2-	398	0.4429293413	Others
hsa-miR-765	101	0.9347350891	Others
hsa-miR-766-3p	387	23.54438161	Others
hsa-miR-766-5p	89	1.946812337	Others
hsa-miR-767-3p	49	4.018941612	Others
hsa-miR-767-5p	139	83.16612842	Others
hsa-miR-769-3p	357	6.210243232	Others
hsa-miR-769-5p	399	835.9643386	Others

hsa-miR-770-5p	239	6.685503707	Others
hsa-miR-7702	12	2.73601425	Others
hsa-miR-7703	182	1.863601462	Others
hsa-miR-7704	7	0.8773461429	Others
hsa-miR-7705	244	7.567523661	Others
hsa-miR-7706	398	100.4797793	Others
hsa-miR-7843-3p	3	1.661210667	Others
hsa-miR-7843-5p	1	0.320996	Others
hsa-miR-7844-5p	7	0.6506335714	Others
hsa-miR-7845-5p	122	1.324668803	Others
hsa-miR-7846-3p	56	1.594142054	Others
hsa-miR-7847-3p	1	0.240645	Others
hsa-miR-7848-3p	21	1.884524143	Others
hsa-miR-7849-3p	36	1.345623833	Others
hsa-miR-7850-5p	18	1.183310556	Others
hsa-miR-7851-3p	75	0.8560479067	Others
hsa-miR-7852-3p	20	0.58539505	Others
hsa-miR-7852-5p	13	0.9984540769	Others
hsa-miR-7853-5p	3	1.035296333	Others
hsa-miR-7854-3p	153	1.936698908	Others
hsa-miR-7855-5p	6	0.4404286667	Others
hsa-miR-7973	32	0.8943087188	Others
hsa-miR-7974	370	1576.546845	Others
hsa-miR-7975	43	4.728626674	Others
hsa-miR-7976	366	11.79225927	Others
hsa-miR-7977	334	5.468736009	Others
hsa-miR-7978	4	2.515406	Others
hsa-miR-802	7	0.9173781429	Others
hsa-miR-8052	1	1.82466	Others
hsa-miR-8056-5p	3	2.724561667	Others
hsa-miR-8058	3	1.320528333	Others

hsa-miR-8061	3	0.397602	Others
hsa-miR-8066	3	0.639715	Others
hsa-miR-8069	1	2.7285	Others
hsa-miR-8072	86	1.142304349	Others
hsa-miR-8072-3p	64	1.187446094	Others
hsa-miR-8075	1	0.419344	Others
hsa-miR-8077	13	1.162372385	Others
hsa-miR-8078	3	1.149470333	Others
hsa-miR-8078-5p	8	1.614212625	Others
hsa-miR-8079	2	2.07394	Others
hsa-miR-8080	2	0.500041	Others
hsa-miR-8082	1	0.185529	Others
hsa-miR-8083	1	5.87161	Others
hsa-miR-8085	3	0.211248	Others
hsa-miR-8085-3p	5	0.7522722	Others
hsa-miR-873-3p	231	45.68708827	Others
hsa-miR-873-5p	235	42.73002319	Others
hsa-miR-874-3p	397	88.57133555	Others
hsa-miR-874-5p	316	7.828132997	Others
hsa-miR-875-3p	6	0.6336568333	Others
hsa-miR-875-5p	1	1.23762	Others
hsa-miR-876-3p	48	1.855581917	Others
hsa-miR-876-5p	50	9.3159822	Others
hsa-miR-877-3p	304	3.179262458	Others
hsa-miR-877-5p	398	76.88321492	Others
hsa-miR-885-3p	23	5.310865783	Others
hsa-miR-885-5p	99	25.36954831	Others
hsa-miR-887-3p	345	75.70619117	Others
hsa-miR-887-5p	324	15.08474472	Others
hsa-miR-888-3p	3	1.340146	Others
hsa-miR-888-5p	35	1.4083362	Others

hsa-miR-889-3p	337	248.7586428	Others
hsa-miR-889-5p	204	4.396284299	Others
hsa-miR-890	6	0.9442425	Others
hsa-miR-891a-5p	108	12.03972747	Others
hsa-miR-891b	5	1.6093104	Others
hsa-miR-892a	35	1.277127657	Others
hsa-miR-892b	6	0.2862943333	Others
hsa-miR-892c-3p	9	4.264981247	Others
hsa-miR-892c-5p	1	9.08E-05	Others
hsa-miR-9-3p	183	200.0534581	Others
hsa-miR-9-5p	327	964.3417059	Others
hsa-miR-920	1	0.355789	Others
hsa-miR-921	1	1.61195	Others
hsa-miR-922	6	0.598048	Others
hsa-miR-92a-1-5	377	21.53344221	Others
hsa-miR-92a-2-5	19	5.760494263	Others
hsa-miR-92a-3p	399	51190.19521	Others
hsa-miR-92b-3p	399	12623.44187	Others
hsa-miR-92b-5p	347	13.28578939	Others
hsa-miR-93-3p	399	42.67003246	Others
hsa-miR-93-5p	399	1391.559794	Others
hsa-miR-933	93	1.283393624	Others
hsa-miR-934	60	19.69487182	Others
hsa-miR-935	247	38.97521612	Others
hsa-miR-936	15	2.0435974	Others
hsa-miR-937-3p	354	6.710006347	Others
hsa-miR-937-5p	56	1.497413679	Others
hsa-miR-938-3p	3	0.3074876667	Others
hsa-miR-939-3p	166	1.541618657	Others
hsa-miR-939-5p	245	2.404638143	Others
hsa-miR-940	392	19.89288644	Others

hsa-miR-941	399	511.6436368	Others
hsa-miR-941-3-5	399	102.5751163	Others
hsa-miR-942-3p	145	1.520966338	Others
hsa-miR-942-5p	387	17.59251996	Others
hsa-miR-943	196	1.698481323	Others
hsa-miR-944	151	79.24886017	Others
hsa-miR-95-3p	201	11.09432466	Others
hsa-miR-95-5p	87	1.572642241	Others
hsa-miR-9500	2	0.666976	Others
hsa-miR-96-3p	67	1.644299478	Others
hsa-miR-96-5p	268	158.2460989	Others
hsa-miR-98-3p	368	36.88152275	Others
hsa-miR-98-5p	399	3951.228136	Others
hsa-miR-99a-3p	350	20.56619033	Others
hsa-miR-99a-5p	398	1019.036429	Others
hsa-miR-99b-3p	397	78.14778336	Others
hsa-miR-99b-5p	399	26319.23689	Others
