# Supplementary material for: Retro-miRs: novel and functional miRNAs originating from mRNA retrotransposition
Source: Mob DNA. 2023 Sep 8;14:12. doi: 10.1186/s13100-023-00301-w (PMC10486083; doi:10.1186/s13100-023-00301-w)
Supplement: Supplementary file 8 — Additional file 8: Table S7. Experimental validation of retro-miRs. [file 13100_2023_301_MOESM8_ESM.pdf]

Table S7. Experimental validation of retro-miRs

Retro-miR	Experimental Validation			
	By qRT-PCR (PubMed ID)	By RNA-seq (# of samples)	Hot link (miRBase)	Articles (PubMed ID)
miR-10527-5p	36636641	123	https://www.mirbase.org/cgi-bin/get_read.pl?acc=MIMAT0041997	25822230
miR-1244	27073334	43	https://www.mirbase.org/cgi-bin/get_read.pl?acc=MIMAT0005896	18285502
miR-3654	27297584	20	https://www.mirbase.org/cgi-bin/get_read.pl?acc=MIMAT0018074	20300190
miR-4426	33725572	50	https://www.mirbase.org/cgi-bin/get_read.pl?acc=MIMAT0018941	20300190
miR-4444	-	115	https://www.mirbase.org/cgi-bin/get_read.pl?acc=MIMAT0018962	20459774
miR-4468	33407103	12	https://www.mirbase.org/cgi-bin/get_read.pl?acc=MIMAT0018995	20459774
miR-4788	33447367	22	https://www.mirbase.org/cgi-bin/get_read.pl?acc=MIMAT0019958	20300190
miR-492	36117850; 30779065	14	https://www.mirbase.org/cgi-bin/get_read.pl?acc=MIMAT0002812	23601657
miR-572	34044640	27	https://www.mirbase.org/cgi-bin/get_read.pl?acc=MIMAT0003237	25893382
miR-622	35413218; 35567340; 35872695	56	https://www.mirbase.org/cgi-bin/get_read.pl?acc=MIMAT0003291	27440378
miR-7161-3p	34749766	13	https://www.mirbase.org/cgi-bin/get_read.pl?acc=MIMAT0028233	23034410
miR-7161-5p	-	13	https://www.mirbase.org/cgi-bin/get_read.pl?acc=MIMAT0028232	23034410
