# Supplementary material for: Retro-miRs: novel and functional miRNAs originating from mRNA retrotransposition
Source: Mob DNA. 2023 Sep 8;14:12. doi: 10.1186/s13100-023-00301-w (PMC10486083; doi:10.1186/s13100-023-00301-w)
Supplement: Supplementary file 9 — Additional file 9: Table S8. Targets of the retro-miRs. [file 13100_2023_301_MOESM9_ESM.pdf]

Table S8. Targets of the retro-miRs

miRNA	Target	Filter	Source
miR-10527	ENSG00000000419	8m	TargetScan
miR-10527	ENSG00000001497	8m	TargetScan
miR-10527	ENSG00000002587	8m	TargetScan
miR-10527	ENSG00000002746	8m	TargetScan
miR-10527	ENSG00000004846	8m	TargetScan
miR-10527	ENSG00000004897	8m	TargetScan
miR-10527	ENSG00000005020	8m	TargetScan
miR-10527	ENSG00000005108	8m	TargetScan
miR-10527	ENSG00000005483	8m	TargetScan
miR-10527	ENSG00000005893	8m	TargetScan
miR-10527	ENSG00000006459	8m	TargetScan
miR-10527	ENSG00000006715	8m	TargetScan
miR-10527	ENSG00000006747	8m	TargetScan
miR-10527	ENSG00000007372	8m	TargetScan
miR-10527	ENSG00000008282	8m	TargetScan
miR-10527	ENSG00000008405	8m	TargetScan
miR-10527	ENSG00000009694	8m	TargetScan
miR-10527	ENSG00000009780	8m	TargetScan
miR-10527	ENSG00000010404	8m	TargetScan
miR-10527	ENSG00000010610	8m	TargetScan
miR-10527	ENSG00000010818	8m	TargetScan
miR-10527	ENSG00000011347	8m	TargetScan
miR-10527	ENSG00000011454	8m	TargetScan
miR-10527	ENSG00000011566	8m	TargetScan
miR-10527	ENSG00000012817	8m	TargetScan
miR-10527	ENSG00000012983	8m	TargetScan
miR-10527	ENSG00000013583	8m	TargetScan
miR-10527	ENSG00000015153	8m	TargetScan
miR-10527	ENSG00000017427	8m	TargetScan
miR-10527	ENSG00000018236	8m	TargetScan

miR-10527	ENSG00000018869	8m	TargetScan
miR-10527	ENSG00000019995	8m	TargetScan
miR-10527	ENSG00000020426	8m	TargetScan
miR-10527	ENSG00000021776	8m	TargetScan
miR-10527	ENSG00000023516	8m	TargetScan
miR-10527	ENSG00000025039	8m	TargetScan
miR-10527	ENSG00000026103	8m	TargetScan
miR-10527	ENSG00000029363	8m	TargetScan
miR-10527	ENSG00000036473	8m	TargetScan
miR-10527	ENSG00000037637	8m	TargetScan
miR-10527	ENSG00000038532	8m	TargetScan
miR-10527	ENSG00000040199	8m	TargetScan
miR-10527	ENSG00000041515	8m	TargetScan
miR-10527	ENSG00000042781	8m	TargetScan
miR-10527	ENSG00000043093	8m	TargetScan
miR-10527	ENSG00000046653	8m	TargetScan
miR-10527	ENSG00000046774	8m	TargetScan
miR-10527	ENSG00000046889	8m	TargetScan
miR-10527	ENSG00000047634	8m	TargetScan
miR-10527	ENSG00000047849	8m	TargetScan
miR-10527	ENSG00000050393	8m	TargetScan
miR-10527	ENSG00000050748	8m	TargetScan
miR-10527	ENSG00000051523	8m	TargetScan
miR-10527	ENSG00000053254	8m	TargetScan
miR-10527	ENSG00000053328	8m	TargetScan
miR-10527	ENSG00000053770	8m	TargetScan
miR-10527	ENSG00000054690	8m	TargetScan
miR-10527	ENSG00000055813	8m	TargetScan
miR-10527	ENSG00000055917	8m	TargetScan
miR-10527	ENSG00000056586	8m	TargetScan
miR-10527	ENSG00000057657	8m	TargetScan

miR-10527	ENSG00000057935	8m	TargetScan
miR-10527	ENSG00000058091	8m	TargetScan
miR-10527	ENSG00000058272	8m	TargetScan
miR-10527	ENSG00000058600	8m	TargetScan
miR-10527	ENSG00000058729	8m	TargetScan
miR-10527	ENSG00000058866	8m	TargetScan
miR-10527	ENSG00000059758	8m	TargetScan
miR-10527	ENSG00000061455	8m	TargetScan
miR-10527	ENSG00000061676	8m	TargetScan
miR-10527	ENSG00000061987	8m	TargetScan
miR-10527	ENSG00000062194	8m	TargetScan
miR-10527	ENSG00000063438	8m	TargetScan
miR-10527	ENSG00000064313	8m	TargetScan
miR-10527	ENSG00000065135	8m	TargetScan
miR-10527	ENSG00000065308	8m	TargetScan
miR-10527	ENSG00000065325	8m	TargetScan
miR-10527	ENSG00000065809	8m	TargetScan
miR-10527	ENSG00000065882	8m	TargetScan
miR-10527	ENSG00000065883	8m	TargetScan
miR-10527	ENSG00000065923	8m	TargetScan
miR-10527	ENSG00000066084	8m	TargetScan
miR-10527	ENSG00000066557	8m	TargetScan
miR-10527	ENSG00000067082	8m	TargetScan
miR-10527	ENSG00000067182	8m	TargetScan
miR-10527	ENSG00000067208	8m	TargetScan
miR-10527	ENSG00000067533	8m	TargetScan
miR-10527	ENSG00000068745	8m	TargetScan
miR-10527	ENSG00000069188	8m	TargetScan
miR-10527	ENSG00000069431	8m	TargetScan
miR-10527	ENSG00000070018	8m	TargetScan
miR-10527	ENSG00000070214	8m	TargetScan

miR-10527	ENSG00000070269	8m	TargetScan
miR-10527	ENSG00000070367	8m	TargetScan
miR-10527	ENSG00000070476	8m	TargetScan
miR-10527	ENSG00000070614	8m	TargetScan
miR-10527	ENSG00000070882	8m	TargetScan
miR-10527	ENSG00000071242	8m	TargetScan
miR-10527	ENSG00000071539	8m	TargetScan
miR-10527	ENSG00000072401	8m	TargetScan
miR-10527	ENSG00000072415	8m	TargetScan
miR-10527	ENSG00000072501	8m	TargetScan
miR-10527	ENSG00000072657	8m	TargetScan
miR-10527	ENSG00000072786	8m	TargetScan
miR-10527	ENSG00000073282	8m	TargetScan
miR-10527	ENSG00000073614	8m	TargetScan
miR-10527	ENSG00000073712	8m	TargetScan
miR-10527	ENSG00000074966	8m	TargetScan
miR-10527	ENSG00000075426	8m	TargetScan
miR-10527	ENSG00000076003	8m	TargetScan
miR-10527	ENSG00000076053	8m	TargetScan
miR-10527	ENSG00000076248	8m	TargetScan
miR-10527	ENSG00000076641	8m	TargetScan
miR-10527	ENSG00000077147	8m	TargetScan
miR-10527	ENSG00000077232	8m	TargetScan
miR-10527	ENSG00000077420	8m	TargetScan
miR-10527	ENSG00000077514	8m	TargetScan
miR-10527	ENSG00000078098	8m	TargetScan
miR-10527	ENSG00000078114	8m	TargetScan
miR-10527	ENSG00000078124	8m	TargetScan
miR-10527	ENSG00000078140	8m	TargetScan
miR-10527	ENSG00000078304	8m	TargetScan
miR-10527	ENSG00000078795	8m	TargetScan

miR-10527	ENSG00000078967	8m	TargetScan
miR-10527	ENSG00000079156	8m	TargetScan
miR-10527	ENSG00000080298	8m	TargetScan
miR-10527	ENSG00000080345	8m	TargetScan
miR-10527	ENSG00000080561	8m	TargetScan
miR-10527	ENSG00000080802	8m	TargetScan
miR-10527	ENSG00000081014	8m	TargetScan
miR-10527	ENSG00000081019	8m	TargetScan
miR-10527	ENSG00000081051	8m	TargetScan
miR-10527	ENSG00000081189	8m	TargetScan
miR-10527	ENSG00000081320	8m	TargetScan
miR-10527	ENSG00000081800	8m	TargetScan
miR-10527	ENSG00000082068	8m	TargetScan
miR-10527	ENSG00000082175	8m	TargetScan
miR-10527	ENSG00000082212	8m	TargetScan
miR-10527	ENSG00000082269	8m	TargetScan
miR-10527	ENSG00000082512	8m	TargetScan
miR-10527	ENSG00000083312	8m	TargetScan
miR-10527	ENSG00000083844	8m	TargetScan
miR-10527	ENSG00000083937	8m	TargetScan
miR-10527	ENSG00000084070	8m	TargetScan
miR-10527	ENSG00000084090	8m	TargetScan
miR-10527	ENSG00000084710	8m	TargetScan
miR-10527	ENSG00000084733	8m	TargetScan
miR-10527	ENSG00000085224	8m	TargetScan
miR-10527	ENSG00000085831	8m	TargetScan
miR-10527	ENSG00000088833	8m	TargetScan
miR-10527	ENSG00000088854	8m	TargetScan
miR-10527	ENSG00000089048	8m	TargetScan
miR-10527	ENSG00000089050	8m	TargetScan
miR-10527	ENSG00000089818	8m	TargetScan

miR-10527	ENSG00000091317	8m	TargetScan
miR-10527	ENSG00000091436	8m	TargetScan
miR-10527	ENSG00000091844	8m	TargetScan
miR-10527	ENSG00000092439	8m	TargetScan
miR-10527	ENSG00000092820	8m	TargetScan
miR-10527	ENSG00000092978	8m	TargetScan
miR-10527	ENSG00000093072	8m	TargetScan
miR-10527	ENSG00000095303	8m	TargetScan
miR-10527	ENSG00000095739	8m	TargetScan
miR-10527	ENSG00000095787	8m	TargetScan
miR-10527	ENSG00000096060	8m	TargetScan
miR-10527	ENSG00000096063	8m	TargetScan
miR-10527	ENSG00000096401	8m	TargetScan
miR-10527	ENSG00000099139	8m	TargetScan
miR-10527	ENSG00000099246	8m	TargetScan
miR-10527	ENSG00000099721	8m	TargetScan
miR-10527	ENSG00000099817	8m	TargetScan
miR-10527	ENSG00000099942	8m	TargetScan
miR-10527	ENSG00000100030	8m	TargetScan
miR-10527	ENSG00000100330	8m	TargetScan
miR-10527	ENSG00000100344	8m	TargetScan
miR-10527	ENSG00000100345	8m	TargetScan
miR-10527	ENSG00000100346	8m	TargetScan
miR-10527	ENSG00000100354	8m	TargetScan
miR-10527	ENSG00000100364	8m	TargetScan
miR-10527	ENSG00000100380	8m	TargetScan
miR-10527	ENSG00000100461	8m	TargetScan
miR-10527	ENSG00000100478	8m	TargetScan
miR-10527	ENSG00000100523	8m	TargetScan
miR-10527	ENSG00000100568	8m	TargetScan
miR-10527	ENSG00000100583	8m	TargetScan

miR-10527	ENSG00000100614	8m	TargetScan
miR-10527	ENSG00000100664	8m	TargetScan
miR-10527	ENSG00000100678	8m	TargetScan
miR-10527	ENSG00000100697	8m	TargetScan
miR-10527	ENSG00000100934	8m	TargetScan
miR-10527	ENSG00000100994	8m	TargetScan
miR-10527	ENSG00000101019	8m	TargetScan
miR-10527	ENSG00000101247	8m	TargetScan
miR-10527	ENSG00000101290	8m	TargetScan
miR-10527	ENSG00000101327	8m	TargetScan
miR-10527	ENSG00000101413	8m	TargetScan
miR-10527	ENSG00000101544	8m	TargetScan
miR-10527	ENSG00000101746	8m	TargetScan
miR-10527	ENSG00000101888	8m	TargetScan
miR-10527	ENSG00000101901	8m	TargetScan
miR-10527	ENSG00000101938	8m	TargetScan
miR-10527	ENSG00000101958	8m	TargetScan
miR-10527	ENSG00000102024	8m	TargetScan
miR-10527	ENSG00000102043	8m	TargetScan
miR-10527	ENSG00000102053	8m	TargetScan
miR-10527	ENSG00000102098	8m	TargetScan
miR-10527	ENSG00000102181	8m	TargetScan
miR-10527	ENSG00000102471	8m	TargetScan
miR-10527	ENSG00000102743	8m	TargetScan
miR-10527	ENSG00000102804	8m	TargetScan
miR-10527	ENSG00000102897	8m	TargetScan
miR-10527	ENSG00000102910	8m	TargetScan
miR-10527	ENSG00000103037	8m	TargetScan
miR-10527	ENSG00000103061	8m	TargetScan
miR-10527	ENSG00000103356	8m	TargetScan
miR-10527	ENSG00000103460	8m	TargetScan

miR-10527	ENSG00000103540	8m	TargetScan
miR-10527	ENSG00000103710	8m	TargetScan
miR-10527	ENSG00000103994	8m	TargetScan
miR-10527	ENSG00000104142	8m	TargetScan
miR-10527	ENSG00000104164	8m	TargetScan
miR-10527	ENSG00000104343	8m	TargetScan
miR-10527	ENSG00000104361	8m	TargetScan
miR-10527	ENSG00000104497	8m	TargetScan
miR-10527	ENSG00000104723	8m	TargetScan
miR-10527	ENSG00000104756	8m	TargetScan
miR-10527	ENSG00000105392	8m	TargetScan
miR-10527	ENSG00000105583	8m	TargetScan
miR-10527	ENSG00000105810	8m	TargetScan
miR-10527	ENSG00000105829	8m	TargetScan
miR-10527	ENSG00000105855	8m	TargetScan
miR-10527	ENSG00000105879	8m	TargetScan
miR-10527	ENSG00000106086	8m	TargetScan
miR-10527	ENSG00000106258	8m	TargetScan
miR-10527	ENSG00000106346	8m	TargetScan
miR-10527	ENSG00000106355	8m	TargetScan
miR-10527	ENSG00000106546	8m	TargetScan
miR-10527	ENSG00000106692	8m	TargetScan
miR-10527	ENSG00000106714	8m	TargetScan
miR-10527	ENSG00000106723	8m	TargetScan
miR-10527	ENSG00000106733	8m	TargetScan
miR-10527	ENSG00000106823	8m	TargetScan
miR-10527	ENSG00000107077	8m	TargetScan
miR-10527	ENSG00000107341	8m	TargetScan
miR-10527	ENSG00000107362	8m	TargetScan
miR-10527	ENSG00000107554	8m	TargetScan
miR-10527	ENSG00000107560	8m	TargetScan

miR-10527	ENSG00000107614	8m	TargetScan
miR-10527	ENSG00000107771	8m	TargetScan
miR-10527	ENSG00000108061	8m	TargetScan
miR-10527	ENSG00000108064	8m	TargetScan
miR-10527	ENSG00000108239	8m	TargetScan
miR-10527	ENSG00000108256	8m	TargetScan
miR-10527	ENSG00000108384	8m	TargetScan
miR-10527	ENSG00000108474	8m	TargetScan
miR-10527	ENSG00000108576	8m	TargetScan
miR-10527	ENSG00000108587	8m	TargetScan
miR-10527	ENSG00000108654	8m	TargetScan
miR-10527	ENSG00000108666	8m	TargetScan
miR-10527	ENSG00000108861	8m	TargetScan
miR-10527	ENSG00000108950	8m	TargetScan
miR-10527	ENSG00000108984	8m	TargetScan
miR-10527	ENSG00000109101	8m	TargetScan
miR-10527	ENSG00000109220	8m	TargetScan
miR-10527	ENSG00000109332	8m	TargetScan
miR-10527	ENSG00000109861	8m	TargetScan
miR-10527	ENSG00000109917	8m	TargetScan
miR-10527	ENSG00000110218	8m	TargetScan
miR-10527	ENSG00000110315	8m	TargetScan
miR-10527	ENSG00000110395	8m	TargetScan
miR-10527	ENSG00000110422	8m	TargetScan
miR-10527	ENSG00000110427	8m	TargetScan
miR-10527	ENSG00000110429	8m	TargetScan
miR-10527	ENSG00000110713	8m	TargetScan
miR-10527	ENSG00000110906	8m	TargetScan
miR-10527	ENSG00000111049	8m	TargetScan
miR-10527	ENSG00000111142	8m	TargetScan
miR-10527	ENSG00000111229	8m	TargetScan

miR-10527	ENSG00000111328	8m	TargetScan
miR-10527	ENSG00000111424	8m	TargetScan
miR-10527	ENSG00000111704	8m	TargetScan
miR-10527	ENSG00000111713	8m	TargetScan
miR-10527	ENSG00000111728	8m	TargetScan
miR-10527	ENSG00000111816	8m	TargetScan
miR-10527	ENSG00000111860	8m	TargetScan
miR-10527	ENSG00000111885	8m	TargetScan
miR-10527	ENSG00000112062	8m	TargetScan
miR-10527	ENSG00000112280	8m	TargetScan
miR-10527	ENSG00000112308	8m	TargetScan
miR-10527	ENSG00000112379	8m	TargetScan
miR-10527	ENSG00000112531	8m	TargetScan
miR-10527	ENSG00000112619	8m	TargetScan
miR-10527	ENSG00000112624	8m	TargetScan
miR-10527	ENSG00000112773	8m	TargetScan
miR-10527	ENSG00000112837	8m	TargetScan
miR-10527	ENSG00000112902	8m	TargetScan
miR-10527	ENSG00000113048	8m	TargetScan
miR-10527	ENSG00000113100	8m	TargetScan
miR-10527	ENSG00000113240	8m	TargetScan
miR-10527	ENSG00000113249	8m	TargetScan
miR-10527	ENSG00000113273	8m	TargetScan
miR-10527	ENSG00000113448	8m	TargetScan
miR-10527	ENSG00000113456	8m	TargetScan
miR-10527	ENSG00000113583	8m	TargetScan
miR-10527	ENSG00000113597	8m	TargetScan
miR-10527	ENSG00000113600	8m	TargetScan
miR-10527	ENSG00000113638	8m	TargetScan
miR-10527	ENSG00000113658	8m	TargetScan
miR-10527	ENSG00000113851	8m	TargetScan

miR-10527	ENSG00000114021	8m	TargetScan
miR-10527	ENSG00000114023	8m	TargetScan
miR-10527	ENSG00000114127	8m	TargetScan
miR-10527	ENSG00000114166	8m	TargetScan
miR-10527	ENSG00000114279	8m	TargetScan
miR-10527	ENSG00000114349	8m	TargetScan
miR-10527	ENSG00000114416	8m	TargetScan
miR-10527	ENSG00000114491	8m	TargetScan
miR-10527	ENSG00000114850	8m	TargetScan
miR-10527	ENSG00000114902	8m	TargetScan
miR-10527	ENSG00000114988	8m	TargetScan
miR-10527	ENSG00000115137	8m	TargetScan
miR-10527	ENSG00000115165	8m	TargetScan
miR-10527	ENSG00000115252	8m	TargetScan
miR-10527	ENSG00000115295	8m	TargetScan
miR-10527	ENSG00000115355	8m	TargetScan
miR-10527	ENSG00000115364	8m	TargetScan
miR-10527	ENSG00000115464	8m	TargetScan
miR-10527	ENSG00000115524	8m	TargetScan
miR-10527	ENSG00000115593	8m	TargetScan
miR-10527	ENSG00000115641	8m	TargetScan
miR-10527	ENSG00000115827	8m	TargetScan
miR-10527	ENSG00000115839	8m	TargetScan
miR-10527	ENSG00000115942	8m	TargetScan
miR-10527	ENSG00000116095	8m	TargetScan
miR-10527	ENSG00000116132	8m	TargetScan
miR-10527	ENSG00000116171	8m	TargetScan
miR-10527	ENSG00000116191	8m	TargetScan
miR-10527	ENSG00000116194	8m	TargetScan
miR-10527	ENSG00000116199	8m	TargetScan
miR-10527	ENSG00000116473	8m	TargetScan

miR-10527	ENSG00000116560	8m	TargetScan
miR-10527	ENSG00000116661	8m	TargetScan
miR-10527	ENSG00000116667	8m	TargetScan
miR-10527	ENSG00000116704	8m	TargetScan
miR-10527	ENSG00000116985	8m	TargetScan
miR-10527	ENSG00000117054	8m	TargetScan
miR-10527	ENSG00000117139	8m	TargetScan
miR-10527	ENSG00000117151	8m	TargetScan
miR-10527	ENSG00000117479	8m	TargetScan
miR-10527	ENSG00000117500	8m	TargetScan
miR-10527	ENSG00000117505	8m	TargetScan
miR-10527	ENSG00000117507	8m	TargetScan
miR-10527	ENSG00000117533	8m	TargetScan
miR-10527	ENSG00000117724	8m	TargetScan
miR-10527	ENSG00000117868	8m	TargetScan
miR-10527	ENSG00000117906	8m	TargetScan
miR-10527	ENSG00000118096	8m	TargetScan
miR-10527	ENSG00000118242	8m	TargetScan
miR-10527	ENSG00000118260	8m	TargetScan
miR-10527	ENSG00000118596	8m	TargetScan
miR-10527	ENSG00000118707	8m	TargetScan
miR-10527	ENSG00000118855	8m	TargetScan
miR-10527	ENSG00000119396	8m	TargetScan
miR-10527	ENSG00000119397	8m	TargetScan
miR-10527	ENSG00000119508	8m	TargetScan
miR-10527	ENSG00000119547	8m	TargetScan
miR-10527	ENSG00000119685	8m	TargetScan
miR-10527	ENSG00000119723	8m	TargetScan
miR-10527	ENSG00000119760	8m	TargetScan
miR-10527	ENSG00000119772	8m	TargetScan
miR-10527	ENSG00000119778	8m	TargetScan

miR-10527	ENSG00000119812	8m	TargetScan
miR-10527	ENSG00000119878	8m	TargetScan
miR-10527	ENSG00000119899	8m	TargetScan
miR-10527	ENSG00000119922	8m	TargetScan
miR-10527	ENSG00000119929	8m	TargetScan
miR-10527	ENSG00000119950	8m	TargetScan
miR-10527	ENSG00000120159	8m	TargetScan
miR-10527	ENSG00000120251	8m	TargetScan
miR-10527	ENSG00000120289	8m	TargetScan
miR-10527	ENSG00000120332	8m	TargetScan
miR-10527	ENSG00000120519	8m	TargetScan
miR-10527	ENSG00000120539	8m	TargetScan
miR-10527	ENSG00000120647	8m	TargetScan
miR-10527	ENSG00000120669	8m	TargetScan
miR-10527	ENSG00000120738	8m	TargetScan
miR-10527	ENSG00000120805	8m	TargetScan
miR-10527	ENSG00000120832	8m	TargetScan
miR-10527	ENSG00000120885	8m	TargetScan
miR-10527	ENSG00000121060	8m	TargetScan
miR-10527	ENSG00000121297	8m	TargetScan
miR-10527	ENSG00000121390	8m	TargetScan
miR-10527	ENSG00000121454	8m	TargetScan
miR-10527	ENSG00000121579	8m	TargetScan
miR-10527	ENSG00000121716	8m	TargetScan
miR-10527	ENSG00000121897	8m	TargetScan
miR-10527	ENSG00000121904	8m	TargetScan
miR-10527	ENSG00000121931	8m	TargetScan
miR-10527	ENSG00000122068	8m	TargetScan
miR-10527	ENSG00000122176	8m	TargetScan
miR-10527	ENSG00000122224	8m	TargetScan
miR-10527	ENSG00000122367	8m	TargetScan

miR-10527	ENSG00000122417	8m	TargetScan
miR-10527	ENSG00000122691	8m	TargetScan
miR-10527	ENSG00000123094	8m	TargetScan
miR-10527	ENSG00000123130	8m	TargetScan
miR-10527	ENSG00000123191	8m	TargetScan
miR-10527	ENSG00000123240	8m	TargetScan
miR-10527	ENSG00000123268	8m	TargetScan
miR-10527	ENSG00000123411	8m	TargetScan
miR-10527	ENSG00000124120	8m	TargetScan
miR-10527	ENSG00000124193	8m	TargetScan
miR-10527	ENSG00000124198	8m	TargetScan
miR-10527	ENSG00000124406	8m	TargetScan
miR-10527	ENSG00000124596	8m	TargetScan
miR-10527	ENSG00000124743	8m	TargetScan
miR-10527	ENSG00000124772	8m	TargetScan
miR-10527	ENSG00000124783	8m	TargetScan
miR-10527	ENSG00000124813	8m	TargetScan
miR-10527	ENSG00000125124	8m	TargetScan
miR-10527	ENSG00000125247	8m	TargetScan
miR-10527	ENSG00000125351	8m	TargetScan
miR-10527	ENSG00000125363	8m	TargetScan
miR-10527	ENSG00000125398	8m	TargetScan
miR-10527	ENSG00000125498	8m	TargetScan
miR-10527	ENSG00000125657	8m	TargetScan
miR-10527	ENSG00000125735	8m	TargetScan
miR-10527	ENSG00000125810	8m	TargetScan
miR-10527	ENSG00000125812	8m	TargetScan
miR-10527	ENSG00000125813	8m	TargetScan
miR-10527	ENSG00000125818	8m	TargetScan
miR-10527	ENSG00000125868	8m	TargetScan
miR-10527	ENSG00000125870	8m	TargetScan

miR-10527	ENSG00000126070	8m	TargetScan
miR-10527	ENSG00000126550	8m	TargetScan
miR-10527	ENSG00000126822	8m	TargetScan
miR-10527	ENSG00000126953	8m	TargetScan
miR-10527	ENSG00000127152	8m	TargetScan
miR-10527	ENSG00000127328	8m	TargetScan
miR-10527	ENSG00000127663	8m	TargetScan
miR-10527	ENSG00000127831	8m	TargetScan
miR-10527	ENSG00000128039	8m	TargetScan
miR-10527	ENSG00000128534	8m	TargetScan
miR-10527	ENSG00000128573	8m	TargetScan
miR-10527	ENSG00000128578	8m	TargetScan
miR-10527	ENSG00000128585	8m	TargetScan
miR-10527	ENSG00000128683	8m	TargetScan
miR-10527	ENSG00000128708	8m	TargetScan
miR-10527	ENSG00000128849	8m	TargetScan
miR-10527	ENSG00000128908	8m	TargetScan
miR-10527	ENSG00000128915	8m	TargetScan
miR-10527	ENSG00000129204	8m	TargetScan
miR-10527	ENSG00000129250	8m	TargetScan
miR-10527	ENSG00000129317	8m	TargetScan
miR-10527	ENSG00000129460	8m	TargetScan
miR-10527	ENSG00000129474	8m	TargetScan
miR-10527	ENSG00000129566	8m	TargetScan
miR-10527	ENSG00000129625	8m	TargetScan
miR-10527	ENSG00000129675	8m	TargetScan
miR-10527	ENSG00000130052	8m	TargetScan
miR-10527	ENSG00000130119	8m	TargetScan
miR-10527	ENSG00000130227	8m	TargetScan
miR-10527	ENSG00000130818	8m	TargetScan
miR-10527	ENSG00000130844	8m	TargetScan

miR-10527	ENSG00000131016	8m	TargetScan
miR-10527	ENSG00000131127	8m	TargetScan
miR-10527	ENSG00000131269	8m	TargetScan
miR-10527	ENSG00000131355	8m	TargetScan
miR-10527	ENSG00000131386	8m	TargetScan
miR-10527	ENSG00000131437	8m	TargetScan
miR-10527	ENSG00000131459	8m	TargetScan
miR-10527	ENSG00000131558	8m	TargetScan
miR-10527	ENSG00000132031	8m	TargetScan
miR-10527	ENSG00000132294	8m	TargetScan
miR-10527	ENSG00000132570	8m	TargetScan
miR-10527	ENSG00000132604	8m	TargetScan
miR-10527	ENSG00000132623	8m	TargetScan
miR-10527	ENSG00000132640	8m	TargetScan
miR-10527	ENSG00000132846	8m	TargetScan
miR-10527	ENSG00000132855	8m	TargetScan
miR-10527	ENSG00000132970	8m	TargetScan
miR-10527	ENSG00000133028	8m	TargetScan
miR-10527	ENSG00000133083	8m	TargetScan
miR-10527	ENSG00000133104	8m	TargetScan
miR-10527	ENSG00000133115	8m	TargetScan
miR-10527	ENSG00000133313	8m	TargetScan
miR-10527	ENSG00000133574	8m	TargetScan
miR-10527	ENSG00000133703	8m	TargetScan
miR-10527	ENSG00000133742	8m	TargetScan
miR-10527	ENSG00000134046	8m	TargetScan
miR-10527	ENSG00000134109	8m	TargetScan
miR-10527	ENSG00000134121	8m	TargetScan
miR-10527	ENSG00000134202	8m	TargetScan
miR-10527	ENSG00000134216	8m	TargetScan
miR-10527	ENSG00000134243	8m	TargetScan

miR-10527	ENSG00000134245	8m	TargetScan
miR-10527	ENSG00000134291	8m	TargetScan
miR-10527	ENSG00000134326	8m	TargetScan
miR-10527	ENSG00000134352	8m	TargetScan
miR-10527	ENSG00000134371	8m	TargetScan
miR-10527	ENSG00000134463	8m	TargetScan
miR-10527	ENSG00000134759	8m	TargetScan
miR-10527	ENSG00000134809	8m	TargetScan
miR-10527	ENSG00000134852	8m	TargetScan
miR-10527	ENSG00000134864	8m	TargetScan
miR-10527	ENSG00000134909	8m	TargetScan
miR-10527	ENSG00000134970	8m	TargetScan
miR-10527	ENSG00000135049	8m	TargetScan
miR-10527	ENSG00000135083	8m	TargetScan
miR-10527	ENSG00000135185	8m	TargetScan
miR-10527	ENSG00000135250	8m	TargetScan
miR-10527	ENSG00000135315	8m	TargetScan
miR-10527	ENSG00000135643	8m	TargetScan
miR-10527	ENSG00000135686	8m	TargetScan
miR-10527	ENSG00000135750	8m	TargetScan
miR-10527	ENSG00000135778	8m	TargetScan
miR-10527	ENSG00000135870	8m	TargetScan
miR-10527	ENSG00000135999	8m	TargetScan
miR-10527	ENSG00000136052	8m	TargetScan
miR-10527	ENSG00000136104	8m	TargetScan
miR-10527	ENSG00000136235	8m	TargetScan
miR-10527	ENSG00000136237	8m	TargetScan
miR-10527	ENSG00000136267	8m	TargetScan
miR-10527	ENSG00000136450	8m	TargetScan
miR-10527	ENSG00000136463	8m	TargetScan
miR-10527	ENSG00000136527	8m	TargetScan

miR-10527	ENSG00000136603	8m	TargetScan
miR-10527	ENSG00000136636	8m	TargetScan
miR-10527	ENSG00000136643	8m	TargetScan
miR-10527	ENSG00000136710	8m	TargetScan
miR-10527	ENSG00000136738	8m	TargetScan
miR-10527	ENSG00000136848	8m	TargetScan
miR-10527	ENSG00000136861	8m	TargetScan
miR-10527	ENSG00000136870	8m	TargetScan
miR-10527	ENSG00000136938	8m	TargetScan
miR-10527	ENSG00000137261	8m	TargetScan
miR-10527	ENSG00000137561	8m	TargetScan
miR-10527	ENSG00000137573	8m	TargetScan
miR-10527	ENSG00000137642	8m	TargetScan
miR-10527	ENSG00000137691	8m	TargetScan
miR-10527	ENSG00000137692	8m	TargetScan
miR-10527	ENSG00000137804	8m	TargetScan
miR-10527	ENSG00000137814	8m	TargetScan
miR-10527	ENSG00000137819	8m	TargetScan
miR-10527	ENSG00000137821	8m	TargetScan
miR-10527	ENSG00000137845	8m	TargetScan
miR-10527	ENSG00000137869	8m	TargetScan
miR-10527	ENSG00000137878	8m	TargetScan
miR-10527	ENSG00000138032	8m	TargetScan
miR-10527	ENSG00000138039	8m	TargetScan
miR-10527	ENSG00000138078	8m	TargetScan
miR-10527	ENSG00000138079	8m	TargetScan
miR-10527	ENSG00000138185	8m	TargetScan
miR-10527	ENSG00000138380	8m	TargetScan
miR-10527	ENSG00000138411	8m	TargetScan
miR-10527	ENSG00000138435	8m	TargetScan
miR-10527	ENSG00000138593	8m	TargetScan

miR-10527	ENSG00000138594	8m	TargetScan
miR-10527	ENSG00000138613	8m	TargetScan
miR-10527	ENSG00000138660	8m	TargetScan
miR-10527	ENSG00000138668	8m	TargetScan
miR-10527	ENSG00000138756	8m	TargetScan
miR-10527	ENSG00000138764	8m	TargetScan
miR-10527	ENSG00000138829	8m	TargetScan
miR-10527	ENSG00000139083	8m	TargetScan
miR-10527	ENSG00000139131	8m	TargetScan
miR-10527	ENSG00000139154	8m	TargetScan
miR-10527	ENSG00000139178	8m	TargetScan
miR-10527	ENSG00000139218	8m	TargetScan
miR-10527	ENSG00000139233	8m	TargetScan
miR-10527	ENSG00000139289	8m	TargetScan
miR-10527	ENSG00000139343	8m	TargetScan
miR-10527	ENSG00000139436	8m	TargetScan
miR-10527	ENSG00000139496	8m	TargetScan
miR-10527	ENSG00000139514	8m	TargetScan
miR-10527	ENSG00000139874	8m	TargetScan
miR-10527	ENSG00000139926	8m	TargetScan
miR-10527	ENSG00000139977	8m	TargetScan
miR-10527	ENSG00000140153	8m	TargetScan
miR-10527	ENSG00000140157	8m	TargetScan
miR-10527	ENSG00000140396	8m	TargetScan
miR-10527	ENSG00000140455	8m	TargetScan
miR-10527	ENSG00000140478	8m	TargetScan
miR-10527	ENSG00000140563	8m	TargetScan
miR-10527	ENSG00000140718	8m	TargetScan
miR-10527	ENSG00000140743	8m	TargetScan
miR-10527	ENSG00000140807	8m	TargetScan
miR-10527	ENSG00000141449	8m	TargetScan

miR-10527	ENSG00000141540	8m	TargetScan
miR-10527	ENSG00000141627	8m	TargetScan
miR-10527	ENSG00000141646	8m	TargetScan
miR-10527	ENSG00000141655	8m	TargetScan
miR-10527	ENSG00000142166	8m	TargetScan
miR-10527	ENSG00000143079	8m	TargetScan
miR-10527	ENSG00000143226	8m	TargetScan
miR-10527	ENSG00000143228	8m	TargetScan
miR-10527	ENSG00000143324	8m	TargetScan
miR-10527	ENSG00000143355	8m	TargetScan
miR-10527	ENSG00000143367	8m	TargetScan
miR-10527	ENSG00000143384	8m	TargetScan
miR-10527	ENSG00000143390	8m	TargetScan
miR-10527	ENSG00000143443	8m	TargetScan
miR-10527	ENSG00000143479	8m	TargetScan
miR-10527	ENSG00000143514	8m	TargetScan
miR-10527	ENSG00000143549	8m	TargetScan
miR-10527	ENSG00000143771	8m	TargetScan
miR-10527	ENSG00000143850	8m	TargetScan
miR-10527	ENSG00000144152	8m	TargetScan
miR-10527	ENSG00000144278	8m	TargetScan
miR-10527	ENSG00000144290	8m	TargetScan
miR-10527	ENSG00000144320	8m	TargetScan
miR-10527	ENSG00000144481	8m	TargetScan
miR-10527	ENSG00000144597	8m	TargetScan
miR-10527	ENSG00000144619	8m	TargetScan
miR-10527	ENSG00000144668	8m	TargetScan
miR-10527	ENSG00000144713	8m	TargetScan
miR-10527	ENSG00000144791	8m	TargetScan
miR-10527	ENSG00000144824	8m	TargetScan
miR-10527	ENSG00000144868	8m	TargetScan

miR-10527	ENSG00000144959	8m	TargetScan
miR-10527	ENSG00000145016	8m	TargetScan
miR-10527	ENSG00000145022	8m	TargetScan
miR-10527	ENSG00000145246	8m	TargetScan
miR-10527	ENSG00000145283	8m	TargetScan
miR-10527	ENSG00000145284	8m	TargetScan
miR-10527	ENSG00000145386	8m	TargetScan
miR-10527	ENSG00000145388	8m	TargetScan
miR-10527	ENSG00000145391	8m	TargetScan
miR-10527	ENSG00000145414	8m	TargetScan
miR-10527	ENSG00000145495	8m	TargetScan
miR-10527	ENSG00000145569	8m	TargetScan
miR-10527	ENSG00000145685	8m	TargetScan
miR-10527	ENSG00000145687	8m	TargetScan
miR-10527	ENSG00000145715	8m	TargetScan
miR-10527	ENSG00000145741	8m	TargetScan
miR-10527	ENSG00000145743	8m	TargetScan
miR-10527	ENSG00000145780	8m	TargetScan
miR-10527	ENSG00000145861	8m	TargetScan
miR-10527	ENSG00000145864	8m	TargetScan
miR-10527	ENSG00000146085	8m	TargetScan
miR-10527	ENSG00000146151	8m	TargetScan
miR-10527	ENSG00000146242	8m	TargetScan
miR-10527	ENSG00000146352	8m	TargetScan
miR-10527	ENSG00000146376	8m	TargetScan
miR-10527	ENSG00000146416	8m	TargetScan
miR-10527	ENSG00000146433	8m	TargetScan
miR-10527	ENSG00000146476	8m	TargetScan
miR-10527	ENSG00000146530	8m	TargetScan
miR-10527	ENSG00000146535	8m	TargetScan
miR-10527	ENSG00000146592	8m	TargetScan

miR-10527	ENSG00000146676	8m	TargetScan
miR-10527	ENSG00000146733	8m	TargetScan
miR-10527	ENSG00000146802	8m	TargetScan
miR-10527	ENSG00000146833	8m	TargetScan
miR-10527	ENSG00000146858	8m	TargetScan
miR-10527	ENSG00000147036	8m	TargetScan
miR-10527	ENSG00000147100	8m	TargetScan
miR-10527	ENSG00000147124	8m	TargetScan
miR-10527	ENSG00000147162	8m	TargetScan
miR-10527	ENSG00000147164	8m	TargetScan
miR-10527	ENSG00000147202	8m	TargetScan
miR-10527	ENSG00000147251	8m	TargetScan
miR-10527	ENSG00000147400	8m	TargetScan
miR-10527	ENSG00000147421	8m	TargetScan
miR-10527	ENSG00000147459	8m	TargetScan
miR-10527	ENSG00000147548	8m	TargetScan
miR-10527	ENSG00000147586	8m	TargetScan
miR-10527	ENSG00000147642	8m	TargetScan
miR-10527	ENSG00000147669	8m	TargetScan
miR-10527	ENSG00000147677	8m	TargetScan
miR-10527	ENSG00000147679	8m	TargetScan
miR-10527	ENSG00000147885	8m	TargetScan
miR-10527	ENSG00000148053	8m	TargetScan
miR-10527	ENSG00000148219	8m	TargetScan
miR-10527	ENSG00000148225	8m	TargetScan
miR-10527	ENSG00000148229	8m	TargetScan
miR-10527	ENSG00000148468	8m	TargetScan
miR-10527	ENSG00000148672	8m	TargetScan
miR-10527	ENSG00000148719	8m	TargetScan
miR-10527	ENSG00000148730	8m	TargetScan
miR-10527	ENSG00000148943	8m	TargetScan

miR-10527	ENSG00000149089	8m	TargetScan
miR-10527	ENSG00000149100	8m	TargetScan
miR-10527	ENSG00000149177	8m	TargetScan
miR-10527	ENSG00000149212	8m	TargetScan
miR-10527	ENSG00000149289	8m	TargetScan
miR-10527	ENSG00000149554	8m	TargetScan
miR-10527	ENSG00000149599	8m	TargetScan
miR-10527	ENSG00000149636	8m	TargetScan
miR-10527	ENSG00000149968	8m	TargetScan
miR-10527	ENSG00000149972	8m	TargetScan
miR-10527	ENSG00000150471	8m	TargetScan
miR-10527	ENSG00000150540	8m	TargetScan
miR-10527	ENSG00000150637	8m	TargetScan
miR-10527	ENSG00000150991	8m	TargetScan
miR-10527	ENSG00000151067	8m	TargetScan
miR-10527	ENSG00000151233	8m	TargetScan
miR-10527	ENSG00000151348	8m	TargetScan
miR-10527	ENSG00000151422	8m	TargetScan
miR-10527	ENSG00000151458	8m	TargetScan
miR-10527	ENSG00000151491	8m	TargetScan
miR-10527	ENSG00000151632	8m	TargetScan
miR-10527	ENSG00000151881	8m	TargetScan
miR-10527	ENSG00000151893	8m	TargetScan
miR-10527	ENSG00000152056	8m	TargetScan
miR-10527	ENSG00000152061	8m	TargetScan
miR-10527	ENSG00000152256	8m	TargetScan
miR-10527	ENSG00000152402	8m	TargetScan
miR-10527	ENSG00000152409	8m	TargetScan
miR-10527	ENSG00000152439	8m	TargetScan
miR-10527	ENSG00000152443	8m	TargetScan
miR-10527	ENSG00000152518	8m	TargetScan

miR-10527	ENSG00000152558	8m	TargetScan
miR-10527	ENSG00000152601	8m	TargetScan
miR-10527	ENSG00000152684	8m	TargetScan
miR-10527	ENSG00000152763	8m	TargetScan
miR-10527	ENSG00000152785	8m	TargetScan
miR-10527	ENSG00000153006	8m	TargetScan
miR-10527	ENSG00000153037	8m	TargetScan
miR-10527	ENSG00000153132	8m	TargetScan
miR-10527	ENSG00000153147	8m	TargetScan
miR-10527	ENSG00000153214	8m	TargetScan
miR-10527	ENSG00000153317	8m	TargetScan
miR-10527	ENSG00000153487	8m	TargetScan
miR-10527	ENSG00000153561	8m	TargetScan
miR-10527	ENSG00000153684	8m	TargetScan
miR-10527	ENSG00000153767	8m	TargetScan
miR-10527	ENSG00000153790	8m	TargetScan
miR-10527	ENSG00000153922	8m	TargetScan
miR-10527	ENSG00000153933	8m	TargetScan
miR-10527	ENSG00000154114	8m	TargetScan
miR-10527	ENSG00000154118	8m	TargetScan
miR-10527	ENSG00000154124	8m	TargetScan
miR-10527	ENSG00000154144	8m	TargetScan
miR-10527	ENSG00000154162	8m	TargetScan
miR-10527	ENSG00000154174	8m	TargetScan
miR-10527	ENSG00000154217	8m	TargetScan
miR-10527	ENSG00000154227	8m	TargetScan
miR-10527	ENSG00000154310	8m	TargetScan
miR-10527	ENSG00000154359	8m	TargetScan
miR-10527	ENSG00000154415	8m	TargetScan
miR-10527	ENSG00000154654	8m	TargetScan
miR-10527	ENSG00000154678	8m	TargetScan

miR-10527	ENSG00000154719	8m	TargetScan
miR-10527	ENSG00000154736	8m	TargetScan
miR-10527	ENSG00000154889	8m	TargetScan
miR-10527	ENSG00000155052	8m	TargetScan
miR-10527	ENSG00000155097	8m	TargetScan
miR-10527	ENSG00000155100	8m	TargetScan
miR-10527	ENSG00000155189	8m	TargetScan
miR-10527	ENSG00000155636	8m	TargetScan
miR-10527	ENSG00000155754	8m	TargetScan
miR-10527	ENSG00000155827	8m	TargetScan
miR-10527	ENSG00000155850	8m	TargetScan
miR-10527	ENSG00000155868	8m	TargetScan
miR-10527	ENSG00000155957	8m	TargetScan
miR-10527	ENSG00000155975	8m	TargetScan
miR-10527	ENSG00000156096	8m	TargetScan
miR-10527	ENSG00000156097	8m	TargetScan
miR-10527	ENSG00000156113	8m	TargetScan
miR-10527	ENSG00000156239	8m	TargetScan
miR-10527	ENSG00000156269	8m	TargetScan
miR-10527	ENSG00000156284	8m	TargetScan
miR-10527	ENSG00000156508	8m	TargetScan
miR-10527	ENSG00000156535	8m	TargetScan
miR-10527	ENSG00000156671	8m	TargetScan
miR-10527	ENSG00000156853	8m	TargetScan
miR-10527	ENSG00000157106	8m	TargetScan
miR-10527	ENSG00000157315	8m	TargetScan
miR-10527	ENSG00000157500	8m	TargetScan
miR-10527	ENSG00000157600	8m	TargetScan
miR-10527	ENSG00000157680	8m	TargetScan
miR-10527	ENSG00000157741	8m	TargetScan
miR-10527	ENSG00000157764	8m	TargetScan

miR-10527	ENSG00000157985	8m	TargetScan
miR-10527	ENSG00000158006	8m	TargetScan
miR-10527	ENSG00000158290	8m	TargetScan
miR-10527	ENSG00000158296	8m	TargetScan
miR-10527	ENSG00000158352	8m	TargetScan
miR-10527	ENSG00000158373	8m	TargetScan
miR-10527	ENSG00000158528	8m	TargetScan
miR-10527	ENSG00000158683	8m	TargetScan
miR-10527	ENSG00000158805	8m	TargetScan
miR-10527	ENSG00000159167	8m	TargetScan
miR-10527	ENSG00000159197	8m	TargetScan
miR-10527	ENSG00000159217	8m	TargetScan
miR-10527	ENSG00000159251	8m	TargetScan
miR-10527	ENSG00000159263	8m	TargetScan
miR-10527	ENSG00000159289	8m	TargetScan
miR-10527	ENSG00000159692	8m	TargetScan
miR-10527	ENSG00000160007	8m	TargetScan
miR-10527	ENSG00000160679	8m	TargetScan
miR-10527	ENSG00000160959	8m	TargetScan
miR-10527	ENSG00000161547	8m	TargetScan
miR-10527	ENSG00000161791	8m	TargetScan
miR-10527	ENSG00000161929	8m	TargetScan
miR-10527	ENSG00000162105	8m	TargetScan
miR-10527	ENSG00000162174	8m	TargetScan
miR-10527	ENSG00000162241	8m	TargetScan
miR-10527	ENSG00000162368	8m	TargetScan
miR-10527	ENSG00000162402	8m	TargetScan
miR-10527	ENSG00000162438	8m	TargetScan
miR-10527	ENSG00000162441	8m	TargetScan
miR-10527	ENSG00000162616	8m	TargetScan
miR-10527	ENSG00000162642	8m	TargetScan

miR-10527	ENSG00000162654	8m	TargetScan
miR-10527	ENSG00000162664	8m	TargetScan
miR-10527	ENSG00000162695	8m	TargetScan
miR-10527	ENSG00000162769	8m	TargetScan
miR-10527	ENSG00000162928	8m	TargetScan
miR-10527	ENSG00000162980	8m	TargetScan
miR-10527	ENSG00000162999	8m	TargetScan
miR-10527	ENSG00000163110	8m	TargetScan
miR-10527	ENSG00000163249	8m	TargetScan
miR-10527	ENSG00000163281	8m	TargetScan
miR-10527	ENSG00000163291	8m	TargetScan
miR-10527	ENSG00000163297	8m	TargetScan
miR-10527	ENSG00000163428	8m	TargetScan
miR-10527	ENSG00000163430	8m	TargetScan
miR-10527	ENSG00000163481	8m	TargetScan
miR-10527	ENSG00000163492	8m	TargetScan
miR-10527	ENSG00000163513	8m	TargetScan
miR-10527	ENSG00000163581	8m	TargetScan
miR-10527	ENSG00000163584	8m	TargetScan
miR-10527	ENSG00000163590	8m	TargetScan
miR-10527	ENSG00000163602	8m	TargetScan
miR-10527	ENSG00000163605	8m	TargetScan
miR-10527	ENSG00000163611	8m	TargetScan
miR-10527	ENSG00000163637	8m	TargetScan
miR-10527	ENSG00000163644	8m	TargetScan
miR-10527	ENSG00000163689	8m	TargetScan
miR-10527	ENSG00000163728	8m	TargetScan
miR-10527	ENSG00000163812	8m	TargetScan
miR-10527	ENSG00000163818	8m	TargetScan
miR-10527	ENSG00000163833	8m	TargetScan
miR-10527	ENSG00000163900	8m	TargetScan

miR-10527	ENSG00000163909	8m	TargetScan
miR-10527	ENSG00000164024	8m	TargetScan
miR-10527	ENSG00000164056	8m	TargetScan
miR-10527	ENSG00000164087	8m	TargetScan
miR-10527	ENSG00000164116	8m	TargetScan
miR-10527	ENSG00000164122	8m	TargetScan
miR-10527	ENSG00000164181	8m	TargetScan
miR-10527	ENSG00000164241	8m	TargetScan
miR-10527	ENSG00000164244	8m	TargetScan
miR-10527	ENSG00000164303	8m	TargetScan
miR-10527	ENSG00000164307	8m	TargetScan
miR-10527	ENSG00000164323	8m	TargetScan
miR-10527	ENSG00000164338	8m	TargetScan
miR-10527	ENSG00000164483	8m	TargetScan
miR-10527	ENSG00000164485	8m	TargetScan
miR-10527	ENSG00000164506	8m	TargetScan
miR-10527	ENSG00000164532	8m	TargetScan
miR-10527	ENSG00000164619	8m	TargetScan
miR-10527	ENSG00000164684	8m	TargetScan
miR-10527	ENSG00000164691	8m	TargetScan
miR-10527	ENSG00000164746	8m	TargetScan
miR-10527	ENSG00000164764	8m	TargetScan
miR-10527	ENSG00000164941	8m	TargetScan
miR-10527	ENSG00000164953	8m	TargetScan
miR-10527	ENSG00000164976	8m	TargetScan
miR-10527	ENSG00000164983	8m	TargetScan
miR-10527	ENSG00000165028	8m	TargetScan
miR-10527	ENSG00000165084	8m	TargetScan
miR-10527	ENSG00000165185	8m	TargetScan
miR-10527	ENSG00000165186	8m	TargetScan
miR-10527	ENSG00000165195	8m	TargetScan

miR-10527	ENSG00000165244	8m	TargetScan
miR-10527	ENSG00000165338	8m	TargetScan
miR-10527	ENSG00000165416	8m	TargetScan
miR-10527	ENSG00000165417	8m	TargetScan
miR-10527	ENSG00000165458	8m	TargetScan
miR-10527	ENSG00000165474	8m	TargetScan
miR-10527	ENSG00000165556	8m	TargetScan
miR-10527	ENSG00000165566	8m	TargetScan
miR-10527	ENSG00000165623	8m	TargetScan
miR-10527	ENSG00000165671	8m	TargetScan
miR-10527	ENSG00000165714	8m	TargetScan
miR-10527	ENSG00000165775	8m	TargetScan
miR-10527	ENSG00000165959	8m	TargetScan
miR-10527	ENSG00000166128	8m	TargetScan
miR-10527	ENSG00000166147	8m	TargetScan
miR-10527	ENSG00000166206	8m	TargetScan
miR-10527	ENSG00000166233	8m	TargetScan
miR-10527	ENSG00000166262	8m	TargetScan
miR-10527	ENSG00000166351	8m	TargetScan
miR-10527	ENSG00000166402	8m	TargetScan
miR-10527	ENSG00000166435	8m	TargetScan
miR-10527	ENSG00000166441	8m	TargetScan
miR-10527	ENSG00000166450	8m	TargetScan
miR-10527	ENSG00000166478	8m	TargetScan
miR-10527	ENSG00000166503	8m	TargetScan
miR-10527	ENSG00000166510	8m	TargetScan
miR-10527	ENSG00000166575	8m	TargetScan
miR-10527	ENSG00000166783	8m	TargetScan
miR-10527	ENSG00000166803	8m	TargetScan
miR-10527	ENSG00000166900	8m	TargetScan
miR-10527	ENSG00000166927	8m	TargetScan

miR-10527	ENSG00000167186	8m	TargetScan
miR-10527	ENSG00000167195	8m	TargetScan
miR-10527	ENSG00000167196	8m	TargetScan
miR-10527	ENSG00000167202	8m	TargetScan
miR-10527	ENSG00000167232	8m	TargetScan
miR-10527	ENSG00000167315	8m	TargetScan
miR-10527	ENSG00000167526	8m	TargetScan
miR-10527	ENSG00000167633	8m	TargetScan
miR-10527	ENSG00000167642	8m	TargetScan
miR-10527	ENSG00000167910	8m	TargetScan
miR-10527	ENSG00000168032	8m	TargetScan
miR-10527	ENSG00000168172	8m	TargetScan
miR-10527	ENSG00000168175	8m	TargetScan
miR-10527	ENSG00000168288	8m	TargetScan
miR-10527	ENSG00000168297	8m	TargetScan
miR-10527	ENSG00000168538	8m	TargetScan
miR-10527	ENSG00000168556	8m	TargetScan
miR-10527	ENSG00000168672	8m	TargetScan
miR-10527	ENSG00000168769	8m	TargetScan
miR-10527	ENSG00000168876	8m	TargetScan
miR-10527	ENSG00000168903	8m	TargetScan
miR-10527	ENSG00000168939	8m	TargetScan
miR-10527	ENSG00000168939	8m	TargetScan
miR-10527	ENSG00000168944	8m	TargetScan
miR-10527	ENSG00000169047	8m	TargetScan
miR-10527	ENSG00000169139	8m	TargetScan
miR-10527	ENSG00000169184	8m	TargetScan
miR-10527	ENSG00000169239	8m	TargetScan
miR-10527	ENSG00000169282	8m	TargetScan
miR-10527	ENSG00000169306	8m	TargetScan
miR-10527	ENSG00000169375	8m	TargetScan

miR-10527	ENSG00000169446	8m	TargetScan
miR-10527	ENSG00000169554	8m	TargetScan
miR-10527	ENSG00000169836	8m	TargetScan
miR-10527	ENSG00000169905	8m	TargetScan
miR-10527	ENSG00000169908	8m	TargetScan
miR-10527	ENSG00000169925	8m	TargetScan
miR-10527	ENSG00000169981	8m	TargetScan
miR-10527	ENSG00000170035	8m	TargetScan
miR-10527	ENSG00000170074	8m	TargetScan
miR-10527	ENSG00000170075	8m	TargetScan
miR-10527	ENSG00000170088	8m	TargetScan
miR-10527	ENSG00000170145	8m	TargetScan
miR-10527	ENSG00000170162	8m	TargetScan
miR-10527	ENSG00000170166	8m	TargetScan
miR-10527	ENSG00000170345	8m	TargetScan
miR-10527	ENSG00000170348	8m	TargetScan
miR-10527	ENSG00000170545	8m	TargetScan
miR-10527	ENSG00000170558	8m	TargetScan
miR-10527	ENSG00000170624	8m	TargetScan
miR-10527	ENSG00000170634	8m	TargetScan
miR-10527	ENSG00000170653	8m	TargetScan
miR-10527	ENSG00000170681	8m	TargetScan
miR-10527	ENSG00000170832	8m	TargetScan
miR-10527	ENSG00000170836	8m	TargetScan
miR-10527	ENSG00000170854	8m	TargetScan
miR-10527	ENSG00000170892	8m	TargetScan
miR-10527	ENSG00000170927	8m	TargetScan
miR-10527	ENSG00000170949	8m	TargetScan
miR-10527	ENSG00000171016	8m	TargetScan
miR-10527	ENSG00000171033	8m	TargetScan
miR-10527	ENSG00000171150	8m	TargetScan

miR-10527	ENSG00000171208	8m	TargetScan
miR-10527	ENSG00000171227	8m	TargetScan
miR-10527	ENSG00000171310	8m	TargetScan
miR-10527	ENSG00000171316	8m	TargetScan
miR-10527	ENSG00000171320	8m	TargetScan
miR-10527	ENSG00000171385	8m	TargetScan
miR-10527	ENSG00000171444	8m	TargetScan
miR-10527	ENSG00000171448	8m	TargetScan
miR-10527	ENSG00000171490	8m	TargetScan
miR-10527	ENSG00000171492	8m	TargetScan
miR-10527	ENSG00000171634	8m	TargetScan
miR-10527	ENSG00000171649	8m	TargetScan
miR-10527	ENSG00000171659	8m	TargetScan
miR-10527	ENSG00000171757	8m	TargetScan
miR-10527	ENSG00000171865	8m	TargetScan
miR-10527	ENSG00000171885	8m	TargetScan
miR-10527	ENSG00000172081	8m	TargetScan
miR-10527	ENSG00000172167	8m	TargetScan
miR-10527	ENSG00000172209	8m	TargetScan
miR-10527	ENSG00000172273	8m	TargetScan
miR-10527	ENSG00000172340	8m	TargetScan
miR-10527	ENSG00000172379	8m	TargetScan
miR-10527	ENSG00000172399	8m	TargetScan
miR-10527	ENSG00000172493	8m	TargetScan
miR-10527	ENSG00000172572	8m	TargetScan
miR-10527	ENSG00000172578	8m	TargetScan
miR-10527	ENSG00000172728	8m	TargetScan
miR-10527	ENSG00000172733	8m	TargetScan
miR-10527	ENSG00000172795	8m	TargetScan
miR-10527	ENSG00000172939	8m	TargetScan
miR-10527	ENSG00000172943	8m	TargetScan

miR-10527	ENSG00000173068	8m	TargetScan
miR-10527	ENSG00000173083	8m	TargetScan
miR-10527	ENSG00000173110	8m	TargetScan
miR-10527	ENSG00000173141	8m	TargetScan
miR-10527	ENSG00000173209	8m	TargetScan
miR-10527	ENSG00000173276	8m	TargetScan
miR-10527	ENSG00000173611	8m	TargetScan
miR-10527	ENSG00000173614	8m	TargetScan
miR-10527	ENSG00000173626	8m	TargetScan
miR-10527	ENSG00000173627	8m	TargetScan
miR-10527	ENSG00000173674	8m	TargetScan
miR-10527	ENSG00000173681	8m	TargetScan
miR-10527	ENSG00000173692	8m	TargetScan
miR-10527	ENSG00000173805	8m	TargetScan
miR-10527	ENSG00000174099	8m	TargetScan
miR-10527	ENSG00000174130	8m	TargetScan
miR-10527	ENSG00000174206	8m	TargetScan
miR-10527	ENSG00000174370	8m	TargetScan
miR-10527	ENSG00000174437	8m	TargetScan
miR-10527	ENSG00000174473	8m	TargetScan
miR-10527	ENSG00000174840	8m	TargetScan
miR-10527	ENSG00000174891	8m	TargetScan
miR-10527	ENSG00000174989	8m	TargetScan
miR-10527	ENSG00000175029	8m	TargetScan
miR-10527	ENSG00000175066	8m	TargetScan
miR-10527	ENSG00000175073	8m	TargetScan
miR-10527	ENSG00000175215	8m	TargetScan
miR-10527	ENSG00000175426	8m	TargetScan
miR-10527	ENSG00000175787	8m	TargetScan
miR-10527	ENSG00000175806	8m	TargetScan
miR-10527	ENSG00000175874	8m	TargetScan

miR-10527	ENSG00000175893	8m	TargetScan
miR-10527	ENSG00000176049	8m	TargetScan
miR-10527	ENSG00000176102	8m	TargetScan
miR-10527	ENSG00000176222	8m	TargetScan
miR-10527	ENSG00000176463	8m	TargetScan
miR-10527	ENSG00000176595	8m	TargetScan
miR-10527	ENSG00000176597	8m	TargetScan
miR-10527	ENSG00000176658	8m	TargetScan
miR-10527	ENSG00000176907	8m	TargetScan
miR-10527	ENSG00000176945	8m	TargetScan
miR-10527	ENSG00000176971	8m	TargetScan
miR-10527	ENSG00000177181	8m	TargetScan
miR-10527	ENSG00000177182	8m	TargetScan
miR-10527	ENSG00000177383	8m	TargetScan
miR-10527	ENSG00000177425	8m	TargetScan
miR-10527	ENSG00000177511	8m	TargetScan
miR-10527	ENSG00000177570	8m	TargetScan
miR-10527	ENSG00000177602	8m	TargetScan
miR-10527	ENSG00000177606	8m	TargetScan
miR-10527	ENSG00000177989	8m	TargetScan
miR-10527	ENSG00000178074	8m	TargetScan
miR-10527	ENSG00000178177	8m	TargetScan
miR-10527	ENSG00000178425	8m	TargetScan
miR-10527	ENSG00000178502	8m	TargetScan
miR-10527	ENSG00000178567	8m	TargetScan
miR-10527	ENSG00000178568	8m	TargetScan
miR-10527	ENSG00000178573	8m	TargetScan
miR-10527	ENSG00000178607	8m	TargetScan
miR-10527	ENSG00000178694	8m	TargetScan
miR-10527	ENSG00000178700	8m	TargetScan
miR-10527	ENSG00000179008	8m	TargetScan

miR-10527	ENSG00000179195	8m	TargetScan
miR-10527	ENSG00000179387	8m	TargetScan
miR-10527	ENSG00000179813	8m	TargetScan
miR-10527	ENSG00000180008	8m	TargetScan
miR-10527	ENSG00000180354	8m	TargetScan
miR-10527	ENSG00000180488	8m	TargetScan
miR-10527	ENSG00000180530	8m	TargetScan
miR-10527	ENSG00000180611	8m	TargetScan
miR-10527	ENSG00000180667	8m	TargetScan
miR-10527	ENSG00000180776	8m	TargetScan
miR-10527	ENSG00000180869	8m	TargetScan
miR-10527	ENSG00000180901	8m	TargetScan
miR-10527	ENSG00000180964	8m	TargetScan
miR-10527	ENSG00000180998	8m	TargetScan
miR-10527	ENSG00000181016	8m	TargetScan
miR-10527	ENSG00000181031	8m	TargetScan
miR-10527	ENSG00000181450	8m	TargetScan
miR-10527	ENSG00000181631	8m	TargetScan
miR-10527	ENSG00000181704	8m	TargetScan
miR-10527	ENSG00000181722	8m	TargetScan
miR-10527	ENSG00000181908	8m	TargetScan
miR-10527	ENSG00000182013	8m	TargetScan
miR-10527	ENSG00000182230	8m	TargetScan
miR-10527	ENSG00000182263	8m	TargetScan
miR-10527	ENSG00000182348	8m	TargetScan
miR-10527	ENSG00000182389	8m	TargetScan
miR-10527	ENSG00000182446	8m	TargetScan
miR-10527	ENSG00000182463	8m	TargetScan
miR-10527	ENSG00000182552	8m	TargetScan
miR-10527	ENSG00000182568	8m	TargetScan
miR-10527	ENSG00000182575	8m	TargetScan

miR-10527	ENSG00000182667	8m	TargetScan
miR-10527	ENSG00000182836	8m	TargetScan
miR-10527	ENSG00000182919	8m	TargetScan
miR-10527	ENSG00000182968	8m	TargetScan
miR-10527	ENSG00000183023	8m	TargetScan
miR-10527	ENSG00000183145	8m	TargetScan
miR-10527	ENSG00000183291	8m	TargetScan
miR-10527	ENSG00000183323	8m	TargetScan
miR-10527	ENSG00000183454	8m	TargetScan
miR-10527	ENSG00000183530	8m	TargetScan
miR-10527	ENSG00000183729	8m	TargetScan
miR-10527	ENSG00000183742	8m	TargetScan
miR-10527	ENSG00000184083	8m	TargetScan
miR-10527	ENSG00000184206	8m	TargetScan
miR-10527	ENSG00000184226	8m	TargetScan
miR-10527	ENSG00000184349	8m	TargetScan
miR-10527	ENSG00000184574	8m	TargetScan
miR-10527	ENSG00000184792	8m	TargetScan
miR-10527	ENSG00000184992	8m	TargetScan
miR-10527	ENSG00000184995	8m	TargetScan
miR-10527	ENSG00000185008	8m	TargetScan
miR-10527	ENSG00000185046	8m	TargetScan
miR-10527	ENSG00000185070	8m	TargetScan
miR-10527	ENSG00000185088	8m	TargetScan
miR-10527	ENSG00000185090	8m	TargetScan
miR-10527	ENSG00000185149	8m	TargetScan
miR-10527	ENSG00000185163	8m	TargetScan
miR-10527	ENSG00000185219	8m	TargetScan
miR-10527	ENSG00000185247	8m	TargetScan
miR-10527	ENSG00000185278	8m	TargetScan
miR-10527	ENSG00000185379	8m	TargetScan

miR-10527	ENSG00000185414	8m	TargetScan
miR-10527	ENSG00000185551	8m	TargetScan
miR-10527	ENSG00000185565	8m	TargetScan
miR-10527	ENSG00000185591	8m	TargetScan
miR-10527	ENSG00000185658	8m	TargetScan
miR-10527	ENSG00000185722	8m	TargetScan
miR-10527	ENSG00000185829	8m	TargetScan
miR-10527	ENSG00000185947	8m	TargetScan
miR-10527	ENSG00000186094	8m	TargetScan
miR-10527	ENSG00000186265	8m	TargetScan
miR-10527	ENSG00000186272	8m	TargetScan
miR-10527	ENSG00000186298	8m	TargetScan
miR-10527	ENSG00000186399	8m	TargetScan
miR-10527	ENSG00000186479	8m	TargetScan
miR-10527	ENSG00000186529	8m	TargetScan
miR-10527	ENSG00000186960	8m	TargetScan
miR-10527	ENSG00000187049	8m	TargetScan
miR-10527	ENSG00000187187	8m	TargetScan
miR-10527	ENSG00000187189	8m	TargetScan
miR-10527	ENSG00000187231	8m	TargetScan
miR-10527	ENSG00000187446	8m	TargetScan
miR-10527	ENSG00000187581	8m	TargetScan
miR-10527	ENSG00000187605	8m	TargetScan
miR-10527	ENSG00000187626	8m	TargetScan
miR-10527	ENSG00000187676	8m	TargetScan
miR-10527	ENSG00000187699	8m	TargetScan
miR-10527	ENSG00000187824	8m	TargetScan
miR-10527	ENSG00000187942	8m	TargetScan
miR-10527	ENSG00000187954	8m	TargetScan
miR-10527	ENSG00000188001	8m	TargetScan
miR-10527	ENSG00000188021	8m	TargetScan

miR-10527	ENSG00000188107	8m	TargetScan
miR-10527	ENSG00000188419	8m	TargetScan
miR-10527	ENSG00000188610	8m	TargetScan
miR-10527	ENSG00000188626	8m	TargetScan
miR-10527	ENSG00000188786	8m	TargetScan
miR-10527	ENSG00000188937	8m	TargetScan
miR-10527	ENSG00000189013	8m	TargetScan
miR-10527	ENSG00000189046	8m	TargetScan
miR-10527	ENSG00000189164	8m	TargetScan
miR-10527	ENSG00000189180	8m	TargetScan
miR-10527	ENSG00000189319	8m	TargetScan
miR-10527	ENSG00000189362	8m	TargetScan
miR-10527	ENSG00000196110	8m	TargetScan
miR-10527	ENSG00000196199	8m	TargetScan
miR-10527	ENSG00000196220	8m	TargetScan
miR-10527	ENSG00000196247	8m	TargetScan
miR-10527	ENSG00000196262	8m	TargetScan
miR-10527	ENSG00000196352	8m	TargetScan
miR-10527	ENSG00000196368	8m	TargetScan
miR-10527	ENSG00000196405	8m	TargetScan
miR-10527	ENSG00000196417	8m	TargetScan
miR-10527	ENSG00000196455	8m	TargetScan
miR-10527	ENSG00000196504	8m	TargetScan
miR-10527	ENSG00000196505	8m	TargetScan
miR-10527	ENSG00000196510	8m	TargetScan
miR-10527	ENSG00000196550	8m	TargetScan
miR-10527	ENSG00000196693	8m	TargetScan
miR-10527	ENSG00000196705	8m	TargetScan
miR-10527	ENSG00000196792	8m	TargetScan
miR-10527	ENSG00000196932	8m	TargetScan
miR-10527	ENSG00000196968	8m	TargetScan

miR-10527	ENSG00000197045	8m	TargetScan
miR-10527	ENSG00000197077	8m	TargetScan
miR-10527	ENSG00000197128	8m	TargetScan
miR-10527	ENSG00000197172	8m	TargetScan
miR-10527	ENSG00000197296	8m	TargetScan
miR-10527	ENSG00000197312	8m	TargetScan
miR-10527	ENSG00000197363	8m	TargetScan
miR-10527	ENSG00000197430	8m	TargetScan
miR-10527	ENSG00000197465	8m	TargetScan
miR-10527	ENSG00000197555	8m	TargetScan
miR-10527	ENSG00000197557	8m	TargetScan
miR-10527	ENSG00000197622	8m	TargetScan
miR-10527	ENSG00000197702	8m	TargetScan
miR-10527	ENSG00000197714	8m	TargetScan
miR-10527	ENSG00000197766	8m	TargetScan
miR-10527	ENSG00000197863	8m	TargetScan
miR-10527	ENSG00000197892	8m	TargetScan
miR-10527	ENSG00000197969	8m	TargetScan
miR-10527	ENSG00000198039	8m	TargetScan
miR-10527	ENSG00000198081	8m	TargetScan
miR-10527	ENSG00000198160	8m	TargetScan
miR-10527	ENSG00000198205	8m	TargetScan
miR-10527	ENSG00000198265	8m	TargetScan
miR-10527	ENSG00000198315	8m	TargetScan
miR-10527	ENSG00000198380	8m	TargetScan
miR-10527	ENSG00000198574	8m	TargetScan
miR-10527	ENSG00000198612	8m	TargetScan
miR-10527	ENSG00000198633	8m	TargetScan
miR-10527	ENSG00000198663	8m	TargetScan
miR-10527	ENSG00000198673	8m	TargetScan
miR-10527	ENSG00000198690	8m	TargetScan

miR-10527	ENSG00000198718	8m	TargetScan
miR-10527	ENSG00000198739	8m	TargetScan
miR-10527	ENSG00000198740	8m	TargetScan
miR-10527	ENSG00000198774	8m	TargetScan
miR-10527	ENSG00000198799	8m	TargetScan
miR-10527	ENSG00000198805	8m	TargetScan
miR-10527	ENSG00000198815	8m	TargetScan
miR-10527	ENSG00000198843	8m	TargetScan
miR-10527	ENSG00000198890	8m	TargetScan
miR-10527	ENSG00000203666	8m	TargetScan
miR-10527	ENSG00000203710	8m	TargetScan
miR-10527	ENSG00000204103	8m	TargetScan
miR-10527	ENSG00000204252	8m	TargetScan
miR-10527	ENSG00000204271	8m	TargetScan
miR-10527	ENSG00000204392	8m	TargetScan
miR-10527	ENSG00000204677	8m	TargetScan
miR-10527	ENSG00000204767	8m	TargetScan
miR-10527	ENSG00000204954	8m	TargetScan
miR-10527	ENSG00000205189	8m	TargetScan
miR-10527	ENSG00000205531	8m	TargetScan
miR-10527	ENSG00000206127	8m	TargetScan
miR-10527	ENSG00000206262	8m	TargetScan
miR-10527	ENSG00000206418	8m	TargetScan
miR-10527	ENSG00000206503	8m	TargetScan
miR-10527	ENSG00000206538	8m	TargetScan
miR-10527	ENSG00000206562	8m	TargetScan
miR-10527	ENSG00000206579	8m	TargetScan
miR-10527	ENSG00000212747	8m	TargetScan
miR-10527	ENSG00000213020	8m	TargetScan
miR-10527	ENSG00000213186	8m	TargetScan
miR-10527	ENSG00000213380	8m	TargetScan

miR-10527	ENSG00000213463	8m	TargetScan
miR-10527	ENSG00000213923	8m	TargetScan
miR-10527	ENSG00000214114	8m	TargetScan
miR-10527	ENSG00000214274	8m	TargetScan
miR-10527	ENSG00000214435	8m	TargetScan
miR-10527	ENSG00000214736	8m	TargetScan
miR-10527	ENSG00000214753	8m	TargetScan
miR-10527	ENSG00000214756	8m	TargetScan
miR-10527	ENSG00000215186	8m	TargetScan
miR-10527	ENSG00000215271	8m	TargetScan
miR-10527	ENSG00000215784	8m	TargetScan
miR-10527	ENSG00000217128	8m	TargetScan
miR-10527	ENSG00000218336	8m	TargetScan
miR-10527	ENSG00000218739	8m	TargetScan
miR-10527	ENSG00000221818	8m	TargetScan
miR-10527	ENSG00000221866	8m	TargetScan
miR-10527	ENSG00000221867	8m	TargetScan
miR-10527	ENSG00000221963	8m	TargetScan
miR-10527	ENSG00000225830	8m	TargetScan
miR-10527	ENSG00000226887	8m	TargetScan
miR-10527	ENSG00000228075	8m	TargetScan
miR-10527	ENSG00000228172	8m	TargetScan
miR-10527	ENSG00000228696	8m	TargetScan
miR-10527	ENSG00000232044	8m	TargetScan
miR-10527	ENSG00000232653	8m	TargetScan
miR-10527	ENSG00000232810	8m	TargetScan
miR-10527	ENSG00000235194	8m	TargetScan
miR-10527	ENSG00000236027	8m	TargetScan
miR-10527	ENSG00000237136	8m	TargetScan
miR-10527	ENSG00000240403	8m	TargetScan
miR-10527	ENSG00000240694	8m	TargetScan

miR-10527	ENSG00000241489	8m	TargetScan
miR-10527	ENSG00000243772	8m	TargetScan
miR-10527	ENSG00000244754	8m	TargetScan
miR-10527	ENSG00000248538	8m	TargetScan
miR-10527	ENSG00000248905	8m	TargetScan
miR-10527	ENSG00000249931	8m	TargetScan
miR-10527	ENSG00000250423	8m	TargetScan
miR-10527	ENSG00000251201	8m	TargetScan
miR-10527	ENSG00000253207	8m	TargetScan
miR-10527	ENSG00000254004	8m	TargetScan
miR-10527	ENSG00000255529	8m	TargetScan
miR-10527	ENSG00000256043	8m	TargetScan
miR-10527	ENSG00000256087	8m	TargetScan
miR-10527	ENSG00000257315	8m	TargetScan
miR-10527	ENSG00000257923	8m	TargetScan
miR-10527	ENSG00000258405	8m	TargetScan
miR-10527	ENSG00000258429	8m	TargetScan
miR-10527	ENSG00000258869	8m	TargetScan
miR-10527	ENSG00000259363	8m	TargetScan
miR-10527	ENSG00000260230	8m	TargetScan
miR-10527	ENSG00000261794	8m	TargetScan
miR-10527	ENSG00000263513	8m	TargetScan
miR-10527	ENSG00000272195	8m	TargetScan
miR-10527	ENSG00000272325	8m	TargetScan
miR-10527	ENSG00000272617	8m	TargetScan
miR-10527	ENSG00000273079	8m	TargetScan
miR-10527	ENSG00000274944	8m	TargetScan
miR-10527	ENSG00000278828	8m	TargetScan
miR-10527	ENSG00000285976	8m	TargetScan
miR-10527	ENSG00000286220	8m	TargetScan
miR-1244	ENSG00000000003	8m	TargetScan

miR-1244	ENSG00000003402	8m	TargetScan
miR-1244	ENSG00000005812	8m	TargetScan
miR-1244	ENSG00000006007	8m	TargetScan
miR-1244	ENSG00000007174	8m	TargetScan
miR-1244	ENSG00000009694	8m	TargetScan
miR-1244	ENSG00000010244	8m	TargetScan
miR-1244	ENSG00000011405	8m	TargetScan
miR-1244	ENSG00000014824	8m	TargetScan
miR-1244	ENSG00000023734	8m	TargetScan
miR-1244	ENSG00000024048	8m	TargetScan
miR-1244	ENSG00000025039	8m	TargetScan
miR-1244	ENSG00000025423	8m	TargetScan
miR-1244	ENSG00000029639	8m	TargetScan
miR-1244	ENSG00000030110	8m	TargetScan
miR-1244	ENSG00000031823	8m	TargetScan
miR-1244	ENSG00000033800	8m	TargetScan
miR-1244	ENSG00000037280	8m	TargetScan
miR-1244	ENSG00000038210	8m	TargetScan
miR-1244	ENSG00000044459	8m	TargetScan
miR-1244	ENSG00000048052	8m	TargetScan
miR-1244	ENSG00000052802	8m	TargetScan
miR-1244	ENSG00000053108	8m	TargetScan
miR-1244	ENSG00000055332	8m	TargetScan
miR-1244	ENSG00000056291	8m	TargetScan
miR-1244	ENSG00000057019	8m	TargetScan
miR-1244	ENSG00000057294	8m	TargetScan
miR-1244	ENSG00000059728	8m	TargetScan
miR-1244	ENSG00000060140	8m	TargetScan
miR-1244	ENSG00000060237	8m	TargetScan
miR-1244	ENSG00000061676	8m	TargetScan
miR-1244	ENSG00000066629	8m	TargetScan

miR-1244	ENSG00000067208	8m	TargetScan
miR-1244	ENSG00000067560	8m	TargetScan
miR-1244	ENSG00000068394	8m	TargetScan
miR-1244	ENSG00000069493	8m	TargetScan
miR-1244	ENSG00000069869	8m	TargetScan
miR-1244	ENSG00000070214	8m	TargetScan
miR-1244	ENSG00000072042	8m	TargetScan
miR-1244	ENSG00000072121	8m	TargetScan
miR-1244	ENSG00000072201	8m	TargetScan
miR-1244	ENSG00000072657	8m	TargetScan
miR-1244	ENSG00000073008	8m	TargetScan
miR-1244	ENSG00000073711	8m	TargetScan
miR-1244	ENSG00000075223	8m	TargetScan
miR-1244	ENSG00000076554	8m	TargetScan
miR-1244	ENSG00000078018	8m	TargetScan
miR-1244	ENSG00000078237	8m	TargetScan
miR-1244	ENSG00000078596	8m	TargetScan
miR-1244	ENSG00000081386	8m	TargetScan
miR-1244	ENSG00000082153	8m	TargetScan
miR-1244	ENSG00000083067	8m	TargetScan
miR-1244	ENSG00000083099	8m	TargetScan
miR-1244	ENSG00000083223	8m	TargetScan
miR-1244	ENSG00000084652	8m	TargetScan
miR-1244	ENSG00000086717	8m	TargetScan
miR-1244	ENSG00000087128	8m	TargetScan
miR-1244	ENSG00000089041	8m	TargetScan
miR-1244	ENSG00000089775	8m	TargetScan
miR-1244	ENSG00000090989	8m	TargetScan
miR-1244	ENSG00000091157	8m	TargetScan
miR-1244	ENSG00000099139	8m	TargetScan
miR-1244	ENSG00000099715	8m	TargetScan

miR-1244	ENSG00000100077	8m	TargetScan
miR-1244	ENSG00000100478	8m	TargetScan
miR-1244	ENSG00000100505	8m	TargetScan
miR-1244	ENSG00000100528	8m	TargetScan
miR-1244	ENSG00000100934	8m	TargetScan
miR-1244	ENSG00000101166	8m	TargetScan
miR-1244	ENSG00000101265	8m	TargetScan
miR-1244	ENSG00000101557	8m	TargetScan
miR-1244	ENSG00000101958	8m	TargetScan
miR-1244	ENSG00000102158	8m	TargetScan
miR-1244	ENSG00000102218	8m	TargetScan
miR-1244	ENSG00000102290	8m	TargetScan
miR-1244	ENSG00000102393	8m	TargetScan
miR-1244	ENSG00000102471	8m	TargetScan
miR-1244	ENSG00000102780	8m	TargetScan
miR-1244	ENSG00000102804	8m	TargetScan
miR-1244	ENSG00000102910	8m	TargetScan
miR-1244	ENSG00000103061	8m	TargetScan
miR-1244	ENSG00000103404	8m	TargetScan
miR-1244	ENSG00000104218	8m	TargetScan
miR-1244	ENSG00000104231	8m	TargetScan
miR-1244	ENSG00000104327	8m	TargetScan
miR-1244	ENSG00000105492	8m	TargetScan
miR-1244	ENSG00000105750	8m	TargetScan
miR-1244	ENSG00000106261	8m	TargetScan
miR-1244	ENSG00000106299	8m	TargetScan
miR-1244	ENSG00000106328	8m	TargetScan
miR-1244	ENSG00000106366	8m	TargetScan
miR-1244	ENSG00000106443	8m	TargetScan
miR-1244	ENSG00000106952	8m	TargetScan
miR-1244	ENSG00000108219	8m	TargetScan

miR-1244	ENSG00000109220	8m	TargetScan
miR-1244	ENSG00000109436	8m	TargetScan
miR-1244	ENSG00000109572	8m	TargetScan
miR-1244	ENSG00000109911	8m	TargetScan
miR-1244	ENSG00000109917	8m	TargetScan
miR-1244	ENSG00000110048	8m	TargetScan
miR-1244	ENSG00000110344	8m	TargetScan
miR-1244	ENSG00000110395	8m	TargetScan
miR-1244	ENSG00000110987	8m	TargetScan
miR-1244	ENSG00000111145	8m	TargetScan
miR-1244	ENSG00000111799	8m	TargetScan
miR-1244	ENSG00000111911	8m	TargetScan
miR-1244	ENSG00000112234	8m	TargetScan
miR-1244	ENSG00000112242	8m	TargetScan
miR-1244	ENSG00000112309	8m	TargetScan
miR-1244	ENSG00000112339	8m	TargetScan
miR-1244	ENSG00000112379	8m	TargetScan
miR-1244	ENSG00000112499	8m	TargetScan
miR-1244	ENSG00000113361	8m	TargetScan
miR-1244	ENSG00000113600	8m	TargetScan
miR-1244	ENSG00000113615	8m	TargetScan
miR-1244	ENSG00000113851	8m	TargetScan
miR-1244	ENSG00000115183	8m	TargetScan
miR-1244	ENSG00000115524	8m	TargetScan
miR-1244	ENSG00000115540	8m	TargetScan
miR-1244	ENSG00000115760	8m	TargetScan
miR-1244	ENSG00000116544	8m	TargetScan
miR-1244	ENSG00000117151	8m	TargetScan
miR-1244	ENSG00000117724	8m	TargetScan
miR-1244	ENSG00000117758	8m	TargetScan
miR-1244	ENSG00000118217	8m	TargetScan

miR-1244	ENSG00000118495	8m	TargetScan
miR-1244	ENSG00000118514	8m	TargetScan
miR-1244	ENSG00000119203	8m	TargetScan
miR-1244	ENSG00000119328	8m	TargetScan
miR-1244	ENSG00000119673	8m	TargetScan
miR-1244	ENSG00000119820	8m	TargetScan
miR-1244	ENSG00000119973	8m	TargetScan
miR-1244	ENSG00000120907	8m	TargetScan
miR-1244	ENSG00000120925	8m	TargetScan
miR-1244	ENSG00000120992	8m	TargetScan
miR-1244	ENSG00000122435	8m	TargetScan
miR-1244	ENSG00000122952	8m	TargetScan
miR-1244	ENSG00000123307	8m	TargetScan
miR-1244	ENSG00000123594	8m	TargetScan
miR-1244	ENSG00000124374	8m	TargetScan
miR-1244	ENSG00000124532	8m	TargetScan
miR-1244	ENSG00000124596	8m	TargetScan
miR-1244	ENSG00000124613	8m	TargetScan
miR-1244	ENSG00000124783	8m	TargetScan
miR-1244	ENSG00000125285	8m	TargetScan
miR-1244	ENSG00000125457	8m	TargetScan
miR-1244	ENSG00000125629	8m	TargetScan
miR-1244	ENSG00000126070	8m	TargetScan
miR-1244	ENSG00000126266	8m	TargetScan
miR-1244	ENSG00000126803	8m	TargetScan
miR-1244	ENSG00000127920	8m	TargetScan
miR-1244	ENSG00000128159	8m	TargetScan
miR-1244	ENSG00000128617	8m	TargetScan
miR-1244	ENSG00000129691	8m	TargetScan
miR-1244	ENSG00000129810	8m	TargetScan
miR-1244	ENSG00000130119	8m	TargetScan

miR-1244	ENSG00000130695	8m	TargetScan
miR-1244	ENSG00000131375	8m	TargetScan
miR-1244	ENSG00000132122	8m	TargetScan
miR-1244	ENSG00000132254	8m	TargetScan
miR-1244	ENSG00000132300	8m	TargetScan
miR-1244	ENSG00000132554	8m	TargetScan
miR-1244	ENSG00000132964	8m	TargetScan
miR-1244	ENSG00000132975	8m	TargetScan
miR-1244	ENSG00000133083	8m	TargetScan
miR-1244	ENSG00000134153	8m	TargetScan
miR-1244	ENSG00000134242	8m	TargetScan
miR-1244	ENSG00000134248	8m	TargetScan
miR-1244	ENSG00000134294	8m	TargetScan
miR-1244	ENSG00000134444	8m	TargetScan
miR-1244	ENSG00000134970	8m	TargetScan
miR-1244	ENSG00000134987	8m	TargetScan
miR-1244	ENSG00000135205	8m	TargetScan
miR-1244	ENSG00000135299	8m	TargetScan
miR-1244	ENSG00000135387	8m	TargetScan
miR-1244	ENSG00000135541	8m	TargetScan
miR-1244	ENSG00000135899	8m	TargetScan
miR-1244	ENSG00000136021	8m	TargetScan
miR-1244	ENSG00000136144	8m	TargetScan
miR-1244	ENSG00000136783	8m	TargetScan
miR-1244	ENSG00000137033	8m	TargetScan
miR-1244	ENSG00000137501	8m	TargetScan
miR-1244	ENSG00000137504	8m	TargetScan
miR-1244	ENSG00000137727	8m	TargetScan
miR-1244	ENSG00000137760	8m	TargetScan
miR-1244	ENSG00000137812	8m	TargetScan
miR-1244	ENSG00000138081	8m	TargetScan

miR-1244	ENSG00000138185	8m	TargetScan
miR-1244	ENSG00000138286	8m	TargetScan
miR-1244	ENSG00000138347	8m	TargetScan
miR-1244	ENSG00000138653	8m	TargetScan
miR-1244	ENSG00000138670	8m	TargetScan
miR-1244	ENSG00000138767	8m	TargetScan
miR-1244	ENSG00000138769	8m	TargetScan
miR-1244	ENSG00000138772	8m	TargetScan
miR-1244	ENSG00000139133	8m	TargetScan
miR-1244	ENSG00000139278	8m	TargetScan
miR-1244	ENSG00000139291	8m	TargetScan
miR-1244	ENSG00000139971	8m	TargetScan
miR-1244	ENSG00000140319	8m	TargetScan
miR-1244	ENSG00000140832	8m	TargetScan
miR-1244	ENSG00000140987	8m	TargetScan
miR-1244	ENSG00000141027	8m	TargetScan
miR-1244	ENSG00000141404	8m	TargetScan
miR-1244	ENSG00000141627	8m	TargetScan
miR-1244	ENSG00000141639	8m	TargetScan
miR-1244	ENSG00000142405	8m	TargetScan
miR-1244	ENSG00000142599	8m	TargetScan
miR-1244	ENSG00000143179	8m	TargetScan
miR-1244	ENSG00000143228	8m	TargetScan
miR-1244	ENSG00000143322	8m	TargetScan
miR-1244	ENSG00000144278	8m	TargetScan
miR-1244	ENSG00000144791	8m	TargetScan
miR-1244	ENSG00000144848	8m	TargetScan
miR-1244	ENSG00000145147	8m	TargetScan
miR-1244	ENSG00000145335	8m	TargetScan
miR-1244	ENSG00000145431	8m	TargetScan
miR-1244	ENSG00000145451	8m	TargetScan

miR-1244	ENSG00000145687	8m	TargetScan
miR-1244	ENSG00000146587	8m	TargetScan
miR-1244	ENSG00000146592	8m	TargetScan
miR-1244	ENSG00000146755	8m	TargetScan
miR-1244	ENSG00000147010	8m	TargetScan
miR-1244	ENSG00000147036	8m	TargetScan
miR-1244	ENSG00000147364	8m	TargetScan
miR-1244	ENSG00000147852	8m	TargetScan
miR-1244	ENSG00000147853	8m	TargetScan
miR-1244	ENSG00000147996	8m	TargetScan
miR-1244	ENSG00000148219	8m	TargetScan
miR-1244	ENSG00000148229	8m	TargetScan
miR-1244	ENSG00000148248	8m	TargetScan
miR-1244	ENSG00000148498	8m	TargetScan
miR-1244	ENSG00000148734	8m	TargetScan
miR-1244	ENSG00000148842	8m	TargetScan
miR-1244	ENSG00000148942	8m	TargetScan
miR-1244	ENSG00000149084	8m	TargetScan
miR-1244	ENSG00000149131	8m	TargetScan
miR-1244	ENSG00000151962	8m	TargetScan
miR-1244	ENSG00000152061	8m	TargetScan
miR-1244	ENSG00000152207	8m	TargetScan
miR-1244	ENSG00000152256	8m	TargetScan
miR-1244	ENSG00000152578	8m	TargetScan
miR-1244	ENSG00000152582	8m	TargetScan
miR-1244	ENSG00000152749	8m	TargetScan
miR-1244	ENSG00000152763	8m	TargetScan
miR-1244	ENSG00000153721	8m	TargetScan
miR-1244	ENSG00000153989	8m	TargetScan
miR-1244	ENSG00000154188	8m	TargetScan
miR-1244	ENSG00000154310	8m	TargetScan

miR-1244	ENSG00000154518	8m	TargetScan
miR-1244	ENSG00000154642	8m	TargetScan
miR-1244	ENSG00000154736	8m	TargetScan
miR-1244	ENSG00000155792	8m	TargetScan
miR-1244	ENSG00000155959	8m	TargetScan
miR-1244	ENSG00000156136	8m	TargetScan
miR-1244	ENSG00000156140	8m	TargetScan
miR-1244	ENSG00000156398	8m	TargetScan
miR-1244	ENSG00000156531	8m	TargetScan
miR-1244	ENSG00000156675	8m	TargetScan
miR-1244	ENSG00000157214	8m	TargetScan
miR-1244	ENSG00000157680	8m	TargetScan
miR-1244	ENSG00000157890	8m	TargetScan
miR-1244	ENSG00000158201	8m	TargetScan
miR-1244	ENSG00000158528	8m	TargetScan
miR-1244	ENSG00000158813	8m	TargetScan
miR-1244	ENSG00000161649	8m	TargetScan
miR-1244	ENSG00000161654	8m	TargetScan
miR-1244	ENSG00000161921	8m	TargetScan
miR-1244	ENSG00000162129	8m	TargetScan
miR-1244	ENSG00000162241	8m	TargetScan
miR-1244	ENSG00000162441	8m	TargetScan
miR-1244	ENSG00000162599	8m	TargetScan
miR-1244	ENSG00000162618	8m	TargetScan
miR-1244	ENSG00000162706	8m	TargetScan
miR-1244	ENSG00000162814	8m	TargetScan
miR-1244	ENSG00000162946	8m	TargetScan
miR-1244	ENSG00000163041	8m	TargetScan
miR-1244	ENSG00000163154	8m	TargetScan
miR-1244	ENSG00000163288	8m	TargetScan
miR-1244	ENSG00000163291	8m	TargetScan

miR-1244	ENSG00000163444	8m	TargetScan
miR-1244	ENSG00000163596	8m	TargetScan
miR-1244	ENSG00000163673	8m	TargetScan
miR-1244	ENSG00000163803	8m	TargetScan
miR-1244	ENSG00000164093	8m	TargetScan
miR-1244	ENSG00000164114	8m	TargetScan
miR-1244	ENSG00000164125	8m	TargetScan
miR-1244	ENSG00000164168	8m	TargetScan
miR-1244	ENSG00000164172	8m	TargetScan
miR-1244	ENSG00000164188	8m	TargetScan
miR-1244	ENSG00000164252	8m	TargetScan
miR-1244	ENSG00000164342	8m	TargetScan
miR-1244	ENSG00000164764	8m	TargetScan
miR-1244	ENSG00000164815	8m	TargetScan
miR-1244	ENSG00000164867	8m	TargetScan
miR-1244	ENSG00000164902	8m	TargetScan
miR-1244	ENSG00000165283	8m	TargetScan
miR-1244	ENSG00000165309	8m	TargetScan
miR-1244	ENSG00000165338	8m	TargetScan
miR-1244	ENSG00000165474	8m	TargetScan
miR-1244	ENSG00000165506	8m	TargetScan
miR-1244	ENSG00000165732	8m	TargetScan
miR-1244	ENSG00000165775	8m	TargetScan
miR-1244	ENSG00000165997	8m	TargetScan
miR-1244	ENSG00000166037	8m	TargetScan
miR-1244	ENSG00000166148	8m	TargetScan
miR-1244	ENSG00000166398	8m	TargetScan
miR-1244	ENSG00000166446	8m	TargetScan
miR-1244	ENSG00000166454	8m	TargetScan
miR-1244	ENSG00000166479	8m	TargetScan
miR-1244	ENSG00000166575	8m	TargetScan

miR-1244	ENSG00000166845	8m	TargetScan
miR-1244	ENSG00000167005	8m	TargetScan
miR-1244	ENSG00000167191	8m	TargetScan
miR-1244	ENSG00000167202	8m	TargetScan
miR-1244	ENSG00000167384	8m	TargetScan
miR-1244	ENSG00000167526	8m	TargetScan
miR-1244	ENSG00000167548	8m	TargetScan
miR-1244	ENSG00000167613	8m	TargetScan
miR-1244	ENSG00000168303	8m	TargetScan
miR-1244	ENSG00000168453	8m	TargetScan
miR-1244	ENSG00000168610	8m	TargetScan
miR-1244	ENSG00000168939	8m	TargetScan
miR-1244	ENSG00000168939	8m	TargetScan
miR-1244	ENSG00000168944	8m	TargetScan
miR-1244	ENSG00000169122	8m	TargetScan
miR-1244	ENSG00000169504	8m	TargetScan
miR-1244	ENSG00000169860	8m	TargetScan
miR-1244	ENSG00000169967	8m	TargetScan
miR-1244	ENSG00000169991	8m	TargetScan
miR-1244	ENSG00000170191	8m	TargetScan
miR-1244	ENSG00000170312	8m	TargetScan
miR-1244	ENSG00000170340	8m	TargetScan
miR-1244	ENSG00000170456	8m	TargetScan
miR-1244	ENSG00000170502	8m	TargetScan
miR-1244	ENSG00000170542	8m	TargetScan
miR-1244	ENSG00000170832	8m	TargetScan
miR-1244	ENSG00000171368	8m	TargetScan
miR-1244	ENSG00000171843	8m	TargetScan
miR-1244	ENSG00000171862	8m	TargetScan
miR-1244	ENSG00000172209	8m	TargetScan
miR-1244	ENSG00000172339	8m	TargetScan

miR-1244	ENSG00000172380	8m	TargetScan
miR-1244	ENSG00000172466	8m	TargetScan
miR-1244	ENSG00000172493	8m	TargetScan
miR-1244	ENSG00000172785	8m	TargetScan
miR-1244	ENSG00000172817	8m	TargetScan
miR-1244	ENSG00000173207	8m	TargetScan
miR-1244	ENSG00000173273	8m	TargetScan
miR-1244	ENSG00000173285	8m	TargetScan
miR-1244	ENSG00000173334	8m	TargetScan
miR-1244	ENSG00000173451	8m	TargetScan
miR-1244	ENSG00000173517	8m	TargetScan
miR-1244	ENSG00000173744	8m	TargetScan
miR-1244	ENSG00000173890	8m	TargetScan
miR-1244	ENSG00000174013	8m	TargetScan
miR-1244	ENSG00000174016	8m	TargetScan
miR-1244	ENSG00000174429	8m	TargetScan
miR-1244	ENSG00000175170	8m	TargetScan
miR-1244	ENSG00000175189	8m	TargetScan
miR-1244	ENSG00000175305	8m	TargetScan
miR-1244	ENSG00000175387	8m	TargetScan
miR-1244	ENSG00000175390	8m	TargetScan
miR-1244	ENSG00000175471	8m	TargetScan
miR-1244	ENSG00000175548	8m	TargetScan
miR-1244	ENSG00000176049	8m	TargetScan
miR-1244	ENSG00000176463	8m	TargetScan
miR-1244	ENSG00000177324	8m	TargetScan
miR-1244	ENSG00000177570	8m	TargetScan
miR-1244	ENSG00000177689	8m	TargetScan
miR-1244	ENSG00000178201	8m	TargetScan
miR-1244	ENSG00000178338	8m	TargetScan
miR-1244	ENSG00000178562	8m	TargetScan

miR-1244	ENSG00000178795	8m	TargetScan
miR-1244	ENSG00000179028	8m	TargetScan
miR-1244	ENSG00000179387	8m	TargetScan
miR-1244	ENSG00000179915	8m	TargetScan
miR-1244	ENSG00000180287	8m	TargetScan
miR-1244	ENSG00000181704	8m	TargetScan
miR-1244	ENSG00000181722	8m	TargetScan
miR-1244	ENSG00000182010	8m	TargetScan
miR-1244	ENSG00000182261	8m	TargetScan
miR-1244	ENSG00000182621	8m	TargetScan
miR-1244	ENSG00000182670	8m	TargetScan
miR-1244	ENSG00000182776	8m	TargetScan
miR-1244	ENSG00000182957	8m	TargetScan
miR-1244	ENSG00000183230	8m	TargetScan
miR-1244	ENSG00000183530	8m	TargetScan
miR-1244	ENSG00000183662	8m	TargetScan
miR-1244	ENSG00000183775	8m	TargetScan
miR-1244	ENSG00000183814	8m	TargetScan
miR-1244	ENSG00000183831	8m	TargetScan
miR-1244	ENSG00000184743	8m	TargetScan
miR-1244	ENSG00000184939	8m	TargetScan
miR-1244	ENSG00000185010	8m	TargetScan
miR-1244	ENSG00000185219	8m	TargetScan
miR-1244	ENSG00000185621	8m	TargetScan
miR-1244	ENSG00000185774	8m	TargetScan
miR-1244	ENSG00000185829	8m	TargetScan
miR-1244	ENSG00000186009	8m	TargetScan
miR-1244	ENSG00000186260	8m	TargetScan
miR-1244	ENSG00000186318	8m	TargetScan
miR-1244	ENSG00000186628	8m	TargetScan
miR-1244	ENSG00000186714	8m	TargetScan

miR-1244	ENSG00000186814	8m	TargetScan
miR-1244	ENSG00000187753	8m	TargetScan
miR-1244	ENSG00000187772	8m	TargetScan
miR-1244	ENSG00000187866	8m	TargetScan
miR-1244	ENSG00000188042	8m	TargetScan
miR-1244	ENSG00000188133	8m	TargetScan
miR-1244	ENSG00000188211	8m	TargetScan
miR-1244	ENSG00000188517	8m	TargetScan
miR-1244	ENSG00000188848	8m	TargetScan
miR-1244	ENSG00000196109	8m	TargetScan
miR-1244	ENSG00000196233	8m	TargetScan
miR-1244	ENSG00000196458	8m	TargetScan
miR-1244	ENSG00000196549	8m	TargetScan
miR-1244	ENSG00000196693	8m	TargetScan
miR-1244	ENSG00000196730	8m	TargetScan
miR-1244	ENSG00000197044	8m	TargetScan
miR-1244	ENSG00000197124	8m	TargetScan
miR-1244	ENSG00000197312	8m	TargetScan
miR-1244	ENSG00000197619	8m	TargetScan
miR-1244	ENSG00000197714	8m	TargetScan
miR-1244	ENSG00000197779	8m	TargetScan
miR-1244	ENSG00000197872	8m	TargetScan
miR-1244	ENSG00000197885	8m	TargetScan
miR-1244	ENSG00000198105	8m	TargetScan
miR-1244	ENSG00000198162	8m	TargetScan
miR-1244	ENSG00000198168	8m	TargetScan
miR-1244	ENSG00000198590	8m	TargetScan
miR-1244	ENSG00000198612	8m	TargetScan
miR-1244	ENSG00000198677	8m	TargetScan
miR-1244	ENSG00000198843	8m	TargetScan
miR-1244	ENSG00000198846	8m	TargetScan

miR-1244	ENSG00000198874	8m	TargetScan
miR-1244	ENSG00000198890	8m	TargetScan
miR-1244	ENSG00000204176	8m	TargetScan
miR-1244	ENSG00000204304	8m	TargetScan
miR-1244	ENSG00000204655	8m	TargetScan
miR-1244	ENSG00000204767	8m	TargetScan
miR-1244	ENSG00000205045	8m	TargetScan
miR-1244	ENSG00000205138	8m	TargetScan
miR-1244	ENSG00000205213	8m	TargetScan
miR-1244	ENSG00000206432	8m	TargetScan
miR-1244	ENSG00000206557	8m	TargetScan
miR-1244	ENSG00000213047	8m	TargetScan
miR-1244	ENSG00000214113	8m	TargetScan
miR-1244	ENSG00000222009	8m	TargetScan
miR-1244	ENSG00000224470	8m	TargetScan
miR-1244	ENSG00000225526	8m	TargetScan
miR-1244	ENSG00000228696	8m	TargetScan
miR-1244	ENSG00000230989	8m	TargetScan
miR-1244	ENSG00000239900	8m	TargetScan
miR-1244	ENSG00000244734	8m	TargetScan
miR-1244	ENSG00000246705	8m	TargetScan
miR-1244	ENSG00000249139	8m	TargetScan
miR-1244	ENSG00000254004	8m	TargetScan
miR-1244	ENSG00000256043	8m	TargetScan
miR-1244	ENSG00000256294	8m	TargetScan
miR-1244	ENSG00000258708	8m	TargetScan
miR-1244	ENSG00000267260	8m	TargetScan
miR-1244	ENSG00000269067	8m	TargetScan
miR-1244	ENSG00000270757	8m	TargetScan
miR-3654	ENSG00000011275	8m	TargetScan
miR-3654	ENSG00000011465	8m	TargetScan

miR-3654	ENSG00000013275	8m	TargetScan
miR-3654	ENSG00000025800	8m	TargetScan
miR-3654	ENSG00000031081	8m	TargetScan
miR-3654	ENSG00000046604	8m	TargetScan
miR-3654	ENSG00000047849	8m	TargetScan
miR-3654	ENSG00000057657	8m	TargetScan
miR-3654	ENSG00000064703	8m	TargetScan
miR-3654	ENSG00000066294	8m	TargetScan
miR-3654	ENSG00000067900	8m	TargetScan
miR-3654	ENSG00000068024	8m	TargetScan
miR-3654	ENSG00000068394	8m	TargetScan
miR-3654	ENSG00000072364	8m	TargetScan
miR-3654	ENSG00000072803	8m	TargetScan
miR-3654	ENSG00000073584	8m	TargetScan
miR-3654	ENSG00000074410	8m	TargetScan
miR-3654	ENSG00000078589	8m	TargetScan
miR-3654	ENSG00000081177	8m	TargetScan
miR-3654	ENSG00000081760	8m	TargetScan
miR-3654	ENSG00000081985	8m	TargetScan
miR-3654	ENSG00000082175	8m	TargetScan
miR-3654	ENSG00000083099	8m	TargetScan
miR-3654	ENSG00000085831	8m	TargetScan
miR-3654	ENSG00000088543	8m	TargetScan
miR-3654	ENSG00000089101	8m	TargetScan
miR-3654	ENSG00000091986	8m	TargetScan
miR-3654	ENSG00000092847	8m	TargetScan
miR-3654	ENSG00000100030	8m	TargetScan
miR-3654	ENSG00000100380	8m	TargetScan
miR-3654	ENSG00000100665	8m	TargetScan
miR-3654	ENSG00000100815	8m	TargetScan
miR-3654	ENSG00000101384	8m	TargetScan

miR-3654	ENSG00000101856	8m	TargetScan
miR-3654	ENSG00000102096	8m	TargetScan
miR-3654	ENSG00000102466	8m	TargetScan
miR-3654	ENSG00000102908	8m	TargetScan
miR-3654	ENSG00000103061	8m	TargetScan
miR-3654	ENSG00000103160	8m	TargetScan
miR-3654	ENSG00000103888	8m	TargetScan
miR-3654	ENSG00000104142	8m	TargetScan
miR-3654	ENSG00000104219	8m	TargetScan
miR-3654	ENSG00000104689	8m	TargetScan
miR-3654	ENSG00000104974	8m	TargetScan
miR-3654	ENSG00000105085	8m	TargetScan
miR-3654	ENSG00000105197	8m	TargetScan
miR-3654	ENSG00000105204	8m	TargetScan
miR-3654	ENSG00000106258	8m	TargetScan
miR-3654	ENSG00000106772	8m	TargetScan
miR-3654	ENSG00000107362	8m	TargetScan
miR-3654	ENSG00000107864	8m	TargetScan
miR-3654	ENSG00000107897	8m	TargetScan
miR-3654	ENSG00000108107	8m	TargetScan
miR-3654	ENSG00000108375	8m	TargetScan
miR-3654	ENSG00000109158	8m	TargetScan
miR-3654	ENSG00000109265	8m	TargetScan
miR-3654	ENSG00000109787	8m	TargetScan
miR-3654	ENSG00000110090	8m	TargetScan
miR-3654	ENSG00000110218	8m	TargetScan
miR-3654	ENSG00000111371	8m	TargetScan
miR-3654	ENSG00000111481	8m	TargetScan
miR-3654	ENSG00000111530	8m	TargetScan
miR-3654	ENSG00000111665	8m	TargetScan
miR-3654	ENSG00000112697	8m	TargetScan

miR-3654	ENSG00000113119	8m	TargetScan
miR-3654	ENSG00000113300	8m	TargetScan
miR-3654	ENSG00000113318	8m	TargetScan
miR-3654	ENSG00000113494	8m	TargetScan
miR-3654	ENSG00000113719	8m	TargetScan
miR-3654	ENSG00000113812	8m	TargetScan
miR-3654	ENSG00000114331	8m	TargetScan
miR-3654	ENSG00000114503	8m	TargetScan
miR-3654	ENSG00000114544	8m	TargetScan
miR-3654	ENSG00000115306	8m	TargetScan
miR-3654	ENSG00000115353	8m	TargetScan
miR-3654	ENSG00000115421	8m	TargetScan
miR-3654	ENSG00000115507	8m	TargetScan
miR-3654	ENSG00000115896	8m	TargetScan
miR-3654	ENSG00000115919	8m	TargetScan
miR-3654	ENSG00000115977	8m	TargetScan
miR-3654	ENSG00000117115	8m	TargetScan
miR-3654	ENSG00000117151	8m	TargetScan
miR-3654	ENSG00000117155	8m	TargetScan
miR-3654	ENSG00000117280	8m	TargetScan
miR-3654	ENSG00000117751	8m	TargetScan
miR-3654	ENSG00000118217	8m	TargetScan
miR-3654	ENSG00000118777	8m	TargetScan
miR-3654	ENSG00000119655	8m	TargetScan
miR-3654	ENSG00000119661	8m	TargetScan
miR-3654	ENSG00000119927	8m	TargetScan
miR-3654	ENSG00000120458	8m	TargetScan
miR-3654	ENSG00000120549	8m	TargetScan
miR-3654	ENSG00000121454	8m	TargetScan
miR-3654	ENSG00000121671	8m	TargetScan
miR-3654	ENSG00000121964	8m	TargetScan

miR-3654	ENSG00000122254	8m	TargetScan
miR-3654	ENSG00000122490	8m	TargetScan
miR-3654	ENSG00000122642	8m	TargetScan
miR-3654	ENSG00000122986	8m	TargetScan
miR-3654	ENSG00000123353	8m	TargetScan
miR-3654	ENSG00000123739	8m	TargetScan
miR-3654	ENSG00000124155	8m	TargetScan
miR-3654	ENSG00000124641	8m	TargetScan
miR-3654	ENSG00000125037	8m	TargetScan
miR-3654	ENSG00000125885	8m	TargetScan
miR-3654	ENSG00000127337	8m	TargetScan
miR-3654	ENSG00000128641	8m	TargetScan
miR-3654	ENSG00000128944	8m	TargetScan
miR-3654	ENSG00000130338	8m	TargetScan
miR-3654	ENSG00000130958	8m	TargetScan
miR-3654	ENSG00000132549	8m	TargetScan
miR-3654	ENSG00000132768	8m	TargetScan
miR-3654	ENSG00000132879	8m	TargetScan
miR-3654	ENSG00000132964	8m	TargetScan
miR-3654	ENSG00000133302	8m	TargetScan
miR-3654	ENSG00000133958	8m	TargetScan
miR-3654	ENSG00000134108	8m	TargetScan
miR-3654	ENSG00000134352	8m	TargetScan
miR-3654	ENSG00000134759	8m	TargetScan
miR-3654	ENSG00000134852	8m	TargetScan
miR-3654	ENSG00000135870	8m	TargetScan
miR-3654	ENSG00000135999	8m	TargetScan
miR-3654	ENSG00000136104	8m	TargetScan
miR-3654	ENSG00000136381	8m	TargetScan
miR-3654	ENSG00000136824	8m	TargetScan
miR-3654	ENSG00000137692	8m	TargetScan

miR-3654	ENSG00000137727	8m	TargetScan
miR-3654	ENSG00000137947	8m	TargetScan
miR-3654	ENSG00000138593	8m	TargetScan
miR-3654	ENSG00000138670	8m	TargetScan
miR-3654	ENSG00000139437	8m	TargetScan
miR-3654	ENSG00000139496	8m	TargetScan
miR-3654	ENSG00000140090	8m	TargetScan
miR-3654	ENSG00000140391	8m	TargetScan
miR-3654	ENSG00000140526	8m	TargetScan
miR-3654	ENSG00000141068	8m	TargetScan
miR-3654	ENSG00000141127	8m	TargetScan
miR-3654	ENSG00000141378	8m	TargetScan
miR-3654	ENSG00000141449	8m	TargetScan
miR-3654	ENSG00000143106	8m	TargetScan
miR-3654	ENSG00000143190	8m	TargetScan
miR-3654	ENSG00000143198	8m	TargetScan
miR-3654	ENSG00000143457	8m	TargetScan
miR-3654	ENSG00000143536	8m	TargetScan
miR-3654	ENSG00000143622	8m	TargetScan
miR-3654	ENSG00000143727	8m	TargetScan
miR-3654	ENSG00000144535	8m	TargetScan
miR-3654	ENSG00000144848	8m	TargetScan
miR-3654	ENSG00000145779	8m	TargetScan
miR-3654	ENSG00000145782	8m	TargetScan
miR-3654	ENSG00000145907	8m	TargetScan
miR-3654	ENSG00000146278	8m	TargetScan
miR-3654	ENSG00000146352	8m	TargetScan
miR-3654	ENSG00000146574	8m	TargetScan
miR-3654	ENSG00000146729	8m	TargetScan
miR-3654	ENSG00000146950	8m	TargetScan
miR-3654	ENSG00000147113	8m	TargetScan

miR-3654	ENSG00000147121	8m	TargetScan
miR-3654	ENSG00000147127	8m	TargetScan
miR-3654	ENSG00000147234	8m	TargetScan
miR-3654	ENSG00000147588	8m	TargetScan
miR-3654	ENSG00000148200	8m	TargetScan
miR-3654	ENSG00000148688	8m	TargetScan
miR-3654	ENSG00000149927	8m	TargetScan
miR-3654	ENSG00000149972	8m	TargetScan
miR-3654	ENSG00000151117	8m	TargetScan
miR-3654	ENSG00000152127	8m	TargetScan
miR-3654	ENSG00000152402	8m	TargetScan
miR-3654	ENSG00000152443	8m	TargetScan
miR-3654	ENSG00000153391	8m	TargetScan
miR-3654	ENSG00000153936	8m	TargetScan
miR-3654	ENSG00000153944	8m	TargetScan
miR-3654	ENSG00000154080	8m	TargetScan
miR-3654	ENSG00000154222	8m	TargetScan
miR-3654	ENSG00000154310	8m	TargetScan
miR-3654	ENSG00000155096	8m	TargetScan
miR-3654	ENSG00000155158	8m	TargetScan
miR-3654	ENSG00000156486	8m	TargetScan
miR-3654	ENSG00000157388	8m	TargetScan
miR-3654	ENSG00000158480	8m	TargetScan
miR-3654	ENSG00000159958	8m	TargetScan
miR-3654	ENSG00000160471	8m	TargetScan
miR-3654	ENSG00000162733	8m	TargetScan
miR-3654	ENSG00000163013	8m	TargetScan
miR-3654	ENSG00000163646	8m	TargetScan
miR-3654	ENSG00000163873	8m	TargetScan
miR-3654	ENSG00000163909	8m	TargetScan
miR-3654	ENSG00000164161	8m	TargetScan

miR-3654	ENSG00000164167	8m	TargetScan
miR-3654	ENSG00000164484	8m	TargetScan
miR-3654	ENSG00000164983	8m	TargetScan
miR-3654	ENSG00000165152	8m	TargetScan
miR-3654	ENSG00000165185	8m	TargetScan
miR-3654	ENSG00000165209	8m	TargetScan
miR-3654	ENSG00000165416	8m	TargetScan
miR-3654	ENSG00000165572	8m	TargetScan
miR-3654	ENSG00000165806	8m	TargetScan
miR-3654	ENSG00000166123	8m	TargetScan
miR-3654	ENSG00000166147	8m	TargetScan
miR-3654	ENSG00000166148	8m	TargetScan
miR-3654	ENSG00000166167	8m	TargetScan
miR-3654	ENSG00000166225	8m	TargetScan
miR-3654	ENSG00000167378	8m	TargetScan
miR-3654	ENSG00000167797	8m	TargetScan
miR-3654	ENSG00000167904	8m	TargetScan
miR-3654	ENSG00000168159	8m	TargetScan
miR-3654	ENSG00000168228	8m	TargetScan
miR-3654	ENSG00000169045	8m	TargetScan
miR-3654	ENSG00000169067	8m	TargetScan
miR-3654	ENSG00000170006	8m	TargetScan
miR-3654	ENSG00000170579	8m	TargetScan
miR-3654	ENSG00000171759	8m	TargetScan
miR-3654	ENSG00000172006	8m	TargetScan
miR-3654	ENSG00000172466	8m	TargetScan
miR-3654	ENSG00000172663	8m	TargetScan
miR-3654	ENSG00000172795	8m	TargetScan
miR-3654	ENSG00000172893	8m	TargetScan
miR-3654	ENSG00000172943	8m	TargetScan
miR-3654	ENSG00000173166	8m	TargetScan

miR-3654	ENSG00000173218	8m	TargetScan
miR-3654	ENSG00000173349	8m	TargetScan
miR-3654	ENSG00000173530	8m	TargetScan
miR-3654	ENSG00000173597	8m	TargetScan
miR-3654	ENSG00000174579	8m	TargetScan
miR-3654	ENSG00000174749	8m	TargetScan
miR-3654	ENSG00000174945	8m	TargetScan
miR-3654	ENSG00000174953	8m	TargetScan
miR-3654	ENSG00000175193	8m	TargetScan
miR-3654	ENSG00000175387	8m	TargetScan
miR-3654	ENSG00000175414	8m	TargetScan
miR-3654	ENSG00000175538	8m	TargetScan
miR-3654	ENSG00000175868	8m	TargetScan
miR-3654	ENSG00000176142	8m	TargetScan
miR-3654	ENSG00000176593	8m	TargetScan
miR-3654	ENSG00000176714	8m	TargetScan
miR-3654	ENSG00000177733	8m	TargetScan
miR-3654	ENSG00000178105	8m	TargetScan
miR-3654	ENSG00000178568	8m	TargetScan
miR-3654	ENSG00000180902	8m	TargetScan
miR-3654	ENSG00000180938	8m	TargetScan
miR-3654	ENSG00000181378	8m	TargetScan
miR-3654	ENSG00000181982	8m	TargetScan
miR-3654	ENSG00000182831	8m	TargetScan
miR-3654	ENSG00000183044	8m	TargetScan
miR-3654	ENSG00000183137	8m	TargetScan
miR-3654	ENSG00000183762	8m	TargetScan
miR-3654	ENSG00000184465	8m	TargetScan
miR-3654	ENSG00000186088	8m	TargetScan
miR-3654	ENSG00000186431	8m	TargetScan
miR-3654	ENSG00000186479	8m	TargetScan

miR-3654	ENSG00000186814	8m	TargetScan
miR-3654	ENSG00000187097	8m	TargetScan
miR-3654	ENSG00000187325	8m	TargetScan
miR-3654	ENSG00000188177	8m	TargetScan
miR-3654	ENSG00000188211	8m	TargetScan
miR-3654	ENSG00000188266	8m	TargetScan
miR-3654	ENSG00000188729	8m	TargetScan
miR-3654	ENSG00000188848	8m	TargetScan
miR-3654	ENSG00000189007	8m	TargetScan
miR-3654	ENSG00000196090	8m	TargetScan
miR-3654	ENSG00000196150	8m	TargetScan
miR-3654	ENSG00000196167	8m	TargetScan
miR-3654	ENSG00000196678	8m	TargetScan
miR-3654	ENSG00000196735	8m	TargetScan
miR-3654	ENSG00000196747	8m	TargetScan
miR-3654	ENSG00000196867	8m	TargetScan
miR-3654	ENSG00000196876	8m	TargetScan
miR-3654	ENSG00000196914	8m	TargetScan
miR-3654	ENSG00000197157	8m	TargetScan
miR-3654	ENSG00000197891	8m	TargetScan
miR-3654	ENSG00000198252	8m	TargetScan
miR-3654	ENSG00000198455	8m	TargetScan
miR-3654	ENSG00000198612	8m	TargetScan
miR-3654	ENSG00000203667	8m	TargetScan
miR-3654	ENSG00000204070	8m	TargetScan
miR-3654	ENSG00000204323	8m	TargetScan
miR-3654	ENSG00000204978	8m	TargetScan
miR-3654	ENSG00000205730	8m	TargetScan
miR-3654	ENSG00000205927	8m	TargetScan
miR-3654	ENSG00000213064	8m	TargetScan
miR-3654	ENSG00000213995	8m	TargetScan

miR-3654	ENSG00000237541	8m	TargetScan
miR-3654	ENSG00000241839	8m	TargetScan
miR-3654	ENSG00000250305	8m	TargetScan
miR-3654	ENSG00000254726	8m	TargetScan
miR-3654	ENSG00000255552	8m	TargetScan
miR-3654	ENSG00000269155	8m	TargetScan
miR-4426	ENSG00000000457	8m	TargetScan
miR-4426	ENSG00000002587	8m	TargetScan
miR-4426	ENSG00000003402	8m	TargetScan
miR-4426	ENSG00000005238	8m	TargetScan
miR-4426	ENSG00000005436	8m	TargetScan
miR-4426	ENSG00000006747	8m	TargetScan
miR-4426	ENSG00000008196	8m	TargetScan
miR-4426	ENSG00000010539	8m	TargetScan
miR-4426	ENSG00000011405	8m	TargetScan
miR-4426	ENSG00000012061	8m	TargetScan
miR-4426	ENSG00000013293	8m	TargetScan
miR-4426	ENSG00000018869	8m	TargetScan
miR-4426	ENSG00000019505	8m	TargetScan
miR-4426	ENSG00000020256	8m	TargetScan
miR-4426	ENSG00000020426	8m	TargetScan
miR-4426	ENSG00000024048	8m	TargetScan
miR-4426	ENSG00000026950	8m	TargetScan
miR-4426	ENSG00000029993	8m	TargetScan
miR-4426	ENSG00000034677	8m	TargetScan
miR-4426	ENSG00000036828	8m	TargetScan
miR-4426	ENSG00000037637	8m	TargetScan
miR-4426	ENSG00000039139	8m	TargetScan
miR-4426	ENSG00000040341	8m	TargetScan
miR-4426	ENSG00000040731	8m	TargetScan
miR-4426	ENSG00000042832	8m	TargetScan

miR-4426	ENSG00000044574	8m	TargetScan
miR-4426	ENSG00000047597	8m	TargetScan
miR-4426	ENSG00000047662	8m	TargetScan
miR-4426	ENSG00000048471	8m	TargetScan
miR-4426	ENSG00000049246	8m	TargetScan
miR-4426	ENSG00000049247	8m	TargetScan
miR-4426	ENSG00000049618	8m	TargetScan
miR-4426	ENSG00000050344	8m	TargetScan
miR-4426	ENSG00000050748	8m	TargetScan
miR-4426	ENSG00000056097	8m	TargetScan
miR-4426	ENSG00000057019	8m	TargetScan
miR-4426	ENSG00000061455	8m	TargetScan
miR-4426	ENSG00000063127	8m	TargetScan
miR-4426	ENSG00000064042	8m	TargetScan
miR-4426	ENSG00000064201	8m	TargetScan
miR-4426	ENSG00000065427	8m	TargetScan
miR-4426	ENSG00000065613	8m	TargetScan
miR-4426	ENSG00000065923	8m	TargetScan
miR-4426	ENSG00000066427	8m	TargetScan
miR-4426	ENSG00000066697	8m	TargetScan
miR-4426	ENSG00000066933	8m	TargetScan
miR-4426	ENSG00000067191	8m	TargetScan
miR-4426	ENSG00000068796	8m	TargetScan
miR-4426	ENSG00000069345	8m	TargetScan
miR-4426	ENSG00000069431	8m	TargetScan
miR-4426	ENSG00000069493	8m	TargetScan
miR-4426	ENSG00000071054	8m	TargetScan
miR-4426	ENSG00000071189	8m	TargetScan
miR-4426	ENSG00000072315	8m	TargetScan
miR-4426	ENSG00000072422	8m	TargetScan
miR-4426	ENSG00000073614	8m	TargetScan

miR-4426	ENSG00000074054	8m	TargetScan
miR-4426	ENSG00000075035	8m	TargetScan
miR-4426	ENSG00000075426	8m	TargetScan
miR-4426	ENSG00000076053	8m	TargetScan
miR-4426	ENSG00000077274	8m	TargetScan
miR-4426	ENSG00000077616	8m	TargetScan
miR-4426	ENSG00000078142	8m	TargetScan
miR-4426	ENSG00000078900	8m	TargetScan
miR-4426	ENSG00000079931	8m	TargetScan
miR-4426	ENSG00000079950	8m	TargetScan
miR-4426	ENSG00000080224	8m	TargetScan
miR-4426	ENSG00000080815	8m	TargetScan
miR-4426	ENSG00000081026	8m	TargetScan
miR-4426	ENSG00000082068	8m	TargetScan
miR-4426	ENSG00000082438	8m	TargetScan
miR-4426	ENSG00000082701	8m	TargetScan
miR-4426	ENSG00000082805	8m	TargetScan
miR-4426	ENSG00000083642	8m	TargetScan
miR-4426	ENSG00000087266	8m	TargetScan
miR-4426	ENSG00000089006	8m	TargetScan
miR-4426	ENSG00000089057	8m	TargetScan
miR-4426	ENSG00000090376	8m	TargetScan
miR-4426	ENSG00000091831	8m	TargetScan
miR-4426	ENSG00000093217	8m	TargetScan
miR-4426	ENSG00000094880	8m	TargetScan
miR-4426	ENSG00000095637	8m	TargetScan
miR-4426	ENSG00000097046	8m	TargetScan
miR-4426	ENSG00000099219	8m	TargetScan
miR-4426	ENSG00000099250	8m	TargetScan
miR-4426	ENSG00000100056	8m	TargetScan
miR-4426	ENSG00000100201	8m	TargetScan

miR-4426	ENSG00000100216	8m	TargetScan
miR-4426	ENSG00000100219	8m	TargetScan
miR-4426	ENSG00000100350	8m	TargetScan
miR-4426	ENSG00000100364	8m	TargetScan
miR-4426	ENSG00000100461	8m	TargetScan
miR-4426	ENSG00000100596	8m	TargetScan
miR-4426	ENSG00000100815	8m	TargetScan
miR-4426	ENSG00000101115	8m	TargetScan
miR-4426	ENSG00000101190	8m	TargetScan
miR-4426	ENSG00000101236	8m	TargetScan
miR-4426	ENSG00000101546	8m	TargetScan
miR-4426	ENSG00000101751	8m	TargetScan
miR-4426	ENSG00000101916	8m	TargetScan
miR-4426	ENSG00000101935	8m	TargetScan
miR-4426	ENSG00000102309	8m	TargetScan
miR-4426	ENSG00000102471	8m	TargetScan
miR-4426	ENSG00000103018	8m	TargetScan
miR-4426	ENSG00000103047	8m	TargetScan
miR-4426	ENSG00000103404	8m	TargetScan
miR-4426	ENSG00000103489	8m	TargetScan
miR-4426	ENSG00000103550	8m	TargetScan
miR-4426	ENSG00000104081	8m	TargetScan
miR-4426	ENSG00000104290	8m	TargetScan
miR-4426	ENSG00000104325	8m	TargetScan
miR-4426	ENSG00000104643	8m	TargetScan
miR-4426	ENSG00000104825	8m	TargetScan
miR-4426	ENSG00000105122	8m	TargetScan
miR-4426	ENSG00000105983	8m	TargetScan
miR-4426	ENSG00000106049	8m	TargetScan
miR-4426	ENSG00000106258	8m	TargetScan
miR-4426	ENSG00000106304	8m	TargetScan

miR-4426	ENSG00000106443	8m	TargetScan
miR-4426	ENSG00000106460	8m	TargetScan
miR-4426	ENSG00000106477	8m	TargetScan
miR-4426	ENSG00000106479	8m	TargetScan
miR-4426	ENSG00000106665	8m	TargetScan
miR-4426	ENSG00000106714	8m	TargetScan
miR-4426	ENSG00000107738	8m	TargetScan
miR-4426	ENSG00000107771	8m	TargetScan
miR-4426	ENSG00000107789	8m	TargetScan
miR-4426	ENSG00000107864	8m	TargetScan
miR-4426	ENSG00000108064	8m	TargetScan
miR-4426	ENSG00000108219	8m	TargetScan
miR-4426	ENSG00000108239	8m	TargetScan
miR-4426	ENSG00000108379	8m	TargetScan
miR-4426	ENSG00000108702	8m	TargetScan
miR-4426	ENSG00000109189	8m	TargetScan
miR-4426	ENSG00000109265	8m	TargetScan
miR-4426	ENSG00000109738	8m	TargetScan
miR-4426	ENSG00000109861	8m	TargetScan
miR-4426	ENSG00000110395	8m	TargetScan
miR-4426	ENSG00000110427	8m	TargetScan
miR-4426	ENSG00000110756	8m	TargetScan
miR-4426	ENSG00000110777	8m	TargetScan
miR-4426	ENSG00000110917	8m	TargetScan
miR-4426	ENSG00000111203	8m	TargetScan
miR-4426	ENSG00000111335	8m	TargetScan
miR-4426	ENSG00000111713	8m	TargetScan
miR-4426	ENSG00000111737	8m	TargetScan
miR-4426	ENSG00000111785	8m	TargetScan
miR-4426	ENSG00000111817	8m	TargetScan
miR-4426	ENSG00000111837	8m	TargetScan

miR-4426	ENSG00000111859	8m	TargetScan
miR-4426	ENSG00000111877	8m	TargetScan
miR-4426	ENSG00000111913	8m	TargetScan
miR-4426	ENSG00000112038	8m	TargetScan
miR-4426	ENSG00000112494	8m	TargetScan
miR-4426	ENSG00000112619	8m	TargetScan
miR-4426	ENSG00000112852	8m	TargetScan
miR-4426	ENSG00000112877	8m	TargetScan
miR-4426	ENSG00000113384	8m	TargetScan
miR-4426	ENSG00000113389	8m	TargetScan
miR-4426	ENSG00000113594	8m	TargetScan
miR-4426	ENSG00000113597	8m	TargetScan
miR-4426	ENSG00000113719	8m	TargetScan
miR-4426	ENSG00000113732	8m	TargetScan
miR-4426	ENSG00000113845	8m	TargetScan
miR-4426	ENSG00000114209	8m	TargetScan
miR-4426	ENSG00000114354	8m	TargetScan
miR-4426	ENSG00000114739	8m	TargetScan
miR-4426	ENSG00000114770	8m	TargetScan
miR-4426	ENSG00000114805	8m	TargetScan
miR-4426	ENSG00000114861	8m	TargetScan
miR-4426	ENSG00000115808	8m	TargetScan
miR-4426	ENSG00000115866	8m	TargetScan
miR-4426	ENSG00000115875	8m	TargetScan
miR-4426	ENSG00000115896	8m	TargetScan
miR-4426	ENSG00000115977	8m	TargetScan
miR-4426	ENSG00000116005	8m	TargetScan
miR-4426	ENSG00000116095	8m	TargetScan
miR-4426	ENSG00000116171	8m	TargetScan
miR-4426	ENSG00000116489	8m	TargetScan
miR-4426	ENSG00000116539	8m	TargetScan

miR-4426	ENSG00000116852	8m	TargetScan
miR-4426	ENSG00000117139	8m	TargetScan
miR-4426	ENSG00000117226	8m	TargetScan
miR-4426	ENSG00000117395	8m	TargetScan
miR-4426	ENSG00000117500	8m	TargetScan
miR-4426	ENSG00000117505	8m	TargetScan
miR-4426	ENSG00000117600	8m	TargetScan
miR-4426	ENSG00000118113	8m	TargetScan
miR-4426	ENSG00000118322	8m	TargetScan
miR-4426	ENSG00000118620	8m	TargetScan
miR-4426	ENSG00000118777	8m	TargetScan
miR-4426	ENSG00000119403	8m	TargetScan
miR-4426	ENSG00000119718	8m	TargetScan
miR-4426	ENSG00000119729	8m	TargetScan
miR-4426	ENSG00000119900	8m	TargetScan
miR-4426	ENSG00000120279	8m	TargetScan
miR-4426	ENSG00000120337	8m	TargetScan
miR-4426	ENSG00000120509	8m	TargetScan
miR-4426	ENSG00000120733	8m	TargetScan
miR-4426	ENSG00000120798	8m	TargetScan
miR-4426	ENSG00000120925	8m	TargetScan
miR-4426	ENSG00000121579	8m	TargetScan
miR-4426	ENSG00000121621	8m	TargetScan
miR-4426	ENSG00000121989	8m	TargetScan
miR-4426	ENSG00000122257	8m	TargetScan
miR-4426	ENSG00000122367	8m	TargetScan
miR-4426	ENSG00000122557	8m	TargetScan
miR-4426	ENSG00000122574	8m	TargetScan
miR-4426	ENSG00000122870	8m	TargetScan
miR-4426	ENSG00000123154	8m	TargetScan
miR-4426	ENSG00000123219	8m	TargetScan

miR-4426	ENSG00000123933	8m	TargetScan
miR-4426	ENSG00000124224	8m	TargetScan
miR-4426	ENSG00000124374	8m	TargetScan
miR-4426	ENSG00000124496	8m	TargetScan
miR-4426	ENSG00000124574	8m	TargetScan
miR-4426	ENSG00000124596	8m	TargetScan
miR-4426	ENSG00000125107	8m	TargetScan
miR-4426	ENSG00000125676	8m	TargetScan
miR-4426	ENSG00000125741	8m	TargetScan
miR-4426	ENSG00000125944	8m	TargetScan
miR-4426	ENSG00000126070	8m	TargetScan
miR-4426	ENSG00000126226	8m	TargetScan
miR-4426	ENSG00000126545	8m	TargetScan
miR-4426	ENSG00000126773	8m	TargetScan
miR-4426	ENSG00000127249	8m	TargetScan
miR-4426	ENSG00000127995	8m	TargetScan
miR-4426	ENSG00000128294	8m	TargetScan
miR-4426	ENSG00000128713	8m	TargetScan
miR-4426	ENSG00000128789	8m	TargetScan
miR-4426	ENSG00000128944	8m	TargetScan
miR-4426	ENSG00000129221	8m	TargetScan
miR-4426	ENSG00000129595	8m	TargetScan
miR-4426	ENSG00000130182	8m	TargetScan
miR-4426	ENSG00000130749	8m	TargetScan
miR-4426	ENSG00000131269	8m	TargetScan
miR-4426	ENSG00000131746	8m	TargetScan
miR-4426	ENSG00000131845	8m	TargetScan
miR-4426	ENSG00000132164	8m	TargetScan
miR-4426	ENSG00000132326	8m	TargetScan
miR-4426	ENSG00000132639	8m	TargetScan
miR-4426	ENSG00000132640	8m	TargetScan

miR-4426	ENSG00000133028	8m	TargetScan
miR-4426	ENSG00000133318	8m	TargetScan
miR-4426	ENSG00000133477	8m	TargetScan
miR-4426	ENSG00000133665	8m	TargetScan
miR-4426	ENSG00000133858	8m	TargetScan
miR-4426	ENSG00000134108	8m	TargetScan
miR-4426	ENSG00000134460	8m	TargetScan
miR-4426	ENSG00000134757	8m	TargetScan
miR-4426	ENSG00000134817	8m	TargetScan
miR-4426	ENSG00000134970	8m	TargetScan
miR-4426	ENSG00000135835	8m	TargetScan
miR-4426	ENSG00000135870	8m	TargetScan
miR-4426	ENSG00000135945	8m	TargetScan
miR-4426	ENSG00000136021	8m	TargetScan
miR-4426	ENSG00000136213	8m	TargetScan
miR-4426	ENSG00000136541	8m	TargetScan
miR-4426	ENSG00000136709	8m	TargetScan
miR-4426	ENSG00000136888	8m	TargetScan
miR-4426	ENSG00000136932	8m	TargetScan
miR-4426	ENSG00000137075	8m	TargetScan
miR-4426	ENSG00000137098	8m	TargetScan
miR-4426	ENSG00000137261	8m	TargetScan
miR-4426	ENSG00000137500	8m	TargetScan
miR-4426	ENSG00000137822	8m	TargetScan
miR-4426	ENSG00000137942	8m	TargetScan
miR-4426	ENSG00000138092	8m	TargetScan
miR-4426	ENSG00000138363	8m	TargetScan
miR-4426	ENSG00000138411	8m	TargetScan
miR-4426	ENSG00000138430	8m	TargetScan
miR-4426	ENSG00000138443	8m	TargetScan
miR-4426	ENSG00000138593	8m	TargetScan

miR-4426	ENSG00000138613	8m	TargetScan
miR-4426	ENSG00000138653	8m	TargetScan
miR-4426	ENSG00000138670	8m	TargetScan
miR-4426	ENSG00000138675	8m	TargetScan
miR-4426	ENSG00000138686	8m	TargetScan
miR-4426	ENSG00000138792	8m	TargetScan
miR-4426	ENSG00000138802	8m	TargetScan
miR-4426	ENSG00000139278	8m	TargetScan
miR-4426	ENSG00000139719	8m	TargetScan
miR-4426	ENSG00000140015	8m	TargetScan
miR-4426	ENSG00000140262	8m	TargetScan
miR-4426	ENSG00000140264	8m	TargetScan
miR-4426	ENSG00000140280	8m	TargetScan
miR-4426	ENSG00000140386	8m	TargetScan
miR-4426	ENSG00000140830	8m	TargetScan
miR-4426	ENSG00000140992	8m	TargetScan
miR-4426	ENSG00000141232	8m	TargetScan
miR-4426	ENSG00000141401	8m	TargetScan
miR-4426	ENSG00000141441	8m	TargetScan
miR-4426	ENSG00000141448	8m	TargetScan
miR-4426	ENSG00000141560	8m	TargetScan
miR-4426	ENSG00000141644	8m	TargetScan
miR-4426	ENSG00000141965	8m	TargetScan
miR-4426	ENSG00000142405	8m	TargetScan
miR-4426	ENSG00000142538	8m	TargetScan
miR-4426	ENSG00000143498	8m	TargetScan
miR-4426	ENSG00000143869	8m	TargetScan
miR-4426	ENSG00000144136	8m	TargetScan
miR-4426	ENSG00000144401	8m	TargetScan
miR-4426	ENSG00000144554	8m	TargetScan
miR-4426	ENSG00000144645	8m	TargetScan

miR-4426	ENSG00000144749	8m	TargetScan
miR-4426	ENSG00000144791	8m	TargetScan
miR-4426	ENSG00000144840	8m	TargetScan
miR-4426	ENSG00000144909	8m	TargetScan
miR-4426	ENSG00000145029	8m	TargetScan
miR-4426	ENSG00000145388	8m	TargetScan
miR-4426	ENSG00000145414	8m	TargetScan
miR-4426	ENSG00000145592	8m	TargetScan
miR-4426	ENSG00000145687	8m	TargetScan
miR-4426	ENSG00000145721	8m	TargetScan
miR-4426	ENSG00000145725	8m	TargetScan
miR-4426	ENSG00000145777	8m	TargetScan
miR-4426	ENSG00000145779	8m	TargetScan
miR-4426	ENSG00000145907	8m	TargetScan
miR-4426	ENSG00000145990	8m	TargetScan
miR-4426	ENSG00000146232	8m	TargetScan
miR-4426	ENSG00000146352	8m	TargetScan
miR-4426	ENSG00000146530	8m	TargetScan
miR-4426	ENSG00000146909	8m	TargetScan
miR-4426	ENSG00000147130	8m	TargetScan
miR-4426	ENSG00000147274	8m	TargetScan
miR-4426	ENSG00000147394	8m	TargetScan
miR-4426	ENSG00000147419	8m	TargetScan
miR-4426	ENSG00000147862	8m	TargetScan
miR-4426	ENSG00000147894	8m	TargetScan
miR-4426	ENSG00000148053	8m	TargetScan
miR-4426	ENSG00000148219	8m	TargetScan
miR-4426	ENSG00000148429	8m	TargetScan
miR-4426	ENSG00000148604	8m	TargetScan
miR-4426	ENSG00000149054	8m	TargetScan
miR-4426	ENSG00000149124	8m	TargetScan

miR-4426	ENSG00000149187	8m	TargetScan
miR-4426	ENSG00000149485	8m	TargetScan
miR-4426	ENSG00000149532	8m	TargetScan
miR-4426	ENSG00000151247	8m	TargetScan
miR-4426	ENSG00000151414	8m	TargetScan
miR-4426	ENSG00000151458	8m	TargetScan
miR-4426	ENSG00000151657	8m	TargetScan
miR-4426	ENSG00000152102	8m	TargetScan
miR-4426	ENSG00000152133	8m	TargetScan
miR-4426	ENSG00000152433	8m	TargetScan
miR-4426	ENSG00000152578	8m	TargetScan
miR-4426	ENSG00000152580	8m	TargetScan
miR-4426	ENSG00000152684	8m	TargetScan
miR-4426	ENSG00000153140	8m	TargetScan
miR-4426	ENSG00000153339	8m	TargetScan
miR-4426	ENSG00000153395	8m	TargetScan
miR-4426	ENSG00000153707	8m	TargetScan
miR-4426	ENSG00000153976	8m	TargetScan
miR-4426	ENSG00000154040	8m	TargetScan
miR-4426	ENSG00000154124	8m	TargetScan
miR-4426	ENSG00000154310	8m	TargetScan
miR-4426	ENSG00000154380	8m	TargetScan
miR-4426	ENSG00000154511	8m	TargetScan
miR-4426	ENSG00000154655	8m	TargetScan
miR-4426	ENSG00000154678	8m	TargetScan
miR-4426	ENSG00000155008	8m	TargetScan
miR-4426	ENSG00000155052	8m	TargetScan
miR-4426	ENSG00000155093	8m	TargetScan
miR-4426	ENSG00000155380	8m	TargetScan
miR-4426	ENSG00000155850	8m	TargetScan
miR-4426	ENSG00000155858	8m	TargetScan

miR-4426	ENSG00000156103	8m	TargetScan
miR-4426	ENSG00000156500	8m	TargetScan
miR-4426	ENSG00000156535	8m	TargetScan
miR-4426	ENSG00000156587	8m	TargetScan
miR-4426	ENSG00000156675	8m	TargetScan
miR-4426	ENSG00000156687	8m	TargetScan
miR-4426	ENSG00000156958	8m	TargetScan
miR-4426	ENSG00000157064	8m	TargetScan
miR-4426	ENSG00000157150	8m	TargetScan
miR-4426	ENSG00000157404	8m	TargetScan
miR-4426	ENSG00000157450	8m	TargetScan
miR-4426	ENSG00000157557	8m	TargetScan
miR-4426	ENSG00000158006	8m	TargetScan
miR-4426	ENSG00000158301	8m	TargetScan
miR-4426	ENSG00000158714	8m	TargetScan
miR-4426	ENSG00000159556	8m	TargetScan
miR-4426	ENSG00000159784	8m	TargetScan
miR-4426	ENSG00000160654	8m	TargetScan
miR-4426	ENSG00000160961	8m	TargetScan
miR-4426	ENSG00000161243	8m	TargetScan
miR-4426	ENSG00000161249	8m	TargetScan
miR-4426	ENSG00000161533	8m	TargetScan
miR-4426	ENSG00000161671	8m	TargetScan
miR-4426	ENSG00000162456	8m	TargetScan
miR-4426	ENSG00000162521	8m	TargetScan
miR-4426	ENSG00000162601	8m	TargetScan
miR-4426	ENSG00000162613	8m	TargetScan
miR-4426	ENSG00000162616	8m	TargetScan
miR-4426	ENSG00000162636	8m	TargetScan
miR-4426	ENSG00000162981	8m	TargetScan
miR-4426	ENSG00000163249	8m	TargetScan

miR-4426	ENSG00000163377	8m	TargetScan
miR-4426	ENSG00000163393	8m	TargetScan
miR-4426	ENSG00000163501	8m	TargetScan
miR-4426	ENSG00000163513	8m	TargetScan
miR-4426	ENSG00000163590	8m	TargetScan
miR-4426	ENSG00000163629	8m	TargetScan
miR-4426	ENSG00000163637	8m	TargetScan
miR-4426	ENSG00000163655	8m	TargetScan
miR-4426	ENSG00000163684	8m	TargetScan
miR-4426	ENSG00000163812	8m	TargetScan
miR-4426	ENSG00000163818	8m	TargetScan
miR-4426	ENSG00000164002	8m	TargetScan
miR-4426	ENSG00000164031	8m	TargetScan
miR-4426	ENSG00000164038	8m	TargetScan
miR-4426	ENSG00000164066	8m	TargetScan
miR-4426	ENSG00000164266	8m	TargetScan
miR-4426	ENSG00000164291	8m	TargetScan
miR-4426	ENSG00000164294	8m	TargetScan
miR-4426	ENSG00000164338	8m	TargetScan
miR-4426	ENSG00000164393	8m	TargetScan
miR-4426	ENSG00000164576	8m	TargetScan
miR-4426	ENSG00000164692	8m	TargetScan
miR-4426	ENSG00000164879	8m	TargetScan
miR-4426	ENSG00000164976	8m	TargetScan
miR-4426	ENSG00000164983	8m	TargetScan
miR-4426	ENSG00000165060	8m	TargetScan
miR-4426	ENSG00000165168	8m	TargetScan
miR-4426	ENSG00000165240	8m	TargetScan
miR-4426	ENSG00000165282	8m	TargetScan
miR-4426	ENSG00000165355	8m	TargetScan
miR-4426	ENSG00000165379	8m	TargetScan

miR-4426	ENSG00000165555	8m	TargetScan
miR-4426	ENSG00000165813	8m	TargetScan
miR-4426	ENSG00000165832	8m	TargetScan
miR-4426	ENSG00000166261	8m	TargetScan
miR-4426	ENSG00000166342	8m	TargetScan
miR-4426	ENSG00000166451	8m	TargetScan
miR-4426	ENSG00000166483	8m	TargetScan
miR-4426	ENSG00000166881	8m	TargetScan
miR-4426	ENSG00000166974	8m	TargetScan
miR-4426	ENSG00000167315	8m	TargetScan
miR-4426	ENSG00000167325	8m	TargetScan
miR-4426	ENSG00000167720	8m	TargetScan
miR-4426	ENSG00000167770	8m	TargetScan
miR-4426	ENSG00000167972	8m	TargetScan
miR-4426	ENSG00000168172	8m	TargetScan
miR-4426	ENSG00000168685	8m	TargetScan
miR-4426	ENSG00000168938	8m	TargetScan
miR-4426	ENSG00000169139	8m	TargetScan
miR-4426	ENSG00000169174	8m	TargetScan
miR-4426	ENSG00000169427	8m	TargetScan
miR-4426	ENSG00000169429	8m	TargetScan
miR-4426	ENSG00000169432	8m	TargetScan
miR-4426	ENSG00000169550	8m	TargetScan
miR-4426	ENSG00000169567	8m	TargetScan
miR-4426	ENSG00000169814	8m	TargetScan
miR-4426	ENSG00000169891	8m	TargetScan
miR-4426	ENSG00000170153	8m	TargetScan
miR-4426	ENSG00000170242	8m	TargetScan
miR-4426	ENSG00000170374	8m	TargetScan
miR-4426	ENSG00000170515	8m	TargetScan
miR-4426	ENSG00000170537	8m	TargetScan

miR-4426	ENSG00000170542	8m	TargetScan
miR-4426	ENSG00000170571	8m	TargetScan
miR-4426	ENSG00000170634	8m	TargetScan
miR-4426	ENSG00000170653	8m	TargetScan
miR-4426	ENSG00000171126	8m	TargetScan
miR-4426	ENSG00000171150	8m	TargetScan
miR-4426	ENSG00000171450	8m	TargetScan
miR-4426	ENSG00000171509	8m	TargetScan
miR-4426	ENSG00000171587	8m	TargetScan
miR-4426	ENSG00000171914	8m	TargetScan
miR-4426	ENSG00000172115	8m	TargetScan
miR-4426	ENSG00000172403	8m	TargetScan
miR-4426	ENSG00000172466	8m	TargetScan
miR-4426	ENSG00000172572	8m	TargetScan
miR-4426	ENSG00000172728	8m	TargetScan
miR-4426	ENSG00000172733	8m	TargetScan
miR-4426	ENSG00000172840	8m	TargetScan
miR-4426	ENSG00000173681	8m	TargetScan
miR-4426	ENSG00000173726	8m	TargetScan
miR-4426	ENSG00000174606	8m	TargetScan
miR-4426	ENSG00000174718	8m	TargetScan
miR-4426	ENSG00000174840	8m	TargetScan
miR-4426	ENSG00000174989	8m	TargetScan
miR-4426	ENSG00000175048	8m	TargetScan
miR-4426	ENSG00000175066	8m	TargetScan
miR-4426	ENSG00000175073	8m	TargetScan
miR-4426	ENSG00000175264	8m	TargetScan
miR-4426	ENSG00000175329	8m	TargetScan
miR-4426	ENSG00000176024	8m	TargetScan
miR-4426	ENSG00000176783	8m	TargetScan
miR-4426	ENSG00000177042	8m	TargetScan

miR-4426	ENSG00000177076	8m	TargetScan
miR-4426	ENSG00000177119	8m	TargetScan
miR-4426	ENSG00000177150	8m	TargetScan
miR-4426	ENSG00000177511	8m	TargetScan
miR-4426	ENSG00000177889	8m	TargetScan
miR-4426	ENSG00000177963	8m	TargetScan
miR-4426	ENSG00000178177	8m	TargetScan
miR-4426	ENSG00000178307	8m	TargetScan
miR-4426	ENSG00000178425	8m	TargetScan
miR-4426	ENSG00000179097	8m	TargetScan
miR-4426	ENSG00000179361	8m	TargetScan
miR-4426	ENSG00000179562	8m	TargetScan
miR-4426	ENSG00000179813	8m	TargetScan
miR-4426	ENSG00000179820	8m	TargetScan
miR-4426	ENSG00000180628	8m	TargetScan
miR-4426	ENSG00000181104	8m	TargetScan
miR-4426	ENSG00000181291	8m	TargetScan
miR-4426	ENSG00000181396	8m	TargetScan
miR-4426	ENSG00000181666	8m	TargetScan
miR-4426	ENSG00000181789	8m	TargetScan
miR-4426	ENSG00000181826	8m	TargetScan
miR-4426	ENSG00000181908	8m	TargetScan
miR-4426	ENSG00000182111	8m	TargetScan
miR-4426	ENSG00000182197	8m	TargetScan
miR-4426	ENSG00000182348	8m	TargetScan
miR-4426	ENSG00000183576	8m	TargetScan
miR-4426	ENSG00000183690	8m	TargetScan
miR-4426	ENSG00000183760	8m	TargetScan
miR-4426	ENSG00000183775	8m	TargetScan
miR-4426	ENSG00000183779	8m	TargetScan
miR-4426	ENSG00000183831	8m	TargetScan

miR-4426	ENSG00000183850	8m	TargetScan
miR-4426	ENSG00000184156	8m	TargetScan
miR-4426	ENSG00000184182	8m	TargetScan
miR-4426	ENSG00000184220	8m	TargetScan
miR-4426	ENSG00000184697	8m	TargetScan
miR-4426	ENSG00000184719	8m	TargetScan
miR-4426	ENSG00000184831	8m	TargetScan
miR-4426	ENSG00000184845	8m	TargetScan
miR-4426	ENSG00000185339	8m	TargetScan
miR-4426	ENSG00000185437	8m	TargetScan
miR-4426	ENSG00000185561	8m	TargetScan
miR-4426	ENSG00000185630	8m	TargetScan
miR-4426	ENSG00000185658	8m	TargetScan
miR-4426	ENSG00000185730	8m	TargetScan
miR-4426	ENSG00000185745	8m	TargetScan
miR-4426	ENSG00000186188	8m	TargetScan
miR-4426	ENSG00000186230	8m	TargetScan
miR-4426	ENSG00000186260	8m	TargetScan
miR-4426	ENSG00000186431	8m	TargetScan
miR-4426	ENSG00000186432	8m	TargetScan
miR-4426	ENSG00000186458	8m	TargetScan
miR-4426	ENSG00000186468	8m	TargetScan
miR-4426	ENSG00000186591	8m	TargetScan
miR-4426	ENSG00000186806	8m	TargetScan
miR-4426	ENSG00000186838	8m	TargetScan
miR-4426	ENSG00000187097	8m	TargetScan
miR-4426	ENSG00000187123	8m	TargetScan
miR-4426	ENSG00000187533	8m	TargetScan
miR-4426	ENSG00000187699	8m	TargetScan
miR-4426	ENSG00000187726	8m	TargetScan
miR-4426	ENSG00000187790	8m	TargetScan

miR-4426	ENSG00000188171	8m	TargetScan
miR-4426	ENSG00000188176	8m	TargetScan
miR-4426	ENSG00000188215	8m	TargetScan
miR-4426	ENSG00000188227	8m	TargetScan
miR-4426	ENSG00000188735	8m	TargetScan
miR-4426	ENSG00000188817	8m	TargetScan
miR-4426	ENSG00000188848	8m	TargetScan
miR-4426	ENSG00000189013	8m	TargetScan
miR-4426	ENSG00000189120	8m	TargetScan
miR-4426	ENSG00000189144	8m	TargetScan
miR-4426	ENSG00000189221	8m	TargetScan
miR-4426	ENSG00000189241	8m	TargetScan
miR-4426	ENSG00000189299	8m	TargetScan
miR-4426	ENSG00000189320	8m	TargetScan
miR-4426	ENSG00000196090	8m	TargetScan
miR-4426	ENSG00000196172	8m	TargetScan
miR-4426	ENSG00000196233	8m	TargetScan
miR-4426	ENSG00000196381	8m	TargetScan
miR-4426	ENSG00000196466	8m	TargetScan
miR-4426	ENSG00000196549	8m	TargetScan
miR-4426	ENSG00000196581	8m	TargetScan
miR-4426	ENSG00000196670	8m	TargetScan
miR-4426	ENSG00000196935	8m	TargetScan
miR-4426	ENSG00000197081	8m	TargetScan
miR-4426	ENSG00000197343	8m	TargetScan
miR-4426	ENSG00000197601	8m	TargetScan
miR-4426	ENSG00000197614	8m	TargetScan
miR-4426	ENSG00000197937	8m	TargetScan
miR-4426	ENSG00000198010	8m	TargetScan
miR-4426	ENSG00000198042	8m	TargetScan
miR-4426	ENSG00000198056	8m	TargetScan

miR-4426	ENSG00000198178	8m	TargetScan
miR-4426	ENSG00000198265	8m	TargetScan
miR-4426	ENSG00000198663	8m	TargetScan
miR-4426	ENSG00000198736	8m	TargetScan
miR-4426	ENSG00000204007	8m	TargetScan
miR-4426	ENSG00000204020	8m	TargetScan
miR-4426	ENSG00000204161	8m	TargetScan
miR-4426	ENSG00000204176	8m	TargetScan
miR-4426	ENSG00000204308	8m	TargetScan
miR-4426	ENSG00000204389	8m	TargetScan
miR-4426	ENSG00000204611	8m	TargetScan
miR-4426	ENSG00000204681	8m	TargetScan
miR-4426	ENSG00000204767	8m	TargetScan
miR-4426	ENSG00000204882	8m	TargetScan
miR-4426	ENSG00000204952	8m	TargetScan
miR-4426	ENSG00000205476	8m	TargetScan
miR-4426	ENSG00000205670	8m	TargetScan
miR-4426	ENSG00000213186	8m	TargetScan
miR-4426	ENSG00000213762	8m	TargetScan
miR-4426	ENSG00000213853	8m	TargetScan
miR-4426	ENSG00000214029	8m	TargetScan
miR-4426	ENSG00000215114	8m	TargetScan
miR-4426	ENSG00000215271	8m	TargetScan
miR-4426	ENSG00000221947	8m	TargetScan
miR-4426	ENSG00000221949	8m	TargetScan
miR-4426	ENSG00000226479	8m	TargetScan
miR-4426	ENSG00000226887	8m	TargetScan
miR-4426	ENSG00000228716	8m	TargetScan
miR-4426	ENSG00000233436	8m	TargetScan
miR-4426	ENSG00000233927	8m	TargetScan
miR-4426	ENSG00000234444	8m	TargetScan

miR-4426	ENSG00000240720	8m	TargetScan
miR-4426	ENSG00000241106	8m	TargetScan
miR-4426	ENSG00000242265	8m	TargetScan
miR-4426	ENSG00000242550	8m	TargetScan
miR-4426	ENSG00000242689	8m	TargetScan
miR-4426	ENSG00000244754	8m	TargetScan
miR-4426	ENSG00000256053	8m	TargetScan
miR-4426	ENSG00000259363	8m	TargetScan
miR-4426	ENSG00000260903	8m	TargetScan
miR-4426	ENSG00000266173	8m	TargetScan
miR-4426	ENSG00000268324	8m	TargetScan
miR-4426	ENSG00000268635	8m	TargetScan
miR-4426	ENSG00000269343	8m	TargetScan
miR-4426	ENSG00000272195	8m	TargetScan
miR-4426	ENSG00000273079	8m	TargetScan
miR-4426	ENSG00000280789	8m	TargetScan
miR-4426	ENSG00000280893	8m	TargetScan
miR-4426	ENSG00000287694	8m	TargetScan
miR-4444	ENSG00000001561	8m	TargetScan
miR-4444	ENSG00000005075	8m	TargetScan
miR-4444	ENSG00000008196	8m	TargetScan
miR-4444	ENSG00000009709	8m	TargetScan
miR-4444	ENSG00000011258	8m	TargetScan
miR-4444	ENSG00000015475	8m	TargetScan
miR-4444	ENSG00000043591	8m	TargetScan
miR-4444	ENSG00000051620	8m	TargetScan
miR-4444	ENSG00000053371	8m	TargetScan
miR-4444	ENSG00000053770	8m	TargetScan
miR-4444	ENSG00000055118	8m	TargetScan
miR-4444	ENSG00000064393	8m	TargetScan
miR-4444	ENSG00000064995	8m	TargetScan

miR-4444	ENSG00000070915	8m	TargetScan
miR-4444	ENSG00000077458	8m	TargetScan
miR-4444	ENSG00000078295	8m	TargetScan
miR-4444	ENSG00000078549	8m	TargetScan
miR-4444	ENSG00000080200	8m	TargetScan
miR-4444	ENSG00000082515	8m	TargetScan
miR-4444	ENSG00000082898	8m	TargetScan
miR-4444	ENSG00000088386	8m	TargetScan
miR-4444	ENSG00000092841	8m	TargetScan
miR-4444	ENSG00000101109	8m	TargetScan
miR-4444	ENSG00000101213	8m	TargetScan
miR-4444	ENSG00000101266	8m	TargetScan
miR-4444	ENSG00000101412	8m	TargetScan
miR-4444	ENSG00000101413	8m	TargetScan
miR-4444	ENSG00000102385	8m	TargetScan
miR-4444	ENSG00000104219	8m	TargetScan
miR-4444	ENSG00000107929	8m	TargetScan
miR-4444	ENSG00000108829	8m	TargetScan
miR-4444	ENSG00000112531	8m	TargetScan
miR-4444	ENSG00000113739	8m	TargetScan
miR-4444	ENSG00000116675	8m	TargetScan
miR-4444	ENSG00000117000	8m	TargetScan
miR-4444	ENSG00000117020	8m	TargetScan
miR-4444	ENSG00000122958	8m	TargetScan
miR-4444	ENSG00000124602	8m	TargetScan
miR-4444	ENSG00000124731	8m	TargetScan
miR-4444	ENSG00000125285	8m	TargetScan
miR-4444	ENSG00000125508	8m	TargetScan
miR-4444	ENSG00000129250	8m	TargetScan
miR-4444	ENSG00000130703	8m	TargetScan
miR-4444	ENSG00000131931	8m	TargetScan

miR-4444	ENSG00000133937	8m	TargetScan
miR-4444	ENSG00000134317	8m	TargetScan
miR-4444	ENSG00000134352	8m	TargetScan
miR-4444	ENSG00000134444	8m	TargetScan
miR-4444	ENSG00000135333	8m	TargetScan
miR-4444	ENSG00000136709	8m	TargetScan
miR-4444	ENSG00000137547	8m	TargetScan
miR-4444	ENSG00000137992	8m	TargetScan
miR-4444	ENSG00000139974	8m	TargetScan
miR-4444	ENSG00000139977	8m	TargetScan
miR-4444	ENSG00000141068	8m	TargetScan
miR-4444	ENSG00000141570	8m	TargetScan
miR-4444	ENSG00000142556	8m	TargetScan
miR-4444	ENSG00000144036	8m	TargetScan
miR-4444	ENSG00000144567	8m	TargetScan
miR-4444	ENSG00000148826	8m	TargetScan
miR-4444	ENSG00000150540	8m	TargetScan
miR-4444	ENSG00000151849	8m	TargetScan
miR-4444	ENSG00000152056	8m	TargetScan
miR-4444	ENSG00000152377	8m	TargetScan
miR-4444	ENSG00000154124	8m	TargetScan
miR-4444	ENSG00000156587	8m	TargetScan
miR-4444	ENSG00000159723	8m	TargetScan
miR-4444	ENSG00000160293	8m	TargetScan
miR-4444	ENSG00000161057	8m	TargetScan
miR-4444	ENSG00000162981	8m	TargetScan
miR-4444	ENSG00000163013	8m	TargetScan
miR-4444	ENSG00000164187	8m	TargetScan
miR-4444	ENSG00000164512	8m	TargetScan
miR-4444	ENSG00000164603	8m	TargetScan
miR-4444	ENSG00000165055	8m	TargetScan

miR-4444	ENSG00000165490	8m	TargetScan
miR-4444	ENSG00000166450	8m	TargetScan
miR-4444	ENSG00000166913	8m	TargetScan
miR-4444	ENSG00000166928	8m	TargetScan
miR-4444	ENSG00000168496	8m	TargetScan
miR-4444	ENSG00000168958	8m	TargetScan
miR-4444	ENSG00000170500	8m	TargetScan
miR-4444	ENSG00000170873	8m	TargetScan
miR-4444	ENSG00000170927	8m	TargetScan
miR-4444	ENSG00000171448	8m	TargetScan
miR-4444	ENSG00000171517	8m	TargetScan
miR-4444	ENSG00000171617	8m	TargetScan
miR-4444	ENSG00000173681	8m	TargetScan
miR-4444	ENSG00000175106	8m	TargetScan
miR-4444	ENSG00000177971	8m	TargetScan
miR-4444	ENSG00000181264	8m	TargetScan
miR-4444	ENSG00000181896	8m	TargetScan
miR-4444	ENSG00000182107	8m	TargetScan
miR-4444	ENSG00000182831	8m	TargetScan
miR-4444	ENSG00000182985	8m	TargetScan
miR-4444	ENSG00000184530	8m	TargetScan
miR-4444	ENSG00000185278	8m	TargetScan
miR-4444	ENSG00000186976	8m	TargetScan
miR-4444	ENSG00000187239	8m	TargetScan
miR-4444	ENSG00000188822	8m	TargetScan
miR-4444	ENSG00000204977	8m	TargetScan
miR-4444	ENSG00000213699	8m	TargetScan
miR-4444	ENSG00000214046	8m	TargetScan
miR-4444	ENSG00000214113	8m	TargetScan
miR-4444	ENSG00000222009	8m	TargetScan
miR-4444	ENSG00000253251	8m	TargetScan

miR-4468	ENSG00000000003	8m	TargetScan
miR-4468	ENSG00000002834	8m	TargetScan
miR-4468	ENSG00000003249	8m	TargetScan
miR-4468	ENSG00000004866	8m	TargetScan
miR-4468	ENSG00000005175	8m	TargetScan
miR-4468	ENSG00000005810	8m	TargetScan
miR-4468	ENSG00000006432	8m	TargetScan
miR-4468	ENSG00000006747	8m	TargetScan
miR-4468	ENSG00000006756	8m	TargetScan
miR-4468	ENSG00000007174	8m	TargetScan
miR-4468	ENSG00000007376	8m	TargetScan
miR-4468	ENSG00000007392	8m	TargetScan
miR-4468	ENSG00000008130	8m	TargetScan
miR-4468	ENSG00000008283	8m	TargetScan
miR-4468	ENSG00000009765	8m	TargetScan
miR-4468	ENSG00000009830	8m	TargetScan
miR-4468	ENSG00000010282	8m	TargetScan
miR-4468	ENSG00000011347	8m	TargetScan
miR-4468	ENSG00000011451	8m	TargetScan
miR-4468	ENSG00000012822	8m	TargetScan
miR-4468	ENSG00000014138	8m	TargetScan
miR-4468	ENSG00000019582	8m	TargetScan
miR-4468	ENSG00000020922	8m	TargetScan
miR-4468	ENSG00000023902	8m	TargetScan
miR-4468	ENSG00000025770	8m	TargetScan
miR-4468	ENSG00000026652	8m	TargetScan
miR-4468	ENSG00000026751	8m	TargetScan
miR-4468	ENSG00000029364	8m	TargetScan
miR-4468	ENSG00000030582	8m	TargetScan
miR-4468	ENSG00000033011	8m	TargetScan
miR-4468	ENSG00000034152	8m	TargetScan

miR-4468	ENSG00000037280	8m	TargetScan
miR-4468	ENSG00000039068	8m	TargetScan
miR-4468	ENSG00000040341	8m	TargetScan
miR-4468	ENSG00000041802	8m	TargetScan
miR-4468	ENSG00000043143	8m	TargetScan
miR-4468	ENSG00000047578	8m	TargetScan
miR-4468	ENSG00000048392	8m	TargetScan
miR-4468	ENSG00000049618	8m	TargetScan
miR-4468	ENSG00000049883	8m	TargetScan
miR-4468	ENSG00000050393	8m	TargetScan
miR-4468	ENSG00000050748	8m	TargetScan
miR-4468	ENSG00000051620	8m	TargetScan
miR-4468	ENSG00000053702	8m	TargetScan
miR-4468	ENSG00000054282	8m	TargetScan
miR-4468	ENSG00000054392	8m	TargetScan
miR-4468	ENSG00000054523	8m	TargetScan
miR-4468	ENSG00000058335	8m	TargetScan
miR-4468	ENSG00000058866	8m	TargetScan
miR-4468	ENSG00000059122	8m	TargetScan
miR-4468	ENSG00000059145	8m	TargetScan
miR-4468	ENSG00000060709	8m	TargetScan
miR-4468	ENSG00000060982	8m	TargetScan
miR-4468	ENSG00000063854	8m	TargetScan
miR-4468	ENSG00000064115	8m	TargetScan
miR-4468	ENSG00000064393	8m	TargetScan
miR-4468	ENSG00000065135	8m	TargetScan
miR-4468	ENSG00000065243	8m	TargetScan
miR-4468	ENSG00000065457	8m	TargetScan
miR-4468	ENSG00000065600	8m	TargetScan
miR-4468	ENSG00000065802	8m	TargetScan
miR-4468	ENSG00000066084	8m	TargetScan

miR-4468	ENSG00000066294	8m	TargetScan
miR-4468	ENSG00000066654	8m	TargetScan
miR-4468	ENSG00000068394	8m	TargetScan
miR-4468	ENSG00000069329	8m	TargetScan
miR-4468	ENSG00000069424	8m	TargetScan
miR-4468	ENSG00000070081	8m	TargetScan
miR-4468	ENSG00000070190	8m	TargetScan
miR-4468	ENSG00000070495	8m	TargetScan
miR-4468	ENSG00000070882	8m	TargetScan
miR-4468	ENSG00000071189	8m	TargetScan
miR-4468	ENSG00000071655	8m	TargetScan
miR-4468	ENSG00000072849	8m	TargetScan
miR-4468	ENSG00000073464	8m	TargetScan
miR-4468	ENSG00000074755	8m	TargetScan
miR-4468	ENSG00000075035	8m	TargetScan
miR-4468	ENSG00000075043	8m	TargetScan
miR-4468	ENSG00000075651	8m	TargetScan
miR-4468	ENSG00000076321	8m	TargetScan
miR-4468	ENSG00000076641	8m	TargetScan
miR-4468	ENSG00000077327	8m	TargetScan
miR-4468	ENSG00000078124	8m	TargetScan
miR-4468	ENSG00000078295	8m	TargetScan
miR-4468	ENSG00000078900	8m	TargetScan
miR-4468	ENSG00000079102	8m	TargetScan
miR-4468	ENSG00000079156	8m	TargetScan
miR-4468	ENSG00000079308	8m	TargetScan
miR-4468	ENSG00000079950	8m	TargetScan
miR-4468	ENSG00000080166	8m	TargetScan
miR-4468	ENSG00000080845	8m	TargetScan
miR-4468	ENSG00000081148	8m	TargetScan
miR-4468	ENSG00000081307	8m	TargetScan

miR-4468	ENSG00000081870	8m	TargetScan
miR-4468	ENSG00000082701	8m	TargetScan
miR-4468	ENSG00000082805	8m	TargetScan
miR-4468	ENSG00000085117	8m	TargetScan
miR-4468	ENSG00000085449	8m	TargetScan
miR-4468	ENSG00000085741	8m	TargetScan
miR-4468	ENSG00000086475	8m	TargetScan
miR-4468	ENSG00000087253	8m	TargetScan
miR-4468	ENSG00000087274	8m	TargetScan
miR-4468	ENSG00000087301	8m	TargetScan
miR-4468	ENSG00000087460	8m	TargetScan
miR-4468	ENSG00000087470	8m	TargetScan
miR-4468	ENSG00000089063	8m	TargetScan
miR-4468	ENSG00000089356	8m	TargetScan
miR-4468	ENSG00000089916	8m	TargetScan
miR-4468	ENSG00000090615	8m	TargetScan
miR-4468	ENSG00000090863	8m	TargetScan
miR-4468	ENSG00000090924	8m	TargetScan
miR-4468	ENSG00000091527	8m	TargetScan
miR-4468	ENSG00000091622	8m	TargetScan
miR-4468	ENSG00000092054	8m	TargetScan
miR-4468	ENSG00000094755	8m	TargetScan
miR-4468	ENSG00000094804	8m	TargetScan
miR-4468	ENSG00000096060	8m	TargetScan
miR-4468	ENSG00000096968	8m	TargetScan
miR-4468	ENSG00000099282	8m	TargetScan
miR-4468	ENSG00000099385	8m	TargetScan
miR-4468	ENSG00000099715	8m	TargetScan
miR-4468	ENSG00000099875	8m	TargetScan
miR-4468	ENSG00000099968	8m	TargetScan
miR-4468	ENSG00000100012	8m	TargetScan

miR-4468	ENSG00000100053	8m	TargetScan
miR-4468	ENSG00000100099	8m	TargetScan
miR-4468	ENSG00000100105	8m	TargetScan
miR-4468	ENSG00000100142	8m	TargetScan
miR-4468	ENSG00000100162	8m	TargetScan
miR-4468	ENSG00000100226	8m	TargetScan
miR-4468	ENSG00000100304	8m	TargetScan
miR-4468	ENSG00000100364	8m	TargetScan
miR-4468	ENSG00000100441	8m	TargetScan
miR-4468	ENSG00000100461	8m	TargetScan
miR-4468	ENSG00000100528	8m	TargetScan
miR-4468	ENSG00000100596	8m	TargetScan
miR-4468	ENSG00000100650	8m	TargetScan
miR-4468	ENSG00000100796	8m	TargetScan
miR-4468	ENSG00000100811	8m	TargetScan
miR-4468	ENSG00000100842	8m	TargetScan
miR-4468	ENSG00000101019	8m	TargetScan
miR-4468	ENSG00000101162	8m	TargetScan
miR-4468	ENSG00000101230	8m	TargetScan
miR-4468	ENSG00000101265	8m	TargetScan
miR-4468	ENSG00000101282	8m	TargetScan
miR-4468	ENSG00000101290	8m	TargetScan
miR-4468	ENSG00000101307	8m	TargetScan
miR-4468	ENSG00000101412	8m	TargetScan
miR-4468	ENSG00000101546	8m	TargetScan
miR-4468	ENSG00000101670	8m	TargetScan
miR-4468	ENSG00000101916	8m	TargetScan
miR-4468	ENSG00000101966	8m	TargetScan
miR-4468	ENSG00000102053	8m	TargetScan
miR-4468	ENSG00000102104	8m	TargetScan
miR-4468	ENSG00000102172	8m	TargetScan

miR-4468	ENSG00000102290	8m	TargetScan
miR-4468	ENSG00000102313	8m	TargetScan
miR-4468	ENSG00000102383	8m	TargetScan
miR-4468	ENSG00000102870	8m	TargetScan
miR-4468	ENSG00000102900	8m	TargetScan
miR-4468	ENSG00000102921	8m	TargetScan
miR-4468	ENSG00000103005	8m	TargetScan
miR-4468	ENSG00000103056	8m	TargetScan
miR-4468	ENSG00000103196	8m	TargetScan
miR-4468	ENSG00000103257	8m	TargetScan
miR-4468	ENSG00000103353	8m	TargetScan
miR-4468	ENSG00000103356	8m	TargetScan
miR-4468	ENSG00000103404	8m	TargetScan
miR-4468	ENSG00000103423	8m	TargetScan
miR-4468	ENSG00000103485	8m	TargetScan
miR-4468	ENSG00000103490	8m	TargetScan
miR-4468	ENSG00000103769	8m	TargetScan
miR-4468	ENSG00000104081	8m	TargetScan
miR-4468	ENSG00000104142	8m	TargetScan
miR-4468	ENSG00000104154	8m	TargetScan
miR-4468	ENSG00000104332	8m	TargetScan
miR-4468	ENSG00000104427	8m	TargetScan
miR-4468	ENSG00000104611	8m	TargetScan
miR-4468	ENSG00000104671	8m	TargetScan
miR-4468	ENSG00000104731	8m	TargetScan
miR-4468	ENSG00000104866	8m	TargetScan
miR-4468	ENSG00000104938	8m	TargetScan
miR-4468	ENSG00000104960	8m	TargetScan
miR-4468	ENSG00000105127	8m	TargetScan
miR-4468	ENSG00000105281	8m	TargetScan
miR-4468	ENSG00000105352	8m	TargetScan

miR-4468	ENSG00000105366	8m	TargetScan
miR-4468	ENSG00000105402	8m	TargetScan
miR-4468	ENSG00000105419	8m	TargetScan
miR-4468	ENSG00000105492	8m	TargetScan
miR-4468	ENSG00000105514	8m	TargetScan
miR-4468	ENSG00000105750	8m	TargetScan
miR-4468	ENSG00000105835	8m	TargetScan
miR-4468	ENSG00000106344	8m	TargetScan
miR-4468	ENSG00000106399	8m	TargetScan
miR-4468	ENSG00000107130	8m	TargetScan
miR-4468	ENSG00000107185	8m	TargetScan
miR-4468	ENSG00000107282	8m	TargetScan
miR-4468	ENSG00000107562	8m	TargetScan
miR-4468	ENSG00000107581	8m	TargetScan
miR-4468	ENSG00000107929	8m	TargetScan
miR-4468	ENSG00000108106	8m	TargetScan
miR-4468	ENSG00000108175	8m	TargetScan
miR-4468	ENSG00000108255	8m	TargetScan
miR-4468	ENSG00000108306	8m	TargetScan
miR-4468	ENSG00000108309	8m	TargetScan
miR-4468	ENSG00000108384	8m	TargetScan
miR-4468	ENSG00000108387	8m	TargetScan
miR-4468	ENSG00000108599	8m	TargetScan
miR-4468	ENSG00000108669	8m	TargetScan
miR-4468	ENSG00000108788	8m	TargetScan
miR-4468	ENSG00000108797	8m	TargetScan
miR-4468	ENSG00000108823	8m	TargetScan
miR-4468	ENSG00000108946	8m	TargetScan
miR-4468	ENSG00000109016	8m	TargetScan
miR-4468	ENSG00000109046	8m	TargetScan
miR-4468	ENSG00000109079	8m	TargetScan

miR-4468	ENSG00000109472	8m	TargetScan
miR-4468	ENSG00000109519	8m	TargetScan
miR-4468	ENSG00000109685	8m	TargetScan
miR-4468	ENSG00000109756	8m	TargetScan
miR-4468	ENSG00000109787	8m	TargetScan
miR-4468	ENSG00000110245	8m	TargetScan
miR-4468	ENSG00000110448	8m	TargetScan
miR-4468	ENSG00000110583	8m	TargetScan
miR-4468	ENSG00000110693	8m	TargetScan
miR-4468	ENSG00000110713	8m	TargetScan
miR-4468	ENSG00000110719	8m	TargetScan
miR-4468	ENSG00000110851	8m	TargetScan
miR-4468	ENSG00000111266	8m	TargetScan
miR-4468	ENSG00000111371	8m	TargetScan
miR-4468	ENSG00000111452	8m	TargetScan
miR-4468	ENSG00000111490	8m	TargetScan
miR-4468	ENSG00000111615	8m	TargetScan
miR-4468	ENSG00000111696	8m	TargetScan
miR-4468	ENSG00000111725	8m	TargetScan
miR-4468	ENSG00000111897	8m	TargetScan
miR-4468	ENSG00000112182	8m	TargetScan
miR-4468	ENSG00000112276	8m	TargetScan
miR-4468	ENSG00000112339	8m	TargetScan
miR-4468	ENSG00000112531	8m	TargetScan
miR-4468	ENSG00000112576	8m	TargetScan
miR-4468	ENSG00000112759	8m	TargetScan
miR-4468	ENSG00000112763	8m	TargetScan
miR-4468	ENSG00000112769	8m	TargetScan
miR-4468	ENSG00000112773	8m	TargetScan
miR-4468	ENSG00000112977	8m	TargetScan
miR-4468	ENSG00000112996	8m	TargetScan

miR-4468	ENSG00000113300	8m	TargetScan
miR-4468	ENSG00000113318	8m	TargetScan
miR-4468	ENSG00000113319	8m	TargetScan
miR-4468	ENSG00000113361	8m	TargetScan
miR-4468	ENSG00000113719	8m	TargetScan
miR-4468	ENSG00000114054	8m	TargetScan
miR-4468	ENSG00000114316	8m	TargetScan
miR-4468	ENSG00000114391	8m	TargetScan
miR-4468	ENSG00000114491	8m	TargetScan
miR-4468	ENSG00000114738	8m	TargetScan
miR-4468	ENSG00000114857	8m	TargetScan
miR-4468	ENSG00000115165	8m	TargetScan
miR-4468	ENSG00000115325	8m	TargetScan
miR-4468	ENSG00000115361	8m	TargetScan
miR-4468	ENSG00000115364	8m	TargetScan
miR-4468	ENSG00000115461	8m	TargetScan
miR-4468	ENSG00000115514	8m	TargetScan
miR-4468	ENSG00000115524	8m	TargetScan
miR-4468	ENSG00000115548	8m	TargetScan
miR-4468	ENSG00000115561	8m	TargetScan
miR-4468	ENSG00000115602	8m	TargetScan
miR-4468	ENSG00000115694	8m	TargetScan
miR-4468	ENSG00000115705	8m	TargetScan
miR-4468	ENSG00000115758	8m	TargetScan
miR-4468	ENSG00000115808	8m	TargetScan
miR-4468	ENSG00000116132	8m	TargetScan
miR-4468	ENSG00000116198	8m	TargetScan
miR-4468	ENSG00000116209	8m	TargetScan
miR-4468	ENSG00000116260	8m	TargetScan
miR-4468	ENSG00000116299	8m	TargetScan
miR-4468	ENSG00000116337	8m	TargetScan

miR-4468	ENSG00000116459	8m	TargetScan
miR-4468	ENSG00000116489	8m	TargetScan
miR-4468	ENSG00000116667	8m	TargetScan
miR-4468	ENSG00000116981	8m	TargetScan
miR-4468	ENSG00000117020	8m	TargetScan
miR-4468	ENSG00000117122	8m	TargetScan
miR-4468	ENSG00000117245	8m	TargetScan
miR-4468	ENSG00000117318	8m	TargetScan
miR-4468	ENSG00000117385	8m	TargetScan
miR-4468	ENSG00000117569	8m	TargetScan
miR-4468	ENSG00000117868	8m	TargetScan
miR-4468	ENSG00000118418	8m	TargetScan
miR-4468	ENSG00000118454	8m	TargetScan
miR-4468	ENSG00000118496	8m	TargetScan
miR-4468	ENSG00000118523	8m	TargetScan
miR-4468	ENSG00000118705	8m	TargetScan
miR-4468	ENSG00000118849	8m	TargetScan
miR-4468	ENSG00000118898	8m	TargetScan
miR-4468	ENSG00000119314	8m	TargetScan
miR-4468	ENSG00000119335	8m	TargetScan
miR-4468	ENSG00000119446	8m	TargetScan
miR-4468	ENSG00000119547	8m	TargetScan
miR-4468	ENSG00000119685	8m	TargetScan
miR-4468	ENSG00000119711	8m	TargetScan
miR-4468	ENSG00000119737	8m	TargetScan
miR-4468	ENSG00000120693	8m	TargetScan
miR-4468	ENSG00000120907	8m	TargetScan
miR-4468	ENSG00000120963	8m	TargetScan
miR-4468	ENSG00000121413	8m	TargetScan
miR-4468	ENSG00000121741	8m	TargetScan
miR-4468	ENSG00000121904	8m	TargetScan

miR-4468	ENSG00000122012	8m	TargetScan
miR-4468	ENSG00000122254	8m	TargetScan
miR-4468	ENSG00000122490	8m	TargetScan
miR-4468	ENSG00000122547	8m	TargetScan
miR-4468	ENSG00000122729	8m	TargetScan
miR-4468	ENSG00000122863	8m	TargetScan
miR-4468	ENSG00000122965	8m	TargetScan
miR-4468	ENSG00000123119	8m	TargetScan
miR-4468	ENSG00000123213	8m	TargetScan
miR-4468	ENSG00000123342	8m	TargetScan
miR-4468	ENSG00000123570	8m	TargetScan
miR-4468	ENSG00000124193	8m	TargetScan
miR-4468	ENSG00000124203	8m	TargetScan
miR-4468	ENSG00000124334	8m	TargetScan
miR-4468	ENSG00000124334	8m	TargetScan
miR-4468	ENSG00000124486	8m	TargetScan
miR-4468	ENSG00000124535	8m	TargetScan
miR-4468	ENSG00000124635	8m	TargetScan
miR-4468	ENSG00000124780	8m	TargetScan
miR-4468	ENSG00000124788	8m	TargetScan
miR-4468	ENSG00000125246	8m	TargetScan
miR-4468	ENSG00000125319	8m	TargetScan
miR-4468	ENSG00000125354	8m	TargetScan
miR-4468	ENSG00000125484	8m	TargetScan
miR-4468	ENSG00000125810	8m	TargetScan
miR-4468	ENSG00000125812	8m	TargetScan
miR-4468	ENSG00000125818	8m	TargetScan
miR-4468	ENSG00000125845	8m	TargetScan
miR-4468	ENSG00000126215	8m	TargetScan
miR-4468	ENSG00000126561	8m	TargetScan
miR-4468	ENSG00000126822	8m	TargetScan

miR-4468	ENSG00000127184	8m	TargetScan
miR-4468	ENSG00000127191	8m	TargetScan
miR-4468	ENSG00000127527	8m	TargetScan
miR-4468	ENSG00000127554	8m	TargetScan
miR-4468	ENSG00000127838	8m	TargetScan
miR-4468	ENSG00000128340	8m	TargetScan
miR-4468	ENSG00000128915	8m	TargetScan
miR-4468	ENSG00000128944	8m	TargetScan
miR-4468	ENSG00000129007	8m	TargetScan
miR-4468	ENSG00000129195	8m	TargetScan
miR-4468	ENSG00000129353	8m	TargetScan
miR-4468	ENSG00000129422	8m	TargetScan
miR-4468	ENSG00000129933	8m	TargetScan
miR-4468	ENSG00000129968	8m	TargetScan
miR-4468	ENSG00000130270	8m	TargetScan
miR-4468	ENSG00000130517	8m	TargetScan
miR-4468	ENSG00000130520	8m	TargetScan
miR-4468	ENSG00000130560	8m	TargetScan
miR-4468	ENSG00000130720	8m	TargetScan
miR-4468	ENSG00000130826	8m	TargetScan
miR-4468	ENSG00000130958	8m	TargetScan
miR-4468	ENSG00000131471	8m	TargetScan
miR-4468	ENSG00000131724	8m	TargetScan
miR-4468	ENSG00000131873	8m	TargetScan
miR-4468	ENSG00000131899	8m	TargetScan
miR-4468	ENSG00000132164	8m	TargetScan
miR-4468	ENSG00000132275	8m	TargetScan
miR-4468	ENSG00000132330	8m	TargetScan
miR-4468	ENSG00000132563	8m	TargetScan
miR-4468	ENSG00000132640	8m	TargetScan
miR-4468	ENSG00000132694	8m	TargetScan

miR-4468	ENSG00000132740	8m	TargetScan
miR-4468	ENSG00000132749	8m	TargetScan
miR-4468	ENSG00000132823	8m	TargetScan
miR-4468	ENSG00000132849	8m	TargetScan
miR-4468	ENSG00000132854	8m	TargetScan
miR-4468	ENSG00000132906	8m	TargetScan
miR-4468	ENSG00000132952	8m	TargetScan
miR-4468	ENSG00000132963	8m	TargetScan
miR-4468	ENSG00000133083	8m	TargetScan
miR-4468	ENSG00000133313	8m	TargetScan
miR-4468	ENSG00000133321	8m	TargetScan
miR-4468	ENSG00000133704	8m	TargetScan
miR-4468	ENSG00000133731	8m	TargetScan
miR-4468	ENSG00000133983	8m	TargetScan
miR-4468	ENSG00000134042	8m	TargetScan
miR-4468	ENSG00000134061	8m	TargetScan
miR-4468	ENSG00000134086	8m	TargetScan
miR-4468	ENSG00000134186	8m	TargetScan
miR-4468	ENSG00000134193	8m	TargetScan
miR-4468	ENSG00000134245	8m	TargetScan
miR-4468	ENSG00000134253	8m	TargetScan
miR-4468	ENSG00000134365	8m	TargetScan
miR-4468	ENSG00000134440	8m	TargetScan
miR-4468	ENSG00000134551	8m	TargetScan
miR-4468	ENSG00000134716	8m	TargetScan
miR-4468	ENSG00000134762	8m	TargetScan
miR-4468	ENSG00000134779	8m	TargetScan
miR-4468	ENSG00000134809	8m	TargetScan
miR-4468	ENSG00000134897	8m	TargetScan
miR-4468	ENSG00000134962	8m	TargetScan
miR-4468	ENSG00000134987	8m	TargetScan

miR-4468	ENSG00000135049	8m	TargetScan
miR-4468	ENSG00000135097	8m	TargetScan
miR-4468	ENSG00000135316	8m	TargetScan
miR-4468	ENSG00000135373	8m	TargetScan
miR-4468	ENSG00000135472	8m	TargetScan
miR-4468	ENSG00000135604	8m	TargetScan
miR-4468	ENSG00000135823	8m	TargetScan
miR-4468	ENSG00000135824	8m	TargetScan
miR-4468	ENSG00000135953	8m	TargetScan
miR-4468	ENSG00000135999	8m	TargetScan
miR-4468	ENSG00000136169	8m	TargetScan
miR-4468	ENSG00000136261	8m	TargetScan
miR-4468	ENSG00000136279	8m	TargetScan
miR-4468	ENSG00000136448	8m	TargetScan
miR-4468	ENSG00000136504	8m	TargetScan
miR-4468	ENSG00000136731	8m	TargetScan
miR-4468	ENSG00000136908	8m	TargetScan
miR-4468	ENSG00000136925	8m	TargetScan
miR-4468	ENSG00000136944	8m	TargetScan
miR-4468	ENSG00000137055	8m	TargetScan
miR-4468	ENSG00000137070	8m	TargetScan
miR-4468	ENSG00000137185	8m	TargetScan
miR-4468	ENSG00000137204	8m	TargetScan
miR-4468	ENSG00000137265	8m	TargetScan
miR-4468	ENSG00000137414	8m	TargetScan
miR-4468	ENSG00000137486	8m	TargetScan
miR-4468	ENSG00000137496	8m	TargetScan
miR-4468	ENSG00000137502	8m	TargetScan
miR-4468	ENSG00000137992	8m	TargetScan
miR-4468	ENSG00000138160	8m	TargetScan
miR-4468	ENSG00000138286	8m	TargetScan

miR-4468	ENSG00000138311	8m	TargetScan
miR-4468	ENSG00000138347	8m	TargetScan
miR-4468	ENSG00000138443	8m	TargetScan
miR-4468	ENSG00000138463	8m	TargetScan
miR-4468	ENSG00000138622	8m	TargetScan
miR-4468	ENSG00000138670	8m	TargetScan
miR-4468	ENSG00000138741	8m	TargetScan
miR-4468	ENSG00000138835	8m	TargetScan
miR-4468	ENSG00000138867	8m	TargetScan
miR-4468	ENSG00000139116	8m	TargetScan
miR-4468	ENSG00000139190	8m	TargetScan
miR-4468	ENSG00000139197	8m	TargetScan
miR-4468	ENSG00000139329	8m	TargetScan
miR-4468	ENSG00000139722	8m	TargetScan
miR-4468	ENSG00000140009	8m	TargetScan
miR-4468	ENSG00000140262	8m	TargetScan
miR-4468	ENSG00000140464	8m	TargetScan
miR-4468	ENSG00000140471	8m	TargetScan
miR-4468	ENSG00000140527	8m	TargetScan
miR-4468	ENSG00000140548	8m	TargetScan
miR-4468	ENSG00000140688	8m	TargetScan
miR-4468	ENSG00000140807	8m	TargetScan
miR-4468	ENSG00000140948	8m	TargetScan
miR-4468	ENSG00000141076	8m	TargetScan
miR-4468	ENSG00000141219	8m	TargetScan
miR-4468	ENSG00000141252	8m	TargetScan
miR-4468	ENSG00000141298	8m	TargetScan
miR-4468	ENSG00000141337	8m	TargetScan
miR-4468	ENSG00000141441	8m	TargetScan
miR-4468	ENSG00000141560	8m	TargetScan
miR-4468	ENSG00000141622	8m	TargetScan

miR-4468	ENSG00000141759	8m	TargetScan
miR-4468	ENSG00000141965	8m	TargetScan
miR-4468	ENSG00000142166	8m	TargetScan
miR-4468	ENSG00000142556	8m	TargetScan
miR-4468	ENSG00000142623	8m	TargetScan
miR-4468	ENSG00000143178	8m	TargetScan
miR-4468	ENSG00000143297	8m	TargetScan
miR-4468	ENSG00000143498	8m	TargetScan
miR-4468	ENSG00000143537	8m	TargetScan
miR-4468	ENSG00000143595	8m	TargetScan
miR-4468	ENSG00000143603	8m	TargetScan
miR-4468	ENSG00000143627	8m	TargetScan
miR-4468	ENSG00000143771	8m	TargetScan
miR-4468	ENSG00000143889	8m	TargetScan
miR-4468	ENSG00000143921	8m	TargetScan
miR-4468	ENSG00000144320	8m	TargetScan
miR-4468	ENSG00000144580	8m	TargetScan
miR-4468	ENSG00000144645	8m	TargetScan
miR-4468	ENSG00000144711	8m	TargetScan
miR-4468	ENSG00000145214	8m	TargetScan
miR-4468	ENSG00000145287	8m	TargetScan
miR-4468	ENSG00000145391	8m	TargetScan
miR-4468	ENSG00000145439	8m	TargetScan
miR-4468	ENSG00000145555	8m	TargetScan
miR-4468	ENSG00000145685	8m	TargetScan
miR-4468	ENSG00000145725	8m	TargetScan
miR-4468	ENSG00000145850	8m	TargetScan
miR-4468	ENSG00000145861	8m	TargetScan
miR-4468	ENSG00000145920	8m	TargetScan
miR-4468	ENSG00000146063	8m	TargetScan
miR-4468	ENSG00000146147	8m	TargetScan

miR-4468	ENSG00000146216	8m	TargetScan
miR-4468	ENSG00000146267	8m	TargetScan
miR-4468	ENSG00000146535	8m	TargetScan
miR-4468	ENSG00000146648	8m	TargetScan
miR-4468	ENSG00000146670	8m	TargetScan
miR-4468	ENSG00000146757	8m	TargetScan
miR-4468	ENSG00000146859	8m	TargetScan
miR-4468	ENSG00000147117	8m	TargetScan
miR-4468	ENSG00000147138	8m	TargetScan
miR-4468	ENSG00000147234	8m	TargetScan
miR-4468	ENSG00000147246	8m	TargetScan
miR-4468	ENSG00000147394	8m	TargetScan
miR-4468	ENSG00000147535	8m	TargetScan
miR-4468	ENSG00000147548	8m	TargetScan
miR-4468	ENSG00000147649	8m	TargetScan
miR-4468	ENSG00000148082	8m	TargetScan
miR-4468	ENSG00000148110	8m	TargetScan
miR-4468	ENSG00000148120	8m	TargetScan
miR-4468	ENSG00000148158	8m	TargetScan
miR-4468	ENSG00000148248	8m	TargetScan
miR-4468	ENSG00000148634	8m	TargetScan
miR-4468	ENSG00000148688	8m	TargetScan
miR-4468	ENSG00000148734	8m	TargetScan
miR-4468	ENSG00000148798	8m	TargetScan
miR-4468	ENSG00000148942	8m	TargetScan
miR-4468	ENSG00000149196	8m	TargetScan
miR-4468	ENSG00000149212	8m	TargetScan
miR-4468	ENSG00000149218	8m	TargetScan
miR-4468	ENSG00000149256	8m	TargetScan
miR-4468	ENSG00000149257	8m	TargetScan
miR-4468	ENSG00000149346	8m	TargetScan

miR-4468	ENSG00000149507	8m	TargetScan
miR-4468	ENSG00000149596	8m	TargetScan
miR-4468	ENSG00000149782	8m	TargetScan
miR-4468	ENSG00000149925	8m	TargetScan
miR-4468	ENSG00000149927	8m	TargetScan
miR-4468	ENSG00000150457	8m	TargetScan
miR-4468	ENSG00000151090	8m	TargetScan
miR-4468	ENSG00000151164	8m	TargetScan
miR-4468	ENSG00000151176	8m	TargetScan
miR-4468	ENSG00000151553	8m	TargetScan
miR-4468	ENSG00000151914	8m	TargetScan
miR-4468	ENSG00000152078	8m	TargetScan
miR-4468	ENSG00000152102	8m	TargetScan
miR-4468	ENSG00000152270	8m	TargetScan
miR-4468	ENSG00000152284	8m	TargetScan
miR-4468	ENSG00000152404	8m	TargetScan
miR-4468	ENSG00000152443	8m	TargetScan
miR-4468	ENSG00000152580	8m	TargetScan
miR-4468	ENSG00000152689	8m	TargetScan
miR-4468	ENSG00000152932	8m	TargetScan
miR-4468	ENSG00000152953	8m	TargetScan
miR-4468	ENSG00000153012	8m	TargetScan
miR-4468	ENSG00000153233	8m	TargetScan
miR-4468	ENSG00000153303	8m	TargetScan
miR-4468	ENSG00000153904	8m	TargetScan
miR-4468	ENSG00000154001	8m	TargetScan
miR-4468	ENSG00000154124	8m	TargetScan
miR-4468	ENSG00000154342	8m	TargetScan
miR-4468	ENSG00000154511	8m	TargetScan
miR-4468	ENSG00000154582	8m	TargetScan
miR-4468	ENSG00000154655	8m	TargetScan

miR-4468	ENSG00000154678	8m	TargetScan
miR-4468	ENSG00000155008	8m	TargetScan
miR-4468	ENSG00000155034	8m	TargetScan
miR-4468	ENSG00000155066	8m	TargetScan
miR-4468	ENSG00000155111	8m	TargetScan
miR-4468	ENSG00000155115	8m	TargetScan
miR-4468	ENSG00000155158	8m	TargetScan
miR-4468	ENSG00000155438	8m	TargetScan
miR-4468	ENSG00000155506	8m	TargetScan
miR-4468	ENSG00000155542	8m	TargetScan
miR-4468	ENSG00000155816	8m	TargetScan
miR-4468	ENSG00000155827	8m	TargetScan
miR-4468	ENSG00000155833	8m	TargetScan
miR-4468	ENSG00000155886	8m	TargetScan
miR-4468	ENSG00000155926	8m	TargetScan
miR-4468	ENSG00000156011	8m	TargetScan
miR-4468	ENSG00000156172	8m	TargetScan
miR-4468	ENSG00000156500	8m	TargetScan
miR-4468	ENSG00000156873	8m	TargetScan
miR-4468	ENSG00000156928	8m	TargetScan
miR-4468	ENSG00000157036	8m	TargetScan
miR-4468	ENSG00000157150	8m	TargetScan
miR-4468	ENSG00000157326	8m	TargetScan
miR-4468	ENSG00000157388	8m	TargetScan
miR-4468	ENSG00000157483	8m	TargetScan
miR-4468	ENSG00000157542	8m	TargetScan
miR-4468	ENSG00000157978	8m	TargetScan
miR-4468	ENSG00000158022	8m	TargetScan
miR-4468	ENSG00000158109	8m	TargetScan
miR-4468	ENSG00000158161	8m	TargetScan
miR-4468	ENSG00000158258	8m	TargetScan

miR-4468	ENSG00000158427	8m	TargetScan
miR-4468	ENSG00000158467	8m	TargetScan
miR-4468	ENSG00000158473	8m	TargetScan
miR-4468	ENSG00000158711	8m	TargetScan
miR-4468	ENSG00000158828	8m	TargetScan
miR-4468	ENSG00000158955	8m	TargetScan
miR-4468	ENSG00000158985	8m	TargetScan
miR-4468	ENSG00000159140	8m	TargetScan
miR-4468	ENSG00000159788	8m	TargetScan
miR-4468	ENSG00000159882	8m	TargetScan
miR-4468	ENSG00000159885	8m	TargetScan
miR-4468	ENSG00000160049	8m	TargetScan
miR-4468	ENSG00000160058	8m	TargetScan
miR-4468	ENSG00000160208	8m	TargetScan
miR-4468	ENSG00000160209	8m	TargetScan
miR-4468	ENSG00000160223	8m	TargetScan
miR-4468	ENSG00000160293	8m	TargetScan
miR-4468	ENSG00000160325	8m	TargetScan
miR-4468	ENSG00000160339	8m	TargetScan
miR-4468	ENSG00000160716	8m	TargetScan
miR-4468	ENSG00000160781	8m	TargetScan
miR-4468	ENSG00000160908	8m	TargetScan
miR-4468	ENSG00000161010	8m	TargetScan
miR-4468	ENSG00000161204	8m	TargetScan
miR-4468	ENSG00000161381	8m	TargetScan
miR-4468	ENSG00000161791	8m	TargetScan
miR-4468	ENSG00000162065	8m	TargetScan
miR-4468	ENSG00000162510	8m	TargetScan
miR-4468	ENSG00000162522	8m	TargetScan
miR-4468	ENSG00000162552	8m	TargetScan
miR-4468	ENSG00000162571	8m	TargetScan

miR-4468	ENSG00000162607	8m	TargetScan
miR-4468	ENSG00000162631	8m	TargetScan
miR-4468	ENSG00000162645	8m	TargetScan
miR-4468	ENSG00000162733	8m	TargetScan
miR-4468	ENSG00000162738	8m	TargetScan
miR-4468	ENSG00000162804	8m	TargetScan
miR-4468	ENSG00000162888	8m	TargetScan
miR-4468	ENSG00000162889	8m	TargetScan
miR-4468	ENSG00000162924	8m	TargetScan
miR-4468	ENSG00000162929	8m	TargetScan
miR-4468	ENSG00000163138	8m	TargetScan
miR-4468	ENSG00000163145	8m	TargetScan
miR-4468	ENSG00000163155	8m	TargetScan
miR-4468	ENSG00000163393	8m	TargetScan
miR-4468	ENSG00000163412	8m	TargetScan
miR-4468	ENSG00000163513	8m	TargetScan
miR-4468	ENSG00000163586	8m	TargetScan
miR-4468	ENSG00000163590	8m	TargetScan
miR-4468	ENSG00000163673	8m	TargetScan
miR-4468	ENSG00000163870	8m	TargetScan
miR-4468	ENSG00000163888	8m	TargetScan
miR-4468	ENSG00000163909	8m	TargetScan
miR-4468	ENSG00000164007	8m	TargetScan
miR-4468	ENSG00000164038	8m	TargetScan
miR-4468	ENSG00000164061	8m	TargetScan
miR-4468	ENSG00000164070	8m	TargetScan
miR-4468	ENSG00000164076	8m	TargetScan
miR-4468	ENSG00000164091	8m	TargetScan
miR-4468	ENSG00000164114	8m	TargetScan
miR-4468	ENSG00000164116	8m	TargetScan
miR-4468	ENSG00000164120	8m	TargetScan

miR-4468	ENSG00000164241	8m	TargetScan
miR-4468	ENSG00000164393	8m	TargetScan
miR-4468	ENSG00000164402	8m	TargetScan
miR-4468	ENSG00000164574	8m	TargetScan
miR-4468	ENSG00000164742	8m	TargetScan
miR-4468	ENSG00000164751	8m	TargetScan
miR-4468	ENSG00000164916	8m	TargetScan
miR-4468	ENSG00000164944	8m	TargetScan
miR-4468	ENSG00000164970	8m	TargetScan
miR-4468	ENSG00000165028	8m	TargetScan
miR-4468	ENSG00000165029	8m	TargetScan
miR-4468	ENSG00000165238	8m	TargetScan
miR-4468	ENSG00000165240	8m	TargetScan
miR-4468	ENSG00000165269	8m	TargetScan
miR-4468	ENSG00000165288	8m	TargetScan
miR-4468	ENSG00000165410	8m	TargetScan
miR-4468	ENSG00000165568	8m	TargetScan
miR-4468	ENSG00000165650	8m	TargetScan
miR-4468	ENSG00000165671	8m	TargetScan
miR-4468	ENSG00000165886	8m	TargetScan
miR-4468	ENSG00000166012	8m	TargetScan
miR-4468	ENSG00000166049	8m	TargetScan
miR-4468	ENSG00000166105	8m	TargetScan
miR-4468	ENSG00000166145	8m	TargetScan
miR-4468	ENSG00000166147	8m	TargetScan
miR-4468	ENSG00000166159	8m	TargetScan
miR-4468	ENSG00000166233	8m	TargetScan
miR-4468	ENSG00000166265	8m	TargetScan
miR-4468	ENSG00000166295	8m	TargetScan
miR-4468	ENSG00000166326	8m	TargetScan
miR-4468	ENSG00000166347	8m	TargetScan

miR-4468	ENSG00000166401	8m	TargetScan
miR-4468	ENSG00000166432	8m	TargetScan
miR-4468	ENSG00000166557	8m	TargetScan
miR-4468	ENSG00000166598	8m	TargetScan
miR-4468	ENSG00000166685	8m	TargetScan
miR-4468	ENSG00000166823	8m	TargetScan
miR-4468	ENSG00000166847	8m	TargetScan
miR-4468	ENSG00000166887	8m	TargetScan
miR-4468	ENSG00000166923	8m	TargetScan
miR-4468	ENSG00000166946	8m	TargetScan
miR-4468	ENSG00000166961	8m	TargetScan
miR-4468	ENSG00000167004	8m	TargetScan
miR-4468	ENSG00000167005	8m	TargetScan
miR-4468	ENSG00000167074	8m	TargetScan
miR-4468	ENSG00000167106	8m	TargetScan
miR-4468	ENSG00000167283	8m	TargetScan
miR-4468	ENSG00000167380	8m	TargetScan
miR-4468	ENSG00000167770	8m	TargetScan
miR-4468	ENSG00000167858	8m	TargetScan
miR-4468	ENSG00000167968	8m	TargetScan
miR-4468	ENSG00000167971	8m	TargetScan
miR-4468	ENSG00000168067	8m	TargetScan
miR-4468	ENSG00000168214	8m	TargetScan
miR-4468	ENSG00000168228	8m	TargetScan
miR-4468	ENSG00000168234	8m	TargetScan
miR-4468	ENSG00000168314	8m	TargetScan
miR-4468	ENSG00000168418	8m	TargetScan
miR-4468	ENSG00000168461	8m	TargetScan
miR-4468	ENSG00000168481	8m	TargetScan
miR-4468	ENSG00000168487	8m	TargetScan
miR-4468	ENSG00000168612	8m	TargetScan

miR-4468	ENSG00000168758	8m	TargetScan
miR-4468	ENSG00000168785	8m	TargetScan
miR-4468	ENSG00000168792	8m	TargetScan
miR-4468	ENSG00000168827	8m	TargetScan
miR-4468	ENSG00000168887	8m	TargetScan
miR-4468	ENSG00000168994	8m	TargetScan
miR-4468	ENSG00000169188	8m	TargetScan
miR-4468	ENSG00000169247	8m	TargetScan
miR-4468	ENSG00000169291	8m	TargetScan
miR-4468	ENSG00000169302	8m	TargetScan
miR-4468	ENSG00000169403	8m	TargetScan
miR-4468	ENSG00000169594	8m	TargetScan
miR-4468	ENSG00000169641	8m	TargetScan
miR-4468	ENSG00000169679	8m	TargetScan
miR-4468	ENSG00000169692	8m	TargetScan
miR-4468	ENSG00000169814	8m	TargetScan
miR-4468	ENSG00000169981	8m	TargetScan
miR-4468	ENSG00000170100	8m	TargetScan
miR-4468	ENSG00000170113	8m	TargetScan
miR-4468	ENSG00000170180	8m	TargetScan
miR-4468	ENSG00000170222	8m	TargetScan
miR-4468	ENSG00000170464	8m	TargetScan
miR-4468	ENSG00000170522	8m	TargetScan
miR-4468	ENSG00000170558	8m	TargetScan
miR-4468	ENSG00000170624	8m	TargetScan
miR-4468	ENSG00000170632	8m	TargetScan
miR-4468	ENSG00000170634	8m	TargetScan
miR-4468	ENSG00000170653	8m	TargetScan
miR-4468	ENSG00000170734	8m	TargetScan
miR-4468	ENSG00000170743	8m	TargetScan
miR-4468	ENSG00000170748	8m	TargetScan

miR-4468	ENSG00000170854	8m	TargetScan
miR-4468	ENSG00000170903	8m	TargetScan
miR-4468	ENSG00000171004	8m	TargetScan
miR-4468	ENSG00000171033	8m	TargetScan
miR-4468	ENSG00000171262	8m	TargetScan
miR-4468	ENSG00000171320	8m	TargetScan
miR-4468	ENSG00000171365	8m	TargetScan
miR-4468	ENSG00000171451	8m	TargetScan
miR-4468	ENSG00000171496	8m	TargetScan
miR-4468	ENSG00000171621	8m	TargetScan
miR-4468	ENSG00000172058	8m	TargetScan
miR-4468	ENSG00000172339	8m	TargetScan
miR-4468	ENSG00000172500	8m	TargetScan
miR-4468	ENSG00000172508	8m	TargetScan
miR-4468	ENSG00000172671	8m	TargetScan
miR-4468	ENSG00000172687	8m	TargetScan
miR-4468	ENSG00000172818	8m	TargetScan
miR-4468	ENSG00000172830	8m	TargetScan
miR-4468	ENSG00000172987	8m	TargetScan
miR-4468	ENSG00000173064	8m	TargetScan
miR-4468	ENSG00000173065	8m	TargetScan
miR-4468	ENSG00000173157	8m	TargetScan
miR-4468	ENSG00000173218	8m	TargetScan
miR-4468	ENSG00000173227	8m	TargetScan
miR-4468	ENSG00000173281	8m	TargetScan
miR-4468	ENSG00000173548	8m	TargetScan
miR-4468	ENSG00000173559	8m	TargetScan
miR-4468	ENSG00000173706	8m	TargetScan
miR-4468	ENSG00000173889	8m	TargetScan
miR-4468	ENSG00000174099	8m	TargetScan
miR-4468	ENSG00000174151	8m	TargetScan

miR-4468	ENSG00000174197	8m	TargetScan
miR-4468	ENSG00000174498	8m	TargetScan
miR-4468	ENSG00000174574	8m	TargetScan
miR-4468	ENSG00000174705	8m	TargetScan
miR-4468	ENSG00000174748	8m	TargetScan
miR-4468	ENSG00000174840	8m	TargetScan
miR-4468	ENSG00000174885	8m	TargetScan
miR-4468	ENSG00000174903	8m	TargetScan
miR-4468	ENSG00000174953	8m	TargetScan
miR-4468	ENSG00000175029	8m	TargetScan
miR-4468	ENSG00000175106	8m	TargetScan
miR-4468	ENSG00000175216	8m	TargetScan
miR-4468	ENSG00000175497	8m	TargetScan
miR-4468	ENSG00000175556	8m	TargetScan
miR-4468	ENSG00000175728	8m	TargetScan
miR-4468	ENSG00000175970	8m	TargetScan
miR-4468	ENSG00000176014	8m	TargetScan
miR-4468	ENSG00000176209	8m	TargetScan
miR-4468	ENSG00000176406	8m	TargetScan
miR-4468	ENSG00000176641	8m	TargetScan
miR-4468	ENSG00000176658	8m	TargetScan
miR-4468	ENSG00000176723	8m	TargetScan
miR-4468	ENSG00000176890	8m	TargetScan
miR-4468	ENSG00000176945	8m	TargetScan
miR-4468	ENSG00000176973	8m	TargetScan
miR-4468	ENSG00000176994	8m	TargetScan
miR-4468	ENSG00000177096	8m	TargetScan
miR-4468	ENSG00000177324	8m	TargetScan
miR-4468	ENSG00000177426	8m	TargetScan
miR-4468	ENSG00000177479	8m	TargetScan
miR-4468	ENSG00000177511	8m	TargetScan

miR-4468	ENSG00000177646	8m	TargetScan
miR-4468	ENSG00000177663	8m	TargetScan
miR-4468	ENSG00000177842	8m	TargetScan
miR-4468	ENSG00000178053	8m	TargetScan
miR-4468	ENSG00000178233	8m	TargetScan
miR-4468	ENSG00000178381	8m	TargetScan
miR-4468	ENSG00000178385	8m	TargetScan
miR-4468	ENSG00000178460	8m	TargetScan
miR-4468	ENSG00000178538	8m	TargetScan
miR-4468	ENSG00000178562	8m	TargetScan
miR-4468	ENSG00000178662	8m	TargetScan
miR-4468	ENSG00000178695	8m	TargetScan
miR-4468	ENSG00000178726	8m	TargetScan
miR-4468	ENSG00000178997	8m	TargetScan
miR-4468	ENSG00000179151	8m	TargetScan
miR-4468	ENSG00000179195	8m	TargetScan
miR-4468	ENSG00000179361	8m	TargetScan
miR-4468	ENSG00000179583	8m	TargetScan
miR-4468	ENSG00000179588	8m	TargetScan
miR-4468	ENSG00000179698	8m	TargetScan
miR-4468	ENSG00000179833	8m	TargetScan
miR-4468	ENSG00000179886	8m	TargetScan
miR-4468	ENSG00000180228	8m	TargetScan
miR-4468	ENSG00000180287	8m	TargetScan
miR-4468	ENSG00000180354	8m	TargetScan
miR-4468	ENSG00000180357	8m	TargetScan
miR-4468	ENSG00000180432	8m	TargetScan
miR-4468	ENSG00000180479	8m	TargetScan
miR-4468	ENSG00000180530	8m	TargetScan
miR-4468	ENSG00000181016	8m	TargetScan
miR-4468	ENSG00000181381	8m	TargetScan

miR-4468	ENSG00000181722	8m	TargetScan
miR-4468	ENSG00000181873	8m	TargetScan
miR-4468	ENSG00000181894	8m	TargetScan
miR-4468	ENSG00000182141	8m	TargetScan
miR-4468	ENSG00000182489	8m	TargetScan
miR-4468	ENSG00000182667	8m	TargetScan
miR-4468	ENSG00000182749	8m	TargetScan
miR-4468	ENSG00000182768	8m	TargetScan
miR-4468	ENSG00000182898	8m	TargetScan
miR-4468	ENSG00000182919	8m	TargetScan
miR-4468	ENSG00000183250	8m	TargetScan
miR-4468	ENSG00000183260	8m	TargetScan
miR-4468	ENSG00000183287	8m	TargetScan
miR-4468	ENSG00000183379	8m	TargetScan
miR-4468	ENSG00000183527	8m	TargetScan
miR-4468	ENSG00000183578	8m	TargetScan
miR-4468	ENSG00000183624	8m	TargetScan
miR-4468	ENSG00000183778	8m	TargetScan
miR-4468	ENSG00000183850	8m	TargetScan
miR-4468	ENSG00000183943	8m	TargetScan
miR-4468	ENSG00000184005	8m	TargetScan
miR-4468	ENSG00000184083	8m	TargetScan
miR-4468	ENSG00000184304	8m	TargetScan
miR-4468	ENSG00000184347	8m	TargetScan
miR-4468	ENSG00000184378	8m	TargetScan
miR-4468	ENSG00000184497	8m	TargetScan
miR-4468	ENSG00000184661	8m	TargetScan
miR-4468	ENSG00000184863	8m	TargetScan
miR-4468	ENSG00000185008	8m	TargetScan
miR-4468	ENSG00000185104	8m	TargetScan
miR-4468	ENSG00000185105	8m	TargetScan

miR-4468	ENSG00000185187	8m	TargetScan
miR-4468	ENSG00000185404	8m	TargetScan
miR-4468	ENSG00000185442	8m	TargetScan
miR-4468	ENSG00000185651	8m	TargetScan
miR-4468	ENSG00000185658	8m	TargetScan
miR-4468	ENSG00000185722	8m	TargetScan
miR-4468	ENSG00000185811	8m	TargetScan
miR-4468	ENSG00000185838	8m	TargetScan
miR-4468	ENSG00000185862	8m	TargetScan
miR-4468	ENSG00000185875	8m	TargetScan
miR-4468	ENSG00000185905	8m	TargetScan
miR-4468	ENSG00000185920	8m	TargetScan
miR-4468	ENSG00000186001	8m	TargetScan
miR-4468	ENSG00000186260	8m	TargetScan
miR-4468	ENSG00000186340	8m	TargetScan
miR-4468	ENSG00000186432	8m	TargetScan
miR-4468	ENSG00000186472	8m	TargetScan
miR-4468	ENSG00000186479	8m	TargetScan
miR-4468	ENSG00000186529	8m	TargetScan
miR-4468	ENSG00000186562	8m	TargetScan
miR-4468	ENSG00000186575	8m	TargetScan
miR-4468	ENSG00000186599	8m	TargetScan
miR-4468	ENSG00000186812	8m	TargetScan
miR-4468	ENSG00000186815	8m	TargetScan
miR-4468	ENSG00000187257	8m	TargetScan
miR-4468	ENSG00000187272	8m	TargetScan
miR-4468	ENSG00000187550	8m	TargetScan
miR-4468	ENSG00000187569	8m	TargetScan
miR-4468	ENSG00000187626	8m	TargetScan
miR-4468	ENSG00000187630	8m	TargetScan
miR-4468	ENSG00000187678	8m	TargetScan

miR-4468	ENSG00000187758	8m	TargetScan
miR-4468	ENSG00000187902	8m	TargetScan
miR-4468	ENSG00000188001	8m	TargetScan
miR-4468	ENSG00000188026	8m	TargetScan
miR-4468	ENSG00000188177	8m	TargetScan
miR-4468	ENSG00000188295	8m	TargetScan
miR-4468	ENSG00000188690	8m	TargetScan
miR-4468	ENSG00000188706	8m	TargetScan
miR-4468	ENSG00000188817	8m	TargetScan
miR-4468	ENSG00000188848	8m	TargetScan
miR-4468	ENSG00000188883	8m	TargetScan
miR-4468	ENSG00000189221	8m	TargetScan
miR-4468	ENSG00000189403	8m	TargetScan
miR-4468	ENSG00000196090	8m	TargetScan
miR-4468	ENSG00000196091	8m	TargetScan
miR-4468	ENSG00000196208	8m	TargetScan
miR-4468	ENSG00000196247	8m	TargetScan
miR-4468	ENSG00000196268	8m	TargetScan
miR-4468	ENSG00000196376	8m	TargetScan
miR-4468	ENSG00000196705	8m	TargetScan
miR-4468	ENSG00000196715	8m	TargetScan
miR-4468	ENSG00000197121	8m	TargetScan
miR-4468	ENSG00000197261	8m	TargetScan
miR-4468	ENSG00000197302	8m	TargetScan
miR-4468	ENSG00000197536	8m	TargetScan
miR-4468	ENSG00000197714	8m	TargetScan
miR-4468	ENSG00000197818	8m	TargetScan
miR-4468	ENSG00000197965	8m	TargetScan
miR-4468	ENSG00000198039	8m	TargetScan
miR-4468	ENSG00000198053	8m	TargetScan
miR-4468	ENSG00000198083	8m	TargetScan

miR-4468	ENSG00000198363	8m	TargetScan
miR-4468	ENSG00000198408	8m	TargetScan
miR-4468	ENSG00000198483	8m	TargetScan
miR-4468	ENSG00000198625	8m	TargetScan
miR-4468	ENSG00000198663	8m	TargetScan
miR-4468	ENSG00000198677	8m	TargetScan
miR-4468	ENSG00000198689	8m	TargetScan
miR-4468	ENSG00000198768	8m	TargetScan
miR-4468	ENSG00000198855	8m	TargetScan
miR-4468	ENSG00000198863	8m	TargetScan
miR-4468	ENSG00000198865	8m	TargetScan
miR-4468	ENSG00000198883	8m	TargetScan
miR-4468	ENSG00000198944	8m	TargetScan
miR-4468	ENSG00000198948	8m	TargetScan
miR-4468	ENSG00000203778	8m	TargetScan
miR-4468	ENSG00000204228	8m	TargetScan
miR-4468	ENSG00000204308	8m	TargetScan
miR-4468	ENSG00000204345	8m	TargetScan
miR-4468	ENSG00000204420	8m	TargetScan
miR-4468	ENSG00000204438	8m	TargetScan
miR-4468	ENSG00000204604	8m	TargetScan
miR-4468	ENSG00000204613	8m	TargetScan
miR-4468	ENSG00000204681	8m	TargetScan
miR-4468	ENSG00000204839	8m	TargetScan
miR-4468	ENSG00000204954	8m	TargetScan
miR-4468	ENSG00000204977	8m	TargetScan
miR-4468	ENSG00000205212	8m	TargetScan
miR-4468	ENSG00000205572	8m	TargetScan
miR-4468	ENSG00000205593	8m	TargetScan
miR-4468	ENSG00000205791	8m	TargetScan
miR-4468	ENSG00000205808	8m	TargetScan

miR-4468	ENSG00000206047	8m	TargetScan
miR-4468	ENSG00000206053	8m	TargetScan
miR-4468	ENSG00000212659	8m	TargetScan
miR-4468	ENSG00000212722	8m	TargetScan
miR-4468	ENSG00000213064	8m	TargetScan
miR-4468	ENSG00000213672	8m	TargetScan
miR-4468	ENSG00000213886	8m	TargetScan
miR-4468	ENSG00000213967	8m	TargetScan
miR-4468	ENSG00000213973	8m	TargetScan
miR-4468	ENSG00000213988	8m	TargetScan
miR-4468	ENSG00000214050	8m	TargetScan
miR-4468	ENSG00000214357	8m	TargetScan
miR-4468	ENSG00000214530	8m	TargetScan
miR-4468	ENSG00000215114	8m	TargetScan
miR-4468	ENSG00000220891	8m	TargetScan
miR-4468	ENSG00000221823	8m	TargetScan
miR-4468	ENSG00000221845	8m	TargetScan
miR-4468	ENSG00000221963	8m	TargetScan
miR-4468	ENSG00000222001	8m	TargetScan
miR-4468	ENSG00000223572	8m	TargetScan
miR-4468	ENSG00000223865	8m	TargetScan
miR-4468	ENSG00000225697	8m	TargetScan
miR-4468	ENSG00000225830	8m	TargetScan
miR-4468	ENSG00000227051	8m	TargetScan
miR-4468	ENSG00000228075	8m	TargetScan
miR-4468	ENSG00000232040	8m	TargetScan
miR-4468	ENSG00000232119	8m	TargetScan
miR-4468	ENSG00000233024	8m	TargetScan
miR-4468	ENSG00000235750	8m	TargetScan
miR-4468	ENSG00000236279	8m	TargetScan
miR-4468	ENSG00000237289	8m	TargetScan

miR-4468	ENSG00000237440	8m	TargetScan
miR-4468	ENSG00000239306	8m	TargetScan
miR-4468	ENSG00000239839	8m	TargetScan
miR-4468	ENSG00000239886	8m	TargetScan
miR-4468	ENSG00000240053	8m	TargetScan
miR-4468	ENSG00000240247	8m	TargetScan
miR-4468	ENSG00000240694	8m	TargetScan
miR-4468	ENSG00000241595	8m	TargetScan
miR-4468	ENSG00000245680	8m	TargetScan
miR-4468	ENSG00000247746	8m	TargetScan
miR-4468	ENSG00000249884	8m	TargetScan
miR-4468	ENSG00000250091	8m	TargetScan
miR-4468	ENSG00000254087	8m	TargetScan
miR-4468	ENSG00000255112	8m	TargetScan
miR-4468	ENSG00000257704	8m	TargetScan
miR-4468	ENSG00000257923	8m	TargetScan
miR-4468	ENSG00000259207	8m	TargetScan
miR-4468	ENSG00000260007	8m	TargetScan
miR-4468	ENSG00000260548	8m	TargetScan
miR-4468	ENSG00000263020	8m	TargetScan
miR-4468	ENSG00000267432	8m	TargetScan
miR-4468	ENSG00000269343	8m	TargetScan
miR-4468	ENSG00000272195	8m	TargetScan
miR-4468	ENSG00000273018	8m	TargetScan
miR-4468	ENSG00000273802	8m	TargetScan
miR-4468	ENSG00000284448	8m	TargetScan
miR-4468	ENSG00000285043	8m	TargetScan
miR-4468	ENSG00000285347	8m	TargetScan
miR-4468	ENSG00000286053	8m	TargetScan
miR-4468	ENSG00000288436	8m	TargetScan
miR-4788	ENSG00000099308	8m	TargetScan

miR-4788	ENSG00000099940	8m	TargetScan
miR-4788	ENSG00000100350	8m	TargetScan
miR-4788	ENSG00000100416	8m	TargetScan
miR-4788	ENSG00000104524	8m	TargetScan
miR-4788	ENSG00000107130	8m	TargetScan
miR-4788	ENSG00000107282	8m	TargetScan
miR-4788	ENSG00000108176	8m	TargetScan
miR-4788	ENSG00000108924	8m	TargetScan
miR-4788	ENSG00000109171	8m	TargetScan
miR-4788	ENSG00000112742	8m	TargetScan
miR-4788	ENSG00000115020	8m	TargetScan
miR-4788	ENSG00000116120	8m	TargetScan
miR-4788	ENSG00000118705	8m	TargetScan
miR-4788	ENSG00000127329	8m	TargetScan
miR-4788	ENSG00000132294	8m	TargetScan
miR-4788	ENSG00000134202	8m	TargetScan
miR-4788	ENSG00000135272	8m	TargetScan
miR-4788	ENSG00000137185	8m	TargetScan
miR-4788	ENSG00000140950	8m	TargetScan
miR-4788	ENSG00000143158	8m	TargetScan
miR-4788	ENSG00000143322	8m	TargetScan
miR-4788	ENSG00000144619	8m	TargetScan
miR-4788	ENSG00000144840	8m	TargetScan
miR-4788	ENSG00000145833	8m	TargetScan
miR-4788	ENSG00000146802	8m	TargetScan
miR-4788	ENSG00000151151	8m	TargetScan
miR-4788	ENSG00000152684	8m	TargetScan
miR-4788	ENSG00000164237	8m	TargetScan
miR-4788	ENSG00000164808	8m	TargetScan
miR-4788	ENSG00000167487	8m	TargetScan
miR-4788	ENSG00000169567	8m	TargetScan

miR-4788	ENSG00000171848	8m	TargetScan
miR-4788	ENSG00000180354	8m	TargetScan
miR-4788	ENSG00000183166	8m	TargetScan
miR-4788	ENSG00000184378	8m	TargetScan
miR-4788	ENSG00000189051	8m	TargetScan
miR-4788	ENSG00000196616	8m	TargetScan
miR-4788	ENSG00000198492	8m	TargetScan
miR-4788	ENSG00000215014	8m	TargetScan
miR-4788	ENSG00000224877	8m	TargetScan
miR-4788	ENSG00000251322	8m	TargetScan
miR-492	ENSG00000003402	8m	TargetScan
miR-492	ENSG00000005156	8m	TargetScan
miR-492	ENSG00000006432	8m	TargetScan
miR-492	ENSG00000006453	8m	TargetScan
miR-492	ENSG00000006740	8m	TargetScan
miR-492	ENSG00000007541	8m	TargetScan
miR-492	ENSG00000007923	8m	TargetScan
miR-492	ENSG00000011523	8m	TargetScan
miR-492	ENSG00000011677	8m	TargetScan
miR-492	ENSG00000012223	8m	TargetScan
miR-492	ENSG00000014919	8m	TargetScan
miR-492	ENSG00000015676	8m	TargetScan
miR-492	ENSG00000019995	8m	TargetScan
miR-492	ENSG00000020129	8m	TargetScan
miR-492	ENSG00000021645	8m	TargetScan
miR-492	ENSG00000026508	8m	TargetScan
miR-492	ENSG00000026652	8m	TargetScan
miR-492	ENSG00000033867	8m	TargetScan
miR-492	ENSG00000034677	8m	TargetScan
miR-492	ENSG00000035403	8m	TargetScan
miR-492	ENSG00000036448	8m	TargetScan

miR-492	ENSG00000048544	8m	TargetScan
miR-492	ENSG00000050555	8m	TargetScan
miR-492	ENSG00000050748	8m	TargetScan
miR-492	ENSG00000051620	8m	TargetScan
miR-492	ENSG00000054965	8m	TargetScan
miR-492	ENSG00000059122	8m	TargetScan
miR-492	ENSG00000063245	8m	TargetScan
miR-492	ENSG00000064199	8m	TargetScan
miR-492	ENSG00000064393	8m	TargetScan
miR-492	ENSG00000070193	8m	TargetScan
miR-492	ENSG00000070526	8m	TargetScan
miR-492	ENSG00000070614	8m	TargetScan
miR-492	ENSG00000070886	8m	TargetScan
miR-492	ENSG00000070915	8m	TargetScan
miR-492	ENSG00000072133	8m	TargetScan
miR-492	ENSG00000073670	8m	TargetScan
miR-492	ENSG00000073849	8m	TargetScan
miR-492	ENSG00000075914	8m	TargetScan
miR-492	ENSG00000076356	8m	TargetScan
miR-492	ENSG00000076641	8m	TargetScan
miR-492	ENSG00000078814	8m	TargetScan
miR-492	ENSG00000079308	8m	TargetScan
miR-492	ENSG00000081138	8m	TargetScan
miR-492	ENSG00000081386	8m	TargetScan
miR-492	ENSG00000082684	8m	TargetScan
miR-492	ENSG00000083223	8m	TargetScan
miR-492	ENSG00000083444	8m	TargetScan
miR-492	ENSG00000084090	8m	TargetScan
miR-492	ENSG00000084628	8m	TargetScan
miR-492	ENSG00000085117	8m	TargetScan
miR-492	ENSG00000085721	8m	TargetScan

miR-492	ENSG00000085831	8m	TargetScan
miR-492	ENSG00000086062	8m	TargetScan
miR-492	ENSG00000087191	8m	TargetScan
miR-492	ENSG00000088812	8m	TargetScan
miR-492	ENSG00000090889	8m	TargetScan
miR-492	ENSG00000092445	8m	TargetScan
miR-492	ENSG00000095539	8m	TargetScan
miR-492	ENSG00000099385	8m	TargetScan
miR-492	ENSG00000099625	8m	TargetScan
miR-492	ENSG00000099917	8m	TargetScan
miR-492	ENSG00000100241	8m	TargetScan
miR-492	ENSG00000100296	8m	TargetScan
miR-492	ENSG00000100341	8m	TargetScan
miR-492	ENSG00000100359	8m	TargetScan
miR-492	ENSG00000100441	8m	TargetScan
miR-492	ENSG00000101213	8m	TargetScan
miR-492	ENSG00000101255	8m	TargetScan
miR-492	ENSG00000101290	8m	TargetScan
miR-492	ENSG00000101444	8m	TargetScan
miR-492	ENSG00000101445	8m	TargetScan
miR-492	ENSG00000101849	8m	TargetScan
miR-492	ENSG00000101892	8m	TargetScan
miR-492	ENSG00000102362	8m	TargetScan
miR-492	ENSG00000102385	8m	TargetScan
miR-492	ENSG00000102763	8m	TargetScan
miR-492	ENSG00000102858	8m	TargetScan
miR-492	ENSG00000102967	8m	TargetScan
miR-492	ENSG00000103064	8m	TargetScan
miR-492	ENSG00000103067	8m	TargetScan
miR-492	ENSG00000103196	8m	TargetScan
miR-492	ENSG00000103275	8m	TargetScan

miR-492	ENSG00000103363	8m	TargetScan
miR-492	ENSG00000103365	8m	TargetScan
miR-492	ENSG00000103550	8m	TargetScan
miR-492	ENSG00000103888	8m	TargetScan
miR-492	ENSG00000104332	8m	TargetScan
miR-492	ENSG00000104805	8m	TargetScan
miR-492	ENSG00000104884	8m	TargetScan
miR-492	ENSG00000105063	8m	TargetScan
miR-492	ENSG00000105429	8m	TargetScan
miR-492	ENSG00000105707	8m	TargetScan
miR-492	ENSG00000106236	8m	TargetScan
miR-492	ENSG00000106246	8m	TargetScan
miR-492	ENSG00000106477	8m	TargetScan
miR-492	ENSG00000106665	8m	TargetScan
miR-492	ENSG00000107185	8m	TargetScan
miR-492	ENSG00000107282	8m	TargetScan
miR-492	ENSG00000107338	8m	TargetScan
miR-492	ENSG00000108306	8m	TargetScan
miR-492	ENSG00000108375	8m	TargetScan
miR-492	ENSG00000108684	8m	TargetScan
miR-492	ENSG00000108823	8m	TargetScan
miR-492	ENSG00000108852	8m	TargetScan
miR-492	ENSG00000109189	8m	TargetScan
miR-492	ENSG00000109572	8m	TargetScan
miR-492	ENSG00000109906	8m	TargetScan
miR-492	ENSG00000110063	8m	TargetScan
miR-492	ENSG00000110245	8m	TargetScan
miR-492	ENSG00000110429	8m	TargetScan
miR-492	ENSG00000110665	8m	TargetScan
miR-492	ENSG00000110711	8m	TargetScan
miR-492	ENSG00000110876	8m	TargetScan

miR-492	ENSG00000110906	8m	TargetScan
miR-492	ENSG00000111262	8m	TargetScan
miR-492	ENSG00000111331	8m	TargetScan
miR-492	ENSG00000111671	8m	TargetScan
miR-492	ENSG00000111886	8m	TargetScan
miR-492	ENSG00000112149	8m	TargetScan
miR-492	ENSG00000112182	8m	TargetScan
miR-492	ENSG00000112208	8m	TargetScan
miR-492	ENSG00000112902	8m	TargetScan
miR-492	ENSG00000113269	8m	TargetScan
miR-492	ENSG00000113300	8m	TargetScan
miR-492	ENSG00000113504	8m	TargetScan
miR-492	ENSG00000113600	8m	TargetScan
miR-492	ENSG00000113971	8m	TargetScan
miR-492	ENSG00000114126	8m	TargetScan
miR-492	ENSG00000114745	8m	TargetScan
miR-492	ENSG00000114904	8m	TargetScan
miR-492	ENSG00000115194	8m	TargetScan
miR-492	ENSG00000115325	8m	TargetScan
miR-492	ENSG00000115368	8m	TargetScan
miR-492	ENSG00000115461	8m	TargetScan
miR-492	ENSG00000115808	8m	TargetScan
miR-492	ENSG00000115884	8m	TargetScan
miR-492	ENSG00000116147	8m	TargetScan
miR-492	ENSG00000116260	8m	TargetScan
miR-492	ENSG00000116667	8m	TargetScan
miR-492	ENSG00000117298	8m	TargetScan
miR-492	ENSG00000117480	8m	TargetScan
miR-492	ENSG00000118271	8m	TargetScan
miR-492	ENSG00000119314	8m	TargetScan
miR-492	ENSG00000119411	8m	TargetScan

miR-492	ENSG00000119547	8m	TargetScan
miR-492	ENSG00000120549	8m	TargetScan
miR-492	ENSG00000120833	8m	TargetScan
miR-492	ENSG00000120907	8m	TargetScan
miR-492	ENSG00000121406	8m	TargetScan
miR-492	ENSG00000122678	8m	TargetScan
miR-492	ENSG00000122783	8m	TargetScan
miR-492	ENSG00000123353	8m	TargetScan
miR-492	ENSG00000123684	8m	TargetScan
miR-492	ENSG00000123908	8m	TargetScan
miR-492	ENSG00000123933	8m	TargetScan
miR-492	ENSG00000124164	8m	TargetScan
miR-492	ENSG00000124249	8m	TargetScan
miR-492	ENSG00000124251	8m	TargetScan
miR-492	ENSG00000125484	8m	TargetScan
miR-492	ENSG00000125869	8m	TargetScan
miR-492	ENSG00000125952	8m	TargetScan
miR-492	ENSG00000125965	8m	TargetScan
miR-492	ENSG00000126003	8m	TargetScan
miR-492	ENSG00000126217	8m	TargetScan
miR-492	ENSG00000126218	8m	TargetScan
miR-492	ENSG00000126785	8m	TargetScan
miR-492	ENSG00000127334	8m	TargetScan
miR-492	ENSG00000127564	8m	TargetScan
miR-492	ENSG00000127946	8m	TargetScan
miR-492	ENSG00000128203	8m	TargetScan
miR-492	ENSG00000128284	8m	TargetScan
miR-492	ENSG00000128487	8m	TargetScan
miR-492	ENSG00000128609	8m	TargetScan
miR-492	ENSG00000128713	8m	TargetScan
miR-492	ENSG00000128872	8m	TargetScan

miR-492	ENSG00000128908	8m	TargetScan
miR-492	ENSG00000129007	8m	TargetScan
miR-492	ENSG00000129295	8m	TargetScan
miR-492	ENSG00000129353	8m	TargetScan
miR-492	ENSG00000129654	8m	TargetScan
miR-492	ENSG00000130309	8m	TargetScan
miR-492	ENSG00000130592	8m	TargetScan
miR-492	ENSG00000130695	8m	TargetScan
miR-492	ENSG00000130720	8m	TargetScan
miR-492	ENSG00000130723	8m	TargetScan
miR-492	ENSG00000130749	8m	TargetScan
miR-492	ENSG00000130856	8m	TargetScan
miR-492	ENSG00000130956	8m	TargetScan
miR-492	ENSG00000131044	8m	TargetScan
miR-492	ENSG00000131408	8m	TargetScan
miR-492	ENSG00000132693	8m	TargetScan
miR-492	ENSG00000132953	8m	TargetScan
miR-492	ENSG00000133030	8m	TargetScan
miR-492	ENSG00000133055	8m	TargetScan
miR-492	ENSG00000134248	8m	TargetScan
miR-492	ENSG00000134531	8m	TargetScan
miR-492	ENSG00000135297	8m	TargetScan
miR-492	ENSG00000135299	8m	TargetScan
miR-492	ENSG00000135372	8m	TargetScan
miR-492	ENSG00000135374	8m	TargetScan
miR-492	ENSG00000135414	8m	TargetScan
miR-492	ENSG00000135525	8m	TargetScan
miR-492	ENSG00000135898	8m	TargetScan
miR-492	ENSG00000136026	8m	TargetScan
miR-492	ENSG00000136155	8m	TargetScan
miR-492	ENSG00000136286	8m	TargetScan

miR-492	ENSG00000136527	8m	TargetScan
miR-492	ENSG00000136718	8m	TargetScan
miR-492	ENSG00000136720	8m	TargetScan
miR-492	ENSG00000136877	8m	TargetScan
miR-492	ENSG00000137077	8m	TargetScan
miR-492	ENSG00000137094	8m	TargetScan
miR-492	ENSG00000137106	8m	TargetScan
miR-492	ENSG00000137135	8m	TargetScan
miR-492	ENSG00000137171	8m	TargetScan
miR-492	ENSG00000137834	8m	TargetScan
miR-492	ENSG00000137959	8m	TargetScan
miR-492	ENSG00000138380	8m	TargetScan
miR-492	ENSG00000138443	8m	TargetScan
miR-492	ENSG00000139990	8m	TargetScan
miR-492	ENSG00000140015	8m	TargetScan
miR-492	ENSG00000140368	8m	TargetScan
miR-492	ENSG00000140650	8m	TargetScan
miR-492	ENSG00000140876	8m	TargetScan
miR-492	ENSG00000140987	8m	TargetScan
miR-492	ENSG00000141252	8m	TargetScan
miR-492	ENSG00000141404	8m	TargetScan
miR-492	ENSG00000141446	8m	TargetScan
miR-492	ENSG00000141519	8m	TargetScan
miR-492	ENSG00000141526	8m	TargetScan
miR-492	ENSG00000141580	8m	TargetScan
miR-492	ENSG00000142661	8m	TargetScan
miR-492	ENSG00000142677	8m	TargetScan
miR-492	ENSG00000143374	8m	TargetScan
miR-492	ENSG00000143376	8m	TargetScan
miR-492	ENSG00000143603	8m	TargetScan
miR-492	ENSG00000143740	8m	TargetScan

miR-492	ENSG00000143847	8m	TargetScan
miR-492	ENSG00000144043	8m	TargetScan
miR-492	ENSG00000144152	8m	TargetScan
miR-492	ENSG00000144228	8m	TargetScan
miR-492	ENSG00000144355	8m	TargetScan
miR-492	ENSG00000144357	8m	TargetScan
miR-492	ENSG00000144847	8m	TargetScan
miR-492	ENSG00000145779	8m	TargetScan
miR-492	ENSG00000145832	8m	TargetScan
miR-492	ENSG00000146216	8m	TargetScan
miR-492	ENSG00000146263	8m	TargetScan
miR-492	ENSG00000146267	8m	TargetScan
miR-492	ENSG00000146676	8m	TargetScan
miR-492	ENSG00000147010	8m	TargetScan
miR-492	ENSG00000147145	8m	TargetScan
miR-492	ENSG00000147234	8m	TargetScan
miR-492	ENSG00000147614	8m	TargetScan
miR-492	ENSG00000148053	8m	TargetScan
miR-492	ENSG00000148120	8m	TargetScan
miR-492	ENSG00000148153	8m	TargetScan
miR-492	ENSG00000148296	8m	TargetScan
miR-492	ENSG00000148384	8m	TargetScan
miR-492	ENSG00000148704	8m	TargetScan
miR-492	ENSG00000148730	8m	TargetScan
miR-492	ENSG00000148848	8m	TargetScan
miR-492	ENSG00000149212	8m	TargetScan
miR-492	ENSG00000149451	8m	TargetScan
miR-492	ENSG00000149488	8m	TargetScan
miR-492	ENSG00000149575	8m	TargetScan
miR-492	ENSG00000151136	8m	TargetScan
miR-492	ENSG00000151164	8m	TargetScan

miR-492	ENSG00000151532	8m	TargetScan
miR-492	ENSG00000151948	8m	TargetScan
miR-492	ENSG00000153037	8m	TargetScan
miR-492	ENSG00000153404	8m	TargetScan
miR-492	ENSG00000153879	8m	TargetScan
miR-492	ENSG00000153904	8m	TargetScan
miR-492	ENSG00000153936	8m	TargetScan
miR-492	ENSG00000154025	8m	TargetScan
miR-492	ENSG00000154174	8m	TargetScan
miR-492	ENSG00000154473	8m	TargetScan
miR-492	ENSG00000154518	8m	TargetScan
miR-492	ENSG00000154734	8m	TargetScan
miR-492	ENSG00000154930	8m	TargetScan
miR-492	ENSG00000155254	8m	TargetScan
miR-492	ENSG00000155561	8m	TargetScan
miR-492	ENSG00000155657	8m	TargetScan
miR-492	ENSG00000155926	8m	TargetScan
miR-492	ENSG00000155966	8m	TargetScan
miR-492	ENSG00000156172	8m	TargetScan
miR-492	ENSG00000156232	8m	TargetScan
miR-492	ENSG00000156239	8m	TargetScan
miR-492	ENSG00000157388	8m	TargetScan
miR-492	ENSG00000157540	8m	TargetScan
miR-492	ENSG00000157617	8m	TargetScan
miR-492	ENSG00000157657	8m	TargetScan
miR-492	ENSG00000157680	8m	TargetScan
miR-492	ENSG00000157741	8m	TargetScan
miR-492	ENSG00000158163	8m	TargetScan
miR-492	ENSG00000158423	8m	TargetScan
miR-492	ENSG00000158445	8m	TargetScan
miR-492	ENSG00000158813	8m	TargetScan

miR-492	ENSG00000158863	8m	TargetScan
miR-492	ENSG00000159111	8m	TargetScan
miR-492	ENSG00000159348	8m	TargetScan
miR-492	ENSG00000160194	8m	TargetScan
miR-492	ENSG00000160199	8m	TargetScan
miR-492	ENSG00000160216	8m	TargetScan
miR-492	ENSG00000160233	8m	TargetScan
miR-492	ENSG00000160716	8m	TargetScan
miR-492	ENSG00000161203	8m	TargetScan
miR-492	ENSG00000162066	8m	TargetScan
miR-492	ENSG00000162341	8m	TargetScan
miR-492	ENSG00000162461	8m	TargetScan
miR-492	ENSG00000162545	8m	TargetScan
miR-492	ENSG00000162676	8m	TargetScan
miR-492	ENSG00000162783	8m	TargetScan
miR-492	ENSG00000162971	8m	TargetScan
miR-492	ENSG00000163393	8m	TargetScan
miR-492	ENSG00000163820	8m	TargetScan
miR-492	ENSG00000164007	8m	TargetScan
miR-492	ENSG00000164080	8m	TargetScan
miR-492	ENSG00000164366	8m	TargetScan
miR-492	ENSG00000164626	8m	TargetScan
miR-492	ENSG00000164627	8m	TargetScan
miR-492	ENSG00000164674	8m	TargetScan
miR-492	ENSG00000164715	8m	TargetScan
miR-492	ENSG00000164978	8m	TargetScan
miR-492	ENSG00000165194	8m	TargetScan
miR-492	ENSG00000165609	8m	TargetScan
miR-492	ENSG00000165650	8m	TargetScan
miR-492	ENSG00000165861	8m	TargetScan
miR-492	ENSG00000166170	8m	TargetScan

miR-492	ENSG00000166257	8m	TargetScan
miR-492	ENSG00000166341	8m	TargetScan
miR-492	ENSG00000166387	8m	TargetScan
miR-492	ENSG00000166436	8m	TargetScan
miR-492	ENSG00000166507	8m	TargetScan
miR-492	ENSG00000166750	8m	TargetScan
miR-492	ENSG00000166833	8m	TargetScan
miR-492	ENSG00000166923	8m	TargetScan
miR-492	ENSG00000166984	8m	TargetScan
miR-492	ENSG00000167131	8m	TargetScan
miR-492	ENSG00000167186	8m	TargetScan
miR-492	ENSG00000167208	8m	TargetScan
miR-492	ENSG00000167291	8m	TargetScan
miR-492	ENSG00000167461	8m	TargetScan
miR-492	ENSG00000167613	8m	TargetScan
miR-492	ENSG00000167656	8m	TargetScan
miR-492	ENSG00000167703	8m	TargetScan
miR-492	ENSG00000167910	8m	TargetScan
miR-492	ENSG00000168062	8m	TargetScan
miR-492	ENSG00000168159	8m	TargetScan
miR-492	ENSG00000168569	8m	TargetScan
miR-492	ENSG00000168724	8m	TargetScan
miR-492	ENSG00000168903	8m	TargetScan
miR-492	ENSG00000168904	8m	TargetScan
miR-492	ENSG00000168906	8m	TargetScan
miR-492	ENSG00000168939	8m	TargetScan
miR-492	ENSG00000168939	8m	TargetScan
miR-492	ENSG00000169021	8m	TargetScan
miR-492	ENSG00000169083	8m	TargetScan
miR-492	ENSG00000169118	8m	TargetScan
miR-492	ENSG00000169379	8m	TargetScan

miR-492	ENSG00000169604	8m	TargetScan
miR-492	ENSG00000169733	8m	TargetScan
miR-492	ENSG00000169855	8m	TargetScan
miR-492	ENSG00000169908	8m	TargetScan
miR-492	ENSG00000169933	8m	TargetScan
miR-492	ENSG00000170160	8m	TargetScan
miR-492	ENSG00000170525	8m	TargetScan
miR-492	ENSG00000170571	8m	TargetScan
miR-492	ENSG00000170962	8m	TargetScan
miR-492	ENSG00000171124	8m	TargetScan
miR-492	ENSG00000171246	8m	TargetScan
miR-492	ENSG00000171792	8m	TargetScan
miR-492	ENSG00000171914	8m	TargetScan
miR-492	ENSG00000172057	8m	TargetScan
miR-492	ENSG00000172113	8m	TargetScan
miR-492	ENSG00000172292	8m	TargetScan
miR-492	ENSG00000172538	8m	TargetScan
miR-492	ENSG00000172819	8m	TargetScan
miR-492	ENSG00000172893	8m	TargetScan
miR-492	ENSG00000172987	8m	TargetScan
miR-492	ENSG00000173218	8m	TargetScan
miR-492	ENSG00000173432	8m	TargetScan
miR-492	ENSG00000173535	8m	TargetScan
miR-492	ENSG00000173548	8m	TargetScan
miR-492	ENSG00000173578	8m	TargetScan
miR-492	ENSG00000173744	8m	TargetScan
miR-492	ENSG00000173801	8m	TargetScan
miR-492	ENSG00000173821	8m	TargetScan
miR-492	ENSG00000173838	8m	TargetScan
miR-492	ENSG00000173894	8m	TargetScan
miR-492	ENSG00000173933	8m	TargetScan

miR-492	ENSG00000173950	8m	TargetScan
miR-492	ENSG00000173992	8m	TargetScan
miR-492	ENSG00000174145	8m	TargetScan
miR-492	ENSG00000174227	8m	TargetScan
miR-492	ENSG00000174358	8m	TargetScan
miR-492	ENSG00000174373	8m	TargetScan
miR-492	ENSG00000174574	8m	TargetScan
miR-492	ENSG00000174600	8m	TargetScan
miR-492	ENSG00000175221	8m	TargetScan
miR-492	ENSG00000175728	8m	TargetScan
miR-492	ENSG00000176171	8m	TargetScan
miR-492	ENSG00000176383	8m	TargetScan
miR-492	ENSG00000176834	8m	TargetScan
miR-492	ENSG00000176896	8m	TargetScan
miR-492	ENSG00000176920	8m	TargetScan
miR-492	ENSG00000176945	8m	TargetScan
miR-492	ENSG00000176974	8m	TargetScan
miR-492	ENSG00000177058	8m	TargetScan
miR-492	ENSG00000177352	8m	TargetScan
miR-492	ENSG00000177463	8m	TargetScan
miR-492	ENSG00000177663	8m	TargetScan
miR-492	ENSG00000177674	8m	TargetScan
miR-492	ENSG00000177732	8m	TargetScan
miR-492	ENSG00000178057	8m	TargetScan
miR-492	ENSG00000178171	8m	TargetScan
miR-492	ENSG00000178233	8m	TargetScan
miR-492	ENSG00000178287	8m	TargetScan
miR-492	ENSG00000178860	8m	TargetScan
miR-492	ENSG00000179348	8m	TargetScan
miR-492	ENSG00000179387	8m	TargetScan
miR-492	ENSG00000179583	8m	TargetScan

miR-492	ENSG00000179813	8m	TargetScan
miR-492	ENSG00000179886	8m	TargetScan
miR-492	ENSG00000180438	8m	TargetScan
miR-492	ENSG00000180535	8m	TargetScan
miR-492	ENSG00000180869	8m	TargetScan
miR-492	ENSG00000180901	8m	TargetScan
miR-492	ENSG00000181027	8m	TargetScan
miR-492	ENSG00000181090	8m	TargetScan
miR-492	ENSG00000181610	8m	TargetScan
miR-492	ENSG00000181982	8m	TargetScan
miR-492	ENSG00000182095	8m	TargetScan
miR-492	ENSG00000182156	8m	TargetScan
miR-492	ENSG00000182185	8m	TargetScan
miR-492	ENSG00000182704	8m	TargetScan
miR-492	ENSG00000182742	8m	TargetScan
miR-492	ENSG00000182858	8m	TargetScan
miR-492	ENSG00000182957	8m	TargetScan
miR-492	ENSG00000182968	8m	TargetScan
miR-492	ENSG00000183287	8m	TargetScan
miR-492	ENSG00000183527	8m	TargetScan
miR-492	ENSG00000183723	8m	TargetScan
miR-492	ENSG00000183751	8m	TargetScan
miR-492	ENSG00000184076	8m	TargetScan
miR-492	ENSG00000184144	8m	TargetScan
miR-492	ENSG00000184185	8m	TargetScan
miR-492	ENSG00000184560	8m	TargetScan
miR-492	ENSG00000184937	8m	TargetScan
miR-492	ENSG00000185055	8m	TargetScan
miR-492	ENSG00000185088	8m	TargetScan
miR-492	ENSG00000185112	8m	TargetScan
miR-492	ENSG00000185262	8m	TargetScan

miR-492	ENSG00000185721	8m	TargetScan
miR-492	ENSG00000185722	8m	TargetScan
miR-492	ENSG00000185736	8m	TargetScan
miR-492	ENSG00000185739	8m	TargetScan
miR-492	ENSG00000185745	8m	TargetScan
miR-492	ENSG00000185860	8m	TargetScan
miR-492	ENSG00000185924	8m	TargetScan
miR-492	ENSG00000185933	8m	TargetScan
miR-492	ENSG00000186051	8m	TargetScan
miR-492	ENSG00000186174	8m	TargetScan
miR-492	ENSG00000186260	8m	TargetScan
miR-492	ENSG00000186431	8m	TargetScan
miR-492	ENSG00000186812	8m	TargetScan
miR-492	ENSG00000187123	8m	TargetScan
miR-492	ENSG00000187187	8m	TargetScan
miR-492	ENSG00000187239	8m	TargetScan
miR-492	ENSG00000187391	8m	TargetScan
miR-492	ENSG00000187726	8m	TargetScan
miR-492	ENSG00000187840	8m	TargetScan
miR-492	ENSG00000187942	8m	TargetScan
miR-492	ENSG00000187980	8m	TargetScan
miR-492	ENSG00000188167	8m	TargetScan
miR-492	ENSG00000188373	8m	TargetScan
miR-492	ENSG00000188488	8m	TargetScan
miR-492	ENSG00000188522	8m	TargetScan
miR-492	ENSG00000188529	8m	TargetScan
miR-492	ENSG00000188735	8m	TargetScan
miR-492	ENSG00000188827	8m	TargetScan
miR-492	ENSG00000189007	8m	TargetScan
miR-492	ENSG00000189023	8m	TargetScan
miR-492	ENSG00000189091	8m	TargetScan

miR-492	ENSG00000189319	8m	TargetScan
miR-492	ENSG00000189320	8m	TargetScan
miR-492	ENSG00000196155	8m	TargetScan
miR-492	ENSG00000196167	8m	TargetScan
miR-492	ENSG00000196187	8m	TargetScan
miR-492	ENSG00000196209	8m	TargetScan
miR-492	ENSG00000196220	8m	TargetScan
miR-492	ENSG00000196242	8m	TargetScan
miR-492	ENSG00000196591	8m	TargetScan
miR-492	ENSG00000196776	8m	TargetScan
miR-492	ENSG00000196981	8m	TargetScan
miR-492	ENSG00000197084	8m	TargetScan
miR-492	ENSG00000197183	8m	TargetScan
miR-492	ENSG00000197275	8m	TargetScan
miR-492	ENSG00000197702	8m	TargetScan
miR-492	ENSG00000197782	8m	TargetScan
miR-492	ENSG00000197912	8m	TargetScan
miR-492	ENSG00000198040	8m	TargetScan
miR-492	ENSG00000198113	8m	TargetScan
miR-492	ENSG00000198133	8m	TargetScan
miR-492	ENSG00000198162	8m	TargetScan
miR-492	ENSG00000198326	8m	TargetScan
miR-492	ENSG00000198420	8m	TargetScan
miR-492	ENSG00000198570	8m	TargetScan
miR-492	ENSG00000198586	8m	TargetScan
miR-492	ENSG00000198755	8m	TargetScan
miR-492	ENSG00000198792	8m	TargetScan
miR-492	ENSG00000198948	8m	TargetScan
miR-492	ENSG00000203485	8m	TargetScan
miR-492	ENSG00000203690	8m	TargetScan
miR-492	ENSG00000204128	8m	TargetScan

miR-492	ENSG00000204271	8m	TargetScan
miR-492	ENSG00000204580	8m	TargetScan
miR-492	ENSG00000204628	8m	TargetScan
miR-492	ENSG00000205268	8m	TargetScan
miR-492	ENSG00000205795	8m	TargetScan
miR-492	ENSG00000205922	8m	TargetScan
miR-492	ENSG00000206199	8m	TargetScan
miR-492	ENSG00000213654	8m	TargetScan
miR-492	ENSG00000213699	8m	TargetScan
miR-492	ENSG00000213741	8m	TargetScan
miR-492	ENSG00000214022	8m	TargetScan
miR-492	ENSG00000214248	8m	TargetScan
miR-492	ENSG00000214517	8m	TargetScan
miR-492	ENSG00000214518	8m	TargetScan
miR-492	ENSG00000214736	8m	TargetScan
miR-492	ENSG00000215612	8m	TargetScan
miR-492	ENSG00000217702	8m	TargetScan
miR-492	ENSG00000218357	8m	TargetScan
miR-492	ENSG00000235194	8m	TargetScan
miR-492	ENSG00000237440	8m	TargetScan
miR-492	ENSG00000237896	8m	TargetScan
miR-492	ENSG00000241127	8m	TargetScan
miR-492	ENSG00000241839	8m	TargetScan
miR-492	ENSG00000244509	8m	TargetScan
miR-492	ENSG00000245680	8m	TargetScan
miR-492	ENSG00000248050	8m	TargetScan
miR-492	ENSG00000248919	8m	TargetScan
miR-492	ENSG00000249242	8m	TargetScan
miR-492	ENSG00000255346	8m	TargetScan
miR-492	ENSG00000258869	8m	TargetScan
miR-492	ENSG00000258986	8m	TargetScan

miR-492	ENSG00000260007	8m	TargetScan
miR-492	ENSG00000265817	8m	TargetScan
miR-492	ENSG00000267270	8m	TargetScan
miR-492	ENSG00000267432	8m	TargetScan
miR-492	ENSG00000267561	8m	TargetScan
miR-492	ENSG00000272325	8m	TargetScan
miR-492	ENSG00000272414	8m	TargetScan
miR-492	ENSG00000277858	8m	TargetScan
miR-492	ENSG00000283597	8m	TargetScan
miR-492	ENSG00000288436	8m	TargetScan
miR-7161-5p	ENSG00000003393	8m	TargetScan
miR-7161-5p	ENSG00000004897	8m	TargetScan
miR-7161-5p	ENSG00000005100	8m	TargetScan
miR-7161-5p	ENSG00000005175	8m	TargetScan
miR-7161-5p	ENSG00000005812	8m	TargetScan
miR-7161-5p	ENSG00000005893	8m	TargetScan
miR-7161-5p	ENSG00000006459	8m	TargetScan
miR-7161-5p	ENSG00000006468	8m	TargetScan
miR-7161-5p	ENSG00000006576	8m	TargetScan
miR-7161-5p	ENSG00000006634	8m	TargetScan
miR-7161-5p	ENSG00000010244	8m	TargetScan
miR-7161-5p	ENSG00000010404	8m	TargetScan
miR-7161-5p	ENSG00000011007	8m	TargetScan
miR-7161-5p	ENSG00000011258	8m	TargetScan
miR-7161-5p	ENSG00000013375	8m	TargetScan
miR-7161-5p	ENSG00000014257	8m	TargetScan
miR-7161-5p	ENSG00000015153	8m	TargetScan
miR-7161-5p	ENSG00000015475	8m	TargetScan
miR-7161-5p	ENSG00000015520	8m	TargetScan
miR-7161-5p	ENSG00000017427	8m	TargetScan
miR-7161-5p	ENSG00000018236	8m	TargetScan

miR-7161-5p	ENSG00000018510	8m	TargetScan
miR-7161-5p	ENSG00000020922	8m	TargetScan
miR-7161-5p	ENSG00000023318	8m	TargetScan
miR-7161-5p	ENSG00000024862	8m	TargetScan
miR-7161-5p	ENSG00000026652	8m	TargetScan
miR-7161-5p	ENSG00000028528	8m	TargetScan
miR-7161-5p	ENSG00000028839	8m	TargetScan
miR-7161-5p	ENSG00000038210	8m	TargetScan
miR-7161-5p	ENSG00000039123	8m	TargetScan
miR-7161-5p	ENSG00000040341	8m	TargetScan
miR-7161-5p	ENSG00000040933	8m	TargetScan
miR-7161-5p	ENSG00000042781	8m	TargetScan
miR-7161-5p	ENSG00000046653	8m	TargetScan
miR-7161-5p	ENSG00000047346	8m	TargetScan
miR-7161-5p	ENSG00000048052	8m	TargetScan
miR-7161-5p	ENSG00000048828	8m	TargetScan
miR-7161-5p	ENSG00000050393	8m	TargetScan
miR-7161-5p	ENSG00000050405	8m	TargetScan
miR-7161-5p	ENSG00000053770	8m	TargetScan
miR-7161-5p	ENSG00000054965	8m	TargetScan
miR-7161-5p	ENSG00000057294	8m	TargetScan
miR-7161-5p	ENSG00000058091	8m	TargetScan
miR-7161-5p	ENSG00000060140	8m	TargetScan
miR-7161-5p	ENSG00000061455	8m	TargetScan
miR-7161-5p	ENSG00000061676	8m	TargetScan
miR-7161-5p	ENSG00000061987	8m	TargetScan
miR-7161-5p	ENSG00000064115	8m	TargetScan
miR-7161-5p	ENSG00000064393	8m	TargetScan
miR-7161-5p	ENSG00000064545	8m	TargetScan
miR-7161-5p	ENSG00000065413	8m	TargetScan
miR-7161-5p	ENSG00000065548	8m	TargetScan

miR-7161-5p	ENSG00000066084	8m	TargetScan
miR-7161-5p	ENSG00000066294	8m	TargetScan
miR-7161-5p	ENSG00000066651	8m	TargetScan
miR-7161-5p	ENSG00000066697	8m	TargetScan
miR-7161-5p	ENSG00000066813	8m	TargetScan
miR-7161-5p	ENSG00000067066	8m	TargetScan
miR-7161-5p	ENSG00000068024	8m	TargetScan
miR-7161-5p	ENSG00000068745	8m	TargetScan
miR-7161-5p	ENSG00000069020	8m	TargetScan
miR-7161-5p	ENSG00000069275	8m	TargetScan
miR-7161-5p	ENSG00000069702	8m	TargetScan
miR-7161-5p	ENSG00000070182	8m	TargetScan
miR-7161-5p	ENSG00000070193	8m	TargetScan
miR-7161-5p	ENSG00000070214	8m	TargetScan
miR-7161-5p	ENSG00000070756	8m	TargetScan
miR-7161-5p	ENSG00000071073	8m	TargetScan
miR-7161-5p	ENSG00000071203	8m	TargetScan
miR-7161-5p	ENSG00000071243	8m	TargetScan
miR-7161-5p	ENSG00000072042	8m	TargetScan
miR-7161-5p	ENSG00000072121	8m	TargetScan
miR-7161-5p	ENSG00000074590	8m	TargetScan
miR-7161-5p	ENSG00000075303	8m	TargetScan
miR-7161-5p	ENSG00000075884	8m	TargetScan
miR-7161-5p	ENSG00000077157	8m	TargetScan
miR-7161-5p	ENSG00000077232	8m	TargetScan
miR-7161-5p	ENSG00000077458	8m	TargetScan
miR-7161-5p	ENSG00000077616	8m	TargetScan
miR-7161-5p	ENSG00000078549	8m	TargetScan
miR-7161-5p	ENSG00000080493	8m	TargetScan
miR-7161-5p	ENSG00000081026	8m	TargetScan
miR-7161-5p	ENSG00000081059	8m	TargetScan

miR-7161-5p	ENSG00000081148	8m	TargetScan
miR-7161-5p	ENSG00000081377	8m	TargetScan
miR-7161-5p	ENSG00000082258	8m	TargetScan
miR-7161-5p	ENSG00000083312	8m	TargetScan
miR-7161-5p	ENSG00000083635	8m	TargetScan
miR-7161-5p	ENSG00000083844	8m	TargetScan
miR-7161-5p	ENSG00000084072	8m	TargetScan
miR-7161-5p	ENSG00000084092	8m	TargetScan
miR-7161-5p	ENSG00000084674	8m	TargetScan
miR-7161-5p	ENSG00000085274	8m	TargetScan
miR-7161-5p	ENSG00000085871	8m	TargetScan
miR-7161-5p	ENSG00000086300	8m	TargetScan
miR-7161-5p	ENSG00000086475	8m	TargetScan
miR-7161-5p	ENSG00000086991	8m	TargetScan
miR-7161-5p	ENSG00000087263	8m	TargetScan
miR-7161-5p	ENSG00000087502	8m	TargetScan
miR-7161-5p	ENSG00000088356	8m	TargetScan
miR-7161-5p	ENSG00000088387	8m	TargetScan
miR-7161-5p	ENSG00000088812	8m	TargetScan
miR-7161-5p	ENSG00000089916	8m	TargetScan
miR-7161-5p	ENSG00000090615	8m	TargetScan
miR-7161-5p	ENSG00000091140	8m	TargetScan
miR-7161-5p	ENSG00000091986	8m	TargetScan
miR-7161-5p	ENSG00000092871	8m	TargetScan
miR-7161-5p	ENSG00000092978	8m	TargetScan
miR-7161-5p	ENSG00000093000	8m	TargetScan
miR-7161-5p	ENSG00000095203	8m	TargetScan
miR-7161-5p	ENSG00000095637	8m	TargetScan
miR-7161-5p	ENSG00000096060	8m	TargetScan
miR-7161-5p	ENSG00000096063	8m	TargetScan
miR-7161-5p	ENSG00000096401	8m	TargetScan

miR-7161-5p	ENSG00000100296	8m	TargetScan
miR-7161-5p	ENSG00000100335	8m	TargetScan
miR-7161-5p	ENSG00000100354	8m	TargetScan
miR-7161-5p	ENSG00000100376	8m	TargetScan
miR-7161-5p	ENSG00000100433	8m	TargetScan
miR-7161-5p	ENSG00000100505	8m	TargetScan
miR-7161-5p	ENSG00000100592	8m	TargetScan
miR-7161-5p	ENSG00000100600	8m	TargetScan
miR-7161-5p	ENSG00000100614	8m	TargetScan
miR-7161-5p	ENSG00000100625	8m	TargetScan
miR-7161-5p	ENSG00000100664	8m	TargetScan
miR-7161-5p	ENSG00000100697	8m	TargetScan
miR-7161-5p	ENSG00000100815	8m	TargetScan
miR-7161-5p	ENSG00000100934	8m	TargetScan
miR-7161-5p	ENSG00000101190	8m	TargetScan
miR-7161-5p	ENSG00000101191	8m	TargetScan
miR-7161-5p	ENSG00000101336	8m	TargetScan
miR-7161-5p	ENSG00000101670	8m	TargetScan
miR-7161-5p	ENSG00000101751	8m	TargetScan
miR-7161-5p	ENSG00000101938	8m	TargetScan
miR-7161-5p	ENSG00000102158	8m	TargetScan
miR-7161-5p	ENSG00000102189	8m	TargetScan
miR-7161-5p	ENSG00000102226	8m	TargetScan
miR-7161-5p	ENSG00000102383	8m	TargetScan
miR-7161-5p	ENSG00000102547	8m	TargetScan
miR-7161-5p	ENSG00000102781	8m	TargetScan
miR-7161-5p	ENSG00000102970	8m	TargetScan
miR-7161-5p	ENSG00000103056	8m	TargetScan
miR-7161-5p	ENSG00000103067	8m	TargetScan
miR-7161-5p	ENSG00000103184	8m	TargetScan
miR-7161-5p	ENSG00000103978	8m	TargetScan

miR-7161-5p	ENSG00000104290	8m	TargetScan
miR-7161-5p	ENSG00000104331	8m	TargetScan
miR-7161-5p	ENSG00000104731	8m	TargetScan
miR-7161-5p	ENSG00000105220	8m	TargetScan
miR-7161-5p	ENSG00000105483	8m	TargetScan
miR-7161-5p	ENSG00000105492	8m	TargetScan
miR-7161-5p	ENSG00000105497	8m	TargetScan
miR-7161-5p	ENSG00000105568	8m	TargetScan
miR-7161-5p	ENSG00000105750	8m	TargetScan
miR-7161-5p	ENSG00000105821	8m	TargetScan
miR-7161-5p	ENSG00000105866	8m	TargetScan
miR-7161-5p	ENSG00000105976	8m	TargetScan
miR-7161-5p	ENSG00000105983	8m	TargetScan
miR-7161-5p	ENSG00000106113	8m	TargetScan
miR-7161-5p	ENSG00000106460	8m	TargetScan
miR-7161-5p	ENSG00000106665	8m	TargetScan
miR-7161-5p	ENSG00000106723	8m	TargetScan
miR-7161-5p	ENSG00000106772	8m	TargetScan
miR-7161-5p	ENSG00000106799	8m	TargetScan
miR-7161-5p	ENSG00000107105	8m	TargetScan
miR-7161-5p	ENSG00000107443	8m	TargetScan
miR-7161-5p	ENSG00000107562	8m	TargetScan
miR-7161-5p	ENSG00000107581	8m	TargetScan
miR-7161-5p	ENSG00000107651	8m	TargetScan
miR-7161-5p	ENSG00000107960	8m	TargetScan
miR-7161-5p	ENSG00000108061	8m	TargetScan
miR-7161-5p	ENSG00000108924	8m	TargetScan
miR-7161-5p	ENSG00000109158	8m	TargetScan
miR-7161-5p	ENSG00000109220	8m	TargetScan
miR-7161-5p	ENSG00000109265	8m	TargetScan
miR-7161-5p	ENSG00000109458	8m	TargetScan

miR-7161-5p	ENSG00000109466	8m	TargetScan
miR-7161-5p	ENSG00000109854	8m	TargetScan
miR-7161-5p	ENSG00000109861	8m	TargetScan
miR-7161-5p	ENSG00000109906	8m	TargetScan
miR-7161-5p	ENSG00000110172	8m	TargetScan
miR-7161-5p	ENSG00000110427	8m	TargetScan
miR-7161-5p	ENSG00000110429	8m	TargetScan
miR-7161-5p	ENSG00000110436	8m	TargetScan
miR-7161-5p	ENSG00000110665	8m	TargetScan
miR-7161-5p	ENSG00000110693	8m	TargetScan
miR-7161-5p	ENSG00000110848	8m	TargetScan
miR-7161-5p	ENSG00000110876	8m	TargetScan
miR-7161-5p	ENSG00000111110	8m	TargetScan
miR-7161-5p	ENSG00000111371	8m	TargetScan
miR-7161-5p	ENSG00000111700	8m	TargetScan
miR-7161-5p	ENSG00000111816	8m	TargetScan
miR-7161-5p	ENSG00000111817	8m	TargetScan
miR-7161-5p	ENSG00000111837	8m	TargetScan
miR-7161-5p	ENSG00000111879	8m	TargetScan
miR-7161-5p	ENSG00000112038	8m	TargetScan
miR-7161-5p	ENSG00000112062	8m	TargetScan
miR-7161-5p	ENSG00000112081	8m	TargetScan
miR-7161-5p	ENSG00000112096	8m	TargetScan
miR-7161-5p	ENSG00000112200	8m	TargetScan
miR-7161-5p	ENSG00000112214	8m	TargetScan
miR-7161-5p	ENSG00000112218	8m	TargetScan
miR-7161-5p	ENSG00000112339	8m	TargetScan
miR-7161-5p	ENSG00000112394	8m	TargetScan
miR-7161-5p	ENSG00000112531	8m	TargetScan
miR-7161-5p	ENSG00000112695	8m	TargetScan
miR-7161-5p	ENSG00000112851	8m	TargetScan

miR-7161-5p	ENSG00000112852	8m	TargetScan
miR-7161-5p	ENSG00000113300	8m	TargetScan
miR-7161-5p	ENSG00000113319	8m	TargetScan
miR-7161-5p	ENSG00000113441	8m	TargetScan
miR-7161-5p	ENSG00000113448	8m	TargetScan
miR-7161-5p	ENSG00000113594	8m	TargetScan
miR-7161-5p	ENSG00000113719	8m	TargetScan
miR-7161-5p	ENSG00000114416	8m	TargetScan
miR-7161-5p	ENSG00000114503	8m	TargetScan
miR-7161-5p	ENSG00000114686	8m	TargetScan
miR-7161-5p	ENSG00000114861	8m	TargetScan
miR-7161-5p	ENSG00000115020	8m	TargetScan
miR-7161-5p	ENSG00000115365	8m	TargetScan
miR-7161-5p	ENSG00000115461	8m	TargetScan
miR-7161-5p	ENSG00000115474	8m	TargetScan
miR-7161-5p	ENSG00000115561	8m	TargetScan
miR-7161-5p	ENSG00000115977	8m	TargetScan
miR-7161-5p	ENSG00000116001	8m	TargetScan
miR-7161-5p	ENSG00000116005	8m	TargetScan
miR-7161-5p	ENSG00000116062	8m	TargetScan
miR-7161-5p	ENSG00000116191	8m	TargetScan
miR-7161-5p	ENSG00000116198	8m	TargetScan
miR-7161-5p	ENSG00000116209	8m	TargetScan
miR-7161-5p	ENSG00000116679	8m	TargetScan
miR-7161-5p	ENSG00000116954	8m	TargetScan
miR-7161-5p	ENSG00000116984	8m	TargetScan
miR-7161-5p	ENSG00000117020	8m	TargetScan
miR-7161-5p	ENSG00000117114	8m	TargetScan
miR-7161-5p	ENSG00000117152	8m	TargetScan
miR-7161-5p	ENSG00000117450	8m	TargetScan
miR-7161-5p	ENSG00000117505	8m	TargetScan

miR-7161-5p	ENSG00000117569	8m	TargetScan
miR-7161-5p	ENSG00000117602	8m	TargetScan
miR-7161-5p	ENSG00000117616	8m	TargetScan
miR-7161-5p	ENSG00000118200	8m	TargetScan
miR-7161-5p	ENSG00000118217	8m	TargetScan
miR-7161-5p	ENSG00000118242	8m	TargetScan
miR-7161-5p	ENSG00000118260	8m	TargetScan
miR-7161-5p	ENSG00000118432	8m	TargetScan
miR-7161-5p	ENSG00000118508	8m	TargetScan
miR-7161-5p	ENSG00000118579	8m	TargetScan
miR-7161-5p	ENSG00000118733	8m	TargetScan
miR-7161-5p	ENSG00000118939	8m	TargetScan
miR-7161-5p	ENSG00000118960	8m	TargetScan
miR-7161-5p	ENSG00000119004	8m	TargetScan
miR-7161-5p	ENSG00000119280	8m	TargetScan
miR-7161-5p	ENSG00000119283	8m	TargetScan
miR-7161-5p	ENSG00000119321	8m	TargetScan
miR-7161-5p	ENSG00000119508	8m	TargetScan
miR-7161-5p	ENSG00000119638	8m	TargetScan
miR-7161-5p	ENSG00000119673	8m	TargetScan
miR-7161-5p	ENSG00000119760	8m	TargetScan
miR-7161-5p	ENSG00000119900	8m	TargetScan
miR-7161-5p	ENSG00000119953	8m	TargetScan
miR-7161-5p	ENSG00000119969	8m	TargetScan
miR-7161-5p	ENSG00000120694	8m	TargetScan
miR-7161-5p	ENSG00000120742	8m	TargetScan
miR-7161-5p	ENSG00000120798	8m	TargetScan
miR-7161-5p	ENSG00000121274	8m	TargetScan
miR-7161-5p	ENSG00000121454	8m	TargetScan
miR-7161-5p	ENSG00000121671	8m	TargetScan
miR-7161-5p	ENSG00000121864	8m	TargetScan

miR-7161-5p	ENSG00000121879	8m	TargetScan
miR-7161-5p	ENSG00000122406	8m	TargetScan
miR-7161-5p	ENSG00000122733	8m	TargetScan
miR-7161-5p	ENSG00000122741	8m	TargetScan
miR-7161-5p	ENSG00000123080	8m	TargetScan
miR-7161-5p	ENSG00000123104	8m	TargetScan
miR-7161-5p	ENSG00000123119	8m	TargetScan
miR-7161-5p	ENSG00000123200	8m	TargetScan
miR-7161-5p	ENSG00000123352	8m	TargetScan
miR-7161-5p	ENSG00000123836	8m	TargetScan
miR-7161-5p	ENSG00000124172	8m	TargetScan
miR-7161-5p	ENSG00000124193	8m	TargetScan
miR-7161-5p	ENSG00000124224	8m	TargetScan
miR-7161-5p	ENSG00000124356	8m	TargetScan
miR-7161-5p	ENSG00000124374	8m	TargetScan
miR-7161-5p	ENSG00000124406	8m	TargetScan
miR-7161-5p	ENSG00000124571	8m	TargetScan
miR-7161-5p	ENSG00000124731	8m	TargetScan
miR-7161-5p	ENSG00000124767	8m	TargetScan
miR-7161-5p	ENSG00000124783	8m	TargetScan
miR-7161-5p	ENSG00000125249	8m	TargetScan
miR-7161-5p	ENSG00000125637	8m	TargetScan
miR-7161-5p	ENSG00000125863	8m	TargetScan
miR-7161-5p	ENSG00000125885	8m	TargetScan
miR-7161-5p	ENSG00000125970	8m	TargetScan
miR-7161-5p	ENSG00000126012	8m	TargetScan
miR-7161-5p	ENSG00000126070	8m	TargetScan
miR-7161-5p	ENSG00000126217	8m	TargetScan
miR-7161-5p	ENSG00000126953	8m	TargetScan
miR-7161-5p	ENSG00000127022	8m	TargetScan
miR-7161-5p	ENSG00000127083	8m	TargetScan

miR-7161-5p	ENSG00000127980	8m	TargetScan
miR-7161-5p	ENSG00000128000	8m	TargetScan
miR-7161-5p	ENSG00000128534	8m	TargetScan
miR-7161-5p	ENSG00000128923	8m	TargetScan
miR-7161-5p	ENSG00000129250	8m	TargetScan
miR-7161-5p	ENSG00000129315	8m	TargetScan
miR-7161-5p	ENSG00000129422	8m	TargetScan
miR-7161-5p	ENSG00000129657	8m	TargetScan
miR-7161-5p	ENSG00000130021	8m	TargetScan
miR-7161-5p	ENSG00000130035	8m	TargetScan
miR-7161-5p	ENSG00000130147	8m	TargetScan
miR-7161-5p	ENSG00000130348	8m	TargetScan
miR-7161-5p	ENSG00000130363	8m	TargetScan
miR-7161-5p	ENSG00000131013	8m	TargetScan
miR-7161-5p	ENSG00000131127	8m	TargetScan
miR-7161-5p	ENSG00000131368	8m	TargetScan
miR-7161-5p	ENSG00000131370	8m	TargetScan
miR-7161-5p	ENSG00000131503	8m	TargetScan
miR-7161-5p	ENSG00000131507	8m	TargetScan
miR-7161-5p	ENSG00000131724	8m	TargetScan
miR-7161-5p	ENSG00000131845	8m	TargetScan
miR-7161-5p	ENSG00000131979	8m	TargetScan
miR-7161-5p	ENSG00000132185	8m	TargetScan
miR-7161-5p	ENSG00000132256	8m	TargetScan
miR-7161-5p	ENSG00000132300	8m	TargetScan
miR-7161-5p	ENSG00000132334	8m	TargetScan
miR-7161-5p	ENSG00000132436	8m	TargetScan
miR-7161-5p	ENSG00000132603	8m	TargetScan
miR-7161-5p	ENSG00000132676	8m	TargetScan
miR-7161-5p	ENSG00000132953	8m	TargetScan
miR-7161-5p	ENSG00000133107	8m	TargetScan

miR-7161-5p	ENSG00000133124	8m	TargetScan
miR-7161-5p	ENSG00000133858	8m	TargetScan
miR-7161-5p	ENSG00000133863	8m	TargetScan
miR-7161-5p	ENSG00000133895	8m	TargetScan
miR-7161-5p	ENSG00000133997	8m	TargetScan
miR-7161-5p	ENSG00000134014	8m	TargetScan
miR-7161-5p	ENSG00000134152	8m	TargetScan
miR-7161-5p	ENSG00000134250	8m	TargetScan
miR-7161-5p	ENSG00000134253	8m	TargetScan
miR-7161-5p	ENSG00000134265	8m	TargetScan
miR-7161-5p	ENSG00000134333	8m	TargetScan
miR-7161-5p	ENSG00000134461	8m	TargetScan
miR-7161-5p	ENSG00000134533	8m	TargetScan
miR-7161-5p	ENSG00000134809	8m	TargetScan
miR-7161-5p	ENSG00000134884	8m	TargetScan
miR-7161-5p	ENSG00000134909	8m	TargetScan
miR-7161-5p	ENSG00000135040	8m	TargetScan
miR-7161-5p	ENSG00000135272	8m	TargetScan
miR-7161-5p	ENSG00000135297	8m	TargetScan
miR-7161-5p	ENSG00000135315	8m	TargetScan
miR-7161-5p	ENSG00000135452	8m	TargetScan
miR-7161-5p	ENSG00000135540	8m	TargetScan
miR-7161-5p	ENSG00000135547	8m	TargetScan
miR-7161-5p	ENSG00000135604	8m	TargetScan
miR-7161-5p	ENSG00000135953	8m	TargetScan
miR-7161-5p	ENSG00000136243	8m	TargetScan
miR-7161-5p	ENSG00000136383	8m	TargetScan
miR-7161-5p	ENSG00000136436	8m	TargetScan
miR-7161-5p	ENSG00000136450	8m	TargetScan
miR-7161-5p	ENSG00000136631	8m	TargetScan
miR-7161-5p	ENSG00000136688	8m	TargetScan

miR-7161-5p	ENSG00000136709	8m	TargetScan
miR-7161-5p	ENSG00000136807	8m	TargetScan
miR-7161-5p	ENSG00000136881	8m	TargetScan
miR-7161-5p	ENSG00000137040	8m	TargetScan
miR-7161-5p	ENSG00000137135	8m	TargetScan
miR-7161-5p	ENSG00000137393	8m	TargetScan
miR-7161-5p	ENSG00000137500	8m	TargetScan
miR-7161-5p	ENSG00000137502	8m	TargetScan
miR-7161-5p	ENSG00000137601	8m	TargetScan
miR-7161-5p	ENSG00000137692	8m	TargetScan
miR-7161-5p	ENSG00000137713	8m	TargetScan
miR-7161-5p	ENSG00000137770	8m	TargetScan
miR-7161-5p	ENSG00000137878	8m	TargetScan
miR-7161-5p	ENSG00000137959	8m	TargetScan
miR-7161-5p	ENSG00000137975	8m	TargetScan
miR-7161-5p	ENSG00000138032	8m	TargetScan
miR-7161-5p	ENSG00000138071	8m	TargetScan
miR-7161-5p	ENSG00000138190	8m	TargetScan
miR-7161-5p	ENSG00000138363	8m	TargetScan
miR-7161-5p	ENSG00000138442	8m	TargetScan
miR-7161-5p	ENSG00000138641	8m	TargetScan
miR-7161-5p	ENSG00000138650	8m	TargetScan
miR-7161-5p	ENSG00000138757	8m	TargetScan
miR-7161-5p	ENSG00000138778	8m	TargetScan
miR-7161-5p	ENSG00000138798	8m	TargetScan
miR-7161-5p	ENSG00000138821	8m	TargetScan
miR-7161-5p	ENSG00000139083	8m	TargetScan
miR-7161-5p	ENSG00000139146	8m	TargetScan
miR-7161-5p	ENSG00000139163	8m	TargetScan
miR-7161-5p	ENSG00000139263	8m	TargetScan
miR-7161-5p	ENSG00000139318	8m	TargetScan

miR-7161-5p	ENSG00000139351	8m	TargetScan
miR-7161-5p	ENSG00000139620	8m	TargetScan
miR-7161-5p	ENSG00000139631	8m	TargetScan
miR-7161-5p	ENSG00000139679	8m	TargetScan
miR-7161-5p	ENSG00000139734	8m	TargetScan
miR-7161-5p	ENSG00000139977	8m	TargetScan
miR-7161-5p	ENSG00000140280	8m	TargetScan
miR-7161-5p	ENSG00000140403	8m	TargetScan
miR-7161-5p	ENSG00000140406	8m	TargetScan
miR-7161-5p	ENSG00000140941	8m	TargetScan
miR-7161-5p	ENSG00000141404	8m	TargetScan
miR-7161-5p	ENSG00000141441	8m	TargetScan
miR-7161-5p	ENSG00000141446	8m	TargetScan
miR-7161-5p	ENSG00000141449	8m	TargetScan
miR-7161-5p	ENSG00000141655	8m	TargetScan
miR-7161-5p	ENSG00000141858	8m	TargetScan
miR-7161-5p	ENSG00000141873	8m	TargetScan
miR-7161-5p	ENSG00000142192	8m	TargetScan
miR-7161-5p	ENSG00000142556	8m	TargetScan
miR-7161-5p	ENSG00000142621	8m	TargetScan
miR-7161-5p	ENSG00000142623	8m	TargetScan
miR-7161-5p	ENSG00000143061	8m	TargetScan
miR-7161-5p	ENSG00000143420	8m	TargetScan
miR-7161-5p	ENSG00000143493	8m	TargetScan
miR-7161-5p	ENSG00000143627	8m	TargetScan
miR-7161-5p	ENSG00000143786	8m	TargetScan
miR-7161-5p	ENSG00000143815	8m	TargetScan
miR-7161-5p	ENSG00000143995	8m	TargetScan
miR-7161-5p	ENSG00000144057	8m	TargetScan
miR-7161-5p	ENSG00000144229	8m	TargetScan
miR-7161-5p	ENSG00000144366	8m	TargetScan

miR-7161-5p	ENSG00000144401	8m	TargetScan
miR-7161-5p	ENSG00000144451	8m	TargetScan
miR-7161-5p	ENSG00000144455	8m	TargetScan
miR-7161-5p	ENSG00000144597	8m	TargetScan
miR-7161-5p	ENSG00000144645	8m	TargetScan
miR-7161-5p	ENSG00000144824	8m	TargetScan
miR-7161-5p	ENSG00000144959	8m	TargetScan
miR-7161-5p	ENSG00000145012	8m	TargetScan
miR-7161-5p	ENSG00000145014	8m	TargetScan
miR-7161-5p	ENSG00000145147	8m	TargetScan
miR-7161-5p	ENSG00000145216	8m	TargetScan
miR-7161-5p	ENSG00000145384	8m	TargetScan
miR-7161-5p	ENSG00000145414	8m	TargetScan
miR-7161-5p	ENSG00000145439	8m	TargetScan
miR-7161-5p	ENSG00000145451	8m	TargetScan
miR-7161-5p	ENSG00000145592	8m	TargetScan
miR-7161-5p	ENSG00000145687	8m	TargetScan
miR-7161-5p	ENSG00000145907	8m	TargetScan
miR-7161-5p	ENSG00000146072	8m	TargetScan
miR-7161-5p	ENSG00000146263	8m	TargetScan
miR-7161-5p	ENSG00000146267	8m	TargetScan
miR-7161-5p	ENSG00000146282	8m	TargetScan
miR-7161-5p	ENSG00000146350	8m	TargetScan
miR-7161-5p	ENSG00000146411	8m	TargetScan
miR-7161-5p	ENSG00000147119	8m	TargetScan
miR-7161-5p	ENSG00000147138	8m	TargetScan
miR-7161-5p	ENSG00000147548	8m	TargetScan
miR-7161-5p	ENSG00000147604	8m	TargetScan
miR-7161-5p	ENSG00000147679	8m	TargetScan
miR-7161-5p	ENSG00000147687	8m	TargetScan
miR-7161-5p	ENSG00000147852	8m	TargetScan

miR-7161-5p	ENSG00000147853	8m	TargetScan
miR-7161-5p	ENSG00000147862	8m	TargetScan
miR-7161-5p	ENSG00000148187	8m	TargetScan
miR-7161-5p	ENSG00000148225	8m	TargetScan
miR-7161-5p	ENSG00000148600	8m	TargetScan
miR-7161-5p	ENSG00000148672	8m	TargetScan
miR-7161-5p	ENSG00000148688	8m	TargetScan
miR-7161-5p	ENSG00000148730	8m	TargetScan
miR-7161-5p	ENSG00000148737	8m	TargetScan
miR-7161-5p	ENSG00000149212	8m	TargetScan
miR-7161-5p	ENSG00000149308	8m	TargetScan
miR-7161-5p	ENSG00000149483	8m	TargetScan
miR-7161-5p	ENSG00000149948	8m	TargetScan
miR-7161-5p	ENSG00000150681	8m	TargetScan
miR-7161-5p	ENSG00000150938	8m	TargetScan
miR-7161-5p	ENSG00000151466	8m	TargetScan
miR-7161-5p	ENSG00000151657	8m	TargetScan
miR-7161-5p	ENSG00000151692	8m	TargetScan
miR-7161-5p	ENSG00000151729	8m	TargetScan
miR-7161-5p	ENSG00000151746	8m	TargetScan
miR-7161-5p	ENSG00000151834	8m	TargetScan
miR-7161-5p	ENSG00000152402	8m	TargetScan
miR-7161-5p	ENSG00000152492	8m	TargetScan
miR-7161-5p	ENSG00000152582	8m	TargetScan
miR-7161-5p	ENSG00000152670	8m	TargetScan
miR-7161-5p	ENSG00000152766	8m	TargetScan
miR-7161-5p	ENSG00000153046	8m	TargetScan
miR-7161-5p	ENSG00000153140	8m	TargetScan
miR-7161-5p	ENSG00000153790	8m	TargetScan
miR-7161-5p	ENSG00000153898	8m	TargetScan
miR-7161-5p	ENSG00000153904	8m	TargetScan

miR-7161-5p	ENSG00000154415	8m	TargetScan
miR-7161-5p	ENSG00000154429	8m	TargetScan
miR-7161-5p	ENSG00000154640	8m	TargetScan
miR-7161-5p	ENSG00000154654	8m	TargetScan
miR-7161-5p	ENSG00000154734	8m	TargetScan
miR-7161-5p	ENSG00000154845	8m	TargetScan
miR-7161-5p	ENSG00000155085	8m	TargetScan
miR-7161-5p	ENSG00000155099	8m	TargetScan
miR-7161-5p	ENSG00000155380	8m	TargetScan
miR-7161-5p	ENSG00000155438	8m	TargetScan
miR-7161-5p	ENSG00000155629	8m	TargetScan
miR-7161-5p	ENSG00000155827	8m	TargetScan
miR-7161-5p	ENSG00000155850	8m	TargetScan
miR-7161-5p	ENSG00000155876	8m	TargetScan
miR-7161-5p	ENSG00000155886	8m	TargetScan
miR-7161-5p	ENSG00000155903	8m	TargetScan
miR-7161-5p	ENSG00000155957	8m	TargetScan
miR-7161-5p	ENSG00000156030	8m	TargetScan
miR-7161-5p	ENSG00000156973	8m	TargetScan
miR-7161-5p	ENSG00000157224	8m	TargetScan
miR-7161-5p	ENSG00000157429	8m	TargetScan
miR-7161-5p	ENSG00000157483	8m	TargetScan
miR-7161-5p	ENSG00000157542	8m	TargetScan
miR-7161-5p	ENSG00000157916	8m	TargetScan
miR-7161-5p	ENSG00000158042	8m	TargetScan
miR-7161-5p	ENSG00000159212	8m	TargetScan
miR-7161-5p	ENSG00000159256	8m	TargetScan
miR-7161-5p	ENSG00000159388	8m	TargetScan
miR-7161-5p	ENSG00000159579	8m	TargetScan
miR-7161-5p	ENSG00000160216	8m	TargetScan
miR-7161-5p	ENSG00000160218	8m	TargetScan

miR-7161-5p	ENSG00000160310	8m	TargetScan
miR-7161-5p	ENSG00000160321	8m	TargetScan
miR-7161-5p	ENSG00000160551	8m	TargetScan
miR-7161-5p	ENSG00000161405	8m	TargetScan
miR-7161-5p	ENSG00000161551	8m	TargetScan
miR-7161-5p	ENSG00000161654	8m	TargetScan
miR-7161-5p	ENSG00000162105	8m	TargetScan
miR-7161-5p	ENSG00000162367	8m	TargetScan
miR-7161-5p	ENSG00000162374	8m	TargetScan
miR-7161-5p	ENSG00000162433	8m	TargetScan
miR-7161-5p	ENSG00000162599	8m	TargetScan
miR-7161-5p	ENSG00000162614	8m	TargetScan
miR-7161-5p	ENSG00000162654	8m	TargetScan
miR-7161-5p	ENSG00000162849	8m	TargetScan
miR-7161-5p	ENSG00000162877	8m	TargetScan
miR-7161-5p	ENSG00000163026	8m	TargetScan
miR-7161-5p	ENSG00000163125	8m	TargetScan
miR-7161-5p	ENSG00000163131	8m	TargetScan
miR-7161-5p	ENSG00000163162	8m	TargetScan
miR-7161-5p	ENSG00000163378	8m	TargetScan
miR-7161-5p	ENSG00000163428	8m	TargetScan
miR-7161-5p	ENSG00000163519	8m	TargetScan
miR-7161-5p	ENSG00000163558	8m	TargetScan
miR-7161-5p	ENSG00000163590	8m	TargetScan
miR-7161-5p	ENSG00000163644	8m	TargetScan
miR-7161-5p	ENSG00000163646	8m	TargetScan
miR-7161-5p	ENSG00000163660	8m	TargetScan
miR-7161-5p	ENSG00000163735	8m	TargetScan
miR-7161-5p	ENSG00000163755	8m	TargetScan
miR-7161-5p	ENSG00000163832	8m	TargetScan
miR-7161-5p	ENSG00000163866	8m	TargetScan

miR-7161-5p	ENSG00000163950	8m	TargetScan
miR-7161-5p	ENSG00000164035	8m	TargetScan
miR-7161-5p	ENSG00000164048	8m	TargetScan
miR-7161-5p	ENSG00000164070	8m	TargetScan
miR-7161-5p	ENSG00000164118	8m	TargetScan
miR-7161-5p	ENSG00000164161	8m	TargetScan
miR-7161-5p	ENSG00000164167	8m	TargetScan
miR-7161-5p	ENSG00000164168	8m	TargetScan
miR-7161-5p	ENSG00000164180	8m	TargetScan
miR-7161-5p	ENSG00000164181	8m	TargetScan
miR-7161-5p	ENSG00000164253	8m	TargetScan
miR-7161-5p	ENSG00000164292	8m	TargetScan
miR-7161-5p	ENSG00000164294	8m	TargetScan
miR-7161-5p	ENSG00000164303	8m	TargetScan
miR-7161-5p	ENSG00000164402	8m	TargetScan
miR-7161-5p	ENSG00000164442	8m	TargetScan
miR-7161-5p	ENSG00000164659	8m	TargetScan
miR-7161-5p	ENSG00000164663	8m	TargetScan
miR-7161-5p	ENSG00000164684	8m	TargetScan
miR-7161-5p	ENSG00000164715	8m	TargetScan
miR-7161-5p	ENSG00000165023	8m	TargetScan
miR-7161-5p	ENSG00000165028	8m	TargetScan
miR-7161-5p	ENSG00000165061	8m	TargetScan
miR-7161-5p	ENSG00000165105	8m	TargetScan
miR-7161-5p	ENSG00000165186	8m	TargetScan
miR-7161-5p	ENSG00000165219	8m	TargetScan
miR-7161-5p	ENSG00000165521	8m	TargetScan
miR-7161-5p	ENSG00000165526	8m	TargetScan
miR-7161-5p	ENSG00000165678	8m	TargetScan
miR-7161-5p	ENSG00000165682	8m	TargetScan
miR-7161-5p	ENSG00000165694	8m	TargetScan

miR-7161-5p	ENSG00000165732	8m	TargetScan
miR-7161-5p	ENSG00000165819	8m	TargetScan
miR-7161-5p	ENSG00000165891	8m	TargetScan
miR-7161-5p	ENSG00000165895	8m	TargetScan
miR-7161-5p	ENSG00000165972	8m	TargetScan
miR-7161-5p	ENSG00000165985	8m	TargetScan
miR-7161-5p	ENSG00000166135	8m	TargetScan
miR-7161-5p	ENSG00000166173	8m	TargetScan
miR-7161-5p	ENSG00000166200	8m	TargetScan
miR-7161-5p	ENSG00000166446	8m	TargetScan
miR-7161-5p	ENSG00000166471	8m	TargetScan
miR-7161-5p	ENSG00000166479	8m	TargetScan
miR-7161-5p	ENSG00000166762	8m	TargetScan
miR-7161-5p	ENSG00000166793	8m	TargetScan
miR-7161-5p	ENSG00000167555	8m	TargetScan
miR-7161-5p	ENSG00000167695	8m	TargetScan
miR-7161-5p	ENSG00000167702	8m	TargetScan
miR-7161-5p	ENSG00000167985	8m	TargetScan
miR-7161-5p	ENSG00000168010	8m	TargetScan
miR-7161-5p	ENSG00000168079	8m	TargetScan
miR-7161-5p	ENSG00000168172	8m	TargetScan
miR-7161-5p	ENSG00000168556	8m	TargetScan
miR-7161-5p	ENSG00000168672	8m	TargetScan
miR-7161-5p	ENSG00000168772	8m	TargetScan
miR-7161-5p	ENSG00000168792	8m	TargetScan
miR-7161-5p	ENSG00000168876	8m	TargetScan
miR-7161-5p	ENSG00000168887	8m	TargetScan
miR-7161-5p	ENSG00000168939	8m	TargetScan
miR-7161-5p	ENSG00000168939	8m	TargetScan
miR-7161-5p	ENSG00000168955	8m	TargetScan
miR-7161-5p	ENSG00000169021	8m	TargetScan

miR-7161-5p	ENSG00000169035	8m	TargetScan
miR-7161-5p	ENSG00000169057	8m	TargetScan
miR-7161-5p	ENSG00000169139	8m	TargetScan
miR-7161-5p	ENSG00000169224	8m	TargetScan
miR-7161-5p	ENSG00000169446	8m	TargetScan
miR-7161-5p	ENSG00000169604	8m	TargetScan
miR-7161-5p	ENSG00000169752	8m	TargetScan
miR-7161-5p	ENSG00000169891	8m	TargetScan
miR-7161-5p	ENSG00000169967	8m	TargetScan
miR-7161-5p	ENSG00000170006	8m	TargetScan
miR-7161-5p	ENSG00000170027	8m	TargetScan
miR-7161-5p	ENSG00000170145	8m	TargetScan
miR-7161-5p	ENSG00000170160	8m	TargetScan
miR-7161-5p	ENSG00000170248	8m	TargetScan
miR-7161-5p	ENSG00000170312	8m	TargetScan
miR-7161-5p	ENSG00000170325	8m	TargetScan
miR-7161-5p	ENSG00000170571	8m	TargetScan
miR-7161-5p	ENSG00000170584	8m	TargetScan
miR-7161-5p	ENSG00000170632	8m	TargetScan
miR-7161-5p	ENSG00000170734	8m	TargetScan
miR-7161-5p	ENSG00000170759	8m	TargetScan
miR-7161-5p	ENSG00000171105	8m	TargetScan
miR-7161-5p	ENSG00000171121	8m	TargetScan
miR-7161-5p	ENSG00000171316	8m	TargetScan
miR-7161-5p	ENSG00000171320	8m	TargetScan
miR-7161-5p	ENSG00000171444	8m	TargetScan
miR-7161-5p	ENSG00000171469	8m	TargetScan
miR-7161-5p	ENSG00000171488	8m	TargetScan
miR-7161-5p	ENSG00000171502	8m	TargetScan
miR-7161-5p	ENSG00000171522	8m	TargetScan
miR-7161-5p	ENSG00000171540	8m	TargetScan

miR-7161-5p	ENSG00000171759	8m	TargetScan
miR-7161-5p	ENSG00000171791	8m	TargetScan
miR-7161-5p	ENSG00000171914	8m	TargetScan
miR-7161-5p	ENSG00000172469	8m	TargetScan
miR-7161-5p	ENSG00000172476	8m	TargetScan
miR-7161-5p	ENSG00000172572	8m	TargetScan
miR-7161-5p	ENSG00000172845	8m	TargetScan
miR-7161-5p	ENSG00000172901	8m	TargetScan
miR-7161-5p	ENSG00000172939	8m	TargetScan
miR-7161-5p	ENSG00000172954	8m	TargetScan
miR-7161-5p	ENSG00000172985	8m	TargetScan
miR-7161-5p	ENSG00000173041	8m	TargetScan
miR-7161-5p	ENSG00000173080	8m	TargetScan
miR-7161-5p	ENSG00000173083	8m	TargetScan
miR-7161-5p	ENSG00000173141	8m	TargetScan
miR-7161-5p	ENSG00000173166	8m	TargetScan
miR-7161-5p	ENSG00000173200	8m	TargetScan
miR-7161-5p	ENSG00000173207	8m	TargetScan
miR-7161-5p	ENSG00000173578	8m	TargetScan
miR-7161-5p	ENSG00000173597	8m	TargetScan
miR-7161-5p	ENSG00000173744	8m	TargetScan
miR-7161-5p	ENSG00000173875	8m	TargetScan
miR-7161-5p	ENSG00000173947	8m	TargetScan
miR-7161-5p	ENSG00000174032	8m	TargetScan
miR-7161-5p	ENSG00000174145	8m	TargetScan
miR-7161-5p	ENSG00000174243	8m	TargetScan
miR-7161-5p	ENSG00000174579	8m	TargetScan
miR-7161-5p	ENSG00000174669	8m	TargetScan
miR-7161-5p	ENSG00000174738	8m	TargetScan
miR-7161-5p	ENSG00000174749	8m	TargetScan
miR-7161-5p	ENSG00000175066	8m	TargetScan

miR-7161-5p	ENSG00000175104	8m	TargetScan
miR-7161-5p	ENSG00000175264	8m	TargetScan
miR-7161-5p	ENSG00000175387	8m	TargetScan
miR-7161-5p	ENSG00000175414	8m	TargetScan
miR-7161-5p	ENSG00000175449	8m	TargetScan
miR-7161-5p	ENSG00000175548	8m	TargetScan
miR-7161-5p	ENSG00000175745	8m	TargetScan
miR-7161-5p	ENSG00000175874	8m	TargetScan
miR-7161-5p	ENSG00000176142	8m	TargetScan
miR-7161-5p	ENSG00000176399	8m	TargetScan
miR-7161-5p	ENSG00000176853	8m	TargetScan
miR-7161-5p	ENSG00000176890	8m	TargetScan
miR-7161-5p	ENSG00000177034	8m	TargetScan
miR-7161-5p	ENSG00000177283	8m	TargetScan
miR-7161-5p	ENSG00000177485	8m	TargetScan
miR-7161-5p	ENSG00000177613	8m	TargetScan
miR-7161-5p	ENSG00000177889	8m	TargetScan
miR-7161-5p	ENSG00000178217	8m	TargetScan
miR-7161-5p	ENSG00000178951	8m	TargetScan
miR-7161-5p	ENSG00000179119	8m	TargetScan
miR-7161-5p	ENSG00000179299	8m	TargetScan
miR-7161-5p	ENSG00000179918	8m	TargetScan
miR-7161-5p	ENSG00000180440	8m	TargetScan
miR-7161-5p	ENSG00000180626	8m	TargetScan
miR-7161-5p	ENSG00000180917	8m	TargetScan
miR-7161-5p	ENSG00000180998	8m	TargetScan
miR-7161-5p	ENSG00000181192	8m	TargetScan
miR-7161-5p	ENSG00000181195	8m	TargetScan
miR-7161-5p	ENSG00000181220	8m	TargetScan
miR-7161-5p	ENSG00000181449	8m	TargetScan
miR-7161-5p	ENSG00000181467	8m	TargetScan

miR-7161-5p	ENSG00000181896	8m	TargetScan
miR-7161-5p	ENSG00000182077	8m	TargetScan
miR-7161-5p	ENSG00000182158	8m	TargetScan
miR-7161-5p	ENSG00000182247	8m	TargetScan
miR-7161-5p	ENSG00000182263	8m	TargetScan
miR-7161-5p	ENSG00000182307	8m	TargetScan
miR-7161-5p	ENSG00000182348	8m	TargetScan
miR-7161-5p	ENSG00000182389	8m	TargetScan
miR-7161-5p	ENSG00000182732	8m	TargetScan
miR-7161-5p	ENSG00000182810	8m	TargetScan
miR-7161-5p	ENSG00000182923	8m	TargetScan
miR-7161-5p	ENSG00000182957	8m	TargetScan
miR-7161-5p	ENSG00000183067	8m	TargetScan
miR-7161-5p	ENSG00000183098	8m	TargetScan
miR-7161-5p	ENSG00000183134	8m	TargetScan
miR-7161-5p	ENSG00000183309	8m	TargetScan
miR-7161-5p	ENSG00000183628	8m	TargetScan
miR-7161-5p	ENSG00000183726	8m	TargetScan
miR-7161-5p	ENSG00000183801	8m	TargetScan
miR-7161-5p	ENSG00000184005	8m	TargetScan
miR-7161-5p	ENSG00000184007	8m	TargetScan
miR-7161-5p	ENSG00000184258	8m	TargetScan
miR-7161-5p	ENSG00000184349	8m	TargetScan
miR-7161-5p	ENSG00000184374	8m	TargetScan
miR-7161-5p	ENSG00000184384	8m	TargetScan
miR-7161-5p	ENSG00000184402	8m	TargetScan
miR-7161-5p	ENSG00000184408	8m	TargetScan
miR-7161-5p	ENSG00000184486	8m	TargetScan
miR-7161-5p	ENSG00000184845	8m	TargetScan
miR-7161-5p	ENSG00000184916	8m	TargetScan
miR-7161-5p	ENSG00000185104	8m	TargetScan

miR-7161-5p	ENSG00000185238	8m	TargetScan
miR-7161-5p	ENSG00000185414	8m	TargetScan
miR-7161-5p	ENSG00000185515	8m	TargetScan
miR-7161-5p	ENSG00000185591	8m	TargetScan
miR-7161-5p	ENSG00000185753	8m	TargetScan
miR-7161-5p	ENSG00000185920	8m	TargetScan
miR-7161-5p	ENSG00000186063	8m	TargetScan
miR-7161-5p	ENSG00000186265	8m	TargetScan
miR-7161-5p	ENSG00000186479	8m	TargetScan
miR-7161-5p	ENSG00000186814	8m	TargetScan
miR-7161-5p	ENSG00000187098	8m	TargetScan
miR-7161-5p	ENSG00000187109	8m	TargetScan
miR-7161-5p	ENSG00000187140	8m	TargetScan
miR-7161-5p	ENSG00000187191	8m	TargetScan
miR-7161-5p	ENSG00000187231	8m	TargetScan
miR-7161-5p	ENSG00000187257	8m	TargetScan
miR-7161-5p	ENSG00000187522	8m	TargetScan
miR-7161-5p	ENSG00000187605	8m	TargetScan
miR-7161-5p	ENSG00000187742	8m	TargetScan
miR-7161-5p	ENSG00000187792	8m	TargetScan
miR-7161-5p	ENSG00000187866	8m	TargetScan
miR-7161-5p	ENSG00000187889	8m	TargetScan
miR-7161-5p	ENSG00000188120	8m	TargetScan
miR-7161-5p	ENSG00000188133	8m	TargetScan
miR-7161-5p	ENSG00000188167	8m	TargetScan
miR-7161-5p	ENSG00000188321	8m	TargetScan
miR-7161-5p	ENSG00000188522	8m	TargetScan
miR-7161-5p	ENSG00000188612	8m	TargetScan
miR-7161-5p	ENSG00000188647	8m	TargetScan
miR-7161-5p	ENSG00000188994	8m	TargetScan
miR-7161-5p	ENSG00000189195	8m	TargetScan

miR-7161-5p	ENSG00000189350	8m	TargetScan
miR-7161-5p	ENSG00000196090	8m	TargetScan
miR-7161-5p	ENSG00000196209	8m	TargetScan
miR-7161-5p	ENSG00000196458	8m	TargetScan
miR-7161-5p	ENSG00000196466	8m	TargetScan
miR-7161-5p	ENSG00000196505	8m	TargetScan
miR-7161-5p	ENSG00000196659	8m	TargetScan
miR-7161-5p	ENSG00000196678	8m	TargetScan
miR-7161-5p	ENSG00000196812	8m	TargetScan
miR-7161-5p	ENSG00000196937	8m	TargetScan
miR-7161-5p	ENSG00000196946	8m	TargetScan
miR-7161-5p	ENSG00000197081	8m	TargetScan
miR-7161-5p	ENSG00000197121	8m	TargetScan
miR-7161-5p	ENSG00000197142	8m	TargetScan
miR-7161-5p	ENSG00000197147	8m	TargetScan
miR-7161-5p	ENSG00000197472	8m	TargetScan
miR-7161-5p	ENSG00000197535	8m	TargetScan
miR-7161-5p	ENSG00000197557	8m	TargetScan
miR-7161-5p	ENSG00000197566	8m	TargetScan
miR-7161-5p	ENSG00000197579	8m	TargetScan
miR-7161-5p	ENSG00000197651	8m	TargetScan
miR-7161-5p	ENSG00000197713	8m	TargetScan
miR-7161-5p	ENSG00000197779	8m	TargetScan
miR-7161-5p	ENSG00000197930	8m	TargetScan
miR-7161-5p	ENSG00000198046	8m	TargetScan
miR-7161-5p	ENSG00000198087	8m	TargetScan
miR-7161-5p	ENSG00000198157	8m	TargetScan
miR-7161-5p	ENSG00000198408	8m	TargetScan
miR-7161-5p	ENSG00000198464	8m	TargetScan
miR-7161-5p	ENSG00000198612	8m	TargetScan
miR-7161-5p	ENSG00000198668	8m	TargetScan

miR-7161-5p	ENSG00000198739	8m	TargetScan
miR-7161-5p	ENSG00000198771	8m	TargetScan
miR-7161-5p	ENSG00000198791	8m	TargetScan
miR-7161-5p	ENSG00000198799	8m	TargetScan
miR-7161-5p	ENSG00000198812	8m	TargetScan
miR-7161-5p	ENSG00000198824	8m	TargetScan
miR-7161-5p	ENSG00000198865	8m	TargetScan
miR-7161-5p	ENSG00000198873	8m	TargetScan
miR-7161-5p	ENSG00000198898	8m	TargetScan
miR-7161-5p	ENSG00000198929	8m	TargetScan
miR-7161-5p	ENSG00000203485	8m	TargetScan
miR-7161-5p	ENSG00000204033	8m	TargetScan
miR-7161-5p	ENSG00000204279	8m	TargetScan
miR-7161-5p	ENSG00000204688	8m	TargetScan
miR-7161-5p	ENSG00000204977	8m	TargetScan
miR-7161-5p	ENSG00000205758	8m	TargetScan
miR-7161-5p	ENSG00000205835	8m	TargetScan
miR-7161-5p	ENSG00000205916	8m	TargetScan
miR-7161-5p	ENSG00000205944	8m	TargetScan
miR-7161-5p	ENSG00000206432	8m	TargetScan
miR-7161-5p	ENSG00000206538	8m	TargetScan
miR-7161-5p	ENSG00000211455	8m	TargetScan
miR-7161-5p	ENSG00000211456	8m	TargetScan
miR-7161-5p	ENSG00000213625	8m	TargetScan
miR-7161-5p	ENSG00000213762	8m	TargetScan
miR-7161-5p	ENSG00000213967	8m	TargetScan
miR-7161-5p	ENSG00000213973	8m	TargetScan
miR-7161-5p	ENSG00000214216	8m	TargetScan
miR-7161-5p	ENSG00000214827	8m	TargetScan
miR-7161-5p	ENSG00000215301	8m	TargetScan
miR-7161-5p	ENSG00000235376	8m	TargetScan

miR-7161-5p	ENSG00000237353	8m	TargetScan
miR-7161-5p	ENSG00000239900	8m	TargetScan
miR-7161-5p	ENSG00000240654	8m	TargetScan
miR-7161-5p	ENSG00000241399	8m	TargetScan
miR-7161-5p	ENSG00000241489	8m	TargetScan
miR-7161-5p	ENSG00000242110	8m	TargetScan
miR-7161-5p	ENSG00000242220	8m	TargetScan
miR-7161-5p	ENSG00000242689	8m	TargetScan
miR-7161-5p	ENSG00000244754	8m	TargetScan
miR-7161-5p	ENSG00000248672	8m	TargetScan
miR-7161-5p	ENSG00000249884	8m	TargetScan
miR-7161-5p	ENSG00000255112	8m	TargetScan
miR-7161-5p	ENSG00000255529	8m	TargetScan
miR-7161-5p	ENSG00000255561	8m	TargetScan
miR-7161-5p	ENSG00000256771	8m	TargetScan
miR-7161-5p	ENSG00000257127	8m	TargetScan
miR-7161-5p	ENSG00000259207	8m	TargetScan
miR-7161-5p	ENSG00000259458	8m	TargetScan
miR-7161-5p	ENSG00000259495	8m	TargetScan
miR-7161-5p	ENSG00000260230	8m	TargetScan
miR-7161-5p	ENSG00000267041	8m	TargetScan
miR-7161-5p	ENSG00000267618	8m	TargetScan
miR-7161-5p	ENSG00000273079	8m	TargetScan
miR-7161-5p	ENSG00000273274	8m	TargetScan
miR-572	ENSG00000011485	8m	TargetScan
miR-572	ENSG00000039068	8m	TargetScan
miR-572	ENSG00000066739	8m	TargetScan
miR-572	ENSG00000070269	8m	TargetScan
miR-572	ENSG00000078061	8m	TargetScan
miR-572	ENSG00000078687	8m	TargetScan
miR-572	ENSG00000086232	8m	TargetScan

miR-572	ENSG00000089250	8m	TargetScan
miR-572	ENSG00000099769	8m	TargetScan
miR-572	ENSG00000100014	8m	TargetScan
miR-572	ENSG00000100364	8m	TargetScan
miR-572	ENSG00000100804	8m	TargetScan
miR-572	ENSG00000101104	8m	TargetScan
miR-572	ENSG00000102935	8m	TargetScan
miR-572	ENSG00000103534	8m	TargetScan
miR-572	ENSG00000114933	8m	TargetScan
miR-572	ENSG00000116731	8m	TargetScan
miR-572	ENSG00000125046	8m	TargetScan
miR-572	ENSG00000129473	8m	TargetScan
miR-572	ENSG00000130669	8m	TargetScan
miR-572	ENSG00000131153	8m	TargetScan
miR-572	ENSG00000132792	8m	TargetScan
miR-572	ENSG00000132932	8m	TargetScan
miR-572	ENSG00000136425	8m	TargetScan
miR-572	ENSG00000137491	8m	TargetScan
miR-572	ENSG00000138622	8m	TargetScan
miR-572	ENSG00000140527	8m	TargetScan
miR-572	ENSG00000140937	8m	TargetScan
miR-572	ENSG00000142599	8m	TargetScan
miR-572	ENSG00000143032	8m	TargetScan
miR-572	ENSG00000145012	8m	TargetScan
miR-572	ENSG00000145016	8m	TargetScan
miR-572	ENSG00000145734	8m	TargetScan
miR-572	ENSG00000149294	8m	TargetScan
miR-572	ENSG00000149403	8m	TargetScan
miR-572	ENSG00000149930	8m	TargetScan
miR-572	ENSG00000152822	8m	TargetScan
miR-572	ENSG00000154227	8m	TargetScan

miR-572	ENSG00000158859	8m	TargetScan
miR-572	ENSG00000158985	8m	TargetScan
miR-572	ENSG00000160683	8m	TargetScan
miR-572	ENSG00000160688	8m	TargetScan
miR-572	ENSG00000160862	8m	TargetScan
miR-572	ENSG00000160953	8m	TargetScan
miR-572	ENSG00000161526	8m	TargetScan
miR-572	ENSG00000162849	8m	TargetScan
miR-572	ENSG00000164402	8m	TargetScan
miR-572	ENSG00000165169	8m	TargetScan
miR-572	ENSG00000168439	8m	TargetScan
miR-572	ENSG00000168490	8m	TargetScan
miR-572	ENSG00000169223	8m	TargetScan
miR-572	ENSG00000169992	8m	TargetScan
miR-572	ENSG00000170525	8m	TargetScan
miR-572	ENSG00000172578	8m	TargetScan
miR-572	ENSG00000172889	8m	TargetScan
miR-572	ENSG00000173210	8m	TargetScan
miR-572	ENSG00000173517	8m	TargetScan
miR-572	ENSG00000175727	8m	TargetScan
miR-572	ENSG00000176658	8m	TargetScan
miR-572	ENSG00000176853	8m	TargetScan
miR-572	ENSG00000177300	8m	TargetScan
miR-572	ENSG00000182473	8m	TargetScan
miR-572	ENSG00000182551	8m	TargetScan
miR-572	ENSG00000182704	8m	TargetScan
miR-572	ENSG00000183153	8m	TargetScan
miR-572	ENSG00000183762	8m	TargetScan
miR-572	ENSG00000183826	8m	TargetScan
miR-572	ENSG00000184471	8m	TargetScan
miR-572	ENSG00000184992	8m	TargetScan

miR-572	ENSG00000186575	8m	TargetScan
miR-572	ENSG00000186591	8m	TargetScan
miR-572	ENSG00000187912	8m	TargetScan
miR-572	ENSG00000196182	8m	TargetScan
miR-572	ENSG00000196557	8m	TargetScan
miR-572	ENSG00000197312	8m	TargetScan
miR-572	ENSG00000198198	8m	TargetScan
miR-572	ENSG00000204283	8m	TargetScan
miR-572	ENSG00000205176	8m	TargetScan
miR-572	ENSG00000213614	8m	TargetScan
miR-572	ENSG00000220205	8m	TargetScan
miR-572	ENSG00000254772	8m	TargetScan
miR-572	ENSG00000257103	8m	TargetScan
miR-572	ENSG00000258436	8m	TargetScan
miR-572	ENSG00000266173	8m	TargetScan
miR-622	ENSG00000001167	8m	TargetScan
miR-622	ENSG00000001460	8m	TargetScan
miR-622	ENSG00000002933	8m	TargetScan
miR-622	ENSG00000004468	8m	TargetScan
miR-622	ENSG00000005249	8m	TargetScan
miR-622	ENSG00000005884	8m	TargetScan
miR-622	ENSG00000005981	8m	TargetScan
miR-622	ENSG00000006194	8m	TargetScan
miR-622	ENSG00000006377	8m	TargetScan
miR-622	ENSG00000006555	8m	TargetScan
miR-622	ENSG00000007944	8m	TargetScan
miR-622	ENSG00000008256	8m	TargetScan
miR-622	ENSG00000009844	8m	TargetScan
miR-622	ENSG00000010671	8m	TargetScan
miR-622	ENSG00000011007	8m	TargetScan
miR-622	ENSG00000011198	8m	TargetScan

miR-622	ENSG00000013016	8m	TargetScan
miR-622	ENSG00000013275	8m	TargetScan
miR-622	ENSG00000014824	8m	TargetScan
miR-622	ENSG00000015153	8m	TargetScan
miR-622	ENSG00000020922	8m	TargetScan
miR-622	ENSG00000021776	8m	TargetScan
miR-622	ENSG00000023318	8m	TargetScan
miR-622	ENSG00000025434	8m	TargetScan
miR-622	ENSG00000025772	8m	TargetScan
miR-622	ENSG00000026652	8m	TargetScan
miR-622	ENSG00000029639	8m	TargetScan
miR-622	ENSG00000032219	8m	TargetScan
miR-622	ENSG00000033627	8m	TargetScan
miR-622	ENSG00000036828	8m	TargetScan
miR-622	ENSG00000042781	8m	TargetScan
miR-622	ENSG00000043093	8m	TargetScan
miR-622	ENSG00000043514	8m	TargetScan
miR-622	ENSG00000044115	8m	TargetScan
miR-622	ENSG00000048649	8m	TargetScan
miR-622	ENSG00000050748	8m	TargetScan
miR-622	ENSG00000052344	8m	TargetScan
miR-622	ENSG00000052841	8m	TargetScan
miR-622	ENSG00000054965	8m	TargetScan
miR-622	ENSG00000056277	8m	TargetScan
miR-622	ENSG00000056558	8m	TargetScan
miR-622	ENSG00000057935	8m	TargetScan
miR-622	ENSG00000058729	8m	TargetScan
miR-622	ENSG00000058866	8m	TargetScan
miR-622	ENSG00000062716	8m	TargetScan
miR-622	ENSG00000064199	8m	TargetScan
miR-622	ENSG00000064999	8m	TargetScan

miR-622	ENSG00000065427	8m	TargetScan
miR-622	ENSG00000066294	8m	TargetScan
miR-622	ENSG00000069431	8m	TargetScan
miR-622	ENSG00000069812	8m	TargetScan
miR-622	ENSG00000070476	8m	TargetScan
miR-622	ENSG00000070614	8m	TargetScan
miR-622	ENSG00000070778	8m	TargetScan
miR-622	ENSG00000070961	8m	TargetScan
miR-622	ENSG00000071242	8m	TargetScan
miR-622	ENSG00000071575	8m	TargetScan
miR-622	ENSG00000072062	8m	TargetScan
miR-622	ENSG00000072135	8m	TargetScan
miR-622	ENSG00000074370	8m	TargetScan
miR-622	ENSG00000074590	8m	TargetScan
miR-622	ENSG00000074695	8m	TargetScan
miR-622	ENSG00000075884	8m	TargetScan
miR-622	ENSG00000076554	8m	TargetScan
miR-622	ENSG00000076650	8m	TargetScan
miR-622	ENSG00000077235	8m	TargetScan
miR-622	ENSG00000077549	8m	TargetScan
miR-622	ENSG00000077684	8m	TargetScan
miR-622	ENSG00000078053	8m	TargetScan
miR-622	ENSG00000078140	8m	TargetScan
miR-622	ENSG00000078399	8m	TargetScan
miR-622	ENSG00000078618	8m	TargetScan
miR-622	ENSG00000078900	8m	TargetScan
miR-622	ENSG00000079950	8m	TargetScan
miR-622	ENSG00000080224	8m	TargetScan
miR-622	ENSG00000081189	8m	TargetScan
miR-622	ENSG00000081760	8m	TargetScan
miR-622	ENSG00000082701	8m	TargetScan

miR-622	ENSG00000082781	8m	TargetScan
miR-622	ENSG00000083520	8m	TargetScan
miR-622	ENSG00000083812	8m	TargetScan
miR-622	ENSG00000083844	8m	TargetScan
miR-622	ENSG00000084733	8m	TargetScan
miR-622	ENSG00000085721	8m	TargetScan
miR-622	ENSG00000086189	8m	TargetScan
miR-622	ENSG00000086232	8m	TargetScan
miR-622	ENSG00000086712	8m	TargetScan
miR-622	ENSG00000089248	8m	TargetScan
miR-622	ENSG00000090686	8m	TargetScan
miR-622	ENSG00000091879	8m	TargetScan
miR-622	ENSG00000091972	8m	TargetScan
miR-622	ENSG00000092853	8m	TargetScan
miR-622	ENSG00000093000	8m	TargetScan
miR-622	ENSG00000093010	8m	TargetScan
miR-622	ENSG00000095637	8m	TargetScan
miR-622	ENSG00000096872	8m	TargetScan
miR-622	ENSG00000097007	8m	TargetScan
miR-622	ENSG00000099999	8m	TargetScan
miR-622	ENSG00000100027	8m	TargetScan
miR-622	ENSG00000100084	8m	TargetScan
miR-622	ENSG00000100345	8m	TargetScan
miR-622	ENSG00000100351	8m	TargetScan
miR-622	ENSG00000100368	8m	TargetScan
miR-622	ENSG00000100376	8m	TargetScan
miR-622	ENSG00000100441	8m	TargetScan
miR-622	ENSG00000100568	8m	TargetScan
miR-622	ENSG00000101193	8m	TargetScan
miR-622	ENSG00000101266	8m	TargetScan
miR-622	ENSG00000101280	8m	TargetScan

miR-622	ENSG00000101290	8m	TargetScan
miR-622	ENSG00000101307	8m	TargetScan
miR-622	ENSG00000101412	8m	TargetScan
miR-622	ENSG00000101445	8m	TargetScan
miR-622	ENSG00000101544	8m	TargetScan
miR-622	ENSG00000101596	8m	TargetScan
miR-622	ENSG00000101695	8m	TargetScan
miR-622	ENSG00000101945	8m	TargetScan
miR-622	ENSG00000102043	8m	TargetScan
miR-622	ENSG00000102317	8m	TargetScan
miR-622	ENSG00000102531	8m	TargetScan
miR-622	ENSG00000102780	8m	TargetScan
miR-622	ENSG00000102870	8m	TargetScan
miR-622	ENSG00000102908	8m	TargetScan
miR-622	ENSG00000103005	8m	TargetScan
miR-622	ENSG00000103021	8m	TargetScan
miR-622	ENSG00000103187	8m	TargetScan
miR-622	ENSG00000103351	8m	TargetScan
miR-622	ENSG00000103375	8m	TargetScan
miR-622	ENSG00000103404	8m	TargetScan
miR-622	ENSG00000103449	8m	TargetScan
miR-622	ENSG00000103507	8m	TargetScan
miR-622	ENSG00000103855	8m	TargetScan
miR-622	ENSG00000103995	8m	TargetScan
miR-622	ENSG00000104043	8m	TargetScan
miR-622	ENSG00000104067	8m	TargetScan
miR-622	ENSG00000104219	8m	TargetScan
miR-622	ENSG00000104313	8m	TargetScan
miR-622	ENSG00000104332	8m	TargetScan
miR-622	ENSG00000104361	8m	TargetScan
miR-622	ENSG00000104447	8m	TargetScan

miR-622	ENSG00000104824	8m	TargetScan
miR-622	ENSG00000104885	8m	TargetScan
miR-622	ENSG00000104886	8m	TargetScan
miR-622	ENSG00000104888	8m	TargetScan
miR-622	ENSG00000104967	8m	TargetScan
miR-622	ENSG00000105197	8m	TargetScan
miR-622	ENSG00000105429	8m	TargetScan
miR-622	ENSG00000105865	8m	TargetScan
miR-622	ENSG00000105866	8m	TargetScan
miR-622	ENSG00000105926	8m	TargetScan
miR-622	ENSG00000106261	8m	TargetScan
miR-622	ENSG00000106692	8m	TargetScan
miR-622	ENSG00000106723	8m	TargetScan
miR-622	ENSG00000106868	8m	TargetScan
miR-622	ENSG00000107130	8m	TargetScan
miR-622	ENSG00000107223	8m	TargetScan
miR-622	ENSG00000107249	8m	TargetScan
miR-622	ENSG00000107372	8m	TargetScan
miR-622	ENSG00000107611	8m	TargetScan
miR-622	ENSG00000107614	8m	TargetScan
miR-622	ENSG00000107771	8m	TargetScan
miR-622	ENSG00000107897	8m	TargetScan
miR-622	ENSG00000107960	8m	TargetScan
miR-622	ENSG00000108219	8m	TargetScan
miR-622	ENSG00000108423	8m	TargetScan
miR-622	ENSG00000108528	8m	TargetScan
miR-622	ENSG00000108559	8m	TargetScan
miR-622	ENSG00000108591	8m	TargetScan
miR-622	ENSG00000108799	8m	TargetScan
miR-622	ENSG00000108854	8m	TargetScan
miR-622	ENSG00000109046	8m	TargetScan

miR-622	ENSG00000109065	8m	TargetScan
miR-622	ENSG00000109667	8m	TargetScan
miR-622	ENSG00000109670	8m	TargetScan
miR-622	ENSG00000109685	8m	TargetScan
miR-622	ENSG00000110042	8m	TargetScan
miR-622	ENSG00000110104	8m	TargetScan
miR-622	ENSG00000110169	8m	TargetScan
miR-622	ENSG00000110321	8m	TargetScan
miR-622	ENSG00000110536	8m	TargetScan
miR-622	ENSG00000111110	8m	TargetScan
miR-622	ENSG00000111300	8m	TargetScan
miR-622	ENSG00000111361	8m	TargetScan
miR-622	ENSG00000111696	8m	TargetScan
miR-622	ENSG00000111817	8m	TargetScan
miR-622	ENSG00000111843	8m	TargetScan
miR-622	ENSG00000112033	8m	TargetScan
miR-622	ENSG00000112062	8m	TargetScan
miR-622	ENSG00000112081	8m	TargetScan
miR-622	ENSG00000112130	8m	TargetScan
miR-622	ENSG00000112137	8m	TargetScan
miR-622	ENSG00000112208	8m	TargetScan
miR-622	ENSG00000112232	8m	TargetScan
miR-622	ENSG00000112343	8m	TargetScan
miR-622	ENSG00000112419	8m	TargetScan
miR-622	ENSG00000112624	8m	TargetScan
miR-622	ENSG00000112769	8m	TargetScan
miR-622	ENSG00000113048	8m	TargetScan
miR-622	ENSG00000113140	8m	TargetScan
miR-622	ENSG00000113163	8m	TargetScan
miR-622	ENSG00000113303	8m	TargetScan
miR-622	ENSG00000113441	8m	TargetScan

miR-622	ENSG00000113494	8m	TargetScan
miR-622	ENSG00000113558	8m	TargetScan
miR-622	ENSG00000114302	8m	TargetScan
miR-622	ENSG00000114648	8m	TargetScan
miR-622	ENSG00000114738	8m	TargetScan
miR-622	ENSG00000114739	8m	TargetScan
miR-622	ENSG00000114805	8m	TargetScan
miR-622	ENSG00000115109	8m	TargetScan
miR-622	ENSG00000115163	8m	TargetScan
miR-622	ENSG00000115232	8m	TargetScan
miR-622	ENSG00000115286	8m	TargetScan
miR-622	ENSG00000115310	8m	TargetScan
miR-622	ENSG00000115540	8m	TargetScan
miR-622	ENSG00000115808	8m	TargetScan
miR-622	ENSG00000115825	8m	TargetScan
miR-622	ENSG00000115896	8m	TargetScan
miR-622	ENSG00000115966	8m	TargetScan
miR-622	ENSG00000116095	8m	TargetScan
miR-622	ENSG00000116132	8m	TargetScan
miR-622	ENSG00000116191	8m	TargetScan
miR-622	ENSG00000116209	8m	TargetScan
miR-622	ENSG00000116649	8m	TargetScan
miR-622	ENSG00000116984	8m	TargetScan
miR-622	ENSG00000117090	8m	TargetScan
miR-622	ENSG00000117139	8m	TargetScan
miR-622	ENSG00000117533	8m	TargetScan
miR-622	ENSG00000118217	8m	TargetScan
miR-622	ENSG00000118271	8m	TargetScan
miR-622	ENSG00000118620	8m	TargetScan
miR-622	ENSG00000118922	8m	TargetScan
miR-622	ENSG00000119121	8m	TargetScan

miR-622	ENSG00000119321	8m	TargetScan
miR-622	ENSG00000119402	8m	TargetScan
miR-622	ENSG00000119522	8m	TargetScan
miR-622	ENSG00000119685	8m	TargetScan
miR-622	ENSG00000119787	8m	TargetScan
miR-622	ENSG00000119865	8m	TargetScan
miR-622	ENSG00000119953	8m	TargetScan
miR-622	ENSG00000120662	8m	TargetScan
miR-622	ENSG00000120709	8m	TargetScan
miR-622	ENSG00000120942	8m	TargetScan
miR-622	ENSG00000120963	8m	TargetScan
miR-622	ENSG00000121060	8m	TargetScan
miR-622	ENSG00000121207	8m	TargetScan
miR-622	ENSG00000121361	8m	TargetScan
miR-622	ENSG00000121579	8m	TargetScan
miR-622	ENSG00000121957	8m	TargetScan
miR-622	ENSG00000122550	8m	TargetScan
miR-622	ENSG00000122574	8m	TargetScan
miR-622	ENSG00000123119	8m	TargetScan
miR-622	ENSG00000123407	8m	TargetScan
miR-622	ENSG00000123561	8m	TargetScan
miR-622	ENSG00000123643	8m	TargetScan
miR-622	ENSG00000124194	8m	TargetScan
miR-622	ENSG00000124243	8m	TargetScan
miR-622	ENSG00000124356	8m	TargetScan
miR-622	ENSG00000124486	8m	TargetScan
miR-622	ENSG00000124496	8m	TargetScan
miR-622	ENSG00000124523	8m	TargetScan
miR-622	ENSG00000124783	8m	TargetScan
miR-622	ENSG00000124788	8m	TargetScan
miR-622	ENSG00000125450	8m	TargetScan

miR-622	ENSG00000125629	8m	TargetScan
miR-622	ENSG00000125870	8m	TargetScan
miR-622	ENSG00000125885	8m	TargetScan
miR-622	ENSG00000125970	8m	TargetScan
miR-622	ENSG00000126070	8m	TargetScan
miR-622	ENSG00000126106	8m	TargetScan
miR-622	ENSG00000126214	8m	TargetScan
miR-622	ENSG00000126215	8m	TargetScan
miR-622	ENSG00000127334	8m	TargetScan
miR-622	ENSG00000127399	8m	TargetScan
miR-622	ENSG00000127418	8m	TargetScan
miR-622	ENSG00000127863	8m	TargetScan
miR-622	ENSG00000128050	8m	TargetScan
miR-622	ENSG00000128165	8m	TargetScan
miR-622	ENSG00000128383	8m	TargetScan
miR-622	ENSG00000128652	8m	TargetScan
miR-622	ENSG00000128655	8m	TargetScan
miR-622	ENSG00000129007	8m	TargetScan
miR-622	ENSG00000129173	8m	TargetScan
miR-622	ENSG00000130052	8m	TargetScan
miR-622	ENSG00000130054	8m	TargetScan
miR-622	ENSG00000130224	8m	TargetScan
miR-622	ENSG00000130517	8m	TargetScan
miR-622	ENSG00000130584	8m	TargetScan
miR-622	ENSG00000130640	8m	TargetScan
miR-622	ENSG00000130669	8m	TargetScan
miR-622	ENSG00000130723	8m	TargetScan
miR-622	ENSG00000130766	8m	TargetScan
miR-622	ENSG00000131149	8m	TargetScan
miR-622	ENSG00000131374	8m	TargetScan
miR-622	ENSG00000131386	8m	TargetScan

miR-622	ENSG00000131873	8m	TargetScan
miR-622	ENSG00000132394	8m	TargetScan
miR-622	ENSG00000132405	8m	TargetScan
miR-622	ENSG00000132964	8m	TargetScan
miR-622	ENSG00000133193	8m	TargetScan
miR-622	ENSG00000134046	8m	TargetScan
miR-622	ENSG00000134201	8m	TargetScan
miR-622	ENSG00000134250	8m	TargetScan
miR-622	ENSG00000134278	8m	TargetScan
miR-622	ENSG00000134294	8m	TargetScan
miR-622	ENSG00000134339	8m	TargetScan
miR-622	ENSG00000134775	8m	TargetScan
miR-622	ENSG00000135018	8m	TargetScan
miR-622	ENSG00000135362	8m	TargetScan
miR-622	ENSG00000135363	8m	TargetScan
miR-622	ENSG00000135426	8m	TargetScan
miR-622	ENSG00000135454	8m	TargetScan
miR-622	ENSG00000135473	8m	TargetScan
miR-622	ENSG00000135482	8m	TargetScan
miR-622	ENSG00000135604	8m	TargetScan
miR-622	ENSG00000135655	8m	TargetScan
miR-622	ENSG00000135823	8m	TargetScan
miR-622	ENSG00000135835	8m	TargetScan
miR-622	ENSG00000136014	8m	TargetScan
miR-622	ENSG00000136237	8m	TargetScan
miR-622	ENSG00000136279	8m	TargetScan
miR-622	ENSG00000136492	8m	TargetScan
miR-622	ENSG00000136807	8m	TargetScan
miR-622	ENSG00000136816	8m	TargetScan
miR-622	ENSG00000136828	8m	TargetScan
miR-622	ENSG00000136842	8m	TargetScan

miR-622	ENSG00000136877	8m	TargetScan
miR-622	ENSG00000136878	8m	TargetScan
miR-622	ENSG00000137210	8m	TargetScan
miR-622	ENSG00000137478	8m	TargetScan
miR-622	ENSG00000137494	8m	TargetScan
miR-622	ENSG00000137714	8m	TargetScan
miR-622	ENSG00000137817	8m	TargetScan
miR-622	ENSG00000137819	8m	TargetScan
miR-622	ENSG00000137959	8m	TargetScan
miR-622	ENSG00000138134	8m	TargetScan
miR-622	ENSG00000138175	8m	TargetScan
miR-622	ENSG00000138411	8m	TargetScan
miR-622	ENSG00000138443	8m	TargetScan
miR-622	ENSG00000138798	8m	TargetScan
miR-622	ENSG00000138944	8m	TargetScan
miR-622	ENSG00000139131	8m	TargetScan
miR-622	ENSG00000139146	8m	TargetScan
miR-622	ENSG00000139428	8m	TargetScan
miR-622	ENSG00000139433	8m	TargetScan
miR-622	ENSG00000140332	8m	TargetScan
miR-622	ENSG00000140367	8m	TargetScan
miR-622	ENSG00000140577	8m	TargetScan
miR-622	ENSG00000140853	8m	TargetScan
miR-622	ENSG00000140931	8m	TargetScan
miR-622	ENSG00000140941	8m	TargetScan
miR-622	ENSG00000140943	8m	TargetScan
miR-622	ENSG00000141013	8m	TargetScan
miR-622	ENSG00000141337	8m	TargetScan
miR-622	ENSG00000141376	8m	TargetScan
miR-622	ENSG00000141380	8m	TargetScan
miR-622	ENSG00000141447	8m	TargetScan

miR-622	ENSG00000141469	8m	TargetScan
miR-622	ENSG00000141506	8m	TargetScan
miR-622	ENSG00000141510	8m	TargetScan
miR-622	ENSG00000141522	8m	TargetScan
miR-622	ENSG00000141527	8m	TargetScan
miR-622	ENSG00000141552	8m	TargetScan
miR-622	ENSG00000141568	8m	TargetScan
miR-622	ENSG00000142408	8m	TargetScan
miR-622	ENSG00000142657	8m	TargetScan
miR-622	ENSG00000143382	8m	TargetScan
miR-622	ENSG00000143393	8m	TargetScan
miR-622	ENSG00000143409	8m	TargetScan
miR-622	ENSG00000143502	8m	TargetScan
miR-622	ENSG00000143603	8m	TargetScan
miR-622	ENSG00000143786	8m	TargetScan
miR-622	ENSG00000143951	8m	TargetScan
miR-622	ENSG00000143995	8m	TargetScan
miR-622	ENSG00000144063	8m	TargetScan
miR-622	ENSG00000144320	8m	TargetScan
miR-622	ENSG00000144381	8m	TargetScan
miR-622	ENSG00000144401	8m	TargetScan
miR-622	ENSG00000144677	8m	TargetScan
miR-622	ENSG00000144741	8m	TargetScan
miR-622	ENSG00000144791	8m	TargetScan
miR-622	ENSG00000144909	8m	TargetScan
miR-622	ENSG00000145242	8m	TargetScan
miR-622	ENSG00000145246	8m	TargetScan
miR-622	ENSG00000145391	8m	TargetScan
miR-622	ENSG00000145416	8m	TargetScan
miR-622	ENSG00000145423	8m	TargetScan
miR-622	ENSG00000145569	8m	TargetScan

miR-622	ENSG00000145685	8m	TargetScan
miR-622	ENSG00000145782	8m	TargetScan
miR-622	ENSG00000145817	8m	TargetScan
miR-622	ENSG00000145919	8m	TargetScan
miR-622	ENSG00000145990	8m	TargetScan
miR-622	ENSG00000146192	8m	TargetScan
miR-622	ENSG00000146592	8m	TargetScan
miR-622	ENSG00000146776	8m	TargetScan
miR-622	ENSG00000146833	8m	TargetScan
miR-622	ENSG00000147421	8m	TargetScan
miR-622	ENSG00000147536	8m	TargetScan
miR-622	ENSG00000147724	8m	TargetScan
miR-622	ENSG00000148053	8m	TargetScan
miR-622	ENSG00000148219	8m	TargetScan
miR-622	ENSG00000148344	8m	TargetScan
miR-622	ENSG00000148600	8m	TargetScan
miR-622	ENSG00000148737	8m	TargetScan
miR-622	ENSG00000148842	8m	TargetScan
miR-622	ENSG00000149257	8m	TargetScan
miR-622	ENSG00000149273	8m	TargetScan
miR-622	ENSG00000149357	8m	TargetScan
miR-622	ENSG00000149527	8m	TargetScan
miR-622	ENSG00000149575	8m	TargetScan
miR-622	ENSG00000149577	8m	TargetScan
miR-622	ENSG00000149651	8m	TargetScan
miR-622	ENSG00000149658	8m	TargetScan
miR-622	ENSG00000149948	8m	TargetScan
miR-622	ENSG00000150977	8m	TargetScan
miR-622	ENSG00000151304	8m	TargetScan
miR-622	ENSG00000151445	8m	TargetScan
miR-622	ENSG00000151466	8m	TargetScan

miR-622	ENSG00000151474	8m	TargetScan
miR-622	ENSG00000151729	8m	TargetScan
miR-622	ENSG00000151923	8m	TargetScan
miR-622	ENSG00000152104	8m	TargetScan
miR-622	ENSG00000152147	8m	TargetScan
miR-622	ENSG00000152601	8m	TargetScan
miR-622	ENSG00000152672	8m	TargetScan
miR-622	ENSG00000153250	8m	TargetScan
miR-622	ENSG00000153339	8m	TargetScan
miR-622	ENSG00000153487	8m	TargetScan
miR-622	ENSG00000153898	8m	TargetScan
miR-622	ENSG00000154237	8m	TargetScan
miR-622	ENSG00000154856	8m	TargetScan
miR-622	ENSG00000155052	8m	TargetScan
miR-622	ENSG00000155926	8m	TargetScan
miR-622	ENSG00000156103	8m	TargetScan
miR-622	ENSG00000156239	8m	TargetScan
miR-622	ENSG00000156414	8m	TargetScan
miR-622	ENSG00000156471	8m	TargetScan
miR-622	ENSG00000156486	8m	TargetScan
miR-622	ENSG00000156671	8m	TargetScan
miR-622	ENSG00000156831	8m	TargetScan
miR-622	ENSG00000157214	8m	TargetScan
miR-622	ENSG00000157500	8m	TargetScan
miR-622	ENSG00000157741	8m	TargetScan
miR-622	ENSG00000158006	8m	TargetScan
miR-622	ENSG00000158163	8m	TargetScan
miR-622	ENSG00000158258	8m	TargetScan
miR-622	ENSG00000158445	8m	TargetScan
miR-622	ENSG00000158470	8m	TargetScan
miR-622	ENSG00000158555	8m	TargetScan

miR-622	ENSG00000158711	8m	TargetScan
miR-622	ENSG00000159256	8m	TargetScan
miR-622	ENSG00000159423	8m	TargetScan
miR-622	ENSG00000160208	8m	TargetScan
miR-622	ENSG00000160214	8m	TargetScan
miR-622	ENSG00000160685	8m	TargetScan
miR-622	ENSG00000160791	8m	TargetScan
miR-622	ENSG00000160886	8m	TargetScan
miR-622	ENSG00000160908	8m	TargetScan
miR-622	ENSG00000161813	8m	TargetScan
miR-622	ENSG00000162076	8m	TargetScan
miR-622	ENSG00000162129	8m	TargetScan
miR-622	ENSG00000162144	8m	TargetScan
miR-622	ENSG00000162419	8m	TargetScan
miR-622	ENSG00000162441	8m	TargetScan
miR-622	ENSG00000162444	8m	TargetScan
miR-622	ENSG00000162599	8m	TargetScan
miR-622	ENSG00000162739	8m	TargetScan
miR-622	ENSG00000162783	8m	TargetScan
miR-622	ENSG00000162804	8m	TargetScan
miR-622	ENSG00000162946	8m	TargetScan
miR-622	ENSG00000163092	8m	TargetScan
miR-622	ENSG00000163104	8m	TargetScan
miR-622	ENSG00000163288	8m	TargetScan
miR-622	ENSG00000163344	8m	TargetScan
miR-622	ENSG00000163412	8m	TargetScan
miR-622	ENSG00000163638	8m	TargetScan
miR-622	ENSG00000163655	8m	TargetScan
miR-622	ENSG00000163684	8m	TargetScan
miR-622	ENSG00000163812	8m	TargetScan
miR-622	ENSG00000163872	8m	TargetScan

miR-622	ENSG00000163877	8m	TargetScan
miR-622	ENSG00000163909	8m	TargetScan
miR-622	ENSG00000163939	8m	TargetScan
miR-622	ENSG00000164022	8m	TargetScan
miR-622	ENSG00000164031	8m	TargetScan
miR-622	ENSG00000164050	8m	TargetScan
miR-622	ENSG00000164070	8m	TargetScan
miR-622	ENSG00000164164	8m	TargetScan
miR-622	ENSG00000164180	8m	TargetScan
miR-622	ENSG00000164220	8m	TargetScan
miR-622	ENSG00000164244	8m	TargetScan
miR-622	ENSG00000164253	8m	TargetScan
miR-622	ENSG00000164292	8m	TargetScan
miR-622	ENSG00000164466	8m	TargetScan
miR-622	ENSG00000164485	8m	TargetScan
miR-622	ENSG00000164574	8m	TargetScan
miR-622	ENSG00000164626	8m	TargetScan
miR-622	ENSG00000164631	8m	TargetScan
miR-622	ENSG00000164684	8m	TargetScan
miR-622	ENSG00000164690	8m	TargetScan
miR-622	ENSG00000164754	8m	TargetScan
miR-622	ENSG00000164938	8m	TargetScan
miR-622	ENSG00000165406	8m	TargetScan
miR-622	ENSG00000165633	8m	TargetScan
miR-622	ENSG00000165816	8m	TargetScan
miR-622	ENSG00000166037	8m	TargetScan
miR-622	ENSG00000166140	8m	TargetScan
miR-622	ENSG00000166233	8m	TargetScan
miR-622	ENSG00000166250	8m	TargetScan
miR-622	ENSG00000166263	8m	TargetScan
miR-622	ENSG00000166411	8m	TargetScan

miR-622	ENSG00000166435	8m	TargetScan
miR-622	ENSG00000166741	8m	TargetScan
miR-622	ENSG00000166747	8m	TargetScan
miR-622	ENSG00000166847	8m	TargetScan
miR-622	ENSG00000166862	8m	TargetScan
miR-622	ENSG00000166900	8m	TargetScan
miR-622	ENSG00000166963	8m	TargetScan
miR-622	ENSG00000167112	8m	TargetScan
miR-622	ENSG00000167196	8m	TargetScan
miR-622	ENSG00000167208	8m	TargetScan
miR-622	ENSG00000167377	8m	TargetScan
miR-622	ENSG00000167528	8m	TargetScan
miR-622	ENSG00000167721	8m	TargetScan
miR-622	ENSG00000167977	8m	TargetScan
miR-622	ENSG00000168118	8m	TargetScan
miR-622	ENSG00000168411	8m	TargetScan
miR-622	ENSG00000168461	8m	TargetScan
miR-622	ENSG00000168631	8m	TargetScan
miR-622	ENSG00000168734	8m	TargetScan
miR-622	ENSG00000168769	8m	TargetScan
miR-622	ENSG00000168795	8m	TargetScan
miR-622	ENSG00000168818	8m	TargetScan
miR-622	ENSG00000168939	8m	TargetScan
miR-622	ENSG00000168939	8m	TargetScan
miR-622	ENSG00000169021	8m	TargetScan
miR-622	ENSG00000169554	8m	TargetScan
miR-622	ENSG00000169814	8m	TargetScan
miR-622	ENSG00000169860	8m	TargetScan
miR-622	ENSG00000169905	8m	TargetScan
miR-622	ENSG00000169908	8m	TargetScan
miR-622	ENSG00000169955	8m	TargetScan

miR-622	ENSG00000169967	8m	TargetScan
miR-622	ENSG00000170017	8m	TargetScan
miR-622	ENSG00000170027	8m	TargetScan
miR-622	ENSG00000170153	8m	TargetScan
miR-622	ENSG00000170242	8m	TargetScan
miR-622	ENSG00000170500	8m	TargetScan
miR-622	ENSG00000170927	8m	TargetScan
miR-622	ENSG00000171451	8m	TargetScan
miR-622	ENSG00000171533	8m	TargetScan
miR-622	ENSG00000171596	8m	TargetScan
miR-622	ENSG00000171862	8m	TargetScan
miR-622	ENSG00000171865	8m	TargetScan
miR-622	ENSG00000172014	8m	TargetScan
miR-622	ENSG00000172575	8m	TargetScan
miR-622	ENSG00000172663	8m	TargetScan
miR-622	ENSG00000172671	8m	TargetScan
miR-622	ENSG00000172819	8m	TargetScan
miR-622	ENSG00000172840	8m	TargetScan
miR-622	ENSG00000172943	8m	TargetScan
miR-622	ENSG00000173275	8m	TargetScan
miR-622	ENSG00000173542	8m	TargetScan
miR-622	ENSG00000173674	8m	TargetScan
miR-622	ENSG00000173698	8m	TargetScan
miR-622	ENSG00000173947	8m	TargetScan
miR-622	ENSG00000174243	8m	TargetScan
miR-622	ENSG00000174485	8m	TargetScan
miR-622	ENSG00000174611	8m	TargetScan
miR-622	ENSG00000174640	8m	TargetScan
miR-622	ENSG00000174749	8m	TargetScan
miR-622	ENSG00000174953	8m	TargetScan
miR-622	ENSG00000175003	8m	TargetScan

miR-622	ENSG00000175104	8m	TargetScan
miR-622	ENSG00000175175	8m	TargetScan
miR-622	ENSG00000175387	8m	TargetScan
miR-622	ENSG00000175455	8m	TargetScan
miR-622	ENSG00000175497	8m	TargetScan
miR-622	ENSG00000175766	8m	TargetScan
miR-622	ENSG00000175854	8m	TargetScan
miR-622	ENSG00000176014	8m	TargetScan
miR-622	ENSG00000176171	8m	TargetScan
miR-622	ENSG00000176410	8m	TargetScan
miR-622	ENSG00000176723	8m	TargetScan
miR-622	ENSG00000177034	8m	TargetScan
miR-622	ENSG00000177511	8m	TargetScan
miR-622	ENSG00000177570	8m	TargetScan
miR-622	ENSG00000178038	8m	TargetScan
miR-622	ENSG00000178104	8m	TargetScan
miR-622	ENSG00000178217	8m	TargetScan
miR-622	ENSG00000178338	8m	TargetScan
miR-622	ENSG00000178385	8m	TargetScan
miR-622	ENSG00000179119	8m	TargetScan
miR-622	ENSG00000179152	8m	TargetScan
miR-622	ENSG00000179314	8m	TargetScan
miR-622	ENSG00000179399	8m	TargetScan
miR-622	ENSG00000179715	8m	TargetScan
miR-622	ENSG00000180011	8m	TargetScan
miR-622	ENSG00000180509	8m	TargetScan
miR-622	ENSG00000180543	8m	TargetScan
miR-622	ENSG00000180628	8m	TargetScan
miR-622	ENSG00000180758	8m	TargetScan
miR-622	ENSG00000180787	8m	TargetScan
miR-622	ENSG00000180822	8m	TargetScan

miR-622	ENSG00000180901	8m	TargetScan
miR-622	ENSG00000181163	8m	TargetScan
miR-622	ENSG00000181544	8m	TargetScan
miR-622	ENSG00000181577	8m	TargetScan
miR-622	ENSG00000181722	8m	TargetScan
miR-622	ENSG00000182218	8m	TargetScan
miR-622	ENSG00000182348	8m	TargetScan
miR-622	ENSG00000182575	8m	TargetScan
miR-622	ENSG00000182919	8m	TargetScan
miR-622	ENSG00000183023	8m	TargetScan
miR-622	ENSG00000183066	8m	TargetScan
miR-622	ENSG00000183098	8m	TargetScan
miR-622	ENSG00000183137	8m	TargetScan
miR-622	ENSG00000183148	8m	TargetScan
miR-622	ENSG00000183154	8m	TargetScan
miR-622	ENSG00000183250	8m	TargetScan
miR-622	ENSG00000183386	8m	TargetScan
miR-622	ENSG00000183423	8m	TargetScan
miR-622	ENSG00000183476	8m	TargetScan
miR-622	ENSG00000183784	8m	TargetScan
miR-622	ENSG00000184113	8m	TargetScan
miR-622	ENSG00000184347	8m	TargetScan
miR-622	ENSG00000184517	8m	TargetScan
miR-622	ENSG00000184571	8m	TargetScan
miR-622	ENSG00000184678	8m	TargetScan
miR-622	ENSG00000185163	8m	TargetScan
miR-622	ENSG00000185267	8m	TargetScan
miR-622	ENSG00000185278	8m	TargetScan
miR-622	ENSG00000185518	8m	TargetScan
miR-622	ENSG00000185681	8m	TargetScan
miR-622	ENSG00000186150	8m	TargetScan

miR-622	ENSG00000186687	8m	TargetScan
miR-622	ENSG00000186895	8m	TargetScan
miR-622	ENSG00000186908	8m	TargetScan
miR-622	ENSG00000187147	8m	TargetScan
miR-622	ENSG00000187510	8m	TargetScan
miR-622	ENSG00000187607	8m	TargetScan
miR-622	ENSG00000187664	8m	TargetScan
miR-622	ENSG00000187753	8m	TargetScan
miR-622	ENSG00000187792	8m	TargetScan
miR-622	ENSG00000187800	8m	TargetScan
miR-622	ENSG00000188026	8m	TargetScan
miR-622	ENSG00000188152	8m	TargetScan
miR-622	ENSG00000188176	8m	TargetScan
miR-622	ENSG00000188215	8m	TargetScan
miR-622	ENSG00000188266	8m	TargetScan
miR-622	ENSG00000188277	8m	TargetScan
miR-622	ENSG00000188511	8m	TargetScan
miR-622	ENSG00000188596	8m	TargetScan
miR-622	ENSG00000188610	8m	TargetScan
miR-622	ENSG00000188612	8m	TargetScan
miR-622	ENSG00000188921	8m	TargetScan
miR-622	ENSG00000189377	8m	TargetScan
miR-622	ENSG00000196167	8m	TargetScan
miR-622	ENSG00000196208	8m	TargetScan
miR-622	ENSG00000196323	8m	TargetScan
miR-622	ENSG00000196376	8m	TargetScan
miR-622	ENSG00000196505	8m	TargetScan
miR-622	ENSG00000196550	8m	TargetScan
miR-622	ENSG00000196569	8m	TargetScan
miR-622	ENSG00000196812	8m	TargetScan
miR-622	ENSG00000197081	8m	TargetScan

miR-622	ENSG00000197406	8m	TargetScan
miR-622	ENSG00000197557	8m	TargetScan
miR-622	ENSG00000197977	8m	TargetScan
miR-622	ENSG00000198040	8m	TargetScan
miR-622	ENSG00000198105	8m	TargetScan
miR-622	ENSG00000198160	8m	TargetScan
miR-622	ENSG00000198198	8m	TargetScan
miR-622	ENSG00000198612	8m	TargetScan
miR-622	ENSG00000198700	8m	TargetScan
miR-622	ENSG00000198720	8m	TargetScan
miR-622	ENSG00000198743	8m	TargetScan
miR-622	ENSG00000198799	8m	TargetScan
miR-622	ENSG00000198815	8m	TargetScan
miR-622	ENSG00000198821	8m	TargetScan
miR-622	ENSG00000198890	8m	TargetScan
miR-622	ENSG00000198914	8m	TargetScan
miR-622	ENSG00000203667	8m	TargetScan
miR-622	ENSG00000203867	8m	TargetScan
miR-622	ENSG00000204131	8m	TargetScan
miR-622	ENSG00000204174	8m	TargetScan
miR-622	ENSG00000204231	8m	TargetScan
miR-622	ENSG00000204381	8m	TargetScan
miR-622	ENSG00000204514	8m	TargetScan
miR-622	ENSG00000204520	8m	TargetScan
miR-622	ENSG00000204814	8m	TargetScan
miR-622	ENSG00000205060	8m	TargetScan
miR-622	ENSG00000205268	8m	TargetScan
miR-622	ENSG00000205334	8m	TargetScan
miR-622	ENSG00000205356	8m	TargetScan
miR-622	ENSG00000205765	8m	TargetScan
miR-622	ENSG00000206344	8m	TargetScan

miR-622	ENSG00000206579	8m	TargetScan
miR-622	ENSG00000212122	8m	TargetScan
miR-622	ENSG00000213380	8m	TargetScan
miR-622	ENSG00000213614	8m	TargetScan
miR-622	ENSG00000213694	8m	TargetScan
miR-622	ENSG00000213699	8m	TargetScan
miR-622	ENSG00000213741	8m	TargetScan
miR-622	ENSG00000214013	8m	TargetScan
miR-622	ENSG00000214097	8m	TargetScan
miR-622	ENSG00000214193	8m	TargetScan
miR-622	ENSG00000214367	8m	TargetScan
miR-622	ENSG00000214491	8m	TargetScan
miR-622	ENSG00000214655	8m	TargetScan
miR-622	ENSG00000215568	8m	TargetScan
miR-622	ENSG00000215784	8m	TargetScan
miR-622	ENSG00000221823	8m	TargetScan
miR-622	ENSG00000221866	8m	TargetScan
miR-622	ENSG00000221990	8m	TargetScan
miR-622	ENSG00000224916	8m	TargetScan
miR-622	ENSG00000233493	8m	TargetScan
miR-622	ENSG00000234906	8m	TargetScan
miR-622	ENSG00000235568	8m	TargetScan
miR-622	ENSG00000240021	8m	TargetScan
miR-622	ENSG00000240053	8m	TargetScan
miR-622	ENSG00000240682	8m	TargetScan
miR-622	ENSG00000243710	8m	TargetScan
miR-622	ENSG00000244005	8m	TargetScan
miR-622	ENSG00000244274	8m	TargetScan
miR-622	ENSG00000248485	8m	TargetScan
miR-622	ENSG00000249459	8m	TargetScan
miR-622	ENSG00000254726	8m	TargetScan

miR-622	ENSG00000255874	8m	TargetScan
miR-622	ENSG00000256043	8m	TargetScan
miR-622	ENSG00000256162	8m	TargetScan
miR-622	ENSG00000256229	8m	TargetScan
miR-622	ENSG00000256574	8m	TargetScan
miR-622	ENSG00000257184	8m	TargetScan
miR-622	ENSG00000257315	8m	TargetScan
miR-622	ENSG00000258429	8m	TargetScan
miR-622	ENSG00000258729	8m	TargetScan
miR-622	ENSG00000259030	8m	TargetScan
miR-622	ENSG00000261221	8m	TargetScan
miR-622	ENSG00000263002	8m	TargetScan
miR-622	ENSG00000263020	8m	TargetScan
miR-622	ENSG00000263513	8m	TargetScan
miR-622	ENSG00000268926	8m	TargetScan
miR-622	ENSG00000269155	8m	TargetScan
miR-622	ENSG00000269846	8m	TargetScan
miR-622	ENSG00000270757	8m	TargetScan
miR-622	ENSG00000272617	8m	TargetScan
miR-622	ENSG00000285347	8m	TargetScan
miR-7161-3p	ENSG00000001617	8m	TargetScan
miR-7161-3p	ENSG00000002746	8m	TargetScan
miR-7161-3p	ENSG00000003400	8m	TargetScan
miR-7161-3p	ENSG00000003402	8m	TargetScan
miR-7161-3p	ENSG00000004478	8m	TargetScan
miR-7161-3p	ENSG00000004700	8m	TargetScan
miR-7161-3p	ENSG00000005981	8m	TargetScan
miR-7161-3p	ENSG00000006116	8m	TargetScan
miR-7161-3p	ENSG00000007174	8m	TargetScan
miR-7161-3p	ENSG00000007237	8m	TargetScan
miR-7161-3p	ENSG00000008311	8m	TargetScan

miR-7161-3p	ENSG00000009694	8m	TargetScan
miR-7161-3p	ENSG00000011275	8m	TargetScan
miR-7161-3p	ENSG00000019995	8m	TargetScan
miR-7161-3p	ENSG00000031081	8m	TargetScan
miR-7161-3p	ENSG00000033122	8m	TargetScan
miR-7161-3p	ENSG00000036549	8m	TargetScan
miR-7161-3p	ENSG00000039139	8m	TargetScan
miR-7161-3p	ENSG00000046653	8m	TargetScan
miR-7161-3p	ENSG00000047457	8m	TargetScan
miR-7161-3p	ENSG00000048471	8m	TargetScan
miR-7161-3p	ENSG00000051382	8m	TargetScan
miR-7161-3p	ENSG00000056291	8m	TargetScan
miR-7161-3p	ENSG00000057657	8m	TargetScan
miR-7161-3p	ENSG00000057935	8m	TargetScan
miR-7161-3p	ENSG00000062598	8m	TargetScan
miR-7161-3p	ENSG00000064607	8m	TargetScan
miR-7161-3p	ENSG00000065325	8m	TargetScan
miR-7161-3p	ENSG00000065413	8m	TargetScan
miR-7161-3p	ENSG00000065457	8m	TargetScan
miR-7161-3p	ENSG00000065665	8m	TargetScan
miR-7161-3p	ENSG00000066084	8m	TargetScan
miR-7161-3p	ENSG00000066185	8m	TargetScan
miR-7161-3p	ENSG00000066813	8m	TargetScan
miR-7161-3p	ENSG00000067082	8m	TargetScan
miR-7161-3p	ENSG00000067533	8m	TargetScan
miR-7161-3p	ENSG00000067900	8m	TargetScan
miR-7161-3p	ENSG00000068024	8m	TargetScan
miR-7161-3p	ENSG00000069667	8m	TargetScan
miR-7161-3p	ENSG00000069812	8m	TargetScan
miR-7161-3p	ENSG00000070159	8m	TargetScan
miR-7161-3p	ENSG00000070214	8m	TargetScan

miR-7161-3p	ENSG00000070756	8m	TargetScan
miR-7161-3p	ENSG00000072401	8m	TargetScan
miR-7161-3p	ENSG00000073849	8m	TargetScan
miR-7161-3p	ENSG00000074695	8m	TargetScan
miR-7161-3p	ENSG00000074706	8m	TargetScan
miR-7161-3p	ENSG00000075142	8m	TargetScan
miR-7161-3p	ENSG00000075426	8m	TargetScan
miR-7161-3p	ENSG00000075568	8m	TargetScan
miR-7161-3p	ENSG00000077264	8m	TargetScan
miR-7161-3p	ENSG00000077498	8m	TargetScan
miR-7161-3p	ENSG00000077684	8m	TargetScan
miR-7161-3p	ENSG00000078124	8m	TargetScan
miR-7161-3p	ENSG00000078237	8m	TargetScan
miR-7161-3p	ENSG00000080298	8m	TargetScan
miR-7161-3p	ENSG00000080345	8m	TargetScan
miR-7161-3p	ENSG00000080561	8m	TargetScan
miR-7161-3p	ENSG00000080603	8m	TargetScan
miR-7161-3p	ENSG00000080822	8m	TargetScan
miR-7161-3p	ENSG00000082805	8m	TargetScan
miR-7161-3p	ENSG00000085563	8m	TargetScan
miR-7161-3p	ENSG00000086102	8m	TargetScan
miR-7161-3p	ENSG00000086619	8m	TargetScan
miR-7161-3p	ENSG00000087074	8m	TargetScan
miR-7161-3p	ENSG00000088356	8m	TargetScan
miR-7161-3p	ENSG00000089041	8m	TargetScan
miR-7161-3p	ENSG00000089916	8m	TargetScan
miR-7161-3p	ENSG00000090054	8m	TargetScan
miR-7161-3p	ENSG00000090060	8m	TargetScan
miR-7161-3p	ENSG00000090376	8m	TargetScan
miR-7161-3p	ENSG00000091039	8m	TargetScan
miR-7161-3p	ENSG00000091592	8m	TargetScan

miR-7161-3p	ENSG00000091831	8m	TargetScan
miR-7161-3p	ENSG00000092531	8m	TargetScan
miR-7161-3p	ENSG00000092871	8m	TargetScan
miR-7161-3p	ENSG00000096060	8m	TargetScan
miR-7161-3p	ENSG00000096401	8m	TargetScan
miR-7161-3p	ENSG00000097033	8m	TargetScan
miR-7161-3p	ENSG00000099337	8m	TargetScan
miR-7161-3p	ENSG00000099810	8m	TargetScan
miR-7161-3p	ENSG00000099968	8m	TargetScan
miR-7161-3p	ENSG00000100167	8m	TargetScan
miR-7161-3p	ENSG00000100201	8m	TargetScan
miR-7161-3p	ENSG00000100344	8m	TargetScan
miR-7161-3p	ENSG00000100345	8m	TargetScan
miR-7161-3p	ENSG00000100523	8m	TargetScan
miR-7161-3p	ENSG00000100568	8m	TargetScan
miR-7161-3p	ENSG00000100601	8m	TargetScan
miR-7161-3p	ENSG00000100678	8m	TargetScan
miR-7161-3p	ENSG00000100852	8m	TargetScan
miR-7161-3p	ENSG00000100934	8m	TargetScan
miR-7161-3p	ENSG00000101019	8m	TargetScan
miR-7161-3p	ENSG00000101144	8m	TargetScan
miR-7161-3p	ENSG00000101343	8m	TargetScan
miR-7161-3p	ENSG00000101751	8m	TargetScan
miR-7161-3p	ENSG00000101752	8m	TargetScan
miR-7161-3p	ENSG00000101842	8m	TargetScan
miR-7161-3p	ENSG00000101945	8m	TargetScan
miR-7161-3p	ENSG00000102172	8m	TargetScan
miR-7161-3p	ENSG00000102755	8m	TargetScan
miR-7161-3p	ENSG00000104290	8m	TargetScan
miR-7161-3p	ENSG00000104447	8m	TargetScan
miR-7161-3p	ENSG00000104626	8m	TargetScan

miR-7161-3p	ENSG00000104756	8m	TargetScan
miR-7161-3p	ENSG00000105392	8m	TargetScan
miR-7161-3p	ENSG00000105656	8m	TargetScan
miR-7161-3p	ENSG00000105819	8m	TargetScan
miR-7161-3p	ENSG00000105835	8m	TargetScan
miR-7161-3p	ENSG00000105968	8m	TargetScan
miR-7161-3p	ENSG00000106331	8m	TargetScan
miR-7161-3p	ENSG00000106609	8m	TargetScan
miR-7161-3p	ENSG00000106689	8m	TargetScan
miR-7161-3p	ENSG00000106733	8m	TargetScan
miR-7161-3p	ENSG00000108387	8m	TargetScan
miR-7161-3p	ENSG00000108395	8m	TargetScan
miR-7161-3p	ENSG00000108669	8m	TargetScan
miR-7161-3p	ENSG00000109046	8m	TargetScan
miR-7161-3p	ENSG00000109332	8m	TargetScan
miR-7161-3p	ENSG00000109381	8m	TargetScan
miR-7161-3p	ENSG00000109667	8m	TargetScan
miR-7161-3p	ENSG00000109787	8m	TargetScan
miR-7161-3p	ENSG00000110395	8m	TargetScan
miR-7161-3p	ENSG00000110427	8m	TargetScan
miR-7161-3p	ENSG00000110723	8m	TargetScan
miR-7161-3p	ENSG00000111012	8m	TargetScan
miR-7161-3p	ENSG00000111142	8m	TargetScan
miR-7161-3p	ENSG00000111596	8m	TargetScan
miR-7161-3p	ENSG00000111727	8m	TargetScan
miR-7161-3p	ENSG00000111732	8m	TargetScan
miR-7161-3p	ENSG00000111850	8m	TargetScan
miR-7161-3p	ENSG00000112246	8m	TargetScan
miR-7161-3p	ENSG00000112249	8m	TargetScan
miR-7161-3p	ENSG00000112357	8m	TargetScan
miR-7161-3p	ENSG00000112796	8m	TargetScan

miR-7161-3p	ENSG00000112902	8m	TargetScan
miR-7161-3p	ENSG00000112964	8m	TargetScan
miR-7161-3p	ENSG00000113361	8m	TargetScan
miR-7161-3p	ENSG00000113369	8m	TargetScan
miR-7161-3p	ENSG00000113384	8m	TargetScan
miR-7161-3p	ENSG00000114030	8m	TargetScan
miR-7161-3p	ENSG00000114346	8m	TargetScan
miR-7161-3p	ENSG00000114374	8m	TargetScan
miR-7161-3p	ENSG00000114656	8m	TargetScan
miR-7161-3p	ENSG00000114686	8m	TargetScan
miR-7161-3p	ENSG00000114933	8m	TargetScan
miR-7161-3p	ENSG00000114948	8m	TargetScan
miR-7161-3p	ENSG00000115109	8m	TargetScan
miR-7161-3p	ENSG00000115252	8m	TargetScan
miR-7161-3p	ENSG00000115355	8m	TargetScan
miR-7161-3p	ENSG00000115750	8m	TargetScan
miR-7161-3p	ENSG00000115827	8m	TargetScan
miR-7161-3p	ENSG00000116273	8m	TargetScan
miR-7161-3p	ENSG00000116641	8m	TargetScan
miR-7161-3p	ENSG00000116747	8m	TargetScan
miR-7161-3p	ENSG00000116750	8m	TargetScan
miR-7161-3p	ENSG00000117020	8m	TargetScan
miR-7161-3p	ENSG00000117533	8m	TargetScan
miR-7161-3p	ENSG00000118432	8m	TargetScan
miR-7161-3p	ENSG00000118508	8m	TargetScan
miR-7161-3p	ENSG00000119509	8m	TargetScan
miR-7161-3p	ENSG00000119547	8m	TargetScan
miR-7161-3p	ENSG00000119900	8m	TargetScan
miR-7161-3p	ENSG00000119953	8m	TargetScan
miR-7161-3p	ENSG00000119969	8m	TargetScan
miR-7161-3p	ENSG00000121152	8m	TargetScan

miR-7161-3p	ENSG00000121281	8m	TargetScan
miR-7161-3p	ENSG00000121413	8m	TargetScan
miR-7161-3p	ENSG00000121579	8m	TargetScan
miR-7161-3p	ENSG00000122378	8m	TargetScan
miR-7161-3p	ENSG00000122591	8m	TargetScan
miR-7161-3p	ENSG00000122778	8m	TargetScan
miR-7161-3p	ENSG00000123104	8m	TargetScan
miR-7161-3p	ENSG00000123892	8m	TargetScan
miR-7161-3p	ENSG00000123983	8m	TargetScan
miR-7161-3p	ENSG00000124802	8m	TargetScan
miR-7161-3p	ENSG00000125520	8m	TargetScan
miR-7161-3p	ENSG00000125703	8m	TargetScan
miR-7161-3p	ENSG00000125812	8m	TargetScan
miR-7161-3p	ENSG00000126070	8m	TargetScan
miR-7161-3p	ENSG00000126773	8m	TargetScan
miR-7161-3p	ENSG00000127329	8m	TargetScan
miR-7161-3p	ENSG00000128059	8m	TargetScan
miR-7161-3p	ENSG00000128510	8m	TargetScan
miR-7161-3p	ENSG00000129292	8m	TargetScan
miR-7161-3p	ENSG00000129559	8m	TargetScan
miR-7161-3p	ENSG00000130348	8m	TargetScan
miR-7161-3p	ENSG00000130827	8m	TargetScan
miR-7161-3p	ENSG00000130844	8m	TargetScan
miR-7161-3p	ENSG00000131115	8m	TargetScan
miR-7161-3p	ENSG00000131238	8m	TargetScan
miR-7161-3p	ENSG00000131323	8m	TargetScan
miR-7161-3p	ENSG00000131375	8m	TargetScan
miR-7161-3p	ENSG00000131381	8m	TargetScan
miR-7161-3p	ENSG00000131711	8m	TargetScan
miR-7161-3p	ENSG00000133019	8m	TargetScan
miR-7161-3p	ENSG00000133083	8m	TargetScan

miR-7161-3p	ENSG00000133687	8m	TargetScan
miR-7161-3p	ENSG00000133731	8m	TargetScan
miR-7161-3p	ENSG00000134253	8m	TargetScan
miR-7161-3p	ENSG00000134333	8m	TargetScan
miR-7161-3p	ENSG00000134352	8m	TargetScan
miR-7161-3p	ENSG00000134531	8m	TargetScan
miR-7161-3p	ENSG00000134940	8m	TargetScan
miR-7161-3p	ENSG00000135049	8m	TargetScan
miR-7161-3p	ENSG00000135185	8m	TargetScan
miR-7161-3p	ENSG00000135341	8m	TargetScan
miR-7161-3p	ENSG00000135414	8m	TargetScan
miR-7161-3p	ENSG00000135547	8m	TargetScan
miR-7161-3p	ENSG00000135778	8m	TargetScan
miR-7161-3p	ENSG00000135930	8m	TargetScan
miR-7161-3p	ENSG00000136371	8m	TargetScan
miR-7161-3p	ENSG00000137055	8m	TargetScan
miR-7161-3p	ENSG00000137210	8m	TargetScan
miR-7161-3p	ENSG00000137414	8m	TargetScan
miR-7161-3p	ENSG00000137460	8m	TargetScan
miR-7161-3p	ENSG00000137502	8m	TargetScan
miR-7161-3p	ENSG00000137573	8m	TargetScan
miR-7161-3p	ENSG00000137642	8m	TargetScan
miR-7161-3p	ENSG00000137941	8m	TargetScan
miR-7161-3p	ENSG00000138109	8m	TargetScan
miR-7161-3p	ENSG00000138279	8m	TargetScan
miR-7161-3p	ENSG00000138347	8m	TargetScan
miR-7161-3p	ENSG00000138395	8m	TargetScan
miR-7161-3p	ENSG00000138755	8m	TargetScan
miR-7161-3p	ENSG00000138802	8m	TargetScan
miR-7161-3p	ENSG00000139133	8m	TargetScan
miR-7161-3p	ENSG00000139291	8m	TargetScan

miR-7161-3p	ENSG00000139668	8m	TargetScan
miR-7161-3p	ENSG00000139734	8m	TargetScan
miR-7161-3p	ENSG00000139746	8m	TargetScan
miR-7161-3p	ENSG00000139874	8m	TargetScan
miR-7161-3p	ENSG00000140691	8m	TargetScan
miR-7161-3p	ENSG00000140807	8m	TargetScan
miR-7161-3p	ENSG00000140853	8m	TargetScan
miR-7161-3p	ENSG00000141161	8m	TargetScan
miR-7161-3p	ENSG00000141179	8m	TargetScan
miR-7161-3p	ENSG00000141524	8m	TargetScan
miR-7161-3p	ENSG00000141570	8m	TargetScan
miR-7161-3p	ENSG00000143217	8m	TargetScan
miR-7161-3p	ENSG00000143248	8m	TargetScan
miR-7161-3p	ENSG00000143252	8m	TargetScan
miR-7161-3p	ENSG00000143337	8m	TargetScan
miR-7161-3p	ENSG00000143498	8m	TargetScan
miR-7161-3p	ENSG00000143603	8m	TargetScan
miR-7161-3p	ENSG00000143842	8m	TargetScan
miR-7161-3p	ENSG00000143970	8m	TargetScan
miR-7161-3p	ENSG00000144283	8m	TargetScan
miR-7161-3p	ENSG00000144407	8m	TargetScan
miR-7161-3p	ENSG00000144481	8m	TargetScan
miR-7161-3p	ENSG00000144824	8m	TargetScan
miR-7161-3p	ENSG00000144908	8m	TargetScan
miR-7161-3p	ENSG00000145335	8m	TargetScan
miR-7161-3p	ENSG00000145384	8m	TargetScan
miR-7161-3p	ENSG00000145725	8m	TargetScan
miR-7161-3p	ENSG00000145734	8m	TargetScan
miR-7161-3p	ENSG00000145743	8m	TargetScan
miR-7161-3p	ENSG00000145781	8m	TargetScan
miR-7161-3p	ENSG00000145826	8m	TargetScan

miR-7161-3p	ENSG00000145832	8m	TargetScan
miR-7161-3p	ENSG00000146267	8m	TargetScan
miR-7161-3p	ENSG00000146278	8m	TargetScan
miR-7161-3p	ENSG00000146285	8m	TargetScan
miR-7161-3p	ENSG00000146414	8m	TargetScan
miR-7161-3p	ENSG00000146463	8m	TargetScan
miR-7161-3p	ENSG00000146674	8m	TargetScan
miR-7161-3p	ENSG00000147535	8m	TargetScan
miR-7161-3p	ENSG00000148411	8m	TargetScan
miR-7161-3p	ENSG00000148429	8m	TargetScan
miR-7161-3p	ENSG00000148600	8m	TargetScan
miR-7161-3p	ENSG00000148672	8m	TargetScan
miR-7161-3p	ENSG00000149124	8m	TargetScan
miR-7161-3p	ENSG00000149218	8m	TargetScan
miR-7161-3p	ENSG00000150394	8m	TargetScan
miR-7161-3p	ENSG00000150471	8m	TargetScan
miR-7161-3p	ENSG00000150477	8m	TargetScan
miR-7161-3p	ENSG00000150938	8m	TargetScan
miR-7161-3p	ENSG00000151135	8m	TargetScan
miR-7161-3p	ENSG00000151422	8m	TargetScan
miR-7161-3p	ENSG00000151491	8m	TargetScan
miR-7161-3p	ENSG00000151892	8m	TargetScan
miR-7161-3p	ENSG00000152192	8m	TargetScan
miR-7161-3p	ENSG00000152207	8m	TargetScan
miR-7161-3p	ENSG00000152380	8m	TargetScan
miR-7161-3p	ENSG00000152601	8m	TargetScan
miR-7161-3p	ENSG00000152749	8m	TargetScan
miR-7161-3p	ENSG00000152782	8m	TargetScan
miR-7161-3p	ENSG00000153214	8m	TargetScan
miR-7161-3p	ENSG00000153790	8m	TargetScan
miR-7161-3p	ENSG00000153827	8m	TargetScan

miR-7161-3p	ENSG00000154222	8m	TargetScan
miR-7161-3p	ENSG00000154319	8m	TargetScan
miR-7161-3p	ENSG00000154710	8m	TargetScan
miR-7161-3p	ENSG00000154845	8m	TargetScan
miR-7161-3p	ENSG00000155016	8m	TargetScan
miR-7161-3p	ENSG00000155052	8m	TargetScan
miR-7161-3p	ENSG00000155097	8m	TargetScan
miR-7161-3p	ENSG00000155229	8m	TargetScan
miR-7161-3p	ENSG00000155926	8m	TargetScan
miR-7161-3p	ENSG00000155966	8m	TargetScan
miR-7161-3p	ENSG00000156162	8m	TargetScan
miR-7161-3p	ENSG00000156711	8m	TargetScan
miR-7161-3p	ENSG00000157036	8m	TargetScan
miR-7161-3p	ENSG00000157131	8m	TargetScan
miR-7161-3p	ENSG00000157540	8m	TargetScan
miR-7161-3p	ENSG00000158163	8m	TargetScan
miR-7161-3p	ENSG00000158258	8m	TargetScan
miR-7161-3p	ENSG00000160049	8m	TargetScan
miR-7161-3p	ENSG00000160200	8m	TargetScan
miR-7161-3p	ENSG00000160551	8m	TargetScan
miR-7161-3p	ENSG00000161405	8m	TargetScan
miR-7161-3p	ENSG00000161911	8m	TargetScan
miR-7161-3p	ENSG00000162409	8m	TargetScan
miR-7161-3p	ENSG00000162545	8m	TargetScan
miR-7161-3p	ENSG00000162614	8m	TargetScan
miR-7161-3p	ENSG00000162616	8m	TargetScan
miR-7161-3p	ENSG00000162636	8m	TargetScan
miR-7161-3p	ENSG00000162645	8m	TargetScan
miR-7161-3p	ENSG00000162733	8m	TargetScan
miR-7161-3p	ENSG00000162849	8m	TargetScan
miR-7161-3p	ENSG00000163125	8m	TargetScan

miR-7161-3p	ENSG00000163235	8m	TargetScan
miR-7161-3p	ENSG00000163281	8m	TargetScan
miR-7161-3p	ENSG00000163297	8m	TargetScan
miR-7161-3p	ENSG00000163507	8m	TargetScan
miR-7161-3p	ENSG00000163605	8m	TargetScan
miR-7161-3p	ENSG00000163608	8m	TargetScan
miR-7161-3p	ENSG00000163697	8m	TargetScan
miR-7161-3p	ENSG00000163710	8m	TargetScan
miR-7161-3p	ENSG00000163904	8m	TargetScan
miR-7161-3p	ENSG00000163950	8m	TargetScan
miR-7161-3p	ENSG00000164048	8m	TargetScan
miR-7161-3p	ENSG00000164061	8m	TargetScan
miR-7161-3p	ENSG00000164113	8m	TargetScan
miR-7161-3p	ENSG00000164299	8m	TargetScan
miR-7161-3p	ENSG00000164309	8m	TargetScan
miR-7161-3p	ENSG00000164430	8m	TargetScan
miR-7161-3p	ENSG00000164484	8m	TargetScan
miR-7161-3p	ENSG00000164651	8m	TargetScan
miR-7161-3p	ENSG00000164663	8m	TargetScan
miR-7161-3p	ENSG00000164736	8m	TargetScan
miR-7161-3p	ENSG00000164879	8m	TargetScan
miR-7161-3p	ENSG00000165084	8m	TargetScan
miR-7161-3p	ENSG00000165120	8m	TargetScan
miR-7161-3p	ENSG00000165194	8m	TargetScan
miR-7161-3p	ENSG00000165195	8m	TargetScan
miR-7161-3p	ENSG00000165240	8m	TargetScan
miR-7161-3p	ENSG00000165416	8m	TargetScan
miR-7161-3p	ENSG00000165434	8m	TargetScan
miR-7161-3p	ENSG00000165609	8m	TargetScan
miR-7161-3p	ENSG00000165633	8m	TargetScan
miR-7161-3p	ENSG00000165688	8m	TargetScan

miR-7161-3p	ENSG00000165757	8m	TargetScan
miR-7161-3p	ENSG00000165841	8m	TargetScan
miR-7161-3p	ENSG00000166206	8m	TargetScan
miR-7161-3p	ENSG00000166265	8m	TargetScan
miR-7161-3p	ENSG00000166272	8m	TargetScan
miR-7161-3p	ENSG00000166441	8m	TargetScan
miR-7161-3p	ENSG00000166569	8m	TargetScan
miR-7161-3p	ENSG00000166664	8m	TargetScan
miR-7161-3p	ENSG00000166797	8m	TargetScan
miR-7161-3p	ENSG00000166848	8m	TargetScan
miR-7161-3p	ENSG00000166928	8m	TargetScan
miR-7161-3p	ENSG00000167637	8m	TargetScan
miR-7161-3p	ENSG00000167981	8m	TargetScan
miR-7161-3p	ENSG00000168118	8m	TargetScan
miR-7161-3p	ENSG00000168564	8m	TargetScan
miR-7161-3p	ENSG00000168566	8m	TargetScan
miR-7161-3p	ENSG00000168743	8m	TargetScan
miR-7161-3p	ENSG00000168906	8m	TargetScan
miR-7161-3p	ENSG00000169306	8m	TargetScan
miR-7161-3p	ENSG00000169856	8m	TargetScan
miR-7161-3p	ENSG00000169905	8m	TargetScan
miR-7161-3p	ENSG00000169914	8m	TargetScan
miR-7161-3p	ENSG00000169946	8m	TargetScan
miR-7161-3p	ENSG00000169955	8m	TargetScan
miR-7161-3p	ENSG00000170419	8m	TargetScan
miR-7161-3p	ENSG00000170677	8m	TargetScan
miR-7161-3p	ENSG00000171004	8m	TargetScan
miR-7161-3p	ENSG00000171109	8m	TargetScan
miR-7161-3p	ENSG00000171320	8m	TargetScan
miR-7161-3p	ENSG00000171435	8m	TargetScan
miR-7161-3p	ENSG00000171530	8m	TargetScan

miR-7161-3p	ENSG00000171634	8m	TargetScan
miR-7161-3p	ENSG00000171649	8m	TargetScan
miR-7161-3p	ENSG00000171791	8m	TargetScan
miR-7161-3p	ENSG00000171817	8m	TargetScan
miR-7161-3p	ENSG00000171928	8m	TargetScan
miR-7161-3p	ENSG00000172380	8m	TargetScan
miR-7161-3p	ENSG00000173068	8m	TargetScan
miR-7161-3p	ENSG00000173349	8m	TargetScan
miR-7161-3p	ENSG00000173641	8m	TargetScan
miR-7161-3p	ENSG00000173681	8m	TargetScan
miR-7161-3p	ENSG00000173786	8m	TargetScan
miR-7161-3p	ENSG00000174749	8m	TargetScan
miR-7161-3p	ENSG00000174792	8m	TargetScan
miR-7161-3p	ENSG00000174943	8m	TargetScan
miR-7161-3p	ENSG00000175066	8m	TargetScan
miR-7161-3p	ENSG00000175097	8m	TargetScan
miR-7161-3p	ENSG00000175344	8m	TargetScan
miR-7161-3p	ENSG00000175387	8m	TargetScan
miR-7161-3p	ENSG00000175395	8m	TargetScan
miR-7161-3p	ENSG00000175893	8m	TargetScan
miR-7161-3p	ENSG00000176371	8m	TargetScan
miR-7161-3p	ENSG00000176595	8m	TargetScan
miR-7161-3p	ENSG00000176887	8m	TargetScan
miR-7161-3p	ENSG00000176915	8m	TargetScan
miR-7161-3p	ENSG00000176953	8m	TargetScan
miR-7161-3p	ENSG00000177034	8m	TargetScan
miR-7161-3p	ENSG00000177181	8m	TargetScan
miR-7161-3p	ENSG00000177614	8m	TargetScan
miR-7161-3p	ENSG00000177689	8m	TargetScan
miR-7161-3p	ENSG00000177707	8m	TargetScan
miR-7161-3p	ENSG00000177733	8m	TargetScan

miR-7161-3p	ENSG00000178033	8m	TargetScan
miR-7161-3p	ENSG00000178171	8m	TargetScan
miR-7161-3p	ENSG00000178201	8m	TargetScan
miR-7161-3p	ENSG00000178338	8m	TargetScan
miR-7161-3p	ENSG00000178662	8m	TargetScan
miR-7161-3p	ENSG00000178752	8m	TargetScan
miR-7161-3p	ENSG00000179133	8m	TargetScan
miR-7161-3p	ENSG00000179520	8m	TargetScan
miR-7161-3p	ENSG00000179813	8m	TargetScan
miR-7161-3p	ENSG00000179933	8m	TargetScan
miR-7161-3p	ENSG00000180008	8m	TargetScan
miR-7161-3p	ENSG00000180525	8m	TargetScan
miR-7161-3p	ENSG00000180884	8m	TargetScan
miR-7161-3p	ENSG00000180914	8m	TargetScan
miR-7161-3p	ENSG00000181518	8m	TargetScan
miR-7161-3p	ENSG00000181982	8m	TargetScan
miR-7161-3p	ENSG00000182261	8m	TargetScan
miR-7161-3p	ENSG00000182287	8m	TargetScan
miR-7161-3p	ENSG00000182348	8m	TargetScan
miR-7161-3p	ENSG00000182359	8m	TargetScan
miR-7161-3p	ENSG00000182890	8m	TargetScan
miR-7161-3p	ENSG00000183114	8m	TargetScan
miR-7161-3p	ENSG00000183695	8m	TargetScan
miR-7161-3p	ENSG00000183742	8m	TargetScan
miR-7161-3p	ENSG00000183747	8m	TargetScan
miR-7161-3p	ENSG00000183762	8m	TargetScan
miR-7161-3p	ENSG00000184378	8m	TargetScan
miR-7161-3p	ENSG00000184937	8m	TargetScan
miR-7161-3p	ENSG00000185565	8m	TargetScan
miR-7161-3p	ENSG00000186103	8m	TargetScan
miR-7161-3p	ENSG00000186265	8m	TargetScan

miR-7161-3p	ENSG00000186479	8m	TargetScan
miR-7161-3p	ENSG00000186838	8m	TargetScan
miR-7161-3p	ENSG00000187134	8m	TargetScan
miR-7161-3p	ENSG00000187726	8m	TargetScan
miR-7161-3p	ENSG00000187792	8m	TargetScan
miR-7161-3p	ENSG00000187866	8m	TargetScan
miR-7161-3p	ENSG00000188056	8m	TargetScan
miR-7161-3p	ENSG00000188089	8m	TargetScan
miR-7161-3p	ENSG00000188215	8m	TargetScan
miR-7161-3p	ENSG00000188227	8m	TargetScan
miR-7161-3p	ENSG00000188266	8m	TargetScan
miR-7161-3p	ENSG00000188282	8m	TargetScan
miR-7161-3p	ENSG00000188343	8m	TargetScan
miR-7161-3p	ENSG00000188386	8m	TargetScan
miR-7161-3p	ENSG00000188730	8m	TargetScan
miR-7161-3p	ENSG00000188811	8m	TargetScan
miR-7161-3p	ENSG00000189057	8m	TargetScan
miR-7161-3p	ENSG00000189180	8m	TargetScan
miR-7161-3p	ENSG00000189190	8m	TargetScan
miR-7161-3p	ENSG00000189233	8m	TargetScan
miR-7161-3p	ENSG00000196208	8m	TargetScan
miR-7161-3p	ENSG00000196233	8m	TargetScan
miR-7161-3p	ENSG00000196284	8m	TargetScan
miR-7161-3p	ENSG00000196381	8m	TargetScan
miR-7161-3p	ENSG00000196504	8m	TargetScan
miR-7161-3p	ENSG00000196549	8m	TargetScan
miR-7161-3p	ENSG00000196693	8m	TargetScan
miR-7161-3p	ENSG00000196876	8m	TargetScan
miR-7161-3p	ENSG00000196937	8m	TargetScan
miR-7161-3p	ENSG00000197063	8m	TargetScan
miR-7161-3p	ENSG00000197142	8m	TargetScan

miR-7161-3p	ENSG00000197181	8m	TargetScan
miR-7161-3p	ENSG00000197183	8m	TargetScan
miR-7161-3p	ENSG00000197506	8m	TargetScan
miR-7161-3p	ENSG00000197557	8m	TargetScan
miR-7161-3p	ENSG00000197665	8m	TargetScan
miR-7161-3p	ENSG00000197724	8m	TargetScan
miR-7161-3p	ENSG00000197782	8m	TargetScan
miR-7161-3p	ENSG00000198015	8m	TargetScan
miR-7161-3p	ENSG00000198108	8m	TargetScan
miR-7161-3p	ENSG00000198162	8m	TargetScan
miR-7161-3p	ENSG00000198168	8m	TargetScan
miR-7161-3p	ENSG00000198464	8m	TargetScan
miR-7161-3p	ENSG00000198561	8m	TargetScan
miR-7161-3p	ENSG00000198677	8m	TargetScan
miR-7161-3p	ENSG00000198826	8m	TargetScan
miR-7161-3p	ENSG00000198873	8m	TargetScan
miR-7161-3p	ENSG00000198933	8m	TargetScan
miR-7161-3p	ENSG00000198944	8m	TargetScan
miR-7161-3p	ENSG00000204022	8m	TargetScan
miR-7161-3p	ENSG00000204118	8m	TargetScan
miR-7161-3p	ENSG00000204130	8m	TargetScan
miR-7161-3p	ENSG00000204179	8m	TargetScan
miR-7161-3p	ENSG00000204262	8m	TargetScan
miR-7161-3p	ENSG00000204397	8m	TargetScan
miR-7161-3p	ENSG00000204580	8m	TargetScan
miR-7161-3p	ENSG00000204634	8m	TargetScan
miR-7161-3p	ENSG00000204694	8m	TargetScan
miR-7161-3p	ENSG00000205084	8m	TargetScan
miR-7161-3p	ENSG00000205269	8m	TargetScan
miR-7161-3p	ENSG00000205339	8m	TargetScan
miR-7161-3p	ENSG00000205413	8m	TargetScan

miR-7161-3p	ENSG00000205726	8m	TargetScan
miR-7161-3p	ENSG00000206538	8m	TargetScan
miR-7161-3p	ENSG00000206557	8m	TargetScan
miR-7161-3p	ENSG00000213029	8m	TargetScan
miR-7161-3p	ENSG00000213160	8m	TargetScan
miR-7161-3p	ENSG00000213186	8m	TargetScan
miR-7161-3p	ENSG00000213337	8m	TargetScan
miR-7161-3p	ENSG00000213551	8m	TargetScan
miR-7161-3p	ENSG00000213625	8m	TargetScan
miR-7161-3p	ENSG00000213760	8m	TargetScan
miR-7161-3p	ENSG00000213762	8m	TargetScan
miR-7161-3p	ENSG00000214216	8m	TargetScan
miR-7161-3p	ENSG00000214456	8m	TargetScan
miR-7161-3p	ENSG00000217930	8m	TargetScan
miR-7161-3p	ENSG00000218336	8m	TargetScan
miR-7161-3p	ENSG00000224531	8m	TargetScan
miR-7161-3p	ENSG00000228157	8m	TargetScan
miR-7161-3p	ENSG00000241388	8m	TargetScan
miR-7161-3p	ENSG00000242265	8m	TargetScan
miR-7161-3p	ENSG00000248511	8m	TargetScan
miR-7161-3p	ENSG00000251201	8m	TargetScan
miR-7161-3p	ENSG00000256087	8m	TargetScan
miR-7161-3p	ENSG00000259332	8m	TargetScan
miR-7161-3p	ENSG00000264364	8m	TargetScan
miR-7161-3p	ENSG00000267618	8m	TargetScan
miR-10527	ENSG00000000419	7mer-m8	TargetScan
miR-10527	ENSG00000001497	7mer-m8	TargetScan
miR-10527	ENSG00000002587	7mer-m8	TargetScan
miR-10527	ENSG00000002746	7mer-m8	TargetScan
miR-10527	ENSG00000004846	7mer-m8	TargetScan
miR-10527	ENSG00000004897	7mer-m8	TargetScan

miR-10527	ENSG00000005020	7mer-m8	TargetScan
miR-10527	ENSG00000005108	7mer-m8	TargetScan
miR-10527	ENSG00000005483	7mer-m8	TargetScan
miR-10527	ENSG00000005893	7mer-m8	TargetScan
miR-10527	ENSG00000006459	7mer-m8	TargetScan
miR-10527	ENSG00000006715	7mer-m8	TargetScan
miR-10527	ENSG00000006747	7mer-m8	TargetScan
miR-10527	ENSG00000007372	7mer-m8	TargetScan
miR-10527	ENSG00000008282	7mer-m8	TargetScan
miR-10527	ENSG00000008405	7mer-m8	TargetScan
miR-10527	ENSG00000009694	7mer-m8	TargetScan
miR-10527	ENSG00000009780	7mer-m8	TargetScan
miR-10527	ENSG00000010404	7mer-m8	TargetScan
miR-10527	ENSG00000010610	7mer-m8	TargetScan
miR-10527	ENSG00000010818	7mer-m8	TargetScan
miR-10527	ENSG00000011347	7mer-m8	TargetScan
miR-10527	ENSG00000011454	7mer-m8	TargetScan
miR-10527	ENSG00000011566	7mer-m8	TargetScan
miR-10527	ENSG00000012817	7mer-m8	TargetScan
miR-10527	ENSG00000012983	7mer-m8	TargetScan
miR-10527	ENSG00000013583	7mer-m8	TargetScan
miR-10527	ENSG00000015153	7mer-m8	TargetScan
miR-10527	ENSG00000017427	7mer-m8	TargetScan
miR-10527	ENSG00000018236	7mer-m8	TargetScan
miR-10527	ENSG00000018869	7mer-m8	TargetScan
miR-10527	ENSG00000019995	7mer-m8	TargetScan
miR-10527	ENSG00000020426	7mer-m8	TargetScan
miR-10527	ENSG00000021776	7mer-m8	TargetScan
miR-10527	ENSG00000023516	7mer-m8	TargetScan
miR-10527	ENSG00000025039	7mer-m8	TargetScan
miR-10527	ENSG00000026103	7mer-m8	TargetScan

miR-10527	ENSG00000029363	7mer-m8	TargetScan
miR-10527	ENSG00000036473	7mer-m8	TargetScan
miR-10527	ENSG00000037637	7mer-m8	TargetScan
miR-10527	ENSG00000038532	7mer-m8	TargetScan
miR-10527	ENSG00000040199	7mer-m8	TargetScan
miR-10527	ENSG00000041515	7mer-m8	TargetScan
miR-10527	ENSG00000042781	7mer-m8	TargetScan
miR-10527	ENSG00000043093	7mer-m8	TargetScan
miR-10527	ENSG00000046653	7mer-m8	TargetScan
miR-10527	ENSG00000046774	7mer-m8	TargetScan
miR-10527	ENSG00000046889	7mer-m8	TargetScan
miR-10527	ENSG00000047634	7mer-m8	TargetScan
miR-10527	ENSG00000047849	7mer-m8	TargetScan
miR-10527	ENSG00000050393	7mer-m8	TargetScan
miR-10527	ENSG00000050748	7mer-m8	TargetScan
miR-10527	ENSG00000051523	7mer-m8	TargetScan
miR-10527	ENSG00000053254	7mer-m8	TargetScan
miR-10527	ENSG00000053328	7mer-m8	TargetScan
miR-10527	ENSG00000053770	7mer-m8	TargetScan
miR-10527	ENSG00000054690	7mer-m8	TargetScan
miR-10527	ENSG00000055813	7mer-m8	TargetScan
miR-10527	ENSG00000055917	7mer-m8	TargetScan
miR-10527	ENSG00000056586	7mer-m8	TargetScan
miR-10527	ENSG00000057657	7mer-m8	TargetScan
miR-10527	ENSG00000057935	7mer-m8	TargetScan
miR-10527	ENSG00000058091	7mer-m8	TargetScan
miR-10527	ENSG00000058272	7mer-m8	TargetScan
miR-10527	ENSG00000058600	7mer-m8	TargetScan
miR-10527	ENSG00000058729	7mer-m8	TargetScan
miR-10527	ENSG00000058866	7mer-m8	TargetScan
miR-10527	ENSG00000059758	7mer-m8	TargetScan

miR-10527	ENSG00000061455	7mer-m8	TargetScan
miR-10527	ENSG00000061676	7mer-m8	TargetScan
miR-10527	ENSG00000061987	7mer-m8	TargetScan
miR-10527	ENSG00000062194	7mer-m8	TargetScan
miR-10527	ENSG00000063438	7mer-m8	TargetScan
miR-10527	ENSG00000064313	7mer-m8	TargetScan
miR-10527	ENSG00000065135	7mer-m8	TargetScan
miR-10527	ENSG00000065308	7mer-m8	TargetScan
miR-10527	ENSG00000065325	7mer-m8	TargetScan
miR-10527	ENSG00000065809	7mer-m8	TargetScan
miR-10527	ENSG00000065882	7mer-m8	TargetScan
miR-10527	ENSG00000065883	7mer-m8	TargetScan
miR-10527	ENSG00000065923	7mer-m8	TargetScan
miR-10527	ENSG00000066084	7mer-m8	TargetScan
miR-10527	ENSG00000066557	7mer-m8	TargetScan
miR-10527	ENSG00000067082	7mer-m8	TargetScan
miR-10527	ENSG00000067182	7mer-m8	TargetScan
miR-10527	ENSG00000067208	7mer-m8	TargetScan
miR-10527	ENSG00000067533	7mer-m8	TargetScan
miR-10527	ENSG00000068745	7mer-m8	TargetScan
miR-10527	ENSG00000069188	7mer-m8	TargetScan
miR-10527	ENSG00000069431	7mer-m8	TargetScan
miR-10527	ENSG00000070018	7mer-m8	TargetScan
miR-10527	ENSG00000070214	7mer-m8	TargetScan
miR-10527	ENSG00000070269	7mer-m8	TargetScan
miR-10527	ENSG00000070367	7mer-m8	TargetScan
miR-10527	ENSG00000070476	7mer-m8	TargetScan
miR-10527	ENSG00000070614	7mer-m8	TargetScan
miR-10527	ENSG00000070882	7mer-m8	TargetScan
miR-10527	ENSG00000071242	7mer-m8	TargetScan
miR-10527	ENSG00000071539	7mer-m8	TargetScan

miR-10527	ENSG00000072401	7mer-m8	TargetScan
miR-10527	ENSG00000072415	7mer-m8	TargetScan
miR-10527	ENSG00000072501	7mer-m8	TargetScan
miR-10527	ENSG00000072657	7mer-m8	TargetScan
miR-10527	ENSG00000072786	7mer-m8	TargetScan
miR-10527	ENSG00000073282	7mer-m8	TargetScan
miR-10527	ENSG00000073614	7mer-m8	TargetScan
miR-10527	ENSG00000073712	7mer-m8	TargetScan
miR-10527	ENSG00000074966	7mer-m8	TargetScan
miR-10527	ENSG00000075426	7mer-m8	TargetScan
miR-10527	ENSG00000076003	7mer-m8	TargetScan
miR-10527	ENSG00000076053	7mer-m8	TargetScan
miR-10527	ENSG00000076248	7mer-m8	TargetScan
miR-10527	ENSG00000076641	7mer-m8	TargetScan
miR-10527	ENSG00000077147	7mer-m8	TargetScan
miR-10527	ENSG00000077232	7mer-m8	TargetScan
miR-10527	ENSG00000077420	7mer-m8	TargetScan
miR-10527	ENSG00000077514	7mer-m8	TargetScan
miR-10527	ENSG00000078098	7mer-m8	TargetScan
miR-10527	ENSG00000078114	7mer-m8	TargetScan
miR-10527	ENSG00000078124	7mer-m8	TargetScan
miR-10527	ENSG00000078140	7mer-m8	TargetScan
miR-10527	ENSG00000078304	7mer-m8	TargetScan
miR-10527	ENSG00000078795	7mer-m8	TargetScan
miR-10527	ENSG00000078967	7mer-m8	TargetScan
miR-10527	ENSG00000079156	7mer-m8	TargetScan
miR-10527	ENSG00000080298	7mer-m8	TargetScan
miR-10527	ENSG00000080345	7mer-m8	TargetScan
miR-10527	ENSG00000080561	7mer-m8	TargetScan
miR-10527	ENSG00000080802	7mer-m8	TargetScan
miR-10527	ENSG00000081014	7mer-m8	TargetScan

miR-10527	ENSG00000081019	7mer-m8	TargetScan
miR-10527	ENSG00000081051	7mer-m8	TargetScan
miR-10527	ENSG00000081189	7mer-m8	TargetScan
miR-10527	ENSG00000081320	7mer-m8	TargetScan
miR-10527	ENSG00000081800	7mer-m8	TargetScan
miR-10527	ENSG00000082068	7mer-m8	TargetScan
miR-10527	ENSG00000082175	7mer-m8	TargetScan
miR-10527	ENSG00000082212	7mer-m8	TargetScan
miR-10527	ENSG00000082269	7mer-m8	TargetScan
miR-10527	ENSG00000082512	7mer-m8	TargetScan
miR-10527	ENSG00000083312	7mer-m8	TargetScan
miR-10527	ENSG00000083844	7mer-m8	TargetScan
miR-10527	ENSG00000083937	7mer-m8	TargetScan
miR-10527	ENSG00000084070	7mer-m8	TargetScan
miR-10527	ENSG00000084090	7mer-m8	TargetScan
miR-10527	ENSG00000084710	7mer-m8	TargetScan
miR-10527	ENSG00000084733	7mer-m8	TargetScan
miR-10527	ENSG00000085224	7mer-m8	TargetScan
miR-10527	ENSG00000085831	7mer-m8	TargetScan
miR-10527	ENSG00000088833	7mer-m8	TargetScan
miR-10527	ENSG00000088854	7mer-m8	TargetScan
miR-10527	ENSG00000089048	7mer-m8	TargetScan
miR-10527	ENSG00000089050	7mer-m8	TargetScan
miR-10527	ENSG00000089818	7mer-m8	TargetScan
miR-10527	ENSG00000091317	7mer-m8	TargetScan
miR-10527	ENSG00000091436	7mer-m8	TargetScan
miR-10527	ENSG00000091844	7mer-m8	TargetScan
miR-10527	ENSG00000092439	7mer-m8	TargetScan
miR-10527	ENSG00000092820	7mer-m8	TargetScan
miR-10527	ENSG00000092978	7mer-m8	TargetScan
miR-10527	ENSG00000093072	7mer-m8	TargetScan

miR-10527	ENSG00000095303	7mer-m8	TargetScan
miR-10527	ENSG00000095739	7mer-m8	TargetScan
miR-10527	ENSG00000095787	7mer-m8	TargetScan
miR-10527	ENSG00000096060	7mer-m8	TargetScan
miR-10527	ENSG00000096063	7mer-m8	TargetScan
miR-10527	ENSG00000096401	7mer-m8	TargetScan
miR-10527	ENSG00000099139	7mer-m8	TargetScan
miR-10527	ENSG00000099246	7mer-m8	TargetScan
miR-10527	ENSG00000099721	7mer-m8	TargetScan
miR-10527	ENSG00000099817	7mer-m8	TargetScan
miR-10527	ENSG00000099942	7mer-m8	TargetScan
miR-10527	ENSG00000100030	7mer-m8	TargetScan
miR-10527	ENSG00000100330	7mer-m8	TargetScan
miR-10527	ENSG00000100344	7mer-m8	TargetScan
miR-10527	ENSG00000100345	7mer-m8	TargetScan
miR-10527	ENSG00000100346	7mer-m8	TargetScan
miR-10527	ENSG00000100354	7mer-m8	TargetScan
miR-10527	ENSG00000100364	7mer-m8	TargetScan
miR-10527	ENSG00000100380	7mer-m8	TargetScan
miR-10527	ENSG00000100461	7mer-m8	TargetScan
miR-10527	ENSG00000100478	7mer-m8	TargetScan
miR-10527	ENSG00000100523	7mer-m8	TargetScan
miR-10527	ENSG00000100568	7mer-m8	TargetScan
miR-10527	ENSG00000100583	7mer-m8	TargetScan
miR-10527	ENSG00000100614	7mer-m8	TargetScan
miR-10527	ENSG00000100664	7mer-m8	TargetScan
miR-10527	ENSG00000100678	7mer-m8	TargetScan
miR-10527	ENSG00000100697	7mer-m8	TargetScan
miR-10527	ENSG00000100934	7mer-m8	TargetScan
miR-10527	ENSG00000100994	7mer-m8	TargetScan
miR-10527	ENSG00000101019	7mer-m8	TargetScan

miR-10527	ENSG00000101247	7mer-m8	TargetScan
miR-10527	ENSG00000101290	7mer-m8	TargetScan
miR-10527	ENSG00000101327	7mer-m8	TargetScan
miR-10527	ENSG00000101413	7mer-m8	TargetScan
miR-10527	ENSG00000101544	7mer-m8	TargetScan
miR-10527	ENSG00000101746	7mer-m8	TargetScan
miR-10527	ENSG00000101888	7mer-m8	TargetScan
miR-10527	ENSG00000101901	7mer-m8	TargetScan
miR-10527	ENSG00000101938	7mer-m8	TargetScan
miR-10527	ENSG00000101958	7mer-m8	TargetScan
miR-10527	ENSG00000102024	7mer-m8	TargetScan
miR-10527	ENSG00000102043	7mer-m8	TargetScan
miR-10527	ENSG00000102053	7mer-m8	TargetScan
miR-10527	ENSG00000102098	7mer-m8	TargetScan
miR-10527	ENSG00000102181	7mer-m8	TargetScan
miR-10527	ENSG00000102471	7mer-m8	TargetScan
miR-10527	ENSG00000102743	7mer-m8	TargetScan
miR-10527	ENSG00000102804	7mer-m8	TargetScan
miR-10527	ENSG00000102897	7mer-m8	TargetScan
miR-10527	ENSG00000102910	7mer-m8	TargetScan
miR-10527	ENSG00000103037	7mer-m8	TargetScan
miR-10527	ENSG00000103061	7mer-m8	TargetScan
miR-10527	ENSG00000103356	7mer-m8	TargetScan
miR-10527	ENSG00000103460	7mer-m8	TargetScan
miR-10527	ENSG00000103540	7mer-m8	TargetScan
miR-10527	ENSG00000103710	7mer-m8	TargetScan
miR-10527	ENSG00000103994	7mer-m8	TargetScan
miR-10527	ENSG00000104142	7mer-m8	TargetScan
miR-10527	ENSG00000104164	7mer-m8	TargetScan
miR-10527	ENSG00000104343	7mer-m8	TargetScan
miR-10527	ENSG00000104361	7mer-m8	TargetScan

miR-10527	ENSG00000104497	7mer-m8	TargetScan
miR-10527	ENSG00000104723	7mer-m8	TargetScan
miR-10527	ENSG00000104756	7mer-m8	TargetScan
miR-10527	ENSG00000105392	7mer-m8	TargetScan
miR-10527	ENSG00000105583	7mer-m8	TargetScan
miR-10527	ENSG00000105810	7mer-m8	TargetScan
miR-10527	ENSG00000105829	7mer-m8	TargetScan
miR-10527	ENSG00000105855	7mer-m8	TargetScan
miR-10527	ENSG00000105879	7mer-m8	TargetScan
miR-10527	ENSG00000106086	7mer-m8	TargetScan
miR-10527	ENSG00000106258	7mer-m8	TargetScan
miR-10527	ENSG00000106346	7mer-m8	TargetScan
miR-10527	ENSG00000106355	7mer-m8	TargetScan
miR-10527	ENSG00000106546	7mer-m8	TargetScan
miR-10527	ENSG00000106692	7mer-m8	TargetScan
miR-10527	ENSG00000106714	7mer-m8	TargetScan
miR-10527	ENSG00000106723	7mer-m8	TargetScan
miR-10527	ENSG00000106733	7mer-m8	TargetScan
miR-10527	ENSG00000106823	7mer-m8	TargetScan
miR-10527	ENSG00000107077	7mer-m8	TargetScan
miR-10527	ENSG00000107341	7mer-m8	TargetScan
miR-10527	ENSG00000107362	7mer-m8	TargetScan
miR-10527	ENSG00000107554	7mer-m8	TargetScan
miR-10527	ENSG00000107560	7mer-m8	TargetScan
miR-10527	ENSG00000107614	7mer-m8	TargetScan
miR-10527	ENSG00000107771	7mer-m8	TargetScan
miR-10527	ENSG00000108061	7mer-m8	TargetScan
miR-10527	ENSG00000108064	7mer-m8	TargetScan
miR-10527	ENSG00000108239	7mer-m8	TargetScan
miR-10527	ENSG00000108256	7mer-m8	TargetScan
miR-10527	ENSG00000108384	7mer-m8	TargetScan

miR-10527	ENSG00000108474	7mer-m8	TargetScan
miR-10527	ENSG00000108576	7mer-m8	TargetScan
miR-10527	ENSG00000108587	7mer-m8	TargetScan
miR-10527	ENSG00000108654	7mer-m8	TargetScan
miR-10527	ENSG00000108666	7mer-m8	TargetScan
miR-10527	ENSG00000108861	7mer-m8	TargetScan
miR-10527	ENSG00000108950	7mer-m8	TargetScan
miR-10527	ENSG00000108984	7mer-m8	TargetScan
miR-10527	ENSG00000109101	7mer-m8	TargetScan
miR-10527	ENSG00000109220	7mer-m8	TargetScan
miR-10527	ENSG00000109332	7mer-m8	TargetScan
miR-10527	ENSG00000109861	7mer-m8	TargetScan
miR-10527	ENSG00000109917	7mer-m8	TargetScan
miR-10527	ENSG00000110218	7mer-m8	TargetScan
miR-10527	ENSG00000110315	7mer-m8	TargetScan
miR-10527	ENSG00000110395	7mer-m8	TargetScan
miR-10527	ENSG00000110422	7mer-m8	TargetScan
miR-10527	ENSG00000110427	7mer-m8	TargetScan
miR-10527	ENSG00000110429	7mer-m8	TargetScan
miR-10527	ENSG00000110713	7mer-m8	TargetScan
miR-10527	ENSG00000110906	7mer-m8	TargetScan
miR-10527	ENSG00000111049	7mer-m8	TargetScan
miR-10527	ENSG00000111142	7mer-m8	TargetScan
miR-10527	ENSG00000111229	7mer-m8	TargetScan
miR-10527	ENSG00000111328	7mer-m8	TargetScan
miR-10527	ENSG00000111424	7mer-m8	TargetScan
miR-10527	ENSG00000111704	7mer-m8	TargetScan
miR-10527	ENSG00000111713	7mer-m8	TargetScan
miR-10527	ENSG00000111728	7mer-m8	TargetScan
miR-10527	ENSG00000111816	7mer-m8	TargetScan
miR-10527	ENSG00000111860	7mer-m8	TargetScan

miR-10527	ENSG00000111885	7mer-m8	TargetScan
miR-10527	ENSG00000112062	7mer-m8	TargetScan
miR-10527	ENSG00000112280	7mer-m8	TargetScan
miR-10527	ENSG00000112308	7mer-m8	TargetScan
miR-10527	ENSG00000112379	7mer-m8	TargetScan
miR-10527	ENSG00000112531	7mer-m8	TargetScan
miR-10527	ENSG00000112619	7mer-m8	TargetScan
miR-10527	ENSG00000112624	7mer-m8	TargetScan
miR-10527	ENSG00000112773	7mer-m8	TargetScan
miR-10527	ENSG00000112837	7mer-m8	TargetScan
miR-10527	ENSG00000112902	7mer-m8	TargetScan
miR-10527	ENSG00000113048	7mer-m8	TargetScan
miR-10527	ENSG00000113100	7mer-m8	TargetScan
miR-10527	ENSG00000113240	7mer-m8	TargetScan
miR-10527	ENSG00000113249	7mer-m8	TargetScan
miR-10527	ENSG00000113273	7mer-m8	TargetScan
miR-10527	ENSG00000113448	7mer-m8	TargetScan
miR-10527	ENSG00000113456	7mer-m8	TargetScan
miR-10527	ENSG00000113583	7mer-m8	TargetScan
miR-10527	ENSG00000113597	7mer-m8	TargetScan
miR-10527	ENSG00000113600	7mer-m8	TargetScan
miR-10527	ENSG00000113638	7mer-m8	TargetScan
miR-10527	ENSG00000113658	7mer-m8	TargetScan
miR-10527	ENSG00000113851	7mer-m8	TargetScan
miR-10527	ENSG00000114021	7mer-m8	TargetScan
miR-10527	ENSG00000114023	7mer-m8	TargetScan
miR-10527	ENSG00000114127	7mer-m8	TargetScan
miR-10527	ENSG00000114166	7mer-m8	TargetScan
miR-10527	ENSG00000114279	7mer-m8	TargetScan
miR-10527	ENSG00000114349	7mer-m8	TargetScan
miR-10527	ENSG00000114416	7mer-m8	TargetScan

miR-10527	ENSG00000114491	7mer-m8	TargetScan
miR-10527	ENSG00000114850	7mer-m8	TargetScan
miR-10527	ENSG00000114902	7mer-m8	TargetScan
miR-10527	ENSG00000114988	7mer-m8	TargetScan
miR-10527	ENSG00000115137	7mer-m8	TargetScan
miR-10527	ENSG00000115165	7mer-m8	TargetScan
miR-10527	ENSG00000115252	7mer-m8	TargetScan
miR-10527	ENSG00000115295	7mer-m8	TargetScan
miR-10527	ENSG00000115355	7mer-m8	TargetScan
miR-10527	ENSG00000115364	7mer-m8	TargetScan
miR-10527	ENSG00000115464	7mer-m8	TargetScan
miR-10527	ENSG00000115524	7mer-m8	TargetScan
miR-10527	ENSG00000115593	7mer-m8	TargetScan
miR-10527	ENSG00000115641	7mer-m8	TargetScan
miR-10527	ENSG00000115827	7mer-m8	TargetScan
miR-10527	ENSG00000115839	7mer-m8	TargetScan
miR-10527	ENSG00000115942	7mer-m8	TargetScan
miR-10527	ENSG00000116095	7mer-m8	TargetScan
miR-10527	ENSG00000116132	7mer-m8	TargetScan
miR-10527	ENSG00000116171	7mer-m8	TargetScan
miR-10527	ENSG00000116191	7mer-m8	TargetScan
miR-10527	ENSG00000116194	7mer-m8	TargetScan
miR-10527	ENSG00000116199	7mer-m8	TargetScan
miR-10527	ENSG00000116473	7mer-m8	TargetScan
miR-10527	ENSG00000116560	7mer-m8	TargetScan
miR-10527	ENSG00000116661	7mer-m8	TargetScan
miR-10527	ENSG00000116667	7mer-m8	TargetScan
miR-10527	ENSG00000116704	7mer-m8	TargetScan
miR-10527	ENSG00000116985	7mer-m8	TargetScan
miR-10527	ENSG00000117054	7mer-m8	TargetScan
miR-10527	ENSG00000117139	7mer-m8	TargetScan

miR-10527	ENSG00000117151	7mer-m8	TargetScan
miR-10527	ENSG00000117479	7mer-m8	TargetScan
miR-10527	ENSG00000117500	7mer-m8	TargetScan
miR-10527	ENSG00000117505	7mer-m8	TargetScan
miR-10527	ENSG00000117507	7mer-m8	TargetScan
miR-10527	ENSG00000117533	7mer-m8	TargetScan
miR-10527	ENSG00000117724	7mer-m8	TargetScan
miR-10527	ENSG00000117868	7mer-m8	TargetScan
miR-10527	ENSG00000117906	7mer-m8	TargetScan
miR-10527	ENSG00000118096	7mer-m8	TargetScan
miR-10527	ENSG00000118242	7mer-m8	TargetScan
miR-10527	ENSG00000118260	7mer-m8	TargetScan
miR-10527	ENSG00000118596	7mer-m8	TargetScan
miR-10527	ENSG00000118707	7mer-m8	TargetScan
miR-10527	ENSG00000118855	7mer-m8	TargetScan
miR-10527	ENSG00000119396	7mer-m8	TargetScan
miR-10527	ENSG00000119397	7mer-m8	TargetScan
miR-10527	ENSG00000119508	7mer-m8	TargetScan
miR-10527	ENSG00000119547	7mer-m8	TargetScan
miR-10527	ENSG00000119685	7mer-m8	TargetScan
miR-10527	ENSG00000119723	7mer-m8	TargetScan
miR-10527	ENSG00000119760	7mer-m8	TargetScan
miR-10527	ENSG00000119772	7mer-m8	TargetScan
miR-10527	ENSG00000119778	7mer-m8	TargetScan
miR-10527	ENSG00000119812	7mer-m8	TargetScan
miR-10527	ENSG00000119878	7mer-m8	TargetScan
miR-10527	ENSG00000119899	7mer-m8	TargetScan
miR-10527	ENSG00000119922	7mer-m8	TargetScan
miR-10527	ENSG00000119929	7mer-m8	TargetScan
miR-10527	ENSG00000119950	7mer-m8	TargetScan
miR-10527	ENSG00000120159	7mer-m8	TargetScan

miR-10527	ENSG00000120251	7mer-m8	TargetScan
miR-10527	ENSG00000120289	7mer-m8	TargetScan
miR-10527	ENSG00000120332	7mer-m8	TargetScan
miR-10527	ENSG00000120519	7mer-m8	TargetScan
miR-10527	ENSG00000120539	7mer-m8	TargetScan
miR-10527	ENSG00000120647	7mer-m8	TargetScan
miR-10527	ENSG00000120669	7mer-m8	TargetScan
miR-10527	ENSG00000120738	7mer-m8	TargetScan
miR-10527	ENSG00000120805	7mer-m8	TargetScan
miR-10527	ENSG00000120832	7mer-m8	TargetScan
miR-10527	ENSG00000120885	7mer-m8	TargetScan
miR-10527	ENSG00000121060	7mer-m8	TargetScan
miR-10527	ENSG00000121297	7mer-m8	TargetScan
miR-10527	ENSG00000121390	7mer-m8	TargetScan
miR-10527	ENSG00000121454	7mer-m8	TargetScan
miR-10527	ENSG00000121579	7mer-m8	TargetScan
miR-10527	ENSG00000121716	7mer-m8	TargetScan
miR-10527	ENSG00000121897	7mer-m8	TargetScan
miR-10527	ENSG00000121904	7mer-m8	TargetScan
miR-10527	ENSG00000121931	7mer-m8	TargetScan
miR-10527	ENSG00000122068	7mer-m8	TargetScan
miR-10527	ENSG00000122176	7mer-m8	TargetScan
miR-10527	ENSG00000122224	7mer-m8	TargetScan
miR-10527	ENSG00000122367	7mer-m8	TargetScan
miR-10527	ENSG00000122417	7mer-m8	TargetScan
miR-10527	ENSG00000122691	7mer-m8	TargetScan
miR-10527	ENSG00000123094	7mer-m8	TargetScan
miR-10527	ENSG00000123130	7mer-m8	TargetScan
miR-10527	ENSG00000123191	7mer-m8	TargetScan
miR-10527	ENSG00000123240	7mer-m8	TargetScan
miR-10527	ENSG00000123268	7mer-m8	TargetScan

miR-10527	ENSG00000123411	7mer-m8	TargetScan
miR-10527	ENSG00000124120	7mer-m8	TargetScan
miR-10527	ENSG00000124193	7mer-m8	TargetScan
miR-10527	ENSG00000124198	7mer-m8	TargetScan
miR-10527	ENSG00000124406	7mer-m8	TargetScan
miR-10527	ENSG00000124596	7mer-m8	TargetScan
miR-10527	ENSG00000124743	7mer-m8	TargetScan
miR-10527	ENSG00000124772	7mer-m8	TargetScan
miR-10527	ENSG00000124783	7mer-m8	TargetScan
miR-10527	ENSG00000124813	7mer-m8	TargetScan
miR-10527	ENSG00000125124	7mer-m8	TargetScan
miR-10527	ENSG00000125247	7mer-m8	TargetScan
miR-10527	ENSG00000125351	7mer-m8	TargetScan
miR-10527	ENSG00000125363	7mer-m8	TargetScan
miR-10527	ENSG00000125398	7mer-m8	TargetScan
miR-10527	ENSG00000125498	7mer-m8	TargetScan
miR-10527	ENSG00000125657	7mer-m8	TargetScan
miR-10527	ENSG00000125735	7mer-m8	TargetScan
miR-10527	ENSG00000125810	7mer-m8	TargetScan
miR-10527	ENSG00000125812	7mer-m8	TargetScan
miR-10527	ENSG00000125813	7mer-m8	TargetScan
miR-10527	ENSG00000125818	7mer-m8	TargetScan
miR-10527	ENSG00000125868	7mer-m8	TargetScan
miR-10527	ENSG00000125870	7mer-m8	TargetScan
miR-10527	ENSG00000126070	7mer-m8	TargetScan
miR-10527	ENSG00000126550	7mer-m8	TargetScan
miR-10527	ENSG00000126822	7mer-m8	TargetScan
miR-10527	ENSG00000126953	7mer-m8	TargetScan
miR-10527	ENSG00000127152	7mer-m8	TargetScan
miR-10527	ENSG00000127328	7mer-m8	TargetScan
miR-10527	ENSG00000127663	7mer-m8	TargetScan

miR-10527	ENSG00000127831	7mer-m8	TargetScan
miR-10527	ENSG00000128039	7mer-m8	TargetScan
miR-10527	ENSG00000128534	7mer-m8	TargetScan
miR-10527	ENSG00000128573	7mer-m8	TargetScan
miR-10527	ENSG00000128578	7mer-m8	TargetScan
miR-10527	ENSG00000128585	7mer-m8	TargetScan
miR-10527	ENSG00000128683	7mer-m8	TargetScan
miR-10527	ENSG00000128708	7mer-m8	TargetScan
miR-10527	ENSG00000128849	7mer-m8	TargetScan
miR-10527	ENSG00000128908	7mer-m8	TargetScan
miR-10527	ENSG00000128915	7mer-m8	TargetScan
miR-10527	ENSG00000129204	7mer-m8	TargetScan
miR-10527	ENSG00000129250	7mer-m8	TargetScan
miR-10527	ENSG00000129317	7mer-m8	TargetScan
miR-10527	ENSG00000129460	7mer-m8	TargetScan
miR-10527	ENSG00000129474	7mer-m8	TargetScan
miR-10527	ENSG00000129566	7mer-m8	TargetScan
miR-10527	ENSG00000129625	7mer-m8	TargetScan
miR-10527	ENSG00000129675	7mer-m8	TargetScan
miR-10527	ENSG00000130052	7mer-m8	TargetScan
miR-10527	ENSG00000130119	7mer-m8	TargetScan
miR-10527	ENSG00000130227	7mer-m8	TargetScan
miR-10527	ENSG00000130818	7mer-m8	TargetScan
miR-10527	ENSG00000130844	7mer-m8	TargetScan
miR-10527	ENSG00000131016	7mer-m8	TargetScan
miR-10527	ENSG00000131127	7mer-m8	TargetScan
miR-10527	ENSG00000131269	7mer-m8	TargetScan
miR-10527	ENSG00000131355	7mer-m8	TargetScan
miR-10527	ENSG00000131386	7mer-m8	TargetScan
miR-10527	ENSG00000131437	7mer-m8	TargetScan
miR-10527	ENSG00000131459	7mer-m8	TargetScan

miR-10527	ENSG00000131558	7mer-m8	TargetScan
miR-10527	ENSG00000132031	7mer-m8	TargetScan
miR-10527	ENSG00000132294	7mer-m8	TargetScan
miR-10527	ENSG00000132570	7mer-m8	TargetScan
miR-10527	ENSG00000132604	7mer-m8	TargetScan
miR-10527	ENSG00000132623	7mer-m8	TargetScan
miR-10527	ENSG00000132640	7mer-m8	TargetScan
miR-10527	ENSG00000132846	7mer-m8	TargetScan
miR-10527	ENSG00000132855	7mer-m8	TargetScan
miR-10527	ENSG00000132970	7mer-m8	TargetScan
miR-10527	ENSG00000133028	7mer-m8	TargetScan
miR-10527	ENSG00000133083	7mer-m8	TargetScan
miR-10527	ENSG00000133104	7mer-m8	TargetScan
miR-10527	ENSG00000133115	7mer-m8	TargetScan
miR-10527	ENSG00000133313	7mer-m8	TargetScan
miR-10527	ENSG00000133574	7mer-m8	TargetScan
miR-10527	ENSG00000133703	7mer-m8	TargetScan
miR-10527	ENSG00000133742	7mer-m8	TargetScan
miR-10527	ENSG00000134046	7mer-m8	TargetScan
miR-10527	ENSG00000134109	7mer-m8	TargetScan
miR-10527	ENSG00000134121	7mer-m8	TargetScan
miR-10527	ENSG00000134202	7mer-m8	TargetScan
miR-10527	ENSG00000134216	7mer-m8	TargetScan
miR-10527	ENSG00000134243	7mer-m8	TargetScan
miR-10527	ENSG00000134245	7mer-m8	TargetScan
miR-10527	ENSG00000134291	7mer-m8	TargetScan
miR-10527	ENSG00000134326	7mer-m8	TargetScan
miR-10527	ENSG00000134352	7mer-m8	TargetScan
miR-10527	ENSG00000134371	7mer-m8	TargetScan
miR-10527	ENSG00000134463	7mer-m8	TargetScan
miR-10527	ENSG00000134759	7mer-m8	TargetScan

miR-10527	ENSG00000134809	7mer-m8	TargetScan
miR-10527	ENSG00000134852	7mer-m8	TargetScan
miR-10527	ENSG00000134864	7mer-m8	TargetScan
miR-10527	ENSG00000134909	7mer-m8	TargetScan
miR-10527	ENSG00000134970	7mer-m8	TargetScan
miR-10527	ENSG00000135049	7mer-m8	TargetScan
miR-10527	ENSG00000135083	7mer-m8	TargetScan
miR-10527	ENSG00000135185	7mer-m8	TargetScan
miR-10527	ENSG00000135250	7mer-m8	TargetScan
miR-10527	ENSG00000135315	7mer-m8	TargetScan
miR-10527	ENSG00000135643	7mer-m8	TargetScan
miR-10527	ENSG00000135686	7mer-m8	TargetScan
miR-10527	ENSG00000135750	7mer-m8	TargetScan
miR-10527	ENSG00000135778	7mer-m8	TargetScan
miR-10527	ENSG00000135870	7mer-m8	TargetScan
miR-10527	ENSG00000135999	7mer-m8	TargetScan
miR-10527	ENSG00000136052	7mer-m8	TargetScan
miR-10527	ENSG00000136104	7mer-m8	TargetScan
miR-10527	ENSG00000136235	7mer-m8	TargetScan
miR-10527	ENSG00000136237	7mer-m8	TargetScan
miR-10527	ENSG00000136267	7mer-m8	TargetScan
miR-10527	ENSG00000136450	7mer-m8	TargetScan
miR-10527	ENSG00000136463	7mer-m8	TargetScan
miR-10527	ENSG00000136527	7mer-m8	TargetScan
miR-10527	ENSG00000136603	7mer-m8	TargetScan
miR-10527	ENSG00000136636	7mer-m8	TargetScan
miR-10527	ENSG00000136643	7mer-m8	TargetScan
miR-10527	ENSG00000136710	7mer-m8	TargetScan
miR-10527	ENSG00000136738	7mer-m8	TargetScan
miR-10527	ENSG00000136848	7mer-m8	TargetScan
miR-10527	ENSG00000136861	7mer-m8	TargetScan

miR-10527	ENSG00000136870	7mer-m8	TargetScan
miR-10527	ENSG00000136938	7mer-m8	TargetScan
miR-10527	ENSG00000137261	7mer-m8	TargetScan
miR-10527	ENSG00000137561	7mer-m8	TargetScan
miR-10527	ENSG00000137573	7mer-m8	TargetScan
miR-10527	ENSG00000137642	7mer-m8	TargetScan
miR-10527	ENSG00000137691	7mer-m8	TargetScan
miR-10527	ENSG00000137692	7mer-m8	TargetScan
miR-10527	ENSG00000137804	7mer-m8	TargetScan
miR-10527	ENSG00000137814	7mer-m8	TargetScan
miR-10527	ENSG00000137819	7mer-m8	TargetScan
miR-10527	ENSG00000137821	7mer-m8	TargetScan
miR-10527	ENSG00000137845	7mer-m8	TargetScan
miR-10527	ENSG00000137869	7mer-m8	TargetScan
miR-10527	ENSG00000137878	7mer-m8	TargetScan
miR-10527	ENSG00000138032	7mer-m8	TargetScan
miR-10527	ENSG00000138039	7mer-m8	TargetScan
miR-10527	ENSG00000138078	7mer-m8	TargetScan
miR-10527	ENSG00000138079	7mer-m8	TargetScan
miR-10527	ENSG00000138185	7mer-m8	TargetScan
miR-10527	ENSG00000138380	7mer-m8	TargetScan
miR-10527	ENSG00000138411	7mer-m8	TargetScan
miR-10527	ENSG00000138435	7mer-m8	TargetScan
miR-10527	ENSG00000138593	7mer-m8	TargetScan
miR-10527	ENSG00000138594	7mer-m8	TargetScan
miR-10527	ENSG00000138613	7mer-m8	TargetScan
miR-10527	ENSG00000138660	7mer-m8	TargetScan
miR-10527	ENSG00000138668	7mer-m8	TargetScan
miR-10527	ENSG00000138756	7mer-m8	TargetScan
miR-10527	ENSG00000138764	7mer-m8	TargetScan
miR-10527	ENSG00000138829	7mer-m8	TargetScan

miR-10527	ENSG00000139083	7mer-m8	TargetScan
miR-10527	ENSG00000139131	7mer-m8	TargetScan
miR-10527	ENSG00000139154	7mer-m8	TargetScan
miR-10527	ENSG00000139178	7mer-m8	TargetScan
miR-10527	ENSG00000139218	7mer-m8	TargetScan
miR-10527	ENSG00000139233	7mer-m8	TargetScan
miR-10527	ENSG00000139289	7mer-m8	TargetScan
miR-10527	ENSG00000139343	7mer-m8	TargetScan
miR-10527	ENSG00000139436	7mer-m8	TargetScan
miR-10527	ENSG00000139496	7mer-m8	TargetScan
miR-10527	ENSG00000139514	7mer-m8	TargetScan
miR-10527	ENSG00000139874	7mer-m8	TargetScan
miR-10527	ENSG00000139926	7mer-m8	TargetScan
miR-10527	ENSG00000139977	7mer-m8	TargetScan
miR-10527	ENSG00000140153	7mer-m8	TargetScan
miR-10527	ENSG00000140157	7mer-m8	TargetScan
miR-10527	ENSG00000140396	7mer-m8	TargetScan
miR-10527	ENSG00000140455	7mer-m8	TargetScan
miR-10527	ENSG00000140478	7mer-m8	TargetScan
miR-10527	ENSG00000140563	7mer-m8	TargetScan
miR-10527	ENSG00000140718	7mer-m8	TargetScan
miR-10527	ENSG00000140743	7mer-m8	TargetScan
miR-10527	ENSG00000140807	7mer-m8	TargetScan
miR-10527	ENSG00000141449	7mer-m8	TargetScan
miR-10527	ENSG00000141540	7mer-m8	TargetScan
miR-10527	ENSG00000141627	7mer-m8	TargetScan
miR-10527	ENSG00000141646	7mer-m8	TargetScan
miR-10527	ENSG00000141655	7mer-m8	TargetScan
miR-10527	ENSG00000142166	7mer-m8	TargetScan
miR-10527	ENSG00000143079	7mer-m8	TargetScan
miR-10527	ENSG00000143226	7mer-m8	TargetScan

miR-10527	ENSG00000143228	7mer-m8	TargetScan
miR-10527	ENSG00000143324	7mer-m8	TargetScan
miR-10527	ENSG00000143355	7mer-m8	TargetScan
miR-10527	ENSG00000143367	7mer-m8	TargetScan
miR-10527	ENSG00000143384	7mer-m8	TargetScan
miR-10527	ENSG00000143390	7mer-m8	TargetScan
miR-10527	ENSG00000143443	7mer-m8	TargetScan
miR-10527	ENSG00000143479	7mer-m8	TargetScan
miR-10527	ENSG00000143514	7mer-m8	TargetScan
miR-10527	ENSG00000143549	7mer-m8	TargetScan
miR-10527	ENSG00000143771	7mer-m8	TargetScan
miR-10527	ENSG00000143850	7mer-m8	TargetScan
miR-10527	ENSG00000144152	7mer-m8	TargetScan
miR-10527	ENSG00000144278	7mer-m8	TargetScan
miR-10527	ENSG00000144290	7mer-m8	TargetScan
miR-10527	ENSG00000144320	7mer-m8	TargetScan
miR-10527	ENSG00000144481	7mer-m8	TargetScan
miR-10527	ENSG00000144597	7mer-m8	TargetScan
miR-10527	ENSG00000144619	7mer-m8	TargetScan
miR-10527	ENSG00000144668	7mer-m8	TargetScan
miR-10527	ENSG00000144713	7mer-m8	TargetScan
miR-10527	ENSG00000144791	7mer-m8	TargetScan
miR-10527	ENSG00000144824	7mer-m8	TargetScan
miR-10527	ENSG00000144868	7mer-m8	TargetScan
miR-10527	ENSG00000144959	7mer-m8	TargetScan
miR-10527	ENSG00000145016	7mer-m8	TargetScan
miR-10527	ENSG00000145022	7mer-m8	TargetScan
miR-10527	ENSG00000145246	7mer-m8	TargetScan
miR-10527	ENSG00000145283	7mer-m8	TargetScan
miR-10527	ENSG00000145284	7mer-m8	TargetScan
miR-10527	ENSG00000145386	7mer-m8	TargetScan

miR-10527	ENSG00000145388	7mer-m8	TargetScan
miR-10527	ENSG00000145391	7mer-m8	TargetScan
miR-10527	ENSG00000145414	7mer-m8	TargetScan
miR-10527	ENSG00000145495	7mer-m8	TargetScan
miR-10527	ENSG00000145569	7mer-m8	TargetScan
miR-10527	ENSG00000145685	7mer-m8	TargetScan
miR-10527	ENSG00000145687	7mer-m8	TargetScan
miR-10527	ENSG00000145715	7mer-m8	TargetScan
miR-10527	ENSG00000145741	7mer-m8	TargetScan
miR-10527	ENSG00000145743	7mer-m8	TargetScan
miR-10527	ENSG00000145780	7mer-m8	TargetScan
miR-10527	ENSG00000145861	7mer-m8	TargetScan
miR-10527	ENSG00000145864	7mer-m8	TargetScan
miR-10527	ENSG00000146085	7mer-m8	TargetScan
miR-10527	ENSG00000146151	7mer-m8	TargetScan
miR-10527	ENSG00000146242	7mer-m8	TargetScan
miR-10527	ENSG00000146352	7mer-m8	TargetScan
miR-10527	ENSG00000146376	7mer-m8	TargetScan
miR-10527	ENSG00000146416	7mer-m8	TargetScan
miR-10527	ENSG00000146433	7mer-m8	TargetScan
miR-10527	ENSG00000146476	7mer-m8	TargetScan
miR-10527	ENSG00000146530	7mer-m8	TargetScan
miR-10527	ENSG00000146535	7mer-m8	TargetScan
miR-10527	ENSG00000146592	7mer-m8	TargetScan
miR-10527	ENSG00000146676	7mer-m8	TargetScan
miR-10527	ENSG00000146733	7mer-m8	TargetScan
miR-10527	ENSG00000146802	7mer-m8	TargetScan
miR-10527	ENSG00000146833	7mer-m8	TargetScan
miR-10527	ENSG00000146858	7mer-m8	TargetScan
miR-10527	ENSG00000147036	7mer-m8	TargetScan
miR-10527	ENSG00000147100	7mer-m8	TargetScan

miR-10527	ENSG00000147124	7mer-m8	TargetScan
miR-10527	ENSG00000147162	7mer-m8	TargetScan
miR-10527	ENSG00000147164	7mer-m8	TargetScan
miR-10527	ENSG00000147202	7mer-m8	TargetScan
miR-10527	ENSG00000147251	7mer-m8	TargetScan
miR-10527	ENSG00000147400	7mer-m8	TargetScan
miR-10527	ENSG00000147421	7mer-m8	TargetScan
miR-10527	ENSG00000147459	7mer-m8	TargetScan
miR-10527	ENSG00000147548	7mer-m8	TargetScan
miR-10527	ENSG00000147586	7mer-m8	TargetScan
miR-10527	ENSG00000147642	7mer-m8	TargetScan
miR-10527	ENSG00000147669	7mer-m8	TargetScan
miR-10527	ENSG00000147677	7mer-m8	TargetScan
miR-10527	ENSG00000147679	7mer-m8	TargetScan
miR-10527	ENSG00000147885	7mer-m8	TargetScan
miR-10527	ENSG00000148053	7mer-m8	TargetScan
miR-10527	ENSG00000148219	7mer-m8	TargetScan
miR-10527	ENSG00000148225	7mer-m8	TargetScan
miR-10527	ENSG00000148229	7mer-m8	TargetScan
miR-10527	ENSG00000148468	7mer-m8	TargetScan
miR-10527	ENSG00000148672	7mer-m8	TargetScan
miR-10527	ENSG00000148719	7mer-m8	TargetScan
miR-10527	ENSG00000148730	7mer-m8	TargetScan
miR-10527	ENSG00000148943	7mer-m8	TargetScan
miR-10527	ENSG00000149089	7mer-m8	TargetScan
miR-10527	ENSG00000149100	7mer-m8	TargetScan
miR-10527	ENSG00000149177	7mer-m8	TargetScan
miR-10527	ENSG00000149212	7mer-m8	TargetScan
miR-10527	ENSG00000149289	7mer-m8	TargetScan
miR-10527	ENSG00000149554	7mer-m8	TargetScan
miR-10527	ENSG00000149599	7mer-m8	TargetScan

miR-10527	ENSG00000149636	7mer-m8	TargetScan
miR-10527	ENSG00000149968	7mer-m8	TargetScan
miR-10527	ENSG00000149972	7mer-m8	TargetScan
miR-10527	ENSG00000150471	7mer-m8	TargetScan
miR-10527	ENSG00000150540	7mer-m8	TargetScan
miR-10527	ENSG00000150637	7mer-m8	TargetScan
miR-10527	ENSG00000150991	7mer-m8	TargetScan
miR-10527	ENSG00000151067	7mer-m8	TargetScan
miR-10527	ENSG00000151233	7mer-m8	TargetScan
miR-10527	ENSG00000151348	7mer-m8	TargetScan
miR-10527	ENSG00000151422	7mer-m8	TargetScan
miR-10527	ENSG00000151458	7mer-m8	TargetScan
miR-10527	ENSG00000151491	7mer-m8	TargetScan
miR-10527	ENSG00000151632	7mer-m8	TargetScan
miR-10527	ENSG00000151881	7mer-m8	TargetScan
miR-10527	ENSG00000151893	7mer-m8	TargetScan
miR-10527	ENSG00000152056	7mer-m8	TargetScan
miR-10527	ENSG00000152061	7mer-m8	TargetScan
miR-10527	ENSG00000152256	7mer-m8	TargetScan
miR-10527	ENSG00000152402	7mer-m8	TargetScan
miR-10527	ENSG00000152409	7mer-m8	TargetScan
miR-10527	ENSG00000152439	7mer-m8	TargetScan
miR-10527	ENSG00000152443	7mer-m8	TargetScan
miR-10527	ENSG00000152518	7mer-m8	TargetScan
miR-10527	ENSG00000152558	7mer-m8	TargetScan
miR-10527	ENSG00000152601	7mer-m8	TargetScan
miR-10527	ENSG00000152684	7mer-m8	TargetScan
miR-10527	ENSG00000152763	7mer-m8	TargetScan
miR-10527	ENSG00000152785	7mer-m8	TargetScan
miR-10527	ENSG00000153006	7mer-m8	TargetScan
miR-10527	ENSG00000153037	7mer-m8	TargetScan

miR-10527	ENSG00000153132	7mer-m8	TargetScan
miR-10527	ENSG00000153147	7mer-m8	TargetScan
miR-10527	ENSG00000153214	7mer-m8	TargetScan
miR-10527	ENSG00000153317	7mer-m8	TargetScan
miR-10527	ENSG00000153487	7mer-m8	TargetScan
miR-10527	ENSG00000153561	7mer-m8	TargetScan
miR-10527	ENSG00000153684	7mer-m8	TargetScan
miR-10527	ENSG00000153767	7mer-m8	TargetScan
miR-10527	ENSG00000153790	7mer-m8	TargetScan
miR-10527	ENSG00000153922	7mer-m8	TargetScan
miR-10527	ENSG00000153933	7mer-m8	TargetScan
miR-10527	ENSG00000154114	7mer-m8	TargetScan
miR-10527	ENSG00000154118	7mer-m8	TargetScan
miR-10527	ENSG00000154124	7mer-m8	TargetScan
miR-10527	ENSG00000154144	7mer-m8	TargetScan
miR-10527	ENSG00000154162	7mer-m8	TargetScan
miR-10527	ENSG00000154174	7mer-m8	TargetScan
miR-10527	ENSG00000154217	7mer-m8	TargetScan
miR-10527	ENSG00000154227	7mer-m8	TargetScan
miR-10527	ENSG00000154310	7mer-m8	TargetScan
miR-10527	ENSG00000154359	7mer-m8	TargetScan
miR-10527	ENSG00000154415	7mer-m8	TargetScan
miR-10527	ENSG00000154654	7mer-m8	TargetScan
miR-10527	ENSG00000154678	7mer-m8	TargetScan
miR-10527	ENSG00000154719	7mer-m8	TargetScan
miR-10527	ENSG00000154736	7mer-m8	TargetScan
miR-10527	ENSG00000154889	7mer-m8	TargetScan
miR-10527	ENSG00000155052	7mer-m8	TargetScan
miR-10527	ENSG00000155097	7mer-m8	TargetScan
miR-10527	ENSG00000155100	7mer-m8	TargetScan
miR-10527	ENSG00000155189	7mer-m8	TargetScan

miR-10527	ENSG00000155636	7mer-m8	TargetScan
miR-10527	ENSG00000155754	7mer-m8	TargetScan
miR-10527	ENSG00000155827	7mer-m8	TargetScan
miR-10527	ENSG00000155850	7mer-m8	TargetScan
miR-10527	ENSG00000155868	7mer-m8	TargetScan
miR-10527	ENSG00000155957	7mer-m8	TargetScan
miR-10527	ENSG00000155975	7mer-m8	TargetScan
miR-10527	ENSG00000156096	7mer-m8	TargetScan
miR-10527	ENSG00000156097	7mer-m8	TargetScan
miR-10527	ENSG00000156113	7mer-m8	TargetScan
miR-10527	ENSG00000156239	7mer-m8	TargetScan
miR-10527	ENSG00000156269	7mer-m8	TargetScan
miR-10527	ENSG00000156284	7mer-m8	TargetScan
miR-10527	ENSG00000156508	7mer-m8	TargetScan
miR-10527	ENSG00000156535	7mer-m8	TargetScan
miR-10527	ENSG00000156671	7mer-m8	TargetScan
miR-10527	ENSG00000156853	7mer-m8	TargetScan
miR-10527	ENSG00000157106	7mer-m8	TargetScan
miR-10527	ENSG00000157315	7mer-m8	TargetScan
miR-10527	ENSG00000157500	7mer-m8	TargetScan
miR-10527	ENSG00000157600	7mer-m8	TargetScan
miR-10527	ENSG00000157680	7mer-m8	TargetScan
miR-10527	ENSG00000157741	7mer-m8	TargetScan
miR-10527	ENSG00000157764	7mer-m8	TargetScan
miR-10527	ENSG00000157985	7mer-m8	TargetScan
miR-10527	ENSG00000158006	7mer-m8	TargetScan
miR-10527	ENSG00000158290	7mer-m8	TargetScan
miR-10527	ENSG00000158296	7mer-m8	TargetScan
miR-10527	ENSG00000158352	7mer-m8	TargetScan
miR-10527	ENSG00000158373	7mer-m8	TargetScan
miR-10527	ENSG00000158528	7mer-m8	TargetScan

miR-10527	ENSG00000158683	7mer-m8	TargetScan
miR-10527	ENSG00000158805	7mer-m8	TargetScan
miR-10527	ENSG00000159167	7mer-m8	TargetScan
miR-10527	ENSG00000159197	7mer-m8	TargetScan
miR-10527	ENSG00000159217	7mer-m8	TargetScan
miR-10527	ENSG00000159251	7mer-m8	TargetScan
miR-10527	ENSG00000159263	7mer-m8	TargetScan
miR-10527	ENSG00000159289	7mer-m8	TargetScan
miR-10527	ENSG00000159692	7mer-m8	TargetScan
miR-10527	ENSG00000160007	7mer-m8	TargetScan
miR-10527	ENSG00000160679	7mer-m8	TargetScan
miR-10527	ENSG00000160959	7mer-m8	TargetScan
miR-10527	ENSG00000161547	7mer-m8	TargetScan
miR-10527	ENSG00000161791	7mer-m8	TargetScan
miR-10527	ENSG00000161929	7mer-m8	TargetScan
miR-10527	ENSG00000162105	7mer-m8	TargetScan
miR-10527	ENSG00000162174	7mer-m8	TargetScan
miR-10527	ENSG00000162241	7mer-m8	TargetScan
miR-10527	ENSG00000162368	7mer-m8	TargetScan
miR-10527	ENSG00000162402	7mer-m8	TargetScan
miR-10527	ENSG00000162438	7mer-m8	TargetScan
miR-10527	ENSG00000162441	7mer-m8	TargetScan
miR-10527	ENSG00000162616	7mer-m8	TargetScan
miR-10527	ENSG00000162642	7mer-m8	TargetScan
miR-10527	ENSG00000162654	7mer-m8	TargetScan
miR-10527	ENSG00000162664	7mer-m8	TargetScan
miR-10527	ENSG00000162695	7mer-m8	TargetScan
miR-10527	ENSG00000162769	7mer-m8	TargetScan
miR-10527	ENSG00000162928	7mer-m8	TargetScan
miR-10527	ENSG00000162980	7mer-m8	TargetScan
miR-10527	ENSG00000162999	7mer-m8	TargetScan

miR-10527	ENSG00000163110	7mer-m8	TargetScan
miR-10527	ENSG00000163249	7mer-m8	TargetScan
miR-10527	ENSG00000163281	7mer-m8	TargetScan
miR-10527	ENSG00000163291	7mer-m8	TargetScan
miR-10527	ENSG00000163297	7mer-m8	TargetScan
miR-10527	ENSG00000163428	7mer-m8	TargetScan
miR-10527	ENSG00000163430	7mer-m8	TargetScan
miR-10527	ENSG00000163481	7mer-m8	TargetScan
miR-10527	ENSG00000163492	7mer-m8	TargetScan
miR-10527	ENSG00000163513	7mer-m8	TargetScan
miR-10527	ENSG00000163581	7mer-m8	TargetScan
miR-10527	ENSG00000163584	7mer-m8	TargetScan
miR-10527	ENSG00000163590	7mer-m8	TargetScan
miR-10527	ENSG00000163602	7mer-m8	TargetScan
miR-10527	ENSG00000163605	7mer-m8	TargetScan
miR-10527	ENSG00000163611	7mer-m8	TargetScan
miR-10527	ENSG00000163637	7mer-m8	TargetScan
miR-10527	ENSG00000163644	7mer-m8	TargetScan
miR-10527	ENSG00000163689	7mer-m8	TargetScan
miR-10527	ENSG00000163728	7mer-m8	TargetScan
miR-10527	ENSG00000163812	7mer-m8	TargetScan
miR-10527	ENSG00000163818	7mer-m8	TargetScan
miR-10527	ENSG00000163833	7mer-m8	TargetScan
miR-10527	ENSG00000163900	7mer-m8	TargetScan
miR-10527	ENSG00000163909	7mer-m8	TargetScan
miR-10527	ENSG00000164024	7mer-m8	TargetScan
miR-10527	ENSG00000164056	7mer-m8	TargetScan
miR-10527	ENSG00000164087	7mer-m8	TargetScan
miR-10527	ENSG00000164116	7mer-m8	TargetScan
miR-10527	ENSG00000164122	7mer-m8	TargetScan
miR-10527	ENSG00000164181	7mer-m8	TargetScan

miR-10527	ENSG00000164241	7mer-m8	TargetScan
miR-10527	ENSG00000164244	7mer-m8	TargetScan
miR-10527	ENSG00000164303	7mer-m8	TargetScan
miR-10527	ENSG00000164307	7mer-m8	TargetScan
miR-10527	ENSG00000164323	7mer-m8	TargetScan
miR-10527	ENSG00000164338	7mer-m8	TargetScan
miR-10527	ENSG00000164483	7mer-m8	TargetScan
miR-10527	ENSG00000164485	7mer-m8	TargetScan
miR-10527	ENSG00000164506	7mer-m8	TargetScan
miR-10527	ENSG00000164532	7mer-m8	TargetScan
miR-10527	ENSG00000164619	7mer-m8	TargetScan
miR-10527	ENSG00000164684	7mer-m8	TargetScan
miR-10527	ENSG00000164691	7mer-m8	TargetScan
miR-10527	ENSG00000164746	7mer-m8	TargetScan
miR-10527	ENSG00000164764	7mer-m8	TargetScan
miR-10527	ENSG00000164941	7mer-m8	TargetScan
miR-10527	ENSG00000164953	7mer-m8	TargetScan
miR-10527	ENSG00000164976	7mer-m8	TargetScan
miR-10527	ENSG00000164983	7mer-m8	TargetScan
miR-10527	ENSG00000165028	7mer-m8	TargetScan
miR-10527	ENSG00000165084	7mer-m8	TargetScan
miR-10527	ENSG00000165185	7mer-m8	TargetScan
miR-10527	ENSG00000165186	7mer-m8	TargetScan
miR-10527	ENSG00000165195	7mer-m8	TargetScan
miR-10527	ENSG00000165244	7mer-m8	TargetScan
miR-10527	ENSG00000165338	7mer-m8	TargetScan
miR-10527	ENSG00000165416	7mer-m8	TargetScan
miR-10527	ENSG00000165417	7mer-m8	TargetScan
miR-10527	ENSG00000165458	7mer-m8	TargetScan
miR-10527	ENSG00000165474	7mer-m8	TargetScan
miR-10527	ENSG00000165556	7mer-m8	TargetScan

miR-10527	ENSG00000165566	7mer-m8	TargetScan
miR-10527	ENSG00000165623	7mer-m8	TargetScan
miR-10527	ENSG00000165671	7mer-m8	TargetScan
miR-10527	ENSG00000165714	7mer-m8	TargetScan
miR-10527	ENSG00000165775	7mer-m8	TargetScan
miR-10527	ENSG00000165959	7mer-m8	TargetScan
miR-10527	ENSG00000166128	7mer-m8	TargetScan
miR-10527	ENSG00000166147	7mer-m8	TargetScan
miR-10527	ENSG00000166206	7mer-m8	TargetScan
miR-10527	ENSG00000166233	7mer-m8	TargetScan
miR-10527	ENSG00000166262	7mer-m8	TargetScan
miR-10527	ENSG00000166351	7mer-m8	TargetScan
miR-10527	ENSG00000166402	7mer-m8	TargetScan
miR-10527	ENSG00000166435	7mer-m8	TargetScan
miR-10527	ENSG00000166441	7mer-m8	TargetScan
miR-10527	ENSG00000166450	7mer-m8	TargetScan
miR-10527	ENSG00000166478	7mer-m8	TargetScan
miR-10527	ENSG00000166503	7mer-m8	TargetScan
miR-10527	ENSG00000166510	7mer-m8	TargetScan
miR-10527	ENSG00000166575	7mer-m8	TargetScan
miR-10527	ENSG00000166783	7mer-m8	TargetScan
miR-10527	ENSG00000166803	7mer-m8	TargetScan
miR-10527	ENSG00000166900	7mer-m8	TargetScan
miR-10527	ENSG00000166927	7mer-m8	TargetScan
miR-10527	ENSG00000167186	7mer-m8	TargetScan
miR-10527	ENSG00000167195	7mer-m8	TargetScan
miR-10527	ENSG00000167196	7mer-m8	TargetScan
miR-10527	ENSG00000167202	7mer-m8	TargetScan
miR-10527	ENSG00000167232	7mer-m8	TargetScan
miR-10527	ENSG00000167315	7mer-m8	TargetScan
miR-10527	ENSG00000167526	7mer-m8	TargetScan

miR-10527	ENSG00000167633	7mer-m8	TargetScan
miR-10527	ENSG00000167642	7mer-m8	TargetScan
miR-10527	ENSG00000167910	7mer-m8	TargetScan
miR-10527	ENSG00000168032	7mer-m8	TargetScan
miR-10527	ENSG00000168172	7mer-m8	TargetScan
miR-10527	ENSG00000168175	7mer-m8	TargetScan
miR-10527	ENSG00000168288	7mer-m8	TargetScan
miR-10527	ENSG00000168297	7mer-m8	TargetScan
miR-10527	ENSG00000168538	7mer-m8	TargetScan
miR-10527	ENSG00000168556	7mer-m8	TargetScan
miR-10527	ENSG00000168672	7mer-m8	TargetScan
miR-10527	ENSG00000168769	7mer-m8	TargetScan
miR-10527	ENSG00000168876	7mer-m8	TargetScan
miR-10527	ENSG00000168903	7mer-m8	TargetScan
miR-10527	ENSG00000168939	7mer-m8	TargetScan
miR-10527	ENSG00000168939	7mer-m8	TargetScan
miR-10527	ENSG00000168944	7mer-m8	TargetScan
miR-10527	ENSG00000169047	7mer-m8	TargetScan
miR-10527	ENSG00000169139	7mer-m8	TargetScan
miR-10527	ENSG00000169184	7mer-m8	TargetScan
miR-10527	ENSG00000169239	7mer-m8	TargetScan
miR-10527	ENSG00000169282	7mer-m8	TargetScan
miR-10527	ENSG00000169306	7mer-m8	TargetScan
miR-10527	ENSG00000169375	7mer-m8	TargetScan
miR-10527	ENSG00000169446	7mer-m8	TargetScan
miR-10527	ENSG00000169554	7mer-m8	TargetScan
miR-10527	ENSG00000169836	7mer-m8	TargetScan
miR-10527	ENSG00000169905	7mer-m8	TargetScan
miR-10527	ENSG00000169908	7mer-m8	TargetScan
miR-10527	ENSG00000169925	7mer-m8	TargetScan
miR-10527	ENSG00000169981	7mer-m8	TargetScan

miR-10527	ENSG00000170035	7mer-m8	TargetScan
miR-10527	ENSG00000170074	7mer-m8	TargetScan
miR-10527	ENSG00000170075	7mer-m8	TargetScan
miR-10527	ENSG00000170088	7mer-m8	TargetScan
miR-10527	ENSG00000170145	7mer-m8	TargetScan
miR-10527	ENSG00000170162	7mer-m8	TargetScan
miR-10527	ENSG00000170166	7mer-m8	TargetScan
miR-10527	ENSG00000170345	7mer-m8	TargetScan
miR-10527	ENSG00000170348	7mer-m8	TargetScan
miR-10527	ENSG00000170545	7mer-m8	TargetScan
miR-10527	ENSG00000170558	7mer-m8	TargetScan
miR-10527	ENSG00000170624	7mer-m8	TargetScan
miR-10527	ENSG00000170634	7mer-m8	TargetScan
miR-10527	ENSG00000170653	7mer-m8	TargetScan
miR-10527	ENSG00000170681	7mer-m8	TargetScan
miR-10527	ENSG00000170832	7mer-m8	TargetScan
miR-10527	ENSG00000170836	7mer-m8	TargetScan
miR-10527	ENSG00000170854	7mer-m8	TargetScan
miR-10527	ENSG00000170892	7mer-m8	TargetScan
miR-10527	ENSG00000170927	7mer-m8	TargetScan
miR-10527	ENSG00000170949	7mer-m8	TargetScan
miR-10527	ENSG00000171016	7mer-m8	TargetScan
miR-10527	ENSG00000171033	7mer-m8	TargetScan
miR-10527	ENSG00000171150	7mer-m8	TargetScan
miR-10527	ENSG00000171208	7mer-m8	TargetScan
miR-10527	ENSG00000171227	7mer-m8	TargetScan
miR-10527	ENSG00000171310	7mer-m8	TargetScan
miR-10527	ENSG00000171316	7mer-m8	TargetScan
miR-10527	ENSG00000171320	7mer-m8	TargetScan
miR-10527	ENSG00000171385	7mer-m8	TargetScan
miR-10527	ENSG00000171444	7mer-m8	TargetScan

miR-10527	ENSG00000171448	7mer-m8	TargetScan
miR-10527	ENSG00000171490	7mer-m8	TargetScan
miR-10527	ENSG00000171492	7mer-m8	TargetScan
miR-10527	ENSG00000171634	7mer-m8	TargetScan
miR-10527	ENSG00000171649	7mer-m8	TargetScan
miR-10527	ENSG00000171659	7mer-m8	TargetScan
miR-10527	ENSG00000171757	7mer-m8	TargetScan
miR-10527	ENSG00000171865	7mer-m8	TargetScan
miR-10527	ENSG00000171885	7mer-m8	TargetScan
miR-10527	ENSG00000172081	7mer-m8	TargetScan
miR-10527	ENSG00000172167	7mer-m8	TargetScan
miR-10527	ENSG00000172209	7mer-m8	TargetScan
miR-10527	ENSG00000172273	7mer-m8	TargetScan
miR-10527	ENSG00000172340	7mer-m8	TargetScan
miR-10527	ENSG00000172379	7mer-m8	TargetScan
miR-10527	ENSG00000172399	7mer-m8	TargetScan
miR-10527	ENSG00000172493	7mer-m8	TargetScan
miR-10527	ENSG00000172572	7mer-m8	TargetScan
miR-10527	ENSG00000172578	7mer-m8	TargetScan
miR-10527	ENSG00000172728	7mer-m8	TargetScan
miR-10527	ENSG00000172733	7mer-m8	TargetScan
miR-10527	ENSG00000172795	7mer-m8	TargetScan
miR-10527	ENSG00000172939	7mer-m8	TargetScan
miR-10527	ENSG00000172943	7mer-m8	TargetScan
miR-10527	ENSG00000173068	7mer-m8	TargetScan
miR-10527	ENSG00000173083	7mer-m8	TargetScan
miR-10527	ENSG00000173110	7mer-m8	TargetScan
miR-10527	ENSG00000173141	7mer-m8	TargetScan
miR-10527	ENSG00000173209	7mer-m8	TargetScan
miR-10527	ENSG00000173276	7mer-m8	TargetScan
miR-10527	ENSG00000173611	7mer-m8	TargetScan

miR-10527	ENSG00000173614	7mer-m8	TargetScan
miR-10527	ENSG00000173626	7mer-m8	TargetScan
miR-10527	ENSG00000173627	7mer-m8	TargetScan
miR-10527	ENSG00000173674	7mer-m8	TargetScan
miR-10527	ENSG00000173681	7mer-m8	TargetScan
miR-10527	ENSG00000173692	7mer-m8	TargetScan
miR-10527	ENSG00000173805	7mer-m8	TargetScan
miR-10527	ENSG00000174099	7mer-m8	TargetScan
miR-10527	ENSG00000174130	7mer-m8	TargetScan
miR-10527	ENSG00000174206	7mer-m8	TargetScan
miR-10527	ENSG00000174370	7mer-m8	TargetScan
miR-10527	ENSG00000174437	7mer-m8	TargetScan
miR-10527	ENSG00000174473	7mer-m8	TargetScan
miR-10527	ENSG00000174840	7mer-m8	TargetScan
miR-10527	ENSG00000174891	7mer-m8	TargetScan
miR-10527	ENSG00000174989	7mer-m8	TargetScan
miR-10527	ENSG00000175029	7mer-m8	TargetScan
miR-10527	ENSG00000175066	7mer-m8	TargetScan
miR-10527	ENSG00000175073	7mer-m8	TargetScan
miR-10527	ENSG00000175215	7mer-m8	TargetScan
miR-10527	ENSG00000175426	7mer-m8	TargetScan
miR-10527	ENSG00000175787	7mer-m8	TargetScan
miR-10527	ENSG00000175806	7mer-m8	TargetScan
miR-10527	ENSG00000175874	7mer-m8	TargetScan
miR-10527	ENSG00000175893	7mer-m8	TargetScan
miR-10527	ENSG00000176049	7mer-m8	TargetScan
miR-10527	ENSG00000176102	7mer-m8	TargetScan
miR-10527	ENSG00000176222	7mer-m8	TargetScan
miR-10527	ENSG00000176463	7mer-m8	TargetScan
miR-10527	ENSG00000176595	7mer-m8	TargetScan
miR-10527	ENSG00000176597	7mer-m8	TargetScan

miR-10527	ENSG00000176658	7mer-m8	TargetScan
miR-10527	ENSG00000176907	7mer-m8	TargetScan
miR-10527	ENSG00000176945	7mer-m8	TargetScan
miR-10527	ENSG00000176971	7mer-m8	TargetScan
miR-10527	ENSG00000177181	7mer-m8	TargetScan
miR-10527	ENSG00000177182	7mer-m8	TargetScan
miR-10527	ENSG00000177383	7mer-m8	TargetScan
miR-10527	ENSG00000177425	7mer-m8	TargetScan
miR-10527	ENSG00000177511	7mer-m8	TargetScan
miR-10527	ENSG00000177570	7mer-m8	TargetScan
miR-10527	ENSG00000177602	7mer-m8	TargetScan
miR-10527	ENSG00000177606	7mer-m8	TargetScan
miR-10527	ENSG00000177989	7mer-m8	TargetScan
miR-10527	ENSG00000178074	7mer-m8	TargetScan
miR-10527	ENSG00000178177	7mer-m8	TargetScan
miR-10527	ENSG00000178425	7mer-m8	TargetScan
miR-10527	ENSG00000178502	7mer-m8	TargetScan
miR-10527	ENSG00000178567	7mer-m8	TargetScan
miR-10527	ENSG00000178568	7mer-m8	TargetScan
miR-10527	ENSG00000178573	7mer-m8	TargetScan
miR-10527	ENSG00000178607	7mer-m8	TargetScan
miR-10527	ENSG00000178694	7mer-m8	TargetScan
miR-10527	ENSG00000178700	7mer-m8	TargetScan
miR-10527	ENSG00000179008	7mer-m8	TargetScan
miR-10527	ENSG00000179195	7mer-m8	TargetScan
miR-10527	ENSG00000179387	7mer-m8	TargetScan
miR-10527	ENSG00000179813	7mer-m8	TargetScan
miR-10527	ENSG00000180008	7mer-m8	TargetScan
miR-10527	ENSG00000180354	7mer-m8	TargetScan
miR-10527	ENSG00000180488	7mer-m8	TargetScan
miR-10527	ENSG00000180530	7mer-m8	TargetScan

miR-10527	ENSG00000180611	7mer-m8	TargetScan
miR-10527	ENSG00000180667	7mer-m8	TargetScan
miR-10527	ENSG00000180776	7mer-m8	TargetScan
miR-10527	ENSG00000180869	7mer-m8	TargetScan
miR-10527	ENSG00000180901	7mer-m8	TargetScan
miR-10527	ENSG00000180964	7mer-m8	TargetScan
miR-10527	ENSG00000180998	7mer-m8	TargetScan
miR-10527	ENSG00000181016	7mer-m8	TargetScan
miR-10527	ENSG00000181031	7mer-m8	TargetScan
miR-10527	ENSG00000181450	7mer-m8	TargetScan
miR-10527	ENSG00000181631	7mer-m8	TargetScan
miR-10527	ENSG00000181704	7mer-m8	TargetScan
miR-10527	ENSG00000181722	7mer-m8	TargetScan
miR-10527	ENSG00000181908	7mer-m8	TargetScan
miR-10527	ENSG00000182013	7mer-m8	TargetScan
miR-10527	ENSG00000182230	7mer-m8	TargetScan
miR-10527	ENSG00000182263	7mer-m8	TargetScan
miR-10527	ENSG00000182348	7mer-m8	TargetScan
miR-10527	ENSG00000182389	7mer-m8	TargetScan
miR-10527	ENSG00000182446	7mer-m8	TargetScan
miR-10527	ENSG00000182463	7mer-m8	TargetScan
miR-10527	ENSG00000182552	7mer-m8	TargetScan
miR-10527	ENSG00000182568	7mer-m8	TargetScan
miR-10527	ENSG00000182575	7mer-m8	TargetScan
miR-10527	ENSG00000182667	7mer-m8	TargetScan
miR-10527	ENSG00000182836	7mer-m8	TargetScan
miR-10527	ENSG00000182919	7mer-m8	TargetScan
miR-10527	ENSG00000182968	7mer-m8	TargetScan
miR-10527	ENSG00000183023	7mer-m8	TargetScan
miR-10527	ENSG00000183145	7mer-m8	TargetScan
miR-10527	ENSG00000183291	7mer-m8	TargetScan

miR-10527	ENSG00000183323	7mer-m8	TargetScan
miR-10527	ENSG00000183454	7mer-m8	TargetScan
miR-10527	ENSG00000183530	7mer-m8	TargetScan
miR-10527	ENSG00000183729	7mer-m8	TargetScan
miR-10527	ENSG00000183742	7mer-m8	TargetScan
miR-10527	ENSG00000184083	7mer-m8	TargetScan
miR-10527	ENSG00000184206	7mer-m8	TargetScan
miR-10527	ENSG00000184226	7mer-m8	TargetScan
miR-10527	ENSG00000184349	7mer-m8	TargetScan
miR-10527	ENSG00000184574	7mer-m8	TargetScan
miR-10527	ENSG00000184792	7mer-m8	TargetScan
miR-10527	ENSG00000184992	7mer-m8	TargetScan
miR-10527	ENSG00000184995	7mer-m8	TargetScan
miR-10527	ENSG00000185008	7mer-m8	TargetScan
miR-10527	ENSG00000185046	7mer-m8	TargetScan
miR-10527	ENSG00000185070	7mer-m8	TargetScan
miR-10527	ENSG00000185088	7mer-m8	TargetScan
miR-10527	ENSG00000185090	7mer-m8	TargetScan
miR-10527	ENSG00000185149	7mer-m8	TargetScan
miR-10527	ENSG00000185163	7mer-m8	TargetScan
miR-10527	ENSG00000185219	7mer-m8	TargetScan
miR-10527	ENSG00000185247	7mer-m8	TargetScan
miR-10527	ENSG00000185278	7mer-m8	TargetScan
miR-10527	ENSG00000185379	7mer-m8	TargetScan
miR-10527	ENSG00000185414	7mer-m8	TargetScan
miR-10527	ENSG00000185551	7mer-m8	TargetScan
miR-10527	ENSG00000185565	7mer-m8	TargetScan
miR-10527	ENSG00000185591	7mer-m8	TargetScan
miR-10527	ENSG00000185658	7mer-m8	TargetScan
miR-10527	ENSG00000185722	7mer-m8	TargetScan
miR-10527	ENSG00000185829	7mer-m8	TargetScan

miR-10527	ENSG00000185947	7mer-m8	TargetScan
miR-10527	ENSG00000186094	7mer-m8	TargetScan
miR-10527	ENSG00000186265	7mer-m8	TargetScan
miR-10527	ENSG00000186272	7mer-m8	TargetScan
miR-10527	ENSG00000186298	7mer-m8	TargetScan
miR-10527	ENSG00000186399	7mer-m8	TargetScan
miR-10527	ENSG00000186479	7mer-m8	TargetScan
miR-10527	ENSG00000186529	7mer-m8	TargetScan
miR-10527	ENSG00000186960	7mer-m8	TargetScan
miR-10527	ENSG00000187049	7mer-m8	TargetScan
miR-10527	ENSG00000187187	7mer-m8	TargetScan
miR-10527	ENSG00000187189	7mer-m8	TargetScan
miR-10527	ENSG00000187231	7mer-m8	TargetScan
miR-10527	ENSG00000187446	7mer-m8	TargetScan
miR-10527	ENSG00000187581	7mer-m8	TargetScan
miR-10527	ENSG00000187605	7mer-m8	TargetScan
miR-10527	ENSG00000187626	7mer-m8	TargetScan
miR-10527	ENSG00000187676	7mer-m8	TargetScan
miR-10527	ENSG00000187699	7mer-m8	TargetScan
miR-10527	ENSG00000187824	7mer-m8	TargetScan
miR-10527	ENSG00000187942	7mer-m8	TargetScan
miR-10527	ENSG00000187954	7mer-m8	TargetScan
miR-10527	ENSG00000188001	7mer-m8	TargetScan
miR-10527	ENSG00000188021	7mer-m8	TargetScan
miR-10527	ENSG00000188107	7mer-m8	TargetScan
miR-10527	ENSG00000188419	7mer-m8	TargetScan
miR-10527	ENSG00000188610	7mer-m8	TargetScan
miR-10527	ENSG00000188626	7mer-m8	TargetScan
miR-10527	ENSG00000188786	7mer-m8	TargetScan
miR-10527	ENSG00000188937	7mer-m8	TargetScan
miR-10527	ENSG00000189013	7mer-m8	TargetScan

miR-10527	ENSG00000189046	7mer-m8	TargetScan
miR-10527	ENSG00000189164	7mer-m8	TargetScan
miR-10527	ENSG00000189180	7mer-m8	TargetScan
miR-10527	ENSG00000189319	7mer-m8	TargetScan
miR-10527	ENSG00000189362	7mer-m8	TargetScan
miR-10527	ENSG00000196110	7mer-m8	TargetScan
miR-10527	ENSG00000196199	7mer-m8	TargetScan
miR-10527	ENSG00000196220	7mer-m8	TargetScan
miR-10527	ENSG00000196247	7mer-m8	TargetScan
miR-10527	ENSG00000196262	7mer-m8	TargetScan
miR-10527	ENSG00000196352	7mer-m8	TargetScan
miR-10527	ENSG00000196368	7mer-m8	TargetScan
miR-10527	ENSG00000196405	7mer-m8	TargetScan
miR-10527	ENSG00000196417	7mer-m8	TargetScan
miR-10527	ENSG00000196455	7mer-m8	TargetScan
miR-10527	ENSG00000196504	7mer-m8	TargetScan
miR-10527	ENSG00000196505	7mer-m8	TargetScan
miR-10527	ENSG00000196510	7mer-m8	TargetScan
miR-10527	ENSG00000196550	7mer-m8	TargetScan
miR-10527	ENSG00000196693	7mer-m8	TargetScan
miR-10527	ENSG00000196705	7mer-m8	TargetScan
miR-10527	ENSG00000196792	7mer-m8	TargetScan
miR-10527	ENSG00000196932	7mer-m8	TargetScan
miR-10527	ENSG00000196968	7mer-m8	TargetScan
miR-10527	ENSG00000197045	7mer-m8	TargetScan
miR-10527	ENSG00000197077	7mer-m8	TargetScan
miR-10527	ENSG00000197128	7mer-m8	TargetScan
miR-10527	ENSG00000197172	7mer-m8	TargetScan
miR-10527	ENSG00000197296	7mer-m8	TargetScan
miR-10527	ENSG00000197312	7mer-m8	TargetScan
miR-10527	ENSG00000197363	7mer-m8	TargetScan

miR-10527	ENSG00000197430	7mer-m8	TargetScan
miR-10527	ENSG00000197465	7mer-m8	TargetScan
miR-10527	ENSG00000197555	7mer-m8	TargetScan
miR-10527	ENSG00000197557	7mer-m8	TargetScan
miR-10527	ENSG00000197622	7mer-m8	TargetScan
miR-10527	ENSG00000197702	7mer-m8	TargetScan
miR-10527	ENSG00000197714	7mer-m8	TargetScan
miR-10527	ENSG00000197766	7mer-m8	TargetScan
miR-10527	ENSG00000197863	7mer-m8	TargetScan
miR-10527	ENSG00000197892	7mer-m8	TargetScan
miR-10527	ENSG00000197969	7mer-m8	TargetScan
miR-10527	ENSG00000198039	7mer-m8	TargetScan
miR-10527	ENSG00000198081	7mer-m8	TargetScan
miR-10527	ENSG00000198160	7mer-m8	TargetScan
miR-10527	ENSG00000198205	7mer-m8	TargetScan
miR-10527	ENSG00000198265	7mer-m8	TargetScan
miR-10527	ENSG00000198315	7mer-m8	TargetScan
miR-10527	ENSG00000198380	7mer-m8	TargetScan
miR-10527	ENSG00000198574	7mer-m8	TargetScan
miR-10527	ENSG00000198612	7mer-m8	TargetScan
miR-10527	ENSG00000198633	7mer-m8	TargetScan
miR-10527	ENSG00000198663	7mer-m8	TargetScan
miR-10527	ENSG00000198673	7mer-m8	TargetScan
miR-10527	ENSG00000198690	7mer-m8	TargetScan
miR-10527	ENSG00000198718	7mer-m8	TargetScan
miR-10527	ENSG00000198739	7mer-m8	TargetScan
miR-10527	ENSG00000198740	7mer-m8	TargetScan
miR-10527	ENSG00000198774	7mer-m8	TargetScan
miR-10527	ENSG00000198799	7mer-m8	TargetScan
miR-10527	ENSG00000198805	7mer-m8	TargetScan
miR-10527	ENSG00000198815	7mer-m8	TargetScan

miR-10527	ENSG00000198843	7mer-m8	TargetScan
miR-10527	ENSG00000198890	7mer-m8	TargetScan
miR-10527	ENSG00000203666	7mer-m8	TargetScan
miR-10527	ENSG00000203710	7mer-m8	TargetScan
miR-10527	ENSG00000204103	7mer-m8	TargetScan
miR-10527	ENSG00000204252	7mer-m8	TargetScan
miR-10527	ENSG00000204271	7mer-m8	TargetScan
miR-10527	ENSG00000204392	7mer-m8	TargetScan
miR-10527	ENSG00000204677	7mer-m8	TargetScan
miR-10527	ENSG00000204767	7mer-m8	TargetScan
miR-10527	ENSG00000204954	7mer-m8	TargetScan
miR-10527	ENSG00000205189	7mer-m8	TargetScan
miR-10527	ENSG00000205531	7mer-m8	TargetScan
miR-10527	ENSG00000206127	7mer-m8	TargetScan
miR-10527	ENSG00000206262	7mer-m8	TargetScan
miR-10527	ENSG00000206418	7mer-m8	TargetScan
miR-10527	ENSG00000206503	7mer-m8	TargetScan
miR-10527	ENSG00000206538	7mer-m8	TargetScan
miR-10527	ENSG00000206562	7mer-m8	TargetScan
miR-10527	ENSG00000206579	7mer-m8	TargetScan
miR-10527	ENSG00000212747	7mer-m8	TargetScan
miR-10527	ENSG00000213020	7mer-m8	TargetScan
miR-10527	ENSG00000213186	7mer-m8	TargetScan
miR-10527	ENSG00000213380	7mer-m8	TargetScan
miR-10527	ENSG00000213463	7mer-m8	TargetScan
miR-10527	ENSG00000213923	7mer-m8	TargetScan
miR-10527	ENSG00000214114	7mer-m8	TargetScan
miR-10527	ENSG00000214274	7mer-m8	TargetScan
miR-10527	ENSG00000214435	7mer-m8	TargetScan
miR-10527	ENSG00000214736	7mer-m8	TargetScan
miR-10527	ENSG00000214753	7mer-m8	TargetScan

miR-10527	ENSG00000214756	7mer-m8	TargetScan
miR-10527	ENSG00000215186	7mer-m8	TargetScan
miR-10527	ENSG00000215271	7mer-m8	TargetScan
miR-10527	ENSG00000215784	7mer-m8	TargetScan
miR-10527	ENSG00000217128	7mer-m8	TargetScan
miR-10527	ENSG00000218336	7mer-m8	TargetScan
miR-10527	ENSG00000218739	7mer-m8	TargetScan
miR-10527	ENSG00000221818	7mer-m8	TargetScan
miR-10527	ENSG00000221866	7mer-m8	TargetScan
miR-10527	ENSG00000221867	7mer-m8	TargetScan
miR-10527	ENSG00000221963	7mer-m8	TargetScan
miR-10527	ENSG00000225830	7mer-m8	TargetScan
miR-10527	ENSG00000226887	7mer-m8	TargetScan
miR-10527	ENSG00000228075	7mer-m8	TargetScan
miR-10527	ENSG00000228172	7mer-m8	TargetScan
miR-10527	ENSG00000228696	7mer-m8	TargetScan
miR-10527	ENSG00000232044	7mer-m8	TargetScan
miR-10527	ENSG00000232653	7mer-m8	TargetScan
miR-10527	ENSG00000232810	7mer-m8	TargetScan
miR-10527	ENSG00000235194	7mer-m8	TargetScan
miR-10527	ENSG00000236027	7mer-m8	TargetScan
miR-10527	ENSG00000237136	7mer-m8	TargetScan
miR-10527	ENSG00000240403	7mer-m8	TargetScan
miR-10527	ENSG00000240694	7mer-m8	TargetScan
miR-10527	ENSG00000241489	7mer-m8	TargetScan
miR-10527	ENSG00000243772	7mer-m8	TargetScan
miR-10527	ENSG00000244754	7mer-m8	TargetScan
miR-10527	ENSG00000248538	7mer-m8	TargetScan
miR-10527	ENSG00000248905	7mer-m8	TargetScan
miR-10527	ENSG00000249931	7mer-m8	TargetScan
miR-10527	ENSG00000250423	7mer-m8	TargetScan

miR-10527	ENSG00000251201	7mer-m8	TargetScan
miR-10527	ENSG00000253207	7mer-m8	TargetScan
miR-10527	ENSG00000254004	7mer-m8	TargetScan
miR-10527	ENSG00000255529	7mer-m8	TargetScan
miR-10527	ENSG00000256043	7mer-m8	TargetScan
miR-10527	ENSG00000256087	7mer-m8	TargetScan
miR-10527	ENSG00000257315	7mer-m8	TargetScan
miR-10527	ENSG00000257923	7mer-m8	TargetScan
miR-10527	ENSG00000258405	7mer-m8	TargetScan
miR-10527	ENSG00000258429	7mer-m8	TargetScan
miR-10527	ENSG00000258869	7mer-m8	TargetScan
miR-10527	ENSG00000259363	7mer-m8	TargetScan
miR-10527	ENSG00000260230	7mer-m8	TargetScan
miR-10527	ENSG00000261794	7mer-m8	TargetScan
miR-10527	ENSG00000263513	7mer-m8	TargetScan
miR-10527	ENSG00000272195	7mer-m8	TargetScan
miR-10527	ENSG00000272325	7mer-m8	TargetScan
miR-10527	ENSG00000272617	7mer-m8	TargetScan
miR-10527	ENSG00000273079	7mer-m8	TargetScan
miR-10527	ENSG00000274944	7mer-m8	TargetScan
miR-10527	ENSG00000278828	7mer-m8	TargetScan
miR-10527	ENSG00000285976	7mer-m8	TargetScan
miR-10527	ENSG00000286220	7mer-m8	TargetScan
miR-1244	ENSG00000000003	7mer-m8	TargetScan
miR-1244	ENSG00000003402	7mer-m8	TargetScan
miR-1244	ENSG00000005812	7mer-m8	TargetScan
miR-1244	ENSG00000006007	7mer-m8	TargetScan
miR-1244	ENSG00000007174	7mer-m8	TargetScan
miR-1244	ENSG00000009694	7mer-m8	TargetScan
miR-1244	ENSG00000010244	7mer-m8	TargetScan
miR-1244	ENSG00000011405	7mer-m8	TargetScan

miR-1244	ENSG00000014824	7mer-m8	TargetScan
miR-1244	ENSG00000023734	7mer-m8	TargetScan
miR-1244	ENSG00000024048	7mer-m8	TargetScan
miR-1244	ENSG00000025039	7mer-m8	TargetScan
miR-1244	ENSG00000025423	7mer-m8	TargetScan
miR-1244	ENSG00000029639	7mer-m8	TargetScan
miR-1244	ENSG00000030110	7mer-m8	TargetScan
miR-1244	ENSG00000031823	7mer-m8	TargetScan
miR-1244	ENSG00000033800	7mer-m8	TargetScan
miR-1244	ENSG00000037280	7mer-m8	TargetScan
miR-1244	ENSG00000038210	7mer-m8	TargetScan
miR-1244	ENSG00000044459	7mer-m8	TargetScan
miR-1244	ENSG00000048052	7mer-m8	TargetScan
miR-1244	ENSG00000052802	7mer-m8	TargetScan
miR-1244	ENSG00000053108	7mer-m8	TargetScan
miR-1244	ENSG00000055332	7mer-m8	TargetScan
miR-1244	ENSG00000056291	7mer-m8	TargetScan
miR-1244	ENSG00000057019	7mer-m8	TargetScan
miR-1244	ENSG00000057294	7mer-m8	TargetScan
miR-1244	ENSG00000059728	7mer-m8	TargetScan
miR-1244	ENSG00000060140	7mer-m8	TargetScan
miR-1244	ENSG00000060237	7mer-m8	TargetScan
miR-1244	ENSG00000061676	7mer-m8	TargetScan
miR-1244	ENSG00000066629	7mer-m8	TargetScan
miR-1244	ENSG00000067208	7mer-m8	TargetScan
miR-1244	ENSG00000067560	7mer-m8	TargetScan
miR-1244	ENSG00000068394	7mer-m8	TargetScan
miR-1244	ENSG00000069493	7mer-m8	TargetScan
miR-1244	ENSG00000069869	7mer-m8	TargetScan
miR-1244	ENSG00000070214	7mer-m8	TargetScan
miR-1244	ENSG00000072042	7mer-m8	TargetScan

miR-1244	ENSG00000072121	7mer-m8	TargetScan
miR-1244	ENSG00000072201	7mer-m8	TargetScan
miR-1244	ENSG00000072657	7mer-m8	TargetScan
miR-1244	ENSG00000073008	7mer-m8	TargetScan
miR-1244	ENSG00000073711	7mer-m8	TargetScan
miR-1244	ENSG00000075223	7mer-m8	TargetScan
miR-1244	ENSG00000076554	7mer-m8	TargetScan
miR-1244	ENSG00000078018	7mer-m8	TargetScan
miR-1244	ENSG00000078237	7mer-m8	TargetScan
miR-1244	ENSG00000078596	7mer-m8	TargetScan
miR-1244	ENSG00000081386	7mer-m8	TargetScan
miR-1244	ENSG00000082153	7mer-m8	TargetScan
miR-1244	ENSG00000083067	7mer-m8	TargetScan
miR-1244	ENSG00000083099	7mer-m8	TargetScan
miR-1244	ENSG00000083223	7mer-m8	TargetScan
miR-1244	ENSG00000084652	7mer-m8	TargetScan
miR-1244	ENSG00000086717	7mer-m8	TargetScan
miR-1244	ENSG00000087128	7mer-m8	TargetScan
miR-1244	ENSG00000089041	7mer-m8	TargetScan
miR-1244	ENSG00000089775	7mer-m8	TargetScan
miR-1244	ENSG00000090989	7mer-m8	TargetScan
miR-1244	ENSG00000091157	7mer-m8	TargetScan
miR-1244	ENSG00000099139	7mer-m8	TargetScan
miR-1244	ENSG00000099715	7mer-m8	TargetScan
miR-1244	ENSG00000100077	7mer-m8	TargetScan
miR-1244	ENSG00000100478	7mer-m8	TargetScan
miR-1244	ENSG00000100505	7mer-m8	TargetScan
miR-1244	ENSG00000100528	7mer-m8	TargetScan
miR-1244	ENSG00000100934	7mer-m8	TargetScan
miR-1244	ENSG00000101166	7mer-m8	TargetScan
miR-1244	ENSG00000101265	7mer-m8	TargetScan

miR-1244	ENSG00000101557	7mer-m8	TargetScan
miR-1244	ENSG00000101958	7mer-m8	TargetScan
miR-1244	ENSG00000102158	7mer-m8	TargetScan
miR-1244	ENSG00000102218	7mer-m8	TargetScan
miR-1244	ENSG00000102290	7mer-m8	TargetScan
miR-1244	ENSG00000102393	7mer-m8	TargetScan
miR-1244	ENSG00000102471	7mer-m8	TargetScan
miR-1244	ENSG00000102780	7mer-m8	TargetScan
miR-1244	ENSG00000102804	7mer-m8	TargetScan
miR-1244	ENSG00000102910	7mer-m8	TargetScan
miR-1244	ENSG00000103061	7mer-m8	TargetScan
miR-1244	ENSG00000103404	7mer-m8	TargetScan
miR-1244	ENSG00000104218	7mer-m8	TargetScan
miR-1244	ENSG00000104231	7mer-m8	TargetScan
miR-1244	ENSG00000104327	7mer-m8	TargetScan
miR-1244	ENSG00000105492	7mer-m8	TargetScan
miR-1244	ENSG00000105750	7mer-m8	TargetScan
miR-1244	ENSG00000106261	7mer-m8	TargetScan
miR-1244	ENSG00000106299	7mer-m8	TargetScan
miR-1244	ENSG00000106328	7mer-m8	TargetScan
miR-1244	ENSG00000106366	7mer-m8	TargetScan
miR-1244	ENSG00000106443	7mer-m8	TargetScan
miR-1244	ENSG00000106952	7mer-m8	TargetScan
miR-1244	ENSG00000108219	7mer-m8	TargetScan
miR-1244	ENSG00000109220	7mer-m8	TargetScan
miR-1244	ENSG00000109436	7mer-m8	TargetScan
miR-1244	ENSG00000109572	7mer-m8	TargetScan
miR-1244	ENSG00000109911	7mer-m8	TargetScan
miR-1244	ENSG00000109917	7mer-m8	TargetScan
miR-1244	ENSG00000110048	7mer-m8	TargetScan
miR-1244	ENSG00000110344	7mer-m8	TargetScan

miR-1244	ENSG00000110395	7mer-m8	TargetScan
miR-1244	ENSG00000110987	7mer-m8	TargetScan
miR-1244	ENSG00000111145	7mer-m8	TargetScan
miR-1244	ENSG00000111799	7mer-m8	TargetScan
miR-1244	ENSG00000111911	7mer-m8	TargetScan
miR-1244	ENSG00000112234	7mer-m8	TargetScan
miR-1244	ENSG00000112242	7mer-m8	TargetScan
miR-1244	ENSG00000112309	7mer-m8	TargetScan
miR-1244	ENSG00000112339	7mer-m8	TargetScan
miR-1244	ENSG00000112379	7mer-m8	TargetScan
miR-1244	ENSG00000112499	7mer-m8	TargetScan
miR-1244	ENSG00000113361	7mer-m8	TargetScan
miR-1244	ENSG00000113600	7mer-m8	TargetScan
miR-1244	ENSG00000113615	7mer-m8	TargetScan
miR-1244	ENSG00000113851	7mer-m8	TargetScan
miR-1244	ENSG00000115183	7mer-m8	TargetScan
miR-1244	ENSG00000115524	7mer-m8	TargetScan
miR-1244	ENSG00000115540	7mer-m8	TargetScan
miR-1244	ENSG00000115760	7mer-m8	TargetScan
miR-1244	ENSG00000116544	7mer-m8	TargetScan
miR-1244	ENSG00000117151	7mer-m8	TargetScan
miR-1244	ENSG00000117724	7mer-m8	TargetScan
miR-1244	ENSG00000117758	7mer-m8	TargetScan
miR-1244	ENSG00000118217	7mer-m8	TargetScan
miR-1244	ENSG00000118495	7mer-m8	TargetScan
miR-1244	ENSG00000118514	7mer-m8	TargetScan
miR-1244	ENSG00000119203	7mer-m8	TargetScan
miR-1244	ENSG00000119328	7mer-m8	TargetScan
miR-1244	ENSG00000119673	7mer-m8	TargetScan
miR-1244	ENSG00000119820	7mer-m8	TargetScan
miR-1244	ENSG00000119973	7mer-m8	TargetScan

miR-1244	ENSG00000120907	7mer-m8	TargetScan
miR-1244	ENSG00000120925	7mer-m8	TargetScan
miR-1244	ENSG00000120992	7mer-m8	TargetScan
miR-1244	ENSG00000122435	7mer-m8	TargetScan
miR-1244	ENSG00000122952	7mer-m8	TargetScan
miR-1244	ENSG00000123307	7mer-m8	TargetScan
miR-1244	ENSG00000123594	7mer-m8	TargetScan
miR-1244	ENSG00000124374	7mer-m8	TargetScan
miR-1244	ENSG00000124532	7mer-m8	TargetScan
miR-1244	ENSG00000124596	7mer-m8	TargetScan
miR-1244	ENSG00000124613	7mer-m8	TargetScan
miR-1244	ENSG00000124783	7mer-m8	TargetScan
miR-1244	ENSG00000125285	7mer-m8	TargetScan
miR-1244	ENSG00000125457	7mer-m8	TargetScan
miR-1244	ENSG00000125629	7mer-m8	TargetScan
miR-1244	ENSG00000126070	7mer-m8	TargetScan
miR-1244	ENSG00000126266	7mer-m8	TargetScan
miR-1244	ENSG00000126803	7mer-m8	TargetScan
miR-1244	ENSG00000127920	7mer-m8	TargetScan
miR-1244	ENSG00000128159	7mer-m8	TargetScan
miR-1244	ENSG00000128617	7mer-m8	TargetScan
miR-1244	ENSG00000129691	7mer-m8	TargetScan
miR-1244	ENSG00000129810	7mer-m8	TargetScan
miR-1244	ENSG00000130119	7mer-m8	TargetScan
miR-1244	ENSG00000130695	7mer-m8	TargetScan
miR-1244	ENSG00000131375	7mer-m8	TargetScan
miR-1244	ENSG00000132122	7mer-m8	TargetScan
miR-1244	ENSG00000132254	7mer-m8	TargetScan
miR-1244	ENSG00000132300	7mer-m8	TargetScan
miR-1244	ENSG00000132554	7mer-m8	TargetScan
miR-1244	ENSG00000132964	7mer-m8	TargetScan

miR-1244	ENSG00000132975	7mer-m8	TargetScan
miR-1244	ENSG00000133083	7mer-m8	TargetScan
miR-1244	ENSG00000134153	7mer-m8	TargetScan
miR-1244	ENSG00000134242	7mer-m8	TargetScan
miR-1244	ENSG00000134248	7mer-m8	TargetScan
miR-1244	ENSG00000134294	7mer-m8	TargetScan
miR-1244	ENSG00000134444	7mer-m8	TargetScan
miR-1244	ENSG00000134970	7mer-m8	TargetScan
miR-1244	ENSG00000134987	7mer-m8	TargetScan
miR-1244	ENSG00000135205	7mer-m8	TargetScan
miR-1244	ENSG00000135299	7mer-m8	TargetScan
miR-1244	ENSG00000135387	7mer-m8	TargetScan
miR-1244	ENSG00000135541	7mer-m8	TargetScan
miR-1244	ENSG00000135899	7mer-m8	TargetScan
miR-1244	ENSG00000136021	7mer-m8	TargetScan
miR-1244	ENSG00000136144	7mer-m8	TargetScan
miR-1244	ENSG00000136783	7mer-m8	TargetScan
miR-1244	ENSG00000137033	7mer-m8	TargetScan
miR-1244	ENSG00000137501	7mer-m8	TargetScan
miR-1244	ENSG00000137504	7mer-m8	TargetScan
miR-1244	ENSG00000137727	7mer-m8	TargetScan
miR-1244	ENSG00000137760	7mer-m8	TargetScan
miR-1244	ENSG00000137812	7mer-m8	TargetScan
miR-1244	ENSG00000138081	7mer-m8	TargetScan
miR-1244	ENSG00000138185	7mer-m8	TargetScan
miR-1244	ENSG00000138286	7mer-m8	TargetScan
miR-1244	ENSG00000138347	7mer-m8	TargetScan
miR-1244	ENSG00000138653	7mer-m8	TargetScan
miR-1244	ENSG00000138670	7mer-m8	TargetScan
miR-1244	ENSG00000138767	7mer-m8	TargetScan
miR-1244	ENSG00000138769	7mer-m8	TargetScan

miR-1244	ENSG00000138772	7mer-m8	TargetScan
miR-1244	ENSG00000139133	7mer-m8	TargetScan
miR-1244	ENSG00000139278	7mer-m8	TargetScan
miR-1244	ENSG00000139291	7mer-m8	TargetScan
miR-1244	ENSG00000139971	7mer-m8	TargetScan
miR-1244	ENSG00000140319	7mer-m8	TargetScan
miR-1244	ENSG00000140832	7mer-m8	TargetScan
miR-1244	ENSG00000140987	7mer-m8	TargetScan
miR-1244	ENSG00000141027	7mer-m8	TargetScan
miR-1244	ENSG00000141404	7mer-m8	TargetScan
miR-1244	ENSG00000141627	7mer-m8	TargetScan
miR-1244	ENSG00000141639	7mer-m8	TargetScan
miR-1244	ENSG00000142405	7mer-m8	TargetScan
miR-1244	ENSG00000142599	7mer-m8	TargetScan
miR-1244	ENSG00000143179	7mer-m8	TargetScan
miR-1244	ENSG00000143228	7mer-m8	TargetScan
miR-1244	ENSG00000143322	7mer-m8	TargetScan
miR-1244	ENSG00000144278	7mer-m8	TargetScan
miR-1244	ENSG00000144791	7mer-m8	TargetScan
miR-1244	ENSG00000144848	7mer-m8	TargetScan
miR-1244	ENSG00000145147	7mer-m8	TargetScan
miR-1244	ENSG00000145335	7mer-m8	TargetScan
miR-1244	ENSG00000145431	7mer-m8	TargetScan
miR-1244	ENSG00000145451	7mer-m8	TargetScan
miR-1244	ENSG00000145687	7mer-m8	TargetScan
miR-1244	ENSG00000146587	7mer-m8	TargetScan
miR-1244	ENSG00000146592	7mer-m8	TargetScan
miR-1244	ENSG00000146755	7mer-m8	TargetScan
miR-1244	ENSG00000147010	7mer-m8	TargetScan
miR-1244	ENSG00000147036	7mer-m8	TargetScan
miR-1244	ENSG00000147364	7mer-m8	TargetScan

miR-1244	ENSG00000147852	7mer-m8	TargetScan
miR-1244	ENSG00000147853	7mer-m8	TargetScan
miR-1244	ENSG00000147996	7mer-m8	TargetScan
miR-1244	ENSG00000148219	7mer-m8	TargetScan
miR-1244	ENSG00000148229	7mer-m8	TargetScan
miR-1244	ENSG00000148248	7mer-m8	TargetScan
miR-1244	ENSG00000148498	7mer-m8	TargetScan
miR-1244	ENSG00000148734	7mer-m8	TargetScan
miR-1244	ENSG00000148842	7mer-m8	TargetScan
miR-1244	ENSG00000148942	7mer-m8	TargetScan
miR-1244	ENSG00000149084	7mer-m8	TargetScan
miR-1244	ENSG00000149131	7mer-m8	TargetScan
miR-1244	ENSG00000151962	7mer-m8	TargetScan
miR-1244	ENSG00000152061	7mer-m8	TargetScan
miR-1244	ENSG00000152207	7mer-m8	TargetScan
miR-1244	ENSG00000152256	7mer-m8	TargetScan
miR-1244	ENSG00000152578	7mer-m8	TargetScan
miR-1244	ENSG00000152582	7mer-m8	TargetScan
miR-1244	ENSG00000152749	7mer-m8	TargetScan
miR-1244	ENSG00000152763	7mer-m8	TargetScan
miR-1244	ENSG00000153721	7mer-m8	TargetScan
miR-1244	ENSG00000153989	7mer-m8	TargetScan
miR-1244	ENSG00000154188	7mer-m8	TargetScan
miR-1244	ENSG00000154310	7mer-m8	TargetScan
miR-1244	ENSG00000154518	7mer-m8	TargetScan
miR-1244	ENSG00000154642	7mer-m8	TargetScan
miR-1244	ENSG00000154736	7mer-m8	TargetScan
miR-1244	ENSG00000155792	7mer-m8	TargetScan
miR-1244	ENSG00000155959	7mer-m8	TargetScan
miR-1244	ENSG00000156136	7mer-m8	TargetScan
miR-1244	ENSG00000156140	7mer-m8	TargetScan

miR-1244	ENSG00000156398	7mer-m8	TargetScan
miR-1244	ENSG00000156531	7mer-m8	TargetScan
miR-1244	ENSG00000156675	7mer-m8	TargetScan
miR-1244	ENSG00000157214	7mer-m8	TargetScan
miR-1244	ENSG00000157680	7mer-m8	TargetScan
miR-1244	ENSG00000157890	7mer-m8	TargetScan
miR-1244	ENSG00000158201	7mer-m8	TargetScan
miR-1244	ENSG00000158528	7mer-m8	TargetScan
miR-1244	ENSG00000158813	7mer-m8	TargetScan
miR-1244	ENSG00000161649	7mer-m8	TargetScan
miR-1244	ENSG00000161654	7mer-m8	TargetScan
miR-1244	ENSG00000161921	7mer-m8	TargetScan
miR-1244	ENSG00000162129	7mer-m8	TargetScan
miR-1244	ENSG00000162241	7mer-m8	TargetScan
miR-1244	ENSG00000162441	7mer-m8	TargetScan
miR-1244	ENSG00000162599	7mer-m8	TargetScan
miR-1244	ENSG00000162618	7mer-m8	TargetScan
miR-1244	ENSG00000162706	7mer-m8	TargetScan
miR-1244	ENSG00000162814	7mer-m8	TargetScan
miR-1244	ENSG00000162946	7mer-m8	TargetScan
miR-1244	ENSG00000163041	7mer-m8	TargetScan
miR-1244	ENSG00000163154	7mer-m8	TargetScan
miR-1244	ENSG00000163288	7mer-m8	TargetScan
miR-1244	ENSG00000163291	7mer-m8	TargetScan
miR-1244	ENSG00000163444	7mer-m8	TargetScan
miR-1244	ENSG00000163596	7mer-m8	TargetScan
miR-1244	ENSG00000163673	7mer-m8	TargetScan
miR-1244	ENSG00000163803	7mer-m8	TargetScan
miR-1244	ENSG00000164093	7mer-m8	TargetScan
miR-1244	ENSG00000164114	7mer-m8	TargetScan
miR-1244	ENSG00000164125	7mer-m8	TargetScan

miR-1244	ENSG00000164168	7mer-m8	TargetScan
miR-1244	ENSG00000164172	7mer-m8	TargetScan
miR-1244	ENSG00000164188	7mer-m8	TargetScan
miR-1244	ENSG00000164252	7mer-m8	TargetScan
miR-1244	ENSG00000164342	7mer-m8	TargetScan
miR-1244	ENSG00000164764	7mer-m8	TargetScan
miR-1244	ENSG00000164815	7mer-m8	TargetScan
miR-1244	ENSG00000164867	7mer-m8	TargetScan
miR-1244	ENSG00000164902	7mer-m8	TargetScan
miR-1244	ENSG00000165283	7mer-m8	TargetScan
miR-1244	ENSG00000165309	7mer-m8	TargetScan
miR-1244	ENSG00000165338	7mer-m8	TargetScan
miR-1244	ENSG00000165474	7mer-m8	TargetScan
miR-1244	ENSG00000165506	7mer-m8	TargetScan
miR-1244	ENSG00000165732	7mer-m8	TargetScan
miR-1244	ENSG00000165775	7mer-m8	TargetScan
miR-1244	ENSG00000165997	7mer-m8	TargetScan
miR-1244	ENSG00000166037	7mer-m8	TargetScan
miR-1244	ENSG00000166148	7mer-m8	TargetScan
miR-1244	ENSG00000166398	7mer-m8	TargetScan
miR-1244	ENSG00000166446	7mer-m8	TargetScan
miR-1244	ENSG00000166454	7mer-m8	TargetScan
miR-1244	ENSG00000166479	7mer-m8	TargetScan
miR-1244	ENSG00000166575	7mer-m8	TargetScan
miR-1244	ENSG00000166845	7mer-m8	TargetScan
miR-1244	ENSG00000167005	7mer-m8	TargetScan
miR-1244	ENSG00000167191	7mer-m8	TargetScan
miR-1244	ENSG00000167202	7mer-m8	TargetScan
miR-1244	ENSG00000167384	7mer-m8	TargetScan
miR-1244	ENSG00000167526	7mer-m8	TargetScan
miR-1244	ENSG00000167548	7mer-m8	TargetScan

miR-1244	ENSG00000167613	7mer-m8	TargetScan
miR-1244	ENSG00000168303	7mer-m8	TargetScan
miR-1244	ENSG00000168453	7mer-m8	TargetScan
miR-1244	ENSG00000168610	7mer-m8	TargetScan
miR-1244	ENSG00000168939	7mer-m8	TargetScan
miR-1244	ENSG00000168939	7mer-m8	TargetScan
miR-1244	ENSG00000168944	7mer-m8	TargetScan
miR-1244	ENSG00000169122	7mer-m8	TargetScan
miR-1244	ENSG00000169504	7mer-m8	TargetScan
miR-1244	ENSG00000169860	7mer-m8	TargetScan
miR-1244	ENSG00000169967	7mer-m8	TargetScan
miR-1244	ENSG00000169991	7mer-m8	TargetScan
miR-1244	ENSG00000170191	7mer-m8	TargetScan
miR-1244	ENSG00000170312	7mer-m8	TargetScan
miR-1244	ENSG00000170340	7mer-m8	TargetScan
miR-1244	ENSG00000170456	7mer-m8	TargetScan
miR-1244	ENSG00000170502	7mer-m8	TargetScan
miR-1244	ENSG00000170542	7mer-m8	TargetScan
miR-1244	ENSG00000170832	7mer-m8	TargetScan
miR-1244	ENSG00000171368	7mer-m8	TargetScan
miR-1244	ENSG00000171843	7mer-m8	TargetScan
miR-1244	ENSG00000171862	7mer-m8	TargetScan
miR-1244	ENSG00000172209	7mer-m8	TargetScan
miR-1244	ENSG00000172339	7mer-m8	TargetScan
miR-1244	ENSG00000172380	7mer-m8	TargetScan
miR-1244	ENSG00000172466	7mer-m8	TargetScan
miR-1244	ENSG00000172493	7mer-m8	TargetScan
miR-1244	ENSG00000172785	7mer-m8	TargetScan
miR-1244	ENSG00000172817	7mer-m8	TargetScan
miR-1244	ENSG00000173207	7mer-m8	TargetScan
miR-1244	ENSG00000173273	7mer-m8	TargetScan

miR-1244	ENSG00000173285	7mer-m8	TargetScan
miR-1244	ENSG00000173334	7mer-m8	TargetScan
miR-1244	ENSG00000173451	7mer-m8	TargetScan
miR-1244	ENSG00000173517	7mer-m8	TargetScan
miR-1244	ENSG00000173744	7mer-m8	TargetScan
miR-1244	ENSG00000173890	7mer-m8	TargetScan
miR-1244	ENSG00000174013	7mer-m8	TargetScan
miR-1244	ENSG00000174016	7mer-m8	TargetScan
miR-1244	ENSG00000174429	7mer-m8	TargetScan
miR-1244	ENSG00000175170	7mer-m8	TargetScan
miR-1244	ENSG00000175189	7mer-m8	TargetScan
miR-1244	ENSG00000175305	7mer-m8	TargetScan
miR-1244	ENSG00000175387	7mer-m8	TargetScan
miR-1244	ENSG00000175390	7mer-m8	TargetScan
miR-1244	ENSG00000175471	7mer-m8	TargetScan
miR-1244	ENSG00000175548	7mer-m8	TargetScan
miR-1244	ENSG00000176049	7mer-m8	TargetScan
miR-1244	ENSG00000176463	7mer-m8	TargetScan
miR-1244	ENSG00000177324	7mer-m8	TargetScan
miR-1244	ENSG00000177570	7mer-m8	TargetScan
miR-1244	ENSG00000177689	7mer-m8	TargetScan
miR-1244	ENSG00000178201	7mer-m8	TargetScan
miR-1244	ENSG00000178338	7mer-m8	TargetScan
miR-1244	ENSG00000178562	7mer-m8	TargetScan
miR-1244	ENSG00000178795	7mer-m8	TargetScan
miR-1244	ENSG00000179028	7mer-m8	TargetScan
miR-1244	ENSG00000179387	7mer-m8	TargetScan
miR-1244	ENSG00000179915	7mer-m8	TargetScan
miR-1244	ENSG00000180287	7mer-m8	TargetScan
miR-1244	ENSG00000181704	7mer-m8	TargetScan
miR-1244	ENSG00000181722	7mer-m8	TargetScan

miR-1244	ENSG00000182010	7mer-m8	TargetScan
miR-1244	ENSG00000182261	7mer-m8	TargetScan
miR-1244	ENSG00000182621	7mer-m8	TargetScan
miR-1244	ENSG00000182670	7mer-m8	TargetScan
miR-1244	ENSG00000182776	7mer-m8	TargetScan
miR-1244	ENSG00000182957	7mer-m8	TargetScan
miR-1244	ENSG00000183230	7mer-m8	TargetScan
miR-1244	ENSG00000183530	7mer-m8	TargetScan
miR-1244	ENSG00000183662	7mer-m8	TargetScan
miR-1244	ENSG00000183775	7mer-m8	TargetScan
miR-1244	ENSG00000183814	7mer-m8	TargetScan
miR-1244	ENSG00000183831	7mer-m8	TargetScan
miR-1244	ENSG00000184743	7mer-m8	TargetScan
miR-1244	ENSG00000184939	7mer-m8	TargetScan
miR-1244	ENSG00000185010	7mer-m8	TargetScan
miR-1244	ENSG00000185219	7mer-m8	TargetScan
miR-1244	ENSG00000185621	7mer-m8	TargetScan
miR-1244	ENSG00000185774	7mer-m8	TargetScan
miR-1244	ENSG00000185829	7mer-m8	TargetScan
miR-1244	ENSG00000186009	7mer-m8	TargetScan
miR-1244	ENSG00000186260	7mer-m8	TargetScan
miR-1244	ENSG00000186318	7mer-m8	TargetScan
miR-1244	ENSG00000186628	7mer-m8	TargetScan
miR-1244	ENSG00000186714	7mer-m8	TargetScan
miR-1244	ENSG00000186814	7mer-m8	TargetScan
miR-1244	ENSG00000187753	7mer-m8	TargetScan
miR-1244	ENSG00000187772	7mer-m8	TargetScan
miR-1244	ENSG00000187866	7mer-m8	TargetScan
miR-1244	ENSG00000188042	7mer-m8	TargetScan
miR-1244	ENSG00000188133	7mer-m8	TargetScan
miR-1244	ENSG00000188211	7mer-m8	TargetScan

miR-1244	ENSG00000188517	7mer-m8	TargetScan
miR-1244	ENSG00000188848	7mer-m8	TargetScan
miR-1244	ENSG00000196109	7mer-m8	TargetScan
miR-1244	ENSG00000196233	7mer-m8	TargetScan
miR-1244	ENSG00000196458	7mer-m8	TargetScan
miR-1244	ENSG00000196549	7mer-m8	TargetScan
miR-1244	ENSG00000196693	7mer-m8	TargetScan
miR-1244	ENSG00000196730	7mer-m8	TargetScan
miR-1244	ENSG00000197044	7mer-m8	TargetScan
miR-1244	ENSG00000197124	7mer-m8	TargetScan
miR-1244	ENSG00000197312	7mer-m8	TargetScan
miR-1244	ENSG00000197619	7mer-m8	TargetScan
miR-1244	ENSG00000197714	7mer-m8	TargetScan
miR-1244	ENSG00000197779	7mer-m8	TargetScan
miR-1244	ENSG00000197872	7mer-m8	TargetScan
miR-1244	ENSG00000197885	7mer-m8	TargetScan
miR-1244	ENSG00000198105	7mer-m8	TargetScan
miR-1244	ENSG00000198162	7mer-m8	TargetScan
miR-1244	ENSG00000198168	7mer-m8	TargetScan
miR-1244	ENSG00000198590	7mer-m8	TargetScan
miR-1244	ENSG00000198612	7mer-m8	TargetScan
miR-1244	ENSG00000198677	7mer-m8	TargetScan
miR-1244	ENSG00000198843	7mer-m8	TargetScan
miR-1244	ENSG00000198846	7mer-m8	TargetScan
miR-1244	ENSG00000198874	7mer-m8	TargetScan
miR-1244	ENSG00000198890	7mer-m8	TargetScan
miR-1244	ENSG00000204176	7mer-m8	TargetScan
miR-1244	ENSG00000204304	7mer-m8	TargetScan
miR-1244	ENSG00000204655	7mer-m8	TargetScan
miR-1244	ENSG00000204767	7mer-m8	TargetScan
miR-1244	ENSG00000205045	7mer-m8	TargetScan

miR-1244	ENSG00000205138	7mer-m8	TargetScan
miR-1244	ENSG00000205213	7mer-m8	TargetScan
miR-1244	ENSG00000206432	7mer-m8	TargetScan
miR-1244	ENSG00000206557	7mer-m8	TargetScan
miR-1244	ENSG00000213047	7mer-m8	TargetScan
miR-1244	ENSG00000214113	7mer-m8	TargetScan
miR-1244	ENSG00000222009	7mer-m8	TargetScan
miR-1244	ENSG00000224470	7mer-m8	TargetScan
miR-1244	ENSG00000225526	7mer-m8	TargetScan
miR-1244	ENSG00000228696	7mer-m8	TargetScan
miR-1244	ENSG00000230989	7mer-m8	TargetScan
miR-1244	ENSG00000239900	7mer-m8	TargetScan
miR-1244	ENSG00000244734	7mer-m8	TargetScan
miR-1244	ENSG00000246705	7mer-m8	TargetScan
miR-1244	ENSG00000249139	7mer-m8	TargetScan
miR-1244	ENSG00000254004	7mer-m8	TargetScan
miR-1244	ENSG00000256043	7mer-m8	TargetScan
miR-1244	ENSG00000256294	7mer-m8	TargetScan
miR-1244	ENSG00000258708	7mer-m8	TargetScan
miR-1244	ENSG00000267260	7mer-m8	TargetScan
miR-1244	ENSG00000269067	7mer-m8	TargetScan
miR-1244	ENSG00000270757	7mer-m8	TargetScan
miR-3654	ENSG00000011275	7mer-m8	TargetScan
miR-3654	ENSG00000011465	7mer-m8	TargetScan
miR-3654	ENSG00000013275	7mer-m8	TargetScan
miR-3654	ENSG00000025800	7mer-m8	TargetScan
miR-3654	ENSG00000031081	7mer-m8	TargetScan
miR-3654	ENSG00000046604	7mer-m8	TargetScan
miR-3654	ENSG00000047849	7mer-m8	TargetScan
miR-3654	ENSG00000057657	7mer-m8	TargetScan
miR-3654	ENSG00000064703	7mer-m8	TargetScan

miR-3654	ENSG00000066294	7mer-m8	TargetScan
miR-3654	ENSG00000067900	7mer-m8	TargetScan
miR-3654	ENSG00000068024	7mer-m8	TargetScan
miR-3654	ENSG00000068394	7mer-m8	TargetScan
miR-3654	ENSG00000072364	7mer-m8	TargetScan
miR-3654	ENSG00000072803	7mer-m8	TargetScan
miR-3654	ENSG00000073584	7mer-m8	TargetScan
miR-3654	ENSG00000074410	7mer-m8	TargetScan
miR-3654	ENSG00000078589	7mer-m8	TargetScan
miR-3654	ENSG00000081177	7mer-m8	TargetScan
miR-3654	ENSG00000081760	7mer-m8	TargetScan
miR-3654	ENSG00000081985	7mer-m8	TargetScan
miR-3654	ENSG00000082175	7mer-m8	TargetScan
miR-3654	ENSG00000083099	7mer-m8	TargetScan
miR-3654	ENSG00000085831	7mer-m8	TargetScan
miR-3654	ENSG00000088543	7mer-m8	TargetScan
miR-3654	ENSG00000089101	7mer-m8	TargetScan
miR-3654	ENSG00000091986	7mer-m8	TargetScan
miR-3654	ENSG00000092847	7mer-m8	TargetScan
miR-3654	ENSG00000100030	7mer-m8	TargetScan
miR-3654	ENSG00000100380	7mer-m8	TargetScan
miR-3654	ENSG00000100665	7mer-m8	TargetScan
miR-3654	ENSG00000100815	7mer-m8	TargetScan
miR-3654	ENSG00000101384	7mer-m8	TargetScan
miR-3654	ENSG00000101856	7mer-m8	TargetScan
miR-3654	ENSG00000102096	7mer-m8	TargetScan
miR-3654	ENSG00000102466	7mer-m8	TargetScan
miR-3654	ENSG00000102908	7mer-m8	TargetScan
miR-3654	ENSG00000103061	7mer-m8	TargetScan
miR-3654	ENSG00000103160	7mer-m8	TargetScan
miR-3654	ENSG00000103888	7mer-m8	TargetScan

miR-3654	ENSG00000104142	7mer-m8	TargetScan
miR-3654	ENSG00000104219	7mer-m8	TargetScan
miR-3654	ENSG00000104689	7mer-m8	TargetScan
miR-3654	ENSG00000104974	7mer-m8	TargetScan
miR-3654	ENSG00000105085	7mer-m8	TargetScan
miR-3654	ENSG00000105197	7mer-m8	TargetScan
miR-3654	ENSG00000105204	7mer-m8	TargetScan
miR-3654	ENSG00000106258	7mer-m8	TargetScan
miR-3654	ENSG00000106772	7mer-m8	TargetScan
miR-3654	ENSG00000107362	7mer-m8	TargetScan
miR-3654	ENSG00000107864	7mer-m8	TargetScan
miR-3654	ENSG00000107897	7mer-m8	TargetScan
miR-3654	ENSG00000108107	7mer-m8	TargetScan
miR-3654	ENSG00000108375	7mer-m8	TargetScan
miR-3654	ENSG00000109158	7mer-m8	TargetScan
miR-3654	ENSG00000109265	7mer-m8	TargetScan
miR-3654	ENSG00000109787	7mer-m8	TargetScan
miR-3654	ENSG00000110090	7mer-m8	TargetScan
miR-3654	ENSG00000110218	7mer-m8	TargetScan
miR-3654	ENSG00000111371	7mer-m8	TargetScan
miR-3654	ENSG00000111481	7mer-m8	TargetScan
miR-3654	ENSG00000111530	7mer-m8	TargetScan
miR-3654	ENSG00000111665	7mer-m8	TargetScan
miR-3654	ENSG00000112697	7mer-m8	TargetScan
miR-3654	ENSG00000113119	7mer-m8	TargetScan
miR-3654	ENSG00000113300	7mer-m8	TargetScan
miR-3654	ENSG00000113318	7mer-m8	TargetScan
miR-3654	ENSG00000113494	7mer-m8	TargetScan
miR-3654	ENSG00000113719	7mer-m8	TargetScan
miR-3654	ENSG00000113812	7mer-m8	TargetScan
miR-3654	ENSG00000114331	7mer-m8	TargetScan

miR-3654	ENSG00000114503	7mer-m8	TargetScan
miR-3654	ENSG00000114544	7mer-m8	TargetScan
miR-3654	ENSG00000115306	7mer-m8	TargetScan
miR-3654	ENSG00000115353	7mer-m8	TargetScan
miR-3654	ENSG00000115421	7mer-m8	TargetScan
miR-3654	ENSG00000115507	7mer-m8	TargetScan
miR-3654	ENSG00000115896	7mer-m8	TargetScan
miR-3654	ENSG00000115919	7mer-m8	TargetScan
miR-3654	ENSG00000115977	7mer-m8	TargetScan
miR-3654	ENSG00000117115	7mer-m8	TargetScan
miR-3654	ENSG00000117151	7mer-m8	TargetScan
miR-3654	ENSG00000117155	7mer-m8	TargetScan
miR-3654	ENSG00000117280	7mer-m8	TargetScan
miR-3654	ENSG00000117751	7mer-m8	TargetScan
miR-3654	ENSG00000118217	7mer-m8	TargetScan
miR-3654	ENSG00000118777	7mer-m8	TargetScan
miR-3654	ENSG00000119655	7mer-m8	TargetScan
miR-3654	ENSG00000119661	7mer-m8	TargetScan
miR-3654	ENSG00000119927	7mer-m8	TargetScan
miR-3654	ENSG00000120458	7mer-m8	TargetScan
miR-3654	ENSG00000120549	7mer-m8	TargetScan
miR-3654	ENSG00000121454	7mer-m8	TargetScan
miR-3654	ENSG00000121671	7mer-m8	TargetScan
miR-3654	ENSG00000121964	7mer-m8	TargetScan
miR-3654	ENSG00000122254	7mer-m8	TargetScan
miR-3654	ENSG00000122490	7mer-m8	TargetScan
miR-3654	ENSG00000122642	7mer-m8	TargetScan
miR-3654	ENSG00000122986	7mer-m8	TargetScan
miR-3654	ENSG00000123353	7mer-m8	TargetScan
miR-3654	ENSG00000123739	7mer-m8	TargetScan
miR-3654	ENSG00000124155	7mer-m8	TargetScan

miR-3654	ENSG00000124641	7mer-m8	TargetScan
miR-3654	ENSG00000125037	7mer-m8	TargetScan
miR-3654	ENSG00000125885	7mer-m8	TargetScan
miR-3654	ENSG00000127337	7mer-m8	TargetScan
miR-3654	ENSG00000128641	7mer-m8	TargetScan
miR-3654	ENSG00000128944	7mer-m8	TargetScan
miR-3654	ENSG00000130338	7mer-m8	TargetScan
miR-3654	ENSG00000130958	7mer-m8	TargetScan
miR-3654	ENSG00000132549	7mer-m8	TargetScan
miR-3654	ENSG00000132768	7mer-m8	TargetScan
miR-3654	ENSG00000132879	7mer-m8	TargetScan
miR-3654	ENSG00000132964	7mer-m8	TargetScan
miR-3654	ENSG00000133302	7mer-m8	TargetScan
miR-3654	ENSG00000133958	7mer-m8	TargetScan
miR-3654	ENSG00000134108	7mer-m8	TargetScan
miR-3654	ENSG00000134352	7mer-m8	TargetScan
miR-3654	ENSG00000134759	7mer-m8	TargetScan
miR-3654	ENSG00000134852	7mer-m8	TargetScan
miR-3654	ENSG00000135870	7mer-m8	TargetScan
miR-3654	ENSG00000135999	7mer-m8	TargetScan
miR-3654	ENSG00000136104	7mer-m8	TargetScan
miR-3654	ENSG00000136381	7mer-m8	TargetScan
miR-3654	ENSG00000136824	7mer-m8	TargetScan
miR-3654	ENSG00000137692	7mer-m8	TargetScan
miR-3654	ENSG00000137727	7mer-m8	TargetScan
miR-3654	ENSG00000137947	7mer-m8	TargetScan
miR-3654	ENSG00000138593	7mer-m8	TargetScan
miR-3654	ENSG00000138670	7mer-m8	TargetScan
miR-3654	ENSG00000139437	7mer-m8	TargetScan
miR-3654	ENSG00000139496	7mer-m8	TargetScan
miR-3654	ENSG00000140090	7mer-m8	TargetScan

miR-3654	ENSG00000140391	7mer-m8	TargetScan
miR-3654	ENSG00000140526	7mer-m8	TargetScan
miR-3654	ENSG00000141068	7mer-m8	TargetScan
miR-3654	ENSG00000141127	7mer-m8	TargetScan
miR-3654	ENSG00000141378	7mer-m8	TargetScan
miR-3654	ENSG00000141449	7mer-m8	TargetScan
miR-3654	ENSG00000143106	7mer-m8	TargetScan
miR-3654	ENSG00000143190	7mer-m8	TargetScan
miR-3654	ENSG00000143198	7mer-m8	TargetScan
miR-3654	ENSG00000143457	7mer-m8	TargetScan
miR-3654	ENSG00000143536	7mer-m8	TargetScan
miR-3654	ENSG00000143622	7mer-m8	TargetScan
miR-3654	ENSG00000143727	7mer-m8	TargetScan
miR-3654	ENSG00000144535	7mer-m8	TargetScan
miR-3654	ENSG00000144848	7mer-m8	TargetScan
miR-3654	ENSG00000145779	7mer-m8	TargetScan
miR-3654	ENSG00000145782	7mer-m8	TargetScan
miR-3654	ENSG00000145907	7mer-m8	TargetScan
miR-3654	ENSG00000146278	7mer-m8	TargetScan
miR-3654	ENSG00000146352	7mer-m8	TargetScan
miR-3654	ENSG00000146574	7mer-m8	TargetScan
miR-3654	ENSG00000146729	7mer-m8	TargetScan
miR-3654	ENSG00000146950	7mer-m8	TargetScan
miR-3654	ENSG00000147113	7mer-m8	TargetScan
miR-3654	ENSG00000147121	7mer-m8	TargetScan
miR-3654	ENSG00000147127	7mer-m8	TargetScan
miR-3654	ENSG00000147234	7mer-m8	TargetScan
miR-3654	ENSG00000147588	7mer-m8	TargetScan
miR-3654	ENSG00000148200	7mer-m8	TargetScan
miR-3654	ENSG00000148688	7mer-m8	TargetScan
miR-3654	ENSG00000149927	7mer-m8	TargetScan

miR-3654	ENSG00000149972	7mer-m8	TargetScan
miR-3654	ENSG00000151117	7mer-m8	TargetScan
miR-3654	ENSG00000152127	7mer-m8	TargetScan
miR-3654	ENSG00000152402	7mer-m8	TargetScan
miR-3654	ENSG00000152443	7mer-m8	TargetScan
miR-3654	ENSG00000153391	7mer-m8	TargetScan
miR-3654	ENSG00000153936	7mer-m8	TargetScan
miR-3654	ENSG00000153944	7mer-m8	TargetScan
miR-3654	ENSG00000154080	7mer-m8	TargetScan
miR-3654	ENSG00000154222	7mer-m8	TargetScan
miR-3654	ENSG00000154310	7mer-m8	TargetScan
miR-3654	ENSG00000155096	7mer-m8	TargetScan
miR-3654	ENSG00000155158	7mer-m8	TargetScan
miR-3654	ENSG00000156486	7mer-m8	TargetScan
miR-3654	ENSG00000157388	7mer-m8	TargetScan
miR-3654	ENSG00000158480	7mer-m8	TargetScan
miR-3654	ENSG00000159958	7mer-m8	TargetScan
miR-3654	ENSG00000160471	7mer-m8	TargetScan
miR-3654	ENSG00000162733	7mer-m8	TargetScan
miR-3654	ENSG00000163013	7mer-m8	TargetScan
miR-3654	ENSG00000163646	7mer-m8	TargetScan
miR-3654	ENSG00000163873	7mer-m8	TargetScan
miR-3654	ENSG00000163909	7mer-m8	TargetScan
miR-3654	ENSG00000164161	7mer-m8	TargetScan
miR-3654	ENSG00000164167	7mer-m8	TargetScan
miR-3654	ENSG00000164484	7mer-m8	TargetScan
miR-3654	ENSG00000164983	7mer-m8	TargetScan
miR-3654	ENSG00000165152	7mer-m8	TargetScan
miR-3654	ENSG00000165185	7mer-m8	TargetScan
miR-3654	ENSG00000165209	7mer-m8	TargetScan
miR-3654	ENSG00000165416	7mer-m8	TargetScan

miR-3654	ENSG00000165572	7mer-m8	TargetScan
miR-3654	ENSG00000165806	7mer-m8	TargetScan
miR-3654	ENSG00000166123	7mer-m8	TargetScan
miR-3654	ENSG00000166147	7mer-m8	TargetScan
miR-3654	ENSG00000166148	7mer-m8	TargetScan
miR-3654	ENSG00000166167	7mer-m8	TargetScan
miR-3654	ENSG00000166225	7mer-m8	TargetScan
miR-3654	ENSG00000167378	7mer-m8	TargetScan
miR-3654	ENSG00000167797	7mer-m8	TargetScan
miR-3654	ENSG00000167904	7mer-m8	TargetScan
miR-3654	ENSG00000168159	7mer-m8	TargetScan
miR-3654	ENSG00000168228	7mer-m8	TargetScan
miR-3654	ENSG00000169045	7mer-m8	TargetScan
miR-3654	ENSG00000169067	7mer-m8	TargetScan
miR-3654	ENSG00000170006	7mer-m8	TargetScan
miR-3654	ENSG00000170579	7mer-m8	TargetScan
miR-3654	ENSG00000171759	7mer-m8	TargetScan
miR-3654	ENSG00000172006	7mer-m8	TargetScan
miR-3654	ENSG00000172466	7mer-m8	TargetScan
miR-3654	ENSG00000172663	7mer-m8	TargetScan
miR-3654	ENSG00000172795	7mer-m8	TargetScan
miR-3654	ENSG00000172893	7mer-m8	TargetScan
miR-3654	ENSG00000172943	7mer-m8	TargetScan
miR-3654	ENSG00000173166	7mer-m8	TargetScan
miR-3654	ENSG00000173218	7mer-m8	TargetScan
miR-3654	ENSG00000173349	7mer-m8	TargetScan
miR-3654	ENSG00000173530	7mer-m8	TargetScan
miR-3654	ENSG00000173597	7mer-m8	TargetScan
miR-3654	ENSG00000174579	7mer-m8	TargetScan
miR-3654	ENSG00000174749	7mer-m8	TargetScan
miR-3654	ENSG00000174945	7mer-m8	TargetScan

miR-3654	ENSG00000174953	7mer-m8	TargetScan
miR-3654	ENSG00000175193	7mer-m8	TargetScan
miR-3654	ENSG00000175387	7mer-m8	TargetScan
miR-3654	ENSG00000175414	7mer-m8	TargetScan
miR-3654	ENSG00000175538	7mer-m8	TargetScan
miR-3654	ENSG00000175868	7mer-m8	TargetScan
miR-3654	ENSG00000176142	7mer-m8	TargetScan
miR-3654	ENSG00000176593	7mer-m8	TargetScan
miR-3654	ENSG00000176714	7mer-m8	TargetScan
miR-3654	ENSG00000177733	7mer-m8	TargetScan
miR-3654	ENSG00000178105	7mer-m8	TargetScan
miR-3654	ENSG00000178568	7mer-m8	TargetScan
miR-3654	ENSG00000180902	7mer-m8	TargetScan
miR-3654	ENSG00000180938	7mer-m8	TargetScan
miR-3654	ENSG00000181378	7mer-m8	TargetScan
miR-3654	ENSG00000181982	7mer-m8	TargetScan
miR-3654	ENSG00000182831	7mer-m8	TargetScan
miR-3654	ENSG00000183044	7mer-m8	TargetScan
miR-3654	ENSG00000183137	7mer-m8	TargetScan
miR-3654	ENSG00000183762	7mer-m8	TargetScan
miR-3654	ENSG00000184465	7mer-m8	TargetScan
miR-3654	ENSG00000186088	7mer-m8	TargetScan
miR-3654	ENSG00000186431	7mer-m8	TargetScan
miR-3654	ENSG00000186479	7mer-m8	TargetScan
miR-3654	ENSG00000186814	7mer-m8	TargetScan
miR-3654	ENSG00000187097	7mer-m8	TargetScan
miR-3654	ENSG00000187325	7mer-m8	TargetScan
miR-3654	ENSG00000188177	7mer-m8	TargetScan
miR-3654	ENSG00000188211	7mer-m8	TargetScan
miR-3654	ENSG00000188266	7mer-m8	TargetScan
miR-3654	ENSG00000188729	7mer-m8	TargetScan

miR-3654	ENSG00000188848	7mer-m8	TargetScan
miR-3654	ENSG00000189007	7mer-m8	TargetScan
miR-3654	ENSG00000196090	7mer-m8	TargetScan
miR-3654	ENSG00000196150	7mer-m8	TargetScan
miR-3654	ENSG00000196167	7mer-m8	TargetScan
miR-3654	ENSG00000196678	7mer-m8	TargetScan
miR-3654	ENSG00000196735	7mer-m8	TargetScan
miR-3654	ENSG00000196747	7mer-m8	TargetScan
miR-3654	ENSG00000196867	7mer-m8	TargetScan
miR-3654	ENSG00000196876	7mer-m8	TargetScan
miR-3654	ENSG00000196914	7mer-m8	TargetScan
miR-3654	ENSG00000197157	7mer-m8	TargetScan
miR-3654	ENSG00000197891	7mer-m8	TargetScan
miR-3654	ENSG00000198252	7mer-m8	TargetScan
miR-3654	ENSG00000198455	7mer-m8	TargetScan
miR-3654	ENSG00000198612	7mer-m8	TargetScan
miR-3654	ENSG00000203667	7mer-m8	TargetScan
miR-3654	ENSG00000204070	7mer-m8	TargetScan
miR-3654	ENSG00000204323	7mer-m8	TargetScan
miR-3654	ENSG00000204978	7mer-m8	TargetScan
miR-3654	ENSG00000205730	7mer-m8	TargetScan
miR-3654	ENSG00000205927	7mer-m8	TargetScan
miR-3654	ENSG00000213064	7mer-m8	TargetScan
miR-3654	ENSG00000213995	7mer-m8	TargetScan
miR-3654	ENSG00000237541	7mer-m8	TargetScan
miR-3654	ENSG00000241839	7mer-m8	TargetScan
miR-3654	ENSG00000250305	7mer-m8	TargetScan
miR-3654	ENSG00000254726	7mer-m8	TargetScan
miR-3654	ENSG00000255552	7mer-m8	TargetScan
miR-3654	ENSG00000269155	7mer-m8	TargetScan
miR-4426	ENSG00000000457	7mer-m8	TargetScan

miR-4426	ENSG00000002587	7mer-m8	TargetScan
miR-4426	ENSG00000003402	7mer-m8	TargetScan
miR-4426	ENSG00000005238	7mer-m8	TargetScan
miR-4426	ENSG00000005436	7mer-m8	TargetScan
miR-4426	ENSG00000006747	7mer-m8	TargetScan
miR-4426	ENSG00000008196	7mer-m8	TargetScan
miR-4426	ENSG00000010539	7mer-m8	TargetScan
miR-4426	ENSG00000011405	7mer-m8	TargetScan
miR-4426	ENSG00000012061	7mer-m8	TargetScan
miR-4426	ENSG00000013293	7mer-m8	TargetScan
miR-4426	ENSG00000018869	7mer-m8	TargetScan
miR-4426	ENSG00000019505	7mer-m8	TargetScan
miR-4426	ENSG00000020256	7mer-m8	TargetScan
miR-4426	ENSG00000020426	7mer-m8	TargetScan
miR-4426	ENSG00000024048	7mer-m8	TargetScan
miR-4426	ENSG00000026950	7mer-m8	TargetScan
miR-4426	ENSG00000029993	7mer-m8	TargetScan
miR-4426	ENSG00000034677	7mer-m8	TargetScan
miR-4426	ENSG00000036828	7mer-m8	TargetScan
miR-4426	ENSG00000037637	7mer-m8	TargetScan
miR-4426	ENSG00000039139	7mer-m8	TargetScan
miR-4426	ENSG00000040341	7mer-m8	TargetScan
miR-4426	ENSG00000040731	7mer-m8	TargetScan
miR-4426	ENSG00000042832	7mer-m8	TargetScan
miR-4426	ENSG00000044574	7mer-m8	TargetScan
miR-4426	ENSG00000047597	7mer-m8	TargetScan
miR-4426	ENSG00000047662	7mer-m8	TargetScan
miR-4426	ENSG00000048471	7mer-m8	TargetScan
miR-4426	ENSG00000049246	7mer-m8	TargetScan
miR-4426	ENSG00000049247	7mer-m8	TargetScan
miR-4426	ENSG00000049618	7mer-m8	TargetScan

miR-4426	ENSG00000050344	7mer-m8	TargetScan
miR-4426	ENSG00000050748	7mer-m8	TargetScan
miR-4426	ENSG00000056097	7mer-m8	TargetScan
miR-4426	ENSG00000057019	7mer-m8	TargetScan
miR-4426	ENSG00000061455	7mer-m8	TargetScan
miR-4426	ENSG00000063127	7mer-m8	TargetScan
miR-4426	ENSG00000064042	7mer-m8	TargetScan
miR-4426	ENSG00000064201	7mer-m8	TargetScan
miR-4426	ENSG00000065427	7mer-m8	TargetScan
miR-4426	ENSG00000065613	7mer-m8	TargetScan
miR-4426	ENSG00000065923	7mer-m8	TargetScan
miR-4426	ENSG00000066427	7mer-m8	TargetScan
miR-4426	ENSG00000066697	7mer-m8	TargetScan
miR-4426	ENSG00000066933	7mer-m8	TargetScan
miR-4426	ENSG00000067191	7mer-m8	TargetScan
miR-4426	ENSG00000068796	7mer-m8	TargetScan
miR-4426	ENSG00000069345	7mer-m8	TargetScan
miR-4426	ENSG00000069431	7mer-m8	TargetScan
miR-4426	ENSG00000069493	7mer-m8	TargetScan
miR-4426	ENSG00000071054	7mer-m8	TargetScan
miR-4426	ENSG00000071189	7mer-m8	TargetScan
miR-4426	ENSG00000072315	7mer-m8	TargetScan
miR-4426	ENSG00000072422	7mer-m8	TargetScan
miR-4426	ENSG00000073614	7mer-m8	TargetScan
miR-4426	ENSG00000074054	7mer-m8	TargetScan
miR-4426	ENSG00000075035	7mer-m8	TargetScan
miR-4426	ENSG00000075426	7mer-m8	TargetScan
miR-4426	ENSG00000076053	7mer-m8	TargetScan
miR-4426	ENSG00000077274	7mer-m8	TargetScan
miR-4426	ENSG00000077616	7mer-m8	TargetScan
miR-4426	ENSG00000078142	7mer-m8	TargetScan

miR-4426	ENSG00000078900	7mer-m8	TargetScan
miR-4426	ENSG00000079931	7mer-m8	TargetScan
miR-4426	ENSG00000079950	7mer-m8	TargetScan
miR-4426	ENSG00000080224	7mer-m8	TargetScan
miR-4426	ENSG00000080815	7mer-m8	TargetScan
miR-4426	ENSG00000081026	7mer-m8	TargetScan
miR-4426	ENSG00000082068	7mer-m8	TargetScan
miR-4426	ENSG00000082438	7mer-m8	TargetScan
miR-4426	ENSG00000082701	7mer-m8	TargetScan
miR-4426	ENSG00000082805	7mer-m8	TargetScan
miR-4426	ENSG00000083642	7mer-m8	TargetScan
miR-4426	ENSG00000087266	7mer-m8	TargetScan
miR-4426	ENSG00000089006	7mer-m8	TargetScan
miR-4426	ENSG00000089057	7mer-m8	TargetScan
miR-4426	ENSG00000090376	7mer-m8	TargetScan
miR-4426	ENSG00000091831	7mer-m8	TargetScan
miR-4426	ENSG00000093217	7mer-m8	TargetScan
miR-4426	ENSG00000094880	7mer-m8	TargetScan
miR-4426	ENSG00000095637	7mer-m8	TargetScan
miR-4426	ENSG00000097046	7mer-m8	TargetScan
miR-4426	ENSG00000099219	7mer-m8	TargetScan
miR-4426	ENSG00000099250	7mer-m8	TargetScan
miR-4426	ENSG00000100056	7mer-m8	TargetScan
miR-4426	ENSG00000100201	7mer-m8	TargetScan
miR-4426	ENSG00000100216	7mer-m8	TargetScan
miR-4426	ENSG00000100219	7mer-m8	TargetScan
miR-4426	ENSG00000100350	7mer-m8	TargetScan
miR-4426	ENSG00000100364	7mer-m8	TargetScan
miR-4426	ENSG00000100461	7mer-m8	TargetScan
miR-4426	ENSG00000100596	7mer-m8	TargetScan
miR-4426	ENSG00000100815	7mer-m8	TargetScan

miR-4426	ENSG00000101115	7mer-m8	TargetScan
miR-4426	ENSG00000101190	7mer-m8	TargetScan
miR-4426	ENSG00000101236	7mer-m8	TargetScan
miR-4426	ENSG00000101546	7mer-m8	TargetScan
miR-4426	ENSG00000101751	7mer-m8	TargetScan
miR-4426	ENSG00000101916	7mer-m8	TargetScan
miR-4426	ENSG00000101935	7mer-m8	TargetScan
miR-4426	ENSG00000102309	7mer-m8	TargetScan
miR-4426	ENSG00000102471	7mer-m8	TargetScan
miR-4426	ENSG00000103018	7mer-m8	TargetScan
miR-4426	ENSG00000103047	7mer-m8	TargetScan
miR-4426	ENSG00000103404	7mer-m8	TargetScan
miR-4426	ENSG00000103489	7mer-m8	TargetScan
miR-4426	ENSG00000103550	7mer-m8	TargetScan
miR-4426	ENSG00000104081	7mer-m8	TargetScan
miR-4426	ENSG00000104290	7mer-m8	TargetScan
miR-4426	ENSG00000104325	7mer-m8	TargetScan
miR-4426	ENSG00000104643	7mer-m8	TargetScan
miR-4426	ENSG00000104825	7mer-m8	TargetScan
miR-4426	ENSG00000105122	7mer-m8	TargetScan
miR-4426	ENSG00000105983	7mer-m8	TargetScan
miR-4426	ENSG00000106049	7mer-m8	TargetScan
miR-4426	ENSG00000106258	7mer-m8	TargetScan
miR-4426	ENSG00000106304	7mer-m8	TargetScan
miR-4426	ENSG00000106443	7mer-m8	TargetScan
miR-4426	ENSG00000106460	7mer-m8	TargetScan
miR-4426	ENSG00000106477	7mer-m8	TargetScan
miR-4426	ENSG00000106479	7mer-m8	TargetScan
miR-4426	ENSG00000106665	7mer-m8	TargetScan
miR-4426	ENSG00000106714	7mer-m8	TargetScan
miR-4426	ENSG00000107738	7mer-m8	TargetScan

miR-4426	ENSG00000107771	7mer-m8	TargetScan
miR-4426	ENSG00000107789	7mer-m8	TargetScan
miR-4426	ENSG00000107864	7mer-m8	TargetScan
miR-4426	ENSG00000108064	7mer-m8	TargetScan
miR-4426	ENSG00000108219	7mer-m8	TargetScan
miR-4426	ENSG00000108239	7mer-m8	TargetScan
miR-4426	ENSG00000108379	7mer-m8	TargetScan
miR-4426	ENSG00000108702	7mer-m8	TargetScan
miR-4426	ENSG00000109189	7mer-m8	TargetScan
miR-4426	ENSG00000109265	7mer-m8	TargetScan
miR-4426	ENSG00000109738	7mer-m8	TargetScan
miR-4426	ENSG00000109861	7mer-m8	TargetScan
miR-4426	ENSG00000110395	7mer-m8	TargetScan
miR-4426	ENSG00000110427	7mer-m8	TargetScan
miR-4426	ENSG00000110756	7mer-m8	TargetScan
miR-4426	ENSG00000110777	7mer-m8	TargetScan
miR-4426	ENSG00000110917	7mer-m8	TargetScan
miR-4426	ENSG00000111203	7mer-m8	TargetScan
miR-4426	ENSG00000111335	7mer-m8	TargetScan
miR-4426	ENSG00000111713	7mer-m8	TargetScan
miR-4426	ENSG00000111737	7mer-m8	TargetScan
miR-4426	ENSG00000111785	7mer-m8	TargetScan
miR-4426	ENSG00000111817	7mer-m8	TargetScan
miR-4426	ENSG00000111837	7mer-m8	TargetScan
miR-4426	ENSG00000111859	7mer-m8	TargetScan
miR-4426	ENSG00000111877	7mer-m8	TargetScan
miR-4426	ENSG00000111913	7mer-m8	TargetScan
miR-4426	ENSG00000112038	7mer-m8	TargetScan
miR-4426	ENSG00000112494	7mer-m8	TargetScan
miR-4426	ENSG00000112619	7mer-m8	TargetScan
miR-4426	ENSG00000112852	7mer-m8	TargetScan

miR-4426	ENSG00000112877	7mer-m8	TargetScan
miR-4426	ENSG00000113384	7mer-m8	TargetScan
miR-4426	ENSG00000113389	7mer-m8	TargetScan
miR-4426	ENSG00000113594	7mer-m8	TargetScan
miR-4426	ENSG00000113597	7mer-m8	TargetScan
miR-4426	ENSG00000113719	7mer-m8	TargetScan
miR-4426	ENSG00000113732	7mer-m8	TargetScan
miR-4426	ENSG00000113845	7mer-m8	TargetScan
miR-4426	ENSG00000114209	7mer-m8	TargetScan
miR-4426	ENSG00000114354	7mer-m8	TargetScan
miR-4426	ENSG00000114739	7mer-m8	TargetScan
miR-4426	ENSG00000114770	7mer-m8	TargetScan
miR-4426	ENSG00000114805	7mer-m8	TargetScan
miR-4426	ENSG00000114861	7mer-m8	TargetScan
miR-4426	ENSG00000115808	7mer-m8	TargetScan
miR-4426	ENSG00000115866	7mer-m8	TargetScan
miR-4426	ENSG00000115875	7mer-m8	TargetScan
miR-4426	ENSG00000115896	7mer-m8	TargetScan
miR-4426	ENSG00000115977	7mer-m8	TargetScan
miR-4426	ENSG00000116005	7mer-m8	TargetScan
miR-4426	ENSG00000116095	7mer-m8	TargetScan
miR-4426	ENSG00000116171	7mer-m8	TargetScan
miR-4426	ENSG00000116489	7mer-m8	TargetScan
miR-4426	ENSG00000116539	7mer-m8	TargetScan
miR-4426	ENSG00000116852	7mer-m8	TargetScan
miR-4426	ENSG00000117139	7mer-m8	TargetScan
miR-4426	ENSG00000117226	7mer-m8	TargetScan
miR-4426	ENSG00000117395	7mer-m8	TargetScan
miR-4426	ENSG00000117500	7mer-m8	TargetScan
miR-4426	ENSG00000117505	7mer-m8	TargetScan
miR-4426	ENSG00000117600	7mer-m8	TargetScan

miR-4426	ENSG00000118113	7mer-m8	TargetScan
miR-4426	ENSG00000118322	7mer-m8	TargetScan
miR-4426	ENSG00000118620	7mer-m8	TargetScan
miR-4426	ENSG00000118777	7mer-m8	TargetScan
miR-4426	ENSG00000119403	7mer-m8	TargetScan
miR-4426	ENSG00000119718	7mer-m8	TargetScan
miR-4426	ENSG00000119729	7mer-m8	TargetScan
miR-4426	ENSG00000119900	7mer-m8	TargetScan
miR-4426	ENSG00000120279	7mer-m8	TargetScan
miR-4426	ENSG00000120337	7mer-m8	TargetScan
miR-4426	ENSG00000120509	7mer-m8	TargetScan
miR-4426	ENSG00000120733	7mer-m8	TargetScan
miR-4426	ENSG00000120798	7mer-m8	TargetScan
miR-4426	ENSG00000120925	7mer-m8	TargetScan
miR-4426	ENSG00000121579	7mer-m8	TargetScan
miR-4426	ENSG00000121621	7mer-m8	TargetScan
miR-4426	ENSG00000121989	7mer-m8	TargetScan
miR-4426	ENSG00000122257	7mer-m8	TargetScan
miR-4426	ENSG00000122367	7mer-m8	TargetScan
miR-4426	ENSG00000122557	7mer-m8	TargetScan
miR-4426	ENSG00000122574	7mer-m8	TargetScan
miR-4426	ENSG00000122870	7mer-m8	TargetScan
miR-4426	ENSG00000123154	7mer-m8	TargetScan
miR-4426	ENSG00000123219	7mer-m8	TargetScan
miR-4426	ENSG00000123933	7mer-m8	TargetScan
miR-4426	ENSG00000124224	7mer-m8	TargetScan
miR-4426	ENSG00000124374	7mer-m8	TargetScan
miR-4426	ENSG00000124496	7mer-m8	TargetScan
miR-4426	ENSG00000124574	7mer-m8	TargetScan
miR-4426	ENSG00000124596	7mer-m8	TargetScan
miR-4426	ENSG00000125107	7mer-m8	TargetScan

miR-4426	ENSG00000125676	7mer-m8	TargetScan
miR-4426	ENSG00000125741	7mer-m8	TargetScan
miR-4426	ENSG00000125944	7mer-m8	TargetScan
miR-4426	ENSG00000126070	7mer-m8	TargetScan
miR-4426	ENSG00000126226	7mer-m8	TargetScan
miR-4426	ENSG00000126545	7mer-m8	TargetScan
miR-4426	ENSG00000126773	7mer-m8	TargetScan
miR-4426	ENSG00000127249	7mer-m8	TargetScan
miR-4426	ENSG00000127995	7mer-m8	TargetScan
miR-4426	ENSG00000128294	7mer-m8	TargetScan
miR-4426	ENSG00000128713	7mer-m8	TargetScan
miR-4426	ENSG00000128789	7mer-m8	TargetScan
miR-4426	ENSG00000128944	7mer-m8	TargetScan
miR-4426	ENSG00000129221	7mer-m8	TargetScan
miR-4426	ENSG00000129595	7mer-m8	TargetScan
miR-4426	ENSG00000130182	7mer-m8	TargetScan
miR-4426	ENSG00000130749	7mer-m8	TargetScan
miR-4426	ENSG00000131269	7mer-m8	TargetScan
miR-4426	ENSG00000131746	7mer-m8	TargetScan
miR-4426	ENSG00000131845	7mer-m8	TargetScan
miR-4426	ENSG00000132164	7mer-m8	TargetScan
miR-4426	ENSG00000132326	7mer-m8	TargetScan
miR-4426	ENSG00000132639	7mer-m8	TargetScan
miR-4426	ENSG00000132640	7mer-m8	TargetScan
miR-4426	ENSG00000133028	7mer-m8	TargetScan
miR-4426	ENSG00000133318	7mer-m8	TargetScan
miR-4426	ENSG00000133477	7mer-m8	TargetScan
miR-4426	ENSG00000133665	7mer-m8	TargetScan
miR-4426	ENSG00000133858	7mer-m8	TargetScan
miR-4426	ENSG00000134108	7mer-m8	TargetScan
miR-4426	ENSG00000134460	7mer-m8	TargetScan

miR-4426	ENSG00000134757	7mer-m8	TargetScan
miR-4426	ENSG00000134817	7mer-m8	TargetScan
miR-4426	ENSG00000134970	7mer-m8	TargetScan
miR-4426	ENSG00000135835	7mer-m8	TargetScan
miR-4426	ENSG00000135870	7mer-m8	TargetScan
miR-4426	ENSG00000135945	7mer-m8	TargetScan
miR-4426	ENSG00000136021	7mer-m8	TargetScan
miR-4426	ENSG00000136213	7mer-m8	TargetScan
miR-4426	ENSG00000136541	7mer-m8	TargetScan
miR-4426	ENSG00000136709	7mer-m8	TargetScan
miR-4426	ENSG00000136888	7mer-m8	TargetScan
miR-4426	ENSG00000136932	7mer-m8	TargetScan
miR-4426	ENSG00000137075	7mer-m8	TargetScan
miR-4426	ENSG00000137098	7mer-m8	TargetScan
miR-4426	ENSG00000137261	7mer-m8	TargetScan
miR-4426	ENSG00000137500	7mer-m8	TargetScan
miR-4426	ENSG00000137822	7mer-m8	TargetScan
miR-4426	ENSG00000137942	7mer-m8	TargetScan
miR-4426	ENSG00000138092	7mer-m8	TargetScan
miR-4426	ENSG00000138363	7mer-m8	TargetScan
miR-4426	ENSG00000138411	7mer-m8	TargetScan
miR-4426	ENSG00000138430	7mer-m8	TargetScan
miR-4426	ENSG00000138443	7mer-m8	TargetScan
miR-4426	ENSG00000138593	7mer-m8	TargetScan
miR-4426	ENSG00000138613	7mer-m8	TargetScan
miR-4426	ENSG00000138653	7mer-m8	TargetScan
miR-4426	ENSG00000138670	7mer-m8	TargetScan
miR-4426	ENSG00000138675	7mer-m8	TargetScan
miR-4426	ENSG00000138686	7mer-m8	TargetScan
miR-4426	ENSG00000138792	7mer-m8	TargetScan
miR-4426	ENSG00000138802	7mer-m8	TargetScan

miR-4426	ENSG00000139278	7mer-m8	TargetScan
miR-4426	ENSG00000139719	7mer-m8	TargetScan
miR-4426	ENSG00000140015	7mer-m8	TargetScan
miR-4426	ENSG00000140262	7mer-m8	TargetScan
miR-4426	ENSG00000140264	7mer-m8	TargetScan
miR-4426	ENSG00000140280	7mer-m8	TargetScan
miR-4426	ENSG00000140386	7mer-m8	TargetScan
miR-4426	ENSG00000140830	7mer-m8	TargetScan
miR-4426	ENSG00000140992	7mer-m8	TargetScan
miR-4426	ENSG00000141232	7mer-m8	TargetScan
miR-4426	ENSG00000141401	7mer-m8	TargetScan
miR-4426	ENSG00000141441	7mer-m8	TargetScan
miR-4426	ENSG00000141448	7mer-m8	TargetScan
miR-4426	ENSG00000141560	7mer-m8	TargetScan
miR-4426	ENSG00000141644	7mer-m8	TargetScan
miR-4426	ENSG00000141965	7mer-m8	TargetScan
miR-4426	ENSG00000142405	7mer-m8	TargetScan
miR-4426	ENSG00000142538	7mer-m8	TargetScan
miR-4426	ENSG00000143498	7mer-m8	TargetScan
miR-4426	ENSG00000143869	7mer-m8	TargetScan
miR-4426	ENSG00000144136	7mer-m8	TargetScan
miR-4426	ENSG00000144401	7mer-m8	TargetScan
miR-4426	ENSG00000144554	7mer-m8	TargetScan
miR-4426	ENSG00000144645	7mer-m8	TargetScan
miR-4426	ENSG00000144749	7mer-m8	TargetScan
miR-4426	ENSG00000144791	7mer-m8	TargetScan
miR-4426	ENSG00000144840	7mer-m8	TargetScan
miR-4426	ENSG00000144909	7mer-m8	TargetScan
miR-4426	ENSG00000145029	7mer-m8	TargetScan
miR-4426	ENSG00000145388	7mer-m8	TargetScan
miR-4426	ENSG00000145414	7mer-m8	TargetScan

miR-4426	ENSG00000145592	7mer-m8	TargetScan
miR-4426	ENSG00000145687	7mer-m8	TargetScan
miR-4426	ENSG00000145721	7mer-m8	TargetScan
miR-4426	ENSG00000145725	7mer-m8	TargetScan
miR-4426	ENSG00000145777	7mer-m8	TargetScan
miR-4426	ENSG00000145779	7mer-m8	TargetScan
miR-4426	ENSG00000145907	7mer-m8	TargetScan
miR-4426	ENSG00000145990	7mer-m8	TargetScan
miR-4426	ENSG00000146232	7mer-m8	TargetScan
miR-4426	ENSG00000146352	7mer-m8	TargetScan
miR-4426	ENSG00000146530	7mer-m8	TargetScan
miR-4426	ENSG00000146909	7mer-m8	TargetScan
miR-4426	ENSG00000147130	7mer-m8	TargetScan
miR-4426	ENSG00000147274	7mer-m8	TargetScan
miR-4426	ENSG00000147394	7mer-m8	TargetScan
miR-4426	ENSG00000147419	7mer-m8	TargetScan
miR-4426	ENSG00000147862	7mer-m8	TargetScan
miR-4426	ENSG00000147894	7mer-m8	TargetScan
miR-4426	ENSG00000148053	7mer-m8	TargetScan
miR-4426	ENSG00000148219	7mer-m8	TargetScan
miR-4426	ENSG00000148429	7mer-m8	TargetScan
miR-4426	ENSG00000148604	7mer-m8	TargetScan
miR-4426	ENSG00000149054	7mer-m8	TargetScan
miR-4426	ENSG00000149124	7mer-m8	TargetScan
miR-4426	ENSG00000149187	7mer-m8	TargetScan
miR-4426	ENSG00000149485	7mer-m8	TargetScan
miR-4426	ENSG00000149532	7mer-m8	TargetScan
miR-4426	ENSG00000151247	7mer-m8	TargetScan
miR-4426	ENSG00000151414	7mer-m8	TargetScan
miR-4426	ENSG00000151458	7mer-m8	TargetScan
miR-4426	ENSG00000151657	7mer-m8	TargetScan

miR-4426	ENSG00000152102	7mer-m8	TargetScan
miR-4426	ENSG00000152133	7mer-m8	TargetScan
miR-4426	ENSG00000152433	7mer-m8	TargetScan
miR-4426	ENSG00000152578	7mer-m8	TargetScan
miR-4426	ENSG00000152580	7mer-m8	TargetScan
miR-4426	ENSG00000152684	7mer-m8	TargetScan
miR-4426	ENSG00000153140	7mer-m8	TargetScan
miR-4426	ENSG00000153339	7mer-m8	TargetScan
miR-4426	ENSG00000153395	7mer-m8	TargetScan
miR-4426	ENSG00000153707	7mer-m8	TargetScan
miR-4426	ENSG00000153976	7mer-m8	TargetScan
miR-4426	ENSG00000154040	7mer-m8	TargetScan
miR-4426	ENSG00000154124	7mer-m8	TargetScan
miR-4426	ENSG00000154310	7mer-m8	TargetScan
miR-4426	ENSG00000154380	7mer-m8	TargetScan
miR-4426	ENSG00000154511	7mer-m8	TargetScan
miR-4426	ENSG00000154655	7mer-m8	TargetScan
miR-4426	ENSG00000154678	7mer-m8	TargetScan
miR-4426	ENSG00000155008	7mer-m8	TargetScan
miR-4426	ENSG00000155052	7mer-m8	TargetScan
miR-4426	ENSG00000155093	7mer-m8	TargetScan
miR-4426	ENSG00000155380	7mer-m8	TargetScan
miR-4426	ENSG00000155850	7mer-m8	TargetScan
miR-4426	ENSG00000155858	7mer-m8	TargetScan
miR-4426	ENSG00000156103	7mer-m8	TargetScan
miR-4426	ENSG00000156500	7mer-m8	TargetScan
miR-4426	ENSG00000156535	7mer-m8	TargetScan
miR-4426	ENSG00000156587	7mer-m8	TargetScan
miR-4426	ENSG00000156675	7mer-m8	TargetScan
miR-4426	ENSG00000156687	7mer-m8	TargetScan
miR-4426	ENSG00000156958	7mer-m8	TargetScan

miR-4426	ENSG00000157064	7mer-m8	TargetScan
miR-4426	ENSG00000157150	7mer-m8	TargetScan
miR-4426	ENSG00000157404	7mer-m8	TargetScan
miR-4426	ENSG00000157450	7mer-m8	TargetScan
miR-4426	ENSG00000157557	7mer-m8	TargetScan
miR-4426	ENSG00000158006	7mer-m8	TargetScan
miR-4426	ENSG00000158301	7mer-m8	TargetScan
miR-4426	ENSG00000158714	7mer-m8	TargetScan
miR-4426	ENSG00000159556	7mer-m8	TargetScan
miR-4426	ENSG00000159784	7mer-m8	TargetScan
miR-4426	ENSG00000160654	7mer-m8	TargetScan
miR-4426	ENSG00000160961	7mer-m8	TargetScan
miR-4426	ENSG00000161243	7mer-m8	TargetScan
miR-4426	ENSG00000161249	7mer-m8	TargetScan
miR-4426	ENSG00000161533	7mer-m8	TargetScan
miR-4426	ENSG00000161671	7mer-m8	TargetScan
miR-4426	ENSG00000162456	7mer-m8	TargetScan
miR-4426	ENSG00000162521	7mer-m8	TargetScan
miR-4426	ENSG00000162601	7mer-m8	TargetScan
miR-4426	ENSG00000162613	7mer-m8	TargetScan
miR-4426	ENSG00000162616	7mer-m8	TargetScan
miR-4426	ENSG00000162636	7mer-m8	TargetScan
miR-4426	ENSG00000162981	7mer-m8	TargetScan
miR-4426	ENSG00000163249	7mer-m8	TargetScan
miR-4426	ENSG00000163377	7mer-m8	TargetScan
miR-4426	ENSG00000163393	7mer-m8	TargetScan
miR-4426	ENSG00000163501	7mer-m8	TargetScan
miR-4426	ENSG00000163513	7mer-m8	TargetScan
miR-4426	ENSG00000163590	7mer-m8	TargetScan
miR-4426	ENSG00000163629	7mer-m8	TargetScan
miR-4426	ENSG00000163637	7mer-m8	TargetScan

miR-4426	ENSG00000163655	7mer-m8	TargetScan
miR-4426	ENSG00000163684	7mer-m8	TargetScan
miR-4426	ENSG00000163812	7mer-m8	TargetScan
miR-4426	ENSG00000163818	7mer-m8	TargetScan
miR-4426	ENSG00000164002	7mer-m8	TargetScan
miR-4426	ENSG00000164031	7mer-m8	TargetScan
miR-4426	ENSG00000164038	7mer-m8	TargetScan
miR-4426	ENSG00000164066	7mer-m8	TargetScan
miR-4426	ENSG00000164266	7mer-m8	TargetScan
miR-4426	ENSG00000164291	7mer-m8	TargetScan
miR-4426	ENSG00000164294	7mer-m8	TargetScan
miR-4426	ENSG00000164338	7mer-m8	TargetScan
miR-4426	ENSG00000164393	7mer-m8	TargetScan
miR-4426	ENSG00000164576	7mer-m8	TargetScan
miR-4426	ENSG00000164692	7mer-m8	TargetScan
miR-4426	ENSG00000164879	7mer-m8	TargetScan
miR-4426	ENSG00000164976	7mer-m8	TargetScan
miR-4426	ENSG00000164983	7mer-m8	TargetScan
miR-4426	ENSG00000165060	7mer-m8	TargetScan
miR-4426	ENSG00000165168	7mer-m8	TargetScan
miR-4426	ENSG00000165240	7mer-m8	TargetScan
miR-4426	ENSG00000165282	7mer-m8	TargetScan
miR-4426	ENSG00000165355	7mer-m8	TargetScan
miR-4426	ENSG00000165379	7mer-m8	TargetScan
miR-4426	ENSG00000165555	7mer-m8	TargetScan
miR-4426	ENSG00000165813	7mer-m8	TargetScan
miR-4426	ENSG00000165832	7mer-m8	TargetScan
miR-4426	ENSG00000166261	7mer-m8	TargetScan
miR-4426	ENSG00000166342	7mer-m8	TargetScan
miR-4426	ENSG00000166451	7mer-m8	TargetScan
miR-4426	ENSG00000166483	7mer-m8	TargetScan

miR-4426	ENSG00000166881	7mer-m8	TargetScan
miR-4426	ENSG00000166974	7mer-m8	TargetScan
miR-4426	ENSG00000167315	7mer-m8	TargetScan
miR-4426	ENSG00000167325	7mer-m8	TargetScan
miR-4426	ENSG00000167720	7mer-m8	TargetScan
miR-4426	ENSG00000167770	7mer-m8	TargetScan
miR-4426	ENSG00000167972	7mer-m8	TargetScan
miR-4426	ENSG00000168172	7mer-m8	TargetScan
miR-4426	ENSG00000168685	7mer-m8	TargetScan
miR-4426	ENSG00000168938	7mer-m8	TargetScan
miR-4426	ENSG00000169139	7mer-m8	TargetScan
miR-4426	ENSG00000169174	7mer-m8	TargetScan
miR-4426	ENSG00000169427	7mer-m8	TargetScan
miR-4426	ENSG00000169429	7mer-m8	TargetScan
miR-4426	ENSG00000169432	7mer-m8	TargetScan
miR-4426	ENSG00000169550	7mer-m8	TargetScan
miR-4426	ENSG00000169567	7mer-m8	TargetScan
miR-4426	ENSG00000169814	7mer-m8	TargetScan
miR-4426	ENSG00000169891	7mer-m8	TargetScan
miR-4426	ENSG00000170153	7mer-m8	TargetScan
miR-4426	ENSG00000170242	7mer-m8	TargetScan
miR-4426	ENSG00000170374	7mer-m8	TargetScan
miR-4426	ENSG00000170515	7mer-m8	TargetScan
miR-4426	ENSG00000170537	7mer-m8	TargetScan
miR-4426	ENSG00000170542	7mer-m8	TargetScan
miR-4426	ENSG00000170571	7mer-m8	TargetScan
miR-4426	ENSG00000170634	7mer-m8	TargetScan
miR-4426	ENSG00000170653	7mer-m8	TargetScan
miR-4426	ENSG00000171126	7mer-m8	TargetScan
miR-4426	ENSG00000171150	7mer-m8	TargetScan
miR-4426	ENSG00000171450	7mer-m8	TargetScan

miR-4426	ENSG00000171509	7mer-m8	TargetScan
miR-4426	ENSG00000171587	7mer-m8	TargetScan
miR-4426	ENSG00000171914	7mer-m8	TargetScan
miR-4426	ENSG00000172115	7mer-m8	TargetScan
miR-4426	ENSG00000172403	7mer-m8	TargetScan
miR-4426	ENSG00000172466	7mer-m8	TargetScan
miR-4426	ENSG00000172572	7mer-m8	TargetScan
miR-4426	ENSG00000172728	7mer-m8	TargetScan
miR-4426	ENSG00000172733	7mer-m8	TargetScan
miR-4426	ENSG00000172840	7mer-m8	TargetScan
miR-4426	ENSG00000173681	7mer-m8	TargetScan
miR-4426	ENSG00000173726	7mer-m8	TargetScan
miR-4426	ENSG00000174606	7mer-m8	TargetScan
miR-4426	ENSG00000174718	7mer-m8	TargetScan
miR-4426	ENSG00000174840	7mer-m8	TargetScan
miR-4426	ENSG00000174989	7mer-m8	TargetScan
miR-4426	ENSG00000175048	7mer-m8	TargetScan
miR-4426	ENSG00000175066	7mer-m8	TargetScan
miR-4426	ENSG00000175073	7mer-m8	TargetScan
miR-4426	ENSG00000175264	7mer-m8	TargetScan
miR-4426	ENSG00000175329	7mer-m8	TargetScan
miR-4426	ENSG00000176024	7mer-m8	TargetScan
miR-4426	ENSG00000176783	7mer-m8	TargetScan
miR-4426	ENSG00000177042	7mer-m8	TargetScan
miR-4426	ENSG00000177076	7mer-m8	TargetScan
miR-4426	ENSG00000177119	7mer-m8	TargetScan
miR-4426	ENSG00000177150	7mer-m8	TargetScan
miR-4426	ENSG00000177511	7mer-m8	TargetScan
miR-4426	ENSG00000177889	7mer-m8	TargetScan
miR-4426	ENSG00000177963	7mer-m8	TargetScan
miR-4426	ENSG00000178177	7mer-m8	TargetScan

miR-4426	ENSG00000178307	7mer-m8	TargetScan
miR-4426	ENSG00000178425	7mer-m8	TargetScan
miR-4426	ENSG00000179097	7mer-m8	TargetScan
miR-4426	ENSG00000179361	7mer-m8	TargetScan
miR-4426	ENSG00000179562	7mer-m8	TargetScan
miR-4426	ENSG00000179813	7mer-m8	TargetScan
miR-4426	ENSG00000179820	7mer-m8	TargetScan
miR-4426	ENSG00000180628	7mer-m8	TargetScan
miR-4426	ENSG00000181104	7mer-m8	TargetScan
miR-4426	ENSG00000181291	7mer-m8	TargetScan
miR-4426	ENSG00000181396	7mer-m8	TargetScan
miR-4426	ENSG00000181666	7mer-m8	TargetScan
miR-4426	ENSG00000181789	7mer-m8	TargetScan
miR-4426	ENSG00000181826	7mer-m8	TargetScan
miR-4426	ENSG00000181908	7mer-m8	TargetScan
miR-4426	ENSG00000182111	7mer-m8	TargetScan
miR-4426	ENSG00000182197	7mer-m8	TargetScan
miR-4426	ENSG00000182348	7mer-m8	TargetScan
miR-4426	ENSG00000183576	7mer-m8	TargetScan
miR-4426	ENSG00000183690	7mer-m8	TargetScan
miR-4426	ENSG00000183760	7mer-m8	TargetScan
miR-4426	ENSG00000183775	7mer-m8	TargetScan
miR-4426	ENSG00000183779	7mer-m8	TargetScan
miR-4426	ENSG00000183831	7mer-m8	TargetScan
miR-4426	ENSG00000183850	7mer-m8	TargetScan
miR-4426	ENSG00000184156	7mer-m8	TargetScan
miR-4426	ENSG00000184182	7mer-m8	TargetScan
miR-4426	ENSG00000184220	7mer-m8	TargetScan
miR-4426	ENSG00000184697	7mer-m8	TargetScan
miR-4426	ENSG00000184719	7mer-m8	TargetScan
miR-4426	ENSG00000184831	7mer-m8	TargetScan

miR-4426	ENSG00000184845	7mer-m8	TargetScan
miR-4426	ENSG00000185339	7mer-m8	TargetScan
miR-4426	ENSG00000185437	7mer-m8	TargetScan
miR-4426	ENSG00000185561	7mer-m8	TargetScan
miR-4426	ENSG00000185630	7mer-m8	TargetScan
miR-4426	ENSG00000185658	7mer-m8	TargetScan
miR-4426	ENSG00000185730	7mer-m8	TargetScan
miR-4426	ENSG00000185745	7mer-m8	TargetScan
miR-4426	ENSG00000186188	7mer-m8	TargetScan
miR-4426	ENSG00000186230	7mer-m8	TargetScan
miR-4426	ENSG00000186260	7mer-m8	TargetScan
miR-4426	ENSG00000186431	7mer-m8	TargetScan
miR-4426	ENSG00000186432	7mer-m8	TargetScan
miR-4426	ENSG00000186458	7mer-m8	TargetScan
miR-4426	ENSG00000186468	7mer-m8	TargetScan
miR-4426	ENSG00000186591	7mer-m8	TargetScan
miR-4426	ENSG00000186806	7mer-m8	TargetScan
miR-4426	ENSG00000186838	7mer-m8	TargetScan
miR-4426	ENSG00000187097	7mer-m8	TargetScan
miR-4426	ENSG00000187123	7mer-m8	TargetScan
miR-4426	ENSG00000187533	7mer-m8	TargetScan
miR-4426	ENSG00000187699	7mer-m8	TargetScan
miR-4426	ENSG00000187726	7mer-m8	TargetScan
miR-4426	ENSG00000187790	7mer-m8	TargetScan
miR-4426	ENSG00000188171	7mer-m8	TargetScan
miR-4426	ENSG00000188176	7mer-m8	TargetScan
miR-4426	ENSG00000188215	7mer-m8	TargetScan
miR-4426	ENSG00000188227	7mer-m8	TargetScan
miR-4426	ENSG00000188735	7mer-m8	TargetScan
miR-4426	ENSG00000188817	7mer-m8	TargetScan
miR-4426	ENSG00000188848	7mer-m8	TargetScan

miR-4426	ENSG00000189013	7mer-m8	TargetScan
miR-4426	ENSG00000189120	7mer-m8	TargetScan
miR-4426	ENSG00000189144	7mer-m8	TargetScan
miR-4426	ENSG00000189221	7mer-m8	TargetScan
miR-4426	ENSG00000189241	7mer-m8	TargetScan
miR-4426	ENSG00000189299	7mer-m8	TargetScan
miR-4426	ENSG00000189320	7mer-m8	TargetScan
miR-4426	ENSG00000196090	7mer-m8	TargetScan
miR-4426	ENSG00000196172	7mer-m8	TargetScan
miR-4426	ENSG00000196233	7mer-m8	TargetScan
miR-4426	ENSG00000196381	7mer-m8	TargetScan
miR-4426	ENSG00000196466	7mer-m8	TargetScan
miR-4426	ENSG00000196549	7mer-m8	TargetScan
miR-4426	ENSG00000196581	7mer-m8	TargetScan
miR-4426	ENSG00000196670	7mer-m8	TargetScan
miR-4426	ENSG00000196935	7mer-m8	TargetScan
miR-4426	ENSG00000197081	7mer-m8	TargetScan
miR-4426	ENSG00000197343	7mer-m8	TargetScan
miR-4426	ENSG00000197601	7mer-m8	TargetScan
miR-4426	ENSG00000197614	7mer-m8	TargetScan
miR-4426	ENSG00000197937	7mer-m8	TargetScan
miR-4426	ENSG00000198010	7mer-m8	TargetScan
miR-4426	ENSG00000198042	7mer-m8	TargetScan
miR-4426	ENSG00000198056	7mer-m8	TargetScan
miR-4426	ENSG00000198178	7mer-m8	TargetScan
miR-4426	ENSG00000198265	7mer-m8	TargetScan
miR-4426	ENSG00000198663	7mer-m8	TargetScan
miR-4426	ENSG00000198736	7mer-m8	TargetScan
miR-4426	ENSG00000204007	7mer-m8	TargetScan
miR-4426	ENSG00000204020	7mer-m8	TargetScan
miR-4426	ENSG00000204161	7mer-m8	TargetScan

miR-4426	ENSG00000204176	7mer-m8	TargetScan
miR-4426	ENSG00000204308	7mer-m8	TargetScan
miR-4426	ENSG00000204389	7mer-m8	TargetScan
miR-4426	ENSG00000204611	7mer-m8	TargetScan
miR-4426	ENSG00000204681	7mer-m8	TargetScan
miR-4426	ENSG00000204767	7mer-m8	TargetScan
miR-4426	ENSG00000204882	7mer-m8	TargetScan
miR-4426	ENSG00000204952	7mer-m8	TargetScan
miR-4426	ENSG00000205476	7mer-m8	TargetScan
miR-4426	ENSG00000205670	7mer-m8	TargetScan
miR-4426	ENSG00000213186	7mer-m8	TargetScan
miR-4426	ENSG00000213762	7mer-m8	TargetScan
miR-4426	ENSG00000213853	7mer-m8	TargetScan
miR-4426	ENSG00000214029	7mer-m8	TargetScan
miR-4426	ENSG00000215114	7mer-m8	TargetScan
miR-4426	ENSG00000215271	7mer-m8	TargetScan
miR-4426	ENSG00000221947	7mer-m8	TargetScan
miR-4426	ENSG00000221949	7mer-m8	TargetScan
miR-4426	ENSG00000226479	7mer-m8	TargetScan
miR-4426	ENSG00000226887	7mer-m8	TargetScan
miR-4426	ENSG00000228716	7mer-m8	TargetScan
miR-4426	ENSG00000233436	7mer-m8	TargetScan
miR-4426	ENSG00000233927	7mer-m8	TargetScan
miR-4426	ENSG00000234444	7mer-m8	TargetScan
miR-4426	ENSG00000240720	7mer-m8	TargetScan
miR-4426	ENSG00000241106	7mer-m8	TargetScan
miR-4426	ENSG00000242265	7mer-m8	TargetScan
miR-4426	ENSG00000242550	7mer-m8	TargetScan
miR-4426	ENSG00000242689	7mer-m8	TargetScan
miR-4426	ENSG00000244754	7mer-m8	TargetScan
miR-4426	ENSG00000256053	7mer-m8	TargetScan

miR-4426	ENSG00000259363	7mer-m8	TargetScan
miR-4426	ENSG00000260903	7mer-m8	TargetScan
miR-4426	ENSG00000266173	7mer-m8	TargetScan
miR-4426	ENSG00000268324	7mer-m8	TargetScan
miR-4426	ENSG00000268635	7mer-m8	TargetScan
miR-4426	ENSG00000269343	7mer-m8	TargetScan
miR-4426	ENSG00000272195	7mer-m8	TargetScan
miR-4426	ENSG00000273079	7mer-m8	TargetScan
miR-4426	ENSG00000280789	7mer-m8	TargetScan
miR-4426	ENSG00000280893	7mer-m8	TargetScan
miR-4426	ENSG00000287694	7mer-m8	TargetScan
miR-4444	ENSG00000001561	7mer-m8	TargetScan
miR-4444	ENSG00000005075	7mer-m8	TargetScan
miR-4444	ENSG00000008196	7mer-m8	TargetScan
miR-4444	ENSG00000009709	7mer-m8	TargetScan
miR-4444	ENSG00000011258	7mer-m8	TargetScan
miR-4444	ENSG00000015475	7mer-m8	TargetScan
miR-4444	ENSG00000043591	7mer-m8	TargetScan
miR-4444	ENSG00000051620	7mer-m8	TargetScan
miR-4444	ENSG00000053371	7mer-m8	TargetScan
miR-4444	ENSG00000053770	7mer-m8	TargetScan
miR-4444	ENSG00000055118	7mer-m8	TargetScan
miR-4444	ENSG00000064393	7mer-m8	TargetScan
miR-4444	ENSG00000064995	7mer-m8	TargetScan
miR-4444	ENSG00000070915	7mer-m8	TargetScan
miR-4444	ENSG00000077458	7mer-m8	TargetScan
miR-4444	ENSG00000078295	7mer-m8	TargetScan
miR-4444	ENSG00000078549	7mer-m8	TargetScan
miR-4444	ENSG00000080200	7mer-m8	TargetScan
miR-4444	ENSG00000082515	7mer-m8	TargetScan
miR-4444	ENSG00000082898	7mer-m8	TargetScan

miR-4444	ENSG00000088386	7mer-m8	TargetScan
miR-4444	ENSG00000092841	7mer-m8	TargetScan
miR-4444	ENSG00000101109	7mer-m8	TargetScan
miR-4444	ENSG00000101213	7mer-m8	TargetScan
miR-4444	ENSG00000101266	7mer-m8	TargetScan
miR-4444	ENSG00000101412	7mer-m8	TargetScan
miR-4444	ENSG00000101413	7mer-m8	TargetScan
miR-4444	ENSG00000102385	7mer-m8	TargetScan
miR-4444	ENSG00000104219	7mer-m8	TargetScan
miR-4444	ENSG00000107929	7mer-m8	TargetScan
miR-4444	ENSG00000108829	7mer-m8	TargetScan
miR-4444	ENSG00000112531	7mer-m8	TargetScan
miR-4444	ENSG00000113739	7mer-m8	TargetScan
miR-4444	ENSG00000116675	7mer-m8	TargetScan
miR-4444	ENSG00000117000	7mer-m8	TargetScan
miR-4444	ENSG00000117020	7mer-m8	TargetScan
miR-4444	ENSG00000122958	7mer-m8	TargetScan
miR-4444	ENSG00000124602	7mer-m8	TargetScan
miR-4444	ENSG00000124731	7mer-m8	TargetScan
miR-4444	ENSG00000125285	7mer-m8	TargetScan
miR-4444	ENSG00000125508	7mer-m8	TargetScan
miR-4444	ENSG00000129250	7mer-m8	TargetScan
miR-4444	ENSG00000130703	7mer-m8	TargetScan
miR-4444	ENSG00000131931	7mer-m8	TargetScan
miR-4444	ENSG00000133937	7mer-m8	TargetScan
miR-4444	ENSG00000134317	7mer-m8	TargetScan
miR-4444	ENSG00000134352	7mer-m8	TargetScan
miR-4444	ENSG00000134444	7mer-m8	TargetScan
miR-4444	ENSG00000135333	7mer-m8	TargetScan
miR-4444	ENSG00000136709	7mer-m8	TargetScan
miR-4444	ENSG00000137547	7mer-m8	TargetScan

miR-4444	ENSG00000137992	7mer-m8	TargetScan
miR-4444	ENSG00000139974	7mer-m8	TargetScan
miR-4444	ENSG00000139977	7mer-m8	TargetScan
miR-4444	ENSG00000141068	7mer-m8	TargetScan
miR-4444	ENSG00000141570	7mer-m8	TargetScan
miR-4444	ENSG00000142556	7mer-m8	TargetScan
miR-4444	ENSG00000144036	7mer-m8	TargetScan
miR-4444	ENSG00000144567	7mer-m8	TargetScan
miR-4444	ENSG00000148826	7mer-m8	TargetScan
miR-4444	ENSG00000150540	7mer-m8	TargetScan
miR-4444	ENSG00000151849	7mer-m8	TargetScan
miR-4444	ENSG00000152056	7mer-m8	TargetScan
miR-4444	ENSG00000152377	7mer-m8	TargetScan
miR-4444	ENSG00000154124	7mer-m8	TargetScan
miR-4444	ENSG00000156587	7mer-m8	TargetScan
miR-4444	ENSG00000159723	7mer-m8	TargetScan
miR-4444	ENSG00000160293	7mer-m8	TargetScan
miR-4444	ENSG00000161057	7mer-m8	TargetScan
miR-4444	ENSG00000162981	7mer-m8	TargetScan
miR-4444	ENSG00000163013	7mer-m8	TargetScan
miR-4444	ENSG00000164187	7mer-m8	TargetScan
miR-4444	ENSG00000164512	7mer-m8	TargetScan
miR-4444	ENSG00000164603	7mer-m8	TargetScan
miR-4444	ENSG00000165055	7mer-m8	TargetScan
miR-4444	ENSG00000165490	7mer-m8	TargetScan
miR-4444	ENSG00000166450	7mer-m8	TargetScan
miR-4444	ENSG00000166913	7mer-m8	TargetScan
miR-4444	ENSG00000166928	7mer-m8	TargetScan
miR-4444	ENSG00000168496	7mer-m8	TargetScan
miR-4444	ENSG00000168958	7mer-m8	TargetScan
miR-4444	ENSG00000170500	7mer-m8	TargetScan

miR-4444	ENSG00000170873	7mer-m8	TargetScan
miR-4444	ENSG00000170927	7mer-m8	TargetScan
miR-4444	ENSG00000171448	7mer-m8	TargetScan
miR-4444	ENSG00000171517	7mer-m8	TargetScan
miR-4444	ENSG00000171617	7mer-m8	TargetScan
miR-4444	ENSG00000173681	7mer-m8	TargetScan
miR-4444	ENSG00000175106	7mer-m8	TargetScan
miR-4444	ENSG00000177971	7mer-m8	TargetScan
miR-4444	ENSG00000181264	7mer-m8	TargetScan
miR-4444	ENSG00000181896	7mer-m8	TargetScan
miR-4444	ENSG00000182107	7mer-m8	TargetScan
miR-4444	ENSG00000182831	7mer-m8	TargetScan
miR-4444	ENSG00000182985	7mer-m8	TargetScan
miR-4444	ENSG00000184530	7mer-m8	TargetScan
miR-4444	ENSG00000185278	7mer-m8	TargetScan
miR-4444	ENSG00000186976	7mer-m8	TargetScan
miR-4444	ENSG00000187239	7mer-m8	TargetScan
miR-4444	ENSG00000188822	7mer-m8	TargetScan
miR-4444	ENSG00000204977	7mer-m8	TargetScan
miR-4444	ENSG00000213699	7mer-m8	TargetScan
miR-4444	ENSG00000214046	7mer-m8	TargetScan
miR-4444	ENSG00000214113	7mer-m8	TargetScan
miR-4444	ENSG00000222009	7mer-m8	TargetScan
miR-4444	ENSG00000253251	7mer-m8	TargetScan
miR-4468	ENSG00000000003	7mer-m8	TargetScan
miR-4468	ENSG00000002834	7mer-m8	TargetScan
miR-4468	ENSG00000003249	7mer-m8	TargetScan
miR-4468	ENSG00000004866	7mer-m8	TargetScan
miR-4468	ENSG00000005175	7mer-m8	TargetScan
miR-4468	ENSG00000005810	7mer-m8	TargetScan
miR-4468	ENSG00000006432	7mer-m8	TargetScan

miR-4468	ENSG00000006747	7mer-m8	TargetScan
miR-4468	ENSG00000006756	7mer-m8	TargetScan
miR-4468	ENSG00000007174	7mer-m8	TargetScan
miR-4468	ENSG00000007376	7mer-m8	TargetScan
miR-4468	ENSG00000007392	7mer-m8	TargetScan
miR-4468	ENSG00000008130	7mer-m8	TargetScan
miR-4468	ENSG00000008283	7mer-m8	TargetScan
miR-4468	ENSG00000009765	7mer-m8	TargetScan
miR-4468	ENSG00000009830	7mer-m8	TargetScan
miR-4468	ENSG00000010282	7mer-m8	TargetScan
miR-4468	ENSG00000011347	7mer-m8	TargetScan
miR-4468	ENSG00000011451	7mer-m8	TargetScan
miR-4468	ENSG00000012822	7mer-m8	TargetScan
miR-4468	ENSG00000014138	7mer-m8	TargetScan
miR-4468	ENSG00000019582	7mer-m8	TargetScan
miR-4468	ENSG00000020922	7mer-m8	TargetScan
miR-4468	ENSG00000023902	7mer-m8	TargetScan
miR-4468	ENSG00000025770	7mer-m8	TargetScan
miR-4468	ENSG00000026652	7mer-m8	TargetScan
miR-4468	ENSG00000026751	7mer-m8	TargetScan
miR-4468	ENSG00000029364	7mer-m8	TargetScan
miR-4468	ENSG00000030582	7mer-m8	TargetScan
miR-4468	ENSG00000033011	7mer-m8	TargetScan
miR-4468	ENSG00000034152	7mer-m8	TargetScan
miR-4468	ENSG00000037280	7mer-m8	TargetScan
miR-4468	ENSG00000039068	7mer-m8	TargetScan
miR-4468	ENSG00000040341	7mer-m8	TargetScan
miR-4468	ENSG00000041802	7mer-m8	TargetScan
miR-4468	ENSG00000043143	7mer-m8	TargetScan
miR-4468	ENSG00000047578	7mer-m8	TargetScan
miR-4468	ENSG00000048392	7mer-m8	TargetScan

miR-4468	ENSG00000049618	7mer-m8	TargetScan
miR-4468	ENSG00000049883	7mer-m8	TargetScan
miR-4468	ENSG00000050393	7mer-m8	TargetScan
miR-4468	ENSG00000050748	7mer-m8	TargetScan
miR-4468	ENSG00000051620	7mer-m8	TargetScan
miR-4468	ENSG00000053702	7mer-m8	TargetScan
miR-4468	ENSG00000054282	7mer-m8	TargetScan
miR-4468	ENSG00000054392	7mer-m8	TargetScan
miR-4468	ENSG00000054523	7mer-m8	TargetScan
miR-4468	ENSG00000058335	7mer-m8	TargetScan
miR-4468	ENSG00000058866	7mer-m8	TargetScan
miR-4468	ENSG00000059122	7mer-m8	TargetScan
miR-4468	ENSG00000059145	7mer-m8	TargetScan
miR-4468	ENSG00000060709	7mer-m8	TargetScan
miR-4468	ENSG00000060982	7mer-m8	TargetScan
miR-4468	ENSG00000063854	7mer-m8	TargetScan
miR-4468	ENSG00000064115	7mer-m8	TargetScan
miR-4468	ENSG00000064393	7mer-m8	TargetScan
miR-4468	ENSG00000065135	7mer-m8	TargetScan
miR-4468	ENSG00000065243	7mer-m8	TargetScan
miR-4468	ENSG00000065457	7mer-m8	TargetScan
miR-4468	ENSG00000065600	7mer-m8	TargetScan
miR-4468	ENSG00000065802	7mer-m8	TargetScan
miR-4468	ENSG00000066084	7mer-m8	TargetScan
miR-4468	ENSG00000066294	7mer-m8	TargetScan
miR-4468	ENSG00000066654	7mer-m8	TargetScan
miR-4468	ENSG00000068394	7mer-m8	TargetScan
miR-4468	ENSG00000069329	7mer-m8	TargetScan
miR-4468	ENSG00000069424	7mer-m8	TargetScan
miR-4468	ENSG00000070081	7mer-m8	TargetScan
miR-4468	ENSG00000070190	7mer-m8	TargetScan

miR-4468	ENSG00000070495	7mer-m8	TargetScan
miR-4468	ENSG00000070882	7mer-m8	TargetScan
miR-4468	ENSG00000071189	7mer-m8	TargetScan
miR-4468	ENSG00000071655	7mer-m8	TargetScan
miR-4468	ENSG00000072849	7mer-m8	TargetScan
miR-4468	ENSG00000073464	7mer-m8	TargetScan
miR-4468	ENSG00000074755	7mer-m8	TargetScan
miR-4468	ENSG00000075035	7mer-m8	TargetScan
miR-4468	ENSG00000075043	7mer-m8	TargetScan
miR-4468	ENSG00000075651	7mer-m8	TargetScan
miR-4468	ENSG00000076321	7mer-m8	TargetScan
miR-4468	ENSG00000076641	7mer-m8	TargetScan
miR-4468	ENSG00000077327	7mer-m8	TargetScan
miR-4468	ENSG00000078124	7mer-m8	TargetScan
miR-4468	ENSG00000078295	7mer-m8	TargetScan
miR-4468	ENSG00000078900	7mer-m8	TargetScan
miR-4468	ENSG00000079102	7mer-m8	TargetScan
miR-4468	ENSG00000079156	7mer-m8	TargetScan
miR-4468	ENSG00000079308	7mer-m8	TargetScan
miR-4468	ENSG00000079950	7mer-m8	TargetScan
miR-4468	ENSG00000080166	7mer-m8	TargetScan
miR-4468	ENSG00000080845	7mer-m8	TargetScan
miR-4468	ENSG00000081148	7mer-m8	TargetScan
miR-4468	ENSG00000081307	7mer-m8	TargetScan
miR-4468	ENSG00000081870	7mer-m8	TargetScan
miR-4468	ENSG00000082701	7mer-m8	TargetScan
miR-4468	ENSG00000082805	7mer-m8	TargetScan
miR-4468	ENSG00000085117	7mer-m8	TargetScan
miR-4468	ENSG00000085449	7mer-m8	TargetScan
miR-4468	ENSG00000085741	7mer-m8	TargetScan
miR-4468	ENSG00000086475	7mer-m8	TargetScan

miR-4468	ENSG00000087253	7mer-m8	TargetScan
miR-4468	ENSG00000087274	7mer-m8	TargetScan
miR-4468	ENSG00000087301	7mer-m8	TargetScan
miR-4468	ENSG00000087460	7mer-m8	TargetScan
miR-4468	ENSG00000087470	7mer-m8	TargetScan
miR-4468	ENSG00000089063	7mer-m8	TargetScan
miR-4468	ENSG00000089356	7mer-m8	TargetScan
miR-4468	ENSG00000089916	7mer-m8	TargetScan
miR-4468	ENSG00000090615	7mer-m8	TargetScan
miR-4468	ENSG00000090863	7mer-m8	TargetScan
miR-4468	ENSG00000090924	7mer-m8	TargetScan
miR-4468	ENSG00000091527	7mer-m8	TargetScan
miR-4468	ENSG00000091622	7mer-m8	TargetScan
miR-4468	ENSG00000092054	7mer-m8	TargetScan
miR-4468	ENSG00000094755	7mer-m8	TargetScan
miR-4468	ENSG00000094804	7mer-m8	TargetScan
miR-4468	ENSG00000096060	7mer-m8	TargetScan
miR-4468	ENSG00000096968	7mer-m8	TargetScan
miR-4468	ENSG00000099282	7mer-m8	TargetScan
miR-4468	ENSG00000099385	7mer-m8	TargetScan
miR-4468	ENSG00000099715	7mer-m8	TargetScan
miR-4468	ENSG00000099875	7mer-m8	TargetScan
miR-4468	ENSG00000099968	7mer-m8	TargetScan
miR-4468	ENSG00000100012	7mer-m8	TargetScan
miR-4468	ENSG00000100053	7mer-m8	TargetScan
miR-4468	ENSG00000100099	7mer-m8	TargetScan
miR-4468	ENSG00000100105	7mer-m8	TargetScan
miR-4468	ENSG00000100142	7mer-m8	TargetScan
miR-4468	ENSG00000100162	7mer-m8	TargetScan
miR-4468	ENSG00000100226	7mer-m8	TargetScan
miR-4468	ENSG00000100304	7mer-m8	TargetScan

miR-4468	ENSG00000100364	7mer-m8	TargetScan
miR-4468	ENSG00000100441	7mer-m8	TargetScan
miR-4468	ENSG00000100461	7mer-m8	TargetScan
miR-4468	ENSG00000100528	7mer-m8	TargetScan
miR-4468	ENSG00000100596	7mer-m8	TargetScan
miR-4468	ENSG00000100650	7mer-m8	TargetScan
miR-4468	ENSG00000100796	7mer-m8	TargetScan
miR-4468	ENSG00000100811	7mer-m8	TargetScan
miR-4468	ENSG00000100842	7mer-m8	TargetScan
miR-4468	ENSG00000101019	7mer-m8	TargetScan
miR-4468	ENSG00000101162	7mer-m8	TargetScan
miR-4468	ENSG00000101230	7mer-m8	TargetScan
miR-4468	ENSG00000101265	7mer-m8	TargetScan
miR-4468	ENSG00000101282	7mer-m8	TargetScan
miR-4468	ENSG00000101290	7mer-m8	TargetScan
miR-4468	ENSG00000101307	7mer-m8	TargetScan
miR-4468	ENSG00000101412	7mer-m8	TargetScan
miR-4468	ENSG00000101546	7mer-m8	TargetScan
miR-4468	ENSG00000101670	7mer-m8	TargetScan
miR-4468	ENSG00000101916	7mer-m8	TargetScan
miR-4468	ENSG00000101966	7mer-m8	TargetScan
miR-4468	ENSG00000102053	7mer-m8	TargetScan
miR-4468	ENSG00000102104	7mer-m8	TargetScan
miR-4468	ENSG00000102172	7mer-m8	TargetScan
miR-4468	ENSG00000102290	7mer-m8	TargetScan
miR-4468	ENSG00000102313	7mer-m8	TargetScan
miR-4468	ENSG00000102383	7mer-m8	TargetScan
miR-4468	ENSG00000102870	7mer-m8	TargetScan
miR-4468	ENSG00000102900	7mer-m8	TargetScan
miR-4468	ENSG00000102921	7mer-m8	TargetScan
miR-4468	ENSG00000103005	7mer-m8	TargetScan

miR-4468	ENSG00000103056	7mer-m8	TargetScan
miR-4468	ENSG00000103196	7mer-m8	TargetScan
miR-4468	ENSG00000103257	7mer-m8	TargetScan
miR-4468	ENSG00000103353	7mer-m8	TargetScan
miR-4468	ENSG00000103356	7mer-m8	TargetScan
miR-4468	ENSG00000103404	7mer-m8	TargetScan
miR-4468	ENSG00000103423	7mer-m8	TargetScan
miR-4468	ENSG00000103485	7mer-m8	TargetScan
miR-4468	ENSG00000103490	7mer-m8	TargetScan
miR-4468	ENSG00000103769	7mer-m8	TargetScan
miR-4468	ENSG00000104081	7mer-m8	TargetScan
miR-4468	ENSG00000104142	7mer-m8	TargetScan
miR-4468	ENSG00000104154	7mer-m8	TargetScan
miR-4468	ENSG00000104332	7mer-m8	TargetScan
miR-4468	ENSG00000104427	7mer-m8	TargetScan
miR-4468	ENSG00000104611	7mer-m8	TargetScan
miR-4468	ENSG00000104671	7mer-m8	TargetScan
miR-4468	ENSG00000104731	7mer-m8	TargetScan
miR-4468	ENSG00000104866	7mer-m8	TargetScan
miR-4468	ENSG00000104938	7mer-m8	TargetScan
miR-4468	ENSG00000104960	7mer-m8	TargetScan
miR-4468	ENSG00000105127	7mer-m8	TargetScan
miR-4468	ENSG00000105281	7mer-m8	TargetScan
miR-4468	ENSG00000105352	7mer-m8	TargetScan
miR-4468	ENSG00000105366	7mer-m8	TargetScan
miR-4468	ENSG00000105402	7mer-m8	TargetScan
miR-4468	ENSG00000105419	7mer-m8	TargetScan
miR-4468	ENSG00000105492	7mer-m8	TargetScan
miR-4468	ENSG00000105514	7mer-m8	TargetScan
miR-4468	ENSG00000105750	7mer-m8	TargetScan
miR-4468	ENSG00000105835	7mer-m8	TargetScan

miR-4468	ENSG00000106344	7mer-m8	TargetScan
miR-4468	ENSG00000106399	7mer-m8	TargetScan
miR-4468	ENSG00000107130	7mer-m8	TargetScan
miR-4468	ENSG00000107185	7mer-m8	TargetScan
miR-4468	ENSG00000107282	7mer-m8	TargetScan
miR-4468	ENSG00000107562	7mer-m8	TargetScan
miR-4468	ENSG00000107581	7mer-m8	TargetScan
miR-4468	ENSG00000107929	7mer-m8	TargetScan
miR-4468	ENSG00000108106	7mer-m8	TargetScan
miR-4468	ENSG00000108175	7mer-m8	TargetScan
miR-4468	ENSG00000108255	7mer-m8	TargetScan
miR-4468	ENSG00000108306	7mer-m8	TargetScan
miR-4468	ENSG00000108309	7mer-m8	TargetScan
miR-4468	ENSG00000108384	7mer-m8	TargetScan
miR-4468	ENSG00000108387	7mer-m8	TargetScan
miR-4468	ENSG00000108599	7mer-m8	TargetScan
miR-4468	ENSG00000108669	7mer-m8	TargetScan
miR-4468	ENSG00000108788	7mer-m8	TargetScan
miR-4468	ENSG00000108797	7mer-m8	TargetScan
miR-4468	ENSG00000108823	7mer-m8	TargetScan
miR-4468	ENSG00000108946	7mer-m8	TargetScan
miR-4468	ENSG00000109016	7mer-m8	TargetScan
miR-4468	ENSG00000109046	7mer-m8	TargetScan
miR-4468	ENSG00000109079	7mer-m8	TargetScan
miR-4468	ENSG00000109472	7mer-m8	TargetScan
miR-4468	ENSG00000109519	7mer-m8	TargetScan
miR-4468	ENSG00000109685	7mer-m8	TargetScan
miR-4468	ENSG00000109756	7mer-m8	TargetScan
miR-4468	ENSG00000109787	7mer-m8	TargetScan
miR-4468	ENSG00000110245	7mer-m8	TargetScan
miR-4468	ENSG00000110448	7mer-m8	TargetScan

miR-4468	ENSG00000110583	7mer-m8	TargetScan
miR-4468	ENSG00000110693	7mer-m8	TargetScan
miR-4468	ENSG00000110713	7mer-m8	TargetScan
miR-4468	ENSG00000110719	7mer-m8	TargetScan
miR-4468	ENSG00000110851	7mer-m8	TargetScan
miR-4468	ENSG00000111266	7mer-m8	TargetScan
miR-4468	ENSG00000111371	7mer-m8	TargetScan
miR-4468	ENSG00000111452	7mer-m8	TargetScan
miR-4468	ENSG00000111490	7mer-m8	TargetScan
miR-4468	ENSG00000111615	7mer-m8	TargetScan
miR-4468	ENSG00000111696	7mer-m8	TargetScan
miR-4468	ENSG00000111725	7mer-m8	TargetScan
miR-4468	ENSG00000111897	7mer-m8	TargetScan
miR-4468	ENSG00000112182	7mer-m8	TargetScan
miR-4468	ENSG00000112276	7mer-m8	TargetScan
miR-4468	ENSG00000112339	7mer-m8	TargetScan
miR-4468	ENSG00000112531	7mer-m8	TargetScan
miR-4468	ENSG00000112576	7mer-m8	TargetScan
miR-4468	ENSG00000112759	7mer-m8	TargetScan
miR-4468	ENSG00000112763	7mer-m8	TargetScan
miR-4468	ENSG00000112769	7mer-m8	TargetScan
miR-4468	ENSG00000112773	7mer-m8	TargetScan
miR-4468	ENSG00000112977	7mer-m8	TargetScan
miR-4468	ENSG00000112996	7mer-m8	TargetScan
miR-4468	ENSG00000113300	7mer-m8	TargetScan
miR-4468	ENSG00000113318	7mer-m8	TargetScan
miR-4468	ENSG00000113319	7mer-m8	TargetScan
miR-4468	ENSG00000113361	7mer-m8	TargetScan
miR-4468	ENSG00000113719	7mer-m8	TargetScan
miR-4468	ENSG00000114054	7mer-m8	TargetScan
miR-4468	ENSG00000114316	7mer-m8	TargetScan

miR-4468	ENSG00000114391	7mer-m8	TargetScan
miR-4468	ENSG00000114491	7mer-m8	TargetScan
miR-4468	ENSG00000114738	7mer-m8	TargetScan
miR-4468	ENSG00000114857	7mer-m8	TargetScan
miR-4468	ENSG00000115165	7mer-m8	TargetScan
miR-4468	ENSG00000115325	7mer-m8	TargetScan
miR-4468	ENSG00000115361	7mer-m8	TargetScan
miR-4468	ENSG00000115364	7mer-m8	TargetScan
miR-4468	ENSG00000115461	7mer-m8	TargetScan
miR-4468	ENSG00000115514	7mer-m8	TargetScan
miR-4468	ENSG00000115524	7mer-m8	TargetScan
miR-4468	ENSG00000115548	7mer-m8	TargetScan
miR-4468	ENSG00000115561	7mer-m8	TargetScan
miR-4468	ENSG00000115602	7mer-m8	TargetScan
miR-4468	ENSG00000115694	7mer-m8	TargetScan
miR-4468	ENSG00000115705	7mer-m8	TargetScan
miR-4468	ENSG00000115758	7mer-m8	TargetScan
miR-4468	ENSG00000115808	7mer-m8	TargetScan
miR-4468	ENSG00000116132	7mer-m8	TargetScan
miR-4468	ENSG00000116198	7mer-m8	TargetScan
miR-4468	ENSG00000116209	7mer-m8	TargetScan
miR-4468	ENSG00000116260	7mer-m8	TargetScan
miR-4468	ENSG00000116299	7mer-m8	TargetScan
miR-4468	ENSG00000116337	7mer-m8	TargetScan
miR-4468	ENSG00000116459	7mer-m8	TargetScan
miR-4468	ENSG00000116489	7mer-m8	TargetScan
miR-4468	ENSG00000116667	7mer-m8	TargetScan
miR-4468	ENSG00000116981	7mer-m8	TargetScan
miR-4468	ENSG00000117020	7mer-m8	TargetScan
miR-4468	ENSG00000117122	7mer-m8	TargetScan
miR-4468	ENSG00000117245	7mer-m8	TargetScan

miR-4468	ENSG00000117318	7mer-m8	TargetScan
miR-4468	ENSG00000117385	7mer-m8	TargetScan
miR-4468	ENSG00000117569	7mer-m8	TargetScan
miR-4468	ENSG00000117868	7mer-m8	TargetScan
miR-4468	ENSG00000118418	7mer-m8	TargetScan
miR-4468	ENSG00000118454	7mer-m8	TargetScan
miR-4468	ENSG00000118496	7mer-m8	TargetScan
miR-4468	ENSG00000118523	7mer-m8	TargetScan
miR-4468	ENSG00000118705	7mer-m8	TargetScan
miR-4468	ENSG00000118849	7mer-m8	TargetScan
miR-4468	ENSG00000118898	7mer-m8	TargetScan
miR-4468	ENSG00000119314	7mer-m8	TargetScan
miR-4468	ENSG00000119335	7mer-m8	TargetScan
miR-4468	ENSG00000119446	7mer-m8	TargetScan
miR-4468	ENSG00000119547	7mer-m8	TargetScan
miR-4468	ENSG00000119685	7mer-m8	TargetScan
miR-4468	ENSG00000119711	7mer-m8	TargetScan
miR-4468	ENSG00000119737	7mer-m8	TargetScan
miR-4468	ENSG00000120693	7mer-m8	TargetScan
miR-4468	ENSG00000120907	7mer-m8	TargetScan
miR-4468	ENSG00000120963	7mer-m8	TargetScan
miR-4468	ENSG00000121413	7mer-m8	TargetScan
miR-4468	ENSG00000121741	7mer-m8	TargetScan
miR-4468	ENSG00000121904	7mer-m8	TargetScan
miR-4468	ENSG00000122012	7mer-m8	TargetScan
miR-4468	ENSG00000122254	7mer-m8	TargetScan
miR-4468	ENSG00000122490	7mer-m8	TargetScan
miR-4468	ENSG00000122547	7mer-m8	TargetScan
miR-4468	ENSG00000122729	7mer-m8	TargetScan
miR-4468	ENSG00000122863	7mer-m8	TargetScan
miR-4468	ENSG00000122965	7mer-m8	TargetScan

miR-4468	ENSG00000123119	7mer-m8	TargetScan
miR-4468	ENSG00000123213	7mer-m8	TargetScan
miR-4468	ENSG00000123342	7mer-m8	TargetScan
miR-4468	ENSG00000123570	7mer-m8	TargetScan
miR-4468	ENSG00000124193	7mer-m8	TargetScan
miR-4468	ENSG00000124203	7mer-m8	TargetScan
miR-4468	ENSG00000124334	7mer-m8	TargetScan
miR-4468	ENSG00000124334	7mer-m8	TargetScan
miR-4468	ENSG00000124486	7mer-m8	TargetScan
miR-4468	ENSG00000124535	7mer-m8	TargetScan
miR-4468	ENSG00000124635	7mer-m8	TargetScan
miR-4468	ENSG00000124780	7mer-m8	TargetScan
miR-4468	ENSG00000124788	7mer-m8	TargetScan
miR-4468	ENSG00000125246	7mer-m8	TargetScan
miR-4468	ENSG00000125319	7mer-m8	TargetScan
miR-4468	ENSG00000125354	7mer-m8	TargetScan
miR-4468	ENSG00000125484	7mer-m8	TargetScan
miR-4468	ENSG00000125810	7mer-m8	TargetScan
miR-4468	ENSG00000125812	7mer-m8	TargetScan
miR-4468	ENSG00000125818	7mer-m8	TargetScan
miR-4468	ENSG00000125845	7mer-m8	TargetScan
miR-4468	ENSG00000126215	7mer-m8	TargetScan
miR-4468	ENSG00000126561	7mer-m8	TargetScan
miR-4468	ENSG00000126822	7mer-m8	TargetScan
miR-4468	ENSG00000127184	7mer-m8	TargetScan
miR-4468	ENSG00000127191	7mer-m8	TargetScan
miR-4468	ENSG00000127527	7mer-m8	TargetScan
miR-4468	ENSG00000127554	7mer-m8	TargetScan
miR-4468	ENSG00000127838	7mer-m8	TargetScan
miR-4468	ENSG00000128340	7mer-m8	TargetScan
miR-4468	ENSG00000128915	7mer-m8	TargetScan

miR-4468	ENSG00000128944	7mer-m8	TargetScan
miR-4468	ENSG00000129007	7mer-m8	TargetScan
miR-4468	ENSG00000129195	7mer-m8	TargetScan
miR-4468	ENSG00000129353	7mer-m8	TargetScan
miR-4468	ENSG00000129422	7mer-m8	TargetScan
miR-4468	ENSG00000129933	7mer-m8	TargetScan
miR-4468	ENSG00000129968	7mer-m8	TargetScan
miR-4468	ENSG00000130270	7mer-m8	TargetScan
miR-4468	ENSG00000130517	7mer-m8	TargetScan
miR-4468	ENSG00000130520	7mer-m8	TargetScan
miR-4468	ENSG00000130560	7mer-m8	TargetScan
miR-4468	ENSG00000130720	7mer-m8	TargetScan
miR-4468	ENSG00000130826	7mer-m8	TargetScan
miR-4468	ENSG00000130958	7mer-m8	TargetScan
miR-4468	ENSG00000131471	7mer-m8	TargetScan
miR-4468	ENSG00000131724	7mer-m8	TargetScan
miR-4468	ENSG00000131873	7mer-m8	TargetScan
miR-4468	ENSG00000131899	7mer-m8	TargetScan
miR-4468	ENSG00000132164	7mer-m8	TargetScan
miR-4468	ENSG00000132275	7mer-m8	TargetScan
miR-4468	ENSG00000132330	7mer-m8	TargetScan
miR-4468	ENSG00000132563	7mer-m8	TargetScan
miR-4468	ENSG00000132640	7mer-m8	TargetScan
miR-4468	ENSG00000132694	7mer-m8	TargetScan
miR-4468	ENSG00000132740	7mer-m8	TargetScan
miR-4468	ENSG00000132749	7mer-m8	TargetScan
miR-4468	ENSG00000132823	7mer-m8	TargetScan
miR-4468	ENSG00000132849	7mer-m8	TargetScan
miR-4468	ENSG00000132854	7mer-m8	TargetScan
miR-4468	ENSG00000132906	7mer-m8	TargetScan
miR-4468	ENSG00000132952	7mer-m8	TargetScan

miR-4468	ENSG00000132963	7mer-m8	TargetScan
miR-4468	ENSG00000133083	7mer-m8	TargetScan
miR-4468	ENSG00000133313	7mer-m8	TargetScan
miR-4468	ENSG00000133321	7mer-m8	TargetScan
miR-4468	ENSG00000133704	7mer-m8	TargetScan
miR-4468	ENSG00000133731	7mer-m8	TargetScan
miR-4468	ENSG00000133983	7mer-m8	TargetScan
miR-4468	ENSG00000134042	7mer-m8	TargetScan
miR-4468	ENSG00000134061	7mer-m8	TargetScan
miR-4468	ENSG00000134086	7mer-m8	TargetScan
miR-4468	ENSG00000134186	7mer-m8	TargetScan
miR-4468	ENSG00000134193	7mer-m8	TargetScan
miR-4468	ENSG00000134245	7mer-m8	TargetScan
miR-4468	ENSG00000134253	7mer-m8	TargetScan
miR-4468	ENSG00000134365	7mer-m8	TargetScan
miR-4468	ENSG00000134440	7mer-m8	TargetScan
miR-4468	ENSG00000134551	7mer-m8	TargetScan
miR-4468	ENSG00000134716	7mer-m8	TargetScan
miR-4468	ENSG00000134762	7mer-m8	TargetScan
miR-4468	ENSG00000134779	7mer-m8	TargetScan
miR-4468	ENSG00000134809	7mer-m8	TargetScan
miR-4468	ENSG00000134897	7mer-m8	TargetScan
miR-4468	ENSG00000134962	7mer-m8	TargetScan
miR-4468	ENSG00000134987	7mer-m8	TargetScan
miR-4468	ENSG00000135049	7mer-m8	TargetScan
miR-4468	ENSG00000135097	7mer-m8	TargetScan
miR-4468	ENSG00000135316	7mer-m8	TargetScan
miR-4468	ENSG00000135373	7mer-m8	TargetScan
miR-4468	ENSG00000135472	7mer-m8	TargetScan
miR-4468	ENSG00000135604	7mer-m8	TargetScan
miR-4468	ENSG00000135823	7mer-m8	TargetScan

miR-4468	ENSG00000135824	7mer-m8	TargetScan
miR-4468	ENSG00000135953	7mer-m8	TargetScan
miR-4468	ENSG00000135999	7mer-m8	TargetScan
miR-4468	ENSG00000136169	7mer-m8	TargetScan
miR-4468	ENSG00000136261	7mer-m8	TargetScan
miR-4468	ENSG00000136279	7mer-m8	TargetScan
miR-4468	ENSG00000136448	7mer-m8	TargetScan
miR-4468	ENSG00000136504	7mer-m8	TargetScan
miR-4468	ENSG00000136731	7mer-m8	TargetScan
miR-4468	ENSG00000136908	7mer-m8	TargetScan
miR-4468	ENSG00000136925	7mer-m8	TargetScan
miR-4468	ENSG00000136944	7mer-m8	TargetScan
miR-4468	ENSG00000137055	7mer-m8	TargetScan
miR-4468	ENSG00000137070	7mer-m8	TargetScan
miR-4468	ENSG00000137185	7mer-m8	TargetScan
miR-4468	ENSG00000137204	7mer-m8	TargetScan
miR-4468	ENSG00000137265	7mer-m8	TargetScan
miR-4468	ENSG00000137414	7mer-m8	TargetScan
miR-4468	ENSG00000137486	7mer-m8	TargetScan
miR-4468	ENSG00000137496	7mer-m8	TargetScan
miR-4468	ENSG00000137502	7mer-m8	TargetScan
miR-4468	ENSG00000137992	7mer-m8	TargetScan
miR-4468	ENSG00000138160	7mer-m8	TargetScan
miR-4468	ENSG00000138286	7mer-m8	TargetScan
miR-4468	ENSG00000138311	7mer-m8	TargetScan
miR-4468	ENSG00000138347	7mer-m8	TargetScan
miR-4468	ENSG00000138443	7mer-m8	TargetScan
miR-4468	ENSG00000138463	7mer-m8	TargetScan
miR-4468	ENSG00000138622	7mer-m8	TargetScan
miR-4468	ENSG00000138670	7mer-m8	TargetScan
miR-4468	ENSG00000138741	7mer-m8	TargetScan

miR-4468	ENSG00000138835	7mer-m8	TargetScan
miR-4468	ENSG00000138867	7mer-m8	TargetScan
miR-4468	ENSG00000139116	7mer-m8	TargetScan
miR-4468	ENSG00000139190	7mer-m8	TargetScan
miR-4468	ENSG00000139197	7mer-m8	TargetScan
miR-4468	ENSG00000139329	7mer-m8	TargetScan
miR-4468	ENSG00000139722	7mer-m8	TargetScan
miR-4468	ENSG00000140009	7mer-m8	TargetScan
miR-4468	ENSG00000140262	7mer-m8	TargetScan
miR-4468	ENSG00000140464	7mer-m8	TargetScan
miR-4468	ENSG00000140471	7mer-m8	TargetScan
miR-4468	ENSG00000140527	7mer-m8	TargetScan
miR-4468	ENSG00000140548	7mer-m8	TargetScan
miR-4468	ENSG00000140688	7mer-m8	TargetScan
miR-4468	ENSG00000140807	7mer-m8	TargetScan
miR-4468	ENSG00000140948	7mer-m8	TargetScan
miR-4468	ENSG00000141076	7mer-m8	TargetScan
miR-4468	ENSG00000141219	7mer-m8	TargetScan
miR-4468	ENSG00000141252	7mer-m8	TargetScan
miR-4468	ENSG00000141298	7mer-m8	TargetScan
miR-4468	ENSG00000141337	7mer-m8	TargetScan
miR-4468	ENSG00000141441	7mer-m8	TargetScan
miR-4468	ENSG00000141560	7mer-m8	TargetScan
miR-4468	ENSG00000141622	7mer-m8	TargetScan
miR-4468	ENSG00000141759	7mer-m8	TargetScan
miR-4468	ENSG00000141965	7mer-m8	TargetScan
miR-4468	ENSG00000142166	7mer-m8	TargetScan
miR-4468	ENSG00000142556	7mer-m8	TargetScan
miR-4468	ENSG00000142623	7mer-m8	TargetScan
miR-4468	ENSG00000143178	7mer-m8	TargetScan
miR-4468	ENSG00000143297	7mer-m8	TargetScan

miR-4468	ENSG00000143498	7mer-m8	TargetScan
miR-4468	ENSG00000143537	7mer-m8	TargetScan
miR-4468	ENSG00000143595	7mer-m8	TargetScan
miR-4468	ENSG00000143603	7mer-m8	TargetScan
miR-4468	ENSG00000143627	7mer-m8	TargetScan
miR-4468	ENSG00000143771	7mer-m8	TargetScan
miR-4468	ENSG00000143889	7mer-m8	TargetScan
miR-4468	ENSG00000143921	7mer-m8	TargetScan
miR-4468	ENSG00000144320	7mer-m8	TargetScan
miR-4468	ENSG00000144580	7mer-m8	TargetScan
miR-4468	ENSG00000144645	7mer-m8	TargetScan
miR-4468	ENSG00000144711	7mer-m8	TargetScan
miR-4468	ENSG00000145214	7mer-m8	TargetScan
miR-4468	ENSG00000145287	7mer-m8	TargetScan
miR-4468	ENSG00000145391	7mer-m8	TargetScan
miR-4468	ENSG00000145439	7mer-m8	TargetScan
miR-4468	ENSG00000145555	7mer-m8	TargetScan
miR-4468	ENSG00000145685	7mer-m8	TargetScan
miR-4468	ENSG00000145725	7mer-m8	TargetScan
miR-4468	ENSG00000145850	7mer-m8	TargetScan
miR-4468	ENSG00000145861	7mer-m8	TargetScan
miR-4468	ENSG00000145920	7mer-m8	TargetScan
miR-4468	ENSG00000146063	7mer-m8	TargetScan
miR-4468	ENSG00000146147	7mer-m8	TargetScan
miR-4468	ENSG00000146216	7mer-m8	TargetScan
miR-4468	ENSG00000146267	7mer-m8	TargetScan
miR-4468	ENSG00000146535	7mer-m8	TargetScan
miR-4468	ENSG00000146648	7mer-m8	TargetScan
miR-4468	ENSG00000146670	7mer-m8	TargetScan
miR-4468	ENSG00000146757	7mer-m8	TargetScan
miR-4468	ENSG00000146859	7mer-m8	TargetScan

miR-4468	ENSG00000147117	7mer-m8	TargetScan
miR-4468	ENSG00000147138	7mer-m8	TargetScan
miR-4468	ENSG00000147234	7mer-m8	TargetScan
miR-4468	ENSG00000147246	7mer-m8	TargetScan
miR-4468	ENSG00000147394	7mer-m8	TargetScan
miR-4468	ENSG00000147535	7mer-m8	TargetScan
miR-4468	ENSG00000147548	7mer-m8	TargetScan
miR-4468	ENSG00000147649	7mer-m8	TargetScan
miR-4468	ENSG00000148082	7mer-m8	TargetScan
miR-4468	ENSG00000148110	7mer-m8	TargetScan
miR-4468	ENSG00000148120	7mer-m8	TargetScan
miR-4468	ENSG00000148158	7mer-m8	TargetScan
miR-4468	ENSG00000148248	7mer-m8	TargetScan
miR-4468	ENSG00000148634	7mer-m8	TargetScan
miR-4468	ENSG00000148688	7mer-m8	TargetScan
miR-4468	ENSG00000148734	7mer-m8	TargetScan
miR-4468	ENSG00000148798	7mer-m8	TargetScan
miR-4468	ENSG00000148942	7mer-m8	TargetScan
miR-4468	ENSG00000149196	7mer-m8	TargetScan
miR-4468	ENSG00000149212	7mer-m8	TargetScan
miR-4468	ENSG00000149218	7mer-m8	TargetScan
miR-4468	ENSG00000149256	7mer-m8	TargetScan
miR-4468	ENSG00000149257	7mer-m8	TargetScan
miR-4468	ENSG00000149346	7mer-m8	TargetScan
miR-4468	ENSG00000149507	7mer-m8	TargetScan
miR-4468	ENSG00000149596	7mer-m8	TargetScan
miR-4468	ENSG00000149782	7mer-m8	TargetScan
miR-4468	ENSG00000149925	7mer-m8	TargetScan
miR-4468	ENSG00000149927	7mer-m8	TargetScan
miR-4468	ENSG00000150457	7mer-m8	TargetScan
miR-4468	ENSG00000151090	7mer-m8	TargetScan

miR-4468	ENSG00000151164	7mer-m8	TargetScan
miR-4468	ENSG00000151176	7mer-m8	TargetScan
miR-4468	ENSG00000151553	7mer-m8	TargetScan
miR-4468	ENSG00000151914	7mer-m8	TargetScan
miR-4468	ENSG00000152078	7mer-m8	TargetScan
miR-4468	ENSG00000152102	7mer-m8	TargetScan
miR-4468	ENSG00000152270	7mer-m8	TargetScan
miR-4468	ENSG00000152284	7mer-m8	TargetScan
miR-4468	ENSG00000152404	7mer-m8	TargetScan
miR-4468	ENSG00000152443	7mer-m8	TargetScan
miR-4468	ENSG00000152580	7mer-m8	TargetScan
miR-4468	ENSG00000152689	7mer-m8	TargetScan
miR-4468	ENSG00000152932	7mer-m8	TargetScan
miR-4468	ENSG00000152953	7mer-m8	TargetScan
miR-4468	ENSG00000153012	7mer-m8	TargetScan
miR-4468	ENSG00000153233	7mer-m8	TargetScan
miR-4468	ENSG00000153303	7mer-m8	TargetScan
miR-4468	ENSG00000153904	7mer-m8	TargetScan
miR-4468	ENSG00000154001	7mer-m8	TargetScan
miR-4468	ENSG00000154124	7mer-m8	TargetScan
miR-4468	ENSG00000154342	7mer-m8	TargetScan
miR-4468	ENSG00000154511	7mer-m8	TargetScan
miR-4468	ENSG00000154582	7mer-m8	TargetScan
miR-4468	ENSG00000154655	7mer-m8	TargetScan
miR-4468	ENSG00000154678	7mer-m8	TargetScan
miR-4468	ENSG00000155008	7mer-m8	TargetScan
miR-4468	ENSG00000155034	7mer-m8	TargetScan
miR-4468	ENSG00000155066	7mer-m8	TargetScan
miR-4468	ENSG00000155111	7mer-m8	TargetScan
miR-4468	ENSG00000155115	7mer-m8	TargetScan
miR-4468	ENSG00000155158	7mer-m8	TargetScan

miR-4468	ENSG00000155438	7mer-m8	TargetScan
miR-4468	ENSG00000155506	7mer-m8	TargetScan
miR-4468	ENSG00000155542	7mer-m8	TargetScan
miR-4468	ENSG00000155816	7mer-m8	TargetScan
miR-4468	ENSG00000155827	7mer-m8	TargetScan
miR-4468	ENSG00000155833	7mer-m8	TargetScan
miR-4468	ENSG00000155886	7mer-m8	TargetScan
miR-4468	ENSG00000155926	7mer-m8	TargetScan
miR-4468	ENSG00000156011	7mer-m8	TargetScan
miR-4468	ENSG00000156172	7mer-m8	TargetScan
miR-4468	ENSG00000156500	7mer-m8	TargetScan
miR-4468	ENSG00000156873	7mer-m8	TargetScan
miR-4468	ENSG00000156928	7mer-m8	TargetScan
miR-4468	ENSG00000157036	7mer-m8	TargetScan
miR-4468	ENSG00000157150	7mer-m8	TargetScan
miR-4468	ENSG00000157326	7mer-m8	TargetScan
miR-4468	ENSG00000157388	7mer-m8	TargetScan
miR-4468	ENSG00000157483	7mer-m8	TargetScan
miR-4468	ENSG00000157542	7mer-m8	TargetScan
miR-4468	ENSG00000157978	7mer-m8	TargetScan
miR-4468	ENSG00000158022	7mer-m8	TargetScan
miR-4468	ENSG00000158109	7mer-m8	TargetScan
miR-4468	ENSG00000158161	7mer-m8	TargetScan
miR-4468	ENSG00000158258	7mer-m8	TargetScan
miR-4468	ENSG00000158427	7mer-m8	TargetScan
miR-4468	ENSG00000158467	7mer-m8	TargetScan
miR-4468	ENSG00000158473	7mer-m8	TargetScan
miR-4468	ENSG00000158711	7mer-m8	TargetScan
miR-4468	ENSG00000158828	7mer-m8	TargetScan
miR-4468	ENSG00000158955	7mer-m8	TargetScan
miR-4468	ENSG00000158985	7mer-m8	TargetScan

miR-4468	ENSG00000159140	7mer-m8	TargetScan
miR-4468	ENSG00000159788	7mer-m8	TargetScan
miR-4468	ENSG00000159882	7mer-m8	TargetScan
miR-4468	ENSG00000159885	7mer-m8	TargetScan
miR-4468	ENSG00000160049	7mer-m8	TargetScan
miR-4468	ENSG00000160058	7mer-m8	TargetScan
miR-4468	ENSG00000160208	7mer-m8	TargetScan
miR-4468	ENSG00000160209	7mer-m8	TargetScan
miR-4468	ENSG00000160223	7mer-m8	TargetScan
miR-4468	ENSG00000160293	7mer-m8	TargetScan
miR-4468	ENSG00000160325	7mer-m8	TargetScan
miR-4468	ENSG00000160339	7mer-m8	TargetScan
miR-4468	ENSG00000160716	7mer-m8	TargetScan
miR-4468	ENSG00000160781	7mer-m8	TargetScan
miR-4468	ENSG00000160908	7mer-m8	TargetScan
miR-4468	ENSG00000161010	7mer-m8	TargetScan
miR-4468	ENSG00000161204	7mer-m8	TargetScan
miR-4468	ENSG00000161381	7mer-m8	TargetScan
miR-4468	ENSG00000161791	7mer-m8	TargetScan
miR-4468	ENSG00000162065	7mer-m8	TargetScan
miR-4468	ENSG00000162510	7mer-m8	TargetScan
miR-4468	ENSG00000162522	7mer-m8	TargetScan
miR-4468	ENSG00000162552	7mer-m8	TargetScan
miR-4468	ENSG00000162571	7mer-m8	TargetScan
miR-4468	ENSG00000162607	7mer-m8	TargetScan
miR-4468	ENSG00000162631	7mer-m8	TargetScan
miR-4468	ENSG00000162645	7mer-m8	TargetScan
miR-4468	ENSG00000162733	7mer-m8	TargetScan
miR-4468	ENSG00000162738	7mer-m8	TargetScan
miR-4468	ENSG00000162804	7mer-m8	TargetScan
miR-4468	ENSG00000162888	7mer-m8	TargetScan

miR-4468	ENSG00000162889	7mer-m8	TargetScan
miR-4468	ENSG00000162924	7mer-m8	TargetScan
miR-4468	ENSG00000162929	7mer-m8	TargetScan
miR-4468	ENSG00000163138	7mer-m8	TargetScan
miR-4468	ENSG00000163145	7mer-m8	TargetScan
miR-4468	ENSG00000163155	7mer-m8	TargetScan
miR-4468	ENSG00000163393	7mer-m8	TargetScan
miR-4468	ENSG00000163412	7mer-m8	TargetScan
miR-4468	ENSG00000163513	7mer-m8	TargetScan
miR-4468	ENSG00000163586	7mer-m8	TargetScan
miR-4468	ENSG00000163590	7mer-m8	TargetScan
miR-4468	ENSG00000163673	7mer-m8	TargetScan
miR-4468	ENSG00000163870	7mer-m8	TargetScan
miR-4468	ENSG00000163888	7mer-m8	TargetScan
miR-4468	ENSG00000163909	7mer-m8	TargetScan
miR-4468	ENSG00000164007	7mer-m8	TargetScan
miR-4468	ENSG00000164038	7mer-m8	TargetScan
miR-4468	ENSG00000164061	7mer-m8	TargetScan
miR-4468	ENSG00000164070	7mer-m8	TargetScan
miR-4468	ENSG00000164076	7mer-m8	TargetScan
miR-4468	ENSG00000164091	7mer-m8	TargetScan
miR-4468	ENSG00000164114	7mer-m8	TargetScan
miR-4468	ENSG00000164116	7mer-m8	TargetScan
miR-4468	ENSG00000164120	7mer-m8	TargetScan
miR-4468	ENSG00000164241	7mer-m8	TargetScan
miR-4468	ENSG00000164393	7mer-m8	TargetScan
miR-4468	ENSG00000164402	7mer-m8	TargetScan
miR-4468	ENSG00000164574	7mer-m8	TargetScan
miR-4468	ENSG00000164742	7mer-m8	TargetScan
miR-4468	ENSG00000164751	7mer-m8	TargetScan
miR-4468	ENSG00000164916	7mer-m8	TargetScan

miR-4468	ENSG00000164944	7mer-m8	TargetScan
miR-4468	ENSG00000164970	7mer-m8	TargetScan
miR-4468	ENSG00000165028	7mer-m8	TargetScan
miR-4468	ENSG00000165029	7mer-m8	TargetScan
miR-4468	ENSG00000165238	7mer-m8	TargetScan
miR-4468	ENSG00000165240	7mer-m8	TargetScan
miR-4468	ENSG00000165269	7mer-m8	TargetScan
miR-4468	ENSG00000165288	7mer-m8	TargetScan
miR-4468	ENSG00000165410	7mer-m8	TargetScan
miR-4468	ENSG00000165568	7mer-m8	TargetScan
miR-4468	ENSG00000165650	7mer-m8	TargetScan
miR-4468	ENSG00000165671	7mer-m8	TargetScan
miR-4468	ENSG00000165886	7mer-m8	TargetScan
miR-4468	ENSG00000166012	7mer-m8	TargetScan
miR-4468	ENSG00000166049	7mer-m8	TargetScan
miR-4468	ENSG00000166105	7mer-m8	TargetScan
miR-4468	ENSG00000166145	7mer-m8	TargetScan
miR-4468	ENSG00000166147	7mer-m8	TargetScan
miR-4468	ENSG00000166159	7mer-m8	TargetScan
miR-4468	ENSG00000166233	7mer-m8	TargetScan
miR-4468	ENSG00000166265	7mer-m8	TargetScan
miR-4468	ENSG00000166295	7mer-m8	TargetScan
miR-4468	ENSG00000166326	7mer-m8	TargetScan
miR-4468	ENSG00000166347	7mer-m8	TargetScan
miR-4468	ENSG00000166401	7mer-m8	TargetScan
miR-4468	ENSG00000166432	7mer-m8	TargetScan
miR-4468	ENSG00000166557	7mer-m8	TargetScan
miR-4468	ENSG00000166598	7mer-m8	TargetScan
miR-4468	ENSG00000166685	7mer-m8	TargetScan
miR-4468	ENSG00000166823	7mer-m8	TargetScan
miR-4468	ENSG00000166847	7mer-m8	TargetScan

miR-4468	ENSG00000166887	7mer-m8	TargetScan
miR-4468	ENSG00000166923	7mer-m8	TargetScan
miR-4468	ENSG00000166946	7mer-m8	TargetScan
miR-4468	ENSG00000166961	7mer-m8	TargetScan
miR-4468	ENSG00000167004	7mer-m8	TargetScan
miR-4468	ENSG00000167005	7mer-m8	TargetScan
miR-4468	ENSG00000167074	7mer-m8	TargetScan
miR-4468	ENSG00000167106	7mer-m8	TargetScan
miR-4468	ENSG00000167283	7mer-m8	TargetScan
miR-4468	ENSG00000167380	7mer-m8	TargetScan
miR-4468	ENSG00000167770	7mer-m8	TargetScan
miR-4468	ENSG00000167858	7mer-m8	TargetScan
miR-4468	ENSG00000167968	7mer-m8	TargetScan
miR-4468	ENSG00000167971	7mer-m8	TargetScan
miR-4468	ENSG00000168067	7mer-m8	TargetScan
miR-4468	ENSG00000168214	7mer-m8	TargetScan
miR-4468	ENSG00000168228	7mer-m8	TargetScan
miR-4468	ENSG00000168234	7mer-m8	TargetScan
miR-4468	ENSG00000168314	7mer-m8	TargetScan
miR-4468	ENSG00000168418	7mer-m8	TargetScan
miR-4468	ENSG00000168461	7mer-m8	TargetScan
miR-4468	ENSG00000168481	7mer-m8	TargetScan
miR-4468	ENSG00000168487	7mer-m8	TargetScan
miR-4468	ENSG00000168612	7mer-m8	TargetScan
miR-4468	ENSG00000168758	7mer-m8	TargetScan
miR-4468	ENSG00000168785	7mer-m8	TargetScan
miR-4468	ENSG00000168792	7mer-m8	TargetScan
miR-4468	ENSG00000168827	7mer-m8	TargetScan
miR-4468	ENSG00000168887	7mer-m8	TargetScan
miR-4468	ENSG00000168994	7mer-m8	TargetScan
miR-4468	ENSG00000169188	7mer-m8	TargetScan

miR-4468	ENSG00000169247	7mer-m8	TargetScan
miR-4468	ENSG00000169291	7mer-m8	TargetScan
miR-4468	ENSG00000169302	7mer-m8	TargetScan
miR-4468	ENSG00000169403	7mer-m8	TargetScan
miR-4468	ENSG00000169594	7mer-m8	TargetScan
miR-4468	ENSG00000169641	7mer-m8	TargetScan
miR-4468	ENSG00000169679	7mer-m8	TargetScan
miR-4468	ENSG00000169692	7mer-m8	TargetScan
miR-4468	ENSG00000169814	7mer-m8	TargetScan
miR-4468	ENSG00000169981	7mer-m8	TargetScan
miR-4468	ENSG00000170100	7mer-m8	TargetScan
miR-4468	ENSG00000170113	7mer-m8	TargetScan
miR-4468	ENSG00000170180	7mer-m8	TargetScan
miR-4468	ENSG00000170222	7mer-m8	TargetScan
miR-4468	ENSG00000170464	7mer-m8	TargetScan
miR-4468	ENSG00000170522	7mer-m8	TargetScan
miR-4468	ENSG00000170558	7mer-m8	TargetScan
miR-4468	ENSG00000170624	7mer-m8	TargetScan
miR-4468	ENSG00000170632	7mer-m8	TargetScan
miR-4468	ENSG00000170634	7mer-m8	TargetScan
miR-4468	ENSG00000170653	7mer-m8	TargetScan
miR-4468	ENSG00000170734	7mer-m8	TargetScan
miR-4468	ENSG00000170743	7mer-m8	TargetScan
miR-4468	ENSG00000170748	7mer-m8	TargetScan
miR-4468	ENSG00000170854	7mer-m8	TargetScan
miR-4468	ENSG00000170903	7mer-m8	TargetScan
miR-4468	ENSG00000171004	7mer-m8	TargetScan
miR-4468	ENSG00000171033	7mer-m8	TargetScan
miR-4468	ENSG00000171262	7mer-m8	TargetScan
miR-4468	ENSG00000171320	7mer-m8	TargetScan
miR-4468	ENSG00000171365	7mer-m8	TargetScan

miR-4468	ENSG00000171451	7mer-m8	TargetScan
miR-4468	ENSG00000171496	7mer-m8	TargetScan
miR-4468	ENSG00000171621	7mer-m8	TargetScan
miR-4468	ENSG00000172058	7mer-m8	TargetScan
miR-4468	ENSG00000172339	7mer-m8	TargetScan
miR-4468	ENSG00000172500	7mer-m8	TargetScan
miR-4468	ENSG00000172508	7mer-m8	TargetScan
miR-4468	ENSG00000172671	7mer-m8	TargetScan
miR-4468	ENSG00000172687	7mer-m8	TargetScan
miR-4468	ENSG00000172818	7mer-m8	TargetScan
miR-4468	ENSG00000172830	7mer-m8	TargetScan
miR-4468	ENSG00000172987	7mer-m8	TargetScan
miR-4468	ENSG00000173064	7mer-m8	TargetScan
miR-4468	ENSG00000173065	7mer-m8	TargetScan
miR-4468	ENSG00000173157	7mer-m8	TargetScan
miR-4468	ENSG00000173218	7mer-m8	TargetScan
miR-4468	ENSG00000173227	7mer-m8	TargetScan
miR-4468	ENSG00000173281	7mer-m8	TargetScan
miR-4468	ENSG00000173548	7mer-m8	TargetScan
miR-4468	ENSG00000173559	7mer-m8	TargetScan
miR-4468	ENSG00000173706	7mer-m8	TargetScan
miR-4468	ENSG00000173889	7mer-m8	TargetScan
miR-4468	ENSG00000174099	7mer-m8	TargetScan
miR-4468	ENSG00000174151	7mer-m8	TargetScan
miR-4468	ENSG00000174197	7mer-m8	TargetScan
miR-4468	ENSG00000174498	7mer-m8	TargetScan
miR-4468	ENSG00000174574	7mer-m8	TargetScan
miR-4468	ENSG00000174705	7mer-m8	TargetScan
miR-4468	ENSG00000174748	7mer-m8	TargetScan
miR-4468	ENSG00000174840	7mer-m8	TargetScan
miR-4468	ENSG00000174885	7mer-m8	TargetScan

miR-4468	ENSG00000174903	7mer-m8	TargetScan
miR-4468	ENSG00000174953	7mer-m8	TargetScan
miR-4468	ENSG00000175029	7mer-m8	TargetScan
miR-4468	ENSG00000175106	7mer-m8	TargetScan
miR-4468	ENSG00000175216	7mer-m8	TargetScan
miR-4468	ENSG00000175497	7mer-m8	TargetScan
miR-4468	ENSG00000175556	7mer-m8	TargetScan
miR-4468	ENSG00000175728	7mer-m8	TargetScan
miR-4468	ENSG00000175970	7mer-m8	TargetScan
miR-4468	ENSG00000176014	7mer-m8	TargetScan
miR-4468	ENSG00000176209	7mer-m8	TargetScan
miR-4468	ENSG00000176406	7mer-m8	TargetScan
miR-4468	ENSG00000176641	7mer-m8	TargetScan
miR-4468	ENSG00000176658	7mer-m8	TargetScan
miR-4468	ENSG00000176723	7mer-m8	TargetScan
miR-4468	ENSG00000176890	7mer-m8	TargetScan
miR-4468	ENSG00000176945	7mer-m8	TargetScan
miR-4468	ENSG00000176973	7mer-m8	TargetScan
miR-4468	ENSG00000176994	7mer-m8	TargetScan
miR-4468	ENSG00000177096	7mer-m8	TargetScan
miR-4468	ENSG00000177324	7mer-m8	TargetScan
miR-4468	ENSG00000177426	7mer-m8	TargetScan
miR-4468	ENSG00000177479	7mer-m8	TargetScan
miR-4468	ENSG00000177511	7mer-m8	TargetScan
miR-4468	ENSG00000177646	7mer-m8	TargetScan
miR-4468	ENSG00000177663	7mer-m8	TargetScan
miR-4468	ENSG00000177842	7mer-m8	TargetScan
miR-4468	ENSG00000178053	7mer-m8	TargetScan
miR-4468	ENSG00000178233	7mer-m8	TargetScan
miR-4468	ENSG00000178381	7mer-m8	TargetScan
miR-4468	ENSG00000178385	7mer-m8	TargetScan

miR-4468	ENSG00000178460	7mer-m8	TargetScan
miR-4468	ENSG00000178538	7mer-m8	TargetScan
miR-4468	ENSG00000178562	7mer-m8	TargetScan
miR-4468	ENSG00000178662	7mer-m8	TargetScan
miR-4468	ENSG00000178695	7mer-m8	TargetScan
miR-4468	ENSG00000178726	7mer-m8	TargetScan
miR-4468	ENSG00000178997	7mer-m8	TargetScan
miR-4468	ENSG00000179151	7mer-m8	TargetScan
miR-4468	ENSG00000179195	7mer-m8	TargetScan
miR-4468	ENSG00000179361	7mer-m8	TargetScan
miR-4468	ENSG00000179583	7mer-m8	TargetScan
miR-4468	ENSG00000179588	7mer-m8	TargetScan
miR-4468	ENSG00000179698	7mer-m8	TargetScan
miR-4468	ENSG00000179833	7mer-m8	TargetScan
miR-4468	ENSG00000179886	7mer-m8	TargetScan
miR-4468	ENSG00000180228	7mer-m8	TargetScan
miR-4468	ENSG00000180287	7mer-m8	TargetScan
miR-4468	ENSG00000180354	7mer-m8	TargetScan
miR-4468	ENSG00000180357	7mer-m8	TargetScan
miR-4468	ENSG00000180432	7mer-m8	TargetScan
miR-4468	ENSG00000180479	7mer-m8	TargetScan
miR-4468	ENSG00000180530	7mer-m8	TargetScan
miR-4468	ENSG00000181016	7mer-m8	TargetScan
miR-4468	ENSG00000181381	7mer-m8	TargetScan
miR-4468	ENSG00000181722	7mer-m8	TargetScan
miR-4468	ENSG00000181873	7mer-m8	TargetScan
miR-4468	ENSG00000181894	7mer-m8	TargetScan
miR-4468	ENSG00000182141	7mer-m8	TargetScan
miR-4468	ENSG00000182489	7mer-m8	TargetScan
miR-4468	ENSG00000182667	7mer-m8	TargetScan
miR-4468	ENSG00000182749	7mer-m8	TargetScan

miR-4468	ENSG00000182768	7mer-m8	TargetScan
miR-4468	ENSG00000182898	7mer-m8	TargetScan
miR-4468	ENSG00000182919	7mer-m8	TargetScan
miR-4468	ENSG00000183250	7mer-m8	TargetScan
miR-4468	ENSG00000183260	7mer-m8	TargetScan
miR-4468	ENSG00000183287	7mer-m8	TargetScan
miR-4468	ENSG00000183379	7mer-m8	TargetScan
miR-4468	ENSG00000183527	7mer-m8	TargetScan
miR-4468	ENSG00000183578	7mer-m8	TargetScan
miR-4468	ENSG00000183624	7mer-m8	TargetScan
miR-4468	ENSG00000183778	7mer-m8	TargetScan
miR-4468	ENSG00000183850	7mer-m8	TargetScan
miR-4468	ENSG00000183943	7mer-m8	TargetScan
miR-4468	ENSG00000184005	7mer-m8	TargetScan
miR-4468	ENSG00000184083	7mer-m8	TargetScan
miR-4468	ENSG00000184304	7mer-m8	TargetScan
miR-4468	ENSG00000184347	7mer-m8	TargetScan
miR-4468	ENSG00000184378	7mer-m8	TargetScan
miR-4468	ENSG00000184497	7mer-m8	TargetScan
miR-4468	ENSG00000184661	7mer-m8	TargetScan
miR-4468	ENSG00000184863	7mer-m8	TargetScan
miR-4468	ENSG00000185008	7mer-m8	TargetScan
miR-4468	ENSG00000185104	7mer-m8	TargetScan
miR-4468	ENSG00000185105	7mer-m8	TargetScan
miR-4468	ENSG00000185187	7mer-m8	TargetScan
miR-4468	ENSG00000185404	7mer-m8	TargetScan
miR-4468	ENSG00000185442	7mer-m8	TargetScan
miR-4468	ENSG00000185651	7mer-m8	TargetScan
miR-4468	ENSG00000185658	7mer-m8	TargetScan
miR-4468	ENSG00000185722	7mer-m8	TargetScan
miR-4468	ENSG00000185811	7mer-m8	TargetScan

miR-4468	ENSG00000185838	7mer-m8	TargetScan
miR-4468	ENSG00000185862	7mer-m8	TargetScan
miR-4468	ENSG00000185875	7mer-m8	TargetScan
miR-4468	ENSG00000185905	7mer-m8	TargetScan
miR-4468	ENSG00000185920	7mer-m8	TargetScan
miR-4468	ENSG00000186001	7mer-m8	TargetScan
miR-4468	ENSG00000186260	7mer-m8	TargetScan
miR-4468	ENSG00000186340	7mer-m8	TargetScan
miR-4468	ENSG00000186432	7mer-m8	TargetScan
miR-4468	ENSG00000186472	7mer-m8	TargetScan
miR-4468	ENSG00000186479	7mer-m8	TargetScan
miR-4468	ENSG00000186529	7mer-m8	TargetScan
miR-4468	ENSG00000186562	7mer-m8	TargetScan
miR-4468	ENSG00000186575	7mer-m8	TargetScan
miR-4468	ENSG00000186599	7mer-m8	TargetScan
miR-4468	ENSG00000186812	7mer-m8	TargetScan
miR-4468	ENSG00000186815	7mer-m8	TargetScan
miR-4468	ENSG00000187257	7mer-m8	TargetScan
miR-4468	ENSG00000187272	7mer-m8	TargetScan
miR-4468	ENSG00000187550	7mer-m8	TargetScan
miR-4468	ENSG00000187569	7mer-m8	TargetScan
miR-4468	ENSG00000187626	7mer-m8	TargetScan
miR-4468	ENSG00000187630	7mer-m8	TargetScan
miR-4468	ENSG00000187678	7mer-m8	TargetScan
miR-4468	ENSG00000187758	7mer-m8	TargetScan
miR-4468	ENSG00000187902	7mer-m8	TargetScan
miR-4468	ENSG00000188001	7mer-m8	TargetScan
miR-4468	ENSG00000188026	7mer-m8	TargetScan
miR-4468	ENSG00000188177	7mer-m8	TargetScan
miR-4468	ENSG00000188295	7mer-m8	TargetScan
miR-4468	ENSG00000188690	7mer-m8	TargetScan

miR-4468	ENSG00000188706	7mer-m8	TargetScan
miR-4468	ENSG00000188817	7mer-m8	TargetScan
miR-4468	ENSG00000188848	7mer-m8	TargetScan
miR-4468	ENSG00000188883	7mer-m8	TargetScan
miR-4468	ENSG00000189221	7mer-m8	TargetScan
miR-4468	ENSG00000189403	7mer-m8	TargetScan
miR-4468	ENSG00000196090	7mer-m8	TargetScan
miR-4468	ENSG00000196091	7mer-m8	TargetScan
miR-4468	ENSG00000196208	7mer-m8	TargetScan
miR-4468	ENSG00000196247	7mer-m8	TargetScan
miR-4468	ENSG00000196268	7mer-m8	TargetScan
miR-4468	ENSG00000196376	7mer-m8	TargetScan
miR-4468	ENSG00000196705	7mer-m8	TargetScan
miR-4468	ENSG00000196715	7mer-m8	TargetScan
miR-4468	ENSG00000197121	7mer-m8	TargetScan
miR-4468	ENSG00000197261	7mer-m8	TargetScan
miR-4468	ENSG00000197302	7mer-m8	TargetScan
miR-4468	ENSG00000197536	7mer-m8	TargetScan
miR-4468	ENSG00000197714	7mer-m8	TargetScan
miR-4468	ENSG00000197818	7mer-m8	TargetScan
miR-4468	ENSG00000197965	7mer-m8	TargetScan
miR-4468	ENSG00000198039	7mer-m8	TargetScan
miR-4468	ENSG00000198053	7mer-m8	TargetScan
miR-4468	ENSG00000198083	7mer-m8	TargetScan
miR-4468	ENSG00000198363	7mer-m8	TargetScan
miR-4468	ENSG00000198408	7mer-m8	TargetScan
miR-4468	ENSG00000198483	7mer-m8	TargetScan
miR-4468	ENSG00000198625	7mer-m8	TargetScan
miR-4468	ENSG00000198663	7mer-m8	TargetScan
miR-4468	ENSG00000198677	7mer-m8	TargetScan
miR-4468	ENSG00000198689	7mer-m8	TargetScan

miR-4468	ENSG00000198768	7mer-m8	TargetScan
miR-4468	ENSG00000198855	7mer-m8	TargetScan
miR-4468	ENSG00000198863	7mer-m8	TargetScan
miR-4468	ENSG00000198865	7mer-m8	TargetScan
miR-4468	ENSG00000198883	7mer-m8	TargetScan
miR-4468	ENSG00000198944	7mer-m8	TargetScan
miR-4468	ENSG00000198948	7mer-m8	TargetScan
miR-4468	ENSG00000203778	7mer-m8	TargetScan
miR-4468	ENSG00000204228	7mer-m8	TargetScan
miR-4468	ENSG00000204308	7mer-m8	TargetScan
miR-4468	ENSG00000204345	7mer-m8	TargetScan
miR-4468	ENSG00000204420	7mer-m8	TargetScan
miR-4468	ENSG00000204438	7mer-m8	TargetScan
miR-4468	ENSG00000204604	7mer-m8	TargetScan
miR-4468	ENSG00000204613	7mer-m8	TargetScan
miR-4468	ENSG00000204681	7mer-m8	TargetScan
miR-4468	ENSG00000204839	7mer-m8	TargetScan
miR-4468	ENSG00000204954	7mer-m8	TargetScan
miR-4468	ENSG00000204977	7mer-m8	TargetScan
miR-4468	ENSG00000205212	7mer-m8	TargetScan
miR-4468	ENSG00000205572	7mer-m8	TargetScan
miR-4468	ENSG00000205593	7mer-m8	TargetScan
miR-4468	ENSG00000205791	7mer-m8	TargetScan
miR-4468	ENSG00000205808	7mer-m8	TargetScan
miR-4468	ENSG00000206047	7mer-m8	TargetScan
miR-4468	ENSG00000206053	7mer-m8	TargetScan
miR-4468	ENSG00000212659	7mer-m8	TargetScan
miR-4468	ENSG00000212722	7mer-m8	TargetScan
miR-4468	ENSG00000213064	7mer-m8	TargetScan
miR-4468	ENSG00000213672	7mer-m8	TargetScan
miR-4468	ENSG00000213886	7mer-m8	TargetScan

miR-4468	ENSG00000213967	7mer-m8	TargetScan
miR-4468	ENSG00000213973	7mer-m8	TargetScan
miR-4468	ENSG00000213988	7mer-m8	TargetScan
miR-4468	ENSG00000214050	7mer-m8	TargetScan
miR-4468	ENSG00000214357	7mer-m8	TargetScan
miR-4468	ENSG00000214530	7mer-m8	TargetScan
miR-4468	ENSG00000215114	7mer-m8	TargetScan
miR-4468	ENSG00000220891	7mer-m8	TargetScan
miR-4468	ENSG00000221823	7mer-m8	TargetScan
miR-4468	ENSG00000221845	7mer-m8	TargetScan
miR-4468	ENSG00000221963	7mer-m8	TargetScan
miR-4468	ENSG00000222001	7mer-m8	TargetScan
miR-4468	ENSG00000223572	7mer-m8	TargetScan
miR-4468	ENSG00000223865	7mer-m8	TargetScan
miR-4468	ENSG00000225697	7mer-m8	TargetScan
miR-4468	ENSG00000225830	7mer-m8	TargetScan
miR-4468	ENSG00000227051	7mer-m8	TargetScan
miR-4468	ENSG00000228075	7mer-m8	TargetScan
miR-4468	ENSG00000232040	7mer-m8	TargetScan
miR-4468	ENSG00000232119	7mer-m8	TargetScan
miR-4468	ENSG00000233024	7mer-m8	TargetScan
miR-4468	ENSG00000235750	7mer-m8	TargetScan
miR-4468	ENSG00000236279	7mer-m8	TargetScan
miR-4468	ENSG00000237289	7mer-m8	TargetScan
miR-4468	ENSG00000237440	7mer-m8	TargetScan
miR-4468	ENSG00000239306	7mer-m8	TargetScan
miR-4468	ENSG00000239839	7mer-m8	TargetScan
miR-4468	ENSG00000239886	7mer-m8	TargetScan
miR-4468	ENSG00000240053	7mer-m8	TargetScan
miR-4468	ENSG00000240247	7mer-m8	TargetScan
miR-4468	ENSG00000240694	7mer-m8	TargetScan

miR-4468	ENSG00000241595	7mer-m8	TargetScan
miR-4468	ENSG00000245680	7mer-m8	TargetScan
miR-4468	ENSG00000247746	7mer-m8	TargetScan
miR-4468	ENSG00000249884	7mer-m8	TargetScan
miR-4468	ENSG00000250091	7mer-m8	TargetScan
miR-4468	ENSG00000254087	7mer-m8	TargetScan
miR-4468	ENSG00000255112	7mer-m8	TargetScan
miR-4468	ENSG00000257704	7mer-m8	TargetScan
miR-4468	ENSG00000257923	7mer-m8	TargetScan
miR-4468	ENSG00000259207	7mer-m8	TargetScan
miR-4468	ENSG00000260007	7mer-m8	TargetScan
miR-4468	ENSG00000260548	7mer-m8	TargetScan
miR-4468	ENSG00000263020	7mer-m8	TargetScan
miR-4468	ENSG00000267432	7mer-m8	TargetScan
miR-4468	ENSG00000269343	7mer-m8	TargetScan
miR-4468	ENSG00000272195	7mer-m8	TargetScan
miR-4468	ENSG00000273018	7mer-m8	TargetScan
miR-4468	ENSG00000273802	7mer-m8	TargetScan
miR-4468	ENSG00000284448	7mer-m8	TargetScan
miR-4468	ENSG00000285043	7mer-m8	TargetScan
miR-4468	ENSG00000285347	7mer-m8	TargetScan
miR-4468	ENSG00000286053	7mer-m8	TargetScan
miR-4468	ENSG00000288436	7mer-m8	TargetScan
miR-4788	ENSG00000099308	7mer-m8	TargetScan
miR-4788	ENSG00000099940	7mer-m8	TargetScan
miR-4788	ENSG00000100350	7mer-m8	TargetScan
miR-4788	ENSG00000100416	7mer-m8	TargetScan
miR-4788	ENSG00000104524	7mer-m8	TargetScan
miR-4788	ENSG00000107130	7mer-m8	TargetScan
miR-4788	ENSG00000107282	7mer-m8	TargetScan
miR-4788	ENSG00000108176	7mer-m8	TargetScan

miR-4788	ENSG00000108924	7mer-m8	TargetScan
miR-4788	ENSG00000109171	7mer-m8	TargetScan
miR-4788	ENSG00000112742	7mer-m8	TargetScan
miR-4788	ENSG00000115020	7mer-m8	TargetScan
miR-4788	ENSG00000116120	7mer-m8	TargetScan
miR-4788	ENSG00000118705	7mer-m8	TargetScan
miR-4788	ENSG00000127329	7mer-m8	TargetScan
miR-4788	ENSG00000132294	7mer-m8	TargetScan
miR-4788	ENSG00000134202	7mer-m8	TargetScan
miR-4788	ENSG00000135272	7mer-m8	TargetScan
miR-4788	ENSG00000137185	7mer-m8	TargetScan
miR-4788	ENSG00000140950	7mer-m8	TargetScan
miR-4788	ENSG00000143158	7mer-m8	TargetScan
miR-4788	ENSG00000143322	7mer-m8	TargetScan
miR-4788	ENSG00000144619	7mer-m8	TargetScan
miR-4788	ENSG00000144840	7mer-m8	TargetScan
miR-4788	ENSG00000145833	7mer-m8	TargetScan
miR-4788	ENSG00000146802	7mer-m8	TargetScan
miR-4788	ENSG00000151151	7mer-m8	TargetScan
miR-4788	ENSG00000152684	7mer-m8	TargetScan
miR-4788	ENSG00000164237	7mer-m8	TargetScan
miR-4788	ENSG00000164808	7mer-m8	TargetScan
miR-4788	ENSG00000167487	7mer-m8	TargetScan
miR-4788	ENSG00000169567	7mer-m8	TargetScan
miR-4788	ENSG00000171848	7mer-m8	TargetScan
miR-4788	ENSG00000180354	7mer-m8	TargetScan
miR-4788	ENSG00000183166	7mer-m8	TargetScan
miR-4788	ENSG00000184378	7mer-m8	TargetScan
miR-4788	ENSG00000189051	7mer-m8	TargetScan
miR-4788	ENSG00000196616	7mer-m8	TargetScan
miR-4788	ENSG00000198492	7mer-m8	TargetScan

miR-4788	ENSG00000215014	7mer-m8	TargetScan
miR-4788	ENSG00000224877	7mer-m8	TargetScan
miR-4788	ENSG00000251322	7mer-m8	TargetScan
miR-492	ENSG00000003402	7mer-m8	TargetScan
miR-492	ENSG00000005156	7mer-m8	TargetScan
miR-492	ENSG00000006432	7mer-m8	TargetScan
miR-492	ENSG00000006453	7mer-m8	TargetScan
miR-492	ENSG00000006740	7mer-m8	TargetScan
miR-492	ENSG00000007541	7mer-m8	TargetScan
miR-492	ENSG00000007923	7mer-m8	TargetScan
miR-492	ENSG00000011523	7mer-m8	TargetScan
miR-492	ENSG00000011677	7mer-m8	TargetScan
miR-492	ENSG00000012223	7mer-m8	TargetScan
miR-492	ENSG00000014919	7mer-m8	TargetScan
miR-492	ENSG00000015676	7mer-m8	TargetScan
miR-492	ENSG00000019995	7mer-m8	TargetScan
miR-492	ENSG00000020129	7mer-m8	TargetScan
miR-492	ENSG00000021645	7mer-m8	TargetScan
miR-492	ENSG00000026508	7mer-m8	TargetScan
miR-492	ENSG00000026652	7mer-m8	TargetScan
miR-492	ENSG00000033867	7mer-m8	TargetScan
miR-492	ENSG00000034677	7mer-m8	TargetScan
miR-492	ENSG00000035403	7mer-m8	TargetScan
miR-492	ENSG00000036448	7mer-m8	TargetScan
miR-492	ENSG00000048544	7mer-m8	TargetScan
miR-492	ENSG00000050555	7mer-m8	TargetScan
miR-492	ENSG00000050748	7mer-m8	TargetScan
miR-492	ENSG00000051620	7mer-m8	TargetScan
miR-492	ENSG00000054965	7mer-m8	TargetScan
miR-492	ENSG00000059122	7mer-m8	TargetScan
miR-492	ENSG00000063245	7mer-m8	TargetScan

miR-492	ENSG00000064199	7mer-m8	TargetScan
miR-492	ENSG00000064393	7mer-m8	TargetScan
miR-492	ENSG00000070193	7mer-m8	TargetScan
miR-492	ENSG00000070526	7mer-m8	TargetScan
miR-492	ENSG00000070614	7mer-m8	TargetScan
miR-492	ENSG00000070886	7mer-m8	TargetScan
miR-492	ENSG00000070915	7mer-m8	TargetScan
miR-492	ENSG00000072133	7mer-m8	TargetScan
miR-492	ENSG00000073670	7mer-m8	TargetScan
miR-492	ENSG00000073849	7mer-m8	TargetScan
miR-492	ENSG00000075914	7mer-m8	TargetScan
miR-492	ENSG00000076356	7mer-m8	TargetScan
miR-492	ENSG00000076641	7mer-m8	TargetScan
miR-492	ENSG00000078814	7mer-m8	TargetScan
miR-492	ENSG00000079308	7mer-m8	TargetScan
miR-492	ENSG00000081138	7mer-m8	TargetScan
miR-492	ENSG00000081386	7mer-m8	TargetScan
miR-492	ENSG00000082684	7mer-m8	TargetScan
miR-492	ENSG00000083223	7mer-m8	TargetScan
miR-492	ENSG00000083444	7mer-m8	TargetScan
miR-492	ENSG00000084090	7mer-m8	TargetScan
miR-492	ENSG00000084628	7mer-m8	TargetScan
miR-492	ENSG00000085117	7mer-m8	TargetScan
miR-492	ENSG00000085721	7mer-m8	TargetScan
miR-492	ENSG00000085831	7mer-m8	TargetScan
miR-492	ENSG00000086062	7mer-m8	TargetScan
miR-492	ENSG00000087191	7mer-m8	TargetScan
miR-492	ENSG00000088812	7mer-m8	TargetScan
miR-492	ENSG00000090889	7mer-m8	TargetScan
miR-492	ENSG00000092445	7mer-m8	TargetScan
miR-492	ENSG00000095539	7mer-m8	TargetScan

miR-492	ENSG00000099385	7mer-m8	TargetScan
miR-492	ENSG00000099625	7mer-m8	TargetScan
miR-492	ENSG00000099917	7mer-m8	TargetScan
miR-492	ENSG00000100241	7mer-m8	TargetScan
miR-492	ENSG00000100296	7mer-m8	TargetScan
miR-492	ENSG00000100341	7mer-m8	TargetScan
miR-492	ENSG00000100359	7mer-m8	TargetScan
miR-492	ENSG00000100441	7mer-m8	TargetScan
miR-492	ENSG00000101213	7mer-m8	TargetScan
miR-492	ENSG00000101255	7mer-m8	TargetScan
miR-492	ENSG00000101290	7mer-m8	TargetScan
miR-492	ENSG00000101444	7mer-m8	TargetScan
miR-492	ENSG00000101445	7mer-m8	TargetScan
miR-492	ENSG00000101849	7mer-m8	TargetScan
miR-492	ENSG00000101892	7mer-m8	TargetScan
miR-492	ENSG00000102362	7mer-m8	TargetScan
miR-492	ENSG00000102385	7mer-m8	TargetScan
miR-492	ENSG00000102763	7mer-m8	TargetScan
miR-492	ENSG00000102858	7mer-m8	TargetScan
miR-492	ENSG00000102967	7mer-m8	TargetScan
miR-492	ENSG00000103064	7mer-m8	TargetScan
miR-492	ENSG00000103067	7mer-m8	TargetScan
miR-492	ENSG00000103196	7mer-m8	TargetScan
miR-492	ENSG00000103275	7mer-m8	TargetScan
miR-492	ENSG00000103363	7mer-m8	TargetScan
miR-492	ENSG00000103365	7mer-m8	TargetScan
miR-492	ENSG00000103550	7mer-m8	TargetScan
miR-492	ENSG00000103888	7mer-m8	TargetScan
miR-492	ENSG00000104332	7mer-m8	TargetScan
miR-492	ENSG00000104805	7mer-m8	TargetScan
miR-492	ENSG00000104884	7mer-m8	TargetScan

miR-492	ENSG00000105063	7mer-m8	TargetScan
miR-492	ENSG00000105429	7mer-m8	TargetScan
miR-492	ENSG00000105707	7mer-m8	TargetScan
miR-492	ENSG00000106236	7mer-m8	TargetScan
miR-492	ENSG00000106246	7mer-m8	TargetScan
miR-492	ENSG00000106477	7mer-m8	TargetScan
miR-492	ENSG00000106665	7mer-m8	TargetScan
miR-492	ENSG00000107185	7mer-m8	TargetScan
miR-492	ENSG00000107282	7mer-m8	TargetScan
miR-492	ENSG00000107338	7mer-m8	TargetScan
miR-492	ENSG00000108306	7mer-m8	TargetScan
miR-492	ENSG00000108375	7mer-m8	TargetScan
miR-492	ENSG00000108684	7mer-m8	TargetScan
miR-492	ENSG00000108823	7mer-m8	TargetScan
miR-492	ENSG00000108852	7mer-m8	TargetScan
miR-492	ENSG00000109189	7mer-m8	TargetScan
miR-492	ENSG00000109572	7mer-m8	TargetScan
miR-492	ENSG00000109906	7mer-m8	TargetScan
miR-492	ENSG00000110063	7mer-m8	TargetScan
miR-492	ENSG00000110245	7mer-m8	TargetScan
miR-492	ENSG00000110429	7mer-m8	TargetScan
miR-492	ENSG00000110665	7mer-m8	TargetScan
miR-492	ENSG00000110711	7mer-m8	TargetScan
miR-492	ENSG00000110876	7mer-m8	TargetScan
miR-492	ENSG00000110906	7mer-m8	TargetScan
miR-492	ENSG00000111262	7mer-m8	TargetScan
miR-492	ENSG00000111331	7mer-m8	TargetScan
miR-492	ENSG00000111671	7mer-m8	TargetScan
miR-492	ENSG00000111886	7mer-m8	TargetScan
miR-492	ENSG00000112149	7mer-m8	TargetScan
miR-492	ENSG00000112182	7mer-m8	TargetScan

miR-492	ENSG00000112208	7mer-m8	TargetScan
miR-492	ENSG00000112902	7mer-m8	TargetScan
miR-492	ENSG00000113269	7mer-m8	TargetScan
miR-492	ENSG00000113300	7mer-m8	TargetScan
miR-492	ENSG00000113504	7mer-m8	TargetScan
miR-492	ENSG00000113600	7mer-m8	TargetScan
miR-492	ENSG00000113971	7mer-m8	TargetScan
miR-492	ENSG00000114126	7mer-m8	TargetScan
miR-492	ENSG00000114745	7mer-m8	TargetScan
miR-492	ENSG00000114904	7mer-m8	TargetScan
miR-492	ENSG00000115194	7mer-m8	TargetScan
miR-492	ENSG00000115325	7mer-m8	TargetScan
miR-492	ENSG00000115368	7mer-m8	TargetScan
miR-492	ENSG00000115461	7mer-m8	TargetScan
miR-492	ENSG00000115808	7mer-m8	TargetScan
miR-492	ENSG00000115884	7mer-m8	TargetScan
miR-492	ENSG00000116147	7mer-m8	TargetScan
miR-492	ENSG00000116260	7mer-m8	TargetScan
miR-492	ENSG00000116667	7mer-m8	TargetScan
miR-492	ENSG00000117298	7mer-m8	TargetScan
miR-492	ENSG00000117480	7mer-m8	TargetScan
miR-492	ENSG00000118271	7mer-m8	TargetScan
miR-492	ENSG00000119314	7mer-m8	TargetScan
miR-492	ENSG00000119411	7mer-m8	TargetScan
miR-492	ENSG00000119547	7mer-m8	TargetScan
miR-492	ENSG00000120549	7mer-m8	TargetScan
miR-492	ENSG00000120833	7mer-m8	TargetScan
miR-492	ENSG00000120907	7mer-m8	TargetScan
miR-492	ENSG00000121406	7mer-m8	TargetScan
miR-492	ENSG00000122678	7mer-m8	TargetScan
miR-492	ENSG00000122783	7mer-m8	TargetScan

miR-492	ENSG00000123353	7mer-m8	TargetScan
miR-492	ENSG00000123684	7mer-m8	TargetScan
miR-492	ENSG00000123908	7mer-m8	TargetScan
miR-492	ENSG00000123933	7mer-m8	TargetScan
miR-492	ENSG00000124164	7mer-m8	TargetScan
miR-492	ENSG00000124249	7mer-m8	TargetScan
miR-492	ENSG00000124251	7mer-m8	TargetScan
miR-492	ENSG00000125484	7mer-m8	TargetScan
miR-492	ENSG00000125869	7mer-m8	TargetScan
miR-492	ENSG00000125952	7mer-m8	TargetScan
miR-492	ENSG00000125965	7mer-m8	TargetScan
miR-492	ENSG00000126003	7mer-m8	TargetScan
miR-492	ENSG00000126217	7mer-m8	TargetScan
miR-492	ENSG00000126218	7mer-m8	TargetScan
miR-492	ENSG00000126785	7mer-m8	TargetScan
miR-492	ENSG00000127334	7mer-m8	TargetScan
miR-492	ENSG00000127564	7mer-m8	TargetScan
miR-492	ENSG00000127946	7mer-m8	TargetScan
miR-492	ENSG00000128203	7mer-m8	TargetScan
miR-492	ENSG00000128284	7mer-m8	TargetScan
miR-492	ENSG00000128487	7mer-m8	TargetScan
miR-492	ENSG00000128609	7mer-m8	TargetScan
miR-492	ENSG00000128713	7mer-m8	TargetScan
miR-492	ENSG00000128872	7mer-m8	TargetScan
miR-492	ENSG00000128908	7mer-m8	TargetScan
miR-492	ENSG00000129007	7mer-m8	TargetScan
miR-492	ENSG00000129295	7mer-m8	TargetScan
miR-492	ENSG00000129353	7mer-m8	TargetScan
miR-492	ENSG00000129654	7mer-m8	TargetScan
miR-492	ENSG00000130309	7mer-m8	TargetScan
miR-492	ENSG00000130592	7mer-m8	TargetScan

miR-492	ENSG00000130695	7mer-m8	TargetScan
miR-492	ENSG00000130720	7mer-m8	TargetScan
miR-492	ENSG00000130723	7mer-m8	TargetScan
miR-492	ENSG00000130749	7mer-m8	TargetScan
miR-492	ENSG00000130856	7mer-m8	TargetScan
miR-492	ENSG00000130956	7mer-m8	TargetScan
miR-492	ENSG00000131044	7mer-m8	TargetScan
miR-492	ENSG00000131408	7mer-m8	TargetScan
miR-492	ENSG00000132693	7mer-m8	TargetScan
miR-492	ENSG00000132953	7mer-m8	TargetScan
miR-492	ENSG00000133030	7mer-m8	TargetScan
miR-492	ENSG00000133055	7mer-m8	TargetScan
miR-492	ENSG00000134248	7mer-m8	TargetScan
miR-492	ENSG00000134531	7mer-m8	TargetScan
miR-492	ENSG00000135297	7mer-m8	TargetScan
miR-492	ENSG00000135299	7mer-m8	TargetScan
miR-492	ENSG00000135372	7mer-m8	TargetScan
miR-492	ENSG00000135374	7mer-m8	TargetScan
miR-492	ENSG00000135414	7mer-m8	TargetScan
miR-492	ENSG00000135525	7mer-m8	TargetScan
miR-492	ENSG00000135898	7mer-m8	TargetScan
miR-492	ENSG00000136026	7mer-m8	TargetScan
miR-492	ENSG00000136155	7mer-m8	TargetScan
miR-492	ENSG00000136286	7mer-m8	TargetScan
miR-492	ENSG00000136527	7mer-m8	TargetScan
miR-492	ENSG00000136718	7mer-m8	TargetScan
miR-492	ENSG00000136720	7mer-m8	TargetScan
miR-492	ENSG00000136877	7mer-m8	TargetScan
miR-492	ENSG00000137077	7mer-m8	TargetScan
miR-492	ENSG00000137094	7mer-m8	TargetScan
miR-492	ENSG00000137106	7mer-m8	TargetScan

miR-492	ENSG00000137135	7mer-m8	TargetScan
miR-492	ENSG00000137171	7mer-m8	TargetScan
miR-492	ENSG00000137834	7mer-m8	TargetScan
miR-492	ENSG00000137959	7mer-m8	TargetScan
miR-492	ENSG00000138380	7mer-m8	TargetScan
miR-492	ENSG00000138443	7mer-m8	TargetScan
miR-492	ENSG00000139990	7mer-m8	TargetScan
miR-492	ENSG00000140015	7mer-m8	TargetScan
miR-492	ENSG00000140368	7mer-m8	TargetScan
miR-492	ENSG00000140650	7mer-m8	TargetScan
miR-492	ENSG00000140876	7mer-m8	TargetScan
miR-492	ENSG00000140987	7mer-m8	TargetScan
miR-492	ENSG00000141252	7mer-m8	TargetScan
miR-492	ENSG00000141404	7mer-m8	TargetScan
miR-492	ENSG00000141446	7mer-m8	TargetScan
miR-492	ENSG00000141519	7mer-m8	TargetScan
miR-492	ENSG00000141526	7mer-m8	TargetScan
miR-492	ENSG00000141580	7mer-m8	TargetScan
miR-492	ENSG00000142661	7mer-m8	TargetScan
miR-492	ENSG00000142677	7mer-m8	TargetScan
miR-492	ENSG00000143374	7mer-m8	TargetScan
miR-492	ENSG00000143376	7mer-m8	TargetScan
miR-492	ENSG00000143603	7mer-m8	TargetScan
miR-492	ENSG00000143740	7mer-m8	TargetScan
miR-492	ENSG00000143847	7mer-m8	TargetScan
miR-492	ENSG00000144043	7mer-m8	TargetScan
miR-492	ENSG00000144152	7mer-m8	TargetScan
miR-492	ENSG00000144228	7mer-m8	TargetScan
miR-492	ENSG00000144355	7mer-m8	TargetScan
miR-492	ENSG00000144357	7mer-m8	TargetScan
miR-492	ENSG00000144847	7mer-m8	TargetScan

miR-492	ENSG00000145779	7mer-m8	TargetScan
miR-492	ENSG00000145832	7mer-m8	TargetScan
miR-492	ENSG00000146216	7mer-m8	TargetScan
miR-492	ENSG00000146263	7mer-m8	TargetScan
miR-492	ENSG00000146267	7mer-m8	TargetScan
miR-492	ENSG00000146676	7mer-m8	TargetScan
miR-492	ENSG00000147010	7mer-m8	TargetScan
miR-492	ENSG00000147145	7mer-m8	TargetScan
miR-492	ENSG00000147234	7mer-m8	TargetScan
miR-492	ENSG00000147614	7mer-m8	TargetScan
miR-492	ENSG00000148053	7mer-m8	TargetScan
miR-492	ENSG00000148120	7mer-m8	TargetScan
miR-492	ENSG00000148153	7mer-m8	TargetScan
miR-492	ENSG00000148296	7mer-m8	TargetScan
miR-492	ENSG00000148384	7mer-m8	TargetScan
miR-492	ENSG00000148704	7mer-m8	TargetScan
miR-492	ENSG00000148730	7mer-m8	TargetScan
miR-492	ENSG00000148848	7mer-m8	TargetScan
miR-492	ENSG00000149212	7mer-m8	TargetScan
miR-492	ENSG00000149451	7mer-m8	TargetScan
miR-492	ENSG00000149488	7mer-m8	TargetScan
miR-492	ENSG00000149575	7mer-m8	TargetScan
miR-492	ENSG00000151136	7mer-m8	TargetScan
miR-492	ENSG00000151164	7mer-m8	TargetScan
miR-492	ENSG00000151532	7mer-m8	TargetScan
miR-492	ENSG00000151948	7mer-m8	TargetScan
miR-492	ENSG00000153037	7mer-m8	TargetScan
miR-492	ENSG00000153404	7mer-m8	TargetScan
miR-492	ENSG00000153879	7mer-m8	TargetScan
miR-492	ENSG00000153904	7mer-m8	TargetScan
miR-492	ENSG00000153936	7mer-m8	TargetScan

miR-492	ENSG00000154025	7mer-m8	TargetScan
miR-492	ENSG00000154174	7mer-m8	TargetScan
miR-492	ENSG00000154473	7mer-m8	TargetScan
miR-492	ENSG00000154518	7mer-m8	TargetScan
miR-492	ENSG00000154734	7mer-m8	TargetScan
miR-492	ENSG00000154930	7mer-m8	TargetScan
miR-492	ENSG00000155254	7mer-m8	TargetScan
miR-492	ENSG00000155561	7mer-m8	TargetScan
miR-492	ENSG00000155657	7mer-m8	TargetScan
miR-492	ENSG00000155926	7mer-m8	TargetScan
miR-492	ENSG00000155966	7mer-m8	TargetScan
miR-492	ENSG00000156172	7mer-m8	TargetScan
miR-492	ENSG00000156232	7mer-m8	TargetScan
miR-492	ENSG00000156239	7mer-m8	TargetScan
miR-492	ENSG00000157388	7mer-m8	TargetScan
miR-492	ENSG00000157540	7mer-m8	TargetScan
miR-492	ENSG00000157617	7mer-m8	TargetScan
miR-492	ENSG00000157657	7mer-m8	TargetScan
miR-492	ENSG00000157680	7mer-m8	TargetScan
miR-492	ENSG00000157741	7mer-m8	TargetScan
miR-492	ENSG00000158163	7mer-m8	TargetScan
miR-492	ENSG00000158423	7mer-m8	TargetScan
miR-492	ENSG00000158445	7mer-m8	TargetScan
miR-492	ENSG00000158813	7mer-m8	TargetScan
miR-492	ENSG00000158863	7mer-m8	TargetScan
miR-492	ENSG00000159111	7mer-m8	TargetScan
miR-492	ENSG00000159348	7mer-m8	TargetScan
miR-492	ENSG00000160194	7mer-m8	TargetScan
miR-492	ENSG00000160199	7mer-m8	TargetScan
miR-492	ENSG00000160216	7mer-m8	TargetScan
miR-492	ENSG00000160233	7mer-m8	TargetScan

miR-492	ENSG00000160716	7mer-m8	TargetScan
miR-492	ENSG00000161203	7mer-m8	TargetScan
miR-492	ENSG00000162066	7mer-m8	TargetScan
miR-492	ENSG00000162341	7mer-m8	TargetScan
miR-492	ENSG00000162461	7mer-m8	TargetScan
miR-492	ENSG00000162545	7mer-m8	TargetScan
miR-492	ENSG00000162676	7mer-m8	TargetScan
miR-492	ENSG00000162783	7mer-m8	TargetScan
miR-492	ENSG00000162971	7mer-m8	TargetScan
miR-492	ENSG00000163393	7mer-m8	TargetScan
miR-492	ENSG00000163820	7mer-m8	TargetScan
miR-492	ENSG00000164007	7mer-m8	TargetScan
miR-492	ENSG00000164080	7mer-m8	TargetScan
miR-492	ENSG00000164366	7mer-m8	TargetScan
miR-492	ENSG00000164626	7mer-m8	TargetScan
miR-492	ENSG00000164627	7mer-m8	TargetScan
miR-492	ENSG00000164674	7mer-m8	TargetScan
miR-492	ENSG00000164715	7mer-m8	TargetScan
miR-492	ENSG00000164978	7mer-m8	TargetScan
miR-492	ENSG00000165194	7mer-m8	TargetScan
miR-492	ENSG00000165609	7mer-m8	TargetScan
miR-492	ENSG00000165650	7mer-m8	TargetScan
miR-492	ENSG00000165861	7mer-m8	TargetScan
miR-492	ENSG00000166170	7mer-m8	TargetScan
miR-492	ENSG00000166257	7mer-m8	TargetScan
miR-492	ENSG00000166341	7mer-m8	TargetScan
miR-492	ENSG00000166387	7mer-m8	TargetScan
miR-492	ENSG00000166436	7mer-m8	TargetScan
miR-492	ENSG00000166507	7mer-m8	TargetScan
miR-492	ENSG00000166750	7mer-m8	TargetScan
miR-492	ENSG00000166833	7mer-m8	TargetScan

miR-492	ENSG00000166923	7mer-m8	TargetScan
miR-492	ENSG00000166984	7mer-m8	TargetScan
miR-492	ENSG00000167131	7mer-m8	TargetScan
miR-492	ENSG00000167186	7mer-m8	TargetScan
miR-492	ENSG00000167208	7mer-m8	TargetScan
miR-492	ENSG00000167291	7mer-m8	TargetScan
miR-492	ENSG00000167461	7mer-m8	TargetScan
miR-492	ENSG00000167613	7mer-m8	TargetScan
miR-492	ENSG00000167656	7mer-m8	TargetScan
miR-492	ENSG00000167703	7mer-m8	TargetScan
miR-492	ENSG00000167910	7mer-m8	TargetScan
miR-492	ENSG00000168062	7mer-m8	TargetScan
miR-492	ENSG00000168159	7mer-m8	TargetScan
miR-492	ENSG00000168569	7mer-m8	TargetScan
miR-492	ENSG00000168724	7mer-m8	TargetScan
miR-492	ENSG00000168903	7mer-m8	TargetScan
miR-492	ENSG00000168904	7mer-m8	TargetScan
miR-492	ENSG00000168906	7mer-m8	TargetScan
miR-492	ENSG00000168939	7mer-m8	TargetScan
miR-492	ENSG00000168939	7mer-m8	TargetScan
miR-492	ENSG00000169021	7mer-m8	TargetScan
miR-492	ENSG00000169083	7mer-m8	TargetScan
miR-492	ENSG00000169118	7mer-m8	TargetScan
miR-492	ENSG00000169379	7mer-m8	TargetScan
miR-492	ENSG00000169604	7mer-m8	TargetScan
miR-492	ENSG00000169733	7mer-m8	TargetScan
miR-492	ENSG00000169855	7mer-m8	TargetScan
miR-492	ENSG00000169908	7mer-m8	TargetScan
miR-492	ENSG00000169933	7mer-m8	TargetScan
miR-492	ENSG00000170160	7mer-m8	TargetScan
miR-492	ENSG00000170525	7mer-m8	TargetScan

miR-492	ENSG00000170571	7mer-m8	TargetScan
miR-492	ENSG00000170962	7mer-m8	TargetScan
miR-492	ENSG00000171124	7mer-m8	TargetScan
miR-492	ENSG00000171246	7mer-m8	TargetScan
miR-492	ENSG00000171792	7mer-m8	TargetScan
miR-492	ENSG00000171914	7mer-m8	TargetScan
miR-492	ENSG00000172057	7mer-m8	TargetScan
miR-492	ENSG00000172113	7mer-m8	TargetScan
miR-492	ENSG00000172292	7mer-m8	TargetScan
miR-492	ENSG00000172538	7mer-m8	TargetScan
miR-492	ENSG00000172819	7mer-m8	TargetScan
miR-492	ENSG00000172893	7mer-m8	TargetScan
miR-492	ENSG00000172987	7mer-m8	TargetScan
miR-492	ENSG00000173218	7mer-m8	TargetScan
miR-492	ENSG00000173432	7mer-m8	TargetScan
miR-492	ENSG00000173535	7mer-m8	TargetScan
miR-492	ENSG00000173548	7mer-m8	TargetScan
miR-492	ENSG00000173578	7mer-m8	TargetScan
miR-492	ENSG00000173744	7mer-m8	TargetScan
miR-492	ENSG00000173801	7mer-m8	TargetScan
miR-492	ENSG00000173821	7mer-m8	TargetScan
miR-492	ENSG00000173838	7mer-m8	TargetScan
miR-492	ENSG00000173894	7mer-m8	TargetScan
miR-492	ENSG00000173933	7mer-m8	TargetScan
miR-492	ENSG00000173950	7mer-m8	TargetScan
miR-492	ENSG00000173992	7mer-m8	TargetScan
miR-492	ENSG00000174145	7mer-m8	TargetScan
miR-492	ENSG00000174227	7mer-m8	TargetScan
miR-492	ENSG00000174358	7mer-m8	TargetScan
miR-492	ENSG00000174373	7mer-m8	TargetScan
miR-492	ENSG00000174574	7mer-m8	TargetScan

miR-492	ENSG00000174600	7mer-m8	TargetScan
miR-492	ENSG00000175221	7mer-m8	TargetScan
miR-492	ENSG00000175728	7mer-m8	TargetScan
miR-492	ENSG00000176171	7mer-m8	TargetScan
miR-492	ENSG00000176383	7mer-m8	TargetScan
miR-492	ENSG00000176834	7mer-m8	TargetScan
miR-492	ENSG00000176896	7mer-m8	TargetScan
miR-492	ENSG00000176920	7mer-m8	TargetScan
miR-492	ENSG00000176945	7mer-m8	TargetScan
miR-492	ENSG00000176974	7mer-m8	TargetScan
miR-492	ENSG00000177058	7mer-m8	TargetScan
miR-492	ENSG00000177352	7mer-m8	TargetScan
miR-492	ENSG00000177463	7mer-m8	TargetScan
miR-492	ENSG00000177663	7mer-m8	TargetScan
miR-492	ENSG00000177674	7mer-m8	TargetScan
miR-492	ENSG00000177732	7mer-m8	TargetScan
miR-492	ENSG00000178057	7mer-m8	TargetScan
miR-492	ENSG00000178171	7mer-m8	TargetScan
miR-492	ENSG00000178233	7mer-m8	TargetScan
miR-492	ENSG00000178287	7mer-m8	TargetScan
miR-492	ENSG00000178860	7mer-m8	TargetScan
miR-492	ENSG00000179348	7mer-m8	TargetScan
miR-492	ENSG00000179387	7mer-m8	TargetScan
miR-492	ENSG00000179583	7mer-m8	TargetScan
miR-492	ENSG00000179813	7mer-m8	TargetScan
miR-492	ENSG00000179886	7mer-m8	TargetScan
miR-492	ENSG00000180438	7mer-m8	TargetScan
miR-492	ENSG00000180535	7mer-m8	TargetScan
miR-492	ENSG00000180869	7mer-m8	TargetScan
miR-492	ENSG00000180901	7mer-m8	TargetScan
miR-492	ENSG00000181027	7mer-m8	TargetScan

miR-492	ENSG00000181090	7mer-m8	TargetScan
miR-492	ENSG00000181610	7mer-m8	TargetScan
miR-492	ENSG00000181982	7mer-m8	TargetScan
miR-492	ENSG00000182095	7mer-m8	TargetScan
miR-492	ENSG00000182156	7mer-m8	TargetScan
miR-492	ENSG00000182185	7mer-m8	TargetScan
miR-492	ENSG00000182704	7mer-m8	TargetScan
miR-492	ENSG00000182742	7mer-m8	TargetScan
miR-492	ENSG00000182858	7mer-m8	TargetScan
miR-492	ENSG00000182957	7mer-m8	TargetScan
miR-492	ENSG00000182968	7mer-m8	TargetScan
miR-492	ENSG00000183287	7mer-m8	TargetScan
miR-492	ENSG00000183527	7mer-m8	TargetScan
miR-492	ENSG00000183723	7mer-m8	TargetScan
miR-492	ENSG00000183751	7mer-m8	TargetScan
miR-492	ENSG00000184076	7mer-m8	TargetScan
miR-492	ENSG00000184144	7mer-m8	TargetScan
miR-492	ENSG00000184185	7mer-m8	TargetScan
miR-492	ENSG00000184560	7mer-m8	TargetScan
miR-492	ENSG00000184937	7mer-m8	TargetScan
miR-492	ENSG00000185055	7mer-m8	TargetScan
miR-492	ENSG00000185088	7mer-m8	TargetScan
miR-492	ENSG00000185112	7mer-m8	TargetScan
miR-492	ENSG00000185262	7mer-m8	TargetScan
miR-492	ENSG00000185721	7mer-m8	TargetScan
miR-492	ENSG00000185722	7mer-m8	TargetScan
miR-492	ENSG00000185736	7mer-m8	TargetScan
miR-492	ENSG00000185739	7mer-m8	TargetScan
miR-492	ENSG00000185745	7mer-m8	TargetScan
miR-492	ENSG00000185860	7mer-m8	TargetScan
miR-492	ENSG00000185924	7mer-m8	TargetScan

miR-492	ENSG00000185933	7mer-m8	TargetScan
miR-492	ENSG00000186051	7mer-m8	TargetScan
miR-492	ENSG00000186174	7mer-m8	TargetScan
miR-492	ENSG00000186260	7mer-m8	TargetScan
miR-492	ENSG00000186431	7mer-m8	TargetScan
miR-492	ENSG00000186812	7mer-m8	TargetScan
miR-492	ENSG00000187123	7mer-m8	TargetScan
miR-492	ENSG00000187187	7mer-m8	TargetScan
miR-492	ENSG00000187239	7mer-m8	TargetScan
miR-492	ENSG00000187391	7mer-m8	TargetScan
miR-492	ENSG00000187726	7mer-m8	TargetScan
miR-492	ENSG00000187840	7mer-m8	TargetScan
miR-492	ENSG00000187942	7mer-m8	TargetScan
miR-492	ENSG00000187980	7mer-m8	TargetScan
miR-492	ENSG00000188167	7mer-m8	TargetScan
miR-492	ENSG00000188373	7mer-m8	TargetScan
miR-492	ENSG00000188488	7mer-m8	TargetScan
miR-492	ENSG00000188522	7mer-m8	TargetScan
miR-492	ENSG00000188529	7mer-m8	TargetScan
miR-492	ENSG00000188735	7mer-m8	TargetScan
miR-492	ENSG00000188827	7mer-m8	TargetScan
miR-492	ENSG00000189007	7mer-m8	TargetScan
miR-492	ENSG00000189023	7mer-m8	TargetScan
miR-492	ENSG00000189091	7mer-m8	TargetScan
miR-492	ENSG00000189319	7mer-m8	TargetScan
miR-492	ENSG00000189320	7mer-m8	TargetScan
miR-492	ENSG00000196155	7mer-m8	TargetScan
miR-492	ENSG00000196167	7mer-m8	TargetScan
miR-492	ENSG00000196187	7mer-m8	TargetScan
miR-492	ENSG00000196209	7mer-m8	TargetScan
miR-492	ENSG00000196220	7mer-m8	TargetScan

miR-492	ENSG00000196242	7mer-m8	TargetScan
miR-492	ENSG00000196591	7mer-m8	TargetScan
miR-492	ENSG00000196776	7mer-m8	TargetScan
miR-492	ENSG00000196981	7mer-m8	TargetScan
miR-492	ENSG00000197084	7mer-m8	TargetScan
miR-492	ENSG00000197183	7mer-m8	TargetScan
miR-492	ENSG00000197275	7mer-m8	TargetScan
miR-492	ENSG00000197702	7mer-m8	TargetScan
miR-492	ENSG00000197782	7mer-m8	TargetScan
miR-492	ENSG00000197912	7mer-m8	TargetScan
miR-492	ENSG00000198040	7mer-m8	TargetScan
miR-492	ENSG00000198113	7mer-m8	TargetScan
miR-492	ENSG00000198133	7mer-m8	TargetScan
miR-492	ENSG00000198162	7mer-m8	TargetScan
miR-492	ENSG00000198326	7mer-m8	TargetScan
miR-492	ENSG00000198420	7mer-m8	TargetScan
miR-492	ENSG00000198570	7mer-m8	TargetScan
miR-492	ENSG00000198586	7mer-m8	TargetScan
miR-492	ENSG00000198755	7mer-m8	TargetScan
miR-492	ENSG00000198792	7mer-m8	TargetScan
miR-492	ENSG00000198948	7mer-m8	TargetScan
miR-492	ENSG00000203485	7mer-m8	TargetScan
miR-492	ENSG00000203690	7mer-m8	TargetScan
miR-492	ENSG00000204128	7mer-m8	TargetScan
miR-492	ENSG00000204271	7mer-m8	TargetScan
miR-492	ENSG00000204580	7mer-m8	TargetScan
miR-492	ENSG00000204628	7mer-m8	TargetScan
miR-492	ENSG00000205268	7mer-m8	TargetScan
miR-492	ENSG00000205795	7mer-m8	TargetScan
miR-492	ENSG00000205922	7mer-m8	TargetScan
miR-492	ENSG00000206199	7mer-m8	TargetScan

miR-492	ENSG00000213654	7mer-m8	TargetScan
miR-492	ENSG00000213699	7mer-m8	TargetScan
miR-492	ENSG00000213741	7mer-m8	TargetScan
miR-492	ENSG00000214022	7mer-m8	TargetScan
miR-492	ENSG00000214248	7mer-m8	TargetScan
miR-492	ENSG00000214517	7mer-m8	TargetScan
miR-492	ENSG00000214518	7mer-m8	TargetScan
miR-492	ENSG00000214736	7mer-m8	TargetScan
miR-492	ENSG00000215612	7mer-m8	TargetScan
miR-492	ENSG00000217702	7mer-m8	TargetScan
miR-492	ENSG00000218357	7mer-m8	TargetScan
miR-492	ENSG00000235194	7mer-m8	TargetScan
miR-492	ENSG00000237440	7mer-m8	TargetScan
miR-492	ENSG00000237896	7mer-m8	TargetScan
miR-492	ENSG00000241127	7mer-m8	TargetScan
miR-492	ENSG00000241839	7mer-m8	TargetScan
miR-492	ENSG00000244509	7mer-m8	TargetScan
miR-492	ENSG00000245680	7mer-m8	TargetScan
miR-492	ENSG00000248050	7mer-m8	TargetScan
miR-492	ENSG00000248919	7mer-m8	TargetScan
miR-492	ENSG00000249242	7mer-m8	TargetScan
miR-492	ENSG00000255346	7mer-m8	TargetScan
miR-492	ENSG00000258869	7mer-m8	TargetScan
miR-492	ENSG00000258986	7mer-m8	TargetScan
miR-492	ENSG00000260007	7mer-m8	TargetScan
miR-492	ENSG00000265817	7mer-m8	TargetScan
miR-492	ENSG00000267270	7mer-m8	TargetScan
miR-492	ENSG00000267432	7mer-m8	TargetScan
miR-492	ENSG00000267561	7mer-m8	TargetScan
miR-492	ENSG00000272325	7mer-m8	TargetScan
miR-492	ENSG00000272414	7mer-m8	TargetScan

miR-492	ENSG00000277858	7mer-m8	TargetScan
miR-492	ENSG00000283597	7mer-m8	TargetScan
miR-492	ENSG00000288436	7mer-m8	TargetScan
miR-7161-5p	ENSG00000003393	7mer-m8	TargetScan
miR-7161-5p	ENSG00000004897	7mer-m8	TargetScan
miR-7161-5p	ENSG00000005100	7mer-m8	TargetScan
miR-7161-5p	ENSG00000005175	7mer-m8	TargetScan
miR-7161-5p	ENSG00000005812	7mer-m8	TargetScan
miR-7161-5p	ENSG00000005893	7mer-m8	TargetScan
miR-7161-5p	ENSG00000006459	7mer-m8	TargetScan
miR-7161-5p	ENSG00000006468	7mer-m8	TargetScan
miR-7161-5p	ENSG00000006576	7mer-m8	TargetScan
miR-7161-5p	ENSG00000006634	7mer-m8	TargetScan
miR-7161-5p	ENSG00000010244	7mer-m8	TargetScan
miR-7161-5p	ENSG00000010404	7mer-m8	TargetScan
miR-7161-5p	ENSG00000011007	7mer-m8	TargetScan
miR-7161-5p	ENSG00000011258	7mer-m8	TargetScan
miR-7161-5p	ENSG00000013375	7mer-m8	TargetScan
miR-7161-5p	ENSG00000014257	7mer-m8	TargetScan
miR-7161-5p	ENSG00000015153	7mer-m8	TargetScan
miR-7161-5p	ENSG00000015475	7mer-m8	TargetScan
miR-7161-5p	ENSG00000015520	7mer-m8	TargetScan
miR-7161-5p	ENSG00000017427	7mer-m8	TargetScan
miR-7161-5p	ENSG00000018236	7mer-m8	TargetScan
miR-7161-5p	ENSG00000018510	7mer-m8	TargetScan
miR-7161-5p	ENSG00000020922	7mer-m8	TargetScan
miR-7161-5p	ENSG00000023318	7mer-m8	TargetScan
miR-7161-5p	ENSG00000024862	7mer-m8	TargetScan
miR-7161-5p	ENSG00000026652	7mer-m8	TargetScan
miR-7161-5p	ENSG00000028528	7mer-m8	TargetScan
miR-7161-5p	ENSG00000028839	7mer-m8	TargetScan

miR-7161-5p	ENSG00000038210	7mer-m8	TargetScan
miR-7161-5p	ENSG00000039123	7mer-m8	TargetScan
miR-7161-5p	ENSG00000040341	7mer-m8	TargetScan
miR-7161-5p	ENSG00000040933	7mer-m8	TargetScan
miR-7161-5p	ENSG00000042781	7mer-m8	TargetScan
miR-7161-5p	ENSG00000046653	7mer-m8	TargetScan
miR-7161-5p	ENSG00000047346	7mer-m8	TargetScan
miR-7161-5p	ENSG00000048052	7mer-m8	TargetScan
miR-7161-5p	ENSG00000048828	7mer-m8	TargetScan
miR-7161-5p	ENSG00000050393	7mer-m8	TargetScan
miR-7161-5p	ENSG00000050405	7mer-m8	TargetScan
miR-7161-5p	ENSG00000053770	7mer-m8	TargetScan
miR-7161-5p	ENSG00000054965	7mer-m8	TargetScan
miR-7161-5p	ENSG00000057294	7mer-m8	TargetScan
miR-7161-5p	ENSG00000058091	7mer-m8	TargetScan
miR-7161-5p	ENSG00000060140	7mer-m8	TargetScan
miR-7161-5p	ENSG00000061455	7mer-m8	TargetScan
miR-7161-5p	ENSG00000061676	7mer-m8	TargetScan
miR-7161-5p	ENSG00000061987	7mer-m8	TargetScan
miR-7161-5p	ENSG00000064115	7mer-m8	TargetScan
miR-7161-5p	ENSG00000064393	7mer-m8	TargetScan
miR-7161-5p	ENSG00000064545	7mer-m8	TargetScan
miR-7161-5p	ENSG00000065413	7mer-m8	TargetScan
miR-7161-5p	ENSG00000065548	7mer-m8	TargetScan
miR-7161-5p	ENSG00000066084	7mer-m8	TargetScan
miR-7161-5p	ENSG00000066294	7mer-m8	TargetScan
miR-7161-5p	ENSG00000066651	7mer-m8	TargetScan
miR-7161-5p	ENSG00000066697	7mer-m8	TargetScan
miR-7161-5p	ENSG00000066813	7mer-m8	TargetScan
miR-7161-5p	ENSG00000067066	7mer-m8	TargetScan
miR-7161-5p	ENSG00000068024	7mer-m8	TargetScan

miR-7161-5p	ENSG00000068745	7mer-m8	TargetScan
miR-7161-5p	ENSG00000069020	7mer-m8	TargetScan
miR-7161-5p	ENSG00000069275	7mer-m8	TargetScan
miR-7161-5p	ENSG00000069702	7mer-m8	TargetScan
miR-7161-5p	ENSG00000070182	7mer-m8	TargetScan
miR-7161-5p	ENSG00000070193	7mer-m8	TargetScan
miR-7161-5p	ENSG00000070214	7mer-m8	TargetScan
miR-7161-5p	ENSG00000070756	7mer-m8	TargetScan
miR-7161-5p	ENSG00000071073	7mer-m8	TargetScan
miR-7161-5p	ENSG00000071203	7mer-m8	TargetScan
miR-7161-5p	ENSG00000071243	7mer-m8	TargetScan
miR-7161-5p	ENSG00000072042	7mer-m8	TargetScan
miR-7161-5p	ENSG00000072121	7mer-m8	TargetScan
miR-7161-5p	ENSG00000074590	7mer-m8	TargetScan
miR-7161-5p	ENSG00000075303	7mer-m8	TargetScan
miR-7161-5p	ENSG00000075884	7mer-m8	TargetScan
miR-7161-5p	ENSG00000077157	7mer-m8	TargetScan
miR-7161-5p	ENSG00000077232	7mer-m8	TargetScan
miR-7161-5p	ENSG00000077458	7mer-m8	TargetScan
miR-7161-5p	ENSG00000077616	7mer-m8	TargetScan
miR-7161-5p	ENSG00000078549	7mer-m8	TargetScan
miR-7161-5p	ENSG00000080493	7mer-m8	TargetScan
miR-7161-5p	ENSG00000081026	7mer-m8	TargetScan
miR-7161-5p	ENSG00000081059	7mer-m8	TargetScan
miR-7161-5p	ENSG00000081148	7mer-m8	TargetScan
miR-7161-5p	ENSG00000081377	7mer-m8	TargetScan
miR-7161-5p	ENSG00000082258	7mer-m8	TargetScan
miR-7161-5p	ENSG00000083312	7mer-m8	TargetScan
miR-7161-5p	ENSG00000083635	7mer-m8	TargetScan
miR-7161-5p	ENSG00000083844	7mer-m8	TargetScan
miR-7161-5p	ENSG00000084072	7mer-m8	TargetScan

miR-7161-5p	ENSG00000084092	7mer-m8	TargetScan
miR-7161-5p	ENSG00000084674	7mer-m8	TargetScan
miR-7161-5p	ENSG00000085274	7mer-m8	TargetScan
miR-7161-5p	ENSG00000085871	7mer-m8	TargetScan
miR-7161-5p	ENSG00000086300	7mer-m8	TargetScan
miR-7161-5p	ENSG00000086475	7mer-m8	TargetScan
miR-7161-5p	ENSG00000086991	7mer-m8	TargetScan
miR-7161-5p	ENSG00000087263	7mer-m8	TargetScan
miR-7161-5p	ENSG00000087502	7mer-m8	TargetScan
miR-7161-5p	ENSG00000088356	7mer-m8	TargetScan
miR-7161-5p	ENSG00000088387	7mer-m8	TargetScan
miR-7161-5p	ENSG00000088812	7mer-m8	TargetScan
miR-7161-5p	ENSG00000089916	7mer-m8	TargetScan
miR-7161-5p	ENSG00000090615	7mer-m8	TargetScan
miR-7161-5p	ENSG00000091140	7mer-m8	TargetScan
miR-7161-5p	ENSG00000091986	7mer-m8	TargetScan
miR-7161-5p	ENSG00000092871	7mer-m8	TargetScan
miR-7161-5p	ENSG00000092978	7mer-m8	TargetScan
miR-7161-5p	ENSG00000093000	7mer-m8	TargetScan
miR-7161-5p	ENSG00000095203	7mer-m8	TargetScan
miR-7161-5p	ENSG00000095637	7mer-m8	TargetScan
miR-7161-5p	ENSG00000096060	7mer-m8	TargetScan
miR-7161-5p	ENSG00000096063	7mer-m8	TargetScan
miR-7161-5p	ENSG00000096401	7mer-m8	TargetScan
miR-7161-5p	ENSG00000100296	7mer-m8	TargetScan
miR-7161-5p	ENSG00000100335	7mer-m8	TargetScan
miR-7161-5p	ENSG00000100354	7mer-m8	TargetScan
miR-7161-5p	ENSG00000100376	7mer-m8	TargetScan
miR-7161-5p	ENSG00000100433	7mer-m8	TargetScan
miR-7161-5p	ENSG00000100505	7mer-m8	TargetScan
miR-7161-5p	ENSG00000100592	7mer-m8	TargetScan

miR-7161-5p	ENSG00000100600	7mer-m8	TargetScan
miR-7161-5p	ENSG00000100614	7mer-m8	TargetScan
miR-7161-5p	ENSG00000100625	7mer-m8	TargetScan
miR-7161-5p	ENSG00000100664	7mer-m8	TargetScan
miR-7161-5p	ENSG00000100697	7mer-m8	TargetScan
miR-7161-5p	ENSG00000100815	7mer-m8	TargetScan
miR-7161-5p	ENSG00000100934	7mer-m8	TargetScan
miR-7161-5p	ENSG00000101190	7mer-m8	TargetScan
miR-7161-5p	ENSG00000101191	7mer-m8	TargetScan
miR-7161-5p	ENSG00000101336	7mer-m8	TargetScan
miR-7161-5p	ENSG00000101670	7mer-m8	TargetScan
miR-7161-5p	ENSG00000101751	7mer-m8	TargetScan
miR-7161-5p	ENSG00000101938	7mer-m8	TargetScan
miR-7161-5p	ENSG00000102158	7mer-m8	TargetScan
miR-7161-5p	ENSG00000102189	7mer-m8	TargetScan
miR-7161-5p	ENSG00000102226	7mer-m8	TargetScan
miR-7161-5p	ENSG00000102383	7mer-m8	TargetScan
miR-7161-5p	ENSG00000102547	7mer-m8	TargetScan
miR-7161-5p	ENSG00000102781	7mer-m8	TargetScan
miR-7161-5p	ENSG00000102970	7mer-m8	TargetScan
miR-7161-5p	ENSG00000103056	7mer-m8	TargetScan
miR-7161-5p	ENSG00000103067	7mer-m8	TargetScan
miR-7161-5p	ENSG00000103184	7mer-m8	TargetScan
miR-7161-5p	ENSG00000103978	7mer-m8	TargetScan
miR-7161-5p	ENSG00000104290	7mer-m8	TargetScan
miR-7161-5p	ENSG00000104331	7mer-m8	TargetScan
miR-7161-5p	ENSG00000104731	7mer-m8	TargetScan
miR-7161-5p	ENSG00000105220	7mer-m8	TargetScan
miR-7161-5p	ENSG00000105483	7mer-m8	TargetScan
miR-7161-5p	ENSG00000105492	7mer-m8	TargetScan
miR-7161-5p	ENSG00000105497	7mer-m8	TargetScan

miR-7161-5p	ENSG00000105568	7mer-m8	TargetScan
miR-7161-5p	ENSG00000105750	7mer-m8	TargetScan
miR-7161-5p	ENSG00000105821	7mer-m8	TargetScan
miR-7161-5p	ENSG00000105866	7mer-m8	TargetScan
miR-7161-5p	ENSG00000105976	7mer-m8	TargetScan
miR-7161-5p	ENSG00000105983	7mer-m8	TargetScan
miR-7161-5p	ENSG00000106113	7mer-m8	TargetScan
miR-7161-5p	ENSG00000106460	7mer-m8	TargetScan
miR-7161-5p	ENSG00000106665	7mer-m8	TargetScan
miR-7161-5p	ENSG00000106723	7mer-m8	TargetScan
miR-7161-5p	ENSG00000106772	7mer-m8	TargetScan
miR-7161-5p	ENSG00000106799	7mer-m8	TargetScan
miR-7161-5p	ENSG00000107105	7mer-m8	TargetScan
miR-7161-5p	ENSG00000107443	7mer-m8	TargetScan
miR-7161-5p	ENSG00000107562	7mer-m8	TargetScan
miR-7161-5p	ENSG00000107581	7mer-m8	TargetScan
miR-7161-5p	ENSG00000107651	7mer-m8	TargetScan
miR-7161-5p	ENSG00000107960	7mer-m8	TargetScan
miR-7161-5p	ENSG00000108061	7mer-m8	TargetScan
miR-7161-5p	ENSG00000108924	7mer-m8	TargetScan
miR-7161-5p	ENSG00000109158	7mer-m8	TargetScan
miR-7161-5p	ENSG00000109220	7mer-m8	TargetScan
miR-7161-5p	ENSG00000109265	7mer-m8	TargetScan
miR-7161-5p	ENSG00000109458	7mer-m8	TargetScan
miR-7161-5p	ENSG00000109466	7mer-m8	TargetScan
miR-7161-5p	ENSG00000109854	7mer-m8	TargetScan
miR-7161-5p	ENSG00000109861	7mer-m8	TargetScan
miR-7161-5p	ENSG00000109906	7mer-m8	TargetScan
miR-7161-5p	ENSG00000110172	7mer-m8	TargetScan
miR-7161-5p	ENSG00000110427	7mer-m8	TargetScan
miR-7161-5p	ENSG00000110429	7mer-m8	TargetScan

miR-7161-5p	ENSG00000110436	7mer-m8	TargetScan
miR-7161-5p	ENSG00000110665	7mer-m8	TargetScan
miR-7161-5p	ENSG00000110693	7mer-m8	TargetScan
miR-7161-5p	ENSG00000110848	7mer-m8	TargetScan
miR-7161-5p	ENSG00000110876	7mer-m8	TargetScan
miR-7161-5p	ENSG00000111110	7mer-m8	TargetScan
miR-7161-5p	ENSG00000111371	7mer-m8	TargetScan
miR-7161-5p	ENSG00000111700	7mer-m8	TargetScan
miR-7161-5p	ENSG00000111816	7mer-m8	TargetScan
miR-7161-5p	ENSG00000111817	7mer-m8	TargetScan
miR-7161-5p	ENSG00000111837	7mer-m8	TargetScan
miR-7161-5p	ENSG00000111879	7mer-m8	TargetScan
miR-7161-5p	ENSG00000112038	7mer-m8	TargetScan
miR-7161-5p	ENSG00000112062	7mer-m8	TargetScan
miR-7161-5p	ENSG00000112081	7mer-m8	TargetScan
miR-7161-5p	ENSG00000112096	7mer-m8	TargetScan
miR-7161-5p	ENSG00000112200	7mer-m8	TargetScan
miR-7161-5p	ENSG00000112214	7mer-m8	TargetScan
miR-7161-5p	ENSG00000112218	7mer-m8	TargetScan
miR-7161-5p	ENSG00000112339	7mer-m8	TargetScan
miR-7161-5p	ENSG00000112394	7mer-m8	TargetScan
miR-7161-5p	ENSG00000112531	7mer-m8	TargetScan
miR-7161-5p	ENSG00000112695	7mer-m8	TargetScan
miR-7161-5p	ENSG00000112851	7mer-m8	TargetScan
miR-7161-5p	ENSG00000112852	7mer-m8	TargetScan
miR-7161-5p	ENSG00000113300	7mer-m8	TargetScan
miR-7161-5p	ENSG00000113319	7mer-m8	TargetScan
miR-7161-5p	ENSG00000113441	7mer-m8	TargetScan
miR-7161-5p	ENSG00000113448	7mer-m8	TargetScan
miR-7161-5p	ENSG00000113594	7mer-m8	TargetScan
miR-7161-5p	ENSG00000113719	7mer-m8	TargetScan

miR-7161-5p	ENSG00000114416	7mer-m8	TargetScan
miR-7161-5p	ENSG00000114503	7mer-m8	TargetScan
miR-7161-5p	ENSG00000114686	7mer-m8	TargetScan
miR-7161-5p	ENSG00000114861	7mer-m8	TargetScan
miR-7161-5p	ENSG00000115020	7mer-m8	TargetScan
miR-7161-5p	ENSG00000115365	7mer-m8	TargetScan
miR-7161-5p	ENSG00000115461	7mer-m8	TargetScan
miR-7161-5p	ENSG00000115474	7mer-m8	TargetScan
miR-7161-5p	ENSG00000115561	7mer-m8	TargetScan
miR-7161-5p	ENSG00000115977	7mer-m8	TargetScan
miR-7161-5p	ENSG00000116001	7mer-m8	TargetScan
miR-7161-5p	ENSG00000116005	7mer-m8	TargetScan
miR-7161-5p	ENSG00000116062	7mer-m8	TargetScan
miR-7161-5p	ENSG00000116191	7mer-m8	TargetScan
miR-7161-5p	ENSG00000116198	7mer-m8	TargetScan
miR-7161-5p	ENSG00000116209	7mer-m8	TargetScan
miR-7161-5p	ENSG00000116679	7mer-m8	TargetScan
miR-7161-5p	ENSG00000116954	7mer-m8	TargetScan
miR-7161-5p	ENSG00000116984	7mer-m8	TargetScan
miR-7161-5p	ENSG00000117020	7mer-m8	TargetScan
miR-7161-5p	ENSG00000117114	7mer-m8	TargetScan
miR-7161-5p	ENSG00000117152	7mer-m8	TargetScan
miR-7161-5p	ENSG00000117450	7mer-m8	TargetScan
miR-7161-5p	ENSG00000117505	7mer-m8	TargetScan
miR-7161-5p	ENSG00000117569	7mer-m8	TargetScan
miR-7161-5p	ENSG00000117602	7mer-m8	TargetScan
miR-7161-5p	ENSG00000117616	7mer-m8	TargetScan
miR-7161-5p	ENSG00000118200	7mer-m8	TargetScan
miR-7161-5p	ENSG00000118217	7mer-m8	TargetScan
miR-7161-5p	ENSG00000118242	7mer-m8	TargetScan
miR-7161-5p	ENSG00000118260	7mer-m8	TargetScan

miR-7161-5p	ENSG00000118432	7mer-m8	TargetScan
miR-7161-5p	ENSG00000118508	7mer-m8	TargetScan
miR-7161-5p	ENSG00000118579	7mer-m8	TargetScan
miR-7161-5p	ENSG00000118733	7mer-m8	TargetScan
miR-7161-5p	ENSG00000118939	7mer-m8	TargetScan
miR-7161-5p	ENSG00000118960	7mer-m8	TargetScan
miR-7161-5p	ENSG00000119004	7mer-m8	TargetScan
miR-7161-5p	ENSG00000119280	7mer-m8	TargetScan
miR-7161-5p	ENSG00000119283	7mer-m8	TargetScan
miR-7161-5p	ENSG00000119321	7mer-m8	TargetScan
miR-7161-5p	ENSG00000119508	7mer-m8	TargetScan
miR-7161-5p	ENSG00000119638	7mer-m8	TargetScan
miR-7161-5p	ENSG00000119673	7mer-m8	TargetScan
miR-7161-5p	ENSG00000119760	7mer-m8	TargetScan
miR-7161-5p	ENSG00000119900	7mer-m8	TargetScan
miR-7161-5p	ENSG00000119953	7mer-m8	TargetScan
miR-7161-5p	ENSG00000119969	7mer-m8	TargetScan
miR-7161-5p	ENSG00000120694	7mer-m8	TargetScan
miR-7161-5p	ENSG00000120742	7mer-m8	TargetScan
miR-7161-5p	ENSG00000120798	7mer-m8	TargetScan
miR-7161-5p	ENSG00000121274	7mer-m8	TargetScan
miR-7161-5p	ENSG00000121454	7mer-m8	TargetScan
miR-7161-5p	ENSG00000121671	7mer-m8	TargetScan
miR-7161-5p	ENSG00000121864	7mer-m8	TargetScan
miR-7161-5p	ENSG00000121879	7mer-m8	TargetScan
miR-7161-5p	ENSG00000122406	7mer-m8	TargetScan
miR-7161-5p	ENSG00000122733	7mer-m8	TargetScan
miR-7161-5p	ENSG00000122741	7mer-m8	TargetScan
miR-7161-5p	ENSG00000123080	7mer-m8	TargetScan
miR-7161-5p	ENSG00000123104	7mer-m8	TargetScan
miR-7161-5p	ENSG00000123119	7mer-m8	TargetScan

miR-7161-5p	ENSG00000123200	7mer-m8	TargetScan
miR-7161-5p	ENSG00000123352	7mer-m8	TargetScan
miR-7161-5p	ENSG00000123836	7mer-m8	TargetScan
miR-7161-5p	ENSG00000124172	7mer-m8	TargetScan
miR-7161-5p	ENSG00000124193	7mer-m8	TargetScan
miR-7161-5p	ENSG00000124224	7mer-m8	TargetScan
miR-7161-5p	ENSG00000124356	7mer-m8	TargetScan
miR-7161-5p	ENSG00000124374	7mer-m8	TargetScan
miR-7161-5p	ENSG00000124406	7mer-m8	TargetScan
miR-7161-5p	ENSG00000124571	7mer-m8	TargetScan
miR-7161-5p	ENSG00000124731	7mer-m8	TargetScan
miR-7161-5p	ENSG00000124767	7mer-m8	TargetScan
miR-7161-5p	ENSG00000124783	7mer-m8	TargetScan
miR-7161-5p	ENSG00000125249	7mer-m8	TargetScan
miR-7161-5p	ENSG00000125637	7mer-m8	TargetScan
miR-7161-5p	ENSG00000125863	7mer-m8	TargetScan
miR-7161-5p	ENSG00000125885	7mer-m8	TargetScan
miR-7161-5p	ENSG00000125970	7mer-m8	TargetScan
miR-7161-5p	ENSG00000126012	7mer-m8	TargetScan
miR-7161-5p	ENSG00000126070	7mer-m8	TargetScan
miR-7161-5p	ENSG00000126217	7mer-m8	TargetScan
miR-7161-5p	ENSG00000126953	7mer-m8	TargetScan
miR-7161-5p	ENSG00000127022	7mer-m8	TargetScan
miR-7161-5p	ENSG00000127083	7mer-m8	TargetScan
miR-7161-5p	ENSG00000127980	7mer-m8	TargetScan
miR-7161-5p	ENSG00000128000	7mer-m8	TargetScan
miR-7161-5p	ENSG00000128534	7mer-m8	TargetScan
miR-7161-5p	ENSG00000128923	7mer-m8	TargetScan
miR-7161-5p	ENSG00000129250	7mer-m8	TargetScan
miR-7161-5p	ENSG00000129315	7mer-m8	TargetScan
miR-7161-5p	ENSG00000129422	7mer-m8	TargetScan

miR-7161-5p	ENSG00000129657	7mer-m8	TargetScan
miR-7161-5p	ENSG00000130021	7mer-m8	TargetScan
miR-7161-5p	ENSG00000130035	7mer-m8	TargetScan
miR-7161-5p	ENSG00000130147	7mer-m8	TargetScan
miR-7161-5p	ENSG00000130348	7mer-m8	TargetScan
miR-7161-5p	ENSG00000130363	7mer-m8	TargetScan
miR-7161-5p	ENSG00000131013	7mer-m8	TargetScan
miR-7161-5p	ENSG00000131127	7mer-m8	TargetScan
miR-7161-5p	ENSG00000131368	7mer-m8	TargetScan
miR-7161-5p	ENSG00000131370	7mer-m8	TargetScan
miR-7161-5p	ENSG00000131503	7mer-m8	TargetScan
miR-7161-5p	ENSG00000131507	7mer-m8	TargetScan
miR-7161-5p	ENSG00000131724	7mer-m8	TargetScan
miR-7161-5p	ENSG00000131845	7mer-m8	TargetScan
miR-7161-5p	ENSG00000131979	7mer-m8	TargetScan
miR-7161-5p	ENSG00000132185	7mer-m8	TargetScan
miR-7161-5p	ENSG00000132256	7mer-m8	TargetScan
miR-7161-5p	ENSG00000132300	7mer-m8	TargetScan
miR-7161-5p	ENSG00000132334	7mer-m8	TargetScan
miR-7161-5p	ENSG00000132436	7mer-m8	TargetScan
miR-7161-5p	ENSG00000132603	7mer-m8	TargetScan
miR-7161-5p	ENSG00000132676	7mer-m8	TargetScan
miR-7161-5p	ENSG00000132953	7mer-m8	TargetScan
miR-7161-5p	ENSG00000133107	7mer-m8	TargetScan
miR-7161-5p	ENSG00000133124	7mer-m8	TargetScan
miR-7161-5p	ENSG00000133858	7mer-m8	TargetScan
miR-7161-5p	ENSG00000133863	7mer-m8	TargetScan
miR-7161-5p	ENSG00000133895	7mer-m8	TargetScan
miR-7161-5p	ENSG00000133997	7mer-m8	TargetScan
miR-7161-5p	ENSG00000134014	7mer-m8	TargetScan
miR-7161-5p	ENSG00000134152	7mer-m8	TargetScan

miR-7161-5p	ENSG00000134250	7mer-m8	TargetScan
miR-7161-5p	ENSG00000134253	7mer-m8	TargetScan
miR-7161-5p	ENSG00000134265	7mer-m8	TargetScan
miR-7161-5p	ENSG00000134333	7mer-m8	TargetScan
miR-7161-5p	ENSG00000134461	7mer-m8	TargetScan
miR-7161-5p	ENSG00000134533	7mer-m8	TargetScan
miR-7161-5p	ENSG00000134809	7mer-m8	TargetScan
miR-7161-5p	ENSG00000134884	7mer-m8	TargetScan
miR-7161-5p	ENSG00000134909	7mer-m8	TargetScan
miR-7161-5p	ENSG00000135040	7mer-m8	TargetScan
miR-7161-5p	ENSG00000135272	7mer-m8	TargetScan
miR-7161-5p	ENSG00000135297	7mer-m8	TargetScan
miR-7161-5p	ENSG00000135315	7mer-m8	TargetScan
miR-7161-5p	ENSG00000135452	7mer-m8	TargetScan
miR-7161-5p	ENSG00000135540	7mer-m8	TargetScan
miR-7161-5p	ENSG00000135547	7mer-m8	TargetScan
miR-7161-5p	ENSG00000135604	7mer-m8	TargetScan
miR-7161-5p	ENSG00000135953	7mer-m8	TargetScan
miR-7161-5p	ENSG00000136243	7mer-m8	TargetScan
miR-7161-5p	ENSG00000136383	7mer-m8	TargetScan
miR-7161-5p	ENSG00000136436	7mer-m8	TargetScan
miR-7161-5p	ENSG00000136450	7mer-m8	TargetScan
miR-7161-5p	ENSG00000136631	7mer-m8	TargetScan
miR-7161-5p	ENSG00000136688	7mer-m8	TargetScan
miR-7161-5p	ENSG00000136709	7mer-m8	TargetScan
miR-7161-5p	ENSG00000136807	7mer-m8	TargetScan
miR-7161-5p	ENSG00000136881	7mer-m8	TargetScan
miR-7161-5p	ENSG00000137040	7mer-m8	TargetScan
miR-7161-5p	ENSG00000137135	7mer-m8	TargetScan
miR-7161-5p	ENSG00000137393	7mer-m8	TargetScan
miR-7161-5p	ENSG00000137500	7mer-m8	TargetScan

miR-7161-5p	ENSG00000137502	7mer-m8	TargetScan
miR-7161-5p	ENSG00000137601	7mer-m8	TargetScan
miR-7161-5p	ENSG00000137692	7mer-m8	TargetScan
miR-7161-5p	ENSG00000137713	7mer-m8	TargetScan
miR-7161-5p	ENSG00000137770	7mer-m8	TargetScan
miR-7161-5p	ENSG00000137878	7mer-m8	TargetScan
miR-7161-5p	ENSG00000137959	7mer-m8	TargetScan
miR-7161-5p	ENSG00000137975	7mer-m8	TargetScan
miR-7161-5p	ENSG00000138032	7mer-m8	TargetScan
miR-7161-5p	ENSG00000138071	7mer-m8	TargetScan
miR-7161-5p	ENSG00000138190	7mer-m8	TargetScan
miR-7161-5p	ENSG00000138363	7mer-m8	TargetScan
miR-7161-5p	ENSG00000138442	7mer-m8	TargetScan
miR-7161-5p	ENSG00000138641	7mer-m8	TargetScan
miR-7161-5p	ENSG00000138650	7mer-m8	TargetScan
miR-7161-5p	ENSG00000138757	7mer-m8	TargetScan
miR-7161-5p	ENSG00000138778	7mer-m8	TargetScan
miR-7161-5p	ENSG00000138798	7mer-m8	TargetScan
miR-7161-5p	ENSG00000138821	7mer-m8	TargetScan
miR-7161-5p	ENSG00000139083	7mer-m8	TargetScan
miR-7161-5p	ENSG00000139146	7mer-m8	TargetScan
miR-7161-5p	ENSG00000139163	7mer-m8	TargetScan
miR-7161-5p	ENSG00000139263	7mer-m8	TargetScan
miR-7161-5p	ENSG00000139318	7mer-m8	TargetScan
miR-7161-5p	ENSG00000139351	7mer-m8	TargetScan
miR-7161-5p	ENSG00000139620	7mer-m8	TargetScan
miR-7161-5p	ENSG00000139631	7mer-m8	TargetScan
miR-7161-5p	ENSG00000139679	7mer-m8	TargetScan
miR-7161-5p	ENSG00000139734	7mer-m8	TargetScan
miR-7161-5p	ENSG00000139977	7mer-m8	TargetScan
miR-7161-5p	ENSG00000140280	7mer-m8	TargetScan

miR-7161-5p	ENSG00000140403	7mer-m8	TargetScan
miR-7161-5p	ENSG00000140406	7mer-m8	TargetScan
miR-7161-5p	ENSG00000140941	7mer-m8	TargetScan
miR-7161-5p	ENSG00000141404	7mer-m8	TargetScan
miR-7161-5p	ENSG00000141441	7mer-m8	TargetScan
miR-7161-5p	ENSG00000141446	7mer-m8	TargetScan
miR-7161-5p	ENSG00000141449	7mer-m8	TargetScan
miR-7161-5p	ENSG00000141655	7mer-m8	TargetScan
miR-7161-5p	ENSG00000141858	7mer-m8	TargetScan
miR-7161-5p	ENSG00000141873	7mer-m8	TargetScan
miR-7161-5p	ENSG00000142192	7mer-m8	TargetScan
miR-7161-5p	ENSG00000142556	7mer-m8	TargetScan
miR-7161-5p	ENSG00000142621	7mer-m8	TargetScan
miR-7161-5p	ENSG00000142623	7mer-m8	TargetScan
miR-7161-5p	ENSG00000143061	7mer-m8	TargetScan
miR-7161-5p	ENSG00000143420	7mer-m8	TargetScan
miR-7161-5p	ENSG00000143493	7mer-m8	TargetScan
miR-7161-5p	ENSG00000143627	7mer-m8	TargetScan
miR-7161-5p	ENSG00000143786	7mer-m8	TargetScan
miR-7161-5p	ENSG00000143815	7mer-m8	TargetScan
miR-7161-5p	ENSG00000143995	7mer-m8	TargetScan
miR-7161-5p	ENSG00000144057	7mer-m8	TargetScan
miR-7161-5p	ENSG00000144229	7mer-m8	TargetScan
miR-7161-5p	ENSG00000144366	7mer-m8	TargetScan
miR-7161-5p	ENSG00000144401	7mer-m8	TargetScan
miR-7161-5p	ENSG00000144451	7mer-m8	TargetScan
miR-7161-5p	ENSG00000144455	7mer-m8	TargetScan
miR-7161-5p	ENSG00000144597	7mer-m8	TargetScan
miR-7161-5p	ENSG00000144645	7mer-m8	TargetScan
miR-7161-5p	ENSG00000144824	7mer-m8	TargetScan
miR-7161-5p	ENSG00000144959	7mer-m8	TargetScan

miR-7161-5p	ENSG00000145012	7mer-m8	TargetScan
miR-7161-5p	ENSG00000145014	7mer-m8	TargetScan
miR-7161-5p	ENSG00000145147	7mer-m8	TargetScan
miR-7161-5p	ENSG00000145216	7mer-m8	TargetScan
miR-7161-5p	ENSG00000145384	7mer-m8	TargetScan
miR-7161-5p	ENSG00000145414	7mer-m8	TargetScan
miR-7161-5p	ENSG00000145439	7mer-m8	TargetScan
miR-7161-5p	ENSG00000145451	7mer-m8	TargetScan
miR-7161-5p	ENSG00000145592	7mer-m8	TargetScan
miR-7161-5p	ENSG00000145687	7mer-m8	TargetScan
miR-7161-5p	ENSG00000145907	7mer-m8	TargetScan
miR-7161-5p	ENSG00000146072	7mer-m8	TargetScan
miR-7161-5p	ENSG00000146263	7mer-m8	TargetScan
miR-7161-5p	ENSG00000146267	7mer-m8	TargetScan
miR-7161-5p	ENSG00000146282	7mer-m8	TargetScan
miR-7161-5p	ENSG00000146350	7mer-m8	TargetScan
miR-7161-5p	ENSG00000146411	7mer-m8	TargetScan
miR-7161-5p	ENSG00000147119	7mer-m8	TargetScan
miR-7161-5p	ENSG00000147138	7mer-m8	TargetScan
miR-7161-5p	ENSG00000147548	7mer-m8	TargetScan
miR-7161-5p	ENSG00000147604	7mer-m8	TargetScan
miR-7161-5p	ENSG00000147679	7mer-m8	TargetScan
miR-7161-5p	ENSG00000147687	7mer-m8	TargetScan
miR-7161-5p	ENSG00000147852	7mer-m8	TargetScan
miR-7161-5p	ENSG00000147853	7mer-m8	TargetScan
miR-7161-5p	ENSG00000147862	7mer-m8	TargetScan
miR-7161-5p	ENSG00000148187	7mer-m8	TargetScan
miR-7161-5p	ENSG00000148225	7mer-m8	TargetScan
miR-7161-5p	ENSG00000148600	7mer-m8	TargetScan
miR-7161-5p	ENSG00000148672	7mer-m8	TargetScan
miR-7161-5p	ENSG00000148688	7mer-m8	TargetScan

miR-7161-5p	ENSG00000148730	7mer-m8	TargetScan
miR-7161-5p	ENSG00000148737	7mer-m8	TargetScan
miR-7161-5p	ENSG00000149212	7mer-m8	TargetScan
miR-7161-5p	ENSG00000149308	7mer-m8	TargetScan
miR-7161-5p	ENSG00000149483	7mer-m8	TargetScan
miR-7161-5p	ENSG00000149948	7mer-m8	TargetScan
miR-7161-5p	ENSG00000150681	7mer-m8	TargetScan
miR-7161-5p	ENSG00000150938	7mer-m8	TargetScan
miR-7161-5p	ENSG00000151466	7mer-m8	TargetScan
miR-7161-5p	ENSG00000151657	7mer-m8	TargetScan
miR-7161-5p	ENSG00000151692	7mer-m8	TargetScan
miR-7161-5p	ENSG00000151729	7mer-m8	TargetScan
miR-7161-5p	ENSG00000151746	7mer-m8	TargetScan
miR-7161-5p	ENSG00000151834	7mer-m8	TargetScan
miR-7161-5p	ENSG00000152402	7mer-m8	TargetScan
miR-7161-5p	ENSG00000152492	7mer-m8	TargetScan
miR-7161-5p	ENSG00000152582	7mer-m8	TargetScan
miR-7161-5p	ENSG00000152670	7mer-m8	TargetScan
miR-7161-5p	ENSG00000152766	7mer-m8	TargetScan
miR-7161-5p	ENSG00000153046	7mer-m8	TargetScan
miR-7161-5p	ENSG00000153140	7mer-m8	TargetScan
miR-7161-5p	ENSG00000153790	7mer-m8	TargetScan
miR-7161-5p	ENSG00000153898	7mer-m8	TargetScan
miR-7161-5p	ENSG00000153904	7mer-m8	TargetScan
miR-7161-5p	ENSG00000154415	7mer-m8	TargetScan
miR-7161-5p	ENSG00000154429	7mer-m8	TargetScan
miR-7161-5p	ENSG00000154640	7mer-m8	TargetScan
miR-7161-5p	ENSG00000154654	7mer-m8	TargetScan
miR-7161-5p	ENSG00000154734	7mer-m8	TargetScan
miR-7161-5p	ENSG00000154845	7mer-m8	TargetScan
miR-7161-5p	ENSG00000155085	7mer-m8	TargetScan

miR-7161-5p	ENSG00000155099	7mer-m8	TargetScan
miR-7161-5p	ENSG00000155380	7mer-m8	TargetScan
miR-7161-5p	ENSG00000155438	7mer-m8	TargetScan
miR-7161-5p	ENSG00000155629	7mer-m8	TargetScan
miR-7161-5p	ENSG00000155827	7mer-m8	TargetScan
miR-7161-5p	ENSG00000155850	7mer-m8	TargetScan
miR-7161-5p	ENSG00000155876	7mer-m8	TargetScan
miR-7161-5p	ENSG00000155886	7mer-m8	TargetScan
miR-7161-5p	ENSG00000155903	7mer-m8	TargetScan
miR-7161-5p	ENSG00000155957	7mer-m8	TargetScan
miR-7161-5p	ENSG00000156030	7mer-m8	TargetScan
miR-7161-5p	ENSG00000156973	7mer-m8	TargetScan
miR-7161-5p	ENSG00000157224	7mer-m8	TargetScan
miR-7161-5p	ENSG00000157429	7mer-m8	TargetScan
miR-7161-5p	ENSG00000157483	7mer-m8	TargetScan
miR-7161-5p	ENSG00000157542	7mer-m8	TargetScan
miR-7161-5p	ENSG00000157916	7mer-m8	TargetScan
miR-7161-5p	ENSG00000158042	7mer-m8	TargetScan
miR-7161-5p	ENSG00000159212	7mer-m8	TargetScan
miR-7161-5p	ENSG00000159256	7mer-m8	TargetScan
miR-7161-5p	ENSG00000159388	7mer-m8	TargetScan
miR-7161-5p	ENSG00000159579	7mer-m8	TargetScan
miR-7161-5p	ENSG00000160216	7mer-m8	TargetScan
miR-7161-5p	ENSG00000160218	7mer-m8	TargetScan
miR-7161-5p	ENSG00000160310	7mer-m8	TargetScan
miR-7161-5p	ENSG00000160321	7mer-m8	TargetScan
miR-7161-5p	ENSG00000160551	7mer-m8	TargetScan
miR-7161-5p	ENSG00000161405	7mer-m8	TargetScan
miR-7161-5p	ENSG00000161551	7mer-m8	TargetScan
miR-7161-5p	ENSG00000161654	7mer-m8	TargetScan
miR-7161-5p	ENSG00000162105	7mer-m8	TargetScan

miR-7161-5p	ENSG00000162367	7mer-m8	TargetScan
miR-7161-5p	ENSG00000162374	7mer-m8	TargetScan
miR-7161-5p	ENSG00000162433	7mer-m8	TargetScan
miR-7161-5p	ENSG00000162599	7mer-m8	TargetScan
miR-7161-5p	ENSG00000162614	7mer-m8	TargetScan
miR-7161-5p	ENSG00000162654	7mer-m8	TargetScan
miR-7161-5p	ENSG00000162849	7mer-m8	TargetScan
miR-7161-5p	ENSG00000162877	7mer-m8	TargetScan
miR-7161-5p	ENSG00000163026	7mer-m8	TargetScan
miR-7161-5p	ENSG00000163125	7mer-m8	TargetScan
miR-7161-5p	ENSG00000163131	7mer-m8	TargetScan
miR-7161-5p	ENSG00000163162	7mer-m8	TargetScan
miR-7161-5p	ENSG00000163378	7mer-m8	TargetScan
miR-7161-5p	ENSG00000163428	7mer-m8	TargetScan
miR-7161-5p	ENSG00000163519	7mer-m8	TargetScan
miR-7161-5p	ENSG00000163558	7mer-m8	TargetScan
miR-7161-5p	ENSG00000163590	7mer-m8	TargetScan
miR-7161-5p	ENSG00000163644	7mer-m8	TargetScan
miR-7161-5p	ENSG00000163646	7mer-m8	TargetScan
miR-7161-5p	ENSG00000163660	7mer-m8	TargetScan
miR-7161-5p	ENSG00000163735	7mer-m8	TargetScan
miR-7161-5p	ENSG00000163755	7mer-m8	TargetScan
miR-7161-5p	ENSG00000163832	7mer-m8	TargetScan
miR-7161-5p	ENSG00000163866	7mer-m8	TargetScan
miR-7161-5p	ENSG00000163950	7mer-m8	TargetScan
miR-7161-5p	ENSG00000164035	7mer-m8	TargetScan
miR-7161-5p	ENSG00000164048	7mer-m8	TargetScan
miR-7161-5p	ENSG00000164070	7mer-m8	TargetScan
miR-7161-5p	ENSG00000164118	7mer-m8	TargetScan
miR-7161-5p	ENSG00000164161	7mer-m8	TargetScan
miR-7161-5p	ENSG00000164167	7mer-m8	TargetScan

miR-7161-5p	ENSG00000164168	7mer-m8	TargetScan
miR-7161-5p	ENSG00000164180	7mer-m8	TargetScan
miR-7161-5p	ENSG00000164181	7mer-m8	TargetScan
miR-7161-5p	ENSG00000164253	7mer-m8	TargetScan
miR-7161-5p	ENSG00000164292	7mer-m8	TargetScan
miR-7161-5p	ENSG00000164294	7mer-m8	TargetScan
miR-7161-5p	ENSG00000164303	7mer-m8	TargetScan
miR-7161-5p	ENSG00000164402	7mer-m8	TargetScan
miR-7161-5p	ENSG00000164442	7mer-m8	TargetScan
miR-7161-5p	ENSG00000164659	7mer-m8	TargetScan
miR-7161-5p	ENSG00000164663	7mer-m8	TargetScan
miR-7161-5p	ENSG00000164684	7mer-m8	TargetScan
miR-7161-5p	ENSG00000164715	7mer-m8	TargetScan
miR-7161-5p	ENSG00000165023	7mer-m8	TargetScan
miR-7161-5p	ENSG00000165028	7mer-m8	TargetScan
miR-7161-5p	ENSG00000165061	7mer-m8	TargetScan
miR-7161-5p	ENSG00000165105	7mer-m8	TargetScan
miR-7161-5p	ENSG00000165186	7mer-m8	TargetScan
miR-7161-5p	ENSG00000165219	7mer-m8	TargetScan
miR-7161-5p	ENSG00000165521	7mer-m8	TargetScan
miR-7161-5p	ENSG00000165526	7mer-m8	TargetScan
miR-7161-5p	ENSG00000165678	7mer-m8	TargetScan
miR-7161-5p	ENSG00000165682	7mer-m8	TargetScan
miR-7161-5p	ENSG00000165694	7mer-m8	TargetScan
miR-7161-5p	ENSG00000165732	7mer-m8	TargetScan
miR-7161-5p	ENSG00000165819	7mer-m8	TargetScan
miR-7161-5p	ENSG00000165891	7mer-m8	TargetScan
miR-7161-5p	ENSG00000165895	7mer-m8	TargetScan
miR-7161-5p	ENSG00000165972	7mer-m8	TargetScan
miR-7161-5p	ENSG00000165985	7mer-m8	TargetScan
miR-7161-5p	ENSG00000166135	7mer-m8	TargetScan

miR-7161-5p	ENSG00000166173	7mer-m8	TargetScan
miR-7161-5p	ENSG00000166200	7mer-m8	TargetScan
miR-7161-5p	ENSG00000166446	7mer-m8	TargetScan
miR-7161-5p	ENSG00000166471	7mer-m8	TargetScan
miR-7161-5p	ENSG00000166479	7mer-m8	TargetScan
miR-7161-5p	ENSG00000166762	7mer-m8	TargetScan
miR-7161-5p	ENSG00000166793	7mer-m8	TargetScan
miR-7161-5p	ENSG00000167555	7mer-m8	TargetScan
miR-7161-5p	ENSG00000167695	7mer-m8	TargetScan
miR-7161-5p	ENSG00000167702	7mer-m8	TargetScan
miR-7161-5p	ENSG00000167985	7mer-m8	TargetScan
miR-7161-5p	ENSG00000168010	7mer-m8	TargetScan
miR-7161-5p	ENSG00000168079	7mer-m8	TargetScan
miR-7161-5p	ENSG00000168172	7mer-m8	TargetScan
miR-7161-5p	ENSG00000168556	7mer-m8	TargetScan
miR-7161-5p	ENSG00000168672	7mer-m8	TargetScan
miR-7161-5p	ENSG00000168772	7mer-m8	TargetScan
miR-7161-5p	ENSG00000168792	7mer-m8	TargetScan
miR-7161-5p	ENSG00000168876	7mer-m8	TargetScan
miR-7161-5p	ENSG00000168887	7mer-m8	TargetScan
miR-7161-5p	ENSG00000168939	7mer-m8	TargetScan
miR-7161-5p	ENSG00000168939	7mer-m8	TargetScan
miR-7161-5p	ENSG00000168955	7mer-m8	TargetScan
miR-7161-5p	ENSG00000169021	7mer-m8	TargetScan
miR-7161-5p	ENSG00000169035	7mer-m8	TargetScan
miR-7161-5p	ENSG00000169057	7mer-m8	TargetScan
miR-7161-5p	ENSG00000169139	7mer-m8	TargetScan
miR-7161-5p	ENSG00000169224	7mer-m8	TargetScan
miR-7161-5p	ENSG00000169446	7mer-m8	TargetScan
miR-7161-5p	ENSG00000169604	7mer-m8	TargetScan
miR-7161-5p	ENSG00000169752	7mer-m8	TargetScan

miR-7161-5p	ENSG00000169891	7mer-m8	TargetScan
miR-7161-5p	ENSG00000169967	7mer-m8	TargetScan
miR-7161-5p	ENSG00000170006	7mer-m8	TargetScan
miR-7161-5p	ENSG00000170027	7mer-m8	TargetScan
miR-7161-5p	ENSG00000170145	7mer-m8	TargetScan
miR-7161-5p	ENSG00000170160	7mer-m8	TargetScan
miR-7161-5p	ENSG00000170248	7mer-m8	TargetScan
miR-7161-5p	ENSG00000170312	7mer-m8	TargetScan
miR-7161-5p	ENSG00000170325	7mer-m8	TargetScan
miR-7161-5p	ENSG00000170571	7mer-m8	TargetScan
miR-7161-5p	ENSG00000170584	7mer-m8	TargetScan
miR-7161-5p	ENSG00000170632	7mer-m8	TargetScan
miR-7161-5p	ENSG00000170734	7mer-m8	TargetScan
miR-7161-5p	ENSG00000170759	7mer-m8	TargetScan
miR-7161-5p	ENSG00000171105	7mer-m8	TargetScan
miR-7161-5p	ENSG00000171121	7mer-m8	TargetScan
miR-7161-5p	ENSG00000171316	7mer-m8	TargetScan
miR-7161-5p	ENSG00000171320	7mer-m8	TargetScan
miR-7161-5p	ENSG00000171444	7mer-m8	TargetScan
miR-7161-5p	ENSG00000171469	7mer-m8	TargetScan
miR-7161-5p	ENSG00000171488	7mer-m8	TargetScan
miR-7161-5p	ENSG00000171502	7mer-m8	TargetScan
miR-7161-5p	ENSG00000171522	7mer-m8	TargetScan
miR-7161-5p	ENSG00000171540	7mer-m8	TargetScan
miR-7161-5p	ENSG00000171759	7mer-m8	TargetScan
miR-7161-5p	ENSG00000171791	7mer-m8	TargetScan
miR-7161-5p	ENSG00000171914	7mer-m8	TargetScan
miR-7161-5p	ENSG00000172469	7mer-m8	TargetScan
miR-7161-5p	ENSG00000172476	7mer-m8	TargetScan
miR-7161-5p	ENSG00000172572	7mer-m8	TargetScan
miR-7161-5p	ENSG00000172845	7mer-m8	TargetScan

miR-7161-5p	ENSG00000172901	7mer-m8	TargetScan
miR-7161-5p	ENSG00000172939	7mer-m8	TargetScan
miR-7161-5p	ENSG00000172954	7mer-m8	TargetScan
miR-7161-5p	ENSG00000172985	7mer-m8	TargetScan
miR-7161-5p	ENSG00000173041	7mer-m8	TargetScan
miR-7161-5p	ENSG00000173080	7mer-m8	TargetScan
miR-7161-5p	ENSG00000173083	7mer-m8	TargetScan
miR-7161-5p	ENSG00000173141	7mer-m8	TargetScan
miR-7161-5p	ENSG00000173166	7mer-m8	TargetScan
miR-7161-5p	ENSG00000173200	7mer-m8	TargetScan
miR-7161-5p	ENSG00000173207	7mer-m8	TargetScan
miR-7161-5p	ENSG00000173578	7mer-m8	TargetScan
miR-7161-5p	ENSG00000173597	7mer-m8	TargetScan
miR-7161-5p	ENSG00000173744	7mer-m8	TargetScan
miR-7161-5p	ENSG00000173875	7mer-m8	TargetScan
miR-7161-5p	ENSG00000173947	7mer-m8	TargetScan
miR-7161-5p	ENSG00000174032	7mer-m8	TargetScan
miR-7161-5p	ENSG00000174145	7mer-m8	TargetScan
miR-7161-5p	ENSG00000174243	7mer-m8	TargetScan
miR-7161-5p	ENSG00000174579	7mer-m8	TargetScan
miR-7161-5p	ENSG00000174669	7mer-m8	TargetScan
miR-7161-5p	ENSG00000174738	7mer-m8	TargetScan
miR-7161-5p	ENSG00000174749	7mer-m8	TargetScan
miR-7161-5p	ENSG00000175066	7mer-m8	TargetScan
miR-7161-5p	ENSG00000175104	7mer-m8	TargetScan
miR-7161-5p	ENSG00000175264	7mer-m8	TargetScan
miR-7161-5p	ENSG00000175387	7mer-m8	TargetScan
miR-7161-5p	ENSG00000175414	7mer-m8	TargetScan
miR-7161-5p	ENSG00000175449	7mer-m8	TargetScan
miR-7161-5p	ENSG00000175548	7mer-m8	TargetScan
miR-7161-5p	ENSG00000175745	7mer-m8	TargetScan

miR-7161-5p	ENSG00000175874	7mer-m8	TargetScan
miR-7161-5p	ENSG00000176142	7mer-m8	TargetScan
miR-7161-5p	ENSG00000176399	7mer-m8	TargetScan
miR-7161-5p	ENSG00000176853	7mer-m8	TargetScan
miR-7161-5p	ENSG00000176890	7mer-m8	TargetScan
miR-7161-5p	ENSG00000177034	7mer-m8	TargetScan
miR-7161-5p	ENSG00000177283	7mer-m8	TargetScan
miR-7161-5p	ENSG00000177485	7mer-m8	TargetScan
miR-7161-5p	ENSG00000177613	7mer-m8	TargetScan
miR-7161-5p	ENSG00000177889	7mer-m8	TargetScan
miR-7161-5p	ENSG00000178217	7mer-m8	TargetScan
miR-7161-5p	ENSG00000178951	7mer-m8	TargetScan
miR-7161-5p	ENSG00000179119	7mer-m8	TargetScan
miR-7161-5p	ENSG00000179299	7mer-m8	TargetScan
miR-7161-5p	ENSG00000179918	7mer-m8	TargetScan
miR-7161-5p	ENSG00000180440	7mer-m8	TargetScan
miR-7161-5p	ENSG00000180626	7mer-m8	TargetScan
miR-7161-5p	ENSG00000180917	7mer-m8	TargetScan
miR-7161-5p	ENSG00000180998	7mer-m8	TargetScan
miR-7161-5p	ENSG00000181192	7mer-m8	TargetScan
miR-7161-5p	ENSG00000181195	7mer-m8	TargetScan
miR-7161-5p	ENSG00000181220	7mer-m8	TargetScan
miR-7161-5p	ENSG00000181449	7mer-m8	TargetScan
miR-7161-5p	ENSG00000181467	7mer-m8	TargetScan
miR-7161-5p	ENSG00000181896	7mer-m8	TargetScan
miR-7161-5p	ENSG00000182077	7mer-m8	TargetScan
miR-7161-5p	ENSG00000182158	7mer-m8	TargetScan
miR-7161-5p	ENSG00000182247	7mer-m8	TargetScan
miR-7161-5p	ENSG00000182263	7mer-m8	TargetScan
miR-7161-5p	ENSG00000182307	7mer-m8	TargetScan
miR-7161-5p	ENSG00000182348	7mer-m8	TargetScan

miR-7161-5p	ENSG00000182389	7mer-m8	TargetScan
miR-7161-5p	ENSG00000182732	7mer-m8	TargetScan
miR-7161-5p	ENSG00000182810	7mer-m8	TargetScan
miR-7161-5p	ENSG00000182923	7mer-m8	TargetScan
miR-7161-5p	ENSG00000182957	7mer-m8	TargetScan
miR-7161-5p	ENSG00000183067	7mer-m8	TargetScan
miR-7161-5p	ENSG00000183098	7mer-m8	TargetScan
miR-7161-5p	ENSG00000183134	7mer-m8	TargetScan
miR-7161-5p	ENSG00000183309	7mer-m8	TargetScan
miR-7161-5p	ENSG00000183628	7mer-m8	TargetScan
miR-7161-5p	ENSG00000183726	7mer-m8	TargetScan
miR-7161-5p	ENSG00000183801	7mer-m8	TargetScan
miR-7161-5p	ENSG00000184005	7mer-m8	TargetScan
miR-7161-5p	ENSG00000184007	7mer-m8	TargetScan
miR-7161-5p	ENSG00000184258	7mer-m8	TargetScan
miR-7161-5p	ENSG00000184349	7mer-m8	TargetScan
miR-7161-5p	ENSG00000184374	7mer-m8	TargetScan
miR-7161-5p	ENSG00000184384	7mer-m8	TargetScan
miR-7161-5p	ENSG00000184402	7mer-m8	TargetScan
miR-7161-5p	ENSG00000184408	7mer-m8	TargetScan
miR-7161-5p	ENSG00000184486	7mer-m8	TargetScan
miR-7161-5p	ENSG00000184845	7mer-m8	TargetScan
miR-7161-5p	ENSG00000184916	7mer-m8	TargetScan
miR-7161-5p	ENSG00000185104	7mer-m8	TargetScan
miR-7161-5p	ENSG00000185238	7mer-m8	TargetScan
miR-7161-5p	ENSG00000185414	7mer-m8	TargetScan
miR-7161-5p	ENSG00000185515	7mer-m8	TargetScan
miR-7161-5p	ENSG00000185591	7mer-m8	TargetScan
miR-7161-5p	ENSG00000185753	7mer-m8	TargetScan
miR-7161-5p	ENSG00000185920	7mer-m8	TargetScan
miR-7161-5p	ENSG00000186063	7mer-m8	TargetScan

miR-7161-5p	ENSG00000186265	7mer-m8	TargetScan
miR-7161-5p	ENSG00000186479	7mer-m8	TargetScan
miR-7161-5p	ENSG00000186814	7mer-m8	TargetScan
miR-7161-5p	ENSG00000187098	7mer-m8	TargetScan
miR-7161-5p	ENSG00000187109	7mer-m8	TargetScan
miR-7161-5p	ENSG00000187140	7mer-m8	TargetScan
miR-7161-5p	ENSG00000187191	7mer-m8	TargetScan
miR-7161-5p	ENSG00000187231	7mer-m8	TargetScan
miR-7161-5p	ENSG00000187257	7mer-m8	TargetScan
miR-7161-5p	ENSG00000187522	7mer-m8	TargetScan
miR-7161-5p	ENSG00000187605	7mer-m8	TargetScan
miR-7161-5p	ENSG00000187742	7mer-m8	TargetScan
miR-7161-5p	ENSG00000187792	7mer-m8	TargetScan
miR-7161-5p	ENSG00000187866	7mer-m8	TargetScan
miR-7161-5p	ENSG00000187889	7mer-m8	TargetScan
miR-7161-5p	ENSG00000188120	7mer-m8	TargetScan
miR-7161-5p	ENSG00000188133	7mer-m8	TargetScan
miR-7161-5p	ENSG00000188167	7mer-m8	TargetScan
miR-7161-5p	ENSG00000188321	7mer-m8	TargetScan
miR-7161-5p	ENSG00000188522	7mer-m8	TargetScan
miR-7161-5p	ENSG00000188612	7mer-m8	TargetScan
miR-7161-5p	ENSG00000188647	7mer-m8	TargetScan
miR-7161-5p	ENSG00000188994	7mer-m8	TargetScan
miR-7161-5p	ENSG00000189195	7mer-m8	TargetScan
miR-7161-5p	ENSG00000189350	7mer-m8	TargetScan
miR-7161-5p	ENSG00000196090	7mer-m8	TargetScan
miR-7161-5p	ENSG00000196209	7mer-m8	TargetScan
miR-7161-5p	ENSG00000196458	7mer-m8	TargetScan
miR-7161-5p	ENSG00000196466	7mer-m8	TargetScan
miR-7161-5p	ENSG00000196505	7mer-m8	TargetScan
miR-7161-5p	ENSG00000196659	7mer-m8	TargetScan

miR-7161-5p	ENSG00000196678	7mer-m8	TargetScan
miR-7161-5p	ENSG00000196812	7mer-m8	TargetScan
miR-7161-5p	ENSG00000196937	7mer-m8	TargetScan
miR-7161-5p	ENSG00000196946	7mer-m8	TargetScan
miR-7161-5p	ENSG00000197081	7mer-m8	TargetScan
miR-7161-5p	ENSG00000197121	7mer-m8	TargetScan
miR-7161-5p	ENSG00000197142	7mer-m8	TargetScan
miR-7161-5p	ENSG00000197147	7mer-m8	TargetScan
miR-7161-5p	ENSG00000197472	7mer-m8	TargetScan
miR-7161-5p	ENSG00000197535	7mer-m8	TargetScan
miR-7161-5p	ENSG00000197557	7mer-m8	TargetScan
miR-7161-5p	ENSG00000197566	7mer-m8	TargetScan
miR-7161-5p	ENSG00000197579	7mer-m8	TargetScan
miR-7161-5p	ENSG00000197651	7mer-m8	TargetScan
miR-7161-5p	ENSG00000197713	7mer-m8	TargetScan
miR-7161-5p	ENSG00000197779	7mer-m8	TargetScan
miR-7161-5p	ENSG00000197930	7mer-m8	TargetScan
miR-7161-5p	ENSG00000198046	7mer-m8	TargetScan
miR-7161-5p	ENSG00000198087	7mer-m8	TargetScan
miR-7161-5p	ENSG00000198157	7mer-m8	TargetScan
miR-7161-5p	ENSG00000198408	7mer-m8	TargetScan
miR-7161-5p	ENSG00000198464	7mer-m8	TargetScan
miR-7161-5p	ENSG00000198612	7mer-m8	TargetScan
miR-7161-5p	ENSG00000198668	7mer-m8	TargetScan
miR-7161-5p	ENSG00000198739	7mer-m8	TargetScan
miR-7161-5p	ENSG00000198771	7mer-m8	TargetScan
miR-7161-5p	ENSG00000198791	7mer-m8	TargetScan
miR-7161-5p	ENSG00000198799	7mer-m8	TargetScan
miR-7161-5p	ENSG00000198812	7mer-m8	TargetScan
miR-7161-5p	ENSG00000198824	7mer-m8	TargetScan
miR-7161-5p	ENSG00000198865	7mer-m8	TargetScan

miR-7161-5p	ENSG00000198873	7mer-m8	TargetScan
miR-7161-5p	ENSG00000198898	7mer-m8	TargetScan
miR-7161-5p	ENSG00000198929	7mer-m8	TargetScan
miR-7161-5p	ENSG00000203485	7mer-m8	TargetScan
miR-7161-5p	ENSG00000204033	7mer-m8	TargetScan
miR-7161-5p	ENSG00000204279	7mer-m8	TargetScan
miR-7161-5p	ENSG00000204688	7mer-m8	TargetScan
miR-7161-5p	ENSG00000204977	7mer-m8	TargetScan
miR-7161-5p	ENSG00000205758	7mer-m8	TargetScan
miR-7161-5p	ENSG00000205835	7mer-m8	TargetScan
miR-7161-5p	ENSG00000205916	7mer-m8	TargetScan
miR-7161-5p	ENSG00000205944	7mer-m8	TargetScan
miR-7161-5p	ENSG00000206432	7mer-m8	TargetScan
miR-7161-5p	ENSG00000206538	7mer-m8	TargetScan
miR-7161-5p	ENSG00000211455	7mer-m8	TargetScan
miR-7161-5p	ENSG00000211456	7mer-m8	TargetScan
miR-7161-5p	ENSG00000213625	7mer-m8	TargetScan
miR-7161-5p	ENSG00000213762	7mer-m8	TargetScan
miR-7161-5p	ENSG00000213967	7mer-m8	TargetScan
miR-7161-5p	ENSG00000213973	7mer-m8	TargetScan
miR-7161-5p	ENSG00000214216	7mer-m8	TargetScan
miR-7161-5p	ENSG00000214827	7mer-m8	TargetScan
miR-7161-5p	ENSG00000215301	7mer-m8	TargetScan
miR-7161-5p	ENSG00000235376	7mer-m8	TargetScan
miR-7161-5p	ENSG00000237353	7mer-m8	TargetScan
miR-7161-5p	ENSG00000239900	7mer-m8	TargetScan
miR-7161-5p	ENSG00000240654	7mer-m8	TargetScan
miR-7161-5p	ENSG00000241399	7mer-m8	TargetScan
miR-7161-5p	ENSG00000241489	7mer-m8	TargetScan
miR-7161-5p	ENSG00000242110	7mer-m8	TargetScan
miR-7161-5p	ENSG00000242220	7mer-m8	TargetScan

miR-7161-5p	ENSG00000242689	7mer-m8	TargetScan
miR-7161-5p	ENSG00000244754	7mer-m8	TargetScan
miR-7161-5p	ENSG00000248672	7mer-m8	TargetScan
miR-7161-5p	ENSG00000249884	7mer-m8	TargetScan
miR-7161-5p	ENSG00000255112	7mer-m8	TargetScan
miR-7161-5p	ENSG00000255529	7mer-m8	TargetScan
miR-7161-5p	ENSG00000255561	7mer-m8	TargetScan
miR-7161-5p	ENSG00000256771	7mer-m8	TargetScan
miR-7161-5p	ENSG00000257127	7mer-m8	TargetScan
miR-7161-5p	ENSG00000259207	7mer-m8	TargetScan
miR-7161-5p	ENSG00000259458	7mer-m8	TargetScan
miR-7161-5p	ENSG00000259495	7mer-m8	TargetScan
miR-7161-5p	ENSG00000260230	7mer-m8	TargetScan
miR-7161-5p	ENSG00000267041	7mer-m8	TargetScan
miR-7161-5p	ENSG00000267618	7mer-m8	TargetScan
miR-7161-5p	ENSG00000273079	7mer-m8	TargetScan
miR-7161-5p	ENSG00000273274	7mer-m8	TargetScan
miR-572	ENSG00000011485	7mer-m8	TargetScan
miR-572	ENSG00000039068	7mer-m8	TargetScan
miR-572	ENSG00000066739	7mer-m8	TargetScan
miR-572	ENSG00000070269	7mer-m8	TargetScan
miR-572	ENSG00000078061	7mer-m8	TargetScan
miR-572	ENSG00000078687	7mer-m8	TargetScan
miR-572	ENSG00000086232	7mer-m8	TargetScan
miR-572	ENSG00000089250	7mer-m8	TargetScan
miR-572	ENSG00000099769	7mer-m8	TargetScan
miR-572	ENSG00000100014	7mer-m8	TargetScan
miR-572	ENSG00000100364	7mer-m8	TargetScan
miR-572	ENSG00000100804	7mer-m8	TargetScan
miR-572	ENSG00000101104	7mer-m8	TargetScan
miR-572	ENSG00000102935	7mer-m8	TargetScan

miR-572	ENSG00000103534	7mer-m8	TargetScan
miR-572	ENSG00000114933	7mer-m8	TargetScan
miR-572	ENSG00000116731	7mer-m8	TargetScan
miR-572	ENSG00000125046	7mer-m8	TargetScan
miR-572	ENSG00000129473	7mer-m8	TargetScan
miR-572	ENSG00000130669	7mer-m8	TargetScan
miR-572	ENSG00000131153	7mer-m8	TargetScan
miR-572	ENSG00000132792	7mer-m8	TargetScan
miR-572	ENSG00000132932	7mer-m8	TargetScan
miR-572	ENSG00000136425	7mer-m8	TargetScan
miR-572	ENSG00000137491	7mer-m8	TargetScan
miR-572	ENSG00000138622	7mer-m8	TargetScan
miR-572	ENSG00000140527	7mer-m8	TargetScan
miR-572	ENSG00000140937	7mer-m8	TargetScan
miR-572	ENSG00000142599	7mer-m8	TargetScan
miR-572	ENSG00000143032	7mer-m8	TargetScan
miR-572	ENSG00000145012	7mer-m8	TargetScan
miR-572	ENSG00000145016	7mer-m8	TargetScan
miR-572	ENSG00000145734	7mer-m8	TargetScan
miR-572	ENSG00000149294	7mer-m8	TargetScan
miR-572	ENSG00000149403	7mer-m8	TargetScan
miR-572	ENSG00000149930	7mer-m8	TargetScan
miR-572	ENSG00000152822	7mer-m8	TargetScan
miR-572	ENSG00000154227	7mer-m8	TargetScan
miR-572	ENSG00000158859	7mer-m8	TargetScan
miR-572	ENSG00000158985	7mer-m8	TargetScan
miR-572	ENSG00000160683	7mer-m8	TargetScan
miR-572	ENSG00000160688	7mer-m8	TargetScan
miR-572	ENSG00000160862	7mer-m8	TargetScan
miR-572	ENSG00000160953	7mer-m8	TargetScan
miR-572	ENSG00000161526	7mer-m8	TargetScan

miR-572	ENSG00000162849	7mer-m8	TargetScan
miR-572	ENSG00000164402	7mer-m8	TargetScan
miR-572	ENSG00000165169	7mer-m8	TargetScan
miR-572	ENSG00000168439	7mer-m8	TargetScan
miR-572	ENSG00000168490	7mer-m8	TargetScan
miR-572	ENSG00000169223	7mer-m8	TargetScan
miR-572	ENSG00000169992	7mer-m8	TargetScan
miR-572	ENSG00000170525	7mer-m8	TargetScan
miR-572	ENSG00000172578	7mer-m8	TargetScan
miR-572	ENSG00000172889	7mer-m8	TargetScan
miR-572	ENSG00000173210	7mer-m8	TargetScan
miR-572	ENSG00000173517	7mer-m8	TargetScan
miR-572	ENSG00000175727	7mer-m8	TargetScan
miR-572	ENSG00000176658	7mer-m8	TargetScan
miR-572	ENSG00000176853	7mer-m8	TargetScan
miR-572	ENSG00000177300	7mer-m8	TargetScan
miR-572	ENSG00000182473	7mer-m8	TargetScan
miR-572	ENSG00000182551	7mer-m8	TargetScan
miR-572	ENSG00000182704	7mer-m8	TargetScan
miR-572	ENSG00000183153	7mer-m8	TargetScan
miR-572	ENSG00000183762	7mer-m8	TargetScan
miR-572	ENSG00000183826	7mer-m8	TargetScan
miR-572	ENSG00000184471	7mer-m8	TargetScan
miR-572	ENSG00000184992	7mer-m8	TargetScan
miR-572	ENSG00000186575	7mer-m8	TargetScan
miR-572	ENSG00000186591	7mer-m8	TargetScan
miR-572	ENSG00000187912	7mer-m8	TargetScan
miR-572	ENSG00000196182	7mer-m8	TargetScan
miR-572	ENSG00000196557	7mer-m8	TargetScan
miR-572	ENSG00000197312	7mer-m8	TargetScan
miR-572	ENSG00000198198	7mer-m8	TargetScan

miR-572	ENSG00000204283	7mer-m8	TargetScan
miR-572	ENSG00000205176	7mer-m8	TargetScan
miR-572	ENSG00000213614	7mer-m8	TargetScan
miR-572	ENSG00000220205	7mer-m8	TargetScan
miR-572	ENSG00000254772	7mer-m8	TargetScan
miR-572	ENSG00000257103	7mer-m8	TargetScan
miR-572	ENSG00000258436	7mer-m8	TargetScan
miR-572	ENSG00000266173	7mer-m8	TargetScan
miR-622	ENSG00000001167	7mer-m8	TargetScan
miR-622	ENSG00000001460	7mer-m8	TargetScan
miR-622	ENSG00000002933	7mer-m8	TargetScan
miR-622	ENSG00000004468	7mer-m8	TargetScan
miR-622	ENSG00000005249	7mer-m8	TargetScan
miR-622	ENSG00000005884	7mer-m8	TargetScan
miR-622	ENSG00000005981	7mer-m8	TargetScan
miR-622	ENSG00000006194	7mer-m8	TargetScan
miR-622	ENSG00000006377	7mer-m8	TargetScan
miR-622	ENSG00000006555	7mer-m8	TargetScan
miR-622	ENSG00000007944	7mer-m8	TargetScan
miR-622	ENSG00000008256	7mer-m8	TargetScan
miR-622	ENSG00000009844	7mer-m8	TargetScan
miR-622	ENSG00000010671	7mer-m8	TargetScan
miR-622	ENSG00000011007	7mer-m8	TargetScan
miR-622	ENSG00000011198	7mer-m8	TargetScan
miR-622	ENSG00000013016	7mer-m8	TargetScan
miR-622	ENSG00000013275	7mer-m8	TargetScan
miR-622	ENSG00000014824	7mer-m8	TargetScan
miR-622	ENSG00000015153	7mer-m8	TargetScan
miR-622	ENSG00000020922	7mer-m8	TargetScan
miR-622	ENSG00000021776	7mer-m8	TargetScan
miR-622	ENSG00000023318	7mer-m8	TargetScan

miR-622	ENSG00000025434	7mer-m8	TargetScan
miR-622	ENSG00000025772	7mer-m8	TargetScan
miR-622	ENSG00000026652	7mer-m8	TargetScan
miR-622	ENSG00000029639	7mer-m8	TargetScan
miR-622	ENSG00000032219	7mer-m8	TargetScan
miR-622	ENSG00000033627	7mer-m8	TargetScan
miR-622	ENSG00000036828	7mer-m8	TargetScan
miR-622	ENSG00000042781	7mer-m8	TargetScan
miR-622	ENSG00000043093	7mer-m8	TargetScan
miR-622	ENSG00000043514	7mer-m8	TargetScan
miR-622	ENSG00000044115	7mer-m8	TargetScan
miR-622	ENSG00000048649	7mer-m8	TargetScan
miR-622	ENSG00000050748	7mer-m8	TargetScan
miR-622	ENSG00000052344	7mer-m8	TargetScan
miR-622	ENSG00000052841	7mer-m8	TargetScan
miR-622	ENSG00000054965	7mer-m8	TargetScan
miR-622	ENSG00000056277	7mer-m8	TargetScan
miR-622	ENSG00000056558	7mer-m8	TargetScan
miR-622	ENSG00000057935	7mer-m8	TargetScan
miR-622	ENSG00000058729	7mer-m8	TargetScan
miR-622	ENSG00000058866	7mer-m8	TargetScan
miR-622	ENSG00000062716	7mer-m8	TargetScan
miR-622	ENSG00000064199	7mer-m8	TargetScan
miR-622	ENSG00000064999	7mer-m8	TargetScan
miR-622	ENSG00000065427	7mer-m8	TargetScan
miR-622	ENSG00000066294	7mer-m8	TargetScan
miR-622	ENSG00000069431	7mer-m8	TargetScan
miR-622	ENSG00000069812	7mer-m8	TargetScan
miR-622	ENSG00000070476	7mer-m8	TargetScan
miR-622	ENSG00000070614	7mer-m8	TargetScan
miR-622	ENSG00000070778	7mer-m8	TargetScan

miR-622	ENSG00000070961	7mer-m8	TargetScan
miR-622	ENSG00000071242	7mer-m8	TargetScan
miR-622	ENSG00000071575	7mer-m8	TargetScan
miR-622	ENSG00000072062	7mer-m8	TargetScan
miR-622	ENSG00000072135	7mer-m8	TargetScan
miR-622	ENSG00000074370	7mer-m8	TargetScan
miR-622	ENSG00000074590	7mer-m8	TargetScan
miR-622	ENSG00000074695	7mer-m8	TargetScan
miR-622	ENSG00000075884	7mer-m8	TargetScan
miR-622	ENSG00000076554	7mer-m8	TargetScan
miR-622	ENSG00000076650	7mer-m8	TargetScan
miR-622	ENSG00000077235	7mer-m8	TargetScan
miR-622	ENSG00000077549	7mer-m8	TargetScan
miR-622	ENSG00000077684	7mer-m8	TargetScan
miR-622	ENSG00000078053	7mer-m8	TargetScan
miR-622	ENSG00000078140	7mer-m8	TargetScan
miR-622	ENSG00000078399	7mer-m8	TargetScan
miR-622	ENSG00000078618	7mer-m8	TargetScan
miR-622	ENSG00000078900	7mer-m8	TargetScan
miR-622	ENSG00000079950	7mer-m8	TargetScan
miR-622	ENSG00000080224	7mer-m8	TargetScan
miR-622	ENSG00000081189	7mer-m8	TargetScan
miR-622	ENSG00000081760	7mer-m8	TargetScan
miR-622	ENSG00000082701	7mer-m8	TargetScan
miR-622	ENSG00000082781	7mer-m8	TargetScan
miR-622	ENSG00000083520	7mer-m8	TargetScan
miR-622	ENSG00000083812	7mer-m8	TargetScan
miR-622	ENSG00000083844	7mer-m8	TargetScan
miR-622	ENSG00000084733	7mer-m8	TargetScan
miR-622	ENSG00000085721	7mer-m8	TargetScan
miR-622	ENSG00000086189	7mer-m8	TargetScan

miR-622	ENSG00000086232	7mer-m8	TargetScan
miR-622	ENSG00000086712	7mer-m8	TargetScan
miR-622	ENSG00000089248	7mer-m8	TargetScan
miR-622	ENSG00000090686	7mer-m8	TargetScan
miR-622	ENSG00000091879	7mer-m8	TargetScan
miR-622	ENSG00000091972	7mer-m8	TargetScan
miR-622	ENSG00000092853	7mer-m8	TargetScan
miR-622	ENSG00000093000	7mer-m8	TargetScan
miR-622	ENSG00000093010	7mer-m8	TargetScan
miR-622	ENSG00000095637	7mer-m8	TargetScan
miR-622	ENSG00000096872	7mer-m8	TargetScan
miR-622	ENSG00000097007	7mer-m8	TargetScan
miR-622	ENSG00000099999	7mer-m8	TargetScan
miR-622	ENSG00000100027	7mer-m8	TargetScan
miR-622	ENSG00000100084	7mer-m8	TargetScan
miR-622	ENSG00000100345	7mer-m8	TargetScan
miR-622	ENSG00000100351	7mer-m8	TargetScan
miR-622	ENSG00000100368	7mer-m8	TargetScan
miR-622	ENSG00000100376	7mer-m8	TargetScan
miR-622	ENSG00000100441	7mer-m8	TargetScan
miR-622	ENSG00000100568	7mer-m8	TargetScan
miR-622	ENSG00000101193	7mer-m8	TargetScan
miR-622	ENSG00000101266	7mer-m8	TargetScan
miR-622	ENSG00000101280	7mer-m8	TargetScan
miR-622	ENSG00000101290	7mer-m8	TargetScan
miR-622	ENSG00000101307	7mer-m8	TargetScan
miR-622	ENSG00000101412	7mer-m8	TargetScan
miR-622	ENSG00000101445	7mer-m8	TargetScan
miR-622	ENSG00000101544	7mer-m8	TargetScan
miR-622	ENSG00000101596	7mer-m8	TargetScan
miR-622	ENSG00000101695	7mer-m8	TargetScan

miR-622	ENSG00000101945	7mer-m8	TargetScan
miR-622	ENSG00000102043	7mer-m8	TargetScan
miR-622	ENSG00000102317	7mer-m8	TargetScan
miR-622	ENSG00000102531	7mer-m8	TargetScan
miR-622	ENSG00000102780	7mer-m8	TargetScan
miR-622	ENSG00000102870	7mer-m8	TargetScan
miR-622	ENSG00000102908	7mer-m8	TargetScan
miR-622	ENSG00000103005	7mer-m8	TargetScan
miR-622	ENSG00000103021	7mer-m8	TargetScan
miR-622	ENSG00000103187	7mer-m8	TargetScan
miR-622	ENSG00000103351	7mer-m8	TargetScan
miR-622	ENSG00000103375	7mer-m8	TargetScan
miR-622	ENSG00000103404	7mer-m8	TargetScan
miR-622	ENSG00000103449	7mer-m8	TargetScan
miR-622	ENSG00000103507	7mer-m8	TargetScan
miR-622	ENSG00000103855	7mer-m8	TargetScan
miR-622	ENSG00000103995	7mer-m8	TargetScan
miR-622	ENSG00000104043	7mer-m8	TargetScan
miR-622	ENSG00000104067	7mer-m8	TargetScan
miR-622	ENSG00000104219	7mer-m8	TargetScan
miR-622	ENSG00000104313	7mer-m8	TargetScan
miR-622	ENSG00000104332	7mer-m8	TargetScan
miR-622	ENSG00000104361	7mer-m8	TargetScan
miR-622	ENSG00000104447	7mer-m8	TargetScan
miR-622	ENSG00000104824	7mer-m8	TargetScan
miR-622	ENSG00000104885	7mer-m8	TargetScan
miR-622	ENSG00000104886	7mer-m8	TargetScan
miR-622	ENSG00000104888	7mer-m8	TargetScan
miR-622	ENSG00000104967	7mer-m8	TargetScan
miR-622	ENSG00000105197	7mer-m8	TargetScan
miR-622	ENSG00000105429	7mer-m8	TargetScan

miR-622	ENSG00000105865	7mer-m8	TargetScan
miR-622	ENSG00000105866	7mer-m8	TargetScan
miR-622	ENSG00000105926	7mer-m8	TargetScan
miR-622	ENSG00000106261	7mer-m8	TargetScan
miR-622	ENSG00000106692	7mer-m8	TargetScan
miR-622	ENSG00000106723	7mer-m8	TargetScan
miR-622	ENSG00000106868	7mer-m8	TargetScan
miR-622	ENSG00000107130	7mer-m8	TargetScan
miR-622	ENSG00000107223	7mer-m8	TargetScan
miR-622	ENSG00000107249	7mer-m8	TargetScan
miR-622	ENSG00000107372	7mer-m8	TargetScan
miR-622	ENSG00000107611	7mer-m8	TargetScan
miR-622	ENSG00000107614	7mer-m8	TargetScan
miR-622	ENSG00000107771	7mer-m8	TargetScan
miR-622	ENSG00000107897	7mer-m8	TargetScan
miR-622	ENSG00000107960	7mer-m8	TargetScan
miR-622	ENSG00000108219	7mer-m8	TargetScan
miR-622	ENSG00000108423	7mer-m8	TargetScan
miR-622	ENSG00000108528	7mer-m8	TargetScan
miR-622	ENSG00000108559	7mer-m8	TargetScan
miR-622	ENSG00000108591	7mer-m8	TargetScan
miR-622	ENSG00000108799	7mer-m8	TargetScan
miR-622	ENSG00000108854	7mer-m8	TargetScan
miR-622	ENSG00000109046	7mer-m8	TargetScan
miR-622	ENSG00000109065	7mer-m8	TargetScan
miR-622	ENSG00000109667	7mer-m8	TargetScan
miR-622	ENSG00000109670	7mer-m8	TargetScan
miR-622	ENSG00000109685	7mer-m8	TargetScan
miR-622	ENSG00000110042	7mer-m8	TargetScan
miR-622	ENSG00000110104	7mer-m8	TargetScan
miR-622	ENSG00000110169	7mer-m8	TargetScan

miR-622	ENSG00000110321	7mer-m8	TargetScan
miR-622	ENSG00000110536	7mer-m8	TargetScan
miR-622	ENSG00000111110	7mer-m8	TargetScan
miR-622	ENSG00000111300	7mer-m8	TargetScan
miR-622	ENSG00000111361	7mer-m8	TargetScan
miR-622	ENSG00000111696	7mer-m8	TargetScan
miR-622	ENSG00000111817	7mer-m8	TargetScan
miR-622	ENSG00000111843	7mer-m8	TargetScan
miR-622	ENSG00000112033	7mer-m8	TargetScan
miR-622	ENSG00000112062	7mer-m8	TargetScan
miR-622	ENSG00000112081	7mer-m8	TargetScan
miR-622	ENSG00000112130	7mer-m8	TargetScan
miR-622	ENSG00000112137	7mer-m8	TargetScan
miR-622	ENSG00000112208	7mer-m8	TargetScan
miR-622	ENSG00000112232	7mer-m8	TargetScan
miR-622	ENSG00000112343	7mer-m8	TargetScan
miR-622	ENSG00000112419	7mer-m8	TargetScan
miR-622	ENSG00000112624	7mer-m8	TargetScan
miR-622	ENSG00000112769	7mer-m8	TargetScan
miR-622	ENSG00000113048	7mer-m8	TargetScan
miR-622	ENSG00000113140	7mer-m8	TargetScan
miR-622	ENSG00000113163	7mer-m8	TargetScan
miR-622	ENSG00000113303	7mer-m8	TargetScan
miR-622	ENSG00000113441	7mer-m8	TargetScan
miR-622	ENSG00000113494	7mer-m8	TargetScan
miR-622	ENSG00000113558	7mer-m8	TargetScan
miR-622	ENSG00000114302	7mer-m8	TargetScan
miR-622	ENSG00000114648	7mer-m8	TargetScan
miR-622	ENSG00000114738	7mer-m8	TargetScan
miR-622	ENSG00000114739	7mer-m8	TargetScan
miR-622	ENSG00000114805	7mer-m8	TargetScan

miR-622	ENSG00000115109	7mer-m8	TargetScan
miR-622	ENSG00000115163	7mer-m8	TargetScan
miR-622	ENSG00000115232	7mer-m8	TargetScan
miR-622	ENSG00000115286	7mer-m8	TargetScan
miR-622	ENSG00000115310	7mer-m8	TargetScan
miR-622	ENSG00000115540	7mer-m8	TargetScan
miR-622	ENSG00000115808	7mer-m8	TargetScan
miR-622	ENSG00000115825	7mer-m8	TargetScan
miR-622	ENSG00000115896	7mer-m8	TargetScan
miR-622	ENSG00000115966	7mer-m8	TargetScan
miR-622	ENSG00000116095	7mer-m8	TargetScan
miR-622	ENSG00000116132	7mer-m8	TargetScan
miR-622	ENSG00000116191	7mer-m8	TargetScan
miR-622	ENSG00000116209	7mer-m8	TargetScan
miR-622	ENSG00000116649	7mer-m8	TargetScan
miR-622	ENSG00000116984	7mer-m8	TargetScan
miR-622	ENSG00000117090	7mer-m8	TargetScan
miR-622	ENSG00000117139	7mer-m8	TargetScan
miR-622	ENSG00000117533	7mer-m8	TargetScan
miR-622	ENSG00000118217	7mer-m8	TargetScan
miR-622	ENSG00000118271	7mer-m8	TargetScan
miR-622	ENSG00000118620	7mer-m8	TargetScan
miR-622	ENSG00000118922	7mer-m8	TargetScan
miR-622	ENSG00000119121	7mer-m8	TargetScan
miR-622	ENSG00000119321	7mer-m8	TargetScan
miR-622	ENSG00000119402	7mer-m8	TargetScan
miR-622	ENSG00000119522	7mer-m8	TargetScan
miR-622	ENSG00000119685	7mer-m8	TargetScan
miR-622	ENSG00000119787	7mer-m8	TargetScan
miR-622	ENSG00000119865	7mer-m8	TargetScan
miR-622	ENSG00000119953	7mer-m8	TargetScan

miR-622	ENSG00000120662	7mer-m8	TargetScan
miR-622	ENSG00000120709	7mer-m8	TargetScan
miR-622	ENSG00000120942	7mer-m8	TargetScan
miR-622	ENSG00000120963	7mer-m8	TargetScan
miR-622	ENSG00000121060	7mer-m8	TargetScan
miR-622	ENSG00000121207	7mer-m8	TargetScan
miR-622	ENSG00000121361	7mer-m8	TargetScan
miR-622	ENSG00000121579	7mer-m8	TargetScan
miR-622	ENSG00000121957	7mer-m8	TargetScan
miR-622	ENSG00000122550	7mer-m8	TargetScan
miR-622	ENSG00000122574	7mer-m8	TargetScan
miR-622	ENSG00000123119	7mer-m8	TargetScan
miR-622	ENSG00000123407	7mer-m8	TargetScan
miR-622	ENSG00000123561	7mer-m8	TargetScan
miR-622	ENSG00000123643	7mer-m8	TargetScan
miR-622	ENSG00000124194	7mer-m8	TargetScan
miR-622	ENSG00000124243	7mer-m8	TargetScan
miR-622	ENSG00000124356	7mer-m8	TargetScan
miR-622	ENSG00000124486	7mer-m8	TargetScan
miR-622	ENSG00000124496	7mer-m8	TargetScan
miR-622	ENSG00000124523	7mer-m8	TargetScan
miR-622	ENSG00000124783	7mer-m8	TargetScan
miR-622	ENSG00000124788	7mer-m8	TargetScan
miR-622	ENSG00000125450	7mer-m8	TargetScan
miR-622	ENSG00000125629	7mer-m8	TargetScan
miR-622	ENSG00000125870	7mer-m8	TargetScan
miR-622	ENSG00000125885	7mer-m8	TargetScan
miR-622	ENSG00000125970	7mer-m8	TargetScan
miR-622	ENSG00000126070	7mer-m8	TargetScan
miR-622	ENSG00000126106	7mer-m8	TargetScan
miR-622	ENSG00000126214	7mer-m8	TargetScan

miR-622	ENSG00000126215	7mer-m8	TargetScan
miR-622	ENSG00000127334	7mer-m8	TargetScan
miR-622	ENSG00000127399	7mer-m8	TargetScan
miR-622	ENSG00000127418	7mer-m8	TargetScan
miR-622	ENSG00000127863	7mer-m8	TargetScan
miR-622	ENSG00000128050	7mer-m8	TargetScan
miR-622	ENSG00000128165	7mer-m8	TargetScan
miR-622	ENSG00000128383	7mer-m8	TargetScan
miR-622	ENSG00000128652	7mer-m8	TargetScan
miR-622	ENSG00000128655	7mer-m8	TargetScan
miR-622	ENSG00000129007	7mer-m8	TargetScan
miR-622	ENSG00000129173	7mer-m8	TargetScan
miR-622	ENSG00000130052	7mer-m8	TargetScan
miR-622	ENSG00000130054	7mer-m8	TargetScan
miR-622	ENSG00000130224	7mer-m8	TargetScan
miR-622	ENSG00000130517	7mer-m8	TargetScan
miR-622	ENSG00000130584	7mer-m8	TargetScan
miR-622	ENSG00000130640	7mer-m8	TargetScan
miR-622	ENSG00000130669	7mer-m8	TargetScan
miR-622	ENSG00000130723	7mer-m8	TargetScan
miR-622	ENSG00000130766	7mer-m8	TargetScan
miR-622	ENSG00000131149	7mer-m8	TargetScan
miR-622	ENSG00000131374	7mer-m8	TargetScan
miR-622	ENSG00000131386	7mer-m8	TargetScan
miR-622	ENSG00000131873	7mer-m8	TargetScan
miR-622	ENSG00000132394	7mer-m8	TargetScan
miR-622	ENSG00000132405	7mer-m8	TargetScan
miR-622	ENSG00000132964	7mer-m8	TargetScan
miR-622	ENSG00000133193	7mer-m8	TargetScan
miR-622	ENSG00000134046	7mer-m8	TargetScan
miR-622	ENSG00000134201	7mer-m8	TargetScan

miR-622	ENSG00000134250	7mer-m8	TargetScan
miR-622	ENSG00000134278	7mer-m8	TargetScan
miR-622	ENSG00000134294	7mer-m8	TargetScan
miR-622	ENSG00000134339	7mer-m8	TargetScan
miR-622	ENSG00000134775	7mer-m8	TargetScan
miR-622	ENSG00000135018	7mer-m8	TargetScan
miR-622	ENSG00000135362	7mer-m8	TargetScan
miR-622	ENSG00000135363	7mer-m8	TargetScan
miR-622	ENSG00000135426	7mer-m8	TargetScan
miR-622	ENSG00000135454	7mer-m8	TargetScan
miR-622	ENSG00000135473	7mer-m8	TargetScan
miR-622	ENSG00000135482	7mer-m8	TargetScan
miR-622	ENSG00000135604	7mer-m8	TargetScan
miR-622	ENSG00000135655	7mer-m8	TargetScan
miR-622	ENSG00000135823	7mer-m8	TargetScan
miR-622	ENSG00000135835	7mer-m8	TargetScan
miR-622	ENSG00000136014	7mer-m8	TargetScan
miR-622	ENSG00000136237	7mer-m8	TargetScan
miR-622	ENSG00000136279	7mer-m8	TargetScan
miR-622	ENSG00000136492	7mer-m8	TargetScan
miR-622	ENSG00000136807	7mer-m8	TargetScan
miR-622	ENSG00000136816	7mer-m8	TargetScan
miR-622	ENSG00000136828	7mer-m8	TargetScan
miR-622	ENSG00000136842	7mer-m8	TargetScan
miR-622	ENSG00000136877	7mer-m8	TargetScan
miR-622	ENSG00000136878	7mer-m8	TargetScan
miR-622	ENSG00000137210	7mer-m8	TargetScan
miR-622	ENSG00000137478	7mer-m8	TargetScan
miR-622	ENSG00000137494	7mer-m8	TargetScan
miR-622	ENSG00000137714	7mer-m8	TargetScan
miR-622	ENSG00000137817	7mer-m8	TargetScan

miR-622	ENSG00000137819	7mer-m8	TargetScan
miR-622	ENSG00000137959	7mer-m8	TargetScan
miR-622	ENSG00000138134	7mer-m8	TargetScan
miR-622	ENSG00000138175	7mer-m8	TargetScan
miR-622	ENSG00000138411	7mer-m8	TargetScan
miR-622	ENSG00000138443	7mer-m8	TargetScan
miR-622	ENSG00000138798	7mer-m8	TargetScan
miR-622	ENSG00000138944	7mer-m8	TargetScan
miR-622	ENSG00000139131	7mer-m8	TargetScan
miR-622	ENSG00000139146	7mer-m8	TargetScan
miR-622	ENSG00000139428	7mer-m8	TargetScan
miR-622	ENSG00000139433	7mer-m8	TargetScan
miR-622	ENSG00000140332	7mer-m8	TargetScan
miR-622	ENSG00000140367	7mer-m8	TargetScan
miR-622	ENSG00000140577	7mer-m8	TargetScan
miR-622	ENSG00000140853	7mer-m8	TargetScan
miR-622	ENSG00000140931	7mer-m8	TargetScan
miR-622	ENSG00000140941	7mer-m8	TargetScan
miR-622	ENSG00000140943	7mer-m8	TargetScan
miR-622	ENSG00000141013	7mer-m8	TargetScan
miR-622	ENSG00000141337	7mer-m8	TargetScan
miR-622	ENSG00000141376	7mer-m8	TargetScan
miR-622	ENSG00000141380	7mer-m8	TargetScan
miR-622	ENSG00000141447	7mer-m8	TargetScan
miR-622	ENSG00000141469	7mer-m8	TargetScan
miR-622	ENSG00000141506	7mer-m8	TargetScan
miR-622	ENSG00000141510	7mer-m8	TargetScan
miR-622	ENSG00000141522	7mer-m8	TargetScan
miR-622	ENSG00000141527	7mer-m8	TargetScan
miR-622	ENSG00000141552	7mer-m8	TargetScan
miR-622	ENSG00000141568	7mer-m8	TargetScan

miR-622	ENSG00000142408	7mer-m8	TargetScan
miR-622	ENSG00000142657	7mer-m8	TargetScan
miR-622	ENSG00000143382	7mer-m8	TargetScan
miR-622	ENSG00000143393	7mer-m8	TargetScan
miR-622	ENSG00000143409	7mer-m8	TargetScan
miR-622	ENSG00000143502	7mer-m8	TargetScan
miR-622	ENSG00000143603	7mer-m8	TargetScan
miR-622	ENSG00000143786	7mer-m8	TargetScan
miR-622	ENSG00000143951	7mer-m8	TargetScan
miR-622	ENSG00000143995	7mer-m8	TargetScan
miR-622	ENSG00000144063	7mer-m8	TargetScan
miR-622	ENSG00000144320	7mer-m8	TargetScan
miR-622	ENSG00000144381	7mer-m8	TargetScan
miR-622	ENSG00000144401	7mer-m8	TargetScan
miR-622	ENSG00000144677	7mer-m8	TargetScan
miR-622	ENSG00000144741	7mer-m8	TargetScan
miR-622	ENSG00000144791	7mer-m8	TargetScan
miR-622	ENSG00000144909	7mer-m8	TargetScan
miR-622	ENSG00000145242	7mer-m8	TargetScan
miR-622	ENSG00000145246	7mer-m8	TargetScan
miR-622	ENSG00000145391	7mer-m8	TargetScan
miR-622	ENSG00000145416	7mer-m8	TargetScan
miR-622	ENSG00000145423	7mer-m8	TargetScan
miR-622	ENSG00000145569	7mer-m8	TargetScan
miR-622	ENSG00000145685	7mer-m8	TargetScan
miR-622	ENSG00000145782	7mer-m8	TargetScan
miR-622	ENSG00000145817	7mer-m8	TargetScan
miR-622	ENSG00000145919	7mer-m8	TargetScan
miR-622	ENSG00000145990	7mer-m8	TargetScan
miR-622	ENSG00000146192	7mer-m8	TargetScan
miR-622	ENSG00000146592	7mer-m8	TargetScan

miR-622	ENSG00000146776	7mer-m8	TargetScan
miR-622	ENSG00000146833	7mer-m8	TargetScan
miR-622	ENSG00000147421	7mer-m8	TargetScan
miR-622	ENSG00000147536	7mer-m8	TargetScan
miR-622	ENSG00000147724	7mer-m8	TargetScan
miR-622	ENSG00000148053	7mer-m8	TargetScan
miR-622	ENSG00000148219	7mer-m8	TargetScan
miR-622	ENSG00000148344	7mer-m8	TargetScan
miR-622	ENSG00000148600	7mer-m8	TargetScan
miR-622	ENSG00000148737	7mer-m8	TargetScan
miR-622	ENSG00000148842	7mer-m8	TargetScan
miR-622	ENSG00000149257	7mer-m8	TargetScan
miR-622	ENSG00000149273	7mer-m8	TargetScan
miR-622	ENSG00000149357	7mer-m8	TargetScan
miR-622	ENSG00000149527	7mer-m8	TargetScan
miR-622	ENSG00000149575	7mer-m8	TargetScan
miR-622	ENSG00000149577	7mer-m8	TargetScan
miR-622	ENSG00000149651	7mer-m8	TargetScan
miR-622	ENSG00000149658	7mer-m8	TargetScan
miR-622	ENSG00000149948	7mer-m8	TargetScan
miR-622	ENSG00000150977	7mer-m8	TargetScan
miR-622	ENSG00000151304	7mer-m8	TargetScan
miR-622	ENSG00000151445	7mer-m8	TargetScan
miR-622	ENSG00000151466	7mer-m8	TargetScan
miR-622	ENSG00000151474	7mer-m8	TargetScan
miR-622	ENSG00000151729	7mer-m8	TargetScan
miR-622	ENSG00000151923	7mer-m8	TargetScan
miR-622	ENSG00000152104	7mer-m8	TargetScan
miR-622	ENSG00000152147	7mer-m8	TargetScan
miR-622	ENSG00000152601	7mer-m8	TargetScan
miR-622	ENSG00000152672	7mer-m8	TargetScan

miR-622	ENSG00000153250	7mer-m8	TargetScan
miR-622	ENSG00000153339	7mer-m8	TargetScan
miR-622	ENSG00000153487	7mer-m8	TargetScan
miR-622	ENSG00000153898	7mer-m8	TargetScan
miR-622	ENSG00000154237	7mer-m8	TargetScan
miR-622	ENSG00000154856	7mer-m8	TargetScan
miR-622	ENSG00000155052	7mer-m8	TargetScan
miR-622	ENSG00000155926	7mer-m8	TargetScan
miR-622	ENSG00000156103	7mer-m8	TargetScan
miR-622	ENSG00000156239	7mer-m8	TargetScan
miR-622	ENSG00000156414	7mer-m8	TargetScan
miR-622	ENSG00000156471	7mer-m8	TargetScan
miR-622	ENSG00000156486	7mer-m8	TargetScan
miR-622	ENSG00000156671	7mer-m8	TargetScan
miR-622	ENSG00000156831	7mer-m8	TargetScan
miR-622	ENSG00000157214	7mer-m8	TargetScan
miR-622	ENSG00000157500	7mer-m8	TargetScan
miR-622	ENSG00000157741	7mer-m8	TargetScan
miR-622	ENSG00000158006	7mer-m8	TargetScan
miR-622	ENSG00000158163	7mer-m8	TargetScan
miR-622	ENSG00000158258	7mer-m8	TargetScan
miR-622	ENSG00000158445	7mer-m8	TargetScan
miR-622	ENSG00000158470	7mer-m8	TargetScan
miR-622	ENSG00000158555	7mer-m8	TargetScan
miR-622	ENSG00000158711	7mer-m8	TargetScan
miR-622	ENSG00000159256	7mer-m8	TargetScan
miR-622	ENSG00000159423	7mer-m8	TargetScan
miR-622	ENSG00000160208	7mer-m8	TargetScan
miR-622	ENSG00000160214	7mer-m8	TargetScan
miR-622	ENSG00000160685	7mer-m8	TargetScan
miR-622	ENSG00000160791	7mer-m8	TargetScan

miR-622	ENSG00000160886	7mer-m8	TargetScan
miR-622	ENSG00000160908	7mer-m8	TargetScan
miR-622	ENSG00000161813	7mer-m8	TargetScan
miR-622	ENSG00000162076	7mer-m8	TargetScan
miR-622	ENSG00000162129	7mer-m8	TargetScan
miR-622	ENSG00000162144	7mer-m8	TargetScan
miR-622	ENSG00000162419	7mer-m8	TargetScan
miR-622	ENSG00000162441	7mer-m8	TargetScan
miR-622	ENSG00000162444	7mer-m8	TargetScan
miR-622	ENSG00000162599	7mer-m8	TargetScan
miR-622	ENSG00000162739	7mer-m8	TargetScan
miR-622	ENSG00000162783	7mer-m8	TargetScan
miR-622	ENSG00000162804	7mer-m8	TargetScan
miR-622	ENSG00000162946	7mer-m8	TargetScan
miR-622	ENSG00000163092	7mer-m8	TargetScan
miR-622	ENSG00000163104	7mer-m8	TargetScan
miR-622	ENSG00000163288	7mer-m8	TargetScan
miR-622	ENSG00000163344	7mer-m8	TargetScan
miR-622	ENSG00000163412	7mer-m8	TargetScan
miR-622	ENSG00000163638	7mer-m8	TargetScan
miR-622	ENSG00000163655	7mer-m8	TargetScan
miR-622	ENSG00000163684	7mer-m8	TargetScan
miR-622	ENSG00000163812	7mer-m8	TargetScan
miR-622	ENSG00000163872	7mer-m8	TargetScan
miR-622	ENSG00000163877	7mer-m8	TargetScan
miR-622	ENSG00000163909	7mer-m8	TargetScan
miR-622	ENSG00000163939	7mer-m8	TargetScan
miR-622	ENSG00000164022	7mer-m8	TargetScan
miR-622	ENSG00000164031	7mer-m8	TargetScan
miR-622	ENSG00000164050	7mer-m8	TargetScan
miR-622	ENSG00000164070	7mer-m8	TargetScan

miR-622	ENSG00000164164	7mer-m8	TargetScan
miR-622	ENSG00000164180	7mer-m8	TargetScan
miR-622	ENSG00000164220	7mer-m8	TargetScan
miR-622	ENSG00000164244	7mer-m8	TargetScan
miR-622	ENSG00000164253	7mer-m8	TargetScan
miR-622	ENSG00000164292	7mer-m8	TargetScan
miR-622	ENSG00000164466	7mer-m8	TargetScan
miR-622	ENSG00000164485	7mer-m8	TargetScan
miR-622	ENSG00000164574	7mer-m8	TargetScan
miR-622	ENSG00000164626	7mer-m8	TargetScan
miR-622	ENSG00000164631	7mer-m8	TargetScan
miR-622	ENSG00000164684	7mer-m8	TargetScan
miR-622	ENSG00000164690	7mer-m8	TargetScan
miR-622	ENSG00000164754	7mer-m8	TargetScan
miR-622	ENSG00000164938	7mer-m8	TargetScan
miR-622	ENSG00000165406	7mer-m8	TargetScan
miR-622	ENSG00000165633	7mer-m8	TargetScan
miR-622	ENSG00000165816	7mer-m8	TargetScan
miR-622	ENSG00000166037	7mer-m8	TargetScan
miR-622	ENSG00000166140	7mer-m8	TargetScan
miR-622	ENSG00000166233	7mer-m8	TargetScan
miR-622	ENSG00000166250	7mer-m8	TargetScan
miR-622	ENSG00000166263	7mer-m8	TargetScan
miR-622	ENSG00000166411	7mer-m8	TargetScan
miR-622	ENSG00000166435	7mer-m8	TargetScan
miR-622	ENSG00000166741	7mer-m8	TargetScan
miR-622	ENSG00000166747	7mer-m8	TargetScan
miR-622	ENSG00000166847	7mer-m8	TargetScan
miR-622	ENSG00000166862	7mer-m8	TargetScan
miR-622	ENSG00000166900	7mer-m8	TargetScan
miR-622	ENSG00000166963	7mer-m8	TargetScan

miR-622	ENSG00000167112	7mer-m8	TargetScan
miR-622	ENSG00000167196	7mer-m8	TargetScan
miR-622	ENSG00000167208	7mer-m8	TargetScan
miR-622	ENSG00000167377	7mer-m8	TargetScan
miR-622	ENSG00000167528	7mer-m8	TargetScan
miR-622	ENSG00000167721	7mer-m8	TargetScan
miR-622	ENSG00000167977	7mer-m8	TargetScan
miR-622	ENSG00000168118	7mer-m8	TargetScan
miR-622	ENSG00000168411	7mer-m8	TargetScan
miR-622	ENSG00000168461	7mer-m8	TargetScan
miR-622	ENSG00000168631	7mer-m8	TargetScan
miR-622	ENSG00000168734	7mer-m8	TargetScan
miR-622	ENSG00000168769	7mer-m8	TargetScan
miR-622	ENSG00000168795	7mer-m8	TargetScan
miR-622	ENSG00000168818	7mer-m8	TargetScan
miR-622	ENSG00000168939	7mer-m8	TargetScan
miR-622	ENSG00000168939	7mer-m8	TargetScan
miR-622	ENSG00000169021	7mer-m8	TargetScan
miR-622	ENSG00000169554	7mer-m8	TargetScan
miR-622	ENSG00000169814	7mer-m8	TargetScan
miR-622	ENSG00000169860	7mer-m8	TargetScan
miR-622	ENSG00000169905	7mer-m8	TargetScan
miR-622	ENSG00000169908	7mer-m8	TargetScan
miR-622	ENSG00000169955	7mer-m8	TargetScan
miR-622	ENSG00000169967	7mer-m8	TargetScan
miR-622	ENSG00000170017	7mer-m8	TargetScan
miR-622	ENSG00000170027	7mer-m8	TargetScan
miR-622	ENSG00000170153	7mer-m8	TargetScan
miR-622	ENSG00000170242	7mer-m8	TargetScan
miR-622	ENSG00000170500	7mer-m8	TargetScan
miR-622	ENSG00000170927	7mer-m8	TargetScan

miR-622	ENSG00000171451	7mer-m8	TargetScan
miR-622	ENSG00000171533	7mer-m8	TargetScan
miR-622	ENSG00000171596	7mer-m8	TargetScan
miR-622	ENSG00000171862	7mer-m8	TargetScan
miR-622	ENSG00000171865	7mer-m8	TargetScan
miR-622	ENSG00000172014	7mer-m8	TargetScan
miR-622	ENSG00000172575	7mer-m8	TargetScan
miR-622	ENSG00000172663	7mer-m8	TargetScan
miR-622	ENSG00000172671	7mer-m8	TargetScan
miR-622	ENSG00000172819	7mer-m8	TargetScan
miR-622	ENSG00000172840	7mer-m8	TargetScan
miR-622	ENSG00000172943	7mer-m8	TargetScan
miR-622	ENSG00000173275	7mer-m8	TargetScan
miR-622	ENSG00000173542	7mer-m8	TargetScan
miR-622	ENSG00000173674	7mer-m8	TargetScan
miR-622	ENSG00000173698	7mer-m8	TargetScan
miR-622	ENSG00000173947	7mer-m8	TargetScan
miR-622	ENSG00000174243	7mer-m8	TargetScan
miR-622	ENSG00000174485	7mer-m8	TargetScan
miR-622	ENSG00000174611	7mer-m8	TargetScan
miR-622	ENSG00000174640	7mer-m8	TargetScan
miR-622	ENSG00000174749	7mer-m8	TargetScan
miR-622	ENSG00000174953	7mer-m8	TargetScan
miR-622	ENSG00000175003	7mer-m8	TargetScan
miR-622	ENSG00000175104	7mer-m8	TargetScan
miR-622	ENSG00000175175	7mer-m8	TargetScan
miR-622	ENSG00000175387	7mer-m8	TargetScan
miR-622	ENSG00000175455	7mer-m8	TargetScan
miR-622	ENSG00000175497	7mer-m8	TargetScan
miR-622	ENSG00000175766	7mer-m8	TargetScan
miR-622	ENSG00000175854	7mer-m8	TargetScan

miR-622	ENSG00000176014	7mer-m8	TargetScan
miR-622	ENSG00000176171	7mer-m8	TargetScan
miR-622	ENSG00000176410	7mer-m8	TargetScan
miR-622	ENSG00000176723	7mer-m8	TargetScan
miR-622	ENSG00000177034	7mer-m8	TargetScan
miR-622	ENSG00000177511	7mer-m8	TargetScan
miR-622	ENSG00000177570	7mer-m8	TargetScan
miR-622	ENSG00000178038	7mer-m8	TargetScan
miR-622	ENSG00000178104	7mer-m8	TargetScan
miR-622	ENSG00000178217	7mer-m8	TargetScan
miR-622	ENSG00000178338	7mer-m8	TargetScan
miR-622	ENSG00000178385	7mer-m8	TargetScan
miR-622	ENSG00000179119	7mer-m8	TargetScan
miR-622	ENSG00000179152	7mer-m8	TargetScan
miR-622	ENSG00000179314	7mer-m8	TargetScan
miR-622	ENSG00000179399	7mer-m8	TargetScan
miR-622	ENSG00000179715	7mer-m8	TargetScan
miR-622	ENSG00000180011	7mer-m8	TargetScan
miR-622	ENSG00000180509	7mer-m8	TargetScan
miR-622	ENSG00000180543	7mer-m8	TargetScan
miR-622	ENSG00000180628	7mer-m8	TargetScan
miR-622	ENSG00000180758	7mer-m8	TargetScan
miR-622	ENSG00000180787	7mer-m8	TargetScan
miR-622	ENSG00000180822	7mer-m8	TargetScan
miR-622	ENSG00000180901	7mer-m8	TargetScan
miR-622	ENSG00000181163	7mer-m8	TargetScan
miR-622	ENSG00000181544	7mer-m8	TargetScan
miR-622	ENSG00000181577	7mer-m8	TargetScan
miR-622	ENSG00000181722	7mer-m8	TargetScan
miR-622	ENSG00000182218	7mer-m8	TargetScan
miR-622	ENSG00000182348	7mer-m8	TargetScan

miR-622	ENSG00000182575	7mer-m8	TargetScan
miR-622	ENSG00000182919	7mer-m8	TargetScan
miR-622	ENSG00000183023	7mer-m8	TargetScan
miR-622	ENSG00000183066	7mer-m8	TargetScan
miR-622	ENSG00000183098	7mer-m8	TargetScan
miR-622	ENSG00000183137	7mer-m8	TargetScan
miR-622	ENSG00000183148	7mer-m8	TargetScan
miR-622	ENSG00000183154	7mer-m8	TargetScan
miR-622	ENSG00000183250	7mer-m8	TargetScan
miR-622	ENSG00000183386	7mer-m8	TargetScan
miR-622	ENSG00000183423	7mer-m8	TargetScan
miR-622	ENSG00000183476	7mer-m8	TargetScan
miR-622	ENSG00000183784	7mer-m8	TargetScan
miR-622	ENSG00000184113	7mer-m8	TargetScan
miR-622	ENSG00000184347	7mer-m8	TargetScan
miR-622	ENSG00000184517	7mer-m8	TargetScan
miR-622	ENSG00000184571	7mer-m8	TargetScan
miR-622	ENSG00000184678	7mer-m8	TargetScan
miR-622	ENSG00000185163	7mer-m8	TargetScan
miR-622	ENSG00000185267	7mer-m8	TargetScan
miR-622	ENSG00000185278	7mer-m8	TargetScan
miR-622	ENSG00000185518	7mer-m8	TargetScan
miR-622	ENSG00000185681	7mer-m8	TargetScan
miR-622	ENSG00000186150	7mer-m8	TargetScan
miR-622	ENSG00000186687	7mer-m8	TargetScan
miR-622	ENSG00000186895	7mer-m8	TargetScan
miR-622	ENSG00000186908	7mer-m8	TargetScan
miR-622	ENSG00000187147	7mer-m8	TargetScan
miR-622	ENSG00000187510	7mer-m8	TargetScan
miR-622	ENSG00000187607	7mer-m8	TargetScan
miR-622	ENSG00000187664	7mer-m8	TargetScan

miR-622	ENSG00000187753	7mer-m8	TargetScan
miR-622	ENSG00000187792	7mer-m8	TargetScan
miR-622	ENSG00000187800	7mer-m8	TargetScan
miR-622	ENSG00000188026	7mer-m8	TargetScan
miR-622	ENSG00000188152	7mer-m8	TargetScan
miR-622	ENSG00000188176	7mer-m8	TargetScan
miR-622	ENSG00000188215	7mer-m8	TargetScan
miR-622	ENSG00000188266	7mer-m8	TargetScan
miR-622	ENSG00000188277	7mer-m8	TargetScan
miR-622	ENSG00000188511	7mer-m8	TargetScan
miR-622	ENSG00000188596	7mer-m8	TargetScan
miR-622	ENSG00000188610	7mer-m8	TargetScan
miR-622	ENSG00000188612	7mer-m8	TargetScan
miR-622	ENSG00000188921	7mer-m8	TargetScan
miR-622	ENSG00000189377	7mer-m8	TargetScan
miR-622	ENSG00000196167	7mer-m8	TargetScan
miR-622	ENSG00000196208	7mer-m8	TargetScan
miR-622	ENSG00000196323	7mer-m8	TargetScan
miR-622	ENSG00000196376	7mer-m8	TargetScan
miR-622	ENSG00000196505	7mer-m8	TargetScan
miR-622	ENSG00000196550	7mer-m8	TargetScan
miR-622	ENSG00000196569	7mer-m8	TargetScan
miR-622	ENSG00000196812	7mer-m8	TargetScan
miR-622	ENSG00000197081	7mer-m8	TargetScan
miR-622	ENSG00000197406	7mer-m8	TargetScan
miR-622	ENSG00000197557	7mer-m8	TargetScan
miR-622	ENSG00000197977	7mer-m8	TargetScan
miR-622	ENSG00000198040	7mer-m8	TargetScan
miR-622	ENSG00000198105	7mer-m8	TargetScan
miR-622	ENSG00000198160	7mer-m8	TargetScan
miR-622	ENSG00000198198	7mer-m8	TargetScan

miR-622	ENSG00000198612	7mer-m8	TargetScan
miR-622	ENSG00000198700	7mer-m8	TargetScan
miR-622	ENSG00000198720	7mer-m8	TargetScan
miR-622	ENSG00000198743	7mer-m8	TargetScan
miR-622	ENSG00000198799	7mer-m8	TargetScan
miR-622	ENSG00000198815	7mer-m8	TargetScan
miR-622	ENSG00000198821	7mer-m8	TargetScan
miR-622	ENSG00000198890	7mer-m8	TargetScan
miR-622	ENSG00000198914	7mer-m8	TargetScan
miR-622	ENSG00000203667	7mer-m8	TargetScan
miR-622	ENSG00000203867	7mer-m8	TargetScan
miR-622	ENSG00000204131	7mer-m8	TargetScan
miR-622	ENSG00000204174	7mer-m8	TargetScan
miR-622	ENSG00000204231	7mer-m8	TargetScan
miR-622	ENSG00000204381	7mer-m8	TargetScan
miR-622	ENSG00000204514	7mer-m8	TargetScan
miR-622	ENSG00000204520	7mer-m8	TargetScan
miR-622	ENSG00000204814	7mer-m8	TargetScan
miR-622	ENSG00000205060	7mer-m8	TargetScan
miR-622	ENSG00000205268	7mer-m8	TargetScan
miR-622	ENSG00000205334	7mer-m8	TargetScan
miR-622	ENSG00000205356	7mer-m8	TargetScan
miR-622	ENSG00000205765	7mer-m8	TargetScan
miR-622	ENSG00000206344	7mer-m8	TargetScan
miR-622	ENSG00000206579	7mer-m8	TargetScan
miR-622	ENSG00000212122	7mer-m8	TargetScan
miR-622	ENSG00000213380	7mer-m8	TargetScan
miR-622	ENSG00000213614	7mer-m8	TargetScan
miR-622	ENSG00000213694	7mer-m8	TargetScan
miR-622	ENSG00000213699	7mer-m8	TargetScan
miR-622	ENSG00000213741	7mer-m8	TargetScan

miR-622	ENSG00000214013	7mer-m8	TargetScan
miR-622	ENSG00000214097	7mer-m8	TargetScan
miR-622	ENSG00000214193	7mer-m8	TargetScan
miR-622	ENSG00000214367	7mer-m8	TargetScan
miR-622	ENSG00000214491	7mer-m8	TargetScan
miR-622	ENSG00000214655	7mer-m8	TargetScan
miR-622	ENSG00000215568	7mer-m8	TargetScan
miR-622	ENSG00000215784	7mer-m8	TargetScan
miR-622	ENSG00000221823	7mer-m8	TargetScan
miR-622	ENSG00000221866	7mer-m8	TargetScan
miR-622	ENSG00000221990	7mer-m8	TargetScan
miR-622	ENSG00000224916	7mer-m8	TargetScan
miR-622	ENSG00000233493	7mer-m8	TargetScan
miR-622	ENSG00000234906	7mer-m8	TargetScan
miR-622	ENSG00000235568	7mer-m8	TargetScan
miR-622	ENSG00000240021	7mer-m8	TargetScan
miR-622	ENSG00000240053	7mer-m8	TargetScan
miR-622	ENSG00000240682	7mer-m8	TargetScan
miR-622	ENSG00000243710	7mer-m8	TargetScan
miR-622	ENSG00000244005	7mer-m8	TargetScan
miR-622	ENSG00000244274	7mer-m8	TargetScan
miR-622	ENSG00000248485	7mer-m8	TargetScan
miR-622	ENSG00000249459	7mer-m8	TargetScan
miR-622	ENSG00000254726	7mer-m8	TargetScan
miR-622	ENSG00000255874	7mer-m8	TargetScan
miR-622	ENSG00000256043	7mer-m8	TargetScan
miR-622	ENSG00000256162	7mer-m8	TargetScan
miR-622	ENSG00000256229	7mer-m8	TargetScan
miR-622	ENSG00000256574	7mer-m8	TargetScan
miR-622	ENSG00000257184	7mer-m8	TargetScan
miR-622	ENSG00000257315	7mer-m8	TargetScan

miR-622	ENSG00000258429	7mer-m8	TargetScan
miR-622	ENSG00000258729	7mer-m8	TargetScan
miR-622	ENSG00000259030	7mer-m8	TargetScan
miR-622	ENSG00000261221	7mer-m8	TargetScan
miR-622	ENSG00000263002	7mer-m8	TargetScan
miR-622	ENSG00000263020	7mer-m8	TargetScan
miR-622	ENSG00000263513	7mer-m8	TargetScan
miR-622	ENSG00000268926	7mer-m8	TargetScan
miR-622	ENSG00000269155	7mer-m8	TargetScan
miR-622	ENSG00000269846	7mer-m8	TargetScan
miR-622	ENSG00000270757	7mer-m8	TargetScan
miR-622	ENSG00000272617	7mer-m8	TargetScan
miR-622	ENSG00000285347	7mer-m8	TargetScan
miR-7161-3p	ENSG00000001617	7mer-m8	TargetScan
miR-7161-3p	ENSG00000002746	7mer-m8	TargetScan
miR-7161-3p	ENSG00000003400	7mer-m8	TargetScan
miR-7161-3p	ENSG00000003402	7mer-m8	TargetScan
miR-7161-3p	ENSG00000004478	7mer-m8	TargetScan
miR-7161-3p	ENSG00000004700	7mer-m8	TargetScan
miR-7161-3p	ENSG00000005981	7mer-m8	TargetScan
miR-7161-3p	ENSG00000006116	7mer-m8	TargetScan
miR-7161-3p	ENSG00000007174	7mer-m8	TargetScan
miR-7161-3p	ENSG00000007237	7mer-m8	TargetScan
miR-7161-3p	ENSG00000008311	7mer-m8	TargetScan
miR-7161-3p	ENSG00000009694	7mer-m8	TargetScan
miR-7161-3p	ENSG00000011275	7mer-m8	TargetScan
miR-7161-3p	ENSG00000019995	7mer-m8	TargetScan
miR-7161-3p	ENSG00000031081	7mer-m8	TargetScan
miR-7161-3p	ENSG00000033122	7mer-m8	TargetScan
miR-7161-3p	ENSG00000036549	7mer-m8	TargetScan
miR-7161-3p	ENSG00000039139	7mer-m8	TargetScan

miR-7161-3p	ENSG00000046653	7mer-m8	TargetScan
miR-7161-3p	ENSG00000047457	7mer-m8	TargetScan
miR-7161-3p	ENSG00000048471	7mer-m8	TargetScan
miR-7161-3p	ENSG00000051382	7mer-m8	TargetScan
miR-7161-3p	ENSG00000056291	7mer-m8	TargetScan
miR-7161-3p	ENSG00000057657	7mer-m8	TargetScan
miR-7161-3p	ENSG00000057935	7mer-m8	TargetScan
miR-7161-3p	ENSG00000062598	7mer-m8	TargetScan
miR-7161-3p	ENSG00000064607	7mer-m8	TargetScan
miR-7161-3p	ENSG00000065325	7mer-m8	TargetScan
miR-7161-3p	ENSG00000065413	7mer-m8	TargetScan
miR-7161-3p	ENSG00000065457	7mer-m8	TargetScan
miR-7161-3p	ENSG00000065665	7mer-m8	TargetScan
miR-7161-3p	ENSG00000066084	7mer-m8	TargetScan
miR-7161-3p	ENSG00000066185	7mer-m8	TargetScan
miR-7161-3p	ENSG00000066813	7mer-m8	TargetScan
miR-7161-3p	ENSG00000067082	7mer-m8	TargetScan
miR-7161-3p	ENSG00000067533	7mer-m8	TargetScan
miR-7161-3p	ENSG00000067900	7mer-m8	TargetScan
miR-7161-3p	ENSG00000068024	7mer-m8	TargetScan
miR-7161-3p	ENSG00000069667	7mer-m8	TargetScan
miR-7161-3p	ENSG00000069812	7mer-m8	TargetScan
miR-7161-3p	ENSG00000070159	7mer-m8	TargetScan
miR-7161-3p	ENSG00000070214	7mer-m8	TargetScan
miR-7161-3p	ENSG00000070756	7mer-m8	TargetScan
miR-7161-3p	ENSG00000072401	7mer-m8	TargetScan
miR-7161-3p	ENSG00000073849	7mer-m8	TargetScan
miR-7161-3p	ENSG00000074695	7mer-m8	TargetScan
miR-7161-3p	ENSG00000074706	7mer-m8	TargetScan
miR-7161-3p	ENSG00000075142	7mer-m8	TargetScan
miR-7161-3p	ENSG00000075426	7mer-m8	TargetScan

miR-7161-3p	ENSG00000075568	7mer-m8	TargetScan
miR-7161-3p	ENSG00000077264	7mer-m8	TargetScan
miR-7161-3p	ENSG00000077498	7mer-m8	TargetScan
miR-7161-3p	ENSG00000077684	7mer-m8	TargetScan
miR-7161-3p	ENSG00000078124	7mer-m8	TargetScan
miR-7161-3p	ENSG00000078237	7mer-m8	TargetScan
miR-7161-3p	ENSG00000080298	7mer-m8	TargetScan
miR-7161-3p	ENSG00000080345	7mer-m8	TargetScan
miR-7161-3p	ENSG00000080561	7mer-m8	TargetScan
miR-7161-3p	ENSG00000080603	7mer-m8	TargetScan
miR-7161-3p	ENSG00000080822	7mer-m8	TargetScan
miR-7161-3p	ENSG00000082805	7mer-m8	TargetScan
miR-7161-3p	ENSG00000085563	7mer-m8	TargetScan
miR-7161-3p	ENSG00000086102	7mer-m8	TargetScan
miR-7161-3p	ENSG00000086619	7mer-m8	TargetScan
miR-7161-3p	ENSG00000087074	7mer-m8	TargetScan
miR-7161-3p	ENSG00000088356	7mer-m8	TargetScan
miR-7161-3p	ENSG00000089041	7mer-m8	TargetScan
miR-7161-3p	ENSG00000089916	7mer-m8	TargetScan
miR-7161-3p	ENSG00000090054	7mer-m8	TargetScan
miR-7161-3p	ENSG00000090060	7mer-m8	TargetScan
miR-7161-3p	ENSG00000090376	7mer-m8	TargetScan
miR-7161-3p	ENSG00000091039	7mer-m8	TargetScan
miR-7161-3p	ENSG00000091592	7mer-m8	TargetScan
miR-7161-3p	ENSG00000091831	7mer-m8	TargetScan
miR-7161-3p	ENSG00000092531	7mer-m8	TargetScan
miR-7161-3p	ENSG00000092871	7mer-m8	TargetScan
miR-7161-3p	ENSG00000096060	7mer-m8	TargetScan
miR-7161-3p	ENSG00000096401	7mer-m8	TargetScan
miR-7161-3p	ENSG00000097033	7mer-m8	TargetScan
miR-7161-3p	ENSG00000099337	7mer-m8	TargetScan

miR-7161-3p	ENSG00000099810	7mer-m8	TargetScan
miR-7161-3p	ENSG00000099968	7mer-m8	TargetScan
miR-7161-3p	ENSG00000100167	7mer-m8	TargetScan
miR-7161-3p	ENSG00000100201	7mer-m8	TargetScan
miR-7161-3p	ENSG00000100344	7mer-m8	TargetScan
miR-7161-3p	ENSG00000100345	7mer-m8	TargetScan
miR-7161-3p	ENSG00000100523	7mer-m8	TargetScan
miR-7161-3p	ENSG00000100568	7mer-m8	TargetScan
miR-7161-3p	ENSG00000100601	7mer-m8	TargetScan
miR-7161-3p	ENSG00000100678	7mer-m8	TargetScan
miR-7161-3p	ENSG00000100852	7mer-m8	TargetScan
miR-7161-3p	ENSG00000100934	7mer-m8	TargetScan
miR-7161-3p	ENSG00000101019	7mer-m8	TargetScan
miR-7161-3p	ENSG00000101144	7mer-m8	TargetScan
miR-7161-3p	ENSG00000101343	7mer-m8	TargetScan
miR-7161-3p	ENSG00000101751	7mer-m8	TargetScan
miR-7161-3p	ENSG00000101752	7mer-m8	TargetScan
miR-7161-3p	ENSG00000101842	7mer-m8	TargetScan
miR-7161-3p	ENSG00000101945	7mer-m8	TargetScan
miR-7161-3p	ENSG00000102172	7mer-m8	TargetScan
miR-7161-3p	ENSG00000102755	7mer-m8	TargetScan
miR-7161-3p	ENSG00000104290	7mer-m8	TargetScan
miR-7161-3p	ENSG00000104447	7mer-m8	TargetScan
miR-7161-3p	ENSG00000104626	7mer-m8	TargetScan
miR-7161-3p	ENSG00000104756	7mer-m8	TargetScan
miR-7161-3p	ENSG00000105392	7mer-m8	TargetScan
miR-7161-3p	ENSG00000105656	7mer-m8	TargetScan
miR-7161-3p	ENSG00000105819	7mer-m8	TargetScan
miR-7161-3p	ENSG00000105835	7mer-m8	TargetScan
miR-7161-3p	ENSG00000105968	7mer-m8	TargetScan
miR-7161-3p	ENSG00000106331	7mer-m8	TargetScan

miR-7161-3p	ENSG00000106609	7mer-m8	TargetScan
miR-7161-3p	ENSG00000106689	7mer-m8	TargetScan
miR-7161-3p	ENSG00000106733	7mer-m8	TargetScan
miR-7161-3p	ENSG00000108387	7mer-m8	TargetScan
miR-7161-3p	ENSG00000108395	7mer-m8	TargetScan
miR-7161-3p	ENSG00000108669	7mer-m8	TargetScan
miR-7161-3p	ENSG00000109046	7mer-m8	TargetScan
miR-7161-3p	ENSG00000109332	7mer-m8	TargetScan
miR-7161-3p	ENSG00000109381	7mer-m8	TargetScan
miR-7161-3p	ENSG00000109667	7mer-m8	TargetScan
miR-7161-3p	ENSG00000109787	7mer-m8	TargetScan
miR-7161-3p	ENSG00000110395	7mer-m8	TargetScan
miR-7161-3p	ENSG00000110427	7mer-m8	TargetScan
miR-7161-3p	ENSG00000110723	7mer-m8	TargetScan
miR-7161-3p	ENSG00000111012	7mer-m8	TargetScan
miR-7161-3p	ENSG00000111142	7mer-m8	TargetScan
miR-7161-3p	ENSG00000111596	7mer-m8	TargetScan
miR-7161-3p	ENSG00000111727	7mer-m8	TargetScan
miR-7161-3p	ENSG00000111732	7mer-m8	TargetScan
miR-7161-3p	ENSG00000111850	7mer-m8	TargetScan
miR-7161-3p	ENSG00000112246	7mer-m8	TargetScan
miR-7161-3p	ENSG00000112249	7mer-m8	TargetScan
miR-7161-3p	ENSG00000112357	7mer-m8	TargetScan
miR-7161-3p	ENSG00000112796	7mer-m8	TargetScan
miR-7161-3p	ENSG00000112902	7mer-m8	TargetScan
miR-7161-3p	ENSG00000112964	7mer-m8	TargetScan
miR-7161-3p	ENSG00000113361	7mer-m8	TargetScan
miR-7161-3p	ENSG00000113369	7mer-m8	TargetScan
miR-7161-3p	ENSG00000113384	7mer-m8	TargetScan
miR-7161-3p	ENSG00000114030	7mer-m8	TargetScan
miR-7161-3p	ENSG00000114346	7mer-m8	TargetScan

miR-7161-3p	ENSG00000114374	7mer-m8	TargetScan
miR-7161-3p	ENSG00000114656	7mer-m8	TargetScan
miR-7161-3p	ENSG00000114686	7mer-m8	TargetScan
miR-7161-3p	ENSG00000114933	7mer-m8	TargetScan
miR-7161-3p	ENSG00000114948	7mer-m8	TargetScan
miR-7161-3p	ENSG00000115109	7mer-m8	TargetScan
miR-7161-3p	ENSG00000115252	7mer-m8	TargetScan
miR-7161-3p	ENSG00000115355	7mer-m8	TargetScan
miR-7161-3p	ENSG00000115750	7mer-m8	TargetScan
miR-7161-3p	ENSG00000115827	7mer-m8	TargetScan
miR-7161-3p	ENSG00000116273	7mer-m8	TargetScan
miR-7161-3p	ENSG00000116641	7mer-m8	TargetScan
miR-7161-3p	ENSG00000116747	7mer-m8	TargetScan
miR-7161-3p	ENSG00000116750	7mer-m8	TargetScan
miR-7161-3p	ENSG00000117020	7mer-m8	TargetScan
miR-7161-3p	ENSG00000117533	7mer-m8	TargetScan
miR-7161-3p	ENSG00000118432	7mer-m8	TargetScan
miR-7161-3p	ENSG00000118508	7mer-m8	TargetScan
miR-7161-3p	ENSG00000119509	7mer-m8	TargetScan
miR-7161-3p	ENSG00000119547	7mer-m8	TargetScan
miR-7161-3p	ENSG00000119900	7mer-m8	TargetScan
miR-7161-3p	ENSG00000119953	7mer-m8	TargetScan
miR-7161-3p	ENSG00000119969	7mer-m8	TargetScan
miR-7161-3p	ENSG00000121152	7mer-m8	TargetScan
miR-7161-3p	ENSG00000121281	7mer-m8	TargetScan
miR-7161-3p	ENSG00000121413	7mer-m8	TargetScan
miR-7161-3p	ENSG00000121579	7mer-m8	TargetScan
miR-7161-3p	ENSG00000122378	7mer-m8	TargetScan
miR-7161-3p	ENSG00000122591	7mer-m8	TargetScan
miR-7161-3p	ENSG00000122778	7mer-m8	TargetScan
miR-7161-3p	ENSG00000123104	7mer-m8	TargetScan

miR-7161-3p	ENSG00000123892	7mer-m8	TargetScan
miR-7161-3p	ENSG00000123983	7mer-m8	TargetScan
miR-7161-3p	ENSG00000124802	7mer-m8	TargetScan
miR-7161-3p	ENSG00000125520	7mer-m8	TargetScan
miR-7161-3p	ENSG00000125703	7mer-m8	TargetScan
miR-7161-3p	ENSG00000125812	7mer-m8	TargetScan
miR-7161-3p	ENSG00000126070	7mer-m8	TargetScan
miR-7161-3p	ENSG00000126773	7mer-m8	TargetScan
miR-7161-3p	ENSG00000127329	7mer-m8	TargetScan
miR-7161-3p	ENSG00000128059	7mer-m8	TargetScan
miR-7161-3p	ENSG00000128510	7mer-m8	TargetScan
miR-7161-3p	ENSG00000129292	7mer-m8	TargetScan
miR-7161-3p	ENSG00000129559	7mer-m8	TargetScan
miR-7161-3p	ENSG00000130348	7mer-m8	TargetScan
miR-7161-3p	ENSG00000130827	7mer-m8	TargetScan
miR-7161-3p	ENSG00000130844	7mer-m8	TargetScan
miR-7161-3p	ENSG00000131115	7mer-m8	TargetScan
miR-7161-3p	ENSG00000131238	7mer-m8	TargetScan
miR-7161-3p	ENSG00000131323	7mer-m8	TargetScan
miR-7161-3p	ENSG00000131375	7mer-m8	TargetScan
miR-7161-3p	ENSG00000131381	7mer-m8	TargetScan
miR-7161-3p	ENSG00000131711	7mer-m8	TargetScan
miR-7161-3p	ENSG00000133019	7mer-m8	TargetScan
miR-7161-3p	ENSG00000133083	7mer-m8	TargetScan
miR-7161-3p	ENSG00000133687	7mer-m8	TargetScan
miR-7161-3p	ENSG00000133731	7mer-m8	TargetScan
miR-7161-3p	ENSG00000134253	7mer-m8	TargetScan
miR-7161-3p	ENSG00000134333	7mer-m8	TargetScan
miR-7161-3p	ENSG00000134352	7mer-m8	TargetScan
miR-7161-3p	ENSG00000134531	7mer-m8	TargetScan
miR-7161-3p	ENSG00000134940	7mer-m8	TargetScan

miR-7161-3p	ENSG00000135049	7mer-m8	TargetScan
miR-7161-3p	ENSG00000135185	7mer-m8	TargetScan
miR-7161-3p	ENSG00000135341	7mer-m8	TargetScan
miR-7161-3p	ENSG00000135414	7mer-m8	TargetScan
miR-7161-3p	ENSG00000135547	7mer-m8	TargetScan
miR-7161-3p	ENSG00000135778	7mer-m8	TargetScan
miR-7161-3p	ENSG00000135930	7mer-m8	TargetScan
miR-7161-3p	ENSG00000136371	7mer-m8	TargetScan
miR-7161-3p	ENSG00000137055	7mer-m8	TargetScan
miR-7161-3p	ENSG00000137210	7mer-m8	TargetScan
miR-7161-3p	ENSG00000137414	7mer-m8	TargetScan
miR-7161-3p	ENSG00000137460	7mer-m8	TargetScan
miR-7161-3p	ENSG00000137502	7mer-m8	TargetScan
miR-7161-3p	ENSG00000137573	7mer-m8	TargetScan
miR-7161-3p	ENSG00000137642	7mer-m8	TargetScan
miR-7161-3p	ENSG00000137941	7mer-m8	TargetScan
miR-7161-3p	ENSG00000138109	7mer-m8	TargetScan
miR-7161-3p	ENSG00000138279	7mer-m8	TargetScan
miR-7161-3p	ENSG00000138347	7mer-m8	TargetScan
miR-7161-3p	ENSG00000138395	7mer-m8	TargetScan
miR-7161-3p	ENSG00000138755	7mer-m8	TargetScan
miR-7161-3p	ENSG00000138802	7mer-m8	TargetScan
miR-7161-3p	ENSG00000139133	7mer-m8	TargetScan
miR-7161-3p	ENSG00000139291	7mer-m8	TargetScan
miR-7161-3p	ENSG00000139668	7mer-m8	TargetScan
miR-7161-3p	ENSG00000139734	7mer-m8	TargetScan
miR-7161-3p	ENSG00000139746	7mer-m8	TargetScan
miR-7161-3p	ENSG00000139874	7mer-m8	TargetScan
miR-7161-3p	ENSG00000140691	7mer-m8	TargetScan
miR-7161-3p	ENSG00000140807	7mer-m8	TargetScan
miR-7161-3p	ENSG00000140853	7mer-m8	TargetScan

miR-7161-3p	ENSG00000141161	7mer-m8	TargetScan
miR-7161-3p	ENSG00000141179	7mer-m8	TargetScan
miR-7161-3p	ENSG00000141524	7mer-m8	TargetScan
miR-7161-3p	ENSG00000141570	7mer-m8	TargetScan
miR-7161-3p	ENSG00000143217	7mer-m8	TargetScan
miR-7161-3p	ENSG00000143248	7mer-m8	TargetScan
miR-7161-3p	ENSG00000143252	7mer-m8	TargetScan
miR-7161-3p	ENSG00000143337	7mer-m8	TargetScan
miR-7161-3p	ENSG00000143498	7mer-m8	TargetScan
miR-7161-3p	ENSG00000143603	7mer-m8	TargetScan
miR-7161-3p	ENSG00000143842	7mer-m8	TargetScan
miR-7161-3p	ENSG00000143970	7mer-m8	TargetScan
miR-7161-3p	ENSG00000144283	7mer-m8	TargetScan
miR-7161-3p	ENSG00000144407	7mer-m8	TargetScan
miR-7161-3p	ENSG00000144481	7mer-m8	TargetScan
miR-7161-3p	ENSG00000144824	7mer-m8	TargetScan
miR-7161-3p	ENSG00000144908	7mer-m8	TargetScan
miR-7161-3p	ENSG00000145335	7mer-m8	TargetScan
miR-7161-3p	ENSG00000145384	7mer-m8	TargetScan
miR-7161-3p	ENSG00000145725	7mer-m8	TargetScan
miR-7161-3p	ENSG00000145734	7mer-m8	TargetScan
miR-7161-3p	ENSG00000145743	7mer-m8	TargetScan
miR-7161-3p	ENSG00000145781	7mer-m8	TargetScan
miR-7161-3p	ENSG00000145826	7mer-m8	TargetScan
miR-7161-3p	ENSG00000145832	7mer-m8	TargetScan
miR-7161-3p	ENSG00000146267	7mer-m8	TargetScan
miR-7161-3p	ENSG00000146278	7mer-m8	TargetScan
miR-7161-3p	ENSG00000146285	7mer-m8	TargetScan
miR-7161-3p	ENSG00000146414	7mer-m8	TargetScan
miR-7161-3p	ENSG00000146463	7mer-m8	TargetScan
miR-7161-3p	ENSG00000146674	7mer-m8	TargetScan

miR-7161-3p	ENSG00000147535	7mer-m8	TargetScan
miR-7161-3p	ENSG00000148411	7mer-m8	TargetScan
miR-7161-3p	ENSG00000148429	7mer-m8	TargetScan
miR-7161-3p	ENSG00000148600	7mer-m8	TargetScan
miR-7161-3p	ENSG00000148672	7mer-m8	TargetScan
miR-7161-3p	ENSG00000149124	7mer-m8	TargetScan
miR-7161-3p	ENSG00000149218	7mer-m8	TargetScan
miR-7161-3p	ENSG00000150394	7mer-m8	TargetScan
miR-7161-3p	ENSG00000150471	7mer-m8	TargetScan
miR-7161-3p	ENSG00000150477	7mer-m8	TargetScan
miR-7161-3p	ENSG00000150938	7mer-m8	TargetScan
miR-7161-3p	ENSG00000151135	7mer-m8	TargetScan
miR-7161-3p	ENSG00000151422	7mer-m8	TargetScan
miR-7161-3p	ENSG00000151491	7mer-m8	TargetScan
miR-7161-3p	ENSG00000151892	7mer-m8	TargetScan
miR-7161-3p	ENSG00000152192	7mer-m8	TargetScan
miR-7161-3p	ENSG00000152207	7mer-m8	TargetScan
miR-7161-3p	ENSG00000152380	7mer-m8	TargetScan
miR-7161-3p	ENSG00000152601	7mer-m8	TargetScan
miR-7161-3p	ENSG00000152749	7mer-m8	TargetScan
miR-7161-3p	ENSG00000152782	7mer-m8	TargetScan
miR-7161-3p	ENSG00000153214	7mer-m8	TargetScan
miR-7161-3p	ENSG00000153790	7mer-m8	TargetScan
miR-7161-3p	ENSG00000153827	7mer-m8	TargetScan
miR-7161-3p	ENSG00000154222	7mer-m8	TargetScan
miR-7161-3p	ENSG00000154319	7mer-m8	TargetScan
miR-7161-3p	ENSG00000154710	7mer-m8	TargetScan
miR-7161-3p	ENSG00000154845	7mer-m8	TargetScan
miR-7161-3p	ENSG00000155016	7mer-m8	TargetScan
miR-7161-3p	ENSG00000155052	7mer-m8	TargetScan
miR-7161-3p	ENSG00000155097	7mer-m8	TargetScan

miR-7161-3p	ENSG00000155229	7mer-m8	TargetScan
miR-7161-3p	ENSG00000155926	7mer-m8	TargetScan
miR-7161-3p	ENSG00000155966	7mer-m8	TargetScan
miR-7161-3p	ENSG00000156162	7mer-m8	TargetScan
miR-7161-3p	ENSG00000156711	7mer-m8	TargetScan
miR-7161-3p	ENSG00000157036	7mer-m8	TargetScan
miR-7161-3p	ENSG00000157131	7mer-m8	TargetScan
miR-7161-3p	ENSG00000157540	7mer-m8	TargetScan
miR-7161-3p	ENSG00000158163	7mer-m8	TargetScan
miR-7161-3p	ENSG00000158258	7mer-m8	TargetScan
miR-7161-3p	ENSG00000160049	7mer-m8	TargetScan
miR-7161-3p	ENSG00000160200	7mer-m8	TargetScan
miR-7161-3p	ENSG00000160551	7mer-m8	TargetScan
miR-7161-3p	ENSG00000161405	7mer-m8	TargetScan
miR-7161-3p	ENSG00000161911	7mer-m8	TargetScan
miR-7161-3p	ENSG00000162409	7mer-m8	TargetScan
miR-7161-3p	ENSG00000162545	7mer-m8	TargetScan
miR-7161-3p	ENSG00000162614	7mer-m8	TargetScan
miR-7161-3p	ENSG00000162616	7mer-m8	TargetScan
miR-7161-3p	ENSG00000162636	7mer-m8	TargetScan
miR-7161-3p	ENSG00000162645	7mer-m8	TargetScan
miR-7161-3p	ENSG00000162733	7mer-m8	TargetScan
miR-7161-3p	ENSG00000162849	7mer-m8	TargetScan
miR-7161-3p	ENSG00000163125	7mer-m8	TargetScan
miR-7161-3p	ENSG00000163235	7mer-m8	TargetScan
miR-7161-3p	ENSG00000163281	7mer-m8	TargetScan
miR-7161-3p	ENSG00000163297	7mer-m8	TargetScan
miR-7161-3p	ENSG00000163507	7mer-m8	TargetScan
miR-7161-3p	ENSG00000163605	7mer-m8	TargetScan
miR-7161-3p	ENSG00000163608	7mer-m8	TargetScan
miR-7161-3p	ENSG00000163697	7mer-m8	TargetScan

miR-7161-3p	ENSG00000163710	7mer-m8	TargetScan
miR-7161-3p	ENSG00000163904	7mer-m8	TargetScan
miR-7161-3p	ENSG00000163950	7mer-m8	TargetScan
miR-7161-3p	ENSG00000164048	7mer-m8	TargetScan
miR-7161-3p	ENSG00000164061	7mer-m8	TargetScan
miR-7161-3p	ENSG00000164113	7mer-m8	TargetScan
miR-7161-3p	ENSG00000164299	7mer-m8	TargetScan
miR-7161-3p	ENSG00000164309	7mer-m8	TargetScan
miR-7161-3p	ENSG00000164430	7mer-m8	TargetScan
miR-7161-3p	ENSG00000164484	7mer-m8	TargetScan
miR-7161-3p	ENSG00000164651	7mer-m8	TargetScan
miR-7161-3p	ENSG00000164663	7mer-m8	TargetScan
miR-7161-3p	ENSG00000164736	7mer-m8	TargetScan
miR-7161-3p	ENSG00000164879	7mer-m8	TargetScan
miR-7161-3p	ENSG00000165084	7mer-m8	TargetScan
miR-7161-3p	ENSG00000165120	7mer-m8	TargetScan
miR-7161-3p	ENSG00000165194	7mer-m8	TargetScan
miR-7161-3p	ENSG00000165195	7mer-m8	TargetScan
miR-7161-3p	ENSG00000165240	7mer-m8	TargetScan
miR-7161-3p	ENSG00000165416	7mer-m8	TargetScan
miR-7161-3p	ENSG00000165434	7mer-m8	TargetScan
miR-7161-3p	ENSG00000165609	7mer-m8	TargetScan
miR-7161-3p	ENSG00000165633	7mer-m8	TargetScan
miR-7161-3p	ENSG00000165688	7mer-m8	TargetScan
miR-7161-3p	ENSG00000165757	7mer-m8	TargetScan
miR-7161-3p	ENSG00000165841	7mer-m8	TargetScan
miR-7161-3p	ENSG00000166206	7mer-m8	TargetScan
miR-7161-3p	ENSG00000166265	7mer-m8	TargetScan
miR-7161-3p	ENSG00000166272	7mer-m8	TargetScan
miR-7161-3p	ENSG00000166441	7mer-m8	TargetScan
miR-7161-3p	ENSG00000166569	7mer-m8	TargetScan

miR-7161-3p	ENSG00000166664	7mer-m8	TargetScan
miR-7161-3p	ENSG00000166797	7mer-m8	TargetScan
miR-7161-3p	ENSG00000166848	7mer-m8	TargetScan
miR-7161-3p	ENSG00000166928	7mer-m8	TargetScan
miR-7161-3p	ENSG00000167637	7mer-m8	TargetScan
miR-7161-3p	ENSG00000167981	7mer-m8	TargetScan
miR-7161-3p	ENSG00000168118	7mer-m8	TargetScan
miR-7161-3p	ENSG00000168564	7mer-m8	TargetScan
miR-7161-3p	ENSG00000168566	7mer-m8	TargetScan
miR-7161-3p	ENSG00000168743	7mer-m8	TargetScan
miR-7161-3p	ENSG00000168906	7mer-m8	TargetScan
miR-7161-3p	ENSG00000169306	7mer-m8	TargetScan
miR-7161-3p	ENSG00000169856	7mer-m8	TargetScan
miR-7161-3p	ENSG00000169905	7mer-m8	TargetScan
miR-7161-3p	ENSG00000169914	7mer-m8	TargetScan
miR-7161-3p	ENSG00000169946	7mer-m8	TargetScan
miR-7161-3p	ENSG00000169955	7mer-m8	TargetScan
miR-7161-3p	ENSG00000170419	7mer-m8	TargetScan
miR-7161-3p	ENSG00000170677	7mer-m8	TargetScan
miR-7161-3p	ENSG00000171004	7mer-m8	TargetScan
miR-7161-3p	ENSG00000171109	7mer-m8	TargetScan
miR-7161-3p	ENSG00000171320	7mer-m8	TargetScan
miR-7161-3p	ENSG00000171435	7mer-m8	TargetScan
miR-7161-3p	ENSG00000171530	7mer-m8	TargetScan
miR-7161-3p	ENSG00000171634	7mer-m8	TargetScan
miR-7161-3p	ENSG00000171649	7mer-m8	TargetScan
miR-7161-3p	ENSG00000171791	7mer-m8	TargetScan
miR-7161-3p	ENSG00000171817	7mer-m8	TargetScan
miR-7161-3p	ENSG00000171928	7mer-m8	TargetScan
miR-7161-3p	ENSG00000172380	7mer-m8	TargetScan
miR-7161-3p	ENSG00000173068	7mer-m8	TargetScan

miR-7161-3p	ENSG00000173349	7mer-m8	TargetScan
miR-7161-3p	ENSG00000173641	7mer-m8	TargetScan
miR-7161-3p	ENSG00000173681	7mer-m8	TargetScan
miR-7161-3p	ENSG00000173786	7mer-m8	TargetScan
miR-7161-3p	ENSG00000174749	7mer-m8	TargetScan
miR-7161-3p	ENSG00000174792	7mer-m8	TargetScan
miR-7161-3p	ENSG00000174943	7mer-m8	TargetScan
miR-7161-3p	ENSG00000175066	7mer-m8	TargetScan
miR-7161-3p	ENSG00000175097	7mer-m8	TargetScan
miR-7161-3p	ENSG00000175344	7mer-m8	TargetScan
miR-7161-3p	ENSG00000175387	7mer-m8	TargetScan
miR-7161-3p	ENSG00000175395	7mer-m8	TargetScan
miR-7161-3p	ENSG00000175893	7mer-m8	TargetScan
miR-7161-3p	ENSG00000176371	7mer-m8	TargetScan
miR-7161-3p	ENSG00000176595	7mer-m8	TargetScan
miR-7161-3p	ENSG00000176887	7mer-m8	TargetScan
miR-7161-3p	ENSG00000176915	7mer-m8	TargetScan
miR-7161-3p	ENSG00000176953	7mer-m8	TargetScan
miR-7161-3p	ENSG00000177034	7mer-m8	TargetScan
miR-7161-3p	ENSG00000177181	7mer-m8	TargetScan
miR-7161-3p	ENSG00000177614	7mer-m8	TargetScan
miR-7161-3p	ENSG00000177689	7mer-m8	TargetScan
miR-7161-3p	ENSG00000177707	7mer-m8	TargetScan
miR-7161-3p	ENSG00000177733	7mer-m8	TargetScan
miR-7161-3p	ENSG00000178033	7mer-m8	TargetScan
miR-7161-3p	ENSG00000178171	7mer-m8	TargetScan
miR-7161-3p	ENSG00000178201	7mer-m8	TargetScan
miR-7161-3p	ENSG00000178338	7mer-m8	TargetScan
miR-7161-3p	ENSG00000178662	7mer-m8	TargetScan
miR-7161-3p	ENSG00000178752	7mer-m8	TargetScan
miR-7161-3p	ENSG00000179133	7mer-m8	TargetScan

miR-7161-3p	ENSG00000179520	7mer-m8	TargetScan
miR-7161-3p	ENSG00000179813	7mer-m8	TargetScan
miR-7161-3p	ENSG00000179933	7mer-m8	TargetScan
miR-7161-3p	ENSG00000180008	7mer-m8	TargetScan
miR-7161-3p	ENSG00000180525	7mer-m8	TargetScan
miR-7161-3p	ENSG00000180884	7mer-m8	TargetScan
miR-7161-3p	ENSG00000180914	7mer-m8	TargetScan
miR-7161-3p	ENSG00000181518	7mer-m8	TargetScan
miR-7161-3p	ENSG00000181982	7mer-m8	TargetScan
miR-7161-3p	ENSG00000182261	7mer-m8	TargetScan
miR-7161-3p	ENSG00000182287	7mer-m8	TargetScan
miR-7161-3p	ENSG00000182348	7mer-m8	TargetScan
miR-7161-3p	ENSG00000182359	7mer-m8	TargetScan
miR-7161-3p	ENSG00000182890	7mer-m8	TargetScan
miR-7161-3p	ENSG00000183114	7mer-m8	TargetScan
miR-7161-3p	ENSG00000183695	7mer-m8	TargetScan
miR-7161-3p	ENSG00000183742	7mer-m8	TargetScan
miR-7161-3p	ENSG00000183747	7mer-m8	TargetScan
miR-7161-3p	ENSG00000183762	7mer-m8	TargetScan
miR-7161-3p	ENSG00000184378	7mer-m8	TargetScan
miR-7161-3p	ENSG00000184937	7mer-m8	TargetScan
miR-7161-3p	ENSG00000185565	7mer-m8	TargetScan
miR-7161-3p	ENSG00000186103	7mer-m8	TargetScan
miR-7161-3p	ENSG00000186265	7mer-m8	TargetScan
miR-7161-3p	ENSG00000186479	7mer-m8	TargetScan
miR-7161-3p	ENSG00000186838	7mer-m8	TargetScan
miR-7161-3p	ENSG00000187134	7mer-m8	TargetScan
miR-7161-3p	ENSG00000187726	7mer-m8	TargetScan
miR-7161-3p	ENSG00000187792	7mer-m8	TargetScan
miR-7161-3p	ENSG00000187866	7mer-m8	TargetScan
miR-7161-3p	ENSG00000188056	7mer-m8	TargetScan

miR-7161-3p	ENSG00000188089	7mer-m8	TargetScan
miR-7161-3p	ENSG00000188215	7mer-m8	TargetScan
miR-7161-3p	ENSG00000188227	7mer-m8	TargetScan
miR-7161-3p	ENSG00000188266	7mer-m8	TargetScan
miR-7161-3p	ENSG00000188282	7mer-m8	TargetScan
miR-7161-3p	ENSG00000188343	7mer-m8	TargetScan
miR-7161-3p	ENSG00000188386	7mer-m8	TargetScan
miR-7161-3p	ENSG00000188730	7mer-m8	TargetScan
miR-7161-3p	ENSG00000188811	7mer-m8	TargetScan
miR-7161-3p	ENSG00000189057	7mer-m8	TargetScan
miR-7161-3p	ENSG00000189180	7mer-m8	TargetScan
miR-7161-3p	ENSG00000189190	7mer-m8	TargetScan
miR-7161-3p	ENSG00000189233	7mer-m8	TargetScan
miR-7161-3p	ENSG00000196208	7mer-m8	TargetScan
miR-7161-3p	ENSG00000196233	7mer-m8	TargetScan
miR-7161-3p	ENSG00000196284	7mer-m8	TargetScan
miR-7161-3p	ENSG00000196381	7mer-m8	TargetScan
miR-7161-3p	ENSG00000196504	7mer-m8	TargetScan
miR-7161-3p	ENSG00000196549	7mer-m8	TargetScan
miR-7161-3p	ENSG00000196693	7mer-m8	TargetScan
miR-7161-3p	ENSG00000196876	7mer-m8	TargetScan
miR-7161-3p	ENSG00000196937	7mer-m8	TargetScan
miR-7161-3p	ENSG00000197063	7mer-m8	TargetScan
miR-7161-3p	ENSG00000197142	7mer-m8	TargetScan
miR-7161-3p	ENSG00000197181	7mer-m8	TargetScan
miR-7161-3p	ENSG00000197183	7mer-m8	TargetScan
miR-7161-3p	ENSG00000197506	7mer-m8	TargetScan
miR-7161-3p	ENSG00000197557	7mer-m8	TargetScan
miR-7161-3p	ENSG00000197665	7mer-m8	TargetScan
miR-7161-3p	ENSG00000197724	7mer-m8	TargetScan
miR-7161-3p	ENSG00000197782	7mer-m8	TargetScan

miR-7161-3p	ENSG00000198015	7mer-m8	TargetScan
miR-7161-3p	ENSG00000198108	7mer-m8	TargetScan
miR-7161-3p	ENSG00000198162	7mer-m8	TargetScan
miR-7161-3p	ENSG00000198168	7mer-m8	TargetScan
miR-7161-3p	ENSG00000198464	7mer-m8	TargetScan
miR-7161-3p	ENSG00000198561	7mer-m8	TargetScan
miR-7161-3p	ENSG00000198677	7mer-m8	TargetScan
miR-7161-3p	ENSG00000198826	7mer-m8	TargetScan
miR-7161-3p	ENSG00000198873	7mer-m8	TargetScan
miR-7161-3p	ENSG00000198933	7mer-m8	TargetScan
miR-7161-3p	ENSG00000198944	7mer-m8	TargetScan
miR-7161-3p	ENSG00000204022	7mer-m8	TargetScan
miR-7161-3p	ENSG00000204118	7mer-m8	TargetScan
miR-7161-3p	ENSG00000204130	7mer-m8	TargetScan
miR-7161-3p	ENSG00000204179	7mer-m8	TargetScan
miR-7161-3p	ENSG00000204262	7mer-m8	TargetScan
miR-7161-3p	ENSG00000204397	7mer-m8	TargetScan
miR-7161-3p	ENSG00000204580	7mer-m8	TargetScan
miR-7161-3p	ENSG00000204634	7mer-m8	TargetScan
miR-7161-3p	ENSG00000204694	7mer-m8	TargetScan
miR-7161-3p	ENSG00000205084	7mer-m8	TargetScan
miR-7161-3p	ENSG00000205269	7mer-m8	TargetScan
miR-7161-3p	ENSG00000205339	7mer-m8	TargetScan
miR-7161-3p	ENSG00000205413	7mer-m8	TargetScan
miR-7161-3p	ENSG00000205726	7mer-m8	TargetScan
miR-7161-3p	ENSG00000206538	7mer-m8	TargetScan
miR-7161-3p	ENSG00000206557	7mer-m8	TargetScan
miR-7161-3p	ENSG00000213029	7mer-m8	TargetScan
miR-7161-3p	ENSG00000213160	7mer-m8	TargetScan
miR-7161-3p	ENSG00000213186	7mer-m8	TargetScan
miR-7161-3p	ENSG00000213337	7mer-m8	TargetScan

miR-7161-3p	ENSG00000213551	7mer-m8	TargetScan
miR-7161-3p	ENSG00000213625	7mer-m8	TargetScan
miR-7161-3p	ENSG00000213760	7mer-m8	TargetScan
miR-7161-3p	ENSG00000213762	7mer-m8	TargetScan
miR-7161-3p	ENSG00000214216	7mer-m8	TargetScan
miR-7161-3p	ENSG00000214456	7mer-m8	TargetScan
miR-7161-3p	ENSG00000217930	7mer-m8	TargetScan
miR-7161-3p	ENSG00000218336	7mer-m8	TargetScan
miR-7161-3p	ENSG00000224531	7mer-m8	TargetScan
miR-7161-3p	ENSG00000228157	7mer-m8	TargetScan
miR-7161-3p	ENSG00000241388	7mer-m8	TargetScan
miR-7161-3p	ENSG00000242265	7mer-m8	TargetScan
miR-7161-3p	ENSG00000248511	7mer-m8	TargetScan
miR-7161-3p	ENSG00000251201	7mer-m8	TargetScan
miR-7161-3p	ENSG00000256087	7mer-m8	TargetScan
miR-7161-3p	ENSG00000259332	7mer-m8	TargetScan
miR-7161-3p	ENSG00000264364	7mer-m8	TargetScan
miR-7161-3p	ENSG00000267618	7mer-m8	TargetScan
miR-1244	ENSG00000140526	Validated	miRTarBase
miR-1244	ENSG00000177076	Validated	miRTarBase
miR-1244	ENSG00000198074	Validated	miRTarBase
miR-1244	ENSG00000149925	Validated	miRTarBase
miR-1244	ENSG00000171681	Validated	miRTarBase
miR-1244	ENSG00000166148	Validated	miRTarBase
miR-1244	ENSG00000100211	Validated	miRTarBase
miR-1244	ENSG00000170558	Validated	miRTarBase
miR-1244	ENSG00000059758	Validated	miRTarBase
miR-1244	ENSG00000138180	Validated	miRTarBase
miR-1244	ENSG00000205423	Validated	miRTarBase
miR-1244	ENSG00000113300	Validated	miRTarBase
miR-1244	ENSG00000113712	Validated	miRTarBase

miR-1244	ENSG00000175183	Validated	miRTarBase
miR-1244	ENSG00000163257	Validated	miRTarBase
miR-1244	ENSG00000156136	Validated	miRTarBase
miR-1244	ENSG00000119661	Validated	miRTarBase
miR-1244	ENSG00000142634	Validated	miRTarBase
miR-1244	ENSG00000140285	Validated	miRTarBase
miR-1244	ENSG00000115339	Validated	miRTarBase
miR-1244	ENSG00000149948	Validated	miRTarBase
miR-1244	ENSG00000230989	Validated	miRTarBase
miR-1244	ENSG00000080824	Validated	miRTarBase
miR-1244	ENSG00000078596	Validated	miRTarBase
miR-1244	ENSG00000183340	Validated	miRTarBase
miR-1244	ENSG00000102753	Validated	miRTarBase
miR-1244	ENSG00000189308	Validated	miRTarBase
miR-1244	ENSG00000162441	Validated	miRTarBase
miR-1244	ENSG00000100030	Validated	miRTarBase
miR-1244	ENSG00000152939	Validated	miRTarBase
miR-1244	ENSG00000131844	Validated	miRTarBase
miR-1244	ENSG00000225526	Validated	miRTarBase
miR-1244	ENSG00000160877	Validated	miRTarBase
miR-1244	ENSG00000136352	Validated	miRTarBase
miR-1244	ENSG00000108256	Validated	miRTarBase
miR-1244	ENSG00000113569	Validated	miRTarBase
miR-1244	ENSG00000164902	Validated	miRTarBase
miR-1244	ENSG00000141682	Validated	miRTarBase
miR-1244	ENSG00000084733	Validated	miRTarBase
miR-1244	ENSG00000137710	Validated	miRTarBase
miR-1244	ENSG00000130066	Validated	miRTarBase
miR-1244	ENSG00000162241	Validated	miRTarBase
miR-1244	ENSG00000156222	Validated	miRTarBase
miR-1244	ENSG00000004939	Validated	miRTarBase

miR-1244	ENSG00000101665	Validated	miRTarBase
miR-1244	ENSG00000124380	Validated	miRTarBase
miR-1244	ENSG00000188612	Validated	miRTarBase
miR-1244	ENSG00000160551	Validated	miRTarBase
miR-1244	ENSG00000225366	Validated	miRTarBase
miR-1244	ENSG00000117500	Validated	miRTarBase
miR-1244	ENSG00000164180	Validated	miRTarBase
miR-1244	TMEM35	Validated	miRTarBase
miR-1244	ENSG00000126950	Validated	miRTarBase
miR-1244	ENSG00000109654	Validated	miRTarBase
miR-1244	ENSG00000197323	Validated	miRTarBase
miR-1244	ENSG00000146833	Validated	miRTarBase
miR-1244	ENSG00000213186	Validated	miRTarBase
miR-1244	ENSG00000156467	Validated	miRTarBase
miR-1244	ENSG00000243543	Validated	miRTarBase
miR-1244	ENSG00000149658	Validated	miRTarBase
miR-1244	ENSG00000146858	Validated	miRTarBase
miR-1244	ENSG00000136866	Validated	miRTarBase
miR-3654	ENSG00000134108	Validated	miRTarBase
miR-3654	CDR1-AS	Validated	miRTarBase
miR-3654	ENSG00000198354	Validated	miRTarBase
miR-3654	ENSG00000110367	Validated	miRTarBase
miR-3654	ENSG00000113657	Validated	miRTarBase
miR-3654	ENSG00000173674	Validated	miRTarBase
miR-3654	ENSG00000139641	Validated	miRTarBase
miR-3654	ENSG00000117868	Validated	miRTarBase
miR-3654	ENSG00000185442	Validated	miRTarBase
miR-3654	ENSG00000096060	Validated	miRTarBase
miR-3654	ENSG00000111816	Validated	miRTarBase
miR-3654	ENSG00000166225	Validated	miRTarBase
miR-3654	ENSG00000185477	Validated	miRTarBase

miR-3654	H2AFZ	Validated	miRTarBase
miR-3654	ENSG00000110422	Validated	miRTarBase
miR-3654	ENSG00000182952	Validated	miRTarBase
miR-3654	ENSG00000198700	Validated	miRTarBase
miR-3654	ENSG00000173120	Validated	miRTarBase
miR-3654	ENSG00000171435	Validated	miRTarBase
miR-3654	ENSG00000171421	Validated	miRTarBase
miR-3654	ENSG00000143436	Validated	miRTarBase
miR-3654	ENSG00000120458	Validated	miRTarBase
miR-3654	ENSG00000146938	Validated	miRTarBase
miR-3654	ENSG00000105287	Validated	miRTarBase
miR-3654	ENSG00000187514	Validated	miRTarBase
miR-3654	ENSG00000119383	Validated	miRTarBase
miR-3654	ENSG00000127314	Validated	miRTarBase
miR-3654	ENSG00000203727	Validated	miRTarBase
miR-3654	ENSG00000138593	Validated	miRTarBase
miR-3654	ENSG00000149212	Validated	miRTarBase
miR-3654	ENSG00000213064	Validated	miRTarBase
miR-3654	ENSG00000177058	Validated	miRTarBase
miR-3654	ENSG00000148296	Validated	miRTarBase
miR-3654	ENSG00000198792	Validated	miRTarBase
miR-3654	ENSG00000100354	Validated	miRTarBase
miR-3654	ENSG00000173218	Validated	miRTarBase
miR-4426	ENSG00000143401	Validated	miRTarBase
miR-4426	ENSG00000075884	Validated	miRTarBase
miR-4426	ENSG00000150347	Validated	miRTarBase
miR-4426	ENSG00000198363	Validated	miRTarBase
miR-4426	ENSG00000069399	Validated	miRTarBase
miR-4426	C17orf105	Validated	miRTarBase
miR-4426	ENSG00000105072	Validated	miRTarBase
miR-4426	C9orf170	Validated	miRTarBase

miR-4426	ENSG00000084774	Validated	miRTarBase
miR-4426	ENSG00000090061	Validated	miRTarBase
miR-4426	ENSG00000105810	Validated	miRTarBase
miR-4426	ENSG00000221869	Validated	miRTarBase
miR-4426	ENSG00000135837	Validated	miRTarBase
miR-4426	ENSG00000183196	Validated	miRTarBase
miR-4426	ENSG00000069493	Validated	miRTarBase
miR-4426	ENSG00000062485	Validated	miRTarBase
miR-4426	ENSG00000169429	Validated	miRTarBase
miR-4426	ENSG00000198563	Validated	miRTarBase
miR-4426	ENSG00000069345	Validated	miRTarBase
miR-4426	ENSG00000147251	Validated	miRTarBase
miR-4426	ENSG00000173674	Validated	miRTarBase
miR-4426	ENSG00000180185	Validated	miRTarBase
miR-4426	ENSG00000035141	Validated	miRTarBase
miR-4426	ENSG00000161040	Validated	miRTarBase
miR-4426	ENSG00000156804	Validated	miRTarBase
miR-4426	ENSG00000198855	Validated	miRTarBase
miR-4426	ENSG00000053108	Validated	miRTarBase
miR-4426	ENSG00000057608	Validated	miRTarBase
miR-4426	ENSG00000092208	Validated	miRTarBase
miR-4426	ENSG00000178295	Validated	miRTarBase
miR-4426	ENSG00000168827	Validated	miRTarBase
miR-4426	ENSG00000172380	Validated	miRTarBase
miR-4426	H3F3B	Validated	miRTarBase
miR-4426	H3F3C	Validated	miRTarBase
miR-4426	ENSG00000185436	Validated	miRTarBase
miR-4426	ENSG00000177663	Validated	miRTarBase
miR-4426	ENSG00000148153	Validated	miRTarBase
miR-4426	ENSG00000055118	Validated	miRTarBase
miR-4426	KIAA1551	Validated	miRTarBase

miR-4426	ENSG00000135835	Validated	miRTarBase
miR-4426	ENSG00000272333	Validated	miRTarBase
miR-4426	ENSG00000170423	Validated	miRTarBase
miR-4426	ENSG00000212659	Validated	miRTarBase
miR-4426	ENSG00000050426	Validated	miRTarBase
miR-4426	ENSG00000145012	Validated	miRTarBase
miR-4426	ENSG00000137269	Validated	miRTarBase
miR-4426	ENSG00000003056	Validated	miRTarBase
miR-4426	ENSG00000069535	Validated	miRTarBase
miR-4426	ENSG00000118579	Validated	miRTarBase
miR-4426	ENSG00000089693	Validated	miRTarBase
miR-4426	ENSG00000070444	Validated	miRTarBase
miR-4426	ENSG00000181610	Validated	miRTarBase
miR-4426	ENSG00000136997	Validated	miRTarBase
miR-4426	ENSG00000104825	Validated	miRTarBase
miR-4426	ENSG00000142405	Validated	miRTarBase
miR-4426	ENSG00000069275	Validated	miRTarBase
miR-4426	ENSG00000244171	Validated	miRTarBase
miR-4426	ENSG00000171408	Validated	miRTarBase
miR-4426	ENSG00000120509	Validated	miRTarBase
miR-4426	ENSG00000186951	Validated	miRTarBase
miR-4426	ENSG00000168938	Validated	miRTarBase
miR-4426	ENSG00000171016	Validated	miRTarBase
miR-4426	ENSG00000119396	Validated	miRTarBase
miR-4426	ENSG00000187010	Validated	miRTarBase
miR-4426	ENSG00000085721	Validated	miRTarBase
miR-4426	ENSG00000115310	Validated	miRTarBase
miR-4426	ENSG00000099194	Validated	miRTarBase
miR-4426	ENSG00000153130	Validated	miRTarBase
miR-4426	ENSG00000136193	Validated	miRTarBase
miR-4426	ENSG00000140090	Validated	miRTarBase

miR-4426	ENSG00000114120	Validated	miRTarBase
miR-4426	ENSG00000157106	Validated	miRTarBase
miR-4426	ENSG00000164056	Validated	miRTarBase
miR-4426	ENSG00000144867	Validated	miRTarBase
miR-4426	SSFA2	Validated	miRTarBase
miR-4426	ENSG00000178691	Validated	miRTarBase
miR-4426	ENSG00000136463	Validated	miRTarBase
miR-4426	ENSG00000109680	Validated	miRTarBase
miR-4426	ENSG00000102265	Validated	miRTarBase
miR-4426	ENSG00000142046	Validated	miRTarBase
miR-4426	ENSG00000083312	Validated	miRTarBase
miR-4426	ENSG00000173334	Validated	miRTarBase
miR-4426	ENSG00000204613	Validated	miRTarBase
miR-4426	ENSG00000177238	Validated	miRTarBase
miR-4426	ENSG00000165832	Validated	miRTarBase
miR-4426	ENSG00000162222	Validated	miRTarBase
miR-4426	ENSG00000137941	Validated	miRTarBase
miR-4426	ENSG00000265972	Validated	miRTarBase
miR-4426	ENSG00000103275	Validated	miRTarBase
miR-4426	ENSG00000166483	Validated	miRTarBase
miR-4426	ENSG00000249947	Validated	miRTarBase
miR-4426	ENSG00000175155	Validated	miRTarBase
miR-4426	ENSG00000177485	Validated	miRTarBase
miR-4426	ENSG00000164631	Validated	miRTarBase
miR-4426	ENSG00000186026	Validated	miRTarBase
miR-4426	ENSG00000197937	Validated	miRTarBase
miR-4426	ENSG00000121406	Validated	miRTarBase
miR-4426	ENSG00000245680	Validated	miRTarBase
miR-4426	ENSG00000182141	Validated	miRTarBase
miR-4444	ENSG00000178033	Validated	miRTarBase
miR-4444	ENSG00000174469	Validated	miRTarBase

miR-4444	ENSG00000142634	Validated	miRTarBase
miR-4444	FAM26E	Validated	miRTarBase
miR-4444	ENSG00000143891	Validated	miRTarBase
miR-4444	ENSG00000170837	Validated	miRTarBase
miR-4444	ENSG00000135486	Validated	miRTarBase
miR-4444	ENSG00000139675	Validated	miRTarBase
miR-4444	ENSG00000143603	Validated	miRTarBase
miR-4444	ENSG00000176619	Validated	miRTarBase
miR-4444	ENSG00000197879	Validated	miRTarBase
miR-4444	ENSG00000179094	Validated	miRTarBase
miR-4444	ENSG00000110841	Validated	miRTarBase
miR-4444	ENSG00000146733	Validated	miRTarBase
miR-4444	ENSG00000116514	Validated	miRTarBase
miR-4444	ENSG00000101236	Validated	miRTarBase
miR-4444	ENSG00000105640	Validated	miRTarBase
miR-4444	ENSG00000113739	Validated	miRTarBase
miR-4468	ENSG00000004455	Validated	miRTarBase
miR-4468	ENSG00000006534	Validated	miRTarBase
miR-4468	ENSG00000149925	Validated	miRTarBase
miR-4468	ENSG00000172339	Validated	miRTarBase
miR-4468	ENSG00000113108	Validated	miRTarBase
miR-4468	ENSG00000183111	Validated	miRTarBase
miR-4468	ENSG00000161944	Validated	miRTarBase
miR-4468	ENSG00000126775	Validated	miRTarBase
miR-4468	ENSG00000132932	Validated	miRTarBase
miR-4468	ENSG00000130270	Validated	miRTarBase
miR-4468	ENSG00000124788	Validated	miRTarBase
miR-4468	ENSG00000124243	Validated	miRTarBase
miR-4468	ENSG00000171552	Validated	miRTarBase
miR-4468	ENSG00000156983	Validated	miRTarBase
miR-4468	ENSG00000159388	Validated	miRTarBase

miR-4468	C11orf84	Validated	miRTarBase
miR-4468	ENSG00000156411	Validated	miRTarBase
miR-4468	ENSG00000140688	Validated	miRTarBase
miR-4468	ENSG00000179218	Validated	miRTarBase
miR-4468	ENSG00000130559	Validated	miRTarBase
miR-4468	ENSG00000077549	Validated	miRTarBase
miR-4468	ENSG00000105974	Validated	miRTarBase
miR-4468	ENSG00000170312	Validated	miRTarBase
miR-4468	ENSG00000100162	Validated	miRTarBase
miR-4468	ENSG00000255112	Validated	miRTarBase
miR-4468	ENSG00000101421	Validated	miRTarBase
miR-4468	ENSG00000143771	Validated	miRTarBase
miR-4468	ENSG00000108821	Validated	miRTarBase
miR-4468	ENSG00000241258	Validated	miRTarBase
miR-4468	ENSG00000180432	Validated	miRTarBase
miR-4468	ENSG00000166847	Validated	miRTarBase
miR-4468	ENSG00000185000	Validated	miRTarBase
miR-4468	ENSG00000005100	Validated	miRTarBase
miR-4468	ENSG00000007174	Validated	miRTarBase
miR-4468	ENSG00000147251	Validated	miRTarBase
miR-4468	ENSG00000276023	Validated	miRTarBase
miR-4468	ENSG00000143479	Validated	miRTarBase
miR-4468	ENSG00000169016	Validated	miRTarBase
miR-4468	ENSG00000143590	Validated	miRTarBase
miR-4468	ENSG00000024422	Validated	miRTarBase
miR-4468	ENSG00000158711	Validated	miRTarBase
miR-4468	ENSG00000170571	Validated	miRTarBase
miR-4468	FAM127A	Validated	miRTarBase
miR-4468	FAM46A	Validated	miRTarBase
miR-4468	ENSG00000108306	Validated	miRTarBase
miR-4468	ENSG00000119616	Validated	miRTarBase

miR-4468	ENSG00000161791	Validated	miRTarBase
miR-4468	ENSG00000164930	Validated	miRTarBase
miR-4468	ENSG00000105325	Validated	miRTarBase
miR-4468	ENSG00000138757	Validated	miRTarBase
miR-4468	ENSG00000141349	Validated	miRTarBase
miR-4468	ENSG00000261609	Validated	miRTarBase
miR-4468	ENSG00000143614	Validated	miRTarBase
miR-4468	ENSG00000203879	Validated	miRTarBase
miR-4468	ENSG00000175265	Validated	miRTarBase
miR-4468	ENSG00000215252	Validated	miRTarBase
miR-4468	ENSG00000173905	Validated	miRTarBase
miR-4468	ENSG00000013588	Validated	miRTarBase
miR-4468	ENSG00000164107	Validated	miRTarBase
miR-4468	ENSG00000214367	Validated	miRTarBase
miR-4468	ENSG00000105997	Validated	miRTarBase
miR-4468	ENSG00000166598	Validated	miRTarBase
miR-4468	ENSG00000181873	Validated	miRTarBase
miR-4468	ENSG00000164171	Validated	miRTarBase
miR-4468	ENSG00000101350	Validated	miRTarBase
miR-4468	ENSG00000174010	Validated	miRTarBase
miR-4468	ENSG00000111615	Validated	miRTarBase
miR-4468	ENSG00000150457	Validated	miRTarBase
miR-4468	LEPRE1	Validated	miRTarBase
miR-4468	ENSG00000077454	Validated	miRTarBase
miR-4468	ENSG00000131409	Validated	miRTarBase
miR-4468	ENSG00000161036	Validated	miRTarBase
miR-4468	ENSG00000108848	Validated	miRTarBase
miR-4468	ENSG00000169641	Validated	miRTarBase
miR-4468	ENSG00000162889	Validated	miRTarBase
miR-4468	ENSG00000101367	Validated	miRTarBase
miR-4468	ENSG00000172366	Validated	miRTarBase

miR-4468	ENSG00000050393	Validated	miRTarBase
miR-4468	ENSG00000110492	Validated	miRTarBase
miR-4468	ENSG00000169057	Validated	miRTarBase
miR-4468	ENSG00000135953	Validated	miRTarBase
miR-4468	ENSG00000108788	Validated	miRTarBase
miR-4468	ENSG00000170903	Validated	miRTarBase
miR-4468	ENSG00000138347	Validated	miRTarBase
miR-4468	ENSG00000130414	Validated	miRTarBase
miR-4468	ENSG00000181163	Validated	miRTarBase
miR-4468	ENSG00000213281	Validated	miRTarBase
miR-4468	ENSG00000105953	Validated	miRTarBase
miR-4468	ENSG00000117385	Validated	miRTarBase
miR-4468	ENSG00000182749	Validated	miRTarBase
miR-4468	ENSG00000138964	Validated	miRTarBase
miR-4468	ENSG00000164951	Validated	miRTarBase
miR-4468	ENSG00000179094	Validated	miRTarBase
miR-4468	ENSG00000135365	Validated	miRTarBase
miR-4468	ENSG00000081277	Validated	miRTarBase
miR-4468	ENSG00000137055	Validated	miRTarBase
miR-4468	ENSG00000126003	Validated	miRTarBase
miR-4468	ENSG00000143850	Validated	miRTarBase
miR-4468	ENSG00000115762	Validated	miRTarBase
miR-4468	ENSG00000164087	Validated	miRTarBase
miR-4468	ENSG00000137168	Validated	miRTarBase
miR-4468	ENSG00000163637	Validated	miRTarBase
miR-4468	ENSG00000205352	Validated	miRTarBase
miR-4468	ENSG00000288701	Validated	miRTarBase
miR-4468	ENSG00000112245	Validated	miRTarBase
miR-4468	ENSG00000183010	Validated	miRTarBase
miR-4468	ENSG00000112210	Validated	miRTarBase
miR-4468	ENSG00000104388	Validated	miRTarBase

miR-4468	ENSG00000108774	Validated	miRTarBase
miR-4468	ENSG00000161800	Validated	miRTarBase
miR-4468	ENSG00000102317	Validated	miRTarBase
miR-4468	ENSG00000168214	Validated	miRTarBase
miR-4468	ENSG00000079313	Validated	miRTarBase
miR-4468	ENSG00000138942	Validated	miRTarBase
miR-4468	ENSG00000114391	Validated	miRTarBase
miR-4468	ENSG00000197756	Validated	miRTarBase
miR-4468	ENSG00000233927	Validated	miRTarBase
miR-4468	ENSG00000134590	Validated	miRTarBase
miR-4468	ENSG00000099194	Validated	miRTarBase
miR-4468	ENSG00000058262	Validated	miRTarBase
miR-4468	ENSG00000179833	Validated	miRTarBase
miR-4468	ENSG00000104611	Validated	miRTarBase
miR-4468	ENSG00000176209	Validated	miRTarBase
miR-4468	ENSG00000102172	Validated	miRTarBase
miR-4468	ENSG00000159674	Validated	miRTarBase
miR-4468	ENSG00000167778	Validated	miRTarBase
miR-4468	ENSG00000115525	Validated	miRTarBase
miR-4468	ENSG00000079950	Validated	miRTarBase
miR-4468	ENSG00000136560	Validated	miRTarBase
miR-4468	ENSG00000107021	Validated	miRTarBase
miR-4468	ENSG00000007866	Validated	miRTarBase
miR-4468	ENSG00000115112	Validated	miRTarBase
miR-4468	ENSG00000072274	Validated	miRTarBase
miR-4468	ENSG00000169908	Validated	miRTarBase
miR-4468	ENSG00000184497	Validated	miRTarBase
miR-4468	ENSG00000109079	Validated	miRTarBase
miR-4468	ENSG00000083312	Validated	miRTarBase
miR-4468	ENSG00000090905	Validated	miRTarBase
miR-4468	ENSG00000111669	Validated	miRTarBase

miR-4468	ENSG00000160218	Validated	miRTarBase
miR-4468	ENSG00000206557	Validated	miRTarBase
miR-4468	ENSG00000136925	Validated	miRTarBase
miR-4468	ENSG00000136295	Validated	miRTarBase
miR-4468	ENSG00000173960	Validated	miRTarBase
miR-4468	ENSG00000125753	Validated	miRTarBase
miR-4468	ENSG00000132471	Validated	miRTarBase
miR-4468	ENSG00000109046	Validated	miRTarBase
miR-4468	ENSG00000206579	Validated	miRTarBase
miR-4468	ENSG00000134684	Validated	miRTarBase
miR-4468	ENSG00000108953	Validated	miRTarBase
miR-4468	ENSG00000103994	Validated	miRTarBase
miR-4468	ENSG00000196247	Validated	miRTarBase
miR-4468	ENSG00000083844	Validated	miRTarBase
miR-4468	ENSG00000245680	Validated	miRTarBase
miR-4468	ENSG00000204524	Validated	miRTarBase
miR-4468	ENSG00000176723	Validated	miRTarBase
miR-4788	ENSG00000008441	Validated	miRTarBase
miR-4788	ENSG00000144840	Validated	miRTarBase
miR-4788	ENSG00000142512	Validated	miRTarBase
miR-4788	ENSG00000152332	Validated	miRTarBase
miR-4788	ENSG00000171466	Validated	miRTarBase
miR-492	ENSG00000174574	Validated	miRTarBase
miR-492	ENSG00000163631	Validated	miRTarBase
miR-492	ENSG00000136881	Validated	miRTarBase
miR-492	ENSG00000015475	Validated	miRTarBase
miR-492	ENSG00000172270	Validated	miRTarBase
miR-492	ENSG00000159388	Validated	miRTarBase
miR-492	ENSG00000154473	Validated	miRTarBase
miR-492	ENSG00000099804	Validated	miRTarBase
miR-492	ENSG00000147889	Validated	miRTarBase

miR-492	ENSG00000153879	Validated	miRTarBase
miR-492	ENSG00000130309	Validated	miRTarBase
miR-492	ENSG00000119004	Validated	miRTarBase
miR-492	ENSG00000070190	Validated	miRTarBase
miR-492	ENSG00000005100	Validated	miRTarBase
miR-492	ENSG00000165219	Validated	miRTarBase
miR-492	ENSG00000119125	Validated	miRTarBase
miR-492	ENSG00000101216	Validated	miRTarBase
miR-492	ENSG00000197451	Validated	miRTarBase
miR-492	ENSG00000203857	Validated	miRTarBase
miR-492	ENSG00000140443	Validated	miRTarBase
miR-492	ENSG00000203485	Validated	miRTarBase
miR-492	ENSG00000126934	Validated	miRTarBase
miR-492	ENSG00000168906	Validated	miRTarBase
miR-492	ENSG00000099326	Validated	miRTarBase
miR-492	ENSG00000165650	Validated	miRTarBase
miR-492	ENSG00000105063	Validated	miRTarBase
miR-492	ENSG00000171862	Validated	miRTarBase
miR-492	ENSG00000104524	Validated	miRTarBase
miR-492	PYCRL	Validated	miRTarBase
miR-492	ENSG00000179051	Validated	miRTarBase
miR-492	ENSG00000168411	Validated	miRTarBase
miR-492	ENSG00000181031	Validated	miRTarBase
miR-492	ENSG00000177570	Validated	miRTarBase
miR-492	ENSG00000186193	Validated	miRTarBase
miR-492	ENSG00000017483	Validated	miRTarBase
miR-492	ENSG00000064199	Validated	miRTarBase
miR-492	ENSG00000073849	Validated	miRTarBase
miR-492	ENSG00000148296	Validated	miRTarBase
miR-492	ENSG00000213977	Validated	miRTarBase
miR-492	ENSG00000118526	Validated	miRTarBase

miR-492	ENSG00000163762	Validated	miRTarBase
miR-492	ENSG00000198326	Validated	miRTarBase
miR-492	TMEM246	Validated	miRTarBase
miR-492	ENSG00000157741	Validated	miRTarBase
miR-492	ENSG00000141580	Validated	miRTarBase
miR-492	ENSG00000154767	Validated	miRTarBase
miR-492	ENSG00000160445	Validated	miRTarBase
miR-7161-3p	ENSG00000072364	Validated	miRTarBase
miR-7161-3p	ENSG00000172339	Validated	miRTarBase
miR-7161-3p	ENSG00000144233	Validated	miRTarBase
miR-7161-3p	ENSG00000163297	Validated	miRTarBase
miR-7161-3p	ENSG00000214944	Validated	miRTarBase
miR-7161-3p	ENSG00000168488	Validated	miRTarBase
miR-7161-3p	ENSG00000258643	Validated	miRTarBase
miR-7161-3p	ENSG00000069399	Validated	miRTarBase
miR-7161-3p	ENSG00000182985	Validated	miRTarBase
miR-7161-3p	ENSG00000127022	Validated	miRTarBase
miR-7161-3p	ENSG00000143418	Validated	miRTarBase
miR-7161-3p	ENSG00000168763	Validated	miRTarBase
miR-7161-3p	ENSG00000172301	Validated	miRTarBase
miR-7161-3p	ENSG00000124795	Validated	miRTarBase
miR-7161-3p	ENSG00000255302	Validated	miRTarBase
miR-7161-3p	ENSG00000197594	Validated	miRTarBase
miR-7161-3p	ENSG00000152380	Validated	miRTarBase
miR-7161-3p	ENSG00000164930	Validated	miRTarBase
miR-7161-3p	ENSG00000107937	Validated	miRTarBase
miR-7161-3p	ENSG00000140443	Validated	miRTarBase
miR-7161-3p	ENSG00000142856	Validated	miRTarBase
miR-7161-3p	ENSG00000164626	Validated	miRTarBase
miR-7161-3p	ENSG00000174010	Validated	miRTarBase
miR-7161-3p	ENSG00000151224	Validated	miRTarBase

miR-7161-3p	ENSG00000145388	Validated	miRTarBase
miR-7161-3p	ENSG00000144821	Validated	miRTarBase
miR-7161-3p	ENSG00000144426	Validated	miRTarBase
miR-7161-3p	ENSG00000112992	Validated	miRTarBase
miR-7161-3p	ENSG00000147140	Validated	miRTarBase
miR-7161-3p	ENSG00000167005	Validated	miRTarBase
miR-7161-3p	ENSG00000114054	Validated	miRTarBase
miR-7161-3p	ENSG00000124225	Validated	miRTarBase
miR-7161-3p	ENSG00000187537	Validated	miRTarBase
miR-7161-3p	ENSG00000222036	Validated	miRTarBase
miR-7161-3p	ENSG00000152061	Validated	miRTarBase
miR-7161-3p	ENSG00000129824	Validated	miRTarBase
miR-7161-3p	ENSG00000198301	Validated	miRTarBase
miR-7161-3p	ENSG00000070214	Validated	miRTarBase
miR-7161-3p	ENSG00000113810	Validated	miRTarBase
miR-7161-3p	ENSG00000099940	Validated	miRTarBase
miR-7161-3p	ENSG00000269404	Validated	miRTarBase
miR-7161-3p	ENSG00000172296	Validated	miRTarBase
miR-7161-3p	ENSG00000161011	Validated	miRTarBase
miR-7161-3p	ENSG00000198252	Validated	miRTarBase
miR-7161-3p	ENSG00000204634	Validated	miRTarBase
miR-7161-3p	ENSG00000042832	Validated	miRTarBase
miR-7161-3p	ENSG00000163714	Validated	miRTarBase
miR-7161-3p	ENSG00000223501	Validated	miRTarBase
miR-7161-3p	ENSG00000180667	Validated	miRTarBase
miR-7161-3p	ENSG00000105497	Validated	miRTarBase
miR-7161-3p	ENSG00000197714	Validated	miRTarBase
miR-7161-5p	ENSG00000123130	Validated	miRTarBase
miR-7161-5p	ENSG00000197894	Validated	miRTarBase
miR-7161-5p	ENSG00000172493	Validated	miRTarBase
miR-7161-5p	ENSG00000196510	Validated	miRTarBase

miR-7161-5p	ENSG00000196975	Validated	miRTarBase
miR-7161-5p	ENSG00000134884	Validated	miRTarBase
miR-7161-5p	ENSG00000075884	Validated	miRTarBase
miR-7161-5p	ENSG00000170632	Validated	miRTarBase
miR-7161-5p	ENSG00000155097	Validated	miRTarBase
miR-7161-5p	ENSG00000156735	Validated	miRTarBase
miR-7161-5p	ENSG00000082153	Validated	miRTarBase
miR-7161-5p	ENSG00000164008	Validated	miRTarBase
miR-7161-5p	ENSG00000165995	Validated	miRTarBase
miR-7161-5p	ENSG00000133773	Validated	miRTarBase
miR-7161-5p	ENSG00000197622	Validated	miRTarBase
miR-7161-5p	ENSG00000031691	Validated	miRTarBase
miR-7161-5p	ENSG00000171316	Validated	miRTarBase
miR-7161-5p	ENSG00000163347	Validated	miRTarBase
miR-7161-5p	ENSG00000157224	Validated	miRTarBase
miR-7161-5p	ENSG00000080822	Validated	miRTarBase
miR-7161-5p	ENSG00000168763	Validated	miRTarBase
miR-7161-5p	ENSG00000174469	Validated	miRTarBase
miR-7161-5p	ENSG00000119878	Validated	miRTarBase
miR-7161-5p	ENSG00000035664	Validated	miRTarBase
miR-7161-5p	ENSG00000077232	Validated	miRTarBase
miR-7161-5p	ENSG00000141543	Validated	miRTarBase
miR-7161-5p	ENSG00000082397	Validated	miRTarBase
miR-7161-5p	ENSG00000180263	Validated	miRTarBase
miR-7161-5p	ENSG00000154803	Validated	miRTarBase
miR-7161-5p	ENSG00000145907	Validated	miRTarBase
miR-7161-5p	ENSG00000198380	Validated	miRTarBase
miR-7161-5p	ENSG00000130119	Validated	miRTarBase
miR-7161-5p	ENSG00000278662	Validated	miRTarBase
miR-7161-5p	ENSG00000184206	Validated	miRTarBase
miR-7161-5p	ENSG00000261794	Validated	miRTarBase

miR-7161-5p	ENSG00000179938	Validated	miRTarBase
miR-7161-5p	ENSG00000188626	Validated	miRTarBase
miR-7161-5p	ENSG00000183098	Validated	miRTarBase
miR-7161-5p	ENSG00000143321	Validated	miRTarBase
miR-7161-5p	HIST2H2BE	Validated	miRTarBase
miR-7161-5p	ENSG00000147421	Validated	miRTarBase
miR-7161-5p	ENSG00000189403	Validated	miRTarBase
miR-7161-5p	ENSG00000140443	Validated	miRTarBase
miR-7161-5p	ENSG00000174564	Validated	miRTarBase
miR-7161-5p	ENSG00000149503	Validated	miRTarBase
miR-7161-5p	ENSG00000102781	Validated	miRTarBase
miR-7161-5p	KIAA1456	Validated	miRTarBase
miR-7161-5p	ENSG00000170759	Validated	miRTarBase
miR-7161-5p	ENSG00000162413	Validated	miRTarBase
miR-7161-5p	ENSG00000196233	Validated	miRTarBase
miR-7161-5p	ENSG00000166816	Validated	miRTarBase
miR-7161-5p	ENSG00000186960	Validated	miRTarBase
miR-7161-5p	ENSG00000175556	Validated	miRTarBase
miR-7161-5p	ENSG00000163428	Validated	miRTarBase
miR-7161-5p	ENSG00000198162	Validated	miRTarBase
miR-7161-5p	ENSG00000069956	Validated	miRTarBase
miR-7161-5p	ENSG00000169446	Validated	miRTarBase
miR-7161-5p	ENSG00000169184	Validated	miRTarBase
miR-7161-5p	ENSG00000130675	Validated	miRTarBase
miR-7161-5p	ENSG00000116062	Validated	miRTarBase
miR-7161-5p	ENSG00000082641	Validated	miRTarBase
miR-7161-5p	ENSG00000188158	Validated	miRTarBase
miR-7161-5p	ENSG00000213281	Validated	miRTarBase
miR-7161-5p	ENSG00000069275	Validated	miRTarBase
miR-7161-5p	ENSG00000276045	Validated	miRTarBase
miR-7161-5p	ENSG00000100266	Validated	miRTarBase

miR-7161-5p	ENSG00000165494	Validated	miRTarBase
miR-7161-5p	ENSG00000132646	Validated	miRTarBase
miR-7161-5p	ENSG00000175895	Validated	miRTarBase
miR-7161-5p	ENSG00000090924	Validated	miRTarBase
miR-7161-5p	ENSG00000113575	Validated	miRTarBase
miR-7161-5p	ENSG00000117707	Validated	miRTarBase
miR-7161-5p	ENSG00000112245	Validated	miRTarBase
miR-7161-5p	ENSG00000153201	Validated	miRTarBase
miR-7161-5p	ENSG00000079332	Validated	miRTarBase
miR-7161-5p	ENSG00000144285	Validated	miRTarBase
miR-7161-5p	ENSG00000119335	Validated	miRTarBase
miR-7161-5p	ENSG00000198879	Validated	miRTarBase
miR-7161-5p	ENSG00000213064	Validated	miRTarBase
miR-7161-5p	ENSG00000060140	Validated	miRTarBase
miR-7161-5p	ENSG00000176148	Validated	miRTarBase
miR-7161-5p	ENSG00000163513	Validated	miRTarBase
miR-7161-5p	ENSG00000174695	Validated	miRTarBase
miR-7161-5p	ENSG00000226479	Validated	miRTarBase
miR-7161-5p	ENSG00000135048	Validated	miRTarBase
miR-7161-5p	ENSG00000119777	Validated	miRTarBase
miR-7161-5p	ENSG00000187824	Validated	miRTarBase
miR-7161-5p	ENSG00000188167	Validated	miRTarBase
miR-7161-5p	ENSG00000169905	Validated	miRTarBase
miR-7161-5p	ENSG00000132256	Validated	miRTarBase
miR-7161-5p	ENSG00000165699	Validated	miRTarBase
miR-7161-5p	ENSG00000155158	Validated	miRTarBase
miR-7161-5p	ENSG00000104343	Validated	miRTarBase
miR-7161-5p	ENSG00000173610	Validated	miRTarBase
miR-7161-5p	ENSG00000271271	Validated	miRTarBase
miR-7161-5p	ENSG00000173950	Validated	miRTarBase
miR-7161-5p	ENSG00000166793	Validated	miRTarBase

miR-7161-5p	ENSG00000196449	Validated	miRTarBase
miR-7161-5p	ENSG00000198839	Validated	miRTarBase
miR-7161-5p	ENSG00000168916	Validated	miRTarBase
miR-7161-5p	ENSG00000181450	Validated	miRTarBase
miR-7161-5p	ENSG00000196946	Validated	miRTarBase
miR-7161-5p	ENSG00000197782	Validated	miRTarBase
miR-7161-5p	ENSG00000124201	Validated	miRTarBase
miR-572	ENSG00000149311	Validated	miRTarBase
miR-572	ENSG00000124762	Validated	miRTarBase
miR-572	ENSG00000136425	Validated	miRTarBase
miR-572	ENSG00000114737	Validated	miRTarBase
miR-572	ENSG00000153933	Validated	miRTarBase
miR-572	ENSG00000167536	Validated	miRTarBase
miR-572	ENSG00000187474	Validated	miRTarBase
miR-572	ENSG00000119912	Validated	miRTarBase
miR-572	ENSG00000156959	Validated	miRTarBase
miR-572	ENSG00000063322	Validated	miRTarBase
miR-572	ENSG00000178921	Validated	miRTarBase
miR-572	ENSG00000166794	Validated	miRTarBase
miR-572	ENSG00000163082	Validated	miRTarBase
miR-572	ENSG00000177045	Validated	miRTarBase
miR-572	ENSG00000196182	Validated	miRTarBase
miR-572	ENSG00000132604	Validated	miRTarBase
miR-572	ENSG00000178233	Validated	miRTarBase
miR-572	TRP-AGG2-4	Validated	miRTarBase
miR-572	ENSG00000166402	Validated	miRTarBase
miR-572	ENSG00000154764	Validated	miRTarBase
miR-572	ENSG00000106261	Validated	miRTarBase
miR-572	ENSG00000173875	Validated	miRTarBase
miR-622	ENSG00000115977	Validated	miRTarBase
miR-622	ENSG00000100997	Validated	miRTarBase

miR-622	ENSG00000149925	Validated	miRTarBase
miR-622	ENSG00000064999	Validated	miRTarBase
miR-622	ENSG00000166747	Validated	miRTarBase
miR-622	ENSG00000178878	Validated	miRTarBase
miR-622	ENSG00000134108	Validated	miRTarBase
miR-622	ENSG00000161664	Validated	miRTarBase
miR-622	ENSG00000177556	Validated	miRTarBase
miR-622	ENSG00000183778	Validated	miRTarBase
miR-622	ENSG00000151917	Validated	miRTarBase
miR-622	ENSG00000182919	Validated	miRTarBase
miR-622	C19orf52	Validated	miRTarBase
miR-622	C9orf3	Validated	miRTarBase
miR-622	ENSG00000152495	Validated	miRTarBase
miR-622	ENSG00000163492	Validated	miRTarBase
miR-622	ENSG00000110104	Validated	miRTarBase
miR-622	ENSG00000123374	Validated	miRTarBase
miR-622	ENSG00000101624	Validated	miRTarBase
miR-622	ENSG00000143786	Validated	miRTarBase
miR-622	ENSG00000099942	Validated	miRTarBase
miR-622	ENSG00000121966	Validated	miRTarBase
miR-622	ENSG00000172115	Validated	miRTarBase
miR-622	ENSG00000083799	Validated	miRTarBase
miR-622	ENSG00000142871	Validated	miRTarBase
miR-622	ENSG00000162946	Validated	miRTarBase
miR-622	ENSG00000176124	Validated	miRTarBase
miR-622	FAM127B	Validated	miRTarBase
miR-622	ENSG00000177150	Validated	miRTarBase
miR-622	ENSG00000156860	Validated	miRTarBase
miR-622	ENSG00000108306	Validated	miRTarBase
miR-622	ENSG00000197948	Validated	miRTarBase
miR-622	ENSG00000102678	Validated	miRTarBase

miR-622	ENSG00000166225	Validated	miRTarBase
miR-622	ENSG00000145907	Validated	miRTarBase
miR-622	ENSG00000146830	Validated	miRTarBase
miR-622	ENSG00000124767	Validated	miRTarBase
miR-622	ENSG00000142751	Validated	miRTarBase
miR-622	ENSG00000172432	Validated	miRTarBase
miR-622	ENSG00000249115	Validated	miRTarBase
miR-622	ENSG00000196917	Validated	miRTarBase
miR-622	ENSG00000051620	Validated	miRTarBase
miR-622	ENSG00000158373	Validated	miRTarBase
miR-622	ENSG00000285219	Validated	miRTarBase
miR-622	ENSG00000143603	Validated	miRTarBase
miR-622	ENSG00000179454	Validated	miRTarBase
miR-622	ENSG00000122550	Validated	miRTarBase
miR-622	ENSG00000133703	Validated	miRTarBase
miR-622	ENSG00000182816	Validated	miRTarBase
miR-622	ENSG00000058085	Validated	miRTarBase
miR-622	ENSG00000116212	Validated	miRTarBase
miR-622	ENSG00000162441	Validated	miRTarBase
miR-622	ENSG00000185090	Validated	miRTarBase
miR-622	ENSG00000159256	Validated	miRTarBase
miR-622	ENSG00000048544	Validated	miRTarBase
miR-622	MTL5	Validated	miRTarBase
miR-622	MTRNR2L7	Validated	miRTarBase
miR-622	ENSG00000136997	Validated	miRTarBase
miR-622	ENSG00000188211	Validated	miRTarBase
miR-622	ENSG00000160602	Validated	miRTarBase
miR-622	ENSG00000115761	Validated	miRTarBase
miR-622	ENSG00000108256	Validated	miRTarBase
miR-622	ENSG00000185585	Validated	miRTarBase
miR-622	ENSG00000157654	Validated	miRTarBase

miR-622	ENSG00000161217	Validated	miRTarBase
miR-622	ENSG00000112511	Validated	miRTarBase
miR-622	PLA2G16	Validated	miRTarBase
miR-622	ENSG00000011422	Validated	miRTarBase
miR-622	ENSG00000178385	Validated	miRTarBase
miR-622	ENSG00000141682	Validated	miRTarBase
miR-622	ENSG00000184203	Validated	miRTarBase
miR-622	ENSG00000119608	Validated	miRTarBase
miR-622	ENSG00000101213	Validated	miRTarBase
miR-622	ENSG00000112130	Validated	miRTarBase
miR-622	ENSG00000069667	Validated	miRTarBase
miR-622	ENSG00000178718	Validated	miRTarBase
miR-622	ENSG00000197728	Validated	miRTarBase
miR-622	ENSG00000100220	Validated	miRTarBase
miR-622	ENSG00000203950	Validated	miRTarBase
miR-622	ENSG00000103449	Validated	miRTarBase
miR-622	ENSG00000179134	Validated	miRTarBase
miR-622	ENSG00000116171	Validated	miRTarBase
miR-622	ENSG00000142864	Validated	miRTarBase
miR-622	ENSG00000172058	Validated	miRTarBase
miR-622	ENSG00000205572	Validated	miRTarBase
miR-622	ENSG00000132824	Validated	miRTarBase
miR-622	ENSG00000130766	Validated	miRTarBase
miR-622	ENSG00000149212	Validated	miRTarBase
miR-622	ENSG00000162461	Validated	miRTarBase
miR-622	ENSG00000176463	Validated	miRTarBase
miR-622	ENSG00000138600	Validated	miRTarBase
miR-622	ENSG00000136450	Validated	miRTarBase
miR-622	ENSG00000112081	Validated	miRTarBase
miR-622	ENSG00000148296	Validated	miRTarBase
miR-622	ENSG00000149256	Validated	miRTarBase

miR-622	ENSG00000132749	Validated	miRTarBase
miR-622	ENSG00000114126	Validated	miRTarBase
miR-622	ENSG00000174796	Validated	miRTarBase
miR-622	ENSG00000142444	Validated	miRTarBase
miR-622	ENSG00000169908	Validated	miRTarBase
miR-622	ENSG00000103534	Validated	miRTarBase
miR-622	ENSG00000215712	Validated	miRTarBase
miR-622	ENSG00000141510	Validated	miRTarBase
miR-622	ENSG00000108395	Validated	miRTarBase
miR-622	ENSG00000086712	Validated	miRTarBase
miR-622	ENSG00000118900	Validated	miRTarBase
miR-622	ENSG00000107731	Validated	miRTarBase
miR-622	ENSG00000062716	Validated	miRTarBase
miR-622	ENSG00000106299	Validated	miRTarBase
miR-622	ENSG00000179456	Validated	miRTarBase
miR-622	ENSG00000185670	Validated	miRTarBase
miR-622	ENSG00000005801	Validated	miRTarBase
miR-622	ENSG00000258405	Validated	miRTarBase
miR-622	ENSG00000223547	Validated	miRTarBase
