# Supplementary material for: Retro-miRs: novel and functional miRNAs originating from mRNA retrotransposition
Source: Mob DNA. 2023 Sep 8;14:12. doi: 10.1186/s13100-023-00301-w (PMC10486083; doi:10.1186/s13100-023-00301-w)
Supplement: Supplementary file 10 — Additional file 10: Table S9. Targets of the retro-miRs. [file 13100_2023_301_MOESM10_ESM.pdf]

Table S9. Targets of the retro-miRs

KEGG.URL	KEGG.pathway	n.genes	total.genes	perc.genes	fold.enrichment	FDR	genes
http://www.genome.jp/kegg-bin/show_pathway?hsa04710	Circadian rhythm	15	31	48.39%	2.26915	1.12E-02	FBXL3 CRY1 PER3 RORA FBXW11 PRKAB1 SKP1 CREB1 CRY2 PER2 CLOCK PRKAA2 BTRC PER1 CSNK1E
http://www.genome.jp/kegg-bin/show_pathway?hsa05219	Bladder cancer	17	41	41.46%	1.94446	2.41E-02	DAPK2 ARAF MAPK1 E2F1 E2F3 CDKN1A MAP2K2 KRAS MYC EGF TP53 EGFR CDKN2A BRAF CXCL8 DAPK1 NRAS
http://www.genome.jp/kegg-bin/show_pathway?hsa04520	Adherens junction	29	71	40.85%	1.91546	4.34E-03	VCL CTNNA1 RHOA SORBS1 MAPK1 CSNK2A1 MET WASL TGFBR1 SSX2IP PTPRB RAC2 WASF3 MAP3K7 IGF1R SMAD4 NECTIN4 ACP1 EGFR PARD3 TCF7L2 PTPRJ FER TCF7L1 TGFBR2 INSR NECTIN3 CTNNA3 CTNND1
http://www.genome.jp/kegg-bin/show_pathway?hsa04730	Long-term depression	24	60	40.00%	1.87583	1.12E-02	IGF1 GNAI3 ARAF GNAS NOS1 MAPK1 PPP2R1A PPP2CA GRIA2 ITPR2 MAP2K2 KRAS PPP2R1B IGF1R GNA12 PLCB3 GUCY1A2 GRM1 BRAF GUCY1A1 PLCB1 PLA2G4E NRAS LYN
http://www.genome.jp/kegg-bin/show_pathway?hsa05210	Colorectal cancer	34	86	39.53%	1.85402	2.91E-03	BAK1 MAPK9 PIK3CB RHOA ARAF GSK3B MAPK1 TGFBR1 MSH3 MSH6 PIK3CA CDKN1A MAP2K2 RAC2 CASP9 KRAS MYC EGF TP53 SMAD4 PMAIP1 EGFR TCF7L2 TCF7L1 APPL1 BRAF TGFA TGFBR2 FOS BCL2 CYCS SMAD2 JUN NRAS
http://www.genome.jp/kegg-bin/show_pathway?hsa05218	Melanoma	28	72	38.89%	1.82372	1.03E-02	IGF1 BAK1 PIK3CB FGF10 ARAF MAPK1 E2F1 FGF9 CDK6 MET E2F3 PIK3CA CDKN1A MAP2K2 KRAS FGF5 EGF FGF7 IGF1R TP53 PDGFC EGFR CDKN2A BRAF PDGFD FGF3 MITF NRAS
http://www.genome.jp/kegg-bin/show_pathway?hsa04917	Prolactin signaling pathway	26	70	37.14%	1.74184	1.72E-02	MAPK9 PIK3CB GSK3B ESR1 JAK2 MAPK1 MAPK14 PRLR CISH SOCS2 PIK3CA STAT5A MAP2K2 KRAS ELF5 LHCGR ESR2 TNFRSF11A SHC3 MAPK13 SLC2A2 STAT3 FOS SOCS6 SOCS4 NRAS
http://www.genome.jp/kegg-bin/show_pathway?hsa01524	Platinum drug resistance	27	73	36.99%	1.73450	1.58E-02	ERCC1 BID FAS BAK1 PIK3CB MGST2 MAPK1 XIAP MSH3 MSH6 PIK3CA ATP7B CDKN1A CASP9 GSTM5 GSTM3 PDPK1 TP53 PMAIP1 MGST3 CDKN2A ATM ATP7A POLH BCL2L1 BCL2 CYCS
http://www.genome.jp/kegg-bin/show_pathway?hsa04115	P53 signaling pathway	27	73	36.99%	1.73450	1.58E-02	BID IGF1 FAS RRM2B TP73 CD82 TNFRSF10A CDK6 SERPINE1 CCND3 CDKN1A CASP9 CCNG2 TP53 PMAIP1 IGFBP3 CDKN2A SESN3 ATM CHEK1 PPM1D BCL2L1 BCL2 RRM2 CYCS CCNE2 MDM4
http://www.genome.jp/kegg-bin/show_pathway?hsa05220	Chronic myeloid leukemia	28	76	36.84%	1.72774	1.52E-02	BAK1 PIK3CB ARAF ABL1 CRKL MAPK1 E2F1 CDK6 TGFBR1 CBL E2F3 PIK3CA CDKN1A STAT5A MAP2K2 KRAS MYC TP53 SMAD4 CDKN2A SHC3 BRAF CTBP1 TGFBR2 BCL2L1 CTBP2 HDAC2 NRAS
http://www.genome.jp/kegg-bin/show_pathway?hsa04141	Protein processing in endoplasmic reticulum	62	169	36.69%	1.72043	3.19E-04	BAK1 HSPA5 MAPK9 EIF2AK2 SEC61A1 SEC61A2 ATXN3 DNAJA2 UBE2D1 DERL2 LMAN1 DNAJC10 UBE2D4 SAR1A HSP90AA1 EIF2AK1 ERO1B PPP1R15A NSFL1C ERP29 XBP1 SEC23A TUSC3 UBE2D3 MAN1A1 BAG2 SKP1 SEC24A SSR3 FBXO2 ATF6 RPN2 HSPH1 ATXN3L SSR1 HSPA2 TRAF2 EDEM1 UBQLN1 CKAP4 UGGT1 PLAA SEC24B RNF185 MBTPS1 MARCHF6 DNAJB12 HSPA4L HSP90B1 PDIA3 LMAN2 BCL2 HSPA6 UBXN2A ERN1 CALR YOD1 NPLOC4 UBQLN2 ERO1A MAN1A2 SVIP
http://www.genome.jp/kegg-bin/show_pathway?hsa05213	Endometrial cancer	21	58	36.21%	1.69795	4.07E-02	BAK1 CTNNA1 PIK3CB ARAF GSK3B MAPK1 PIK3CA CDKN1A MAP2K2 CASP9 KRAS MYC EGF PDPK1 TP53 EGFR TCF7L2 TCF7L1 BRAF CTNNA3 NRAS
http://www.genome.jp/kegg-bin/show_pathway?hsa05221	Acute myeloid leukemia	24	67	35.82%	1.67985	3.25E-02	PIK3CB ARAF RUNX1T1 MAPK1 PIM2 ZBTB16 PPARD PIK3CA STAT5A MAP2K2 PER2 KRAS MYC DUSP6 PML CCNA2 TCF7L2 TCF7L1 KIT BRAF STAT3 JUP EIF4EBP1 NRAS
http://www.genome.jp/kegg-bin/show_pathway?hsa01522	Endocrine resistance	34	95	35.79%	1.67837	1.13E-02	IGF1 MAPK9 PIK3CB ARAF ADCY2 GNAS ESR1 MAPK1 JAG1 E2F1 MAPK14 E2F3 ADCY7 PIK3CA CDKN2C CDKN1A MAP2K2 KRAS NOTCH2 ESR2 IGF1R TP53 EGFR CDKN2A SHC3 MAPK13 BRAF ADCY1 FOS BCL2 JUN JAG2 SP1 NRAS
http://www.genome.jp/kegg-bin/show_pathway?hsa05212	Pancreatic cancer	27	76	35.53%	1.66603	2.51E-02	BAK1 MAPK9 PIK3CB PLD1 ARAF MAPK1 E2F1 CDK6 TGFBR1 E2F3 PIK3CA CDKN1A RAC2 ARHGEF6 CASP9 KRAS EGF TP53 SMAD4 EGFR CDKN2A BRAF TGFA TGFBR2 STAT3 BCL2L1 SMAD2
http://www.genome.jp/kegg-bin/show_pathway?hsa05206	MicroRNAs in cancer	57	161	35.40%	1.66028	1.28E-03	CD44 BAK1 PIK3CB RHOA ROCK1 HDAC4 TP63 ABCB1 EZR ABL1 CRKL MAPK1 DICER1 E2F1 UBE2I BMF FZD3 CDK6 MET E2F3 FOXP1 TNR DNMT3A TNN PIK3CA CDKN1A MAP2K2 BCL2L2 PAK4 KRAS NOTCH2 MYC RDX SLC7A1 TP53 MCL1 EFNA3 EGFR CDCA5 CDKN2A ATM HMGA2 WNT3A MMP16 IGF2BP1 STAT3 IRS1 ZEB2 ZFPM2 BCL2 CCNE2 EFNA5 HDAC2 MDM4 TRIM71 NRAS ITGB3
http://www.genome.jp/kegg-bin/show_pathway?hsa05412	Arrhythmogenic right ventricular cardiomyopathy	27	77	35.06%	1.64439	2.98E-02	ITGA3 CACNG3 CTNNA1 DSG2 PKP2 CACNB1 ATP2A3 ITGB5 SLC8A3 ITGB8 SGCA ITGA4 CACNG8 ITGA9 TCF7L2 TCF7L1 CACNA1D CACNB2 CACNG2 SGCD JUP ATP2A2 CACNB4 SLC8A1 CTNNA3 LAMA2 ITGB3
http://www.genome.jp/kegg-bin/show_pathway?hsa05215	Prostate cancer	34	97	35.05%	1.64377	1.48E-02	IGF1 PIK3CB ARAF HSP90AA1 GSK3B MAPK1 E2F1 E2F3 CREB1 PIK3CA CDK2 CDKN1A MAP2K2 CASP9 KRAS EGF IGF1R PDPK1 TP53 PDGFC EGFR TCF7L2 MMP3 TCF7L1 BRAF TGFA SPINT1 HSP90B1 AR PDGFD BCL2 CCNE2 CREB3L2 NRAS
http://www.genome.jp/kegg-bin/show_pathway?hsa05223	Non-small cell lung cancer	25	72	34.72%	1.62832	3.92E-02	BAK1 PIK3CB ARAF MAPK1 STK4 E2F1 CDK6 MET E2F3 PIK3CA CDKN1A STAT5A MAP2K2 CASP9 KRAS EGF PDPK1 TP53 EGFR CDKN2A BRAF TGFA STAT3 KIF5B NRAS
http://www.genome.jp/kegg-bin/show_pathway?hsa05202	Transcriptional misregulation in cancer	66	192	34.38%	1.61204	1.09E-03	ETV1 PAX7 IGF1 BAK1 TRAF1 CDK14 HOXA9 RUNX1T1 MEF2C CCNT2 SIX4 FLT1 EYA1 DOT1L MET DDX5 NSD2 KLF3 ZBTB16 NR4A3 CDKN2C ATF1 CDKN1A RUNX2 MAX BCL11B CCNT1 PER2 H3-3B MEN1 LMO2 CDK9 MYC BMP2K ETV6 DUSP6 IGF1R PML NCOR1 SS18 TP53 MEIS1 CCNA2 IGFBP3 ATM HMGA2 MMP3 ELK4 REL TGFBR2 HPGD SPINT1 SIN3A CXCL8 BCL2L1 MLLT3 AFF1 JUP MLF1 MAF SP1 PBX1 MITF SUPT3H HDAC2 H3C10
http://www.genome.jp/kegg-bin/show_pathway?hsa04015	Rap1 signaling pathway	72	210	34.29%	1.60785	6.22E-04	IGF1 MAP2K3 FLT4 PIK3CB GNAI3 RHOA FGF10 APBB1IP ADCY2 MAGI3 GNAS ANGPT2 CRKL MAPK1 ANGPT4 FGF9 FLT1 PRKD2 MET MAP2K6 RAPGEF2 MAPK14 PRKD3 RAP1A CNR1 ADCY7 PIK3CA VASP MAP2K2 RAP1B RAC2 KRAS RAPGEF5 FGF5 EGF FGF7 IGF1R EFNA3 PDGFC EGFR LPAR4 PARD3 PLCB3 RASGRP3 ANGPT1 ENAH MAPK13 KIT BRAF VAV2 PRKCI ADCY1 P2RY1 PDGFD INSR LPAR3 TLN2 F2R PLCB1 GRIN2A PRKD1 EFNA5 LPAR5 FGF3 MAGI2 EVL SIPA1L1 CTNND1 CALM1 NRAS ITGB3 GRIN2B
http://www.genome.jp/kegg-bin/show_pathway?hsa01521	EGFR tyrosine kinase inhibitor resistance	27	79	34.18%	1.60276	3.82E-02	IGF1 PIK3CB ARAF GSK3B JAK2 MAPK1 MET GAB1 PIK3CA MAP2K2 KRAS EIF4E2 EGF IGF1R PDGFC EGFR SHC3 EIF4E BRAF TGFA STAT3 PDGFD BCL2L1 BCL2 EIF4E1B EIF4EBP1 NRAS
http://www.genome.jp/kegg-bin/show_pathway?hsa04110	Cell cycle	43	126	34.13%	1.60041	1.06E-02	CDC27 DBF4 SMC1A MCM6 CDC14B GSK3B CDC6 CDC23 ABL1 CDC7 E2F1 FZR1 CDK6 E2F3 CCND3 TTK SKP1 TFDP2 ORC2 CDKN2C CDKN1A PKMYT1 PCNA MYC TP53 ANAPC11 SMAD4 CCNA2 CDKN2A ATM CHEK1 BUB3 RAD21 ORC5 ANAPC16 WEE1 YWHAB BUB1 YWHAG CDK1 CCNE2 SMAD2 HDAC2
http://www.genome.jp/kegg-bin/show_pathway?hsa04140	Autophagy	48	141	34.04%	1.59645	6.74E-03	CFLAR LAMP2 RRAGD DAPK2 MAPK9 PIK3CB VMP1 ATG2B PIK3C3 SH3GLB1 SNAP29 MAPK1 MTMR3 PPP2CA RRAGC PIK3CA ATG4C ATG14 MAP2K2 IRS4 KRAS MAP3K7 TANK IGF1R MAP1LC3B PDPK1 ATG3 RUBCN ATG12 C9orf72 DEPTOR RRAGA PRKAA2 WDR41 TSC1 ZFYVE1 RAB8A ATG16L2 IRS1 BCL2L1 BCL2 TRAF6 BNIP3 ERN1 HMGB1 PIK3R4 DAPK1 NRAS
http://www.genome.jp/kegg-bin/show_pathway?hsa05224	Breast cancer	50	147	34.01%	1.59509	6.45E-03	IGF1 BAK1 FLT4 PIK3CB FGF10 ARAF PGR GSK3B WNT11 ESR1 MAPK1 JAG1 E2F1 FGF9 FZD3 CDK6 E2F3 CSNK1A1 PIK3CA CDKN1A MAP2K2 KRAS WNT2B NOTCH2 HEY2 MYC FGF5 EGF ESR2 FGF7 IGF1R TP53 EGFR SHC3 TCF7L2 TCF7L1 WNT3A WNT7A KIT BRAF WNT4 HEYL FZD6 FOS FZD8 JUN JAG2 SP1 FGF3 NRAS
http://www.genome.jp/kegg-bin/show_pathway?hsa05226	Gastric cancer	50	148	33.78%	1.58431	6.72E-03	BAK1 CTNNA1 PIK3CB FGF10 ARAF GSK3B ABCB1 WNT11 MAPK1 E2F1 FGF9 FZD3 MET TGFBR1 GAB1 E2F3 CSNK1A1 PIK3CA CDK2 CDKN1A MAP2K2 KRAS REG4 WNT2B MYC FGF5 EGF FGF7 TP53 SMAD4 EGFR SHC3 TCF7L2 TCF7L1 WNT3A WNT7A BRAF WNT4 TGFBR2 SHH FZD6 CDX2 BCL2 JUP CCNE2 SMAD2 FZD8 CTNNA3 FGF3 NRAS
http://www.genome.jp/kegg-bin/show_pathway?hsa04360	Axon guidance	61	181	33.70%	1.58046	2.54E-03	SEMA3F PIK3CB GNAI3 RHOA ROCK1 EPHA8 TRPC5 SEMA3C PLXNA2 PAK3 SEMA5B GSK3B SEMA4G ABL1 NRP1 MAPK1 BMP7 FZD3 MET CXCL12 UNC5B SEMA5A PIK3CA CXCR4 RAC2 PAK4 TRPC4 KRAS EPHA7 RGS3 PDPK1 SSH2 EFNA3 GDF7 SLIT2 EPHA5 RASA1 PARD3 ENAH UNC5D WNT4 NTNG1 PLXNB1 SHH CFL2 SEMA4C ROBO1 SSH3 ABLIM3 SLIT3 EFNA5 ROBO2 PTCH1 PPP3R2 SRGAP3 ARHGEF12 SRGAP1 LRIG2 NRAS PPP3R1 PLXNA4
http://www.genome.jp/kegg-bin/show_pathway?hsa05231	Choline metabolism in cancer	33	98	33.67%	1.57914	2.51E-02	MAPK9 PIK3CB DGKG SLC44A1 PLD1 MAPK1 DGKH SLC22A2 LYPLA1 PIK3CA MAP2K2 RAC2 SLC44A2 WASF3 KRAS DGKB EGF PDPK1 DGKQ PDGFC EGFR DGKE DGKI PCYT1A TSC1 FOS PDGFD SLC22A1 JUN SP1 EIF4EBP1 PLA2G4E NRAS
http://www.genome.jp/kegg-bin/show_pathway?hsa04934	Cushing syndrome	52	155	33.55%	1.57328	6.70E-03	GNAI3 ADCY2 GSK3B WNT11 GNAS MAPK1 CACNA1I E2F1 FZD3 CDK6 CRHR2 AHR E2F3 ATF2 RAP1A CREB1 ADCY7 CDKN2C ITPR2 CDKN1A MAP2K2 RAP1B PDE11A AIPL1 ASH2L MEN1 WNT2B ARMC5 NCEH1 EGFR CDKN2A TCF7L2 PLCB3 TCF7L1 WNT3A WNT7A CACNA1D BRAF WNT4 ADCY1 FZD6 KMT2D CCNE2 FZD8 CREB3L2 PLCB1 SP1 PBX1 CACNA1H WDR5B HSD3B1 ORAI1
http://www.genome.jp/kegg-bin/show_pathway?hsa04012	ErbB signaling pathway	28	84	33.33%	1.56319	4.34E-02	MAPK9 PIK3CB PAK3 ARAF GSK3B ABL1 CRKL MAPK1 GAB1 CBL PIK3CA CDKN1A STAT5A MAP2K2 PAK4 KRAS MYC EGF ABL2 EGFR SHC3 BRAF TGFA NRG4 JUN ERBB4 EIF4EBP1 NRAS
http://www.genome.jp/kegg-bin/show_pathway?hsa00230	Purine metabolism	42	126	33.33%	1.56319	1.42E-02	ENPP4 AK2 RRM2B ADCY2 ADA2 PDE4D PDE1A NT5C1A ADCY7 PAICS PPAT PDE11A NTPCR ENTPD1 ATIC AK3 PDE3B GUCY1A2 PDE1C AK9 DCK PDE6D AK4 GMPS GUCY1A1 ADCY1 NUDT2 NUDT5 RRM1 ENTPD3 ADPRM NUDT9 PDE7B RRM2 NME6 PDE3A PFAS ENTPD5 ENPP1 PNP PDE7A ADSL
http://www.genome.jp/kegg-bin/show_pathway?hsa04350	TGF-beta signaling pathway	31	93	33.33%	1.56319	3.41E-02	DCN RHOA ROCK1 BAMBI MAPK1 BMP7 SMAD7 PPP2R1A TGFBR1 SMURF2 SKP1 PPP2CA SMAD5 ACVR2B BMP8B TGIF2 SMAD9 ACVR2A FMOD BMP2 MYC PPP2R1B SMAD6 SMAD4 GDF7 TGFBR2 PITX2 FBN1 INHBC SMAD2 SP1
http://www.genome.jp/kegg-bin/show_pathway?hsa04611	Platelet activation	41	124	33.06%	1.55058	1.58E-02	BTK PIK3CB PPP1R12A GNAI3 RHOA ROCK1 APBB1IP ADCY2 GNAS SNAP23 PTGS1 MAPK1 MAPK14 RAP1A ADCY7 PIK3CA ITPR2 VASP RAP1B PIK3R5 FCGR2A PLCB3 GUCY1A2 MAPK13 ARHGAP35 PRKCI GUCY1A1 COL1A2 ADCY1 NOS3 P2RY1 TLN2 RASGRP1 F2R PLCB1 PPP1CC PLA2G4E ARHGEF12 LYN ITGB3 ORAI1
http://www.genome.jp/kegg-bin/show_pathway?hsa04071	Sphingolipid signaling pathway	39	119	32.77%	1.53692	2.23E-02	BID MAPK9 PIK3CB GNAI3 TNFRSF1A RHOA ROCK1 PPP2R3A PLD1 PPP2R5C SPTLC1 MAPK1 SPTLC2 PPP2R1A MAPK14 PPP2CA PIK3CA MAP2K2 TRAF2 RAC2 KRAS PPP2R1B PDPK1 TP53 CERS2 GNA12 PLCB3 PPP2R5E CERS3 MAPK13 SGPP2 NOS3 BCL2 CERS6 SPTLC3 ACER2 PLCB1 NRAS S1PR3
http://www.genome.jp/kegg-bin/show_pathway?hsa05168	Herpes simplex virus 1 infection	162	495	32.73%	1.53477	5.98E-07	ZNF195 ZNF200 BID ZNF582 CD74 FAS BAK1 PIK3CB EIF2AK2 SP100 TNFRSF1A ZNF510 TRAF5 ZNF324 EIF2AK1 SRPK1 JAK2 SRSF5 ZNF175 OAS3 OAS2 EIF2B1 HCFC2 SRSF3 SRSF7 EIF2B2 ZNF549 PILRB PIK3CA SRSF6 TNFSF14 TRAF2 ZNF426 ZNF331 ZNF227 ZNF141 TRAF3 ZNF304 CASP9 MAP3K7 SRSF1 ZFP37 ZNF189 PML ZSCAN32 TP53 IFNAR1 ZNF614 POU2F1 RBAK ZNF157 IFNA16 ZNF547 ZNF773 ZNF689 ZNF19 ZNF230 ZNF222 ZNF208 ZNF333 SRSF2 ZNF577 ZNF589 TLR3 CGAS ZNF12 TSC1 PDIA3 ZNF91 ZNF23 ZNF226 ZNF641 ZNF528 ZNF283 ZNF597 ZNF77[illegible]
http://www.genome.jp/kegg-bin/show_pathway?hsa04218	Cellular senescence	51	156	32.69%	1.53313	1.06E-02	MRE11 MAP2K3 PIK3CB HIPK2 FBXW11 TRPM7 MAPK1 E2F1 CDK6 SERPINE1 TGFBR1 MAP2K6 HIPK3 MAPK14 E2F3 CCND3 RAD1 PIK3CA ITPR2 CDK2 CDKN1A MAP2K2 KRAS MYC TP53 CCNA2 IGFBP3 CDKN2A ATM CHEK1 SLC25A4 ZFP36L2 MAPK13 CACNA1D RBBP4 MAPKAPK2 TGFBR2 TSC1 BTRC CXCL8 CDK1 CCNE2 SMAD2 LIN9 PPP1CC EIF4EBP1 PPP3R2 LIN54 CALM1 NRAS PPP3R1
http://www.genome.jp/kegg-bin/show_pathway?hsa04010	MAPK signaling pathway	96	294	32.65%	1.53129	3.19E-04	CACNG3 PPP5C MAP4K3 IGF1 FAS MAP2K3 FLT4 MAPK9 RASGRF1 TNFRSF1A CACNB1 FGF10 MAP4K4 RPS6KA2 RPS6KA6 ARAF MEF2C MAP3K20 ANGPT2 MKNK2 CRKL MAPK1 CACNA1I PPM1A STK4 ANGPT4 FGF9 FLT1 MET TGFBR1 DUSP3 MAP2K6 RAPGEF2 MAPK14 RASGRF2 MAPKAPK3 ATF2 RAP1A MAX HSPA2 MAP2K2 TRAF2 RAP1B RAC2 KRAS MAP3K7 MYC ARRB1 PPM1B FGF5 EGF DUSP6 FGF7 IGF1R TP53 CACNG8 EFNA3 PDGFC RASA1 GNA12 EGFR NTRK2 TAOK2 RASGRP3 PTPRR ANGPT1 MAPK13 CACNA1D KIT BRAF ELK4 TAOK1 MAPKAPK2 TGFA TGF[illegible]
http://www.genome.jp/kegg-bin/show_pathway?hsa04120	Ubiquitin mediated proteolysis	46	141	32.62%	1.52993	1.42E-02	CDC27 PIAS1 NEDD4 UBE2D1 RHOBTB1 FBXW11 UBE2K UBE2D4 CDC23 CDC34 XIAP MGRN1 UBE2I ELOB UBE2W FZR1 UBE2R2 UBE2S TRIM37 SMURF2 UBE2D3 FBXW7 UBE4A CBL SKP1 FBXO2 VHL UBE2Q2 PML ANAPC11 HERC4 UBC TRIP12 ELOC UBE2L6 CUL4B BTRC ANAPC16 UBE2E3 FBXW8 TRAF6 UBE2N UBE2E2 UBE2F UBE2L3 UBE2H
http://www.genome.jp/kegg-bin/show_pathway?hsa05160	Hepatitis C	51	157	32.48%	1.52336	1.12E-02	CFLAR BID NR1H3 FAS BAK1 PIAS1 PIK3CB EIF2AK2 TNFRSF1A ARAF GSK3B EIF2AK1 MAPK1 E2F1 PPP2R1A CDK6 OAS3 OAS2 E2F3 PPP2CA PIK3CA CDK2 CDKN1A MAP2K2 TRAF2 TRAF3 CASP9 KRAS MYC PPP2R1B EGF TP53 IFNAR1 EGFR IFNA16 CLDN8 BRAF CLDN1 CLDN19 TLR3 YWHAB STAT3 YWHAG CYCS TRAF6 CLDN22 CLDN5 CLDN6 IFIT1 PPARA NRAS
http://www.genome.jp/kegg-bin/show_pathway?hsa04144	Endocytosis	81	252	32.14%	1.50736	1.60E-03	CYTH3 VTA1 EHD3 EHD2 SNX1 EPN1 RHOA VPS35 NEDD4 TFRC PLD1 CAPZB AMPH CHMP2B SMAP2 RAB10 SNX5 SH3GLB1 GRK3 CHMP4B EEA1 RAB11A CAV1 WASL TGFBR1 RAB11FIP2 CYTH1 RAB5C SMURF2 CBL ARPC3 RAB35 ACAP2 CHMP3 CAPZA1 DNAJC6 CXCR4 WIPF3 VPS26A ARFGEF2 STAMBP PSD4 HSPA2 EPS15L1 RBSN SPART IL2RA VPS45 STAM ARRB1 ACTR2 GIT2 VPS37B IGF1R PML IQSEC1 EGFR SH3KBP1 SNX12 PARD3 ASAP1 VPS37A PSD3 RAB11FIP1 LDLRAP1 AGAP1 CCR5 AP2M1 TGFBR2 PRKCI RAB8A RAB4A KIF5B HSPA6 TRAF6 SMAD2 IGF2R
